# Second Coordination Sphere Effects on the Mechanistic Pathways for Dioxygen Activation by a Ferritin: Involvement of a Tyr Radical and the Identification of a Cation Binding Site

**DOI:** 10.1002/cbic.202200257

**Published:** 2022-05-23

**Authors:** Chieh‐Chih George Yeh, Thirakorn Mokkawes, Justin M. Bradley, Nick E. Le Brun, Sam P. de Visser

**Affiliations:** ^1^ Manchester Institute of Biotechnology The University of Manchester 131 Princess Street Manchester M1 7DN UK; ^2^ Department of Chemical Engineering The University of Manchester Oxford Road Manchester M13 9PL UK; ^3^ Centre for Molecular and Structural Biochemistry School of Chemistry University of East Anglia Norwich NR4 7TJ UK

**Keywords:** density functional calculations, enzyme catalysis, enzyme mechanisms, inorganic reaction mechanisms, oxygen activation

## Abstract

Ferritins are ubiquitous diiron enzymes involved in iron(II) detoxification and oxidative stress responses and can act as metabolic iron stores. The overall reaction mechanisms of ferritin enzymes are still unclear, particularly concerning the role of the conserved, near catalytic center Tyr residue. Thus, we carried out a computational study of a ferritin using a large cluster model of well over 300 atoms including its first‐ and second‐coordination sphere. The calculations reveal important insight into the structure and reactivity of ferritins. Specifically, the active site Tyr residue delivers a proton and electron in the catalytic cycle prior to iron(II) oxidation. In addition, the calculations highlight a likely cation binding site at Asp_65_, which through long‐range electrostatic interactions, influences the electronic configuration and charge distributions of the metal center. The results are consistent with experimental observations but reveal novel detail of early mechanistic steps that lead to an unusual mixed‐valent iron(III)‐iron(II) center.

## Graphical abstract

**Density functional calculations** on ferritin implicate novel features in the catalytic cycle for H_2_O_2_ synthesis from dioxygen on a diiron center including a cation‐binding site held by Glu_62_ and Asp_65_. The reaction is stepwise with the involvement of an active site Tyr residue that delivers a hydrogen atom through proton‐coupled‐electron‐transfer.

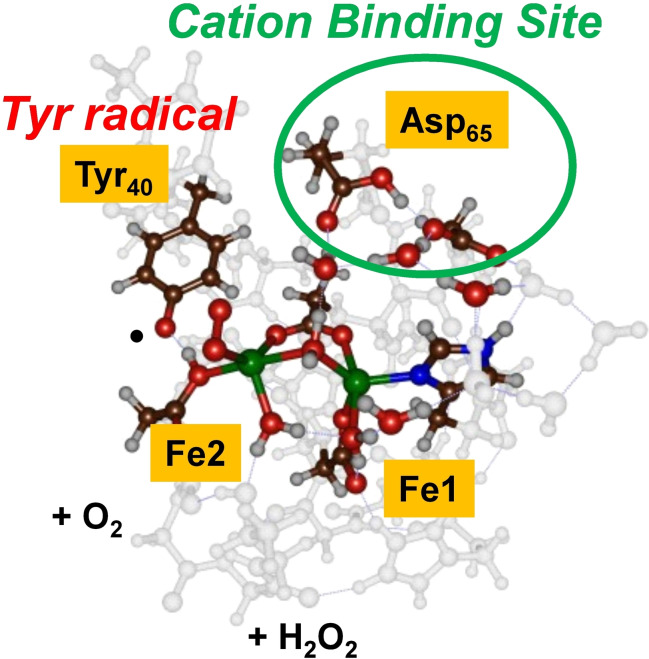

## Introduction

O_2_ activating metalloenzymes are common catalysts in biology and found in all forms of life. Many of these enzymes utilize mononuclear iron in their active site and react as either monooxygenases or dioxygenases.[^1^, ^2^] In addition to these mononuclear iron enzymes, there are a number of nonheme diiron enzymes that activate O_2_ in their catalytic cycles.^[3]^ Among these oxygen activating nonheme diiron enzymes are the soluble methane monooxygenases involved in the hydroxylation of methane in soil,^[4]^ as well as the aldehyde deformylating dioxygenases,^[5]^ and the toluene monooxygenases.^[6]^

Another O_2_ activating nonheme diiron enzyme is ferritin, which has various biological roles and has been implicated in iron(II) detoxification and protection against oxidative stress,^[7]^ in addition to providing an accessible store of iron under conditions of limitation. Ferritins are widely distributed across all kingdoms of life due to their importance in iron homeostasis.^[8]^ Animal ferritins are composed of a mixture of H‐ and L‐ chain subunits that co‐assemble to produce a hetero‐polymeric protein cage in which the H‐chains harbor a diiron site, the ferroxidase center, and the L‐chains do not.[^7a^, ^9^] Ferritins isolated from other organisms are polymers containing only H‐chain like subunits.^[10]^

**Figure 1:**
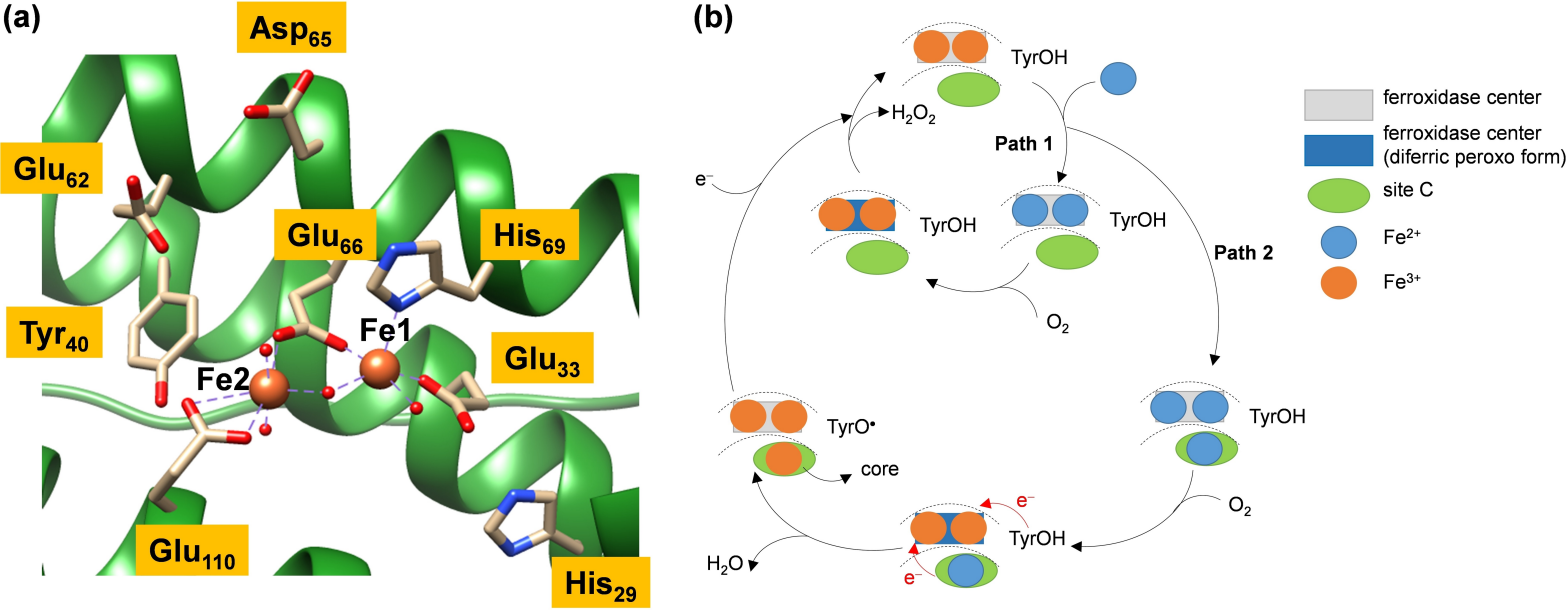
(a) Extract of the diiron active site of ferritin as taken from the 6GKA PDB file. (b) The universal reaction mechanism of dioxygen reduction to hydrogen peroxide or water at the diiron site of ferritins through pathway 1 (independent of site C occupation) and pathway 2 (with Fe^2+^ occupation at site C).

Structural models derived from high resolution X‐ray diffraction data are now available for many ferritins. These reveal an identical 4 α‐helical fold in all examples and similar coordination of the metal ions at the ferroxidase center in the H‐chain proteins, illustrated in Figure 1a. The two ions are bridged by the carboxylate group of a glutamate residue (Glu_66_). Studies in which ferrous iron exposure times were varied identified one high‐affinity (Fe1) and one low‐affinity iron site (Fe2).^[11]^ The coordination of the high affinity Fe1 site is completed by the sidechain of a second carboxylate (Glu_33_), a histidine (His_69_) and a water molecule. The lower affinity Fe2 site is coordinated only by a second carboxylate (Glu_110_) and a water molecule. A strictly conserved tyrosine residue (Tyr_40_) is located close to, but not within bonding distance of, Fe2 and has been identified as important in the mechanism of several ferritins.^[12]^

The similarity of protein structure together with the identification of a common reaction intermediate and product^[13]^ has been invoked to argue in favor of a ‘universal mechanism’ of ferritin activity involving 2 reaction pathways.^[14]^ In pathway 1, dioxygen binds to a diiron(II) center and is converted into H_2_O_2_, concomitant with oxidation of the diiron center to Fe^3+^ (Figure 1b). An alternative dioxygen activation channel (through pathway 2, Figure 1b) requires binding and oxidation of 3 equivalents of Fe^2+^ together with oxidation of the active site Tyr residue (Tyr_40_) coupled to the reduction of dioxygen to water. However, this proposal remains controversial for several reasons. Prokaryotic ferritins contain a third iron binding site, site C, close to the catalytic center that is clearly defined in crystal structures of proteins exposed to iron.^[15]^ Disruption of this binding site by mutagenesis has been shown to affect catalysis in prokaryotic ferritins^[16]^ and the binding of a third equivalent of iron is invoked in the universal mechanism under high iron loadings. In the H‐chain like proteins of prokaryotes three conserved glutamate residues act as ligands to site C. One of these acts as a bridging ligand between site C and the low affinity site of the ferroxidase center. In contrast there is considerable variation in the equivalent residue of the true H‐chains, with a non‐coordinating side chain at this position in many instances (Figure S1, Supporting Information). It has been shown that the site C of prokaryotic ferritins can be disrupted by using site directed mutagenesis to introduce a non‐coordinating residue in place of Glu_52_ calling into question the existence of such a site in the animal H‐chains.[^16^, ^17^]

In the universal mechanism both site C and the conserved tyrosine residue are proposed to play identical roles in all ferritins.^[12c]^ However, the consequences of substitution of Tyr for the non‐oxidizable Phe or of a site C ligand for a non‐coordinating residue vary between proteins. It is also known that the stoichiometry of ferritin catalyzed iron‐O_2_ reaction varies between the extremes of 2 : 1 and 4 : 1 for different proteins.^[10]^ Consequently the view persists that a degree of mechanistic diversity exists within the ferritin family, perhaps reflecting variation in primary role between iron storage and oxidative stress response but also the environmental niche occupied by the organism in which the protein was identified.^[7b]^

*Syn*Ftn is a ferritin isolated from the prokaryotic marine cyanobacterium *Synechoccocus* sp. CC9311 but bears some resemblance to the H‐chain subunits of the animal proteins. Specifically the three glutamate residues that comprise site C are absent from the peptide chain with a serine residue, Ser_146_, in place of the glutamate that bridges site C and the low affinity site of the ferroxidase center in other prokaryotic ferritins. Structural models derived from X‐ray diffraction data from iron enriched crystals revealed only two areas of electron density associated with metal ion binding at *Syn*Ftn ferroxidase centers and that Ser_146_ does not act as a ligand to the low affinity site.^[12a]^ Therefore, the first coordination sphere of iron bound to the ferroxidase center of *Syn*Ftn is identical to that reported for animal H‐chains. Indeed, one of the PDB entries for iron enriched *Syn*Ftn crystals, 6GKA,[^12a^, ^18^] was used to produce the illustration in Figure 1. The first detectable intermediate in *Syn*Ftn reactivity is a previously unreported mixed valent iron(II)/iron(III) ferroxidase center together with a protein based radical assigned to the side chain of Tyr_40_. The diiron(III)‐peroxo species thought to be common to all other ferritins and related diiron enzymes^[19]^ was not reported as a reaction intermediate for this protein. *Syn*Ftn reactivity therefore appears to contradict one of the central arguments in favor of a universal mechanism of ferritin function ‐ similarity in structure inevitably results in similarity of reaction mechanism.

Several computational studies on ferritin and analogous non‐heme O_2_‐activating diiron enzymes have been reported.^[20]^ However, most of these studies used small cluster models or QM/MM with a modest QM region. To be specific, none of the reported computational studies included the Tyr_40_ residue in the models and hence no pathway was ever studied that includes the involvement of this residue. To gain insight into the mechanism of ferritin activity we conducted a computational study into the various possible reaction mechanisms, including the use of large active site models to probe second coordination sphere effects. The latter is relevant because in recent work we showed the importance of the second‐coordination sphere in quantum chemical calculations, whereby the long‐range electrostatic, dipole and electric field perturbations influence optimized geometries and energetics.^[21]^ Our calculations indicate that Tyr_40_ is rapidly oxidized and deprotonated upon O_2_ binding and enables facile reduction of O_2_ to H_2_O_2_ on the diiron center. Our studies also imply an important role of the second‐coordination sphere Asp_65_ residue that can hold a cation or may be protonated during the catalytic cycle.

## Results and Discussion

### Model set‐up and validation

**Figure 2:**
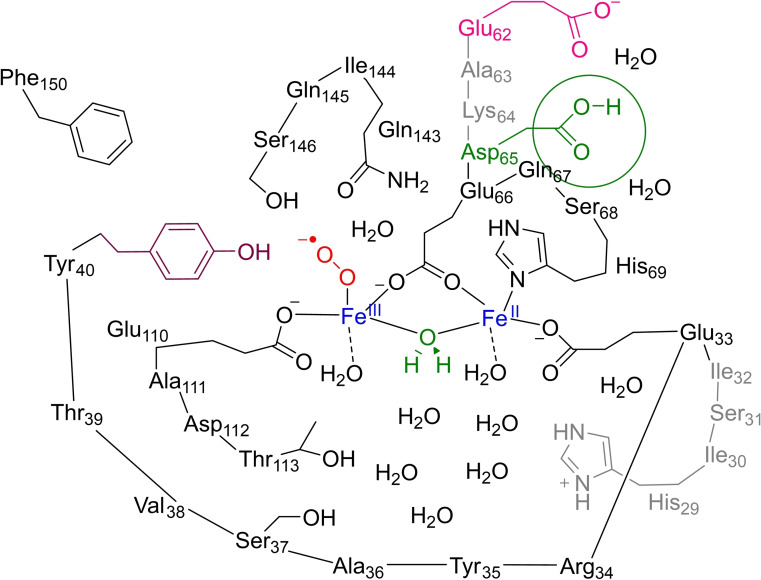
Ferritin model **B** and **BP** as investigated in this work. In model **BP** the Asp_65_ group is protonated, while it is deprotonated in model **B**.

A large active‐site cluster model was created based on the structure of *Syn*Ftn as deposited in the 6GKA PDB file (see Figure 2).[^12a^, ^18^] Dioxygen was added to the Fe2 atom and hydrogen atoms and solvent (water) added in Chimera.^[22]^ The model includes the 4 α‐helical chains around the diiron center with all residues pointing outward as truncated to Gly residues. In addition, a number of polar and charged residues that potentially can induce a local dipole moment or act as part of the hydrogen bonding network have been taken into consideration. The full reaction mechanism was calculated for model **B** and **BP**, which differ in the protonation state of Asp_65_: namely, it is protonated in model **BP** while deprotonated in model **B**. Thus, a p*K*_a_ analysis in PropKa predicts Glu_62_ and Asp_65_ to have deprotonated amino acid chains. However, based on a visual inspection of the structure, we reasoned that since these anionic groups point toward each other that they may be part of a cation binding site although no cation is found in the crystal structure. Therefore, both models **B** and **BP** were tested here. A further expanded model **C** of 439 atoms was tested with Asp_65_ deprotonated, but gave the same structure and electronic configuration as model **B**.

### Geometry optimization of large cluster models

Following the manual addition of a dioxygen group bound to one of the Fe^2+^ ions (Fe2) of the ferroxidase center (see model setup in Methods section), geometry optimization leads to essentially identical end‐on superoxo structures for all residues conserved in each of the large cluster models (**B**, **BP** and **C**). This is due to the presence of a strong hydrogen bonding network included in the models that restrains the systems and maintains the site of iron and oxygen binding in a rigid conformation. An overlay of the optimized geometry of ^11^ **Re**_B_ with the crystal structure coordinates deposited under 6GKA PDB file puts most protein chains in virtually identical positions and retains the basic features of the biochemical system (Figure S14, Supporting Information). The superoxo group is hydrogen bonded to both the side chain of Gln_143_ and the alcohol group of Ser_146_, while the phenol group of Tyr_40_ forms a hydrogen bond with the carboxylate of Glu_110_. Overall, the extra‐large model **C** resulted in the same fold and electronic configuration and structural features as model **B**. We therefore continued the work with model **B** and **BP** only.

### Calculations on unprotonated Asp model B

As previous computational studies on diiron complexes showed that they contain many close‐lying spin and electronic configurations in the various complexes,[^20^, ^23^] we tested the reaction mechanism on multiple spin state surfaces in various possible configurations for each complex. For all spin states for model **B**, optimization resulted in a small redistribution of electrons, giving a diiron(II)‐superoxo complex with an unpaired electron on the superoxo group, as well as an unpaired electron shared between the carboxylate groups of Glu_62_ and Asp_65_ in the orbital labeled as π*_Asp_. Note, that in none of the reactant complexes, **Re**_B_ or **Re**_C_, was spin density found on the Tyr_40_ residue. Attempts were made to swap molecular orbitals and find an iron(III)‐superoxo with closed‐shell deprotonated Asp_65_ instead; however, during the SCF convergence the system returned to an Asp_65_ radical instead. Consequently, in our model without cation bound to Glu_62_ and Asp_65_ and both residues in their deprotonated form, an electron transfer happens from these carboxylates to the iron center that creates an electronic configuration with an Asp radical as the lowest energy structure for model **Re**_B_. This is unusual in bioinorganic chemistry as no experimental evidence for Asp radicals has ever been found in ferritin proteins. As such, model **B** may be a spurious result due to a missing charge and may not be a realistic model of ferritin enzymes. We conclude, therefore, that we expect that in reality Asp_65_ is probably protonated or binds a cation.

To find out if system **B** with deprotonated Glu_62_ and Asp_65_ would be catalytically active, we nevertheless, as a test of principle, investigated the potential energy landscape of dioxygen conversion into H_2_O_2_ using ferritin model **B** on the lowest energy singlet, triplet, quintet, septet, nonet and undecaplet spin states. In all spin states there was radical character on Asp_65_ (Table S10, Supporting Information) in most configurations along the mechanism with ^7^ **Re**_B_ as the lowest in energy. The reaction for model **B** starts from a diiron(II)‐superoxo structure by proton abstraction from the bridging water molecule to form an iron(II)‐iron(III)‐hydroperoxo species with barriers below 11 kcal mol^−1^. As the p*K*_a_ of an ionized Tyr residue is low (p*K*_a_=−2),^[24]^ a proton coupled electron transfer from Tyr_40_ transfers a proton to Glu_110_ while an electron is moved from Tyr_40_ to the diiron system to form a diiron(II)‐hydroperoxo species with a barrier of 15.2 kcal mol^−1^ above reactants (Figure S16, Supporting Information). A final proton transfer from Glu_110_ shuttles the proton to the hydroperoxo group to form H_2_O_2_ in a slightly overall endothermic reaction by 5.1 kcal mol^−1^. As the highest barrier along the calculated mechanism is only 19.9 kcal mol^−1^ this implies that model **B** could be reactive and convert dioxygen to H_2_O_2_ on the diiron(II) center of ferritin despite showing an unusual radical center on Asp_65_. However, the structural orientation of the side chains of Asp_65_ and Glu_110_ imply space for a cation to bind there. As such, we think it more likely that Asp_65_ is either protonated or bears a cation during the reaction mechanism. Nevertheless full details of the predicted transition states and intermediates for the unprotonated Asp model **B** and a discussion on the calculations are given in the Supporting Information.

### Calculations on Asp protonated model BP

**Figure 3:**
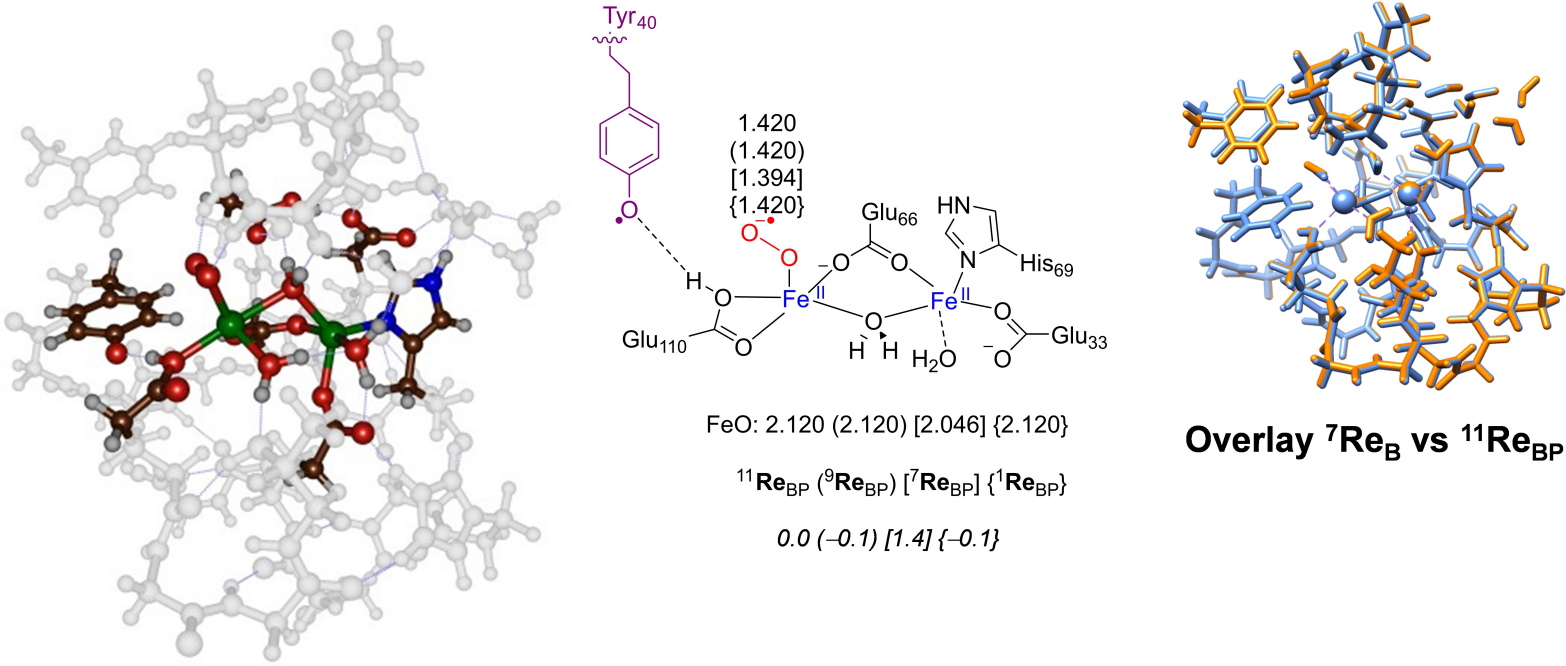
UB3LYP/BS1 optimized geometries of the protonated reactant complexes ^11,9,7^ **Re**_BP_ with bond lengths in angstroms. Relative energies (ΔE+ZPE values) are in kcal mol^−1^. The right‐hand‐side shows an overlay of the ^11^ **Re**_BP_ (light blue) and ^7^ **Re**_B_ (amber) optimized geometries.

Upon visual inspection of the model **B** structures, we decided to repeat some of the calculations with a proton added to the Asp_65_ residue, i. e., Model **BP**. In particular, the Asp_65_ side‐chain of each subunit may either bind a cation such as Na^+^ or K^+^ or even Fe^2+^. Alternatively the side chains of Glu_62_ and Asp_65_ may share a proton during the catalytic cycle of ferritin. While the overall spin state of the reactant complex is the same as that of model **B**, there is an increased charge of +1. The optimized geometries of structures ^11,9,7,1^ **Re**_BP_ have no spin density located on the two carboxylate residues. These optimized geometries are shown in Figure 3 and an overlay of the optimized geometries of ^7^ **Re**_B_ and ^11^ **Re**_BP_ (right‐hand‐side of Figure 3) shows them to be almost identical. Therefore, as expected, the protein fold and second‐coordination sphere are not affected by protonation of Asp_65_ and the characteristic features of the enzyme structure stay the same. The major difference between the ^7^ **Re**_B_ and the **Re**_BP_ structures relates to Tyr_40_, which is deprotonated in **Re**_BP_ and bears an unpaired electron, while it is closed‐shell and protonated in **Re**_B_.

During the geometry optimization of **Re**_BP_ the proton from the phenol group of Tyr_40_ spontaneously moved to Glu_110_. Consequently, Glu_110_ in the ferritin environment is much more basic than Tyr_40_ and accepts its proton rapidly. Attempts to swap molecular orbitals and create an electronic configuration without the Tyr_40_ radical in **Re**_BP_ failed and did not converge. Hence, the lowest energy state has a radical on Tyr_40_. The molecular orbitals of the reactant complexes are shown in Figure S13, Supporting Information. The relevant molecular orbitals mainly originate from the metal 3d sets of orbitals on Fe1 and Fe2 and their interactions with first‐coordination sphere ligands. Overall, all reactant complexes of model BP have an electronic configuration with two iron(II) centers and a radical on superoxo and Tyr_40_, i. e. π*_xy,Fe1_^2^ π*_xz,Fe1_^1^ π*_yz,Fe1_^1^ σ*_z2,Fe1_^1^ σ*_x2‐y2,Fe1_^1^ π*_xy,Fe2_^2^ π*_xz,Fe2_^1^ π*_yz,Fe2_^1^ σ*_z2,Fe2_^1^ σ*_x2‐y2,Fe2_^1^ π*_OO_^1^ π*_Tyr_^1^. The eight unpaired electrons on Fe1/Fe2 are ferromagnetically coupled to the unpaired electrons on the superoxo and Tyr_40_ units in ^11^ **Re**_BP_, while they are antiferromagnetically coupled in ^7^ **Re**_BP_. In ^1^ **Re**_BP_ the electrons on Fe1 and superoxo are up‐spin, whereas those on Fe2 and Tyr_40_ are down‐spin. Due to the same electron distribution in ^1^ **Re**_BP_, ^7^ **Re**_BP_, ^9^ **Re**_BP_ and ^11^ **Re**_BP_ these structures are close in energy and, indeed, we find them all within 1.5 kcal mol^−1^, with ^1^ **Re**_BP_ lowest in energy. As a result equilibration between the four reactant complexes will be possible and all will have a finite lifetime. Moreover, reactivity patterns originating from all reactant complexes can be expected through multistate reactivity.^[19]^ In all structures, the O−O distance is short at 1.420 Å in ^11^ **Re**_BP_, ^9^ **Re**_BP_ and ^1^ **Re**_BP_ while it is 1.394 Å in ^7^ **Re**_BP_.

**Scheme 1:**
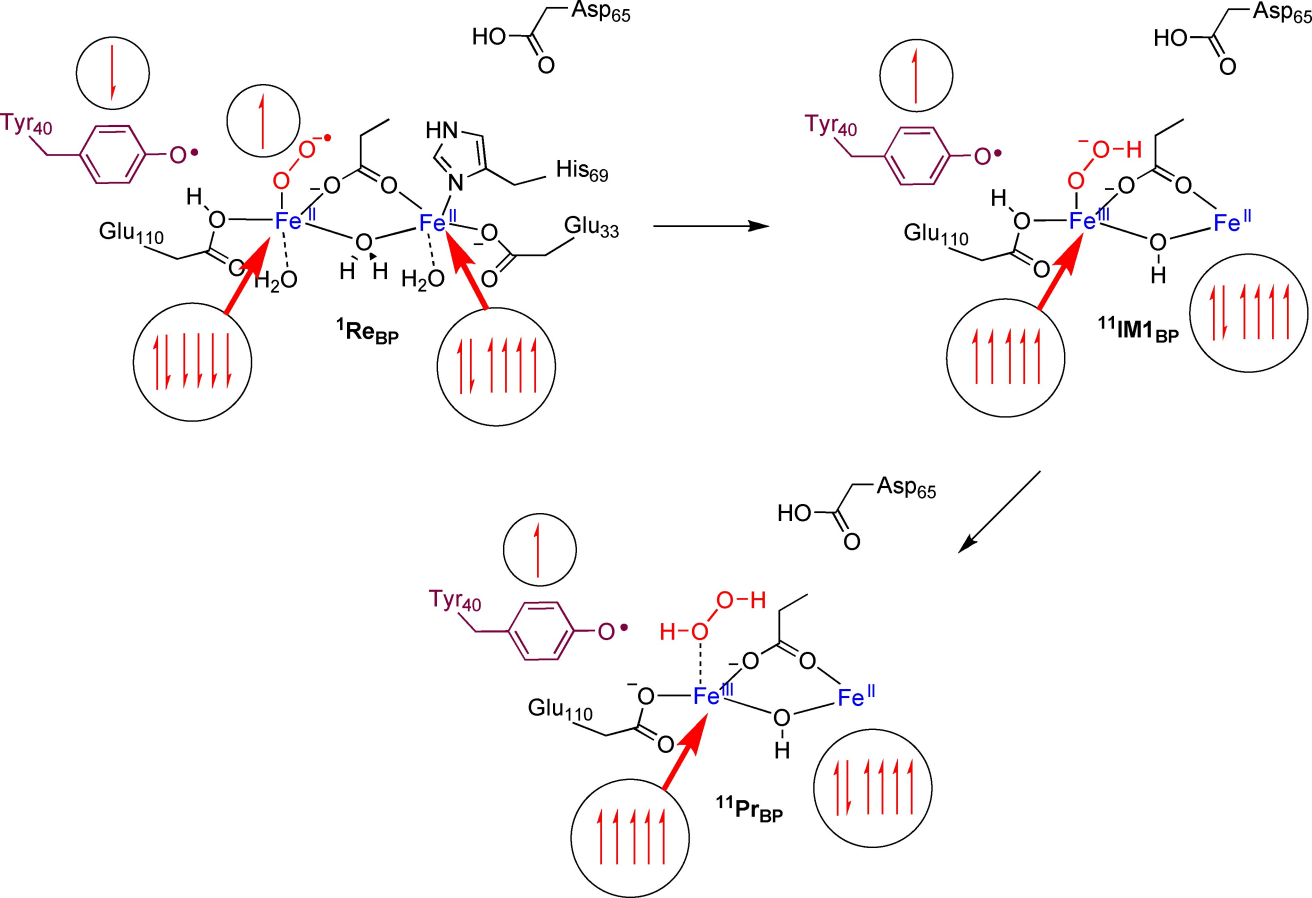
Lowest energy electronic configuration for each intermediate and electron transfer pathways for H_2_O_2_ production from model **Re**_BP_.

**Figure 4:**
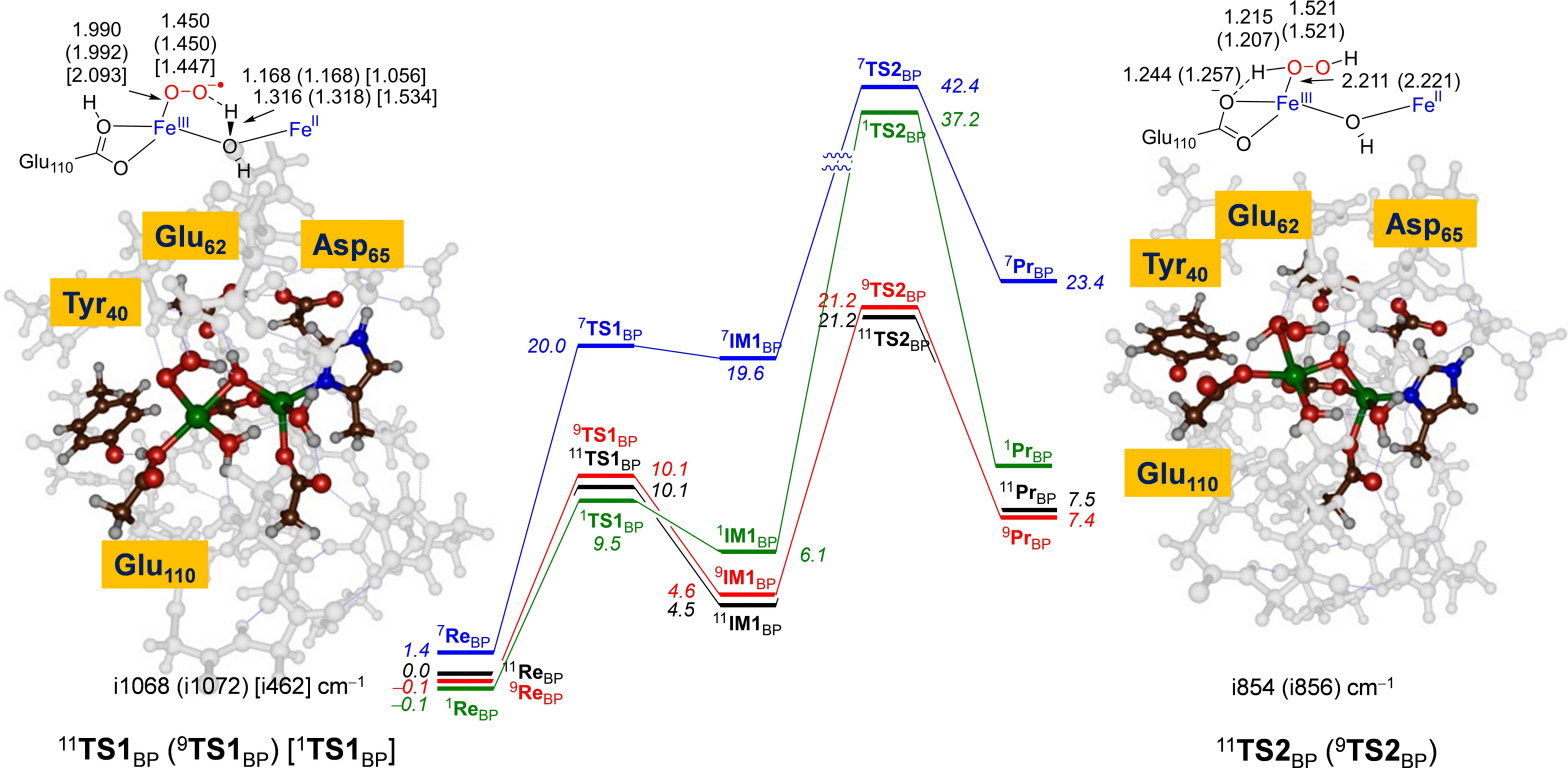
Potential energy profile (ΔE_BS2_+ZPE_BS1_ data in kcal mol^−1^) for dioxygen activation by ferritin models ^11,9,7,1^ **Re**_BP_ as calculated in Gaussian. Optimized geometries of key transition states in the mechanism with bond lengths in angstroms and the imaginary frequency in cm^−1^.

The mechanism of dioxygen conversion into H_2_O_2_ on the diiron center of *Syn*Ftn for model **BP** is illustrated in Scheme 1 and Figure 4. First, proton transfer from the water molecule bridging the two iron ions to the terminal oxygen atom of the iron(II)‐superoxo group occurs, along with electron transfer from the iron(II) to the superoxo group via a transition state **TS1**_BP_. This forms intermediate **IM1**_BP_, which features an iron(II)‐iron(III)‐hydroperoxo species. Barriers for the proton coupled electron transfer are ΔE+ZPE=10.1 (10.1) kcal mol^−1^ on the undecaplet (nonet) spin state surfaces, while the open‐shell singlet spin state is at 9.5 kcal mol^−1^. Energetically these barriers are slightly lower than those for ^11,9^ **TS1**_B_ reported in the Supporting Information Figure S16. The transition states are product‐like with long H−OH distances on the bridging water of 1.316 (1.318) [1.534] Å for ^11^ **TS1**_BP_ (^9^ **TS1**_BP_) [^1^ **TS1**_BP_], while the distance to the terminal oxygen of the superoxo is much shorter: 1.168 Å for both ^11,9^ **TS1**_BP_ and 1.056 Å for ^1^ **TS1**_BP_. The nonet and undecaplet transition states also have relatively large imaginary frequencies, i1068 and i1072 cm^−1^, respectively, for the O−H−O stretch vibration between the peroxo group and the μ‐water ligand, while it is much smaller for the singlet spin structure.

After the transition states, the resulting iron(II)‐iron(III)‐hydroperoxo complex (**IM1**_BP_) retains the radical on Tyr_40_ and a protonated Glu_110_ group. The latter releases its proton to the distal oxygen of the iron(III)‐hydroperoxo species via barriers of 21.2 kcal mol^−1^ on the nonet and undecaplet spin states, while the septet and singlet are much higher lying. The ^9,11^ **TS2**_BP_ structures are shown on the right‐hand‐side of Figure 4. They have imaginary frequencies of i854( i856) cm^−1^ for the undecaplet (nonet) spin states. Both **TS2**_BP_ structures are relatively central, i. e. neither early nor late, with the transferring hydrogen atom almost midway between the donor and acceptor oxygen atom, namely the distances for the donating O−H interaction is 1.244 (1.257) Å and the distances for the accepting O−H interaction is 1.215 (1.207) Å for ^11^ **TS2**_BP_ (^9^ **TS2**_BP_).

The Fe−O distances toward the H_2_O_2_ group have elongated to values above 2.2 Å. Interestingly, the ^1^ **TS2**_BP_ barrier is relatively high in energy and much higher than those on the undecaplet and nonet spin states. The same is true for the singlet spin product complex. This is surprising as the electronic configuration of the singlet, nonet and undecaplet products all have an iron(II)iron(III) complex coupled to a radical on Tyr_40_. However, in the nonet and undecaplet the two iron centers have all metal electrons in up‐spin, while they are antiferromagnetically coupled in the singlet spin state. Moreover, in the singlet spin state the iron(II) center ferromagnetically couples with the tyrosinyl radical, while it is antiferromagnetic with the iron(III) center. Since most metal‐type orbitals are smeared out over the full diiron system this results in a higher singlet spin product complex than the higher‐spin orientations. Several attempts were made to swap orbitals to find a lower energy singlet spin product state, but all converged to the same state shown in Figure 4.

Overall, the potential energy landscapes for dioxygen activation by **Re**_B_ and **Re**_BP_ show similar patterns with a small initial proton coupled electron transfer event with barriers of 9–11 kcal mol^−1^, followed by a larger second barrier of around 20 kcal mol^−1^ for H_2_O_2_ formation. The details of the electronic changes during the reaction mechanism of oxygen activation by the protonated Asp_65_ model **Re**_BP_ are given in Scheme 1. As discussed above, the singlet spin state reactant is the ground state with two antiferromagnetically coupled iron(II) centers coupled to a superoxo and a tyrosyl radical. Upon proton transfer from the μ‐H_2_O group to superoxo a high‐spin state is formed (^11^ **IM1**_BP_) that is built up from an iron(III)‐hydroperoxo group in sextet spin coupled to a quintet spin iron(II) ion and a tyrosyl radical. A final proton transfer from Glu_110_ to iron(III)‐hydroperoxo generates the H_2_O_2_ product and leaves a mixed‐valent Fe(III)‐Fe(II) center with a nearby tyrosyl radical.

The mechanism described for model **Re**_BP_ closely matches experimental data for *Syn*Ftn. Thus, Bradley et al.^[12a]^ detected both a mixed valent iron(III)‐iron(II) ferroxidase center and a tyrosyl radical during the catalytic cycle, consistent with our findings for model **Re**_BP_ and its reaction mechanism. In conclusion, our work points to the mechanism initiating from **Re**_BP_ as the one that best links to experimental work. Even though the mechanism and barriers for the unprotonated model **Re**_B_ are similar to those for model **Re**_BP_, a radical on Tyr_40_ is formed only at the final step leading to a mixed‐valent intermediate and product structure. As such, we believe the Asp_65_ group is either protonated or binds a cation and thereby guides the reaction to mechanism **Re**_BP_.

Interestingly, a recent study of a D65A variant of *Syn*Ftn revealed a significant, unexplained effect on the mechanism of iron(II) oxidation,^[24]^ consistent with the results reported here suggesting an important functional role for this residue in determining the mechanistic path of iron oxidation/O_2_ activation. A survey of ferritin sequences reveals that neither Glu_62_ nor Ser_146_ are conserved within H‐chain peptides (Figure S1, Supporting Information). There is considerable variation in the residues at the position equivalent to 146 whilst that equivalent to position 62 is typically Gln, His or occasionally Ala. The residue equivalent to position 65 is invariably a Glu in the other proteins surveyed. Comparison between coordinate sets deposited in the PDB shows Asp_65_ of *Syn*Ftn to adopt a conformation distinct from the Glu of other ferritins (Figure S1, Supporting Information). The results of our calculations therefore provide a rationale for the unique reactivity reported for *Syn*Ftn with other ferritins utilizing an alternative pathway to O_2_ activation due to the absence of a hydroxyl on residue 146 and a different electronic potential in the region of residues 62 and 65 of the *Syn*Ftn peptide chain.

We also explored possible reaction mechanisms that proceed via a μ‐1,2‐peroxo intermediate. In particular, for the deprotonated Asp_65_ model we located its optimized geometry and found it higher in energy than the lowest reactant complex by ΔE+ZPE=8.5 kcal mol^−1^. Moreover, its formation barrier from a diiron(II)‐peroxo reactant complex was calculated to be well over 25 kcal mol^−1^. This barrier is far higher than the alternative proton coupled electron transfer to form the mixed valent iron(II)‐iron(III)‐hydroperoxo species. Therefore, the μ‐1,2‐peroxo diiron complex will not take part in the catalytic reaction mechanism of *Syn*Ftn and consequently is a side‐product from an alternative higher energy pathway. Our studies match previous computational studies on benzoyl coenzyme A epoxidase,^[23b]^ that also assigned the μ‐1,2‐peroxo diiron(III) complex as a side reaction channel of a higher energy pathway.

## Conclusion

A series of density functional theory calculations on the catalytic cycle of ferritin based on a model of *Syn*Ftn are reported using large cluster models of well over 300 atoms that contain the first‐ and second‐coordination sphere around the diiron center. The calculations suggest that formation of the first experimentally observed reaction intermediate is preceded by several rapid electron and proton transfer events offering new insight into the mechanism of O_2_ activation by ferritin enzymes specifically, and diiron proteins more broadly. Specifically our calculations show that rather than oxidation of Tyr_40_ by an iron(II)‐iron(III)‐superoxo species, as was originally proposed,^[10]^ *Syn*Ftn first forms a diiron(II)‐superoxo species and a tyrosyl radical. The iron(II)‐iron(III)‐hydroperoxo form of the diiron site is then generated via subsequent facile electron and proton transfer processes.

The end point of our calculations here is an iron(II)‐iron(III)‐hydrogen peroxide complex and a Tyr radical. Experimental evidence indicates that the hydrogen peroxide readily dissociates from the ferroxidase center, and that the Tyr radical is rapidly quenched. According to the scheme of Bradley et al., the radical centered on Tyr_40_ is translocated to a remote diiron(II) catalytic center, which has not encountered O_2_, by a series of electron transfer steps, resulting in an iron(III)‐iron(II) ferroxidase center with no hydroperoxo group bound. In this way, the overall reaction of 24 diiron(II) ferroxidase centers with 12 molecules of O_2_ results in 12 molecules of H_2_O_2_ and 24 iron(III)‐iron(II) ferroxidase centers.^[12a]^

The calculations shown in this work highlight a strong hydrogen‐bonding network in the active site that positions the diiron center and dioxygen and enables facile proton transfer. In particular, geometry optimizations indicate that the alcohol group of Ser_146_ stabilizes the superoxo species bound to Fe2 via a hydrogen bonding interaction. We also propose that protonation of, or cation binding between, the carboxylates of Asp_65_ and Glu_62_ is key to determining *Syn*Ftn reactivity. With a proton/cation located on the Asp_65_ group, a mechanism of O_2_ activation is found in which the first proton is delivered by a bridging water molecule, while the second one is shuttled in from Tyr_40_ via Glu_110_. The mechanism produces a mixed‐valent iron(III)‐iron(II) product complex and relies on the nearby Tyr_40_ that forms a tyrosyl radical, in excellent agreement with experimental observation. Overall our studies highlight the importance of second‐coordination sphere effects in the catalytic mechanism of nonheme diiron enzymes, where hydrogen bonding interactions position polar residues and assist with proton‐relay mechanisms. In addition, long‐range electrostatic interactions influence electronic configurations and spin‐state stabilities that are essential for chemical catalysis.

## Experimental Section

**Model set‐up**: We initially ran a 500 ns molecular dynamics (MD) simulation on the ferritin structure starting from the crystal structure coordinates (6GKA PDB file) and with hydrogen atoms added under pH 7 conditions as well as a water layer. The MD simulation (Supporting Information) shows that the structure is highly rigid throughout with a very stable fold and second‐coordination environment. Based on these results, models of the active site of ferritin were created and validated as described previously and followed standard procedures for the set‐up of cluster models.^[26]^ The cluster models were created from the 6GKA PDB file as retrieved from the protein databank.[^12a^, ^18^] The PDB file describes a monomer with both iron atoms included but without dioxygen. Hydrogen atoms were added in Chimera using pH 7 conditions,^[22]^ which assumed all Arg and Lys side‐chains were protonated and all Asp and Glu side‐chains taken to be in their deprotonated forms. The protonation states of all acidic and basic residues were further confirmed with PropKa that also assigned the Glu_62_ and Asp_65_ residues to be deprotonated. However, upon visual inspection of the protein structure we decided to investigate models with Asp_65_ in its deprotonated form (models **A**, **B**, **C**) and in its protonated form (model **BP**). His_29_ was assumed to be doubly protonated, while all other histidine residues were singly protonated. Large active site cluster models were generated that incorporate the first‐, second‐ and third‐coordination sphere of the catalytic center and further studied with density functional theory methods. These models often describe short‐ and long‐range electrostatic interactions well and are the method of choice for enzymatic reaction mechanisms.[^26^, ^27^]

To gain experience with the chemical system, we first explored a small cluster model of 121 atoms (model **A**), see Supporting Information for details. Subsequently, we investigated more realistic models with a large number of second‐ and third‐coordination sphere residues included: Model **B** with 340 atoms and Model **BP** with 341 atoms. Model **B** included the two iron atoms bridged by a water molecule and the carboxylate of Glu_66_ which is part of the peptide chain Glu_62_‐Ala_63_‐Lys_64_‐Asp_65_‐Glu_66_‐Gln_67_‐Ser_68_‐His_69_, whereby the Ala_63_, Lys_64_, Gln_67_ and Ser_68_ residues were truncated to Gly. Another peptide chain incorporated in the model covered the sequence from His_29_ to Tyr_40_. Again, the amino acid side chains pointing away from the active site were truncated to Gly, i. e. Ile_30_, Ser_31_, Ile_32_, Arg_34_, Tyr_35_, Val_38_ and Thr_39_. The Glu_110_ ligand of one of the iron atoms was included as part of the short peptide chain Glu_110_‐Ala_111_‐Asp_112_‐Thr_113_ with the Ala and Asp residues shortened to Gly. A final peptide chain Gln_143_‐Ile_144_‐Gln_145_‐Ser_146_, with the two middle residues without side chains donating hydrogen bonding interactions into the active site. Lastly, the model contains the side chain of Phe_150_ as toluene and eleven water molecules. A dioxygen group was manually added to one of the iron(II) atoms (Fe2) in an end‐on conformation. Our model is overall charge neutral and was investigated with odd spin multiplicity (M=1, 3, 5, 7, 9, 11). To test the reproducibility of the model, we expanded model **B** further by adding Ala_70_‐Val_71_‐His_72_‐Phe_73_, Phe_107_‐Gln_108_‐Met_109_ and Thr_114_ with the side chains of Ala_70_, Val_71_, Phe_107_ and, Gln_108_ truncated to Gly to get model **C** of 439 atoms and minimized its geometry ^11^ **Re**_C_. The structure and electronic configuration of ^11^ **Re**_C_ showed little difference to that of ^11^ **Re**_B_; hence the work was continued with model **B** and **BP** only.

Despite the fact that the Glu_62_ and Asp_65_ side chains are considered to have low p*K*_a_ values, actually their local environment on visual inspection does not show a nearby positive counter charge. Therefore, we investigated a protonated model whereby a proton was added between the two carboxylate side chains of Glu_62_ and Asp_65_: **Re**_BP_. The full mechanism of dioxygen activation on several spin states surfaces was also investigated for model **Re**_BP_. All models were run without geometric constraints and a comparison of optimized geometries with crystal structure coordinates only showed minor deviations.

**Procedures**: Density functional theory (DFT) calculations were performed on the ferritin models using the Gaussian‐09 software package,^[28]^ and utilized the unrestricted B3LYP hybrid density functional method.^[29]^ Geometry optimizations, frequencies, intrinsic reaction coordinate scans and geometry scans were performed with an LANL2DZ basis set on iron with core potential and 6‐31G on the rest of the atoms: basis set BS1.^[30]^ To correct the energies, single point calculations on the optimized geometries were performed with the LACV3P+ basis set on iron with core potential and 6‐311+G* on the rest of the atoms: basis set BS2. All calculations were run with the continuum polarized conductor model (CPCM) included with a dielectric constant mimicking chlorobenzene.^[31]^ For a number of structures, dispersion corrected DFT was tested, but resulted in energies very similar to those obtained without dispersion, in agreement with previous work.^[32]^ The methods used for this work have been extensively tested for analogous systems and reproduce spin‐state orderings, product distributions and free energies of activation well.^[33]^

Transition states were obtained through full geometry optimizations and were characterized with a single imaginary mode for the correct transition. For a selection of transition states we ran intrinsic reaction coordinate scans (IRCs) that confirmed the connection of the TS with the two local minima, see Supporting Information. Free energies were obtained from unscaled vibrational frequencies and entropies at 298 K. All energies reported in this work were obtained using UB3LYP/BS2 with solvent and zero‐point corrections included.

## Conflict of interest

The authors declare no conflict of interest.

1

## Supporting information

As a service to our authors and readers, this journal provides supporting information supplied by the authors. Such materials are peer reviewed and may be re‐organized for online delivery, but are not copy‐edited or typeset. Technical support issues arising from supporting information (other than missing files) should be addressed to the authors.

Supporting Information

## Data Availability

All relevant data are presented in the main paper or in the Supporting Information, and are available from the authors upon reasonable request.

## References

[1a] M. Sono , M. P. Roach , E. D. Coulter , J. H. Dawson , Chem. Rev. 1996, 96, 2841–2888; 11848843 10.1021/cr9500500

[1b] B. Meunier , S. P. de Visser , S. Shaik , Chem. Rev. 2004, 104, 3947–3980; 15352783 10.1021/cr020443g

[1c] I. G. Denisov , T. M. Makris , S. G. Sligar , I. Schlichting , Chem. Rev. 2005, 105, 2253–2277; 15941214 10.1021/cr0307143

[1d] P. R. Ortiz de Montellano , Cytochrome P450: Structure, Mechanism and Biochemistry , 3rd ed., Kluwer/Plenum, New York, 2005;

[1e] A. W. Munro , H. M. Girvan , K. J. McLean , Nat. Prod. Rep. 2007, 24, 585–609; 17534532 10.1039/b604190f

[1f] K. M. Kadish , K. M. Smith , R. Guilard , Handbook of Porphyrin Science, World Scientific, New Jersey, 2010;

[1g] J. Rittle , M. T. Green , Science 2010, 330, 933–937; 21071661 10.1126/science.1193478

[1h] P. R. Ortiz de Montellano , Chem. Rev. 2010, 110, 932–948; 19769330 10.1021/cr9002193 PMC2820140

[1i] X. Huang , J. T. Groves , Chem. Rev. 2018, 118, 2491–2553; 29286645 10.1021/acs.chemrev.7b00373 PMC5855008

[1j] K. D. Dubey , S. Shaik , Acc. Chem. Res. 2019, 52, 389–399; 30633519 10.1021/acs.accounts.8b00467

[1k] N. P. Dunham , F. H. Arnold , ACS Catal. 2020, 10, 12239–12255. 33282461 10.1021/acscatal.0c03606 PMC7710332

[2a] C. J. Schofield , Z. Zhang , Curr. Opin. Struct. Biol. 1999, 9, 722–731; 10607676 10.1016/s0959-440x(99)00036-6

[2b] E. I. Solomon , T. C. Brunold , M. I. Davis , J. N. Kemsley , S. K. Lee , N. Lehnert , F. Neese , A. J. Skulan , Y. S. Yang , J. Zhou , Chem. Rev. 2000, 100, 235–349; 11749238 10.1021/cr9900275

[2c] M. M. Abu-Omar , A. Loaiza , N. Hontzeas , Chem. Rev. 2005, 105, 2227–2252; 15941213 10.1021/cr040653o

[2d] C. Krebs , D. Galonić Fujimori , C. T. Walsh , J. M. Bollinger, Jr. , Acc. Chem. Res. 2007, 40, 484–492; 17542550 10.1021/ar700066p PMC3870002

[2e] E. G. Kovaleva , J. D. Lipscomb , Nat. Chem. Biol. 2008, 4, 186–193; 18277980 10.1038/nchembio.71 PMC2720164

[2f] S. P. de Visser , D. Kumar , Iron-Containing Enzymes: Versatile Catalysts of Hydroxylation Reactions in Nature, Royal Society of Chemistry, Cambridge, 2011;

[2g] M. D. White , E. Flashman , Curr. Opin. Chem. Biol. 2016, 31, 126–135; 27015291 10.1016/j.cbpa.2016.02.017 PMC4879150

[2h] S. P. de Visser , G. Mukherjee , H. S. Ali , C. V. Sastri , Acc. Chem. Res. 2022, 55, 65–74. 34915695 10.1021/acs.accounts.1c00538

[3a] A. J. Jasniewski , L. Que, Jr. , Chem. Rev. 2018, 118, 2554–2592; 29400961 10.1021/acs.chemrev.7b00457 PMC5920527

[3b] A. Trehoux , J.-P. Mahy , F. Avenier , Coord. Chem. Rev. 2016, 322, 142–158.

[4a] C. E. Tinberg , S. J. Lippard , Acc. Chem. Res. 2011, 44, 280–288; 21391602 10.1021/ar1001473 PMC3079780

[4b] K. Yoshizawa , Bull. Chem. Soc. Jpn. 2013, 86, 1083–1116;

[4c] S. Zhang , R. Karthikeyan , S. D. Fernando , Rev. Environ. Sci. Bio/Technol. 2017, 16, 611–623;

[4d] V. C.-C. Wang , S. Maji , P. P.-Y. Chen , H. K. Lee , S. S.-F. Yu , S. I. Chan , Chem. Rev. 2017, 117, 8574–8621; 28206744 10.1021/acs.chemrev.6b00624

[4e] A. C. Ghosh , C. Duboc , M. Gennari , Coord. Chem. Rev. 2021, 428, 213606;

[4f] C. W. Koo , A. C. Rosenzweig , Chem. Soc. Rev. 2021, 50, 3424–3436; 33491685 10.1039/d0cs01291b PMC7965334

[4g] C. E. Schulz , R. G. Castillo , D. A. Pantazis , S. DeBeer , F. Neese , J. Am. Chem. Soc. 2021, 143, 6560–6577. 33884874 10.1021/jacs.1c01180 PMC8154522

[5a] A. Schirmer , M. A. Rude , X. Li , E. Popova , S. B. del Cardayre , Science 2010, 329, 559–562; 20671186 10.1126/science.1187936

[5b] C. Krebs , J. M. Bollinger, Jr. , S. J. Booker , Curr. Opin. Chem. Biol. 2011, 15, 291–303; 21440485 10.1016/j.cbpa.2011.02.019 PMC3113506

[5c] D. M. Warui , N. Li , H. Nørgaard , C. Krebs , J. M. Bollinger, Jr. , S. J. Booker , J. Am. Chem. Soc. 2011, 133, 3316–3319; 21341652 10.1021/ja111607x PMC3069495

[5d] G. E. Cutsail III , E. J. Blaesi , C. J. Pollock , J. M. Bollinger, Jr. , C. Krebs , S. DeBeer , J. Inorg. Biochem. 2020, 203, 110877; 31710865 10.1016/j.jinorgbio.2019.110877 PMC7012765

[5e] U. K. Bagha , J. K. Satpathy , G. Mukherjee , C. V. Sastri , S. P. de Visser , Org. Biomol. Chem. 2021, 19, 1879–1899. 33406196 10.1039/d0ob02204g

[6a] A. Bassan , M. R. A. Blomberg , T. Borowski , P. E. M. Siegbahn , J. Phys. Chem. B 2004, 108, 13031–13041;

[6b] N. L. Elsen , L. J. Bailey , A. D. Hauser , B. G. Fox , Biochemistry 2009, 48, 3838–3846; 19290655 10.1021/bi900144a

[6c] A. D. Bochevarov , J. Li , W. J. Song , R. A. Friesner , S. J. Lippard , J. Am. Chem. Soc. 2011, 133, 7384–7397; 21517016 10.1021/ja110287y PMC3092846

[6d] M. Kaniusaite , R. J. A. Goode , R. B. Schittenhelm , T. M. Makris , M. J. Cryle , ACS Chem. Biol. 2019, 14, 2932–2941. 31774267 10.1021/acschembio.9b00862 PMC6929969

[7a] E. C. Theil , R. K. Behera , T. Tosha , Coord. Chem. Rev. 2013, 257, 579–586; 23470857 10.1016/j.ccr.2012.05.013 PMC3587046

[7b] J. M. Bradley , D. A. Svistunenko , M. T. Wilson , A. M. Hemmings , G. R. Moore , N. E. Le Brun , J. Biol. Chem. 2020, 295, 17602–17623. 33454001 10.1074/jbc.REV120.007746 PMC7762939

[8] E. C. Theil , T. Tosha , R. K. Beherat , Acc. Chem. Res. 2016, 49, 784–791. 27136423 10.1021/ar500469e

[9] W. Wang , Y. Zhang , G. Zhao , H. Wang , Inorg. Chem. 2021, 60, 7207–7216. 33852289 10.1021/acs.inorgchem.1c00265

[10] J. M. Bradley , G. R. Moore , N. E. Le Brun , Curr. Opin. Chem. Biol. 2017, 37, 122–128. 28314217 10.1016/j.cbpa.2017.02.027

[11a] C. Pozzi , F. Di Pisa , D. Lalli , C. Rosa , E. Theil , P. Turano , S. Mangani , Acta Crystallogr. Sect. D 2015, 71, 941–953; 25849404 10.1107/S1399004715002333 PMC4388269

[11b] C. Pozzi , F. Di Pisa , C. Bernacchioni , S. Ciambellotti , P. Turano , S. Mangani , Acta Crystallogr. Sect. D 2015, 71, 1909–1920. 26327381 10.1107/S1399004715013073

[12a] J. M. Bradley , D. A. Svistunenko , J. Pullin , N. Hill , R. K. Stuart , B. Palenik , M. T. Wilson , A. M. Hemmings , G. R. Moore , N. E. Le Brun , Proc. Natl. Acad. Sci. USA 2019, 116, 2058–2067; 30659147 10.1073/pnas.1809913116 PMC6369749

[12b] J. M. Bradley , D. A. Svistunenko , T. L. Lawson , A. M. Hemmings , G. R. Moore , N. E. Le Brun , Angew. Chem. Int. Ed. 2015, 54, 14763–14767; 10.1002/anie.201507486 PMC4691338 26474305

[12c] K. H. Ebrahimi , P. L. Hagedoorn , W. R. Hagen , ChemBioChem 2013, 14, 1123–1133. 23737293 10.1002/cbic.201300149

[13] W. R. Hagen , P. L. Hagedoorn , K. H. Ebrahimi , Metallomics 2017, 9, 595–605. 28573266 10.1039/c7mt00124j

[14] K. H. Ebrahimi , P. L. Hagedoorn , W. R. Hagen , Chem. Rev. 2015, 115, 295–326. 25418839 10.1021/cr5004908

[15] J. Tatur , W. R. Hagen , P. M. Matias , J. Biol. Inorg. Chem. 2007, 12, 615–630. 17541801 10.1007/s00775-007-0212-3 PMC1915633

[16] F. Bou-Abdallah , H. Yang , A. Awomolo , B. Cooper , M. R. Woodhall , S. C. Andrews , N. D. Chasteen , Biochemistry 2014, 53, 483–495. 24380371 10.1021/bi401517f PMC3951517

[17] S. Pfaffen , J. M. Bradley , R. Abdulqadir , M. R. Firme , G. R. Moore , N. E. Le Brun , M. E. P. Murphy , J. Biol. Chem. 2015, 290, 28416–28427. 26396187 10.1074/jbc.M115.669713 PMC4653698

[18] H. M. Berman , J. Westbrook , Z. Feng , G. Gilliland , T. N. Bhat , H. Weissig , I. N. Shindyalov , P. E. Bourne , Nucleic Acids Res. 2000, 28, 235–242. 10592235 10.1093/nar/28.1.235 PMC102472

[19a] A. S. Pereira , W. Small , C. Krebs , P. Tavares , D. E. Edmondson , E. C. Theil , B. H. Huynh , Biochemistry 1998, 37, 9871–9876; 9665690 10.1021/bi980847w

[19b] P. Moënne-Loccoz , J. Baldwin , B. A. Ley , T. M. Loehr , J. M. Bollinger, Jr. , Biochemistry 1998, 37, 14659–14663; 9778340 10.1021/bi981838q

[19c] P. Moënne-Loccoz , C. Krebs , K. Herlihy , D. E. Edmondson , E. C. Theil , B. H. Huynh , T. M. Loehr , Biochemistry 1999, 38, 5290–5295; 10220314 10.1021/bi990095l

[19d] X. Liu , E. C. Theil , Proc. Natl. Acad. Sci. USA 2004, 101, 8557–8562. 15166287 10.1073/pnas.0401146101 PMC423233

[20a] D. E. Bacelo , R. C. Binning, Jr. , Inorg. Chem. 2006, 45, 10263–10269; 17140234 10.1021/ic060388k

[20b] H. Hirao , J. Phys. Chem. B 2011, 115, 11278–11285; 21899302 10.1021/jp2057173

[20c] T. V. Harris , K. Morokuma , Inorg. Chem. 2013, 52, 8551–8563; 23865546 10.1021/ic4006168

[20d] N. Proos Vedin , M. Lundberg , J. Biol. Inorg. Chem. 2016, 21, 645–657; 27364958 10.1007/s00775-016-1374-7

[20e] C. Wang , H. Chen , J. Am. Chem. Soc. 2017, 139, 13038–13046. 28844144 10.1021/jacs.7b06343

[21a] S. P. de Visser , Chem. Eur. J. 2020, 26, 5308–5327; 31804749 10.1002/chem.201905119

[21b] H. S. Ali , S. P. de Visser , Chem. Eur. J. 2022, 28, e202104167. 34967481 10.1002/chem.202104167 PMC9304159

[22] E. F. Pettersen , T. D. Goddard , C. C. Huang , G. S. Couch , D. M. Greenblatt , E. C. Meng , T. E. Ferrin , J. Comput. Chem. 2004, 25, 1605–1612. 15264254 10.1002/jcc.20084

[23a] J. Liu , P. Wu , S. Yan , Y. Li , Z. Cao , B. Wang , ACS Catal. 2021, 11, 6141–6152;

[23b] T. A. Rokob , J. Am. Chem. Soc. 2016, 138, 14623–14638; 27682344 10.1021/jacs.6b06987

[23c] M. Ansari , D. Senthilnathan , G. Rajaraman , Chem. Sci. 2020, 11, 10669–10687; 33209248 10.1039/d0sc02624g PMC7654192

[23d] L. M. Pérez , C. E. Webster , A. A. Low , M. B. Hall , Theor. Chem. Acc. 2008, 120, 467–478;

[23e] W.-L. Han , L. Noodleman , Inorg. Chem. 2008, 47, 2975–2986; 18366153 10.1021/ic701194b

[23f] L. Noodleman , W.-G. H. Du , D. McRee , Y. Chen , T. Goh , A. W. Götz , Phys. Chem. Chem. Phys. 2020, 22, 26652–26668; 33231596 10.1039/d0cp04848h PMC7727307

[23g] L. Wang , M. Gennari , F. G. Cantú Reinhard , J. Gutierrez , A. Morozan , C. Philouze , S. Demeshko , V. Artero , F. Meyer , S. P. de Visser , C. Duboc , J. Am. Chem. Soc. 2019, 141, 8244–8253; 31026148 10.1021/jacs.9b02011

[23h] M. G. Quesne , D. Senthilnathan , D. Singh , D. Kumar , P. Maldivi , A. B. Sorokin , S. P. de Visser , ACS Catal. 2016, 6, 2230–2243;

[23i] C. Colomban , A. H. Tobing , G. Mukherjee , C. V. Sastri , A. B. Sorokin , S. P. de Visser , Chem. Eur. J. 2019, 25, 14320–14331; 31339185 10.1002/chem.201902934

[23j] H. Hirao , L. Que Jr , W. Nam , S. Shaik , Chem. Eur. J. 2008, 14, 1740–1756. 18186094 10.1002/chem.200701739

[24] J. J. Warren , J. R. Winkler , H. B. Gray , FEBS Lett. 2012, 586, 596–602. 22210190 10.1016/j.febslet.2011.12.014 PMC3298607

[25] J. M. Bradley , J. Fair , A. M. Hemmings , N. E. Le Brun , Microbiology 2021, 167, 001105. 10.1099/mic.0.001105 PMC8743623 34825885

[26a] M. R. A. Blomberg , T. Borowski , F. Himo , R.-Z. Liao , P. E. M. Siegbahn , Chem. Rev. 2014, 114, 3601–3658; 24410477 10.1021/cr400388t

[26b] M. G. Quesne , T. Borowski , S. P. de Visser , Chem. Eur. J. 2016, 22, 2562–2581; 26696271 10.1002/chem.201503802

[26c] M. Q. E. Mubarak , E. F. Gérard , C. F. Blanford , S. Hay , S. P. de Visser , ACS Catal. 2020, 10, 14067–14079;

[26d] Q. Cheng , N. J. DeYonker , J. Phys. Chem. B 2021, 125, 3296–3306; 33784103 10.1021/acs.jpcb.0c10761

[26e] D. Li , Y. Wang , K. Han , Coord. Chem. Rev. 2012, 256, 1137–1150;

[26f] C.-C. G. Yeh , S. Ghafoor , J. K. Satpathy , T. Mokkawes , C. V. Sastri , S. P. de Visser , ACS Catal. 2022, 12, 3923–3937.

[27a] X. Sheng , M. Kazemi , F. Planas , F. Himo , ACS Catal. 2020, 10, 6430–6449; 10.1021/acscatal.9b04208 PMC6945686 31929947

[27b] C.-C. G. Yeh , C. Pierides , G. N. L. Jameson , S. P. de Visser , Chem. Eur. J. 2021, 27, 13793–13806; 34310770 10.1002/chem.202101878

[27c] G. Mukherjee , J. K. Satpathy , U. K. Bagha , M. Q. E. Mubarak , C. V. Sastri , S. P. de Visser , ACS Catal. 2021, 11, 9761–9797.

[28] Gaussian-09, Revision D.01, M. J. Frisch, G. W. Trucks, H. B. Schlegel, G. E. Scuseria, M. A. Robb, J. R. Cheeseman, G. Scalmani, V. Barone, B. Mennucci, G. A. Petersson, H. Nakatsuji, M. Caricato, X. Li, H. P. Hratchian, A. F. Izmaylov, J. Bloino, G. Zheng, J. L. Sonnenberg, M. Hada, M. Ehara, K. Toyota, R. Fukuda, J. Hasegawa, M. Ishida, T. Nakajima, Y. Honda, O. Kitao, H. Nakai, T. Vreven, J. A. Montgomery, Jr., J. E. Peralta, F. Ogliaro, M. Bearpark, J. J. Heyd, E. Brothers, K. N. Kudin, V. N. Staroverov, T. Keith, R. Kobayashi, J. Normand, K. Raghavachari, A. Rendell, J. C. Burant, S. S. Iyengar, J. Tomasi, M. Cossi, N. Rega, J. M. Millam, M. Klene, J. E. Knox, J. B. Cross, V. Bakken, C. Adamo, J. Jaramillo, R. Gomperts, R. E. Stratmann, O. Yazyev, A. J. Austin, R. Cammi, C. Pomelli, J. W. Ochterski, R. L. Martin, K. Morokuma, V. G. Zakrzewski, G. A. Voth, P. Salvador, J. J. Dannenberg, S. Dapprich, A. D. Daniels, O. Farkas, J. B. Foresman, J. V. Ortiz, J. Cioslowski, D. J. Fox, Gaussian, Inc., Wallingford CT, **2010**.

[29a] A. D. Becke , J. Chem. Phys. 1993, 98, 5648–5652;

[29b] C. Lee , W. Yang , R. G. Parr , Phys. Rev. B 1988, 37, 785–789. 10.1103/physrevb.37.785 9944570

[30a] P. J. Hay , W. R. Wadt , J. Chem. Phys. 1985, 82, 270–283;

[30b] R. Ditchfield , W. J. Hehre , J. A. Pople , J. Chem. Phys. 1971, 54, 724–728.

[31] J. Tomasi , B. Mennucci , R. Cammi , Chem. Rev. 2005, 105, 2999–3093. 16092826 10.1021/cr9904009

[32a] M. A. Sainna , D. Sil , D. Sahoo , B. Martin , S. P. Rath , P. Comba , S. P. de Visser , Inorg. Chem. 2015, 54, 1919–1930; 25610949 10.1021/ic502803b

[32b] F. G. Cantú Reinhard , A. S. Faponle , S. P. de Visser , J. Phys. Chem. A 2016, 120, 9805–9814; 27973805 10.1021/acs.jpca.6b09765

[32c] T. Yang , M. G. Quesne , H. M. Neu , F. G. Cantú Reinhard , D. P. Goldberg , S. P. de Visser , J. Am. Chem. Soc. 2016, 138, 12375–12386. 27545752 10.1021/jacs.6b05027 PMC5228574

[33a] F. G. Cantú Reinhard , M. A. Sainna , P. Upadhyay , G. A. Balan , D. Kumar , S. Fornarini , M. E. Crestoni , S. P. de Visser , Chem. Eur. J. 2016, 22, 18608–18619; 27727524 10.1002/chem.201604361

[33b] X.-X. Li , V. Postils , W. Sun , A. S. Faponle , M. Solà , Y. Wang , W. Nam , S. P. de Visser , Chem. Eur. J. 2017, 23, 6406–6418. 28295741 10.1002/chem.201700363

