# Supplementary material for: Second Coordination Sphere Effects on the Mechanistic Pathways for Dioxygen Activation by a Ferritin: Involvement of a Tyr Radical and the Identification of a Cation Binding Site
Source: Chembiochem. 2022 May 23;23(13):e202200257. doi: 10.1002/cbic.202200257 (PMC9401865; doi:10.1002/cbic.202200257)
Supplement: Supplementary file 1 — Supporting Information [file CBIC-23-0-s001.pdf]

ChemBioChem

Supporting Information

Second Coordination Sphere Effects on the Mechanistic Pathways for Dioxygen Activation by a Ferritin: Involvement of a Tyr Radical and the Identification of a Cation Binding Site

Chieh-Chih George Yeh, Thirakorn Mokkawes, Justin M. Bradley, Nick E. Le Brun,* and Sam P. de Visser*

Part I: Comparison of various ferritin structures.

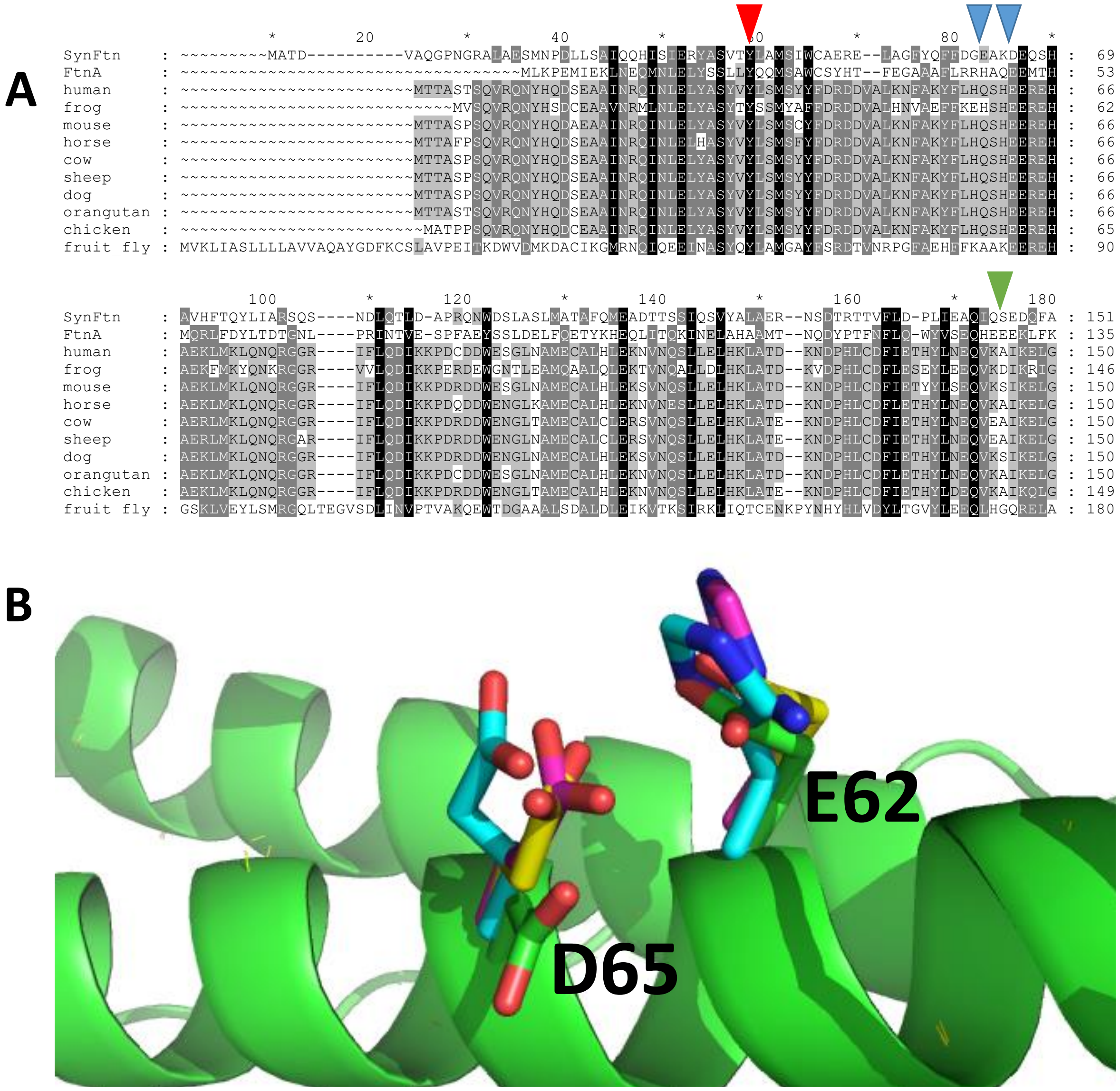

Figure S1. Comparison of selected ferritin sequences and structures. (A) The peptide sequence of *Syn*Ftn aligned with those of the H-chain ferritins identified in *Escherichia coli*, *Homo sapiens*, *Rana catesbeiana* (middle subunit), *Mus musculus*, *Equus caballus*, *Bos taurus*, *Ovis aries*, *Canis familiaris*, *Pongo abelii*, *Gallus domesticus* and *Drosophila melanogaster*. The conserved Tyr at position 40 in the *Syn*Ftn sequence is indicated with a red triangle, the residues equivalent to the carboxylates Glu_{62} and Asp_{65} with blue triangles and those equivalent to Ser_{146} of *Syn*Ftn with a green triangle. (B) Alignments of the structures from the coordinate files for *Syn*Ftn (green), *Escherichia coli* FtnA (cyan), *Rana catesbeiana* middle ferritin (magenta) and *Human H ferritin* (yellow) showing variation in the residue equivalent to Glu_{62}, and that Asp_{65} adopts a conformation distinct from the Glu present at the equivalent position in the other proteins.

Part II: Molecular dynamics simulation and model set-up.

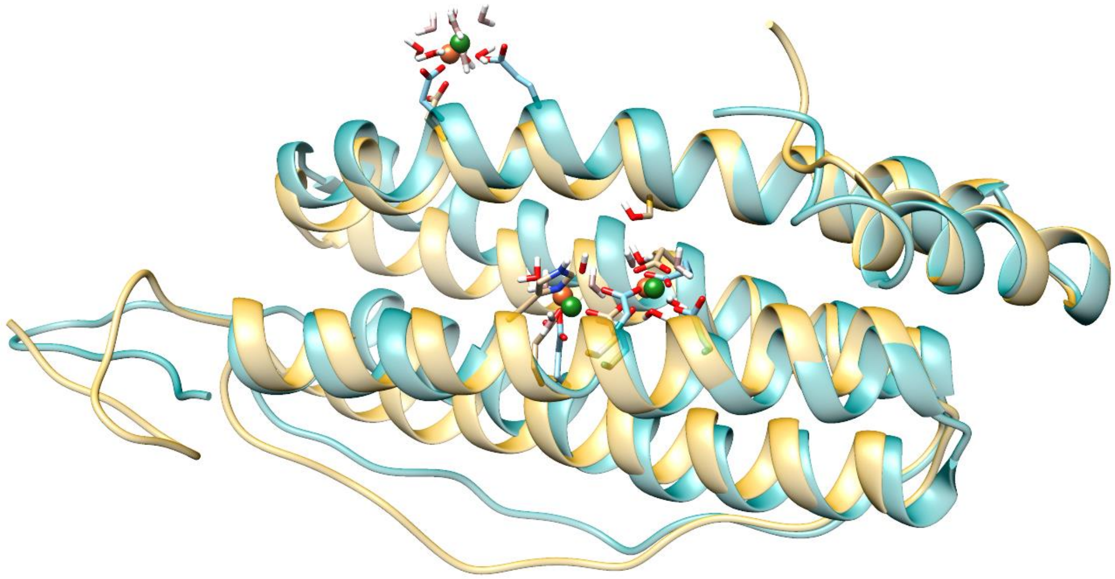

Figure S2: Overlay of the starting and 100 ns snapshot of the MD simulation. Our model was set up from the original pdb file with code 6GKA, which has a single protein chain. Hydrogen atoms were added to the structure by AMBER16. The protein atoms were described with the ff14SB4 force field parameters and the enzyme structure was solvated in octagonal box of TIP3P water molecules. The diiron core with its first-coordination sphere was optimized in Gaussian-09 at the B3LYP/6-31G* level of theory and the RESP charges transferred to the forcefield using the MCPB routine in Amber. A molecular dynamics (MD) simulation was carried out using the Particle Mesh Ewald Molecular Dynamics (PMEMD) module as implemented in the AMBER16 software package. The system was minimized using 2000 steps of steepest decent minimization and subsequently heated for 10 ns from a temperature of 0 K to 310.00 K under NVT ensemble conditions with the Langevin thermostate and thereafter equilibrated for 10 ns under NPT conditions using the Berendsen barostate without constraints to the energy and the structure. A 500 ns MD simulation under NPT conditions was carried out in 2 fs time-steps, using the SHAKE protocol on hydrogen atoms and a 10 Å non-bonded cut-off with periodic boundary conditions.

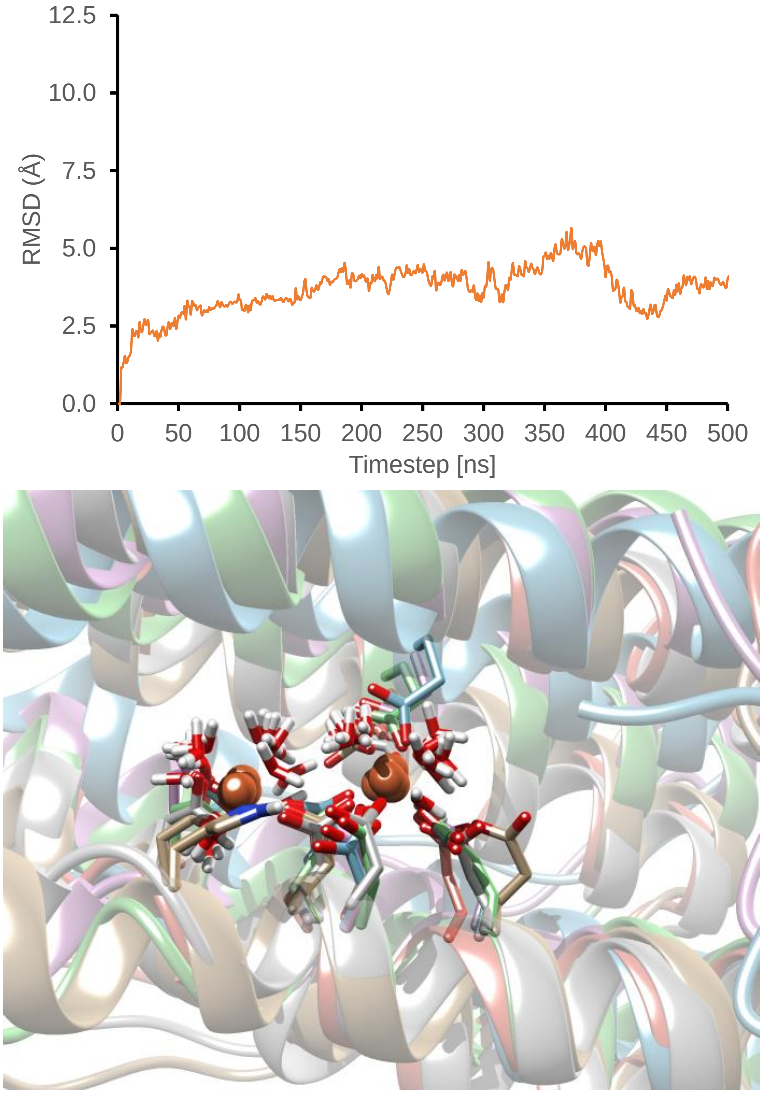

Figure S3: Top: Root-mean-square-deviation of the 500 ns MD simulation in Amber on the ferritin structure. Bottom: Overlays of the structures obtained after 0, 100, 200, 300, 400 and 500 ns.

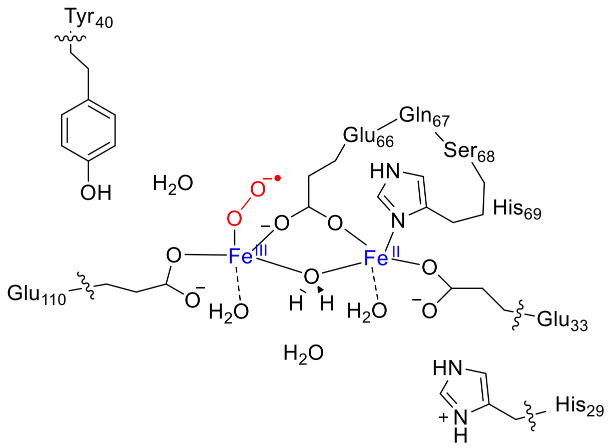

Scheme S1: Small model A investigated in this work. Model **A** included the two iron atoms together with the peptide chain Glu_{66}-Gln_{67}-Ser_{68}-His_{69}, whereby the Gln and Ser residues were abbreviated to Gly. In addition, the iron ligands Glu_{33} and Glu_{110} were included as propionate groups. Two nearby proton sources were identified as Tyr_{40} (mimicked as trans-ethylphenol) and His_{29} (taken as ethylimidazole). A superoxo group was added to one of the iron atoms in an end-on conformation and five water molecules were included. We tested model **A** in two metal oxidation states with overall charge 0 ($Fe^{I}Fe^{I}$, model **A1**) or overall charge +2 ($Fe^{II}Fe^{II}$, model **A2**).

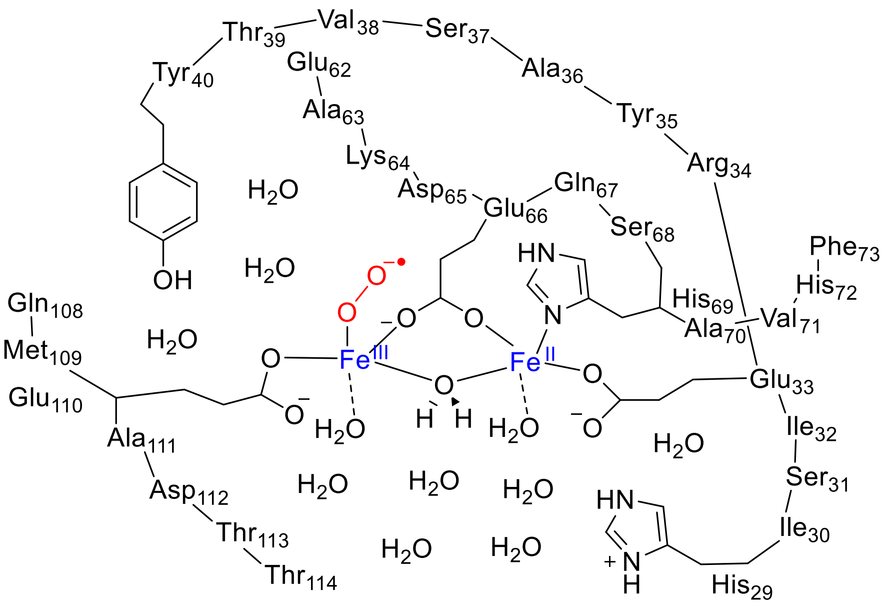

Scheme S2: Large model B investigated in this work. Model **B** contains several protein chains: Firstly, the chain His$_{29}$-Ile$_{30}$-Ser$_{31}$-Ile$_{32}$-Glu$_{33}$-Arg$_{34}$-Tyr$_{35}$-Ala$_{36}$-Ser$_{37}$-Val$_{38}$-Thr$_{39}$-Tyr$_{40}$ was taken with residues 30, 31, 32, 34, 35, 38 and 39 abbreviated to Gly. Secondly, the chain Glu$_{62}$-Ala$_{63}$-Lys$_{64}$-Asp$_{65}$-Glu$_{66}$-Gln$_{67}$-Ser$_{68}$-His$_{69}$-Ala$_{70}$-Val$_{71}$-His$_{72}$-Phe$_{73}$ was included with the residues 63, 64, 67, 68, 70 and 71 abbreviated to Gly. Thirdly, the chain Gln$_{108}$-Met$_{109}$-Glu$_{110}$-Ala$_{111}$-Asp$_{112}$-Thr$_{113}$-Thr$_{114}$ was taken with residues 108, 111, 112 and 114 abbreviated to Gly. Finally, the chain Gln$_{143}$-Ile$_{144}$-Glu$_{145}$-Ser$_{146}$ was included with residues 144 and 145 abbreviated to Gly and Phe$_{150}$ shortened to a toluene molecule. A superoxo group was added to one of the iron atoms in an end-on conformation and twelve water molecules were included. This system was calculated with overall charge 0 and overall odd spin multiplicity (S = 1, 3, 5, 7, 9, and 11) and had 340 atoms. Model **BP** is model **B** with an additional proton added to Asp$_{65}$ and has overall charge +1 and overall odd spin multiplicity (S = 1, 7, 9, and 11).

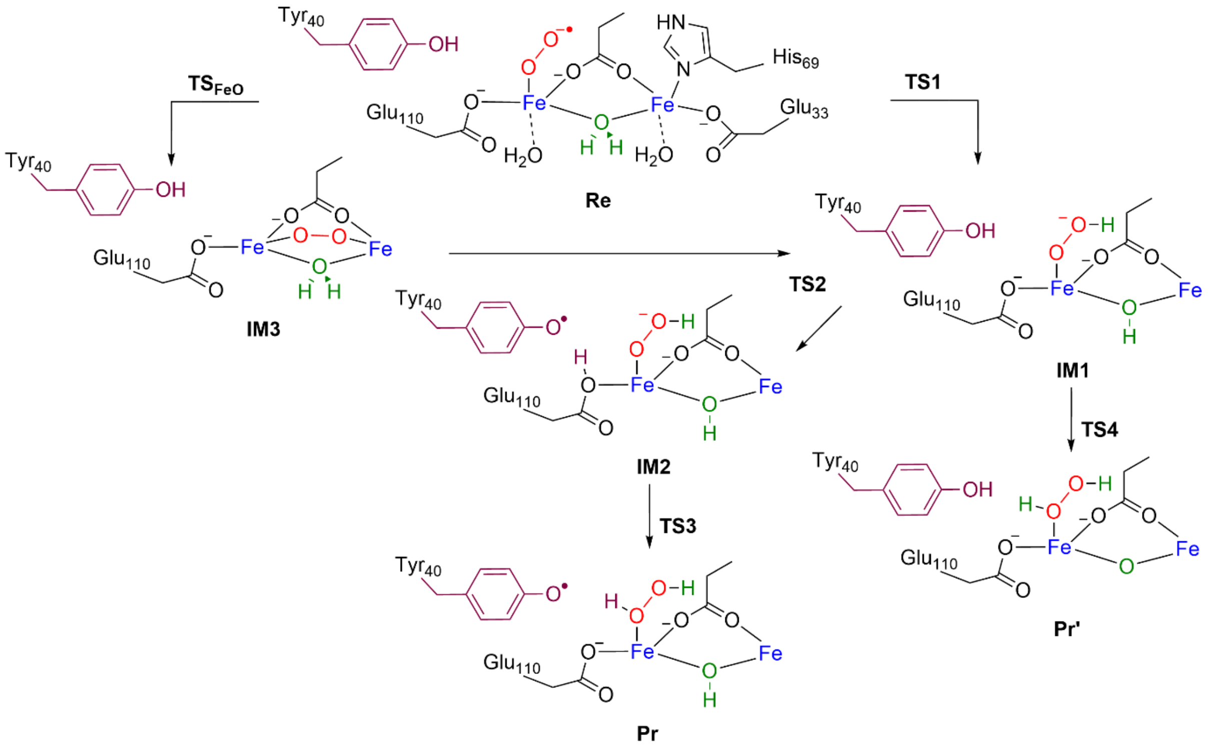

Scheme S3: Reaction mechanism investigated for model B with definition of the labels for H_2O_2 synthesis from dioxygen activation by ferritin. Step 1: proton transfer from μ-H_2O group to superoxo. Step 2: hydrogen atom transfer from Tyr_{40} to Glu_{110}. Step 3: proton transfer from Glu_{110} to hydroperoxo.

Part III: Data on small model A1, i.e. Model A with overall charge = 0.

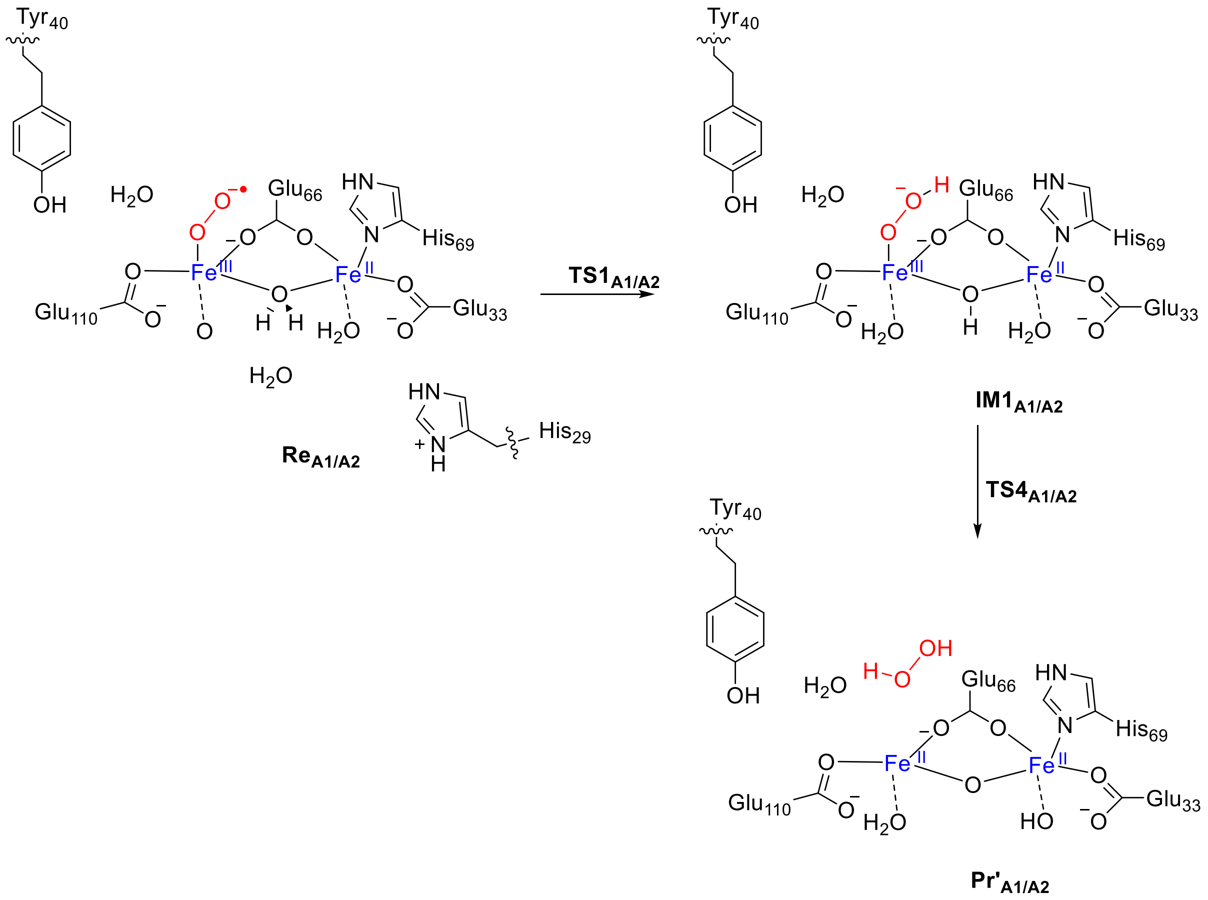

Scheme S4: Reaction mechanism investigated for model A1/A2 with definition of the labels for H_2O_2 synthesis from dioxygen activation by ferritin. Step 1: proton transfer from μ-H_2O group to superoxo. Step 2: proton transfer from μ-OH to hydroperoxo.

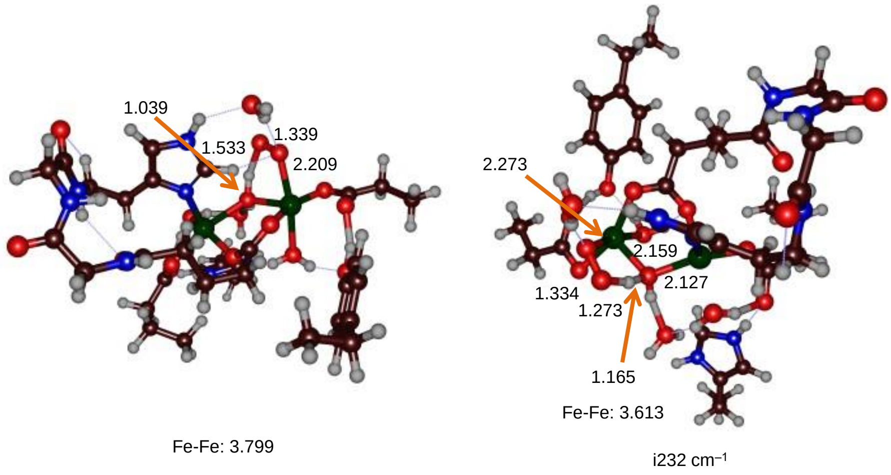

Re_{A1} **$TS1_{A1}$**

Figure S4: UB3LYP/BS1 optimized geometries of the reactant and first proton-transfer transition state, $^{11}Re_{A1}$ and $^{11}TS1_{A1}$, as obtained in Gaussian-09 using Model A1. Bond lengths are in angstroms and the imaginary frequency in cm^{-1}.

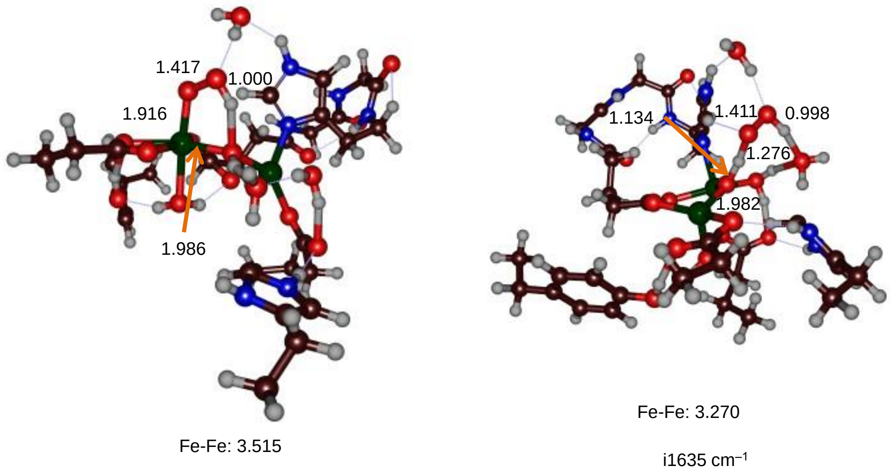

$IM1_{A1}$ **$TS4_{A1}$**

Figure S5: UB3LYP/BS1 optimized geometries of the intermediate and second proton-transfer transition state, $^{11}IM1_{A1}$ and $^{11}TS4_{A1}$, as obtained in Gaussian-09 using Model A1. Bond lengths are in angstroms and the imaginary frequency in cm^{-1}.

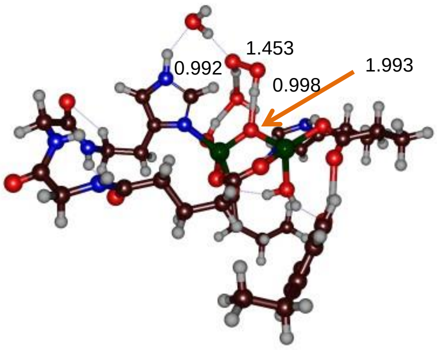

Fe-Fe: 3.192

Pr’$_{A1}$

Figure S6: UB3LYP/BS1 optimized geometry of the H_2O_2-bound product complex ^{11}Pr’$_{A1}$ as obtained in Gaussian-09 on Model A1. Bond lengths are in angstroms.

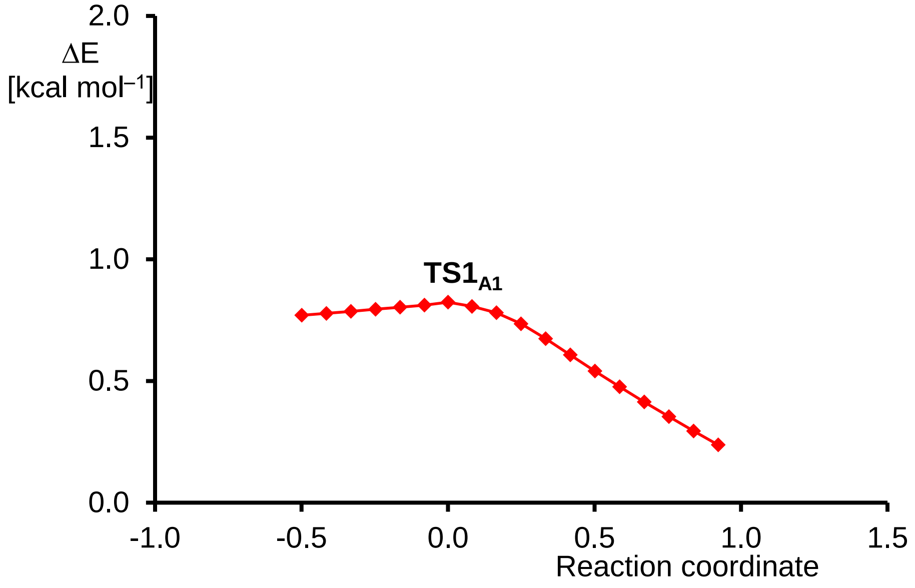

Figure S7: UB3LYP/BS1 calculated intrinsic reaction coordinate scan from ^{11}TS1$_{A1}$. Energies are relative to the reactant complex ^{11}Re$_{A1}$.

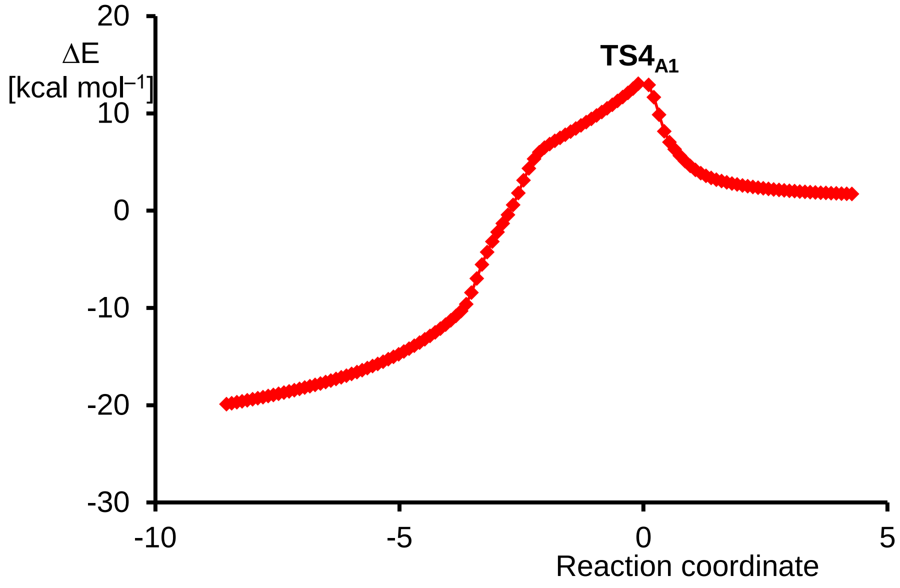

Figure S8: UB3LYP/BS1 calculated intrinsic reaction coordinate scan from ^{11}TS4$_{A1}$. Energies are relative to the reactant complex Re$_{A1}$.

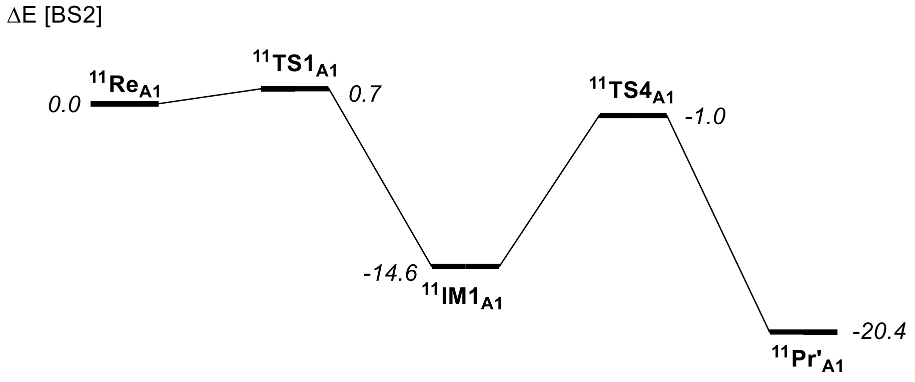

Figure S9: UB3LYP/BS2//UB3LYP/BS1 calculated potential energy profile for the conversion of O_2 to H_2O_2 on the diiron model A1. Energies are in kcal mol^{-1} relative to the reactant complex Re$_{A1}$.

Table S1: Absolute (free) energies (in au) of optimized geometries for H_2O_2 generation on a diiron center of ferritin as calculated with DFT using model A1.

	E [BS1, au]	ZPE [au]	G [au]	E [BS2, au]
**Re**$_{A1}$	-3241.486662	0.986085	-3240.627654	-3242.507461
**TS1**$_{A1}$	-3241.485349	0.982708	-3240.631650	-3242.506340
**IM1**$_{A1}$	-3241.493835	0.987494	-3240.631340	-3242.530678
**TS4**$_{A1}$	-3241.470311	0.985925	-3240.612921	-3242.508971
**Pr'**$_{A1}$	-3241.496681	0.987861	-3240.634659	-3242.539982

Table S2: Relative (free) energies (in kcal mol^{-1}) of optimized geometries for H_2O_2 generation on a diiron center of ferritin as calculated with DFT using model A1.

	ΔE	ΔE+ZPE	ΔG	ΔE	ΔE+ZPE	ΔG
	BS1	BS1	BS1	BS2	BS2	BS2
**Re**$_{A1}$	0.00	0.00	0.00	0.00	0.00	0.00
**TS1**$_{A1}$	0.82	-1.30	-2.51	0.70	-1.42	-2.63
**IM1**$_{A1}$	-4.50	-3.62	-2.31	-14.57	-13.68	-12.38
**TS4**$_{A1}$	10.26	10.16	9.25	-0.95	-1.05	-1.96
**Pr'**$_{A1}$	-6.29	-5.17	-4.40	-20.41	-19.29	-18.52

Part IV: Data on small model A2, i.e. Model A with overall charge = +2.

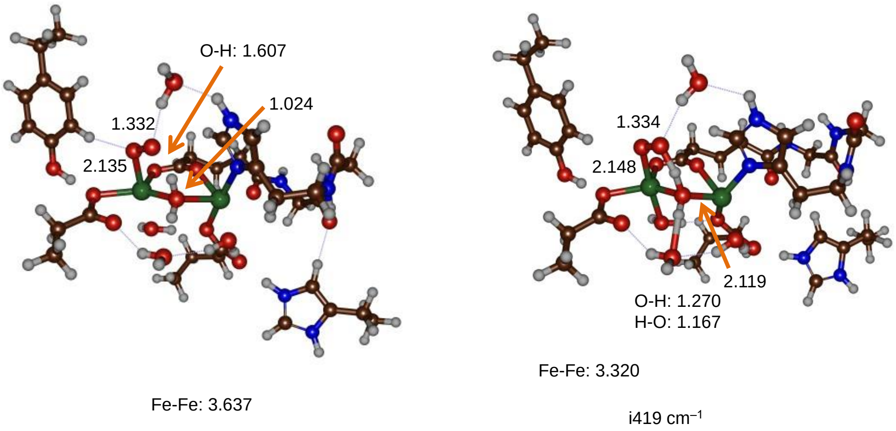

Re$_{A2}$ **TS1$_{A2}$**

Figure S10: UB3LYP/BS1 optimized geometries of the reactant and first proton-transfer transition state, ^{11}Re$_{A2}$ and ^{11}TS1$_{A2}$, as obtained in Gaussian-09 using Model A2. Bond lengths are in angstroms and the imaginary frequency in cm^{-1}.

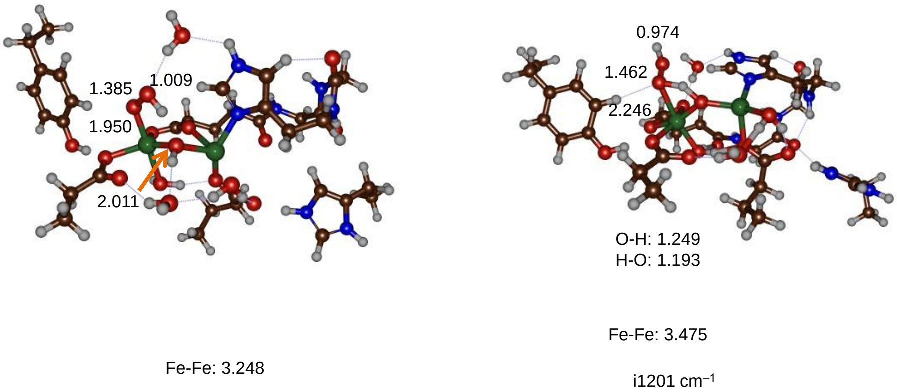

IM1$_{A2}$ **TS4$_{A2}$**

Figure S11: UB3LYP/BS1 optimized geometries of the intermediate and second proton-transfer transition state, ^{11}IM1$_{A2}$ and ^{11}TS4$_{A2}$, as obtained in Gaussian-09 using Model A2. Bond lengths are in angstroms and the imaginary frequency in cm^{-1}.

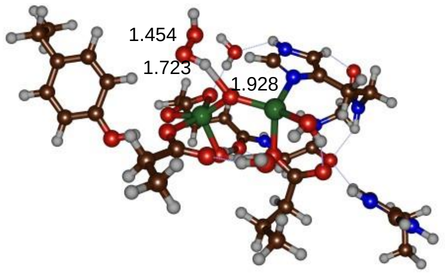

Fe-Fe: 3.355

Pr’$_{A2}$

Figure S12: UB3LYP/BS1 optimized geometry of the H_2O_2-bound product complex ^{11}Pr’$_{A2}$ as obtained in Gaussian-09 on Model A2. Bond lengths are in angstroms.

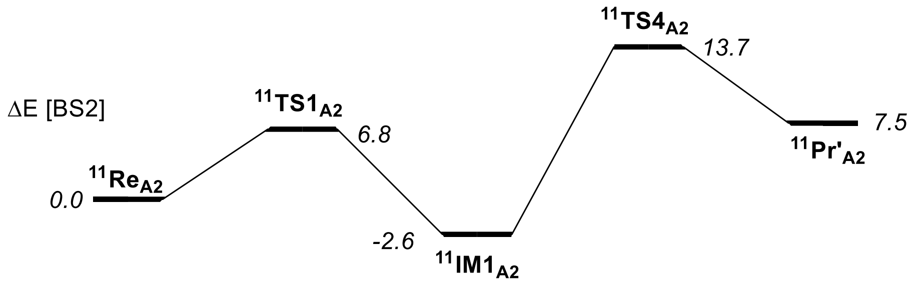

Figure S13: UB3LYP/BS2//UB3LYP/BS1 calculated potential energy profile for the conversion of O_2 to H_2O_2 on the diiron model A2. Energies are in kcal mol^{-1} relative to the reactant complex Re$_{A2}$.

Table S3: Absolute (free) energies (in au) of optimized geometries for H_2O_2 generation on a diiron center of ferritin as calculated with DFT using model A2.

	E [BS1, au]	ZPE [au]	G [au]	E [BS2, au]
**Re**$_{A2}$	-3241.094113	0.990204	-3240.230235	-3242.206070
**TS1**$_{A2}$	-3241.085851	0.985203	-3240.227191	-3242.195230
**IM1**$_{A2}$	-3241.090147	0.989866	-3240.226461	-3242.210250
**TS4**$_{A2}$	-3241.051492	0.987372	-3240.188705	-3242.184300
**Pr'**$_{A2}$	-3241.062173	0.991846	-3240.197713	-3242.194170

Table S4: Relative (free) energies (in kcal mol^{-1}) of optimized geometries for H_2O_2 generation on a diiron center of ferritin as calculated with DFT using model A2.

	ΔE	ΔE+ZPE	ΔG	ΔE	ΔE+ZPE	ΔG
	BS1	BS1	BS1	BS2	BS2	BS2
**Re**$_{A2}$	0.00	0.00	0.00	0.00	0.00	0.00
**TS1**$_{A2}$	5.18	2.05	1.91	6.80	3.66	3.53
**IM1**$_{A2}$	2.49	2.28	2.37	-2.62	-2.84	-2.74
**TS4**$_{A2}$	26.74	24.97	26.06	13.66	11.88	12.98
**Pr'**$_{A2}$	20.04	21.07	20.41	7.47	8.50	7.83

Table S5: Group spin densities of optimized geometries for H_2O_2 generation on a diiron center of ferritin as calculated with DFT using model A2.

Spin	Fe1	Fe2	OOH	Bridge	HisH	Tyr	Prot	Total
**Re**$_{A2}$	3.75	3.79	1.06	0.05	0.00	1.00	0.35	10.00
**TS1**$_{A2}$	3.76	3.77	1.03	0.08	0.00	1.00	0.36	10.00
**IM1**$_{A2}$	3.76	3.93	0.66	0.19	0.00	1.00	0.45	10.00
**TS4**$_{A2}$	4.08	3.76	0.12	0.43	0.00	1.00	0.60	10.00
**Pr'**$_{A2}$	4.03	3.76	0.02	0.64	0.00	1.00	0.56	10.00

Table S6: Group charges of optimized geometries for H_2O_2 generation on a diiron center of ferritin as calculated with DFT using model A2.

Charge	Fe1	Fe2	OOH	Bridge	HisH	Tyr	Prot	Total
**Re**$_{A2}$	0.65	0.66	-0.56	0.11	0.84	0.83	-0.52	2.00
**TS1**$_{A2}$	0.62	0.73	-0.54	0.09	0.84	0.83	-0.57	2.00
**IM1**$_{A2}$	0.72	0.76	-0.21	-0.43	0.84	0.85	-0.53	2.00
**TS4**$_{A2}$	0.89	0.85	-0.45	-0.38	0.84	0.87	-0.62	2.00
**Pr'**$_{A2}$	0.90	0.81	0.07	-0.83	0.84	0.87	-0.66	2.00

Part V: Data on large model B.

Summary of the results on model B calculations:

Starting with a geometry optimization of the superoxo-iron(III)-μ-water-iron(II) reactant complex, energies were calculated for the singlet, triplet, quintet, septet, nonet and undecaplet spin states using the 340 atoms model shown in Figure 2 of the main paper. For all spin states, optimization resulted in a di-iron(II)-superoxo complex, with an unpaired electron on the superoxo group, and an unpaired electron shared between the carboxylate groups of Glu_{62} and Asp_{65} in π^*_{Asp}. Although electronic configurations with a radical on an Asp carboxylate group are highly unusual in bioinorganic systems, we nevertheless determined the mechanism of reaction associated with the spin states of the model.

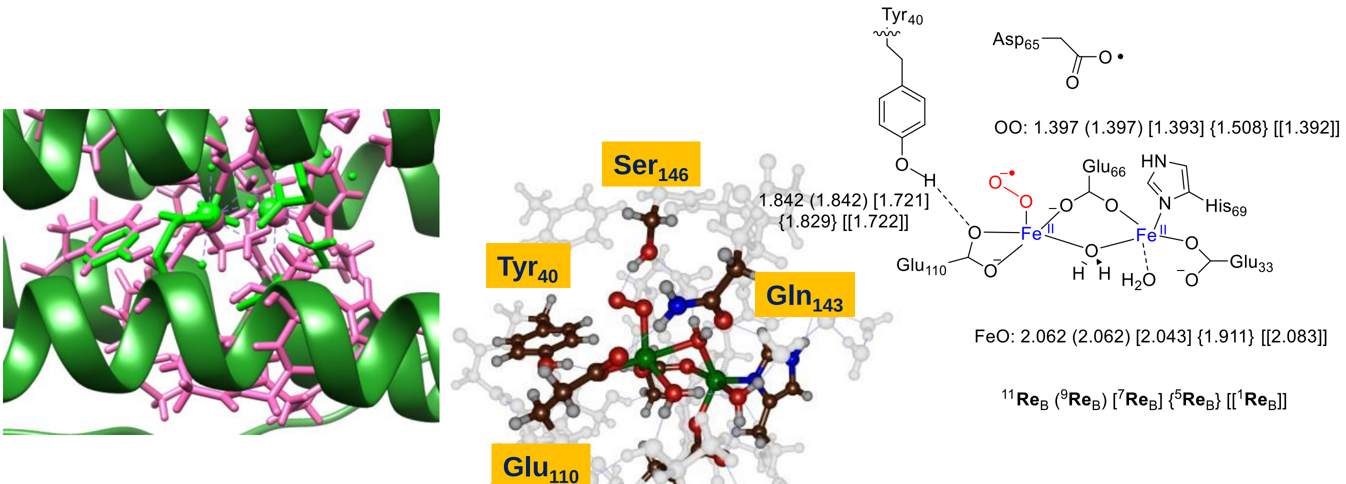

Overlay 6GKA and ^{11}Re$_B$

ΔE+ZPE = 8.8 (8.8) [0.0] {36.4} ((22.8)) [[6.6]] kcal mol^{-1}

^{11}**Re**$_B$ (^{9}**Re**$_B$) [^{7}**Re**$_B$] {^{5}**Re**$_B$} ((^{3}**Re**$_B$)) [[^{1}**Re**$_B$]]

Figure S14. UB3LYP/BS1 optimized geometries of the diiron-superoxo complex as calculated in the spin multiplicity M = 1, 3, 5, 7, 9 and 11. Bond distances are in angstroms and relative energies are UB3LYP/BS2//UB3LYP/BS1+ZPE+E_{solv} in kcal mol^{-1}. The left-hand-side shows an overlay of the ^{11}**Re**$_B$ structure (in pink) with the crystal structure coordinates of the 6GKA pdb file (chains and atoms in green).

The septet spin state (^{7}**Re**$_B$) is the ground state, while the open-shell singlet spin state (^{1}**Re**$_B$) is higher in energy by ΔE+ZPE+E_{solv} = 6.6 kcal mol^{-1} (ZPE = zero-point energy; E_{solv} = solvent correction energy), Figure S14. The undecaplet and nonet spin states are higher in energy than the septet reactant by 8.8 and 8.8 kcal mol^{-1}. Much higher in energy and possibly inaccessible in this conformation are the quintet and triplet spin reactants, namely by 36.4 and 22.8 kcal mol^{-1}, respectively. These spin-state energies implicate a dominant septet spin state as the relevant state for the reaction mechanism. Key features of the optimized geometries of the diiron-superoxo complexes (**Re**$_B$) are shown in Figure S14. In all structures, the superoxo is bound in an end-on conformation with the terminal oxygen atom in hydrogen bonding interactions with the alcohol group of Ser_{146}, while the distal oxygen atom interacts with the side chain of Gln_{143}. The latter group also interacts with the bridging water molecule and the carboxylate of Glu_{110}. The Tyr_{40} residue is protonated at the phenol group and is located at a distance of 3.355 Å to the terminal oxygen atom of the superoxo group and at 1.842 Å to the carboxylate of Glu_{110} in ^{11}**Re**$_B$. The O–O bond is short and ranges from 1.393 Å for ^{7}**Re**$_B$ to 1.397 Å for ^{11}**Re**$_B$, while the Fe–O distance is close for the four spin states in Figure S14 as well.

The molecular orbitals of the reactant complexes are shown in Figure S15. The relevant molecular orbitals mainly originate from the metal 3d sets of orbitals on Fe1 and Fe2 and their interactions with first-coordination sphere ligands. Orbital occupation is $\pi^*_{xy,Fe1}{}^2$ $\pi^*_{xz,Fe1}{}^1$ $\pi^*_{yz,Fe1}{}^1$ $\sigma^*_{z2,Fe1}{}^1$ $\sigma^*_{x2-y2,Fe1}{}^1$ $\pi^*_{xy,Fe2}{}^2$ $\pi^*_{xz,Fe2}{}^1$ $\pi^*_{yz,Fe2}{}^1$ $\sigma^*_{z2,Fe2}{}^1$ $\sigma^*_{x2-y2,Fe2}{}^1$ $\pi^*_{OO}{}^1$ $\pi^*_{Asp}{}^1$ in the undecaplet, nonet, septet and singlet spin states. All unpaired electrons are ferromagnetically coupled in the undecaplet spin state. In the nonet spin state, the radical on Asp is

antiferromagnetically coupled to the other electrons, while in the septet state the π^*_{OO} radical is also antiferromagnetically coupled. The singlet spin state has the electrons on Fe1 with up-spin and those on Fe2 with down-spin, while the π^*_{OO} electron is up and the π^*_{Asp} is down-spin.

Subsequently, we tested several reaction mechanisms for oxygen activation starting from the Fe(II)-Fe(II)-superoxo complexes ^{11,9,7,5,3,1}**Re**$_B$ as shown in Scheme S3 above. Firstly, we tested sequential proton transfer from the μ-H_2O bridged group and Tyr_{40}. This pathway starts with proton transfer from the bridged water molecule to the terminal oxygen atom of the Fe(II)-superoxo group, and electron transfer from the Fe(II) to the superoxo group via a transition state **TS1** to form intermediate **IM1**, which features a Fe(III)-Fe(II)-hydroperoxo species. Thereafter, a proton-coupled-electron-transfer from Tyr_{40} to Glu_{110} via transition state **TS2** gives intermediate **IM2**, in which the electron has been transferred to the Fe(III) ion, resulting in a di-Fe(II)-hydroperoxo species. Finally, shuttling of the proton on Glu_{110} to the distal oxygen atom of the Fe(II)-hydroperoxo group, via another transition state **TS3**, gives the μ-hydroxo bridged diiron(II) complex with H_2O_2 bound (**Pr**). In addition, a reaction channel was explored via a μ-peroxo bridged diiron(III) complex (**IM3**) from the reactant complexes. A proton transfer from the bridging water molecule in **IM3** to the peroxo group, along with oxidation of the Asp side chain to form a radical, leading to **IM1** again. Finally, we tested double proton donation by the bridging water molecule in **Re**$_B$ to dioxygen, whereby first **IM1** is formed and from **IM1** a proton is transferred to the distal oxygen atom of the iron(III)-hydroperoxo group to form H_2O_2 and the Fe(II) is oxidized with reduction of the Asp radical, via transition state **TS4**, to give μ-oxo-bridged diiron(III) complex with H_2O_2 bound (**Pr'**).

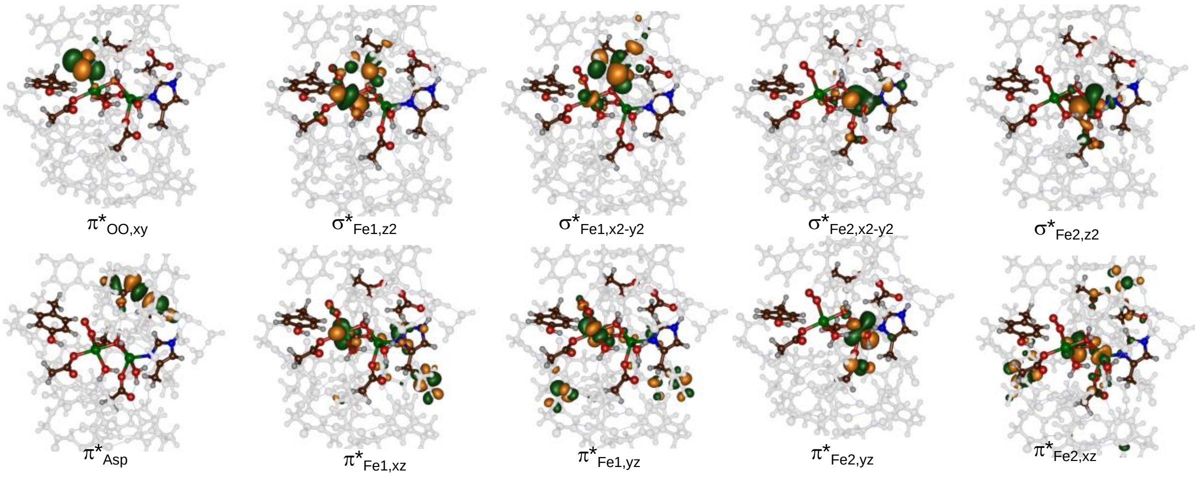

Figure S15: Molecular orbitals of the diiron-superoxo complexes Re$_B$ of ferritin.

For model **B** the lowest energy mechanism was found to proceed via **TS2** and the formation of a μ-hydroxo bridged di-Fe(II) complex. This pathway was explored for all low energy spin states; however, the triplet and quintet spin states were very high in energy, but the competing mechanisms on the singlet, septet, nonet and undecaplet spin states are shown in Figure S16. The reaction happens via multistate reactivity patterns with close lying spin state energies. Although the reactant state is the lowest in the septet spin state, actually the first proton transfer via ^{7}**TS1**$_B$ has a barrier of 14.9 kcal mol^{-1}, while much lower barriers are found for the nonet and undecaplet spin states. Although fully characterized as a transition state structure, their energies were of the same order of magnitude as the reactant complexes and hence these proton transfer steps will happen very quickly on the nonet and undecaplet spin states. The ^{11,9}**TS1**$_B$ barriers relax to stable Fe(II)-Fe(III)-hydroperoxo complexes (**IM1**$_B$) that are ΔE+ZPE = –2.5 (0.9) kcal mol^{-1} in energy with respect to ^{7}**Re**$_B$ on the nonet (undecaplet) spin states. The first proton transfer on the singlet spin state has a barrier of ΔE+ZPE = 14.2 kcal mol^{-1}. The open-shell singlet spin state ^{1}**IM1**$_B$ is 11.1 kcal mol^{-1} above the energy of reactants, but after that point in the mechanism climbs to very high energetic values that make the singlet spin state irrelevant. Nevertheless, the first proton transfer from the bridging water molecule to Fe(II)-superoxo can happen on the singlet, septet, nonet and undecaplet spin states relatively quickly.

Initially, we attempted to abstract a second proton from the bridging water molecule from the **IM1** structures; however, for the large model this pathway had barriers of well over 25 kcal mol^{-1}. Therefore, we explored alternative proton transfer channels and particularly ones where Tyr_{40} would donate a proton. These pathways (via **TS2**) were much lower in energy than the proton transfer from the μ-H_2O bridge. The proton transfer from

Tyr40 is first delivered to the carboxylate of Glu110 via **TS2** and thereafter is relayed to the Fe(II)-hydroperoxo group. The lowest barrier for proton transfer from Tyr40 to Glu110 is via ^{11}**TS2**$_B$ of ΔE+ZPE = 15.2 kcal mol^{-1}. The subsequent barrier for proton transfer from Glu110 to Fe(II)-hydroperoxo is 19.9 kcal mol^{-1} for the septet, nonet and undecaplet spin states. The potential energy landscape, shown in Figure S16, therefore, indicates a rate-determining final proton transfer from Glu110 to the distal oxygen atom of the Fe(II)-hydroperoxo species.

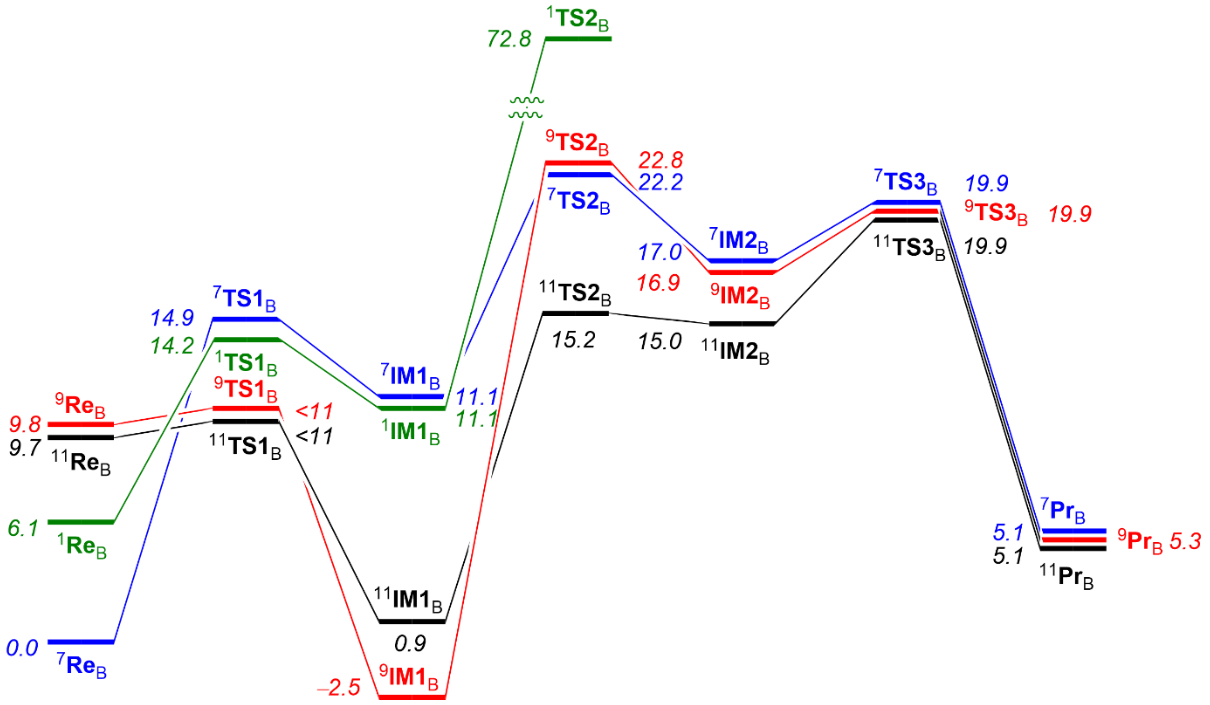

Figure S16: Potential energy profile of dioxygen activation on the diiron center of ferritin using model B. Data represent ΔE_{BS2}+ZPE_{BS1} values (with solvent included) obtained at UB3LYP/BS2//UB3LYP/BS1 level of theory. Values are in kcal mol^{-1}.

To understand the electronic configurations of the various local minima and the electron transfer processes, we highlight the spin and charge distributions in Scheme S5. Thus, as discussed above in Figure S17, the reactant state (^{7}**Re**$_B$) has an electronic configuration of $\pi^*_{xy,Fe1}{}^2$ $\pi^*_{xz,Fe1}{}^1$ $\pi^*_{yz,Fe1}{}^1$ $\sigma^*_{z2,Fe1}{}^1$ $\sigma^*_{x2-y2,Fe1}{}^1$ $\pi^*_{xy,Fe2}{}^2$ $\pi^*_{xz,Fe2}{}^1$ $\pi^*_{yz,Fe2}{}^1$ $\sigma^*_{z2,Fe2}{}^1$ $\sigma^*_{x2-y2,Fe2}{}^1$ $\pi^*_{OO}{}^1$ $\pi^*_{Asp}{}^1$ with an unpaired electron on Asp65 and the superoxo group antiferromagnetically coupled to eight unpaired electrons on the two Fe(II) centers. The first proton transfer from the water bridge to Fe(II)-superoxo occurs with electron transfer from iron to superoxo, leading to a Fe(III)-hydroperoxo species ^{9}**IM1**$_B$ that retains the radical on Asp65. The alternative ^{11}**IM1**$_B$ structure has five unpaired electrons on the Fe(III)-hydroperoxo group and is higher in energy by 3.4 kcal mol^{-1}. In the second step of this mechanism (via **TS2**) a proton-coupled-electron-transfer (PCET) takes place, whereby Tyr40 donates a proton to Glu110, while simultaneously an electron is relayed to the iron to form Fe(II)-hydroperoxo. The **IM2** state has the septet, nonet and undecaplet structures within 2 kcal mol^{-1} and is built up from an Fe(II)-hydroperoxo with a neighboring Fe(II) atom and radicals on Tyr40 and Asp65. In the final stage, the proton is transferred from Glu110 to the hydroperoxo species to form H_2O_2, which retains the electronic configuration of **IM2** into the product complex.

Optimized geometries of key transition states along the H_2O_2 formation mechanism from **Re**$_B$ are shown in Figure S17. The ^{9,11}**TS1**$_B$ structures have a central proton atom for the transfer from the μ-water group to superoxo with distances of 1.209 (1.209) Å toward the superoxo and 1.249 (1.249) Å toward the bridging oxygen atom in ^{11}**TS1**$_B$ (^{9}**TS1**$_B$), respectively. The Fe–O distances in ^{11}**TS1**$_B$ (^{9}**TS1**$_B$) have shortened with respect to those in **Re**$_B$ to values of 1.930 (1.930) Å, probably due to loss of an electron from the antibonding σ^*_{z2} orbital that is located along the Fe–O bond. At the same time the O–O bond is elongated from 1.397 Å in ^{11,9}**Re**$_B$ to 1.519 Å in ^{11,9}**TS1**$_B$. The proton transfer transition state has an imaginary frequency of i779 (i779) cm^{-1} for ^{11}**TS1**$_B$ (^{9}**TS1**$_B$) for the O–H–O stretch vibration. These values are considerably smaller than those typically found for aliphatic hydrogen abstraction transition states, which are often >i1200 cm^{-1}. The hydrogen bonding interactions to the iron(III)-superoxo group probably cause constraints and reduce the imaginary mode.

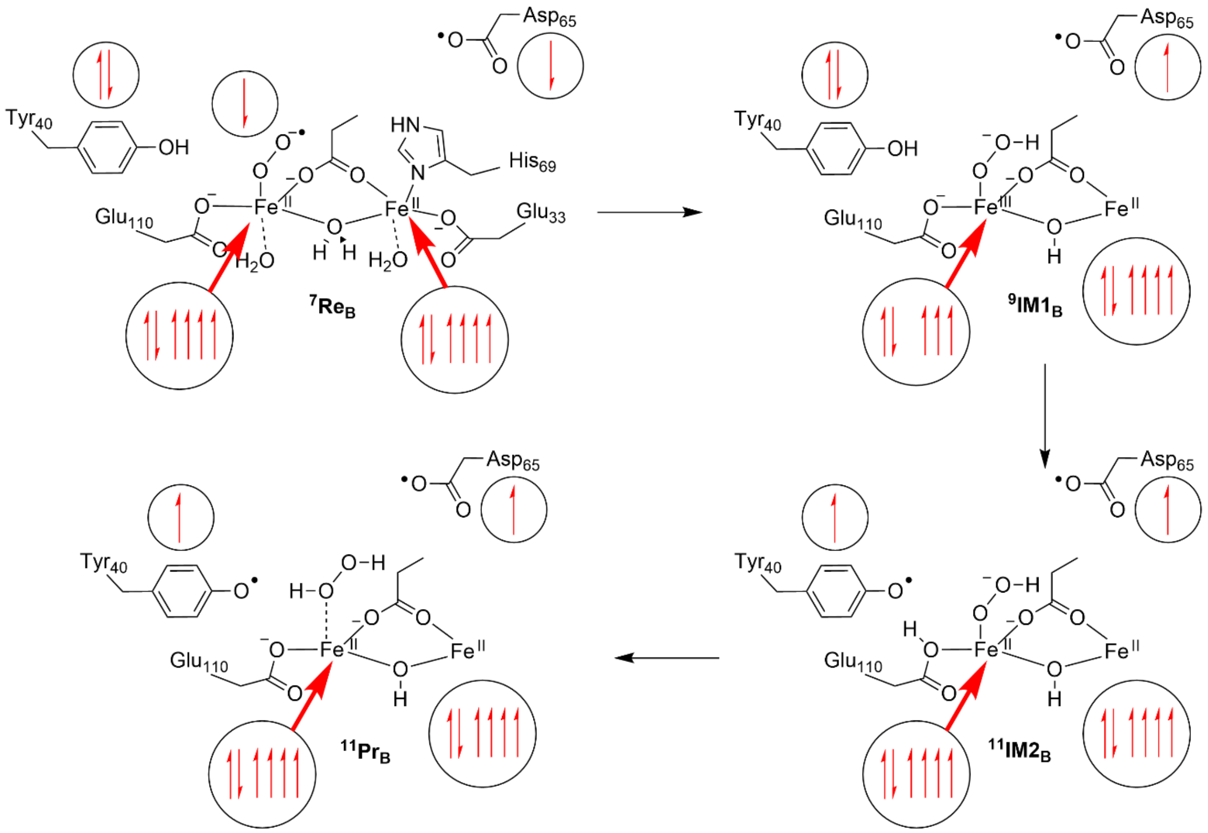

Scheme S5. Electronic configuration and electron transfer pathways for H_2O_2 production from model Re$_B$.

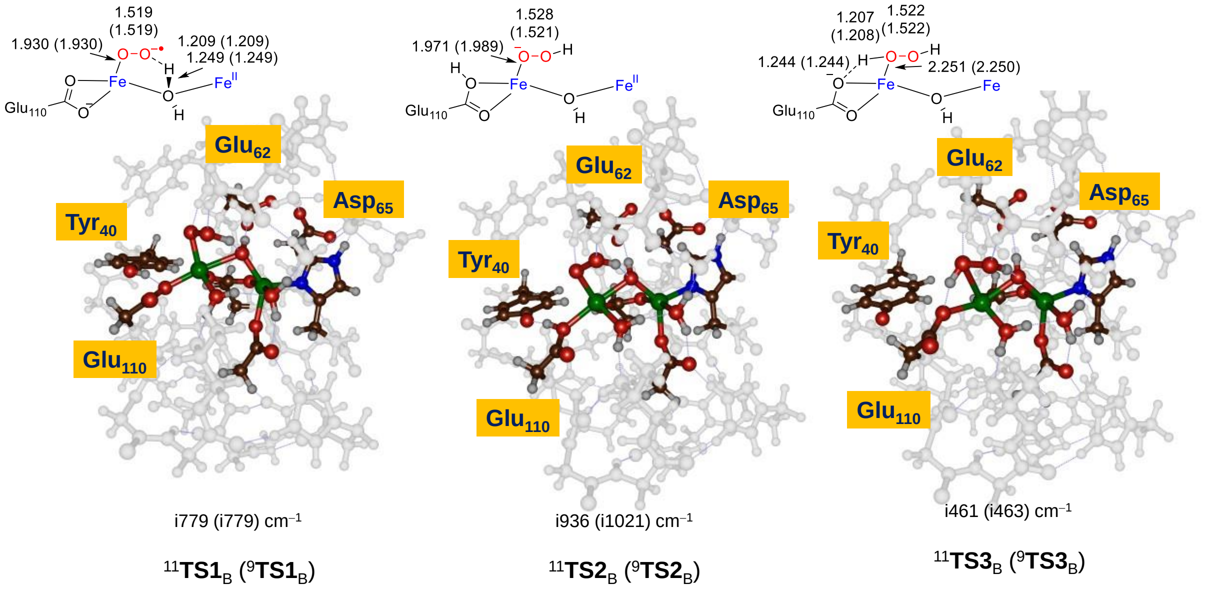

Figure S17. UB3LYP/BS1 optimized geometries of transition states along the H_2O_2 formation mechanism of model Re$_B$. Bond lengths are in angstroms and the imaginary frequency in cm^{-1}.

The lowest PCET transition state occurs via ^{11}**TS2**$_B$ and its structure is shown in the center of Figure S17. Geometrically, the iron(III)-hydroperoxo group is very similar to its precursor structures **IM1** and **TS1**. Consequently, electron transfer from Tyr$_{40}$ to iron results in little change in the geometry of the diiron complex. The proton transfer from Tyr$_{40}$ to Glu$_{110}$ is central with distances of 1.257 Å for the donating group and 1.165 Å for the accepting group for ^{11}**TS2**$_B$. The transition state has an imaginary frequency for proton relay between Tyr$_{40}$ and Glu$_{110}$ of i936 cm^{-1} for ^{11}**TS2**$_B$. The last step creates a H_2O_2-bound di-Fe(II) complex with four unpaired electrons on each of the iron atoms that are ferromagnetically or antiferromagnetically coupled to two unpaired electrons on Tyr$_{40}$ and Asp$_{65}$ in either a septet, nonet or undecaplet spin state. As each of these states has the

same orbital occupancies these states are close in energy for **TS3**$_B$ and **Pr**$_B$. The **TS3** transition states are product-like with short H–OOH distances of 1.207 (1.207) Å and long Glu–H distances of 1.244 (1.244) Å for ^{11}**TS3**$_B$ (^{9}**TS3**$_B$), respectively. These transition states are characterized with small imaginary frequencies of i461 (i463) [i460] cm^{-1}. As a consequence little quantum mechanical tunneling will take place during **TS3** with a small kinetic isotope effect.

To test whether a reaction mechanism via a μ-1,2-peroxo intermediate would be feasible, we ran a geometry optimization of ^{9}**IM3**$_B$ and its structure is shown in Figure S18. Energetically it is 8.5 kcal mol^{-1} higher in energy than ^{7}**Re**$_B$, while a constraint geometry scan from ^{9}**Re**$_B$ for the Fe–O bond formation and ring-closure implicates its formation pathway to be highly costly, i.e. >25 kcal mol^{-1}. As such, the μ-1,2-peroxo diiron complex will have a higher formation energy than the proton transfer energy via **TS1** and will not be able to compete with the mechanism described in Figure S18. Therefore, we do not expect a μ-1,2-peroxo intermediate to be formed in the reaction of dioxygen on the active site of *Syn*Ftn.

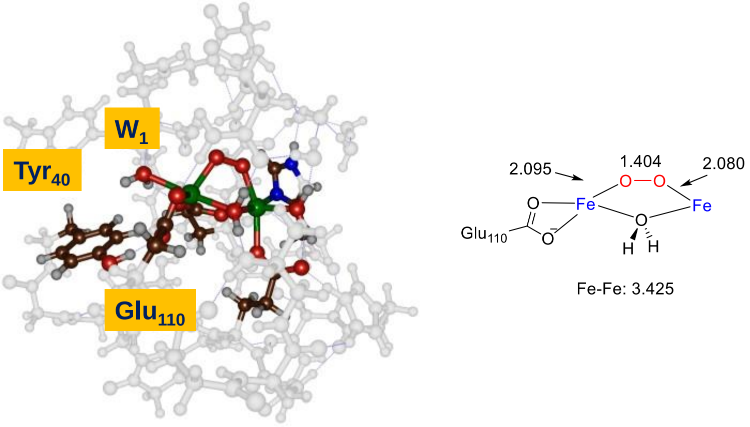

^{9}**IM3**$_B$

Figure S18. UB3LYP/BS1 optimized geometry of ^{9}IM3$_B$ with bond lengths in angstroms. W1 is a ligated water molecule to Fe1.

The optimized geometry of ^{9}**IM3**$_B$ (Figure S18) shows typical features of a μ-1,2-peroxo diiron complex. The O–O bond is 1.404 Å, while the two Fe–O distances are 2.095 and 2.080 Å and the Fe–Fe distance is 3.425 Å. These results match previous calculations on μ-1,2-peroxo diiron complexes well, although the Fe–Fe interaction seems slightly larger than values reported for alternative μ-1,2-peroxo complexes. This large distance may pose additional constraints on the peroxo bridge and make its bond weaker. The optimized geometry of ^{9}**IM3**$_B$ also shows the movement of the ligated water molecule (**W1**) on Fe2. Thus, in the **Re**$_B$ structures **W1** is bound to Fe2 and in hydrogen bonding contact with the carboxylate group of Glu$_{110}$, the alcohol group of Thr$_{113}$ and the water ligand bound to Fe2 in a tight hydrogen bonding network. In the μ-1,2-peroxo structure, by contrast, **W1** has flipped to the other side of the Fe2 atom and forms hydrogen bonding interactions with Glu$_{66}$ and Ser$_{146}$ as well as the phenol group of Tyr$_{40}$. This is possibly the result of the μ-1,2-peroxo formation that has pushed the bridging water molecule down so that it now forms a hydrogen bond with Thr$_{113}$.

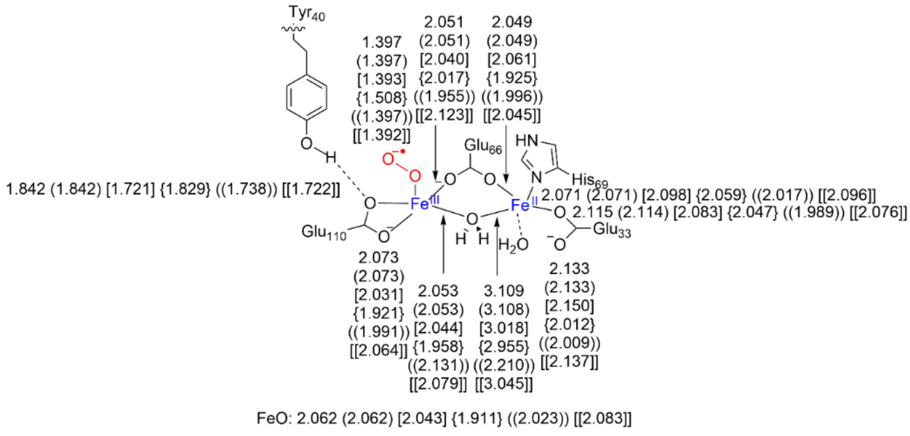

^{11}**Re**$_B$ (^{9}**Re**$_B$) [^{7}**Re**$_B$] {^{5}**Re**$_B$} ((^{3}**Re**$_B$)) [[^{1}**Re**$_B$]]

Figure S19: UB3LYP/BS1 optimized geometries of ^{11,9,7,5,3,1}Re$_B$ as obtained in Gaussian-09 using Model B. Bond lengths are in angstroms.

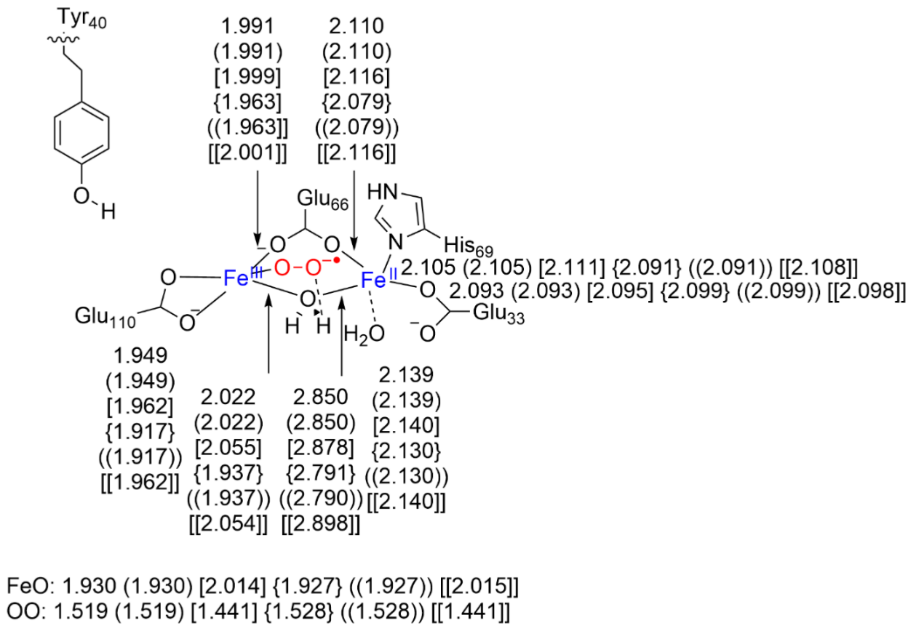

^{11}**TS1**$_B$ (^{9}**TS1**$_B$) [^{7}**TS1**$_B$] {^{5}**TS1**$_B$} ((^{3}**TS1**$_B$)) [[^{1}**TS1**$_B$]]

Figure S20: UB3LYP/BS1 optimized geometries of ^{11,9,7,5,3,1}TS1$_B$ as obtained in Gaussian-09 using Model B. Bond lengths are in angstroms.

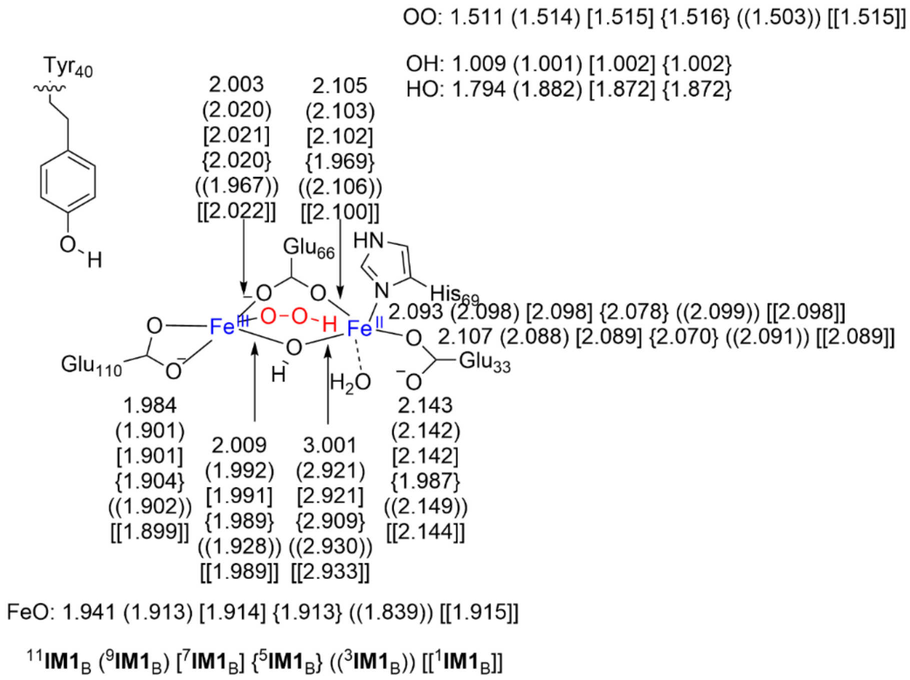

Figure S21: UB3LYP/BS1 optimized geometries of ^{11,9,7,5,3,1}IM1$_B$ as obtained in Gaussian-09 using Model B. Bond lengths are in angstroms.

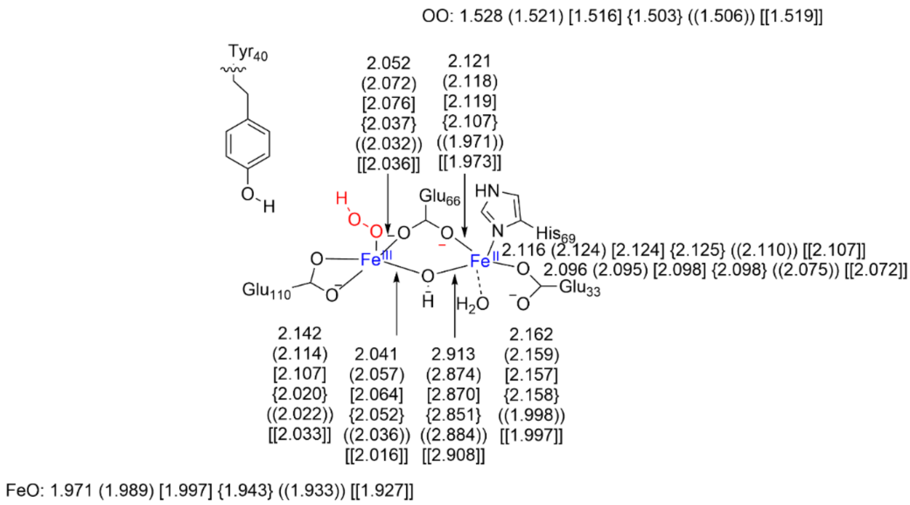

^{11}**TS2**$_B$ (^{9}**TS2**$_B$) [^{7}**TS2**$_B$] {^{5}**TS2**$_B$} ((^{3}**TS2**$_B$)) [[^{1}**TS2**$_B$]]

Figure S22: UB3LYP/BS1 optimized geometries of ^{11,9,7,5,3,1}TS2$_B$ as obtained in Gaussian-09 using Model B. Bond lengths are in angstroms.

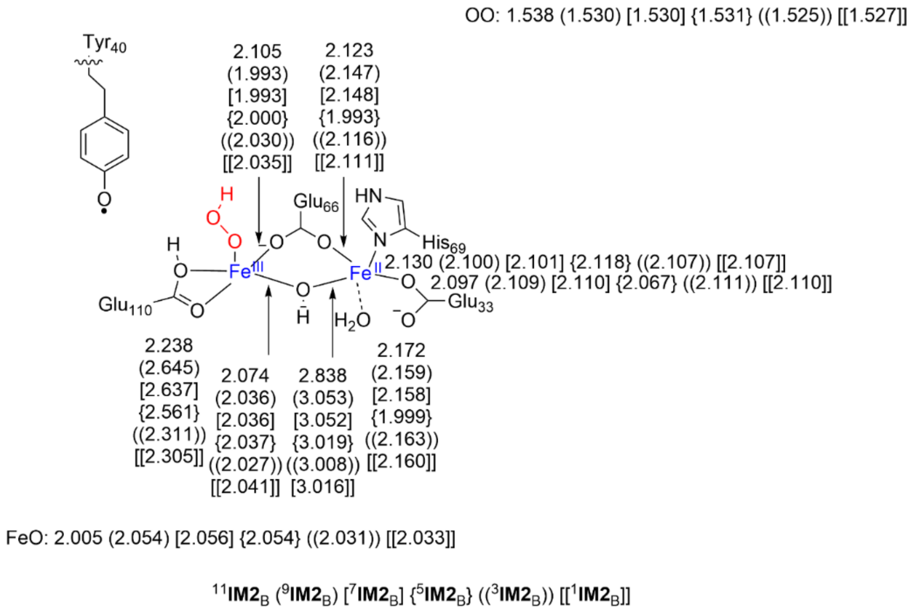

Figure S23: UB3LYP/BS1 optimized geometries of ^{11,9,7,5,3,1}IM2$_B$ as obtained in Gaussian-09 using Model B. Bond lengths are in angstroms.

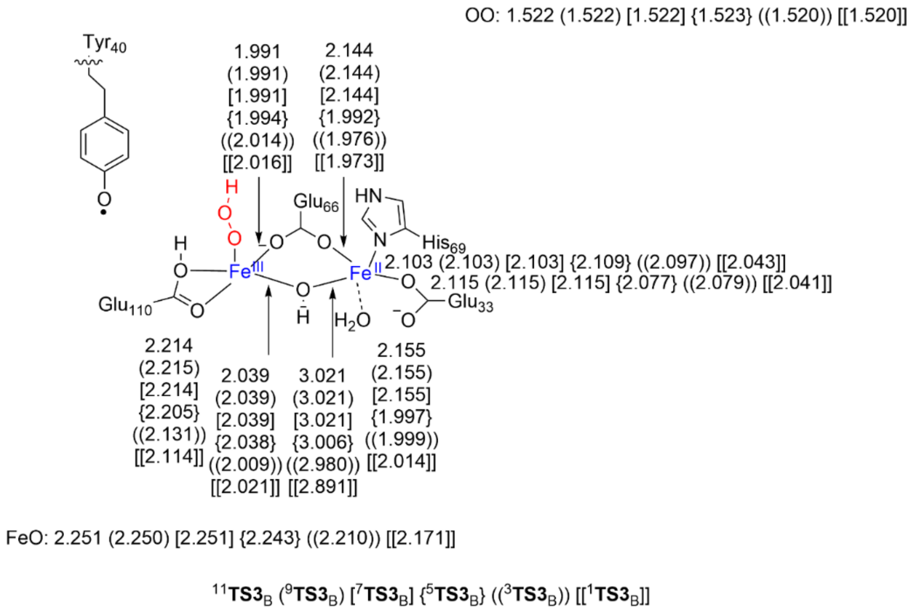

Figure S24: UB3LYP/BS1 optimized geometries of ^{11,9,7,5,3,1}TS3$_B$ as obtained in Gaussian-09 using Model B. Bond lengths are in angstroms.

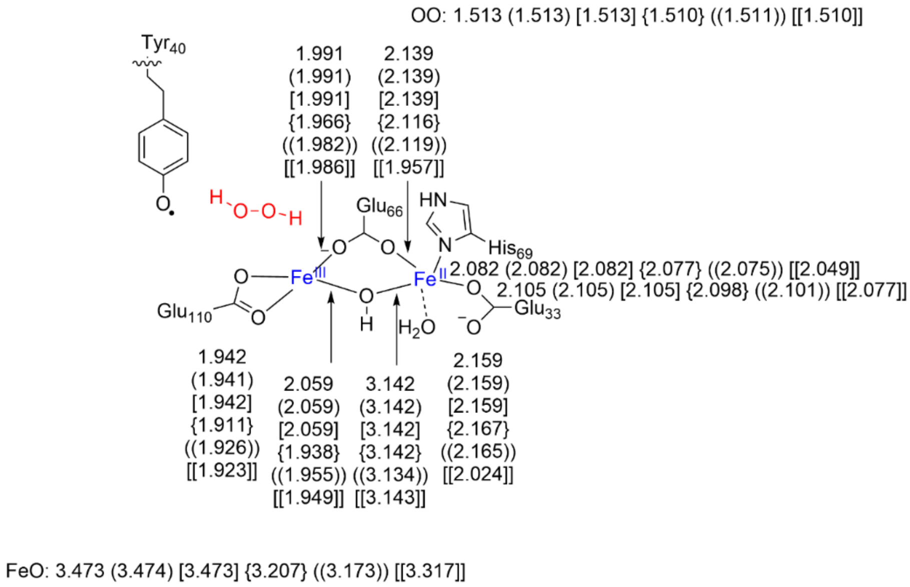

^{11}**Pr**$_B$ (^{9}**Pr**$_B$) [^{7}**Pr**$_B$] {^{5}**Pr**$_B$} ((^{3}**Pr**$_B$)) [[^{1}**Pr**$_B$]]

Figure S25: UB3LYP/BS1 optimized geometries of ^{11,9,7,5,3,1}Pr$_B$ as obtained in Gaussian-09 using Model B. Bond lengths are in angstroms.

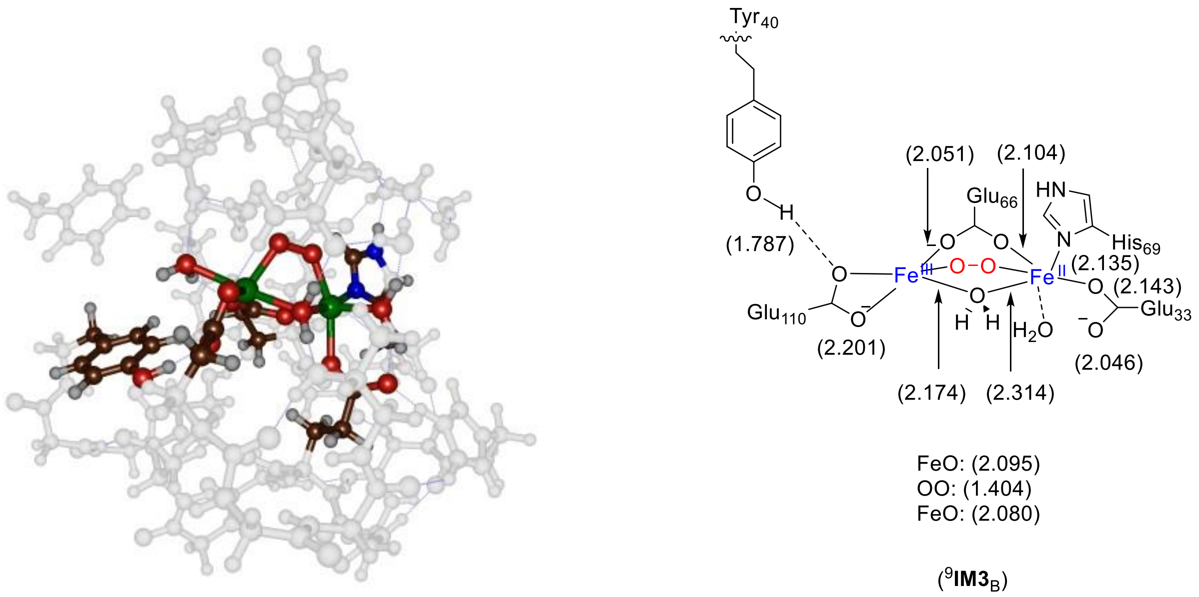

Figure S26: UB3LYP/BS1 optimized geometry of the μ-peroxo-bridged structure ^{9}IM3$_B$ as obtained in Gaussian-09 using Model B. Bond lengths are in angstroms.

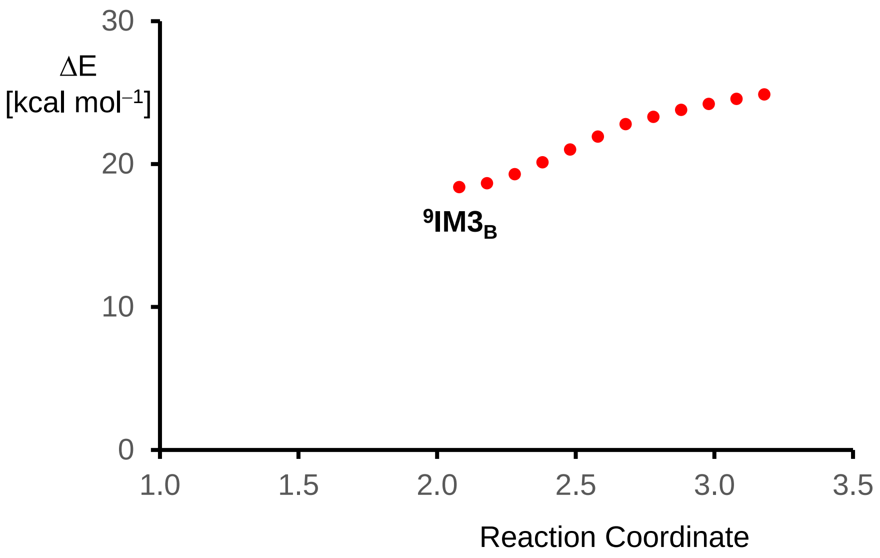

Figure S27: UB3LYP/BS1 calculated constraint geometry scan for the formation of the μ-peroxo-bridged structure ^{9}IM3$_B$ from ^{9}Re$_B$ as obtained in Gaussian-09 using Model B. As can be seen the barrier is >25 kcal mol^{-1} with respect to ^{7}Re$_B$.

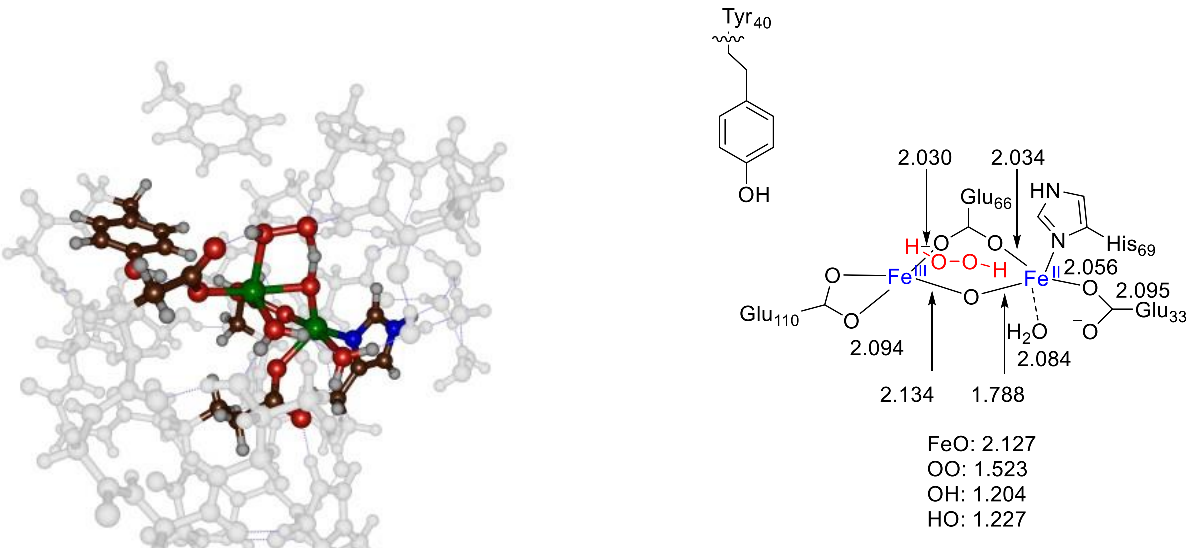

^{11}TS4$_B$

Figure S28: UB3LYP/BS1 optimized geometry of the transition state for the second proton transfer from the μ-H_2O group, i.e. ^{11}TS4$_B$ as obtained in Gaussian-09 using Model B. Bond lengths are in angstroms. The imaginary frequency is 235.8 cm^{-1}.

Table S7: Absolute (free) energies (in au) of optimized geometries for H_2O_2 generation on a diiron center of ferritin as calculated with DFT using model B.

	E(BS1)	ZPE	G	E(BS2)
**^{9}Re$_B$**	-9499.73983	2.79612	-9497.19593	-9505.03063
**^{9}TS1$_B$**	-9499.73533	2.79289	-9497.19165	-9505.03442
**^{9}IM1$_B$**	-9499.74272	2.79697	-9497.19709	-9505.05114
**^{9}TS2$_B$**	-9499.71289	2.79029	-9497.17372	-9505.00412
**^{9}IM2$_B$**	-9499.72569	2.79433	-9497.18552	-9505.01753
**^{9}TS3$_B$**	-9499.72232	2.79121	-9497.18340	-9505.00957
**^{9}Pr$_B$**	-9499.75458	2.79667	-9497.21021	-9505.03839
^{9}**IM3**$_B$	-9499.72445	2.79459	-9497.18337	-9505.031191
**^{7}Re$_B$**	-9499.75377	2.79701	-9497.20845	-9505.04713
**^{7}TS1$_B$**	-9499.71767	2.79357	-9497.17583	-9505.01996
**^{7}IM1$_B$**	-9499.72845	2.79790	-9497.18022	-9505.03027
**^{7}TS2$_B$**	-9499.71314	2.79036	-9497.17313	-9505.00514
**^{7}IM2$_B$**	-9499.72569	2.79432	-9497.18531	-9505.01736
**^{7}TS3$_B$**	-9499.72232	2.79121	-9497.18319	-9505.00962
**^{7}Pr$_B$**	-9499.75458	2.79669	-9497.20994	-9505.03866
**^{11}Re$_B$**	-9499.73982	2.79612	-9497.19620	-9505.03072
**^{11}TS1$_B$**	-9499.73533	2.79289	-9497.19184	-9505.03443
**^{11}IM1$_B$**	-9499.74272	2.79698	-9497.19703	-9505.04568
**^{11}TS2$_B$**	-9499.71997	2.79091	-9497.17997	-9505.01684
**^{11}IM2$_B$**	-9499.72248	2.79416	-9497.18200	-9505.01808
**^{11}TS3$_B$**	-9499.72232	2.79121	-9497.18361	-9505.00963
**^{11}Pr$_B$**	-9499.75458	2.79669	-9497.21037	-9505.03865
^{11}**TS4**$_B$	-9499.71613	2.79155	-9497.17633	-9502.05511
^{11}**Pr'**$_B$	-9499.71621	2.79340	-9497.17570	-9502.05814
**^{1}Re$_B$**	-9499.74321	2.79702	-9497.19490	-9505.03747
**^{1}TS1$_B$**	-9499.71789	2.79363	-9497.17422	-9505.02115
**^{1}IM1$_B$**	-9499.72849	2.79799	-9497.17819	-9505.03040
**^{1}TS2$_B$**	-9499.64439	2.79451	-9497.09660	-9504.92858
**^{1}IM2$_B$**	-9499.68576	2.79439	-9497.14110	-9504.97092
**^{1}TS3$_B$**	-9499.64383	2.79292	-9497.10009	-9504.92153
**^{1}Pr$_B$**	-9499.67973	2.79866	-9497.12976	-9504.95502

Table S8: Relative (free) energies (in kcal mol^{-1}) of optimized geometries for H_2O_2 generation on a diiron center of ferritin as calculated with DFT using model B.

	ΔE [BS1]	ΔE+ZPE	ΔG [BS1]	ΔE [BS2]	ΔE+ZPE	ΔG [BS2]
**^{9}Re$_B$**	0.00	0.00	0.00	0.00	0.00	0.00
**^{9}TS1$_B$**	2.82	0.80	2.69	-2.38	-4.40	-2.51
**^{9}IM1$_B$**	-1.82	-1.28	-0.73	-12.87	-12.34	-11.78
**^{9}TS2$_B$**	16.90	13.25	13.94	16.63	12.97	13.67
**^{9}IM2$_B$**	8.87	7.75	6.53	8.22	7.10	5.88
**^{9}TS3$_B$**	10.98	7.90	7.87	13.21	10.13	10.10
**^{9}Pr$_B$**	-9.26	-8.91	-8.96	-4.87	-4.52	-4.57
^{9}**IM3**$_B$	18.40	16.89	15.74	10.00	8.48	7.34
**^{7}Re$_B$**	-8.75	-8.19	-7.86	-10.35	-9.79	-9.46
**^{7}TS1$_B$**	13.91	12.30	12.61	6.69	5.09	5.40
**^{7}IM1$_B$**	7.14	8.26	9.86	0.23	1.35	2.95
**^{7}TS2$_B$**	16.75	13.13	14.31	15.99	12.38	13.56
**^{7}IM2$_B$**	8.87	7.74	6.67	8.33	7.20	6.12
**^{7}TS3$_B$**	10.98	7.90	8.00	13.18	10.10	10.20
**^{7}Pr$_B$**	-9.26	-8.90	-8.79	-5.04	-4.68	-4.57
**^{11}Re$_B$**	0.00	0.00	-0.17	-0.06	-0.06	-0.23
**^{11}TS1$_B$**	2.82	0.80	2.57	-2.38	-4.40	-2.63
**^{11}IM1$_B$**	-1.81	-1.27	-0.69	-9.44	-8.90	-8.31
**^{11}TS2$_B$**	12.46	9.19	10.02	8.65	5.39	6.21
**^{11}IM2$_B$**	10.88	9.66	8.74	7.88	6.65	5.73
**^{11}TS3$_B$**	10.98	7.90	7.73	13.18	10.10	9.93
**^{11}Pr$_B$**	-9.26	-8.90	-9.06	-5.03	-4.68	-4.84
^{11}**TS4**$_B$	14.86	12.00	12.44	14.53	16.06	13.20
^{11}**Pr'**$_B$	14.82	13.11	12.83	12.63	14.30	12.60
**^{1}Re$_B$**	-2.12	-1.56	0.65	-4.29	-3.73	-1.52
**^{1}TS1$_B$**	13.77	12.21	13.63	5.95	4.39	5.81
**^{1}IM1$_B$**	7.11	8.28	11.13	0.15	1.32	4.17
**^{1}TS2$_B$**	59.89	58.88	62.33	64.04	63.03	66.49
**^{1}IM2$_B$**	33.93	32.84	34.41	37.47	36.38	37.95
**^{1}TS3$_B$**	60.24	58.23	60.14	68.46	66.46	68.37
**^{1}Pr$_B$**	37.71	39.31	41.52	47.44	49.04	51.26

Table S9: Absolute (in au) and relative (in kcal mol^{-1}) energies and free energies of triplet and quintet optimized geometries for H_2O_2 generation on a diiron center of ferritin as calculated with DFT using model B.

	E(BS1)	ZPE	G	ΔE [BS1]	ΔE+ZPE	ΔG [BS1]
**^{5}Re$_B$**	-9499.69571	2.79901	-9497.14290	27.68	29.50	33.28
**^{5}TS1$_B$**	-9499.69432	2.79393	-9497.14820	28.56	27.18	29.95
**^{5}IM1$_B$**	-9499.70007	2.79886	-9497.14992	24.95	26.67	28.87
**^{5}TS2$_B$**	-9499.67305	2.79191	-9497.13040	41.90	39.26	41.12
**^{5}IM2$_B$**	-9499.69480	2.79542	-9497.15141	28.25	27.82	27.94
**^{5}TS3$_B$**	-9499.69115	2.79204	-9497.14956	30.55	27.98	29.10
**^{5}Pr$_B$**	-9499.72426	2.79773	-9497.17705	9.77	10.78	11.85
**^{3}Re$_B$**	-9499.71756	2.79951	-9497.16543	13.97	16.10	19.14
**^{3}TS1$_B$**	-9499.69432	2.79395	-9497.14770	28.56	27.19	30.26
**^{3}IM1$_B$**	-9499.70831	2.79830	-9497.15937	19.78	21.14	22.94
**^{3}TS2$_B$**	-9499.65368	2.79282	-9497.10858	54.06	51.99	54.81
**^{3}IM2$_B$**	-9499.67555	2.79478	-9497.13309	40.33	39.49	39.44
**^{3}TS3$_B$**	-9499.65279	2.79228	-9497.11077	54.61	52.20	53.44
**^{3}Pr$_B$**	-9499.70772	2.79726	-9497.16078	20.15	20.86	22.06

Table S10: Group spin densities of optimized geometries for H_2O_2 generation on a diiron center of ferritin as calculated with DFT using model B. The "rest" spin is mostly located on the carboxylate groups of Asp_{65} and Glu_{62}.

	ρ(Fe1)	ρ(Fe2)	ρ(μ-H_2O)	ρ(O_2)	ρ(Tyr_{40})	ρ(Rest)	Total
**^{9}Re$_B$**	3.72	3.77	0.06	1.10	0.00	-0.66	8.00
**^{9}TS1$_B$**	4.02	3.78	0.12	0.48	0.00	-0.40	8.00
**^{9}IM1$_B$**	2.88	3.76	-0.02	0.18	0.00	1.21	8.00
**^{9}TS2$_B$**	2.89	3.73	0.04	0.03	0.00	1.30	8.00
**^{9}IM2$_B$**	3.72	3.76	0.17	0.07	-1.00	1.28	8.00
**^{9}TS3$_B$**	3.73	3.76	0.15	0.04	-1.00	1.31	8.00
**^{9}Pr$_B$**	3.73	3.77	0.13	0.00	0.99	-0.62	8.00
**^{7}Re$_B$**	3.41	3.78	0.07	-0.62	0.00	-0.63	6.00
**^{7}TS1$_B$**	3.45	3.77	0.06	-0.56	0.00	-0.72	6.00
**^{7}IM1$_B$**	2.79	3.77	0.02	0.14	0.00	-0.73	6.00
**^{7}TS2$_B$**	3.57	3.76	0.08	0.01	-0.66	-0.76	6.00
**^{7}IM2$_B$**	3.57	3.76	0.08	0.01	-0.66	-0.76	6.00
**^{7}TS3$_B$**	3.73	3.76	0.15	0.04	-1.00	-0.69	6.00
**^{7}Pr$_B$**	3.73	3.77	0.12	0.00	-0.99	-0.63	6.00
**^{5}Re$_B$**	1.48	1.99	0.00	-0.47	0.00	1.01	4.00
**^{5}TS1$_B$**	-1.10	3.79	0.06	0.08	0.00	1.18	4.00
**^{5}IM1$_B$**	2.92	1.95	-0.09	0.17	0.00	-0.94	4.00
**^{5}TS2$_B$**	3.44	1.97	-0.01	0.05	-0.56	-0.89	4.00
**^{5}IM2$_B$**	3.72	1.97	0.09	0.07	-1.00	-0.85	4.00
**^{5}TS3$_B$**	3.74	1.96	0.08	0.04	-1.00	-0.82	4.00
**^{5}Pr$_B$**	1.96	3.77	0.04	0.00	-0.99	-0.77	4.00
**^{3}Re$_B$**	3.73	0.00	0.00	-0.94	0.00	-0.79	2.00
**^{3}TS1$_B$**	-1.10	3.79	0.06	0.08	0.00	-0.82	2.00
**^{3}IM1$_B$**	-0.95	3.77	0.07	-0.03	0.00	-0.87	2.00
**^{3}TS2$_B$**	1.83	1.97	-0.02	-0.20	-0.62	-0.96	2.00
**^{3}IM2$_B$**	-0.01	3.76	0.09	0.03	-1.00	-0.88	2.00
**^{3}TS3$_B$**	1.83	1.97	-0.02	-0.20	-0.62	-0.96	2.00
**^{3}Pr$_B$**	-0.03	3.78	0.07	0.00	-0.99	-0.82	2.00
**^{1}Re$_B$**	-2.35	3.78	0.05	-0.67	0.00	-0.81	0.00
**^{1}TS1$_B$**	-3.27	3.79	0.05	0.37	0.01	-0.95	0.00
**^{1}IM1$_B$**	-2.79	3.77	0.08	-0.15	0.00	-0.92	0.00
**^{1}TS2$_B$**	-0.24	1.98	0.00	-0.15	-0.59	-0.99	0.00
**^{1}IM2$_B$**	-1.93	3.77	0.13	-0.03	-1.00	-0.94	0.00
**^{1}TS3$_B$**	1.97	0.01	-0.06	0.02	-1.00	-0.94	0.00
**^{1}Pr$_B$**	0.02	1.97	0.01	0.00	-0.99	-1.01	0.00
**^{11}Re$_B$**	3.72	3.78	0.06	1.10	0.00	1.34	10.00
**^{11}TS1$_B$**	4.02	3.78	0.12	0.48	0.00	1.60	10.00
**^{11}IM1$_B$**	4.06	3.77	0.22	0.35	0.00	1.61	10.00
**^{11}TS2$_B$**	3.78	4.01	0.58	0.08	0.00	1.56	10.00
**^{11}IM2$_B$**	3.73	3.76	0.14	0.12	0.99	1.26	10.00
**^{11}TS3$_B$**	3.91	3.77	0.17	0.21	0.59	1.35	10.00
**^{11}Pr$_B$**	3.77	3.99	0.63	0.07	0.00	1.54	10.00

Table S11: Group charges of optimized geometries for H_2O_2 generation on a diiron center of ferritin as calculated with DFT using model B.

	Q(Fe1)	Q(Fe2)	Q(μ-H_2O)	Q(O_2)	Q(Tyr_{40})	Q(Rest)	Total
**$^{9}Re_B$**	0.68	0.62	0.15	-0.60	0.00	-0.86	0.00
**$^{9}TS1_B$**	0.99	0.60	0.10	-0.87	-0.02	-0.79	0.00
**$^{9}IM1_B$**	0.86	0.58	0.13	-0.87	-0.02	-0.68	0.00
**$^{9}TS2_B$**	0.86	0.61	0.14	-0.87	-0.02	-0.73	0.00
**$^{9}IM2_B$**	0.68	0.56	0.05	-0.99	0.50	-0.80	0.00
**$^{9}TS3_B$**	0.68	0.57	0.06	-0.96	0.52	-0.86	0.00
**$^{9}Pr_B$**	0.70	0.59	0.06	-0.96	0.50	-0.89	0.00
**$^{7}Re_B$**	0.85	0.58	0.13	-0.69	-0.03	-0.84	0.00
**$^{7}TS1_B$**	0.81	0.57	0.09	-0.67	-0.03	-0.77	0.00
**$^{7}IM1_B$**	0.86	0.57	0.13	-0.87	-0.02	-0.68	0.00
**$^{7}TS2_B$**	0.79	0.57	0.06	-0.92	0.40	-0.91	0.00
**$^{7}IM2_B$**	0.79	0.57	0.06	-0.92	0.40	-0.91	0.00
**$^{7}TS3_B$**	0.68	0.57	0.06	-0.96	0.52	-0.86	0.00
**$^{7}Pr_B$**	0.68	0.57	0.06	-0.96	0.52	-0.86	0.00
**$^{5}Re_B$**	0.76	0.50	0.17	-0.74	-0.02	-0.67	0.00
**$^{5}TS1_B$**	0.75	0.59	0.15	-0.82	-0.01	-0.66	0.00
**$^{5}IM1_B$**	0.86	0.48	0.16	-0.87	-0.02	-0.60	0.00
**$^{5}TS2_B$**	0.81	0.48	0.09	-0.91	0.31	-0.79	0.00
**$^{5}IM2_B$**	0.68	0.49	0.07	-0.99	0.49	-0.74	0.00
**$^{5}TS3_B$**	0.68	0.49	0.07	-0.96	0.52	-0.80	0.00
**$^{5}Pr_B$**	0.62	0.61	0.10	-0.97	0.51	-0.86	0.00
**$^{3}Re_B$**	0.83	0.45	0.10	-0.60	-0.03	-0.74	0.00
**$^{3}TS1_B$**	0.75	0.59	0.15	-0.82	-0.01	-0.66	0.00
**$^{3}IM1_B$**	0.83	0.57	0.10	-0.82	-0.02	-0.66	0.00
**$^{3}TS2_B$**	0.70	0.48	0.10	-0.84	0.36	-0.79	0.00
**$^{3}IM2_B$**	0.61	0.57	0.07	-0.97	0.49	-0.78	0.00
**$^{3}TS3_B$**	0.70	0.48	0.10	-0.84	0.36	-0.79	0.00
**$^{3}Pr_B$**	0.65	0.61	0.08	-0.97	0.51	-0.87	0.00
**$^{1}Re_B$**	0.81	0.59	0.16	-0.77	-0.02	-0.76	0.00
**$^{1}TS1_B$**	0.88	0.60	0.10	-0.79	-0.01	-0.78	0.00
**$^{1}IM1_B$**	0.86	0.57	0.13	-0.87	-0.01	-0.68	0.00
**$^{1}TS2_B$**	0.73	0.46	0.11	-0.88	0.29	-0.71	0.00
**$^{1}IM2_B$**	0.62	0.57	0.07	-0.97	0.49	-0.78	0.00
**$^{1}TS3_B$**	0.60	0.44	0.11	-0.95	0.52	-0.71	0.00
**$^{1}Pr_B$**	0.65	0.53	0.10	-0.97	0.51	-0.81	0.00
**$^{11}Re_B$**	0.68	0.62	0.15	-0.60	0.00	-0.86	0.00
**$^{11}TS1_B$**	0.99	0.60	0.10	-0.87	-0.02	-0.79	0.00
**$^{11}IM1_B$**	0.99	0.58	0.07	-0.87	-0.02	-0.76	0.00
**$^{11}TS2_B$**	0.74	0.83	0.18	-0.94	0.00	-0.81	0.00
**$^{11}IM2_B$**	0.75	0.56	0.03	-0.97	0.53	-0.90	0.00
**$^{11}TS3_B$**	0.88	0.57	0.05	-0.93	0.32	-0.88	0.00
**$^{11}Pr_B$**	0.73	0.80	0.20	-0.92	0.00	-0.81	0.00

Table S12: Relative (in kcal mol^{-1}) energies and free energies of selected structures along the mechanism of H_2O_2 generation on a diiron center of ferritin as calculated with DFT using model B. Geometries calculated at UB3LYP/BS1 followed by a single point UB3LYP/BS2+E_{solv} or UB3LYP-D3/BS2+E_{solv} calculation.

	ΔE	ΔE+ZPE	ΔG	ΔE	ΔE+ZPE	ΔG	ΔE	ΔE+ZPE	ΔG
	BS1	BS1	BS1	BS2+E_{solv}	BS2+E_{solv}	BS2+E_{solv}	GD3	GD3	GD3
^{11}**Re**$_B$	0.00	0.00	0.00	0.00	0.00	0.00	0.00	0.00	0.00
^{11}**TS1**$_B$	2.82	0.79	2.70	2.85	0.83	2.74	6.12	4.10	6.01
^{11}**IM1**$_B$	-1.82	-1.27	-0.55	-5.36	-4.81	-4.09	-2.37	-1.83	-1.11
^{11}**TS4**$_B$	14.86	12.00	12.44	16.06	13.20	13.63	22.70	19.84	20.27
^{11}**Pr'**$_B$	14.82	13.11	12.83	14.30	12.60	12.32	21.54	19.84	19.56

Part VI: Data on extra-large model C.

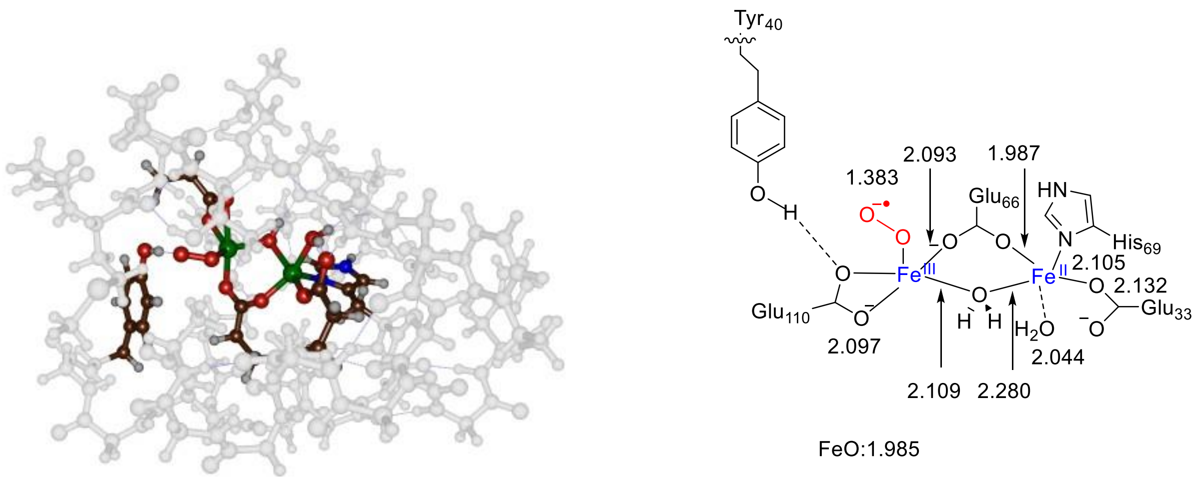

^{11}**Re**$_{C}$

Figure S29: UB3LYP/BS1 optimized geometries of ^{11}Re$_{C}$ as obtained in Gaussian-09 using Model C. Bond lengths are in angstroms.

Part VII: Data on large model BP.

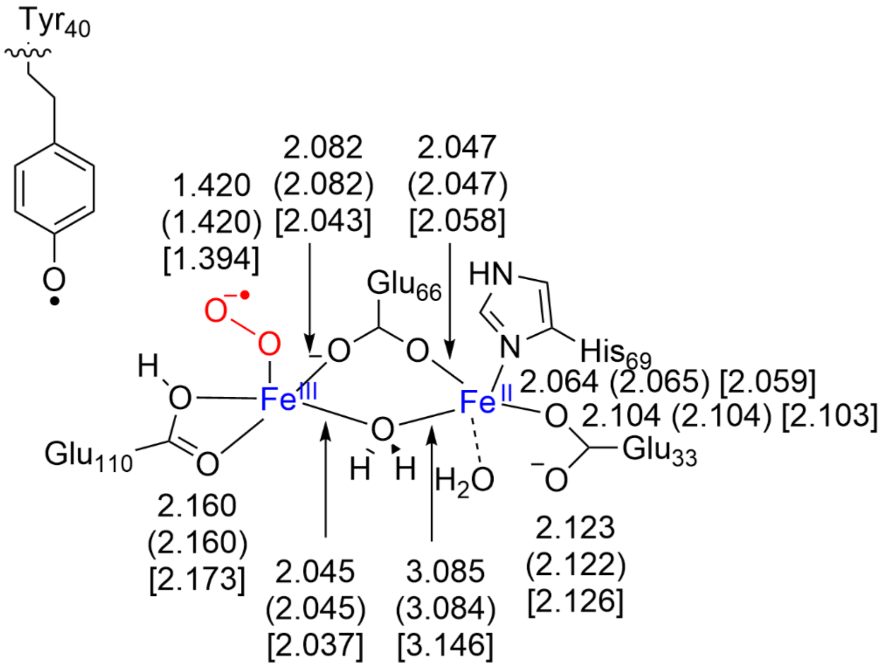

FeO: 2.120 (2.120) [2.046]

^{11}**Re**$_{BP}$ (^{9}**Re**$_{BP}$) [^{7}**Re**$_{BP}$]

Figure S30: UB3LYP/BS1 optimized geometries of ^{11,9,7}Re$_{BP}$ as obtained in Gaussian-09 using Model BP. Bond lengths are in angstroms.

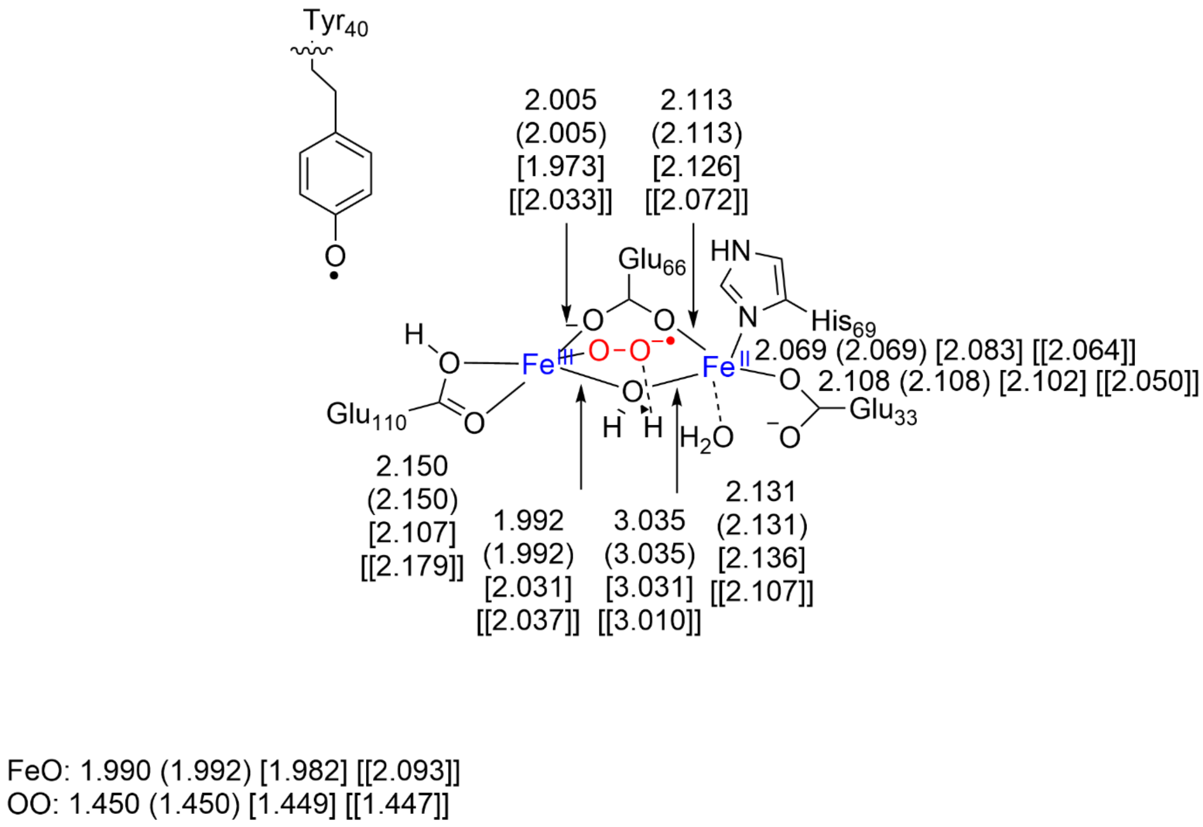

^{11}**TS1**$_{BP}$ (^{9}**TS1**$_{BP}$) [^{7}**TS1**$_{BP}$] [[^{1}**TS1**$_{BP}$]]

Figure S31: UB3LYP/BS1 optimized geometries of ^{11,9,7,1}TS1$_{BP}$ as obtained in Gaussian-09 using Model B. Bond lengths are in angstroms.

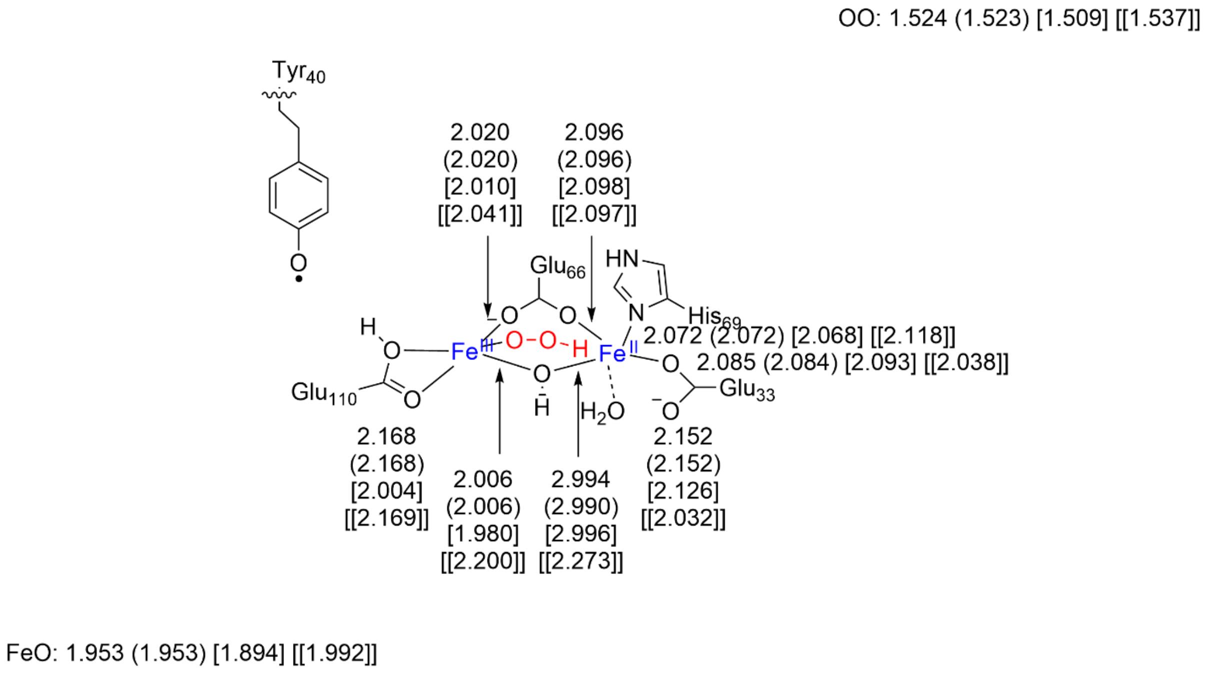

^{11}**IM1**$_{BP}$ (^{9}**IM1**$_{BP}$) [^{7}**IM1**$_{BP}$] [[^{1}**IM1**$_{BP}$]]

Figure S32: UB3LYP/BS1 optimized geometries of ^{11,9,7,1}IM1$_{BP}$ as obtained in Gaussian-09 using Model B. Bond lengths are in angstroms.

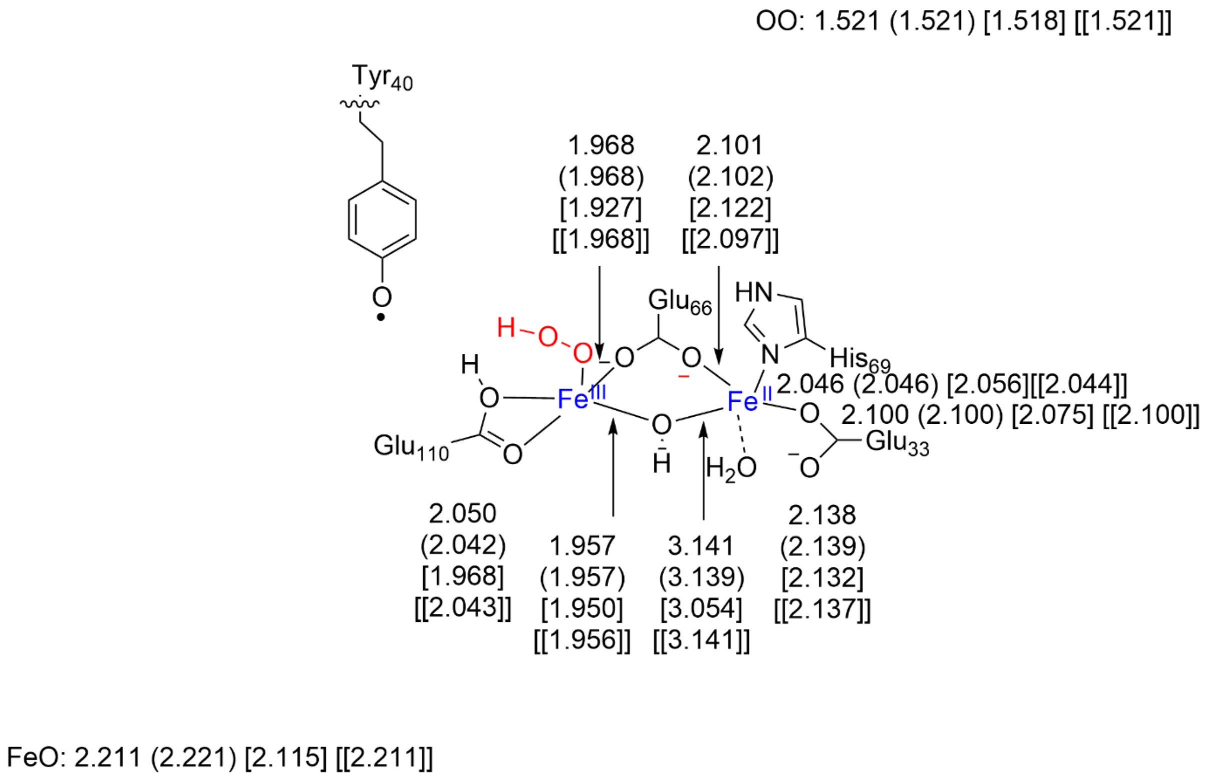

^{11}**TS2**$_{BP}$ (^{9}**TS2**$_{BP}$) [^{7}**TS2**$_{BP}$] [[^{1}**TS2**$_{BP}$]]

Figure S33: UB3LYP/BS1 optimized geometries of ^{11,9,7,1}TS2$_{BP}$ as obtained in Gaussian-09 using Model B. Bond lengths are in angstroms.

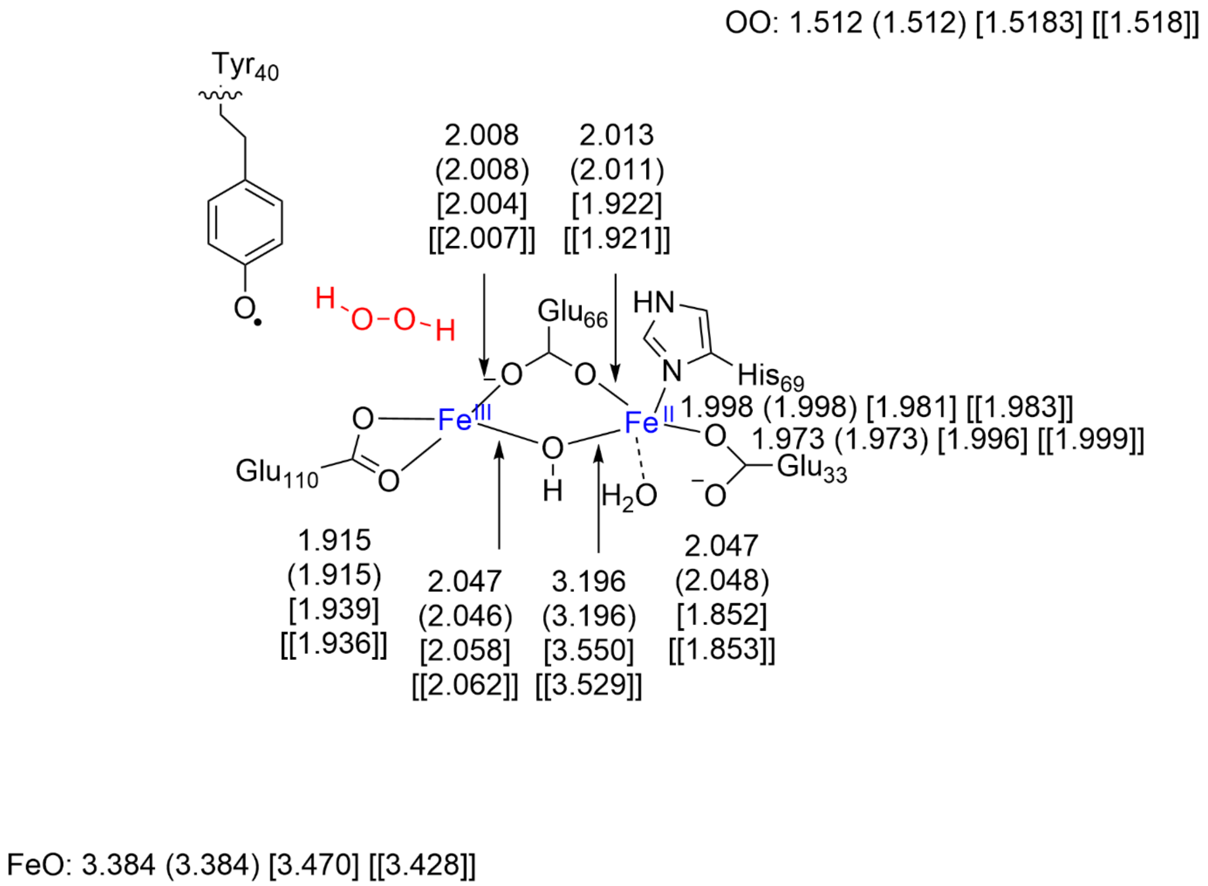

^{11}**Pr**$_{BP}$ (^{9}**Pr**$_{BP}$) [^{7}**Pr**$_{BP}$] [[^{1}**Pr**$_{BP}$]]

Figure S34: UB3LYP/BS1 optimized geometries of ^{11,9,7,1}Pr$_{BP}$ as obtained in Gaussian-09 using Model B. Bond lengths are in angstroms.

Table S13: Absolute (free) energies (in au) of optimized geometries for H_2O_2 generation on a diiron center of ferritin as calculated with DFT using model BP.

	BS1	ZPE	G	BS2
**^{11}Re$_{BP}$**	-9500.24365	2.80876	-9497.68538	-9505.53324
**^{11}TS1$_{BP}$**	-9500.20997	2.80361	-9497.65728	-9505.51198
**^{11}IM1$_{BP}$**	-9500.21927	2.80780	-9497.65995	-9505.52515
**^{11}TS2$_{BP}$**	-9500.18203	2.80143	-9497.63550	-9505.49217
**^{11}Pr$_{BP}$**	-9500.22725	2.80849	-9497.66592	-9505.52100
**^{9}Re$_{BP}$**	-9500.24364	2.80875	-9497.68516	-9505.53333
**^{9}TS1$_{BP}$**	-9500.20996	2.80360	-9497.65709	-9505.51195
**^{9}IM1$_{BP}$**	-9500.21928	2.80785	-9497.66254	-9505.52495
**^{9}TS2$_{BP}$**	-9500.18202	2.80143	-9497.63553	-9505.49217
**^{9}Pr$_{BP}$**	-9500.22725	2.80851	-9497.66580	-9505.52124
**^{7}Re$_{BP}$**	-9500.24338	2.80882	-9497.68341	-9505.53102
**^{7}TS1$_{BP}$**	-9500.19479	2.80631	-9497.63866	-9505.49900
**^{7}IM1$_{BP}$**	-9500.20215	2.80825	-9497.64399	-9505.50150
**^{7}TS2$_{BP}$**	-9500.17445	2.80487	-9497.62171	-9505.46179
**^{7}Pr$_{BP}$**	-9500.21691	2.80880	-9497.65350	-9505.49607
**^{1}Re$_{BP}$**	-9500.24375	2.80876	-9497.68308	-9505.53337
**^{1}TS1$_{BP}$**	-9500.20008	2.80571	-9497.64339	-9505.51501
**^{1}IM1$_{BP}$**	-9500.22533	2.80916	-9497.66109	-9505.52385
**^{1}TS2$_{BP}$**	-9500.15497	2.80039	-9497.60772	-9505.46551
**^{1}Pr$_{BP}$**	-9500.21784	2.80806	-9497.65368	-9505.49710

Table S14: Relative (free) energies (in kcal mol^{-1}) of optimized geometries for H_2O_2 generation on a diiron center of ferritin as calculated with DFT using model BP.

	ΔE [BS1]	ΔE+ZPE	ΔG [BS1]	ΔE [BS2]	ΔE+ZPE	ΔG [BS2]
**^{11}Re$_{BP}$**	0.00	0.00	0.00	0.00	0.00	0.00
**^{11}TS1$_{BP}$**	21.13	17.90	17.63	13.34	10.11	9.84
**^{11}IM1$_{BP}$**	15.30	14.69	15.96	5.07	4.47	5.73
**^{11}TS2$_{BP}$**	38.67	34.07	31.30	25.77	21.17	18.41
**^{11}Pr$_{BP}$**	10.29	10.12	12.21	7.68	7.51	9.60
**^{9}Re$_{BP}$**	0.00	0.00	0.14	-0.05	-0.06	0.08
**^{9}TS1$_{BP}$**	21.14	17.90	17.75	13.36	10.12	9.97
**^{9}IM1$_{BP}$**	15.29	14.72	14.33	5.21	4.63	4.25
**^{9}TS2$_{BP}$**	38.68	34.07	31.28	25.77	21.16	18.38
**^{9}Pr$_{BP}$**	10.29	10.13	12.29	7.53	7.37	9.53
**^{7}Re$_{BP}$**	0.17	0.21	1.24	1.39	1.43	2.46
**^{7}TS1$_{BP}$**	30.66	29.12	29.32	21.49	19.95	20.15
**^{7}IM1$_{BP}$**	26.04	25.72	25.97	19.91	19.59	19.84
**^{7}TS2$_{BP}$**	43.42	40.98	39.96	44.83	42.39	41.37
**^{7}Pr$_{BP}$**	16.78	16.80	20.01	23.33	23.35	26.55
**^{1}Re$_{BP}$**	-0.06	-0.07	1.45	-0.08	-0.08	1.43
**^{1}TS1$_{BP}$**	27.34	25.42	26.35	11.44	9.52	10.45
**^{1}IM1$_{BP}$**	11.50	11.75	15.24	5.89	6.14	9.64
**^{1}TS2$_{BP}$**	55.65	50.40	48.73	42.50	37.24	35.58
**^{1}Pr$_{BP}$**	16.20	15.76	19.89	22.68	22.24	26.38

Table S15: Group spin densities of optimized geometries for H_2O_2 generation on a diiron center of ferritin as calculated with DFT using model BP.

	ρ(Fe1)	ρ(Fe2)	ρ(μ-H_2O)	ρ(O_2)	ρ(Tyr_{40})	ρ(Rest)	Total
**$^9Re_{BP}$**	3.76	3.77	0.06	1.07	-0.99	0.33	8.00
**$^9TS1_{BP}$**	3.90	3.78	0.14	0.76	-0.99	0.41	8.00
**$^9IM1_{BP}$**	4.05	3.78	0.32	0.34	-0.99	0.49	8.00
**$^9TS2_{BP}$**	4.03	3.79	0.37	0.13	-1.00	0.67	8.00
**$^9Pr_{BP}$**	3.74	4.10	0.27	0.01	-0.99	0.87	8.00
**$^7Re_{BP}$**	3.74	3.77	0.05	-0.91	-0.99	0.33	6.00
**$^7TS1_{BP}$**	2.69	3.77	-0.01	0.22	-0.99	0.32	6.00
**$^7IM1_{BP}$**	3.03	3.75	-0.02	-0.09	-0.99	0.33	6.00
**$^7TS2_{BP}$**	2.80	3.76	0.03	0.03	-1.00	0.37	6.00
**$^7Pr_{BP}$**	3.72	2.92	0.09	0.00	-0.99	0.26	6.00
**$^{11}Re_{BP}$**	3.78	3.78	0.06	1.04	0.99	0.35	10.00
**$^{11}TS1_{BP}$**	3.80	3.77	0.10	0.97	0.99	0.36	10.00
**$^{11}IM1_{BP}$**	4.05	3.91	0.23	0.39	0.40	1.02	10.00
**$^{11}TS2_{BP}$**	4.03	3.79	0.37	0.13	1.00	0.67	10.00
**$^{11}Pr_{BP}$**	3.82	3.97	0.18	0.01	0.99	1.04	10.00
**$^1Re_{BP}$**	-3.74	3.78	0.01	-1.12	0.99	0.07	0.00
**$^1TS1_{BP}$**	-3.75	3.96	0.08	0.55	-0.99	0.16	0.00
**$^1IM1_{BP}$**	-3.17	3.87	0.20	-0.01	-0.99	0.10	0.00
**$^1TS2_{BP}$**	-3.30	3.90	0.26	-0.05	-1.00	0.19	0.00
**$^1Pr_{BP}$**	-3.30	3.90	0.26	-0.05	-1.00	0.19	0.00

Table S16: Group charges of optimized geometries for H_2O_2 generation on a diiron center of ferritin as calculated with DFT using model BP.

	Q(Fe1)	Q(Fe2)	Q(μ-H_2O)	Q(O_2)	Q(Tyr_{40})	Q(Rest)	Total
**$^9Re_{BP}$**	0.80	0.62	0.13	-0.59	0.56	-0.53	1.00
**$^9TS1_{BP}$**	0.90	0.62	0.10	-0.70	0.58	-0.50	1.00
**$^9IM1_{BP}$**	0.99	0.63	0.11	-0.86	0.57	-0.44	1.00
**$^9TS2_{BP}$**	0.95	0.63	0.13	-0.89	0.51	-0.33	1.00
**$^9Pr_{BP}$**	0.72	0.90	0.10	-0.96	0.51	-0.28	1.00
**$^7Re_{BP}$**	0.79	0.62	0.13	-0.57	0.56	-0.54	1.00
**$^7TS1_{BP}$**	0.87	0.62	0.12	-0.84	0.60	-0.37	1.00
**$^7IM1_{BP}$**	0.88	0.62	0.16	-0.82	0.57	-0.41	1.00
**$^7TS2_{BP}$**	0.83	0.62	0.20	-0.90	0.50	-0.24	1.00
**$^7Pr_{BP}$**	0.71	0.78	0.12	-0.95	0.51	-0.16	1.00
**$^{11}Re_{BP}$**	0.82	0.62	0.13	-0.62	0.56	-0.51	1.00
**$^{11}TS1_{BP}$**	0.82	0.62	0.10	-0.58	0.57	-0.53	1.00
**$^{11}IM1_{BP}$**	0.99	0.65	0.09	-0.85	0.34	-0.22	1.00
**$^{11}TS2_{BP}$**	0.95	0.63	0.13	-0.89	0.51	-0.33	1.00
**$^{11}Pr_{BP}$**	0.79	0.71	0.08	-0.96	0.51	-0.13	1.00
**$^1Re_{BP}$**	0.79	0.63	0.13	-0.55	0.56	-0.55	1.00
**$^1TS1_{BP}$**	0.78	0.73	0.08	-0.69	0.56	-0.46	1.00
**$^1IM1_{BP}$**	0.87	0.66	0.16	-0.86	0.57	-0.40	1.00
**$^1TS2_{BP}$**	0.84	0.68	0.17	-0.91	0.50	-0.29	1.00
**$^1Pr_{BP}$**	0.84	0.68	0.17	-0.91	0.50	-0.29	1.00

Table S17: Absolute (in au) and relative energies (in kcal mol^{-1}) of single point BS2 + solvent calculations on UB3LYP/BS1 optimized geometries of reactant complexes for H_2O_2 generation on a diiron center of ferritin as calculated with DFT using model BP.

	E [au]	ΔE	E [au]	ΔE	E [au]	ΔE
	B3LYP/BS2	B3LYP	B3LYP-D3	B3LYP-D3	PBE0	PBE0
^{11}**Re**$_{BP}$	-9505.53324	0.00	-9506.17942	0.00	-9495.054455	0.00
^{9}**Re**$_{BP}$	-9505.53333	-0.05	-9506.17946	-0.02	-9495.054569	-0.07
^{7}**Re**$_{BP}$	-9505.53102	1.39	-9506.17920	0.13	-9495.053781	0.42
^{1}**Re**$_{BP}$	-9505.53337	-0.08	-9506.17961	-0.12	-9495.054601	-0.09

Part VIII: Cartesian coordinates of optimized structures.

Model A1 structures

$^{11}Re_{A1}$:

6	8.638257000	-5.564078000	-0.961226000
6	7.526765000	-5.495884000	0.103704000
6	6.574137000	-4.355705000	-0.077105000
7	5.746107000	-4.235166000	-1.194846000
6	6.350998000	-3.258003000	0.699198000
6	4.820008000	-3.155941000	-1.001386000
7	5.387165000	-2.470890000	0.108433000
1	8.220803000	-5.665210000	-1.969190000
1	6.970518000	-6.446070000	0.101462000
1	7.975650000	-5.410754000	1.099707000
1	6.830377000	-2.969260000	1.623091000
1	4.644258000	-2.535999000	-1.890682000
1	4.929182000	-1.693286000	0.588615000
6	3.512991000	3.070700000	-0.274283000
6	3.563000000	3.098608000	1.257940000
6	4.167534000	1.827372000	1.866380000
6	3.347254000	0.555763000	1.664952000
8	2.090965000	0.682107000	1.499347000
8	3.951958000	-0.557547000	1.722800000
1	2.870459000	2.257454000	-0.629837000
1	4.161305000	3.958175000	1.588236000
1	2.553534000	3.239220000	1.660019000
1	4.277511000	1.949211000	2.953914000
1	5.176480000	1.639150000	1.480515000
6	-3.107537000	5.666563000	-4.322822000
6	-2.870897000	4.999393000	-5.690150000
6	-2.266031000	3.613248000	-5.591377000
6	-0.886527000	3.406940000	-5.729501000
6	-3.067737000	2.489879000	-5.342839000
6	-0.323503000	2.134097000	-5.630022000
6	-2.525328000	1.209807000	-5.236308000
6	-1.144564000	1.029167000	-5.383855000
8	-0.562783000	-0.210422000	-5.314399000
1	-2.165923000	5.774221000	-3.771460000
1	-3.826608000	4.944919000	-6.227974000
1	-2.215654000	5.641254000	-6.292551000
1	-0.237557000	4.256236000	-5.933826000
1	-4.144104000	2.613906000	-5.239445000
1	0.744302000	1.982206000	-5.760266000
1	-3.161593000	0.350285000	-5.044558000
1	-1.244523000	-0.898998000	-5.095249000
6	-1.688097000	2.986225000	0.703119000
6	-1.364883000	3.805270000	1.943447000
8	-0.448738000	3.525937000	2.714276000
6	-0.605154000	3.107433000	-0.385838000
6	-0.913679000	2.230321000	-1.610784000
6	-0.672725000	0.744126000	-1.386528000
8	0.448844000	0.405275000	-0.863896000
8	-1.541904000	-0.082218000	-1.777101000
1	-1.760592000	1.942699000	1.028394000
1	0.362873000	2.822517000	0.035389000
1	-0.530483000	4.153405000	-0.709018000
1	-1.938011000	2.370170000	-1.968190000
1	-0.251008000	2.521250000	-2.436979000
7	-2.187612000	4.878528000	2.182789000
6	-1.931518000	5.790400000	3.288509000
6	-2.283000000	5.255232000	4.688031000
8	-1.917874000	5.861196000	5.684729000
1	-2.872939000	5.133907000	1.486193000
1	-0.869513000	6.049634000	3.324782000
7	-3.008307000	4.100550000	4.709567000
6	-3.280922000	3.355822000	5.922008000
6	-2.552677000	2.004667000	6.020137000
8	-3.067804000	1.077195000	6.642288000
1	-3.231361000	3.674348000	3.821250000
1	-2.971548000	3.989160000	6.759689000
7	-1.338538000	1.936654000	5.415679000
6	-0.499313000	0.747114000	5.483081000
6	-0.089417000	0.212820000	4.105674000
6	-1.227694000	-0.253815000	3.247405000
7	-1.030178000	-0.539763000	1.898163000
6	-2.551808000	-0.469550000	3.547612000
6	-2.213107000	-0.905605000	1.413309000
7	-3.153090000	-0.873384000	2.368195000
1	-1.015412000	2.715121000	4.850898000
1	-1.061315000	0.003713000	6.052998000
1	0.465351000	0.982525000	3.555782000
1	0.615946000	-0.615975000	4.261317000
1	-3.096481000	-0.357581000	4.473692000
1	-2.422940000	-1.190534000	0.398258000
1	-4.101940000	-1.190575000	2.164070000
6	-2.563526000	-3.525947000	-6.729442000
6	-2.056590000	-4.261761000	-5.488718000
6	-1.711456000	-3.365051000	-4.314991000
8	-2.100290000	-2.146433000	-4.280564000
8	-1.048829000	-3.842878000	-3.344037000
1	-3.460048000	-2.942653000	-6.500324000
1	-2.809533000	-4.238829000	-7.523845000
1	-1.169259000	-4.865754000	-5.711094000
1	-2.812440000	-4.971301000	-5.123663000
26	0.766466000	-0.758986000	0.805164000
26	-1.101885000	-2.071890000	-2.230535000
8	1.920144000	-2.191234000	1.740406000
1	2.792578000	-1.689552000	1.848313000
1	2.101540000	-2.990184000	1.180944000
8	-2.763607000	-2.855693000	-1.003531000
8	0.000034000	-2.595301000	-0.422938000
8	0.778312000	-1.313597000	-3.145862000
1	0.525059000	-0.812833000	-3.956103000
1	1.064134000	-0.651469000	-2.487281000
8	-2.251646000	-3.662408000	-0.065061000
8	2.303299000	-4.003227000	-0.212382000
1	2.313648000	-4.968468000	-0.112238000
1	3.251484000	-3.717353000	-0.577866000
8	-5.011134000	-1.981024000	0.536803000
1	-4.357614000	-2.459632000	-0.020794000
1	-5.470598000	-1.402266000	-0.089434000
1	-1.807143000	-2.835376000	-7.118469000
1	-3.548207000	6.663502000	-4.441804000
1	-3.787611000	5.066642000	-3.706518000
1	9.292912000	-6.423338000	-0.779352000
1	9.248947000	-4.655349000	-0.944594000
1	4.513929000	2.927577000	-0.702132000
1	3.115313000	4.011365000	-0.673723000
1	0.411055000	0.977602000	6.051585000
1	-4.349456000	3.147356000	6.028911000
1	-2.497823000	6.711284000	3.123532000
1	-2.664994000	3.271782000	0.290498000
1	5.482205000	-5.043584000	-1.741800000
1	0.783992000	-3.193244000	-0.548011000
1	-0.798807000	-3.175114000	-0.097949000

$^{11}TS1_{A1}$:

6	8.458869000	-4.319433000	-1.687887000
6	7.340376000	-4.443239000	-0.635280000
6	6.366606000	-3.307090000	-0.650393000
7	5.523248000	-3.055178000	-1.736195000
6	6.150129000	-2.314330000	0.257023000
6	4.599211000	-2.010641000	-1.399728000
7	5.176375000	-1.465516000	-0.221411000
1	8.047402000	-4.266724000	-2.701879000
1	6.805022000	-5.392718000	-0.790103000
1	7.781572000	-4.503984000	0.365998000
1	6.643445000	-2.137685000	1.201701000
1	4.409573000	-1.286769000	-2.203960000
1	4.713827000	-0.751299000	0.345027000
6	3.490645000	4.105116000	-0.814555000
6	3.489036000	4.078009000	0.718505000
6	4.037259000	2.774889000	1.312329000

6	3.187715000	1.525623000	1.093989000
8	1.995462000	1.695754000	0.656985000
8	3.682928000	0.405367000	1.385908000
1	2.820861000	3.338975000	-1.217907000
1	4.101335000	4.907358000	1.097446000
1	2.470746000	4.239656000	1.089859000
1	4.152611000	2.872442000	2.401205000
1	5.043123000	2.555359000	0.931148000
6	-3.308163000	6.914597000	-5.061896000
6	-3.045948000	6.259397000	-6.430313000
6	-2.388221000	4.898280000	-6.328027000
6	-0.995918000	4.754343000	-6.403778000
6	-3.150683000	3.738795000	-6.128700000
6	-0.382908000	3.506324000	-6.288866000
6	-2.558097000	2.482472000	-6.008040000
6	-1.165736000	2.364722000	-6.090223000
8	-0.531857000	1.151313000	-5.999573000
1	-2.371533000	7.056740000	-4.509927000
1	-3.998136000	6.167538000	-6.969006000
1	-2.415286000	6.926646000	-7.031545000
1	-0.376102000	5.633514000	-6.568522000
1	-4.234892000	3.815535000	-6.072332000
1	0.695604000	3.402431000	-6.367972000
1	-3.163781000	1.594359000	-5.851036000
1	-1.187618000	0.435159000	-5.792551000
6	-1.817695000	4.282931000	0.203355000
6	-1.359138000	5.027299000	1.447747000
8	-0.411268000	4.662368000	2.140227000
6	-0.905940000	4.499616000	-1.020065000
6	-1.327643000	3.617574000	-2.205406000
6	-0.984114000	2.141640000	-2.032585000
8	0.120642000	1.844909000	-1.482840000
8	-1.789181000	1.278369000	-2.507199000
1	-1.812409000	3.221733000	0.469342000
1	0.127170000	4.267333000	-0.747085000
1	-0.941609000	5.553604000	-1.323872000
1	-2.396370000	3.707025000	-2.424604000
1	-0.801144000	3.945194000	-3.112864000
7	-2.103500000	6.129689000	1.793926000
6	-1.747231000	6.949369000	2.941866000
6	-2.114229000	6.363468000	4.317844000
8	-1.707061000	6.895703000	5.340154000
1	-2.824656000	6.452505000	1.164789000
1	-0.666255000	7.113536000	2.962135000
7	-2.898818000	5.248146000	4.288814000
6	-3.222529000	4.468596000	5.466244000
6	-2.598176000	3.063198000	5.492262000
8	-3.182497000	2.142484000	6.060099000
1	-3.162640000	4.888729000	3.382461000
1	-2.861216000	5.035533000	6.330402000
7	-1.385972000	2.942361000	4.891453000
6	-0.634501000	1.694109000	4.891466000
6	-0.259285000	1.213322000	3.484996000
6	-1.424937000	0.895556000	2.595606000
7	-1.249827000	0.779653000	1.220665000
6	-2.753636000	0.694967000	2.885166000
6	-2.448430000	0.525407000	0.707207000
7	-3.380007000	0.466412000	1.672388000
1	-1.008925000	3.727383000	4.372046000
1	-1.251450000	0.961739000	5.417159000
1	0.352082000	1.976118000	2.986324000
1	0.379154000	0.324398000	3.582413000
1	-3.288810000	0.724194000	3.822948000
1	-2.675657000	0.385505000	-0.334959000
1	-4.331367000	0.173458000	1.457859000
6	-2.297918000	-2.287592000	-7.393556000
6	-1.808215000	-2.974114000	-6.118120000
6	-1.558274000	-2.039974000	-4.948823000
8	-2.006396000	-0.839533000	-4.959971000
8	-0.920564000	-2.462606000	-3.938872000
1	-3.231289000	-1.746272000	-7.213639000
1	-2.474248000	-3.026072000	-8.183118000
1	-0.882979000	-3.535844000	-6.291646000
1	-2.542373000	-3.714914000	-5.771368000
26	0.545903000	0.437668000	0.129696000
26	-1.105901000	-0.627642000	-2.902071000
8	1.465550000	-1.106463000	1.393086000
1	2.350461000	-0.666808000	1.534840000
1	1.662986000	-1.889325000	0.826129000
8	-2.772922000	-1.571504000	-1.678560000
8	-0.078855000	-1.190077000	-1.088230000
8	0.750634000	0.221862000	-3.727677000
1	0.517325000	0.673763000	-4.572329000
1	0.930640000	0.922925000	-3.069828000
8	-2.206044000	-2.237762000	-0.670887000
8	2.025010000	-2.809943000	-0.699430000
1	1.930064000	-3.770637000	-0.797648000
1	2.993405000	-2.562528000	-1.011771000
8	-5.139012000	-0.639819000	-0.286112000
1	-4.460075000	-1.149760000	-0.776626000
1	-5.581994000	-0.118943000	-0.972191000
1	-1.561091000	-1.566734000	-7.764643000
1	-3.786291000	7.894445000	-5.178707000
1	-3.964591000	6.287520000	-4.447146000
1	9.132341000	-5.182333000	-1.640113000
1	9.048423000	-3.411846000	-1.521497000
1	4.498132000	3.929399000	-1.214570000
1	3.153044000	5.078659000	-1.189911000
1	0.287182000	1.824181000	5.473453000
1	-4.303363000	4.336560000	5.572231000
1	-2.233755000	7.924224000	2.847571000
1	-2.850149000	4.556847000	-0.053540000
1	5.243160000	-3.799685000	-2.360921000
1	0.677531000	-1.834330000	-1.170173000
1	-1.013968000	-1.800515000	-0.757246000

^{11}IM1$_{A1}$:

6	8.458869000	-4.319433000	-1.687887000
6	7.451567000	-4.266251000	-0.523572000
6	6.494193000	-3.119438000	-0.602653000
7	5.576683000	-2.979263000	-1.649552000
6	6.351194000	-2.028119000	0.198331000
6	4.685788000	-1.892071000	-1.364092000
7	5.352590000	-1.220982000	-0.304626000
1	7.947767000	-4.398671000	-2.653758000
1	6.892842000	-5.214632000	-0.490088000
1	7.990350000	-4.198782000	0.428355000
1	6.913105000	-1.753874000	1.079333000
1	4.425833000	-1.265344000	-2.227338000
1	4.913960000	-0.465412000	0.226114000
6	3.351106000	4.199364000	-1.326947000
6	3.618942000	4.315870000	0.177980000
6	4.228565000	3.055430000	0.802348000
6	3.334165000	1.818869000	0.858990000
8	2.090275000	1.974357000	0.590821000
8	3.855452000	0.725226000	1.196835000
1	2.584172000	3.444813000	-1.526410000
1	4.308214000	5.151089000	0.361425000
1	2.684876000	4.557043000	0.697911000
1	4.531262000	3.254358000	1.840098000
1	5.150203000	2.763750000	0.281664000
6	-3.308163000	6.914597000	-5.061896000
6	-3.021015000	6.203204000	-6.396667000
6	-2.301883000	4.880678000	-6.226263000
6	-0.901464000	4.809239000	-6.231323000
6	-3.012626000	3.689146000	-6.028278000
6	-0.231279000	3.599918000	-6.049995000
6	-2.361598000	2.469817000	-5.841341000
6	-0.963464000	2.425154000	-5.854037000
8	-0.269164000	1.251347000	-5.693972000
1	-2.377581000	7.126022000	-4.522318000
1	-3.968734000	6.039827000	-6.925732000
1	-2.422577000	6.866721000	-7.033980000
1	-0.320992000	5.715595000	-6.391152000
1	-4.100620000	3.709962000	-6.024578000
1	0.853602000	3.550844000	-6.070592000
1	-2.930056000	1.557014000	-5.684308000
1	-0.886125000	0.504020000	-5.496343000
6	-2.435725000	4.086953000	0.346319000
6	-2.200261000	4.941575000	1.581523000

8	-1.204098000	4.833577000	2.294079000
6	-1.572441000	4.513912000	-0.857230000
6	-1.767852000	3.580911000	-2.060952000
6	-1.161204000	2.194991000	-1.870844000
8	-0.063048000	2.095576000	-1.252428000
8	-1.771393000	1.207558000	-2.400002000
1	-2.175896000	3.061706000	0.627559000
1	-0.519131000	4.510200000	-0.563028000
1	-1.831583000	5.538635000	-1.153124000
1	-2.824325000	3.468498000	-2.324453000
1	-1.274044000	4.011366000	-2.943255000
7	-3.194242000	5.839128000	1.889613000
6	-3.038297000	6.787369000	2.983671000
6	-3.191749000	6.203808000	4.399553000
8	-2.887527000	6.875711000	5.374348000
1	-3.952688000	5.969540000	1.235376000
1	-2.042473000	7.239048000	2.952621000
7	-3.679266000	4.931812000	4.461044000
6	-3.727552000	4.158551000	5.685390000
6	-2.743296000	2.977658000	5.737216000
8	-3.021790000	1.979113000	6.399008000
1	-3.874050000	4.466322000	3.585870000
1	-3.498456000	4.847933000	6.504363000
7	-1.583318000	3.139988000	5.050418000
6	-0.530451000	2.132936000	5.047524000
6	-0.148054000	1.663586000	3.638625000
6	-1.254988000	0.998385000	2.875792000
7	-1.146197000	0.805904000	1.504657000
6	-2.465452000	0.496860000	3.293367000
6	-2.268330000	0.211001000	1.115479000
7	-3.090347000	0.008455000	2.160912000
1	-1.465120000	3.952583000	4.454351000
1	-0.890603000	1.305733000	5.663457000
1	0.209217000	2.519557000	3.052358000
1	0.700667000	0.970776000	3.724239000
1	-2.925149000	0.470651000	4.270530000
1	-2.511606000	-0.086447000	0.108174000
1	-3.932877000	-0.559897000	2.055945000
6	-1.147747000	-2.088985000	-7.204538000
6	-0.768369000	-2.850270000	-5.932296000
6	-0.792540000	-2.021698000	-4.665772000
8	-1.459171000	-0.915083000	-4.617298000
8	-0.188362000	-2.405884000	-3.631157000
1	-2.164850000	-1.692141000	-7.135890000
1	-1.097203000	-2.752637000	-8.074045000
1	0.228891000	-3.297305000	-6.009584000
1	-1.460519000	-3.687874000	-5.765872000
26	0.617101000	0.667406000	0.302774000
26	-0.938520000	-0.651165000	-2.560495000
8	1.665387000	-0.771487000	1.618793000
1	2.556622000	-0.325067000	1.612968000
1	1.807470000	-1.592819000	1.092991000
8	-2.434675000	-1.576814000	-1.802799000
8	0.072448000	-0.935161000	-0.875410000
8	0.885611000	0.407483000	-3.302086000
1	0.680445000	0.831621000	-4.167767000
1	0.919595000	1.134245000	-2.645307000
8	-2.127142000	-2.421845000	-0.707427000
8	2.152121000	-2.598495000	-0.401688000
1	2.007535000	-3.549232000	-0.529790000
1	3.092879000	-2.384441000	-0.788962000
8	-4.571503000	-1.896991000	0.750032000
1	-3.740689000	-2.236046000	0.355777000
1	-5.098537000	-1.641589000	-0.022088000
1	-0.469463000	-1.247395000	-7.382094000
1	-3.829265000	7.865263000	-5.225876000
1	-3.934723000	6.291079000	-4.413296000
1	9.123876000	-5.184209000	-1.585410000
1	9.072884000	-3.413015000	-1.711501000
1	4.262679000	3.922731000	-1.873069000
1	2.997873000	5.153385000	-1.736107000
1	0.364493000	2.540476000	5.535154000
1	-4.726247000	3.746116000	5.856217000
1	-3.774716000	7.586956000	2.863971000
1	-3.497275000	4.098616000	0.063440000
1	5.238677000	-3.790611000	-2.150603000
1	0.833673000	-1.570400000	-0.930283000
1	-1.231884000	-2.059494000	-0.448753000

^{11}TS4$_{A1}$:

6	7.806569000	-1.678994000	-0.211617000
6	7.361226000	-3.064687000	0.289370000
6	5.970947000	-3.087082000	0.844234000
7	4.847659000	-2.801379000	0.074190000
6	5.536050000	-3.432725000	2.089455000
6	3.663225000	-2.968282000	0.843785000
7	4.151908000	-3.375297000	2.115361000
1	7.156910000	-1.319097000	-1.017121000
1	7.438561000	-3.785461000	-0.537626000
1	8.048710000	-3.411503000	1.069336000
1	6.111034000	-3.786399000	2.933012000
1	2.979142000	-2.115197000	0.842148000
1	3.577361000	-3.261031000	2.942358000
6	4.149765000	0.337226000	4.056512000
6	2.737692000	0.811777000	4.416567000
6	1.736195000	-0.334227000	4.638907000
6	1.426776000	-1.191210000	3.413095000
8	0.989099000	-0.570947000	2.363615000
8	1.562364000	-2.433606000	3.489488000
1	4.165772000	-0.196383000	3.100133000
1	2.777110000	1.407787000	5.337318000
1	2.354191000	1.482778000	3.638120000
1	0.777313000	0.088592000	4.968745000
1	2.091060000	-1.002891000	5.429423000
6	-1.522005000	7.393810000	-0.069809000
6	-0.050755000	7.843074000	-0.004796000
6	0.920895000	6.684686000	0.089447000
6	1.305186000	6.159141000	1.331548000
6	1.439361000	6.076367000	-1.061796000
6	2.171526000	5.070768000	1.427019000
6	2.304790000	4.984873000	-0.988396000
6	2.671777000	4.479186000	0.263117000
8	3.531687000	3.416821000	0.397608000
1	-1.796459000	6.820269000	0.823316000
1	0.179366000	8.444236000	-0.893805000
1	0.082520000	8.506331000	0.859502000
1	0.927603000	6.615024000	2.244624000
1	1.164918000	6.464236000	-2.040807000
1	2.475476000	4.679733000	2.393836000
1	2.691345000	4.520637000	-1.891351000
1	3.758292000	3.046036000	-0.489970000
6	-3.408300000	1.910728000	-0.733207000
6	-4.776723000	1.716071000	-0.102984000
8	-4.938795000	1.122110000	0.962964000
6	-2.486854000	2.773760000	0.149657000
6	-1.077194000	2.910920000	-0.433150000
6	-0.251829000	1.629344000	-0.432337000
8	-0.551303000	0.730769000	0.408262000
8	0.716092000	1.576664000	-1.255206000
1	-2.964232000	0.914849000	-0.843766000
1	-2.435078000	2.324210000	1.144949000
1	-2.921495000	3.775305000	0.263740000
1	-1.094090000	3.300372000	-1.457047000
1	-0.502209000	3.642060000	0.151845000
7	-5.846203000	2.219777000	-0.799572000
6	-7.182858000	2.233711000	-0.218217000
6	-7.889220000	0.870672000	-0.126881000
8	-8.891027000	0.749113000	0.562576000
1	-5.666855000	2.787694000	-1.615454000
1	-7.149525000	2.619787000	0.804908000
7	-7.330846000	-0.135122000	-0.859232000
6	-7.746126000	-1.518417000	-0.740065000
6	-6.723654000	-2.441654000	-0.056186000
8	-6.722538000	-3.644282000	-0.316224000
1	-6.483678000	0.073534000	-1.368948000
1	-8.672902000	-1.522189000	-0.157743000
7	-5.880775000	-1.848844000	0.826553000
6	-4.882973000	-2.593739000	1.582711000
6	-3.445645000	-2.126204000	1.313991000
6	-2.955888000	-2.364114000	-0.084217000

7	-1.728701000	-1.859183000	-0.503539000
6	-3.517455000	-3.058574000	-1.127656000
6	-1.570235000	-2.241135000	-1.766627000
7	-2.628036000	-2.956634000	-2.182915000
1	-5.903307000	-0.840875000	0.947426000
1	-5.014589000	-3.646812000	1.324143000
1	-3.369331000	-1.054723000	1.543988000
1	-2.779952000	-2.646882000	2.017052000
1	-4.454885000	-3.590390000	-1.203427000
1	-0.694031000	-2.058245000	-2.370497000
1	-2.592687000	-3.472043000	-3.068892000
6	6.211792000	2.473153000	-3.295411000
6	5.969154000	1.107078000	-2.650836000
6	4.567882000	0.913138000	-2.106063000
8	3.823024000	1.904750000	-1.837006000
8	4.132366000	-0.273320000	-1.905677000
1	5.542313000	2.631177000	-4.147369000
1	7.243609000	2.547828000	-3.654676000
1	6.659270000	0.955296000	-1.808495000
1	6.166890000	0.288619000	-3.351809000
26	-0.009071000	-1.243825000	0.600789000
26	2.295605000	0.329453000	-1.103843000
8	0.021844000	-3.143438000	1.478904000
1	0.615815000	-3.034370000	2.283196000
1	0.500668000	-3.790742000	0.891083000
8	1.428576000	-2.755679000	-2.662681000
8	1.335385000	-1.359034000	-0.711253000
8	2.815890000	0.812243000	0.881924000
1	2.981564000	1.774994000	1.000278000
1	2.152755000	0.492766000	1.540894000
8	0.780909000	-3.989206000	-2.441651000
8	1.707929000	-4.652747000	-0.031111000
1	1.921679000	-5.563714000	0.229580000
1	2.554070000	-4.058679000	0.247709000
8	-1.358544000	-4.261259000	-4.279585000
1	-0.593087000	-4.333411000	-3.668591000
1	-1.015914000	-3.737302000	-5.019173000
1	6.037289000	3.282299000	-2.580662000
1	-2.195705000	8.256111000	-0.139703000
1	-1.696487000	6.754377000	-0.943232000
1	8.829168000	-1.719959000	-0.602663000
1	7.776974000	-0.942869000	0.599320000
1	4.546357000	-0.334566000	4.827843000
1	4.836807000	1.186549000	3.968534000
1	-5.090763000	-2.485439000	2.654591000
1	-7.960056000	-1.954950000	-1.720083000
1	-7.811631000	2.904328000	-0.810851000
1	-3.493463000	2.351182000	-1.735660000
1	4.848410000	-2.188606000	-0.735019000
1	1.397446000	-2.096427000	-1.570751000
1	1.232574000	-4.361792000	-1.632744000

[11]Pr'$_{A1}$:

6	-7.668869000	-1.721580000	0.333869000
6	-7.232164000	-3.118708000	-0.142815000
6	-5.832422000	-3.163559000	-0.671577000
7	-4.717654000	-2.910186000	0.125149000
6	-5.381750000	-3.500774000	-1.912135000
6	-3.524757000	-3.082750000	-0.628716000
7	-3.994791000	-3.469850000	-1.913580000
1	-7.026652000	-1.359068000	1.144215000
1	-7.333399000	-3.829221000	0.690318000
1	-7.909896000	-3.465087000	-0.931503000
1	-5.947141000	-3.836511000	-2.769501000
1	-2.825781000	-2.242027000	-0.600981000
1	-3.412018000	-3.334118000	-2.731990000
6	-4.033768000	0.149475000	-4.063561000
6	-2.631208000	0.672099000	-4.392738000
6	-1.579147000	-0.437978000	-4.565829000
6	-1.296120000	-1.274273000	-3.320754000
8	-0.851210000	-0.632879000	-2.282766000
8	-1.462321000	-2.512234000	-3.364148000
1	-4.058183000	-0.367004000	-3.097910000
1	-2.665663000	1.248803000	-5.325776000
1	-2.296788000	1.372485000	-3.617675000
1	-0.624331000	0.017597000	-4.860501000
1	-1.881460000	-1.124943000	-5.362227000
6	1.477528000	7.494649000	0.191742000
6	-0.003562000	7.902812000	0.090240000
6	-0.941607000	6.717469000	-0.009367000
6	-1.321501000	6.194221000	-1.253430000
6	-1.432182000	6.082188000	1.139819000
6	-2.158112000	5.082675000	-1.353250000
6	-2.266472000	4.967521000	1.062105000
6	-2.631022000	4.465289000	-0.191610000
8	-3.464858000	3.382496000	-0.328403000
1	1.786318000	6.918305000	-0.688363000
1	-0.267883000	8.508559000	0.966688000
1	-0.136967000	8.551482000	-0.784924000
1	-0.965437000	6.670536000	-2.164713000
1	-1.160236000	6.467448000	2.120583000
1	-2.460654000	4.694656000	-2.321790000
1	-2.631202000	4.482133000	1.962964000
1	-3.672188000	2.998469000	0.558046000
6	3.508635000	2.001310000	0.614254000
6	4.867836000	1.792507000	-0.031604000
8	5.012034000	1.182015000	-1.090921000
6	2.562711000	2.810764000	-0.292598000
6	1.153442000	2.939844000	0.292281000
6	0.353848000	1.642366000	0.347876000
8	0.706403000	0.697686000	-0.421412000
8	-0.638933000	1.621515000	1.138245000
1	3.077369000	1.006960000	0.777996000
1	2.516413000	2.322806000	-1.269843000
1	2.975381000	3.816225000	-0.446968000
1	1.165946000	3.369013000	1.300409000
1	0.559453000	3.636372000	-0.315457000
7	5.949259000	2.300256000	0.642798000
6	7.276501000	2.305312000	0.039493000
6	7.975618000	0.939181000	-0.058149000
8	8.961961000	0.810015000	-0.768125000
1	5.783168000	2.882784000	1.451216000
1	7.228883000	2.687603000	-0.984532000
7	7.430786000	-0.060257000	0.693020000
6	7.841132000	-1.444977000	0.573176000
6	6.807973000	-2.370473000	-0.091114000
8	6.820019000	-3.574260000	0.163558000
1	6.595509000	0.153632000	1.219947000
1	8.758734000	-1.453337000	-0.023382000
7	5.940488000	-1.778926000	-0.950524000
6	4.931791000	-2.527052000	-1.688385000
6	3.495544000	-2.087202000	-1.369636000
6	3.050802000	-2.357916000	0.037404000
7	1.832196000	-1.874611000	0.503886000
6	3.648987000	-3.065211000	1.051379000
6	1.714065000	-2.284406000	1.764413000
7	2.790348000	-2.994704000	2.134215000
1	5.957100000	-0.770761000	-1.071990000
1	5.086555000	-3.582150000	-1.451822000
1	3.395125000	-1.013364000	-1.578031000
1	2.818423000	-2.606835000	-2.062524000
1	4.595437000	-3.585037000	1.089928000
1	0.858255000	-2.119677000	2.398294000
1	2.787939000	-3.526443000	3.014713000
6	-6.021343000	2.383521000	3.490106000
6	-5.793062000	1.022411000	2.830328000
6	-4.422711000	0.851701000	2.205077000
8	-3.703678000	1.855370000	1.910006000
8	-3.986185000	-0.326465000	1.963973000
1	-5.307101000	2.552351000	4.302735000
1	-7.032059000	2.440302000	3.907908000
1	-6.528242000	0.858820000	2.029370000
1	-5.935896000	0.200196000	3.540326000
26	0.073928000	-1.262101000	-0.504946000
26	-2.194776000	0.319474000	1.089695000
8	0.085044000	-3.189004000	-1.349587000
1	-0.516910000	-3.106768000	-2.148188000
1	-0.368054000	-3.841395000	-0.746173000
8	-1.334597000	-3.022502000	2.924052000
8	-1.141980000	-1.344749000	0.780709000

8	-2.768272000	0.792171000	-0.889841000
1	-2.926275000	1.758674000	-0.990716000
1	-2.084037000	0.487686000	-1.529978000
8	-0.514014000	-4.175774000	2.594449000
8	-1.520385000	-4.781476000	0.171724000
1	-1.716792000	-5.678710000	-0.144165000
1	-2.379227000	-4.205568000	-0.048428000
8	1.714542000	-4.370464000	4.278527000
1	0.897445000	-4.458115000	3.737236000
1	1.424573000	-3.882880000	5.063968000
1	-5.901821000	3.196133000	2.767987000
1	2.126039000	8.375754000	0.265406000
1	1.650626000	6.870458000	1.076291000
1	-8.697757000	-1.745048000	0.709744000
1	-7.617460000	-0.996427000	-0.485790000
1	-4.381454000	-0.550876000	-4.832994000
1	-4.754697000	0.973149000	-4.011659000
1	5.105280000	-2.397376000	-2.763854000
1	8.069823000	-1.877462000	1.551661000
1	7.917574000	2.975315000	0.619532000
1	3.605766000	2.488059000	1.593857000
1	-4.722787000	-2.282013000	0.922832000
1	-1.252205000	-2.459535000	2.104580000
1	-0.983448000	-4.564196000	1.811531000

Model A2 structures:

^{11}Re$_{A2}$:

6	7.302671000	-1.344990000	6.065696000
6	5.942498000	-1.864184000	5.562775000
6	5.714399000	-1.642946000	4.099119000
7	6.508323000	-2.218032000	3.110875000
6	4.782380000	-0.897608000	3.425180000
6	6.078081000	-1.842443000	1.897062000
7	5.032267000	-1.039450000	2.071021000
1	8.142622000	-1.851972000	5.576385000
1	5.846501000	-2.934930000	5.786973000
1	5.129354000	-1.364856000	6.098782000
1	3.967041000	-0.289419000	3.803429000
1	6.503897000	-2.144202000	0.952648000
1	4.475345000	-0.599878000	1.298074000
6	4.902012000	0.783304000	-3.465615000
6	4.726288000	2.005100000	-2.556783000
6	4.583003000	1.652085000	-1.067923000
6	3.306870000	0.906295000	-0.689594000
8	2.204902000	1.271844000	-1.216218000
8	3.379406000	-0.023378000	0.173326000
1	4.026778000	0.123356000	-3.439841000
1	5.592828000	2.667557000	-2.666555000
1	3.852539000	2.590983000	-2.868499000
1	4.580812000	2.578913000	-0.475173000
1	5.443697000	1.064780000	-0.730021000
6	-6.824468000	6.356184000	-6.569343000
6	-7.029248000	4.883916000	-7.014637000
6	-5.722551000	4.177152000	-7.205668000
6	-5.033052000	4.253533000	-8.452000000
6	-5.122701000	3.450489000	-6.135311000
6	-3.821599000	3.638516000	-8.627874000
6	-3.912082000	2.819988000	-6.278110000
6	-3.228955000	2.903098000	-7.542285000
8	-2.083292000	2.338240000	-7.766948000
1	-6.257849000	6.921798000	-7.315592000
1	-7.627274000	4.361397000	-6.259945000
1	-7.595700000	4.872607000	-7.952949000
1	-5.486266000	4.804758000	-9.270877000
1	-5.644661000	3.391527000	-5.184372000
1	-3.281174000	3.678039000	-9.567837000
1	-3.476004000	2.263879000	-5.452347000
1	-1.663233000	1.790837000	-6.946626000
6	0.589900000	4.605809000	0.809154000
6	1.246473000	3.493566000	1.599163000
8	1.149364000	2.286306000	1.311954000
6	1.304582000	4.858117000	-0.554951000
6	0.356479000	4.903597000	-1.766652000
6	-0.179732000	3.527525000	-2.131512000
8	-0.432642000	2.712990000	-1.191521000
8	-0.358026000	3.243289000	-3.354300000
1	-0.443695000	4.298748000	0.638832000
1	2.048383000	4.074136000	-0.735106000
1	1.851189000	5.804890000	-0.500088000
1	-0.499132000	5.563143000	-1.564812000
1	0.867528000	5.317495000	-2.640520000
7	1.998312000	3.852965000	2.670804000
6	2.771950000	2.860709000	3.405797000
6	1.950530000	1.967022000	4.348289000
8	2.360006000	0.835581000	4.633522000
1	2.152900000	4.836244000	2.849467000
1	3.278874000	2.196232000	2.702610000
7	0.838713000	2.516286000	4.870863000
6	-0.024765000	1.833508000	5.833906000
6	-1.072985000	0.910475000	5.177937000
8	-2.267524000	1.163346000	5.235532000
1	0.525497000	3.402783000	4.499642000
1	0.615845000	1.261920000	6.512090000
7	-0.540091000	-0.186087000	4.568515000
6	-1.360266000	-1.241539000	3.994873000
6	-1.391872000	-1.265749000	2.452316000
6	-2.191731000	-0.171618000	1.800222000
7	-1.646631000	0.799191000	0.962922000
6	-3.553117000	0.019696000	1.849610000
6	-2.668168000	1.543099000	0.541763000
7	-3.830165000	1.111612000	1.059965000
1	0.467942000	-0.295807000	4.590249000
1	-2.367279000	-1.113264000	4.398418000
1	-0.372166000	-1.252008000	2.058176000
1	-1.817506000	-2.235970000	2.159896000
1	-4.325591000	-0.511782000	2.384115000
1	-2.597326000	2.353826000	-0.165402000
1	-4.734378000	1.412401000	0.672511000
6	-1.112588000	-2.524222000	-6.905390000
6	-1.158161000	-1.008429000	-7.094460000
6	-1.023967000	-0.208208000	-5.817672000
8	-1.093349000	1.085167000	-5.867692000
8	-0.841627000	-0.744069000	-4.696653000
1	-0.161922000	-2.837922000	-6.463173000
1	-1.217214000	-3.027295000	-7.870977000
1	-2.095894000	-0.700354000	-7.576382000
1	-0.360788000	-0.671224000	-7.770570000
26	0.191994000	1.020185000	-0.156089000
26	-0.797212000	1.262114000	-3.648017000
8	0.926085000	-0.984313000	0.069597000
1	1.904833000	-0.788389000	0.162932000
1	0.824434000	-1.506641000	-0.752062000
8	-2.912710000	1.427563000	-3.412231000
8	-0.990640000	0.042648000	-1.871558000
8	1.379132000	0.965004000	-3.774795000
1	1.762264000	1.603354000	-4.397396000
1	1.798624000	1.148159000	-2.896225000
8	-3.401195000	0.736784000	-2.383815000
8	0.103408000	-2.289183000	-2.558660000
1	-0.372084000	-3.135507000	-2.553267000
1	0.055537000	-1.967990000	-3.486237000
8	-5.622716000	1.798273000	-0.938072000
1	-4.988990000	1.412596000	-1.583815000
1	-6.465493000	1.349076000	-1.102304000
1	-1.922243000	-2.866948000	-6.253855000
1	-7.800298000	6.834492000	-6.444729000
1	-6.289429000	6.411015000	-5.616043000
1	7.394623000	-1.525692000	7.140301000
1	7.406399000	-0.269806000	5.889633000
1	5.777296000	0.193324000	-3.168764000
1	5.054343000	1.089576000	-4.506212000
1	-0.976968000	-2.205709000	4.350392000
1	-0.567779000	2.586902000	6.405019000
1	3.528216000	3.383832000	3.999042000
1	0.567006000	5.525844000	1.403300000
1	7.296508000	-2.832156000	3.276848000
1	-0.791155000	-0.910239000	-2.064074000
1	-2.006616000	0.160367000	-1.830706000

^{11}TS1$_{A2}$:

6	6.890258000	0.323798000	5.796291000
6	5.520899000	-0.343579000	5.565502000
6	5.307816000	-0.803702000	4.156751000
7	6.069559000	-1.803439000	3.557327000
6	4.423326000	-0.401671000	3.189530000
6	5.665251000	-2.000353000	2.293087000
7	4.666624000	-1.154874000	2.055355000
1	7.723785000	-0.360742000	5.599033000
1	5.405976000	-1.198809000	6.245047000
1	4.712139000	0.354200000	5.797906000
1	3.649958000	0.351263000	3.226690000
1	6.075861000	-2.718388000	1.600069000
1	4.131457000	-1.073478000	1.156361000
6	4.760932000	0.598542000	-3.489425000
6	4.468737000	1.640382000	-2.403370000
6	4.262064000	1.034578000	-1.005817000
6	3.034734000	0.151599000	-0.849709000
8	1.948393000	0.536049000	-1.427952000
8	3.091587000	-0.889167000	-0.142069000
1	3.921447000	-0.093296000	-3.630495000
1	5.304427000	2.347256000	-2.343041000
1	3.585781000	2.233873000	-2.671359000
1	4.134331000	1.838716000	-0.266895000
1	5.143684000	0.457725000	-0.706733000
6	-6.630942000	6.849361000	-6.729102000
6	-6.807035000	5.446413000	-7.367936000
6	-5.505257000	4.710943000	-7.458385000
6	-4.626075000	4.930485000	-8.559445000
6	-5.101034000	3.811338000	-6.427842000
6	-3.419796000	4.286081000	-8.640466000
6	-3.901077000	3.148160000	-6.479838000
6	-3.027035000	3.369791000	-7.602756000
8	-1.885768000	2.772766000	-7.735294000
1	-5.948334000	7.471239000	-7.316605000
1	-7.521522000	4.867215000	-6.772940000
1	-7.231899000	5.565730000	-8.371490000
1	-4.929812000	5.615328000	-9.345902000
1	-5.768527000	3.646904000	-5.586810000
1	-2.738629000	4.430755000	-9.472469000
1	-3.600385000	2.463212000	-5.692862000
1	-1.637500000	2.061329000	-6.955971000
6	0.520717000	3.859049000	0.835723000
6	1.424907000	3.241064000	1.891538000
8	1.977767000	2.150215000	1.736536000
6	1.250672000	4.080084000	-0.503418000
6	0.305281000	4.325297000	-1.695667000
6	-0.287746000	3.052593000	-2.277262000
8	-0.518188000	2.050810000	-1.527343000
8	-0.543272000	3.000593000	-3.520968000
1	-0.296317000	3.147341000	0.682902000
1	1.869155000	3.202055000	-0.711418000
1	1.926295000	4.938690000	-0.409530000
1	-0.528009000	4.981484000	-1.405479000
1	0.827056000	4.843592000	-2.505705000
7	1.574563000	3.949276000	3.056357000
6	2.485721000	3.475269000	4.087230000
6	1.919538000	2.318054000	4.926042000
8	2.616426000	1.336098000	5.209970000
1	1.256926000	4.908313000	3.085620000
1	3.414703000	3.116740000	3.638852000
7	0.651999000	2.470034000	5.365775000
6	-0.007027000	1.523833000	6.266376000
6	-0.662357000	0.329739000	5.544887000
8	-1.867885000	0.122413000	5.617165000
1	0.107160000	3.239060000	4.999376000
1	0.740934000	1.163716000	6.979697000
7	0.217307000	-0.444326000	4.852467000
6	-0.178610000	-1.613529000	4.083845000
6	-0.198252000	-1.375408000	2.563652000
6	-1.294151000	-0.470059000	2.076586000
7	-1.187445000	0.213648000	0.868069000
6	-2.529007000	-0.196293000	2.613252000
6	-2.331862000	0.876021000	0.695298000
7	-3.158771000	0.652042000	1.724429000
1	1.198094000	-0.184268000	4.894584000
1	-1.160502000	-1.925722000	4.447396000
1	0.767514000	-0.956070000	2.253598000
1	-0.281738000	-2.349380000	2.061427000
1	-2.966367000	-0.472156000	3.559628000
1	-2.580466000	1.501297000	-0.145829000
1	-4.122046000	0.977464000	1.708706000
6	-1.371717000	-2.208657000	-7.631764000
6	-1.419062000	-0.683642000	-7.543800000
6	-1.316191000	-0.131916000	-6.139033000
8	-1.323937000	1.157238000	-5.961015000
8	-1.222915000	-0.857055000	-5.124397000
1	-0.426598000	-2.597247000	-7.239890000
1	-1.460711000	-2.528032000	-8.674024000
1	-2.351113000	-0.295417000	-7.977451000
1	-0.612148000	-0.228220000	-8.133323000
26	0.053229000	0.070952000	-0.773501000
26	-1.071473000	0.998252000	-3.755856000
8	0.674290000	-1.997079000	-0.675035000
1	1.638362000	-1.882927000	-0.485141000
1	0.577814000	-2.422521000	-1.552918000
8	-3.157277000	1.260190000	-3.316287000
8	-1.360854000	-0.488875000	-2.248674000
8	1.116593000	0.649982000	-4.035896000
1	1.432540000	1.392129000	-4.577254000
1	1.597775000	0.713265000	-3.177421000
8	-3.579612000	0.414763000	-2.375020000
8	-0.376201000	-2.786471000	-3.278694000
1	-0.848154000	-3.625269000	-3.403469000
1	-0.422208000	-2.322834000	-4.142883000
8	-5.128517000	1.550286000	-0.073816000
1	-4.879838000	1.120424000	-0.913810000
1	-6.033028000	1.869778000	-0.204982000
1	-2.189893000	-2.663590000	-7.065466000
1	-7.601980000	7.351252000	-6.688088000
1	-6.238042000	6.774783000	-5.710183000
1	6.973710000	0.647521000	6.837677000
1	7.015107000	1.202065000	5.155029000
1	5.645455000	0.002025000	-3.237325000
1	4.956314000	1.081847000	-4.452635000
1	0.521565000	-2.428037000	4.301499000
1	-0.793090000	2.048224000	6.810211000
1	2.719683000	4.311279000	4.754174000
1	0.078987000	4.799304000	1.188883000
1	6.821408000	-2.312411000	4.006321000
1	-1.251629000	-1.420910000	-2.559835000
1	-2.491095000	-0.208794000	-2.173976000

^{11}IM1$_{A2}$:

6	6.864155000	0.344698000	5.801227000
6	5.490930000	-0.317373000	5.577890000
6	5.278309000	-0.798244000	4.176040000
7	6.032893000	-1.814536000	3.595706000
6	4.401492000	-0.403238000	3.199047000
6	5.631627000	-2.027639000	2.333100000
7	4.642169000	-1.176522000	2.078023000
1	7.693488000	-0.348270000	5.616180000
1	5.368970000	-1.160976000	6.270602000
1	4.686091000	0.389127000	5.797328000
1	3.634807000	0.357079000	3.221337000
1	6.037843000	-2.760678000	1.653333000
1	4.110044000	-1.104293000	1.176372000
6	4.721612000	0.452563000	-3.507829000
6	4.459681000	1.527819000	-2.447266000
6	4.252859000	0.962351000	-1.032823000
6	3.020206000	0.092347000	-0.850382000
8	1.933553000	0.470881000	-1.434571000
8	3.070010000	-0.932118000	-0.121087000
1	3.867531000	-0.226317000	-3.619783000
1	5.308815000	2.220241000	-2.411498000
1	3.585964000	2.130678000	-2.724497000
1	4.134198000	1.786859000	-0.315222000
1	5.130766000	0.386392000	-0.721425000
6	-6.742094000	6.720979000	-6.531493000

6	-6.809246000	5.391150000	-7.329224000
6	-5.477430000	4.708505000	-7.377056000
6	-4.530870000	5.037342000	-8.392180000
6	-5.112786000	3.750674000	-6.384042000
6	-3.298180000	4.440645000	-8.428782000
6	-3.887597000	3.133541000	-6.392994000
6	-2.948267000	3.465616000	-7.431329000
8	-1.776171000	2.916677000	-7.523745000
1	-6.035907000	7.422217000	-6.986831000
1	-7.549319000	4.730825000	-6.863854000
1	-7.150657000	5.606214000	-8.348208000
1	-4.803954000	5.767138000	-9.148773000
1	-5.831455000	3.504331000	-5.607677000
1	-2.565347000	4.667034000	-9.196153000
1	-3.615327000	2.407127000	-5.633110000
1	-1.569662000	2.172536000	-6.795943000
6	0.516093000	3.815056000	0.756458000
6	1.423630000	3.221390000	1.824236000
8	1.990890000	2.136343000	1.685477000
6	1.247238000	4.023532000	-0.583172000
6	0.304107000	4.302855000	-1.770238000
6	-0.313940000	3.056091000	-2.376201000
8	-0.561018000	2.031766000	-1.657171000
8	-0.579265000	3.019658000	-3.616420000
1	-0.295671000	3.095080000	0.613534000
1	1.844001000	3.131870000	-0.796799000
1	1.943869000	4.864988000	-0.487175000
1	-0.516521000	4.969524000	-1.468179000
1	0.833278000	4.825354000	-2.573003000
7	1.559338000	3.947761000	2.979487000
6	2.467823000	3.498163000	4.023861000
6	1.901016000	2.356629000	4.884020000
8	2.591124000	1.371443000	5.172578000
1	1.226634000	4.901908000	2.995560000
1	3.398578000	3.132455000	3.585278000
7	0.639507000	2.526165000	5.335464000
6	-0.021159000	1.591802000	6.247140000
6	-0.702252000	0.407611000	5.533448000
8	-1.912360000	0.227692000	5.606280000
1	0.096645000	3.294398000	4.964606000
1	0.731251000	1.222623000	6.950846000
7	0.160292000	-0.388235000	4.844722000
6	-0.260083000	-1.549352000	4.075867000
6	-0.254382000	-1.317695000	2.554812000
6	-1.318120000	-0.384847000	2.048751000
7	-1.191878000	0.257287000	0.820328000
6	-2.536467000	-0.045197000	2.586733000
6	-2.309082000	0.959107000	0.635605000
7	-3.137955000	0.801441000	1.677032000
1	1.145721000	-0.145786000	4.881191000
1	-1.254857000	-1.830818000	4.429275000
1	0.726669000	-0.927239000	2.254591000
1	-0.358490000	-2.291119000	2.055533000
1	-2.976668000	-0.276300000	3.543987000
1	-2.533166000	1.572451000	-0.220966000
1	-4.082441000	1.176891000	1.664311000
6	-1.100130000	-2.097957000	-7.704829000
6	-1.208093000	-0.584037000	-7.525029000
6	-1.224525000	-0.125325000	-6.084993000
8	-1.294913000	1.151380000	-5.826494000
8	-1.173110000	-0.906185000	-5.112513000
1	-0.172236000	-2.481256000	-7.269354000
1	-1.103285000	-2.351300000	-8.768781000
1	-2.119993000	-0.196044000	-7.999780000
1	-0.377043000	-0.065733000	-8.022087000
26	0.043167000	-0.009667000	-0.823334000
26	-1.175142000	0.956413000	-3.674642000
8	0.651108000	-2.081503000	-0.657116000
1	1.611414000	-1.966810000	-0.458067000
1	0.564723000	-2.490675000	-1.545304000
8	-3.085100000	1.275880000	-3.442787000
8	-1.322268000	-0.475332000	-2.269646000
8	1.027195000	0.740817000	-4.016110000
1	1.257578000	1.599370000	-4.410264000
1	1.528335000	0.690993000	-3.167159000

8	-3.727351000	0.494676000	-2.496949000
8	-0.277708000	-2.843811000	-3.274756000
1	-0.751902000	-3.678116000	-3.417686000
1	-0.324310000	-2.363347000	-4.127625000
8	-5.078853000	1.833306000	-0.104844000
1	-4.866940000	1.387448000	-0.945028000
1	-6.000619000	2.117709000	-0.190522000
1	-1.939414000	-2.613959000	-7.229169000
1	-7.731813000	7.186626000	-6.524726000
1	-6.435507000	6.547104000	-5.495230000
1	6.947752000	0.683969000	6.837656000
1	6.995969000	1.212085000	5.146682000
1	5.598289000	-0.152664000	-3.249036000
1	4.912836000	0.905832000	-4.486121000
1	0.413497000	-2.382709000	4.305763000
1	-0.792639000	2.128385000	6.799942000
1	2.697709000	4.348927000	4.673326000
1	0.066353000	4.757232000	1.094431000
1	6.777871000	-2.323594000	4.055868000
1	-1.217621000	-1.395287000	-2.596705000
1	-2.965204000	-0.092413000	-2.191255000

^{11}TS4$_{A2}$:

6	7.837880000	-0.653112000	4.813929000
6	6.341135000	-0.935187000	5.044773000
6	5.645969000	-1.519309000	3.853731000
7	5.973673000	-2.759599000	3.313879000
6	4.633184000	-1.040203000	3.064555000
6	5.197034000	-3.020359000	2.251032000
7	4.377437000	-1.986543000	2.086917000
1	8.398146000	-1.568143000	4.589277000
1	6.216779000	-1.607871000	5.903827000
1	5.820634000	-0.007714000	5.303308000
1	4.079371000	-0.111641000	3.127624000
1	5.233685000	-3.910947000	1.642530000
1	3.648649000	-1.894983000	1.328690000
6	4.422234000	-1.244472000	-2.639448000
6	3.987007000	0.158657000	-2.204746000
6	3.551406000	0.250503000	-0.722103000
6	2.329848000	-0.603290000	-0.430212000
8	1.225012000	-0.211208000	-0.949499000
8	2.411275000	-1.660889000	0.257029000
1	3.586984000	-1.953915000	-2.633230000
1	4.813777000	0.864734000	-2.345289000
1	3.165340000	0.507671000	-2.840841000
1	3.301549000	1.291978000	-0.485644000
1	4.379126000	-0.056841000	-0.074847000
6	-7.138936000	5.642209000	-7.077291000
6	-6.656080000	4.384366000	-7.847493000
6	-5.248301000	4.021923000	-7.488383000
6	-4.149239000	4.641802000	-8.155376000
6	-4.977590000	3.084874000	-6.447947000
6	-2.857134000	4.336304000	-7.819556000
6	-3.695474000	2.754288000	-6.086348000
6	-2.600686000	3.376055000	-6.781204000
8	-1.356119000	3.103996000	-6.527157000
1	-6.515213000	6.512157000	-7.305299000
1	-7.325225000	3.546777000	-7.622349000
1	-6.722392000	4.581701000	-8.923726000
1	-4.350373000	5.357604000	-8.947099000
1	-5.814721000	2.618839000	-5.936178000
1	-2.004887000	4.785032000	-8.318976000
1	-3.498858000	2.043316000	-5.287055000
1	-1.199651000	2.378276000	-5.795360000
6	-0.685809000	5.234947000	0.628616000
6	0.482007000	4.283803000	0.845484000
8	0.869298000	3.472644000	0.001564000
6	-0.793857000	5.818433000	-0.804697000
6	-2.043907000	5.378217000	-1.613300000
6	-1.921345000	3.964098000	-2.122917000
8	-1.997891000	2.973170000	-1.310437000
8	-1.660610000	3.712937000	-3.338406000
1	-1.590791000	4.664806000	0.879832000
1	0.106985000	5.580072000	-1.380291000
1	-0.831533000	6.909365000	-0.732502000

1	-2.934825000	5.453222000	-0.979012000
1	-2.172061000	6.041821000	-2.472049000
7	1.069358000	4.330188000	2.084580000
6	2.189252000	3.465406000	2.429931000
6	1.936527000	2.441527000	3.544842000
8	2.711471000	1.488185000	3.693167000
1	0.902240000	5.153460000	2.648562000
1	2.472546000	2.918510000	1.530700000
7	0.902626000	2.684694000	4.376486000
6	0.648251000	1.896856000	5.581893000
6	-0.207799000	0.642735000	5.329303000
8	-1.347959000	0.556002000	5.772266000
1	0.226887000	3.387688000	4.110019000
1	1.616880000	1.619461000	6.008085000
7	0.407090000	-0.317751000	4.586338000
6	-0.226408000	-1.585842000	4.249203000
6	-0.556299000	-1.750217000	2.755564000
6	-1.571102000	-0.780119000	2.224053000
7	-1.775073000	-0.622818000	0.854931000
6	-2.432133000	0.059002000	2.887758000
6	-2.738903000	0.286553000	0.714450000
7	-3.156684000	0.716389000	1.914702000
1	1.346875000	-0.119262000	4.257988000
1	-1.130925000	-1.655340000	4.857517000
1	0.367812000	-1.663464000	2.165671000
1	-0.913501000	-2.779562000	2.606005000
1	-2.565392000	0.246516000	3.943168000
1	-3.128510000	0.654018000	-0.221736000
1	-3.746719000	1.542376000	2.005730000
6	1.027377000	-1.141815000	-6.918323000
6	0.142973000	0.061063000	-6.594857000
6	-0.057512000	0.298385000	-5.106803000
8	-0.869474000	1.303639000	-4.802024000
8	0.485411000	-0.375782000	-4.234638000
1	2.018326000	-1.036311000	-6.467316000
1	1.152617000	-1.240643000	-8.000444000
1	-0.849757000	-0.044347000	-7.052143000
1	0.567892000	0.981192000	-7.018932000
26	-0.653289000	-1.091294000	-0.842733000
26	-1.321743000	1.649127000	-2.871800000
8	0.227660000	-2.932804000	-0.941594000
1	1.055878000	-2.795478000	-0.420021000
1	0.539405000	-3.067723000	-1.892160000
8	-3.421240000	0.960522000	-3.274338000
8	-1.667848000	-0.036246000	-2.123821000
8	0.577926000	2.015717000	-2.223493000
1	0.609535000	2.662784000	-1.471632000
1	0.972192000	1.174564000	-1.858805000
8	-4.346546000	1.538373000	-2.301457000
8	1.177661000	-2.925250000	-3.398438000
1	0.946397000	-3.594044000	-4.060573000
1	0.996839000	-2.049047000	-3.810503000
8	-3.809946000	3.271773000	0.883351000
1	-3.262837000	3.091547000	0.089309000
1	-4.655329000	3.599295000	0.540152000
1	0.581650000	-2.070702000	-6.547100000
1	-8.167477000	5.871377000	-7.370551000
1	-7.117574000	5.476490000	-5.995530000
1	8.276327000	-0.215412000	5.715097000
1	7.985954000	0.047221000	3.986118000
1	5.212961000	-1.636817000	-1.987606000
1	4.821342000	-1.220967000	-3.658828000
1	0.434913000	-2.405895000	4.552807000
1	0.107475000	2.518370000	6.296254000
1	3.053468000	4.066706000	2.738194000
1	-0.634045000	6.049064000	1.360632000
1	6.692499000	-3.380219000	3.666443000
1	-2.740827000	0.148545000	-2.612245000
1	-5.146251000	1.021405000	-2.508071000

^{11}Pr'$_{A2}$:

6	7.776839000	-0.476344000	4.903987000
6	6.277125000	-0.774892000	5.090790000
6	5.630494000	-1.394562000	3.890534000
7	5.986065000	-2.645680000	3.395068000
6	4.644886000	-0.943198000	3.052100000
6	5.251183000	-2.939127000	2.311241000
7	4.431719000	-1.915919000	2.090242000
1	8.357154000	-1.387943000	4.720072000
1	6.132962000	-1.430609000	5.959778000
1	5.735734000	0.150522000	5.311151000
1	4.085599000	-0.016089000	3.070697000
1	5.315989000	-3.844730000	1.727729000
1	3.729429000	-1.848253000	1.304585000
6	4.486469000	-1.269541000	-2.737737000
6	4.111405000	0.145834000	-2.287034000
6	3.706690000	0.244071000	-0.795979000
6	2.460787000	-0.568231000	-0.485927000
8	1.360594000	-0.141018000	-0.986892000
8	2.520780000	-1.627283000	0.201303000
1	3.626371000	-1.948493000	-2.720734000
1	4.961119000	0.821794000	-2.438375000
1	3.292350000	0.529479000	-2.906356000
1	3.503240000	1.292962000	-0.547536000
1	4.534585000	-0.098146000	-0.166760000
6	-6.820654000	5.984011000	-6.970394000
6	-6.394509000	4.751635000	-7.811688000
6	-5.010586000	4.296785000	-7.464312000
6	-3.875226000	4.876692000	-8.105062000
6	-4.798691000	3.307940000	-6.458348000
6	-2.603542000	4.487030000	-7.777162000
6	-3.538578000	2.894969000	-6.106113000
6	-2.405514000	3.477691000	-6.772934000
8	-1.181922000	3.124432000	-6.524055000
1	-6.151302000	6.832873000	-7.141514000
1	-7.107293000	3.937409000	-7.640664000
1	-6.440554000	5.014959000	-8.874589000
1	-4.031901000	5.630713000	-8.870975000
1	-5.662321000	2.869383000	-5.966769000
1	-1.725241000	4.904924000	-8.257849000
1	-3.384616000	2.144305000	-5.337308000
1	-1.072886000	2.369342000	-5.805228000
6	-0.757421000	5.331158000	0.528301000
6	0.436966000	4.410071000	0.731275000
8	0.878474000	3.660859000	-0.141671000
6	-0.909467000	5.894881000	-0.907485000
6	-2.117092000	5.354008000	-1.719626000
6	-1.902546000	3.940517000	-2.202160000
8	-1.964622000	2.958954000	-1.376295000
8	-1.584735000	3.685570000	-3.402365000
1	-1.645181000	4.747271000	0.809390000
1	0.010357000	5.728607000	-1.478149000
1	-1.037983000	6.979229000	-0.839823000
1	-3.017630000	5.383663000	-1.095363000
1	-2.277923000	5.993152000	-2.591449000
7	0.985592000	4.411552000	1.989330000
6	2.125924000	3.568699000	2.323132000
6	1.909861000	2.544071000	3.444539000
8	2.718058000	1.619227000	3.595415000
1	0.780942000	5.204849000	2.583399000
1	2.405423000	3.026496000	1.419868000
7	0.871227000	2.756121000	4.279324000
6	0.655295000	1.972807000	5.495217000
6	-0.153968000	0.683757000	5.265449000
8	-1.285048000	0.557965000	5.722054000
1	0.166214000	3.428206000	4.009139000
1	1.637003000	1.737465000	5.916758000
7	0.489996000	-0.260320000	4.526136000
6	-0.100394000	-1.552673000	4.202836000
6	-0.429226000	-1.741312000	2.711800000
6	-1.467101000	-0.799887000	2.173723000
7	-1.670706000	-0.650732000	0.803931000
6	-2.350905000	0.018471000	2.833418000
6	-2.657102000	0.233380000	0.659169000
7	-3.089238000	0.655919000	1.857057000
1	1.416177000	-0.027184000	4.182489000
1	-0.999920000	-1.646949000	4.815205000
1	0.490403000	-1.639652000	2.117589000
1	-0.762516000	-2.780165000	2.573771000
1	-2.492831000	0.204285000	3.887926000

1	-3.047650000	0.589728000	-0.280585000
1	-3.691878000	1.472755000	1.945859000
6	0.808883000	-1.329332000	-6.983666000
6	-0.002393000	-0.079712000	-6.645857000
6	-0.075958000	0.228048000	-5.159055000
8	-0.817164000	1.280021000	-4.834868000
8	0.505325000	-0.434197000	-4.301617000
1	1.837674000	-1.242750000	-6.622027000
1	0.839025000	-1.481199000	-8.066478000
1	-1.031768000	-0.165074000	-7.018737000
1	0.420386000	0.803759000	-7.143685000
26	-0.540397000	-1.023710000	-0.915403000
26	-1.104234000	1.650175000	-2.861797000
8	0.315006000	-2.883132000	-0.973682000
1	1.152348000	-2.744338000	-0.468219000
1	0.606490000	-3.055063000	-1.923977000
8	-3.750264000	0.821629000	-3.350644000
8	-1.536022000	0.069922000	-2.152395000
8	0.782754000	2.076126000	-2.281622000
1	0.794544000	2.772553000	-1.574625000
1	1.165910000	1.253211000	-1.858296000
8	-4.576064000	1.340300000	-2.271843000
8	1.221089000	-2.966068000	-3.447150000
1	0.991768000	-3.658854000	-4.084757000
1	1.023662000	-2.104866000	-3.882830000
8	-3.758550000	3.215398000	0.830916000
1	-3.191306000	3.052462000	0.046501000
1	-4.605888000	3.518481000	0.470475000
1	0.362816000	-2.222385000	-6.533209000
1	-7.833321000	6.282964000	-7.256058000
1	-6.818510000	5.753002000	-5.900400000
1	8.177958000	-0.011440000	5.808920000
1	7.943137000	0.206862000	4.065425000
1	5.273694000	-1.695082000	-2.102827000
1	4.867644000	-1.253467000	-3.764083000
1	0.589187000	-2.347125000	4.511871000
1	0.096800000	2.581682000	6.206858000
1	2.986025000	4.183561000	2.616997000
1	-0.703174000	6.155493000	1.249019000
1	6.695302000	-3.251882000	3.789710000
1	-3.102820000	0.253661000	-2.845818000
1	-5.374896000	0.792404000	-2.384757000

Model B structure:

$^{11}Re_B$:

6	8.697444000	-0.847891000	3.689884000
6	8.071036000	0.203743000	2.783568000
8	8.131042000	0.046918000	1.526046000
6	7.811131000	-2.128389000	3.856676000
6	7.002560000	-2.484248000	2.648655000
7	7.518089000	-2.813214000	1.387970000
6	5.644472000	-2.430839000	2.476616000
6	6.516348000	-2.915471000	0.481860000
7	5.371111000	-2.691512000	1.139800000
1	9.651635000	-1.121677000	3.227380000
1	8.458122000	-2.958946000	4.165051000
1	7.104428000	-1.963096000	4.676034000
1	4.865953000	-2.210170000	3.182711000
1	6.606382000	-3.091362000	-0.591420000
1	4.398211000	-2.544436000	0.725550000
7	7.483199000	1.273113000	3.368977000
6	6.701397000	2.276630000	2.633913000
6	7.345853000	2.863780000	1.403826000
8	6.606928000	3.295577000	0.471802000
1	7.290385000	1.276297000	4.375038000
1	5.732367000	1.880273000	2.313210000
7	8.691914000	2.930864000	1.297338000
6	9.320517000	3.395317000	0.059058000
6	8.822338000	2.680496000	-1.207986000
8	8.823664000	3.291554000	-2.300459000
1	9.263139000	2.596266000	2.058683000
1	9.133409000	4.459832000	-0.103088000
7	8.415614000	1.381602000	-1.084886000
6	7.908077000	0.665445000	-2.250229000
6	6.470654000	1.008042000	-2.664441000
8	6.036112000	0.625936000	-3.784253000
1	8.394834000	0.923899000	-0.172757000
1	8.537724000	0.878490000	-3.118012000
7	5.713499000	1.676230000	-1.759927000
6	4.319548000	2.039999000	-2.015569000
6	4.109021000	3.557021000	-2.090312000
8	3.061680000	4.009227000	-2.645768000
6	3.358283000	1.402315000	-0.976046000
6	3.499536000	-0.127982000	-0.937517000
6	2.647506000	-0.799073000	0.124115000
8	1.643328000	-0.172522000	0.598963000
8	2.960867000	-2.017023000	0.494872000
1	6.124699000	1.948401000	-0.870324000
1	4.081004000	1.654006000	-3.011351000
1	2.331514000	1.670284000	-1.242745000
1	3.549311000	1.827074000	0.017108000
1	4.544518000	-0.421121000	-0.783700000
1	3.196415000	-0.573292000	-1.897794000
7	5.049711000	4.374155000	-1.563685000
6	4.855507000	5.822424000	-1.486372000
6	4.710057000	6.520362000	-2.838666000
8	4.014367000	7.559508000	-2.943233000
1	5.800667000	3.987387000	-0.995610000
1	3.959242000	6.068438000	-0.911901000
7	5.373327000	5.961443000	-3.888023000
6	5.257274000	6.511748000	-5.237916000
6	3.858843000	6.376399000	-5.863314000
8	3.533626000	7.129254000	-6.805774000
1	5.961616000	5.156118000	-3.719406000
1	5.481249000	7.581507000	-5.234870000
7	3.052600000	5.403392000	-5.348337000
6	1.629981000	5.306706000	-5.694967000
6	0.755493000	6.145861000	-4.742519000
8	-0.206914000	6.815307000	-5.191996000
6	1.172641000	3.838389000	-5.714855000
1	3.376114000	4.848096000	-4.564567000
1	1.503322000	5.764751000	-6.677828000
1	0.096944000	3.769167000	-5.894880000
1	1.700935000	3.303829000	-6.512777000
1	1.388135000	3.350794000	-4.757441000
7	1.084127000	6.113488000	-3.422966000
6	0.316158000	6.791624000	-2.370046000
6	-0.052695000	8.236835000	-2.725752000
8	-1.188682000	8.697357000	-2.447885000
6	1.169635000	6.755586000	-1.088771000
8	0.328425000	7.162797000	0.013518000
1	1.825824000	5.487812000	-3.119456000
1	-0.633080000	6.276990000	-2.187360000
1	1.533689000	5.731031000	-0.938607000
1	2.031996000	7.423734000	-1.204201000
1	0.800643000	7.219101000	0.876418000
7	0.923439000	8.985268000	-3.306265000
6	0.699519000	10.383578000	-3.668014000
6	-0.391336000	10.652339000	-4.716888000
8	-0.804135000	11.828916000	-4.854168000
1	1.830430000	8.566511000	-3.483221000
1	0.406241000	10.963650000	-2.787524000
7	-0.837063000	9.594595000	-5.442384000
6	-1.941698000	9.709988000	-6.384217000
6	-3.198015000	8.925995000	-5.991063000
8	-4.064957000	8.670623000	-6.865773000
1	-0.456372000	8.660249000	-5.299660000
1	-2.200166000	10.771223000	-6.461965000
7	-3.321172000	8.569381000	-4.685512000
6	-4.507587000	7.873452000	-4.180615000
6	-4.175902000	6.775043000	-3.158056000
6	-3.595562000	5.468579000	-3.688167000
6	-3.255877000	5.248715000	-5.033268000
6	-3.403864000	4.403102000	-2.786659000
6	-2.736044000	4.018939000	-5.454654000
6	-2.883970000	3.169989000	-3.191524000
6	-2.537179000	2.980233000	-4.537347000
8	-1.980941000	1.798831000	-5.010488000
1	-2.575294000	8.777181000	-4.026984000
1	-5.042923000	7.475409000	-5.047309000
1	-5.106432000	6.543833000	-2.618520000
1	-3.487186000	7.199704000	-2.411964000
1	-3.388467000	6.035295000	-5.766791000
1	-3.684787000	4.535177000	-1.743670000
1	-2.484755000	3.853567000	-6.496752000
1	-2.757634000	2.363020000	-2.479371000
1	-1.896200000	1.066144000	-4.347034000
6	-4.734948000	6.218708000	2.844216000
6	-3.275634000	6.586519000	2.739131000
8	-2.359902000	5.731731000	2.920512000
6	-5.172010000	5.207078000	1.733860000
6	-5.923583000	3.990338000	2.307309000
6	-4.957300000	3.064103000	3.049494000
8	-4.025919000	2.472997000	2.433957000
8	-5.169214000	3.018636000	4.350297000
1	-4.869109000	5.743727000	3.824449000
1	-5.819965000	5.712065000	1.010353000
1	-4.295343000	4.843486000	1.187769000
1	-6.369437000	3.402203000	1.501504000
1	-6.739419000	4.292282000	2.967295000
7	-2.945425000	7.884401000	2.469010000
6	-1.565543000	8.250347000	2.110758000
6	-0.537929000	7.809535000	3.150143000
8	0.594890000	7.374160000	2.795695000
1	-3.682145000	8.534384000	2.232937000
1	-1.274582000	7.807389000	1.152235000
7	-0.858831000	7.953064000	4.462899000
6	0.068065000	7.563903000	5.527995000
6	0.199976000	6.046282000	5.746127000
8	1.277183000	5.578528000	6.195017000
1	-1.769769000	8.321653000	4.698638000
1	1.069294000	7.933870000	5.299526000
7	-0.893457000	5.301350000	5.463017000
6	-0.913436000	3.822609000	5.539348000
6	0.097639000	3.272989000	4.509923000
8	1.034684000	2.504793000	4.851293000
6	-2.365442000	3.369391000	5.282278000
6	-2.773966000	2.016805000	5.842283000
8	-2.121166000	1.435404000	6.761926000
8	-3.876219000	1.500048000	5.347185000
1	-1.630113000	5.722454000	4.907104000
1	-0.569768000	3.497050000	6.522630000

1	-2.587124000	3.360560000	4.213749000
1	-3.047967000	4.099389000	5.737044000
7	-0.047503000	3.731532000	3.240458000
6	0.860028000	3.316537000	2.164425000
6	2.292211000	3.812697000	2.416151000
8	3.289572000	3.098312000	2.127631000
6	0.347710000	3.820589000	0.794877000
6	-1.005971000	3.236542000	0.311641000
6	-0.989406000	1.729242000	0.202937000
8	-0.866929000	1.075183000	1.304984000
8	-1.081912000	1.164728000	-0.956283000
1	-0.847976000	4.311867000	3.003190000
1	0.919894000	2.228454000	2.151460000
1	1.119042000	3.582816000	0.052711000
1	0.242775000	4.911704000	0.820985000
1	-1.807240000	3.505723000	1.010090000
1	-1.242728000	3.666642000	-0.664421000
7	2.431572000	5.064436000	2.934634000
6	3.747535000	5.618613000	3.227722000
6	4.533278000	4.833056000	4.279481000
8	5.780505000	4.700668000	4.153151000
1	1.623079000	5.661344000	3.076481000
1	4.383344000	5.622649000	2.337721000
7	3.849740000	4.310330000	5.326989000
6	4.536420000	3.446334000	6.287480000
6	4.758989000	2.018117000	5.763660000
8	5.917448000	1.498287000	5.761817000
1	2.867494000	4.534414000	5.475997000
1	5.523462000	3.852861000	6.512896000
7	3.666060000	1.353457000	5.330553000
6	3.784202000	0.005892000	4.761658000
6	2.669179000	-0.300013000	3.755480000
6	1.342166000	-0.626848000	4.367628000
7	0.205637000	-0.813544000	3.572884000
6	0.963371000	-0.770298000	5.686741000
6	-0.824040000	-1.042413000	4.402380000
7	-0.398041000	-1.035370000	5.681825000
1	2.758590000	1.821330000	5.260506000
1	3.800921000	-0.743087000	5.567714000
1	2.981168000	-1.140903000	3.120902000
1	2.565686000	0.556332000	3.078957000
1	1.516044000	-0.642359000	6.600754000
1	-1.849552000	-1.185306000	4.111827000
6	1.042990000	-0.157369000	-5.595883000
6	2.193375000	-1.051515000	-5.978860000
8	2.345905000	-2.218535000	-5.492632000
6	-0.290619000	-0.566445000	-6.298943000
6	-0.976134000	-1.819739000	-5.731067000
6	-1.476947000	-1.732727000	-4.288564000
8	-1.397067000	-0.570153000	-3.668210000
8	-1.925793000	-2.780952000	-3.735191000
1	0.887340000	-0.196793000	-4.513986000
1	-0.962203000	0.292255000	-6.215702000
1	-0.098659000	-0.724160000	-7.369704000
1	-0.317655000	-2.695552000	-5.786348000
1	-1.847666000	-2.061676000	-6.354928000
7	3.063525000	-0.593435000	-6.919897000
6	4.024593000	-1.458085000	-7.615472000
6	5.065451000	-2.178642000	-6.757012000
8	5.507786000	-3.280609000	-7.152980000
1	2.909948000	0.333485000	-7.290993000
1	3.500523000	-2.252269000	-8.155457000
7	5.492930000	-1.574192000	-5.611939000
6	6.454842000	-2.223769000	-4.732953000
6	5.828143000	-2.813113000	-3.471078000
8	6.536527000	-2.971096000	-2.429467000
1	5.200682000	-0.642107000	-5.341616000
1	6.923772000	-3.038967000	-5.295172000
7	4.539218000	-3.193803000	-3.556218000
6	3.833749000	-3.891462000	-2.474421000
6	2.386323000	-4.208518000	-2.869841000
8	1.650555000	-2.956412000	-3.052634000
6	1.672498000	-5.034382000	-1.804298000
1	4.016930000	-2.958177000	-4.397294000
1	3.810312000	-3.270322000	-1.572537000
1	2.377944000	-4.750849000	-3.825839000
1	1.741331000	-2.629016000	-3.991016000
1	0.634068000	-5.203522000	-2.101657000
1	2.161185000	-6.006091000	-1.672673000
1	1.673898000	-4.510730000	-0.842993000
6	-5.121552000	-4.093565000	2.195495000
6	-6.351527000	-3.661251000	2.989503000
8	-7.446000000	-3.416806000	2.409458000
6	-5.533233000	-4.880590000	0.933008000
6	-4.367866000	-5.488095000	0.106053000
6	-3.409239000	-4.458522000	-0.459830000
8	-2.628282000	-3.841066000	0.351210000
7	-3.406261000	-4.227445000	-1.772336000
1	-4.559680000	-3.196303000	1.907283000
1	-6.142076000	-4.230367000	0.296728000
1	-6.187966000	-5.709480000	1.228516000
1	-4.793063000	-6.096456000	-0.700542000
1	-3.779431000	-6.160525000	0.743168000
1	-2.804801000	-3.532525000	-2.238651000
1	-4.060264000	-4.703855000	-2.375679000
7	-6.215714000	-3.549303000	4.340165000
6	-7.364756000	-3.250207000	5.196201000
6	-8.008159000	-1.870038000	5.026612000
8	-9.193482000	-1.700800000	5.395291000
1	-5.294664000	-3.556273000	4.775646000
1	-8.165644000	-3.970848000	5.010620000
7	-7.229981000	-0.892138000	4.488761000
6	-7.763320000	0.427233000	4.183618000
6	-8.012253000	0.670421000	2.692783000
8	-8.169819000	1.850698000	2.280263000
1	-6.238091000	-1.046398000	4.294609000
1	-8.716038000	0.537319000	4.712337000
7	-8.077846000	-0.427298000	1.897799000
6	-8.333265000	-0.354255000	0.461497000
6	-7.166504000	-0.866984000	-0.377408000
8	-6.018445000	0.022388000	-0.254898000
1	-7.966709000	-1.352572000	2.305895000
1	-8.541895000	0.687705000	0.212479000
1	-6.886553000	-1.877728000	-0.052841000
1	-7.472157000	-0.912108000	-1.429964000
1	-5.479339000	0.068158000	-1.105201000
6	-9.863384000	6.772679000	-2.490570000
6	-9.025972000	5.592618000	-2.044599000
6	-9.308761000	4.914926000	-0.846779000
6	-7.956027000	5.133139000	-2.834914000
6	-8.553755000	3.803785000	-0.447238000
6	-7.196027000	4.025174000	-2.442960000
6	-7.493328000	3.356275000	-1.247344000
1	-10.592857000	6.477750000	-3.257424000
1	-10.424960000	7.203029000	-1.654395000
1	-10.130311000	5.258542000	-0.222633000
1	-7.720259000	5.644264000	-3.765039000
1	-8.777759000	3.288016000	0.482085000
1	-6.371494000	3.689383000	-3.063958000
1	-6.911383000	2.491004000	-0.942064000
26	-0.138926000	-0.826391000	1.531099000
26	-1.624090000	-0.701769000	-1.611630000
8	0.509867000	-2.823565000	1.156301000
1	1.501493000	-2.795743000	1.081774000
1	0.044236000	-3.548773000	1.739794000
8	-4.373887000	-0.728525000	3.935188000
1	-4.231438000	0.106397000	4.462877000
1	-4.284166000	-0.540365000	2.947062000
8	-4.057056000	0.011774000	1.434411000
1	-4.880917000	-0.044675000	0.826653000
1	-3.893157000	0.964313000	1.686716000
8	-1.989354000	-1.317170000	0.662053000
1	-2.245692000	-2.275376000	0.502064000
1	-2.807069000	-0.744877000	0.936838000
8	-1.402635000	-2.786724000	8.938596000
1	-0.955768000	-3.374578000	8.245168000
1	-1.610360000	-3.251691000	9.762767000
8	-3.634512000	-1.155422000	-1.560384000
8	-4.164016000	0.014735000	-2.108642000
8	-0.115401000	-2.063630000	-1.321290000

1	0.510757000	-2.388204000	-2.034018000
8	-0.965661000	-4.402418000	2.393645000
1	-1.720002000	-4.382319000	1.753709000
1	-1.259437000	-4.384354000	3.345574000
8	-2.534654000	-1.213796000	7.328751000
1	-2.210663000	-1.691910000	8.163938000
1	-2.598173000	-0.220654000	7.343541000
8	-1.971969000	-4.455233000	4.911006000
1	-2.370535000	-5.338902000	5.017398000
1	-2.690382000	-3.679289000	5.091324000
8	-0.332177000	-4.084854000	6.970998000
1	-0.911311000	-4.249013000	6.163589000
1	0.560198000	-3.811086000	6.702538000
8	-3.595458000	-2.651588000	5.430018000
1	-3.899399000	-1.954283000	4.746792000
1	-3.312838000	-2.150505000	6.270654000
1	3.938114000	3.413210000	7.202761000
1	3.607320000	6.651334000	3.562086000
1	-0.263131000	8.028120000	6.461919000
1	-1.522575000	9.339523000	2.003665000
1	-5.369973000	7.111222000	2.827383000
1	-5.177992000	8.602066000	-3.700785000
1	-1.647996000	9.358574000	-7.377688000
1	1.642703000	10.792172000	-4.041450000
1	5.989200000	6.012269000	-5.879196000
1	5.718523000	6.239391000	-0.957863000
1	7.942584000	-0.411867000	-2.054018000
1	10.401288000	3.250909000	0.151228000
1	6.482908000	3.105712000	3.322133000
1	8.914701000	-0.444625000	4.683622000
1	4.548985000	-0.840588000	-8.351874000
1	7.217428000	-1.509419000	-4.428021000
1	-7.042101000	-3.360867000	6.236238000
1	-7.082689000	1.208402000	4.533246000
1	-4.451578000	-4.689900000	2.828330000
1	-9.221876000	-0.953654000	0.221806000
1	8.496434000	-2.822503000	1.146107000
1	-9.243479000	7.564887000	-2.926381000
1	1.270213000	0.882617000	-5.854084000
1	4.369423000	-4.819683000	-2.236960000
1	0.028292000	-2.535385000	-0.457819000
1	4.757567000	-0.048850000	4.264300000

^{11}TS1$_B$:

6	8.807846000	-1.286417000	3.257449000
6	8.100051000	-0.147475000	2.537499000
8	8.120712000	-0.097181000	1.272398000
6	7.894578000	-2.538611000	3.455170000
6	7.030113000	-2.874606000	2.280092000
7	7.492593000	-3.219622000	1.001762000
6	5.664236000	-2.846285000	2.169778000
6	6.449341000	-3.373311000	0.148818000
7	5.333222000	-3.151867000	0.855080000
1	9.676071000	-1.550302000	2.645785000
1	8.532534000	-3.387955000	3.731533000
1	7.227187000	-2.363590000	4.305383000
1	4.913984000	-2.624763000	2.906738000
1	6.491378000	-3.598175000	-0.919107000
1	4.332756000	-3.046893000	0.481683000
7	7.453148000	0.769280000	3.299232000
6	6.546923000	1.783140000	2.749028000
6	7.065011000	2.579931000	1.582627000
8	6.271185000	2.958804000	0.674876000
1	7.335237000	0.637585000	4.302886000
1	5.597458000	1.346801000	2.426350000
7	8.372917000	2.918661000	1.501855000
6	8.846445000	3.696094000	0.353789000
6	8.446726000	3.086453000	-0.996879000
8	8.201546000	3.831338000	-1.977658000
1	9.007412000	2.657262000	2.241628000
1	8.433472000	4.707965000	0.366476000
7	8.410969000	1.725862000	-1.076529000
6	8.063038000	1.066944000	-2.328996000
6	6.574076000	1.059508000	-2.693151000
8	6.234351000	0.645373000	-3.834203000
1	8.491752000	1.154425000	-0.235672000
1	8.575575000	1.560799000	-3.158988000
7	5.702234000	1.491102000	-1.755901000
6	4.265616000	1.662858000	-2.027229000
6	3.916971000	3.160698000	-2.071426000
8	3.358852000	3.665691000	-3.086645000
6	3.373976000	0.918960000	-1.012498000
6	3.574556000	-0.602320000	-1.023511000
6	2.629399000	-1.321792000	-0.074460000
8	1.595672000	-0.691490000	0.332762000
8	2.888216000	-2.553974000	0.272234000
1	6.059467000	1.835320000	-0.866292000
1	4.097037000	1.281502000	-3.035305000
1	2.328377000	1.138597000	-1.254348000
1	3.536235000	1.314360000	-0.003060000
1	4.610019000	-0.865691000	-0.776996000
1	3.376047000	-1.019318000	-2.021744000
7	4.250367000	3.879492000	-0.972488000
6	3.936514000	5.292761000	-0.812298000
6	4.646497000	6.239608000	-1.766965000
8	4.188601000	7.410750000	-1.912389000
1	4.705083000	3.427250000	-0.181108000
1	2.865499000	5.484967000	-0.951935000
7	5.685345000	5.787217000	-2.504345000
6	6.172186000	6.576263000	-3.635404000
6	5.079999000	6.899309000	-4.668938000
8	5.237516000	7.867702000	-5.447043000
1	6.149257000	4.912783000	-2.281969000
1	6.569754000	7.540854000	-3.309157000
7	3.974050000	6.095770000	-4.682408000
6	2.836393000	6.370403000	-5.557240000
6	1.630089000	6.969215000	-4.808835000
8	0.516490000	7.064709000	-5.394318000
6	2.405380000	5.113661000	-6.332297000
1	3.902439000	5.276284000	-4.078062000
1	3.174422000	7.139388000	-6.263453000
1	1.510876000	5.328527000	-6.920429000
1	3.213819000	4.797836000	-6.998890000
1	2.177169000	4.298270000	-5.636743000
7	1.814217000	7.440515000	-3.544395000
6	0.672854000	7.893375000	-2.741600000
6	-0.013103000	9.145008000	-3.310129000
8	-1.187647000	9.426965000	-2.952895000
6	1.065459000	8.141839000	-1.277549000
8	0.981787000	6.876025000	-0.553214000
1	2.724494000	7.374974000	-3.085991000
1	-0.106765000	7.122890000	-2.753653000
1	2.079867000	8.549477000	-1.216189000
1	0.354340000	8.861851000	-0.854947000
1	1.142669000	7.009796000	0.412178000
7	0.694414000	9.900545000	-4.185317000
6	0.096788000	11.034821000	-4.881319000
6	-0.983167000	10.682499000	-5.915852000
8	-1.655958000	11.613535000	-6.414194000
1	1.629655000	9.602091000	-4.425689000
1	-0.384630000	11.702008000	-4.160819000
7	-1.156954000	9.365933000	-6.219800000
6	-2.247402000	8.928384000	-7.081688000
6	-3.473276000	8.373716000	-6.347802000
8	-4.402411000	7.855118000	-7.017692000
1	-0.542292000	8.644982000	-5.842734000
1	-2.577506000	9.783349000	-7.678981000
7	-3.490099000	8.489048000	-4.993945000
6	-4.626913000	8.052635000	-4.183015000
6	-4.274118000	6.969942000	-3.148475000
6	-3.889429000	5.592306000	-3.673337000
6	-4.134789000	5.164053000	-4.990891000
6	-3.316542000	4.667880000	-2.781946000
6	-3.823236000	3.860909000	-5.393864000
6	-3.005861000	3.361461000	-3.168545000
6	-3.261036000	2.956431000	-4.486598000
8	-2.971411000	1.676675000	-4.940535000
1	-2.702646000	8.910771000	-4.512358000
1	-5.398211000	7.708830000	-4.876254000
1	-5.152061000	6.861565000	-2.490571000

1	-3.461245000	7.354753000	-2.514049000
1	-4.551905000	5.845910000	-5.725024000
1	-3.122813000	4.971781000	-1.754780000
1	-4.009137000	3.534522000	-6.410799000
1	-2.579372000	2.656905000	-2.461462000
1	-2.551100000	1.090254000	-4.262079000
6	-4.697992000	6.239422000	2.431099000
6	-3.217871000	6.486520000	2.282293000
8	-2.354120000	5.603488000	2.549559000
6	-5.229808000	5.131118000	1.460622000
6	-5.950503000	3.992511000	2.203084000
6	-4.951203000	3.079074000	2.917993000
8	-4.028508000	2.503570000	2.274561000
8	-5.147657000	3.011853000	4.219858000
1	-4.859623000	5.908966000	3.465306000
1	-5.925211000	5.579012000	0.743828000
1	-4.406526000	4.697172000	0.884261000
1	-6.497413000	3.355019000	1.502210000
1	-6.681773000	4.380792000	2.917208000
7	-2.806339000	7.727630000	1.873294000
6	-1.406873000	7.965100000	1.506632000
6	-0.409765000	7.637662000	2.615124000
8	0.729583000	7.176290000	2.320054000
1	-3.498844000	8.398412000	1.571106000
1	-1.112927000	7.358189000	0.644059000
7	-0.764082000	7.898431000	3.897853000
6	0.131085000	7.596025000	5.019004000
6	0.246844000	6.098016000	5.356672000
8	1.325195000	5.648875000	5.818783000
1	-1.680789000	8.285236000	4.075316000
1	1.139931000	7.943536000	4.790125000
7	-0.863093000	5.349592000	5.152377000
6	-0.903892000	3.882513000	5.344786000
6	0.075510000	3.227840000	4.347483000
8	0.982452000	2.446480000	4.736031000
6	-2.366791000	3.441147000	5.147759000
6	-2.744687000	2.085917000	5.723363000
8	-2.080388000	1.547220000	6.660374000
8	-3.823740000	1.530834000	5.224291000
1	-1.615106000	5.741441000	4.597508000
1	-0.550265000	3.626773000	6.344895000
1	-2.628793000	3.439654000	4.088902000
1	-3.022992000	4.173971000	5.636472000
7	-0.058557000	3.610349000	3.050461000
6	0.844754000	3.108115000	2.008248000
6	2.292115000	3.557546000	2.266729000
8	3.263377000	2.803177000	1.989687000
6	0.368548000	3.549099000	0.603265000
6	-1.020194000	3.025150000	0.150902000
6	-1.100011000	1.516190000	0.057947000
8	-0.742770000	0.835639000	1.071734000
8	-1.534877000	0.981385000	-1.056873000
1	-0.838014000	4.202327000	2.777380000
1	0.859348000	2.018869000	2.050154000
1	1.125954000	3.214338000	-0.114637000
1	0.348651000	4.643592000	0.539968000
1	-1.805681000	3.344026000	0.849266000
1	-1.248105000	3.458558000	-0.824939000
7	2.470549000	4.799346000	2.795058000
6	3.785212000	5.305342000	3.170984000
6	4.507144000	4.461566000	4.222820000
8	5.753044000	4.292149000	4.128805000
1	1.682126000	5.429305000	2.886795000
1	4.468005000	5.334721000	2.317412000
7	3.771990000	3.929140000	5.227935000
6	4.390052000	3.012587000	6.187098000
6	4.607499000	1.595571000	5.628498000
8	5.743006000	1.035529000	5.701708000
1	2.793024000	4.181412000	5.337174000
1	5.373143000	3.385925000	6.478228000
7	3.531585000	0.987738000	5.080744000
6	3.647201000	-0.355484000	4.498942000
6	2.558960000	-0.639645000	3.458435000
6	1.196795000	-0.864026000	4.038999000
7	0.078048000	-1.095488000	3.230461000
6	0.777773000	-0.852776000	5.354966000
6	-0.980566000	-1.201122000	4.049327000
7	-0.590616000	-1.065655000	5.335635000
1	2.649746000	1.493306000	4.967528000
1	3.625316000	-1.111081000	5.298323000
1	2.849440000	-1.526297000	2.877870000
1	2.528245000	0.190165000	2.741717000
1	1.305437000	-0.641913000	6.268620000
1	-2.002765000	-1.344336000	3.745922000
6	1.577345000	0.682978000	-4.788314000
6	2.460327000	-0.291136000	-5.533470000
8	2.340180000	-1.548395000	-5.392109000
6	0.250167000	0.991154000	-5.540872000
6	-0.641835000	-0.228695000	-5.850700000
6	-1.075505000	-1.039509000	-4.636956000
8	-1.515645000	-0.306495000	-3.583869000
8	-1.031772000	-2.282076000	-4.617482000
1	1.336580000	0.242499000	-3.815147000
1	-0.315551000	1.698735000	-4.929244000
1	0.479178000	1.502125000	-6.485303000
1	-0.155350000	-0.924506000	-6.539673000
1	-1.551434000	0.150021000	-6.334206000
7	3.372999000	0.208979000	-6.406851000
6	4.105761000	-0.641928000	-7.352623000
6	5.008595000	-1.717452000	-6.738682000
8	5.237149000	-2.764087000	-7.385701000
1	3.468263000	1.211690000	-6.483681000
1	3.405927000	-1.182409000	-7.996232000
7	5.533399000	-1.461560000	-5.508798000
6	6.359965000	-2.433212000	-4.810089000
6	5.624873000	-3.198588000	-3.707328000
8	6.275382000	-3.684161000	-2.733180000
1	5.448052000	-0.550759000	-5.063305000
1	6.727281000	-3.161863000	-5.542135000
7	4.298088000	-3.365504000	-3.863753000
6	3.482936000	-4.183391000	-2.955546000
6	1.998930000	-4.100869000	-3.323787000
8	1.552871000	-2.710682000	-3.141011000
6	1.134826000	-5.013953000	-2.460948000
1	3.829746000	-2.880227000	-4.625349000
1	3.602559000	-3.832173000	-1.925296000
1	1.860645000	-4.356667000	-4.382513000
1	1.619997000	-2.221102000	-4.007557000
1	0.084217000	-4.903838000	-2.742991000
1	1.423319000	-6.061668000	-2.600249000
1	1.239160000	-4.764172000	-1.399984000
6	-5.451106000	-4.513528000	3.168489000
6	-6.596324000	-3.817772000	3.892116000
8	-7.692411000	-3.599050000	3.301851000
6	-5.990482000	-5.667844000	2.298377000
6	-4.924227000	-6.337713000	1.393564000
6	-4.210746000	-5.323703000	0.518543000
8	-3.103502000	-4.824971000	0.872773000
7	-4.827674000	-4.905181000	-0.606009000
1	-4.951320000	-3.770739000	2.532198000
1	-6.811786000	-5.274438000	1.690051000
1	-6.427749000	-6.439596000	2.944572000
1	-5.408365000	-7.106251000	0.778147000
1	-4.162382000	-6.831660000	2.004446000
1	-4.402725000	-4.129658000	-1.128642000
1	-5.701962000	-5.302322000	-0.911389000
7	-6.381990000	-3.446975000	5.181836000
6	-7.458142000	-2.878066000	5.992962000
6	-8.036876000	-1.543711000	5.511775000
8	-9.198656000	-1.219612000	5.850894000
1	-5.432317000	-3.453509000	5.560283000
1	-8.305445000	-3.568144000	6.031924000
7	-7.221406000	-0.767543000	4.748019000
6	-7.680694000	0.484470000	4.169059000
6	-7.822467000	0.453791000	2.647256000
8	-7.868551000	1.541151000	2.011181000
1	-6.246694000	-1.025384000	4.574809000
1	-8.661199000	0.709788000	4.603071000
7	-7.928870000	-0.766844000	2.060873000
6	-8.148015000	-0.930074000	0.624742000

6	-6.969274000	-1.555729000	-0.116094000
8	-5.841756000	-0.633805000	-0.144382000
1	-7.927076000	-1.604887000	2.640260000
1	-8.359404000	0.056285000	0.206763000
1	-6.671901000	-2.498001000	0.364448000
1	-7.277080000	-1.773526000	-1.146812000
1	-5.195280000	-0.838720000	-0.881840000
6	-9.505758000	6.295926000	-2.962890000
6	-8.766275000	5.124838000	-2.350466000
6	-9.202461000	4.546088000	-1.145437000
6	-7.642150000	4.570748000	-2.988482000
6	-8.543770000	3.438821000	-0.596474000
6	-6.978400000	3.464255000	-2.444807000
6	-7.427945000	2.892985000	-1.247294000
1	-10.291463000	5.955457000	-3.651640000
1	-9.992068000	6.910073000	-2.196380000
1	-10.068067000	4.964593000	-0.636923000
1	-7.283689000	5.004533000	-3.918130000
1	-8.879740000	2.997062000	0.335888000
1	-6.110747000	3.057398000	-2.953867000
1	-6.920727000	2.032464000	-0.820839000
26	-0.255465000	-1.215634000	1.155908000
26	-1.921216000	-0.843266000	-1.754865000
8	0.277684000	-3.258725000	0.813406000
1	1.266111000	-3.289204000	0.743002000
1	-0.169275000	-3.889791000	1.520428000
8	-4.393807000	-0.853538000	4.133032000
1	-4.219474000	-0.000610000	4.619383000
1	-4.302813000	-0.680791000	3.136097000
8	-4.083435000	-0.105544000	1.663305000
1	-4.876788000	-0.272196000	1.044426000
1	-3.952270000	0.869169000	1.827328000
8	-2.153307000	-1.464385000	0.450300000
1	-2.827386000	-2.281536000	-0.211928000
1	-2.813773000	-0.874212000	0.945692000
8	-0.767710000	-2.407112000	8.765832000
1	-0.390768000	-3.040352000	8.067026000
1	-0.744420000	-2.768515000	9.664475000
8	-3.807537000	-1.249977000	-1.736876000
8	-3.355564000	-2.615070000	-1.246994000
8	-0.385382000	-2.139946000	-1.537144000
1	0.319910000	-2.347212000	-2.242585000
8	-1.002097000	-4.656063000	2.446346000
1	-1.835637000	-4.862033000	1.944247000
1	-1.194502000	-4.447542000	3.394500000
8	-2.401749000	-1.078934000	7.362280000
1	-1.836517000	-1.478758000	8.107563000
1	-2.464502000	-0.086059000	7.326417000
8	-1.849070000	-4.424540000	5.087330000
1	-2.169390000	-5.336332000	5.214605000
1	-2.603804000	-3.720696000	5.313435000
8	0.090270000	-3.837451000	6.793486000
1	-0.622423000	-4.110897000	6.134232000
1	0.830207000	-3.417270000	6.321832000
8	-3.593669000	-2.718331000	5.709522000
1	-3.909157000	-2.065927000	4.989915000
1	-3.263189000	-2.153071000	6.484649000
1	3.746054000	2.961404000	7.069918000
1	3.651612000	6.325109000	3.544138000
1	-0.226812000	8.135504000	5.901013000
1	-1.304105000	9.019445000	1.229241000
1	-5.261511000	7.169950000	2.300929000
1	-5.032123000	8.921953000	-3.647441000
1	-1.897265000	8.148063000	-7.762748000
1	0.889320000	11.596479000	-5.382595000
1	6.979842000	6.016333000	-4.114542000
1	4.183183000	5.575380000	0.214080000
1	8.406755000	0.029258000	-2.288031000
1	9.937000000	3.765508000	0.411313000
1	6.324622000	2.500778000	3.546765000
1	9.177166000	-0.970859000	4.239029000
1	4.713902000	0.011350000	-7.985891000
1	7.213985000	-1.931553000	-4.352052000
1	-7.077488000	-2.750223000	7.011317000
1	-6.999916000	1.303373000	4.417736000
1	-4.698082000	-4.871231000	3.880282000
1	-9.026615000	-1.570114000	0.471025000
1	8.459957000	-3.262976000	0.721518000
1	-8.831780000	6.940476000	-3.538149000
1	2.106374000	1.625563000	-4.599184000
1	3.824774000	-5.225410000	-3.000326000
1	-0.324543000	-2.784085000	-0.777039000
1	4.635015000	-0.425780000	4.031633000

^{11}IM1$_B$:

6	8.517404000	-1.122238000	3.615441000
6	7.928629000	-0.048249000	2.710537000
8	7.985147000	-0.199468000	1.453003000
6	7.596881000	-2.379135000	3.770289000
6	6.817189000	-2.731237000	2.542789000
7	7.361172000	-3.121859000	1.311802000
6	5.470431000	-2.619292000	2.318909000
6	6.387268000	-3.207935000	0.373116000
7	5.232437000	-2.908558000	0.981554000
1	9.467339000	-1.420047000	3.158727000
1	8.215393000	-3.220046000	4.107603000
1	6.870638000	-2.188412000	4.566777000
1	4.678039000	-2.344112000	2.989734000
1	6.502315000	-3.433457000	-0.688342000
1	4.277967000	-2.734365000	0.532114000
7	7.379101000	1.042333000	3.297803000
6	6.643554000	2.073142000	2.555431000
6	7.345754000	2.662374000	1.358487000
8	6.657547000	3.149651000	0.415897000
1	7.168152000	1.048673000	4.299794000
1	5.680924000	1.698108000	2.190875000
7	8.694613000	2.663628000	1.283690000
6	9.372803000	3.119211000	0.068164000
6	8.884936000	2.439722000	-1.222200000
8	8.976585000	3.050896000	-2.309718000
1	9.230502000	2.284360000	2.049792000
1	9.227064000	4.191441000	-0.084200000
7	8.388126000	1.169443000	-1.122673000
6	7.879621000	0.490877000	-2.308400000
6	6.449152000	0.858450000	-2.725127000
8	5.998852000	0.442375000	-3.827047000
1	8.315508000	0.703731000	-0.217256000
1	8.520204000	0.719915000	-3.164276000
7	5.716741000	1.589953000	-1.851993000
6	4.319756000	1.952829000	-2.112392000
6	4.091924000	3.471195000	-2.137157000
8	3.108378000	3.945457000	-2.785344000
6	3.344303000	1.269770000	-1.113494000
6	3.517565000	-0.258562000	-1.088836000
6	2.570543000	-0.968124000	-0.134629000
8	1.508185000	-0.363717000	0.233727000
8	2.866651000	-2.179506000	0.257444000
1	6.144340000	1.880278000	-0.975714000
1	4.103065000	1.597225000	-3.123279000
1	2.320680000	1.517943000	-1.410670000
1	3.483823000	1.687348000	-0.109376000
1	4.546013000	-0.530840000	-0.824674000
1	3.332827000	-0.692676000	-2.082898000
7	4.949760000	4.259859000	-1.450250000
6	4.733461000	5.696682000	-1.293492000
6	4.721067000	6.494179000	-2.595854000
8	4.055863000	7.555652000	-2.678834000
1	5.646039000	3.845669000	-0.832417000
1	3.781570000	5.898026000	-0.796403000
7	5.457071000	6.001237000	-3.628870000
6	5.454809000	6.660233000	-4.934050000
6	4.090504000	6.654959000	-5.641041000
8	3.831215000	7.541199000	-6.482519000
1	6.014822000	5.171081000	-3.480634000
1	5.735135000	7.711812000	-4.835366000
7	3.232483000	5.640704000	-5.320055000
6	1.844662000	5.638475000	-5.794937000
6	0.899888000	6.390141000	-4.838874000
8	-0.067304000	7.047393000	-5.296019000
6	1.341392000	4.203780000	-6.019662000

1	3.484758000	4.971705000	-4.599851000
1	1.819819000	6.205932000	-6.727398000
1	0.287504000	4.213525000	-6.311294000
1	1.923251000	3.723996000	-6.814010000
1	1.436439000	3.615271000	-5.100408000
7	1.165165000	6.288699000	-3.509204000
6	0.330124000	6.891141000	-2.461189000
6	-0.011647000	8.361395000	-2.733530000
8	-1.143538000	8.823163000	-2.440603000
6	1.101085000	6.755866000	-1.136214000
8	0.201412000	7.095822000	-0.061435000
1	1.911917000	5.669332000	-3.208956000
1	-0.629957000	6.370310000	-2.379594000
1	1.442729000	5.716269000	-1.037422000
1	1.978058000	7.414613000	-1.153842000
1	0.643729000	7.186486000	0.816049000
7	0.979846000	9.127242000	-3.262025000
6	0.773851000	10.541796000	-3.566373000
6	-0.298744000	10.861889000	-4.620019000
8	-0.697941000	12.046738000	-4.717901000
1	1.883954000	8.707125000	-3.445989000
1	0.471045000	11.086297000	-2.666683000
7	-0.747630000	9.836617000	-5.390047000
6	-1.839659000	10.001083000	-6.339341000
6	-3.130251000	9.267578000	-5.959570000
8	-4.003860000	9.056211000	-6.838892000
1	-0.374471000	8.894492000	-5.283932000
1	-2.056871000	11.071857000	-6.412049000
7	-3.275199000	8.908894000	-4.656393000
6	-4.501006000	8.281510000	-4.157694000
6	-4.233413000	7.176316000	-3.124971000
6	-3.698608000	5.845031000	-3.639355000
6	-3.406216000	5.583146000	-4.989049000
6	-3.512689000	4.798468000	-2.717079000
6	-2.946887000	4.325496000	-5.397253000
6	-3.056080000	3.536841000	-3.109645000
6	-2.766289000	3.301350000	-4.460712000
8	-2.300931000	2.076265000	-4.921977000
1	-2.522123000	9.074359000	-3.994143000
1	-5.050490000	7.905832000	-5.025364000
1	-5.182485000	6.984254000	-2.601837000
1	-3.538838000	7.572517000	-2.369521000
1	-3.530618000	6.358860000	-5.735984000
1	-3.753058000	4.966676000	-1.669633000
1	-2.729602000	4.125939000	-6.440731000
1	-2.929010000	2.741174000	-2.383071000
1	-2.109824000	1.404634000	-4.218479000
6	-4.837258000	6.241162000	2.695357000
6	-3.381696000	6.626341000	2.595618000
8	-2.457789000	5.779646000	2.771667000
6	-5.267832000	5.239905000	1.571748000
6	-5.990379000	3.998061000	2.129066000
6	-4.997891000	3.055925000	2.817977000
8	-4.077328000	2.494619000	2.163579000
8	-5.193631000	2.965449000	4.121237000
1	-4.965013000	5.751386000	3.669032000
1	-5.933044000	5.747026000	0.865353000
1	-4.391922000	4.903214000	1.007384000
1	-6.452739000	3.423554000	1.322037000
1	-6.786175000	4.279712000	2.823339000
7	-3.063164000	7.929126000	2.336177000
6	-1.685689000	8.312015000	1.983608000
6	-0.654643000	7.870241000	3.020184000
8	0.471260000	7.417102000	2.665804000
1	-3.805383000	8.575392000	2.107374000
1	-1.390768000	7.881556000	1.020648000
7	-0.959774000	8.030913000	4.334406000
6	-0.015398000	7.646356000	5.387000000
6	0.130225000	6.128068000	5.588336000
8	1.217588000	5.658396000	6.008991000
1	-1.860874000	8.416032000	4.580850000
1	0.979608000	8.027169000	5.149431000
7	-0.967989000	5.384577000	5.320600000
6	-0.997977000	3.908311000	5.403327000
6	-0.028433000	3.334311000	4.348805000
8	0.912480000	2.564563000	4.676275000
6	-2.462235000	3.471633000	5.197256000
6	-2.818667000	2.077175000	5.687696000
8	-2.193545000	1.539112000	6.653331000
8	-3.831386000	1.493566000	5.095929000
1	-1.716339000	5.810825000	4.785782000
1	-0.629232000	3.583617000	6.377614000
1	-2.741229000	3.546211000	4.146973000
1	-3.110739000	4.164200000	5.751227000
7	-0.210045000	3.767591000	3.075089000
6	0.663110000	3.315558000	1.985374000
6	2.105868000	3.805829000	2.182989000
8	3.087134000	3.095715000	1.834667000
6	0.113585000	3.772632000	0.613577000
6	-1.251809000	3.179258000	0.176111000
6	-1.240157000	1.672468000	0.058137000
8	-0.872858000	1.007555000	1.079480000
8	-1.604641000	1.123251000	-1.077991000
1	-1.004787000	4.361088000	2.850402000
1	0.712245000	2.227136000	2.004468000
1	0.868746000	3.510936000	-0.137852000
1	0.009387000	4.864467000	0.605402000
1	-2.036379000	3.435396000	0.899813000
1	-1.526090000	3.619004000	-0.784571000
7	2.275426000	5.042230000	2.729800000
6	3.605559000	5.566640000	3.016719000
6	4.389793000	4.744405000	4.043359000
8	5.626481000	4.563339000	3.883370000
1	1.476706000	5.637071000	2.921308000
1	4.229579000	5.577003000	2.119046000
7	3.713624000	4.248365000	5.108890000
6	4.391914000	3.370890000	6.062413000
6	4.605563000	1.944428000	5.531786000
8	5.745446000	1.389894000	5.605330000
1	2.744084000	4.507914000	5.280045000
1	5.382017000	3.765242000	6.297593000
7	3.528447000	1.320799000	5.009719000
6	3.648671000	-0.019747000	4.423698000
6	2.534129000	-0.317336000	3.414647000
6	1.203809000	-0.625991000	4.027744000
7	0.081020000	-0.889069000	3.235793000
6	0.812689000	-0.668608000	5.351099000
6	-0.955972000	-1.062222000	4.070419000
7	-0.546105000	-0.943103000	5.351745000
1	2.633242000	1.808517000	4.916734000
1	3.664207000	-0.777562000	5.221532000
1	2.838941000	-1.165870000	2.787548000
1	2.442021000	0.536034000	2.731334000
1	1.349040000	-0.449713000	6.257801000
1	-1.977873000	-1.239310000	3.782579000
6	1.724246000	0.340865000	-5.013825000
6	2.540508000	-0.705087000	-5.735462000
8	2.410898000	-1.947567000	-5.509602000
6	0.408015000	0.724861000	-5.743774000
6	-0.636960000	-0.399735000	-5.879159000
6	-1.055802000	-1.054970000	-4.569157000
8	-1.369302000	-0.214625000	-3.555561000
8	-1.092990000	-2.295205000	-4.449995000
1	1.482053000	-0.043068000	-4.017952000
1	-0.046229000	1.554369000	-5.198317000
1	0.645134000	1.101247000	-6.748140000
1	-0.287169000	-1.203269000	-6.533568000
1	-1.531750000	0.045020000	-6.332963000
7	3.429046000	-0.275538000	-6.671881000
6	4.164424000	-1.205386000	-7.533956000
6	5.147583000	-2.134593000	-6.814697000
8	5.467543000	-3.219555000	-7.349096000
1	3.517168000	0.716427000	-6.838473000
1	3.469508000	-1.863137000	-8.063039000
7	5.630350000	-1.713992000	-5.611884000
6	6.489195000	-2.552183000	-4.792148000
6	5.778456000	-3.141508000	-3.572465000
8	6.444469000	-3.419268000	-2.529049000
1	5.440142000	-0.787522000	-5.241919000
1	6.850651000	-3.377518000	-5.416261000

7	4.460379000	-3.388993000	-3.699203000
6	3.669597000	-4.081566000	-2.672518000
6	2.205775000	-4.232763000	-3.103660000
8	1.586858000	-2.904127000	-3.158735000
6	1.409125000	-5.098536000	-2.132744000
1	3.976927000	-3.054966000	-4.529436000
1	3.687324000	-3.517091000	-1.734491000
1	2.160833000	-4.671415000	-4.110028000
1	1.648870000	-2.529090000	-4.078652000
1	0.366561000	-5.154976000	-2.457408000
1	1.817026000	-6.114670000	-2.093700000
1	1.431385000	-4.673987000	-1.124151000
6	-5.297341000	-4.582006000	3.125919000
6	-6.490503000	-3.909518000	3.790396000
8	-7.572326000	-3.744008000	3.159407000
6	-5.754339000	-5.806728000	2.307328000
6	-4.648406000	-6.415462000	1.403481000
6	-4.110792000	-5.383773000	0.432569000
8	-3.127170000	-4.646333000	0.743668000
7	-4.766677000	-5.202752000	-0.731386000
1	-4.823033000	-3.847671000	2.461986000
1	-6.612844000	-5.503357000	1.698353000
1	-6.113314000	-6.590222000	2.986733000
1	-5.057931000	-7.279025000	0.865640000
1	-3.809477000	-6.768185000	2.011919000
1	-4.493162000	-4.407474000	-1.313781000
1	-5.558048000	-5.769312000	-0.991930000
7	-6.329517000	-3.496736000	5.075703000
6	-7.448153000	-2.936249000	5.831885000
6	-8.025026000	-1.616703000	5.310372000
8	-9.196105000	-1.294430000	5.617704000
1	-5.391227000	-3.463609000	5.479977000
1	-8.285167000	-3.639717000	5.841048000
7	-7.198574000	-0.849499000	4.549091000
6	-7.648915000	0.397088000	3.952017000
6	-7.751845000	0.363571000	2.427509000
8	-7.805330000	1.450438000	1.792084000
1	-6.218965000	-1.102112000	4.401629000
1	-8.642397000	0.617643000	4.358213000
7	-7.822217000	-0.859133000	1.837776000
6	-8.037246000	-1.025519000	0.400144000
6	-6.834033000	-1.576230000	-0.360228000
8	-5.771897000	-0.577989000	-0.413336000
1	-7.814058000	-1.694580000	2.419626000
1	-8.309513000	-0.050104000	-0.008737000
1	-6.459641000	-2.494064000	0.111342000
1	-7.145790000	-1.817870000	-1.384190000
1	-5.106071000	-0.784706000	-1.128023000
6	-9.449616000	6.317328000	-3.072807000
6	-8.735594000	5.127827000	-2.465782000
6	-9.187693000	4.548896000	-1.266718000
6	-7.619101000	4.557044000	-3.102646000
6	-8.551320000	3.426747000	-0.721590000
6	-6.980353000	3.433856000	-2.563645000
6	-7.443564000	2.863649000	-1.370958000
1	-10.226954000	5.997462000	-3.780727000
1	-9.941853000	6.925272000	-2.305308000
1	-10.047555000	4.980365000	-0.759286000
1	-7.247773000	4.993722000	-4.026098000
1	-8.898077000	2.985872000	0.207073000
1	-6.116761000	3.014591000	-3.069587000
1	-6.952486000	1.993452000	-0.945957000
26	-0.264404000	-1.005404000	1.174740000
26	-1.818529000	-0.767212000	-1.703413000
8	0.352956000	-3.026255000	0.820542000
1	1.342331000	-3.009735000	0.764986000
1	-0.112178000	-3.679931000	1.495450000
8	-4.356615000	-0.885231000	3.962407000
1	-4.202668000	-0.024717000	4.442207000
1	-4.281677000	-0.721166000	2.961888000
8	-4.077964000	-0.122464000	1.489810000
1	-4.843788000	-0.261479000	0.832726000
1	-3.946979000	0.845515000	1.681678000
8	-2.022520000	-1.361441000	0.257498000
1	-3.106942000	-2.576152000	-0.446982000
1	-2.765142000	-0.870734000	0.737544000
8	-0.861254000	-2.424840000	8.755118000
1	-0.440058000	-3.017404000	8.047555000
1	-0.862052000	-2.820502000	9.639384000
8	-3.658371000	-1.349984000	-1.912770000
8	-3.754406000	-2.686631000	-1.215516000
8	-0.372699000	-2.157849000	-1.593600000
1	0.272590000	-2.454447000	-2.311518000
8	-1.005516000	-4.456687000	2.345082000
1	-1.836834000	-4.620774000	1.824565000
1	-1.192136000	-4.326274000	3.309800000
8	-2.434545000	-1.082358000	7.301108000
1	-1.910464000	-1.487062000	8.071944000
1	-2.527143000	-0.089926000	7.275166000
8	-1.788003000	-4.368055000	4.998935000
1	-2.083166000	-5.287024000	5.135493000
1	-2.562244000	-3.678647000	5.228081000
8	0.106036000	-3.741079000	6.748743000
1	-0.580708000	-4.023773000	6.066975000
1	0.833355000	-3.268481000	6.308183000
8	-3.563025000	-2.702011000	5.603441000
1	-3.876923000	-2.069642000	4.866190000
1	-3.250616000	-2.126620000	6.382279000
1	3.791022000	3.335554000	6.976014000
1	3.487829000	6.594122000	3.374779000
1	-0.341943000	8.099965000	6.327605000
1	-1.653741000	9.402751000	1.889171000
1	-5.481120000	7.127537000	2.691773000
1	-5.136515000	9.047689000	-3.688899000
1	-1.550748000	9.643712000	-7.331755000
1	1.725779000	10.957896000	-3.907603000
1	6.195376000	6.166917000	-5.570513000
1	5.533444000	6.076690000	-0.649995000
1	7.901684000	-0.591625000	-2.141709000
1	10.444880000	2.935420000	0.186890000
1	6.411335000	2.896847000	3.245067000
1	8.738601000	-0.729592000	4.612664000
1	4.709747000	-0.618144000	-8.278884000
1	7.345506000	-1.980624000	-4.431758000
1	-7.116562000	-2.790209000	6.864823000
1	-6.980930000	1.220945000	4.218109000
1	-4.542402000	-4.864776000	3.868206000
1	-8.878978000	-1.713948000	0.250284000
1	8.345044000	-3.206375000	1.110402000
1	-8.757796000	6.962724000	-3.625412000
1	2.323382000	1.250110000	-4.878265000
1	4.111376000	-5.069045000	-2.487966000
1	-0.238114000	-2.664915000	-0.739338000
1	4.621448000	-0.071498000	3.924226000

[11]TS2$_B$:

6	8.755158000	-3.020456000	3.158368000
6	8.050935000	-1.878300000	2.439900000
8	8.081540000	-1.818929000	1.175254000
6	7.833462000	-4.264355000	3.365374000
6	6.968445000	-4.602350000	2.191330000
7	7.431340000	-4.937559000	0.910632000
6	5.602007000	-4.585406000	2.083676000
6	6.387018000	-5.096231000	0.059407000
7	5.270395000	-4.887882000	0.768499000
1	9.618119000	-3.292062000	2.542709000
1	8.465488000	-5.115947000	3.648674000
1	7.166383000	-4.077909000	4.213429000
1	4.851254000	-4.373898000	2.823141000
1	6.432569000	-5.313736000	-1.009934000
1	4.263407000	-4.784640000	0.396971000
7	7.396731000	-0.969626000	3.204681000
6	6.488083000	0.045373000	2.660787000
6	7.001078000	0.846555000	1.495649000
8	6.204081000	1.226599000	0.590702000
1	7.268629000	-1.113558000	4.205839000
1	5.537153000	-0.390054000	2.341324000
7	8.307448000	1.190075000	1.411579000
6	8.773612000	1.970811000	0.262940000
6	8.362234000	1.366663000	-1.086851000

8	8.102968000	2.116290000	-2.060935000
1	8.946138000	0.926121000	2.146771000
1	8.362180000	2.983232000	0.282034000
7	8.332405000	0.006740000	-1.174072000
6	7.976261000	-0.647052000	-2.427205000
6	6.485044000	-0.652681000	-2.782504000
8	6.138601000	-1.051707000	-3.927196000
1	8.424009000	-0.568830000	-0.336774000
1	8.484135000	-0.150947000	-3.258656000
7	5.618004000	-0.237601000	-1.833552000
6	4.177895000	-0.075265000	-2.091560000
6	3.817083000	1.419889000	-2.116510000
8	3.254777000	1.933820000	-3.126198000
6	3.299876000	-0.840148000	-1.081035000
6	3.498125000	-2.362131000	-1.125284000
6	2.553400000	-3.092855000	-0.182295000
8	1.504227000	-2.476291000	0.198001000
8	2.831369000	-4.314981000	0.185686000
1	5.979400000	0.099503000	-0.942791000
1	4.006295000	-0.446658000	-3.102676000
1	2.250495000	-0.616995000	-1.301882000
1	3.476356000	-0.464066000	-0.066285000
1	4.534430000	-2.633738000	-0.892777000
1	3.286896000	-2.758020000	-2.129578000
7	4.144291000	2.129006000	-1.010020000
6	3.822968000	3.538864000	-0.835680000
6	4.529884000	4.497782000	-1.780806000
8	4.061402000	5.665449000	-1.924647000
1	4.602863000	1.672288000	-0.222951000
1	2.751738000	3.727743000	-0.975763000
7	5.578040000	4.059811000	-2.513336000
6	6.064870000	4.859732000	-3.636743000
6	4.974623000	5.179603000	-4.672788000
8	5.123176000	6.155566000	-5.443266000
1	6.048651000	3.187574000	-2.295358000
1	6.453250000	5.825411000	-3.302763000
7	3.877433000	4.363999000	-4.696760000
6	2.741010000	4.632922000	-5.573660000
6	1.524933000	5.208120000	-4.823298000
8	0.405247000	5.276480000	-5.403642000
6	2.331739000	3.380196000	-6.366301000
1	3.810201000	3.540121000	-4.097376000
1	3.072363000	5.415270000	-6.268785000
1	1.437042000	3.588892000	-6.956440000
1	3.147888000	3.084898000	-7.032728000
1	2.114056000	2.552942000	-5.681513000
7	1.703878000	5.695180000	-3.564875000
6	0.555497000	6.145845000	-2.770393000
6	-0.154262000	7.365689000	-3.375912000
8	-1.348980000	7.613370000	-3.061168000
6	0.947053000	6.439297000	-1.313322000
8	0.867064000	5.192309000	-0.559532000
1	2.612828000	5.637668000	-3.100727000
1	-0.208843000	5.360712000	-2.757285000
1	1.959506000	6.853183000	-1.263685000
1	0.233271000	7.167854000	-0.909126000
1	1.053937000	5.339564000	0.399420000
7	0.557515000	8.136274000	-4.234611000
6	-0.047014000	9.253356000	-4.952808000
6	-1.103554000	8.873026000	-6.000970000
8	-1.789678000	9.785291000	-6.514995000
1	1.508360000	7.863554000	-4.443312000
1	-0.551000000	9.921481000	-4.248641000
7	-1.246052000	7.550756000	-6.298454000
6	-2.318708000	7.084386000	-7.166060000
6	-3.529065000	6.497790000	-6.433460000
8	-4.422769000	5.909469000	-7.095034000
1	-0.626065000	6.843645000	-5.903942000
1	-2.669794000	7.931831000	-7.762846000
7	-3.577847000	6.664567000	-5.084924000
6	-4.724110000	6.235934000	-4.283965000
6	-4.374531000	5.187430000	-3.214648000
6	-3.973587000	3.802337000	-3.685376000
6	-4.153371000	3.343287000	-5.012639000
6	-3.440713000	2.899325000	-2.730244000
6	-3.820807000	2.042032000	-5.363170000
6	-3.111513000	1.595884000	-3.057016000
6	-3.296509000	1.133264000	-4.395632000
8	-3.005759000	-0.107406000	-4.757204000
1	-2.813950000	7.128889000	-4.602682000
1	-5.481023000	5.865500000	-4.979409000
1	-5.256074000	5.079832000	-2.560746000
1	-3.572594000	5.594293000	-2.580245000
1	-4.536594000	4.012052000	-5.776878000
1	-3.301887000	3.241314000	-1.707562000
1	-3.953977000	1.683271000	-6.377454000
1	-2.711803000	0.905071000	-2.322049000
1	-2.363383000	-0.963506000	-4.097111000
6	-4.722328000	4.498449000	2.465357000
6	-3.249711000	4.780819000	2.308328000
8	-2.367943000	3.903354000	2.526666000
6	-5.243780000	3.438599000	1.436225000
6	-5.977643000	2.270275000	2.118168000
6	-4.983033000	1.357873000	2.843466000
8	-4.058024000	0.780536000	2.207431000
8	-5.183870000	1.305688000	4.146184000
1	-4.859328000	4.098566000	3.478109000
1	-5.924778000	3.921089000	0.727534000
1	-4.409772000	3.024567000	0.860089000
1	-6.489486000	1.644720000	1.381354000
1	-6.736634000	2.630788000	2.817726000
7	-2.860713000	6.042660000	1.939936000
6	-1.468258000	6.304043000	1.561101000
6	-0.452391000	5.953290000	2.646467000
8	0.683655000	5.503955000	2.323713000
1	-3.564850000	6.719115000	1.680889000
1	-1.181266000	5.720376000	0.680265000
7	-0.792290000	6.183952000	3.939208000
6	0.113911000	5.858046000	5.044582000
6	0.220414000	4.354863000	5.362106000
8	1.295665000	3.894548000	5.819748000
1	-1.709050000	6.561439000	4.135557000
1	1.122661000	6.199560000	4.806502000
7	-0.893391000	3.616021000	5.144118000
6	-0.944793000	2.145874000	5.311629000
6	0.045158000	1.504562000	4.315993000
8	0.954704000	0.726244000	4.704645000
6	-2.406726000	1.713357000	5.087805000
6	-2.803058000	0.365612000	5.668357000
8	-2.146437000	-0.175404000	6.609434000
8	-3.887663000	-0.179407000	5.170794000
1	-1.640656000	4.021537000	4.593563000
1	-0.606335000	1.871389000	6.312048000
1	-2.649911000	1.702122000	4.024401000
1	-3.068826000	2.453177000	5.557598000
7	-0.081721000	1.899945000	3.022606000
6	0.823627000	1.408221000	1.977117000
6	2.269017000	1.852573000	2.241895000
8	3.241569000	1.106645000	1.947035000
6	0.352716000	1.873655000	0.578623000
6	-1.029726000	1.351651000	0.112155000
6	-1.104475000	-0.158789000	-0.018971000
8	-0.722308000	-0.849312000	0.982865000
8	-1.557584000	-0.665532000	-1.133389000
1	-0.866213000	2.485106000	2.750152000
1	0.829989000	0.318195000	1.998963000
1	1.114846000	1.553441000	-0.141243000
1	0.330484000	2.969249000	0.537983000
1	-1.818905000	1.651130000	0.815028000
1	-1.255355000	1.808713000	-0.853884000
7	2.448115000	3.086532000	2.790168000
6	3.763293000	3.583270000	3.175795000
6	4.485112000	2.709752000	4.203669000
8	5.731233000	2.543331000	4.106537000
1	1.657851000	3.710758000	2.897986000
1	4.444443000	3.635812000	2.321739000
7	3.746870000	2.149569000	5.190976000
6	4.357498000	1.204404000	6.126647000
6	4.554134000	-0.204178000	5.539485000
8	5.686214000	-0.774815000	5.589987000

1	2.764901000	2.390165000	5.296366000
1	5.346585000	1.560084000	6.419157000
7	3.466606000	-0.793495000	4.995918000
6	3.561176000	-2.130243000	4.394549000
6	2.463201000	-2.390135000	3.357786000
6	1.100484000	-2.602758000	3.941951000
7	-0.018774000	-2.828788000	3.133537000
6	0.683029000	-2.586288000	5.258890000
6	-1.077164000	-2.926867000	3.952177000
7	-0.686690000	-2.790882000	5.240010000
1	2.588005000	-0.278231000	4.901640000
1	3.534648000	-2.895565000	5.184472000
1	2.740218000	-3.275316000	2.768414000
1	2.439178000	-1.554267000	2.647588000
1	1.212136000	-2.378414000	6.172502000
1	-2.100090000	-3.065723000	3.648491000
6	1.545452000	-1.035203000	-4.869689000
6	2.401860000	-2.018923000	-5.639104000
8	2.264784000	-3.273589000	-5.518466000
6	0.236145000	-0.644157000	-5.613309000
6	-0.729073000	-1.811843000	-5.927019000
6	-1.141679000	-2.603688000	-4.697415000
8	-1.768874000	-1.887696000	-3.711538000
8	-0.897524000	-3.807891000	-4.561457000
1	1.291131000	-1.496543000	-3.909609000
1	-0.283501000	0.093043000	-4.993404000
1	0.487965000	-0.141598000	-6.556200000
1	-0.280454000	-2.522491000	-6.625336000
1	-1.628744000	-1.387258000	-6.386783000
7	3.320116000	-1.513048000	-6.507136000
6	4.046738000	-2.361817000	-7.457814000
6	4.967158000	-3.425759000	-6.849322000
8	5.216101000	-4.463312000	-7.503342000
1	3.438786000	-0.511462000	-6.560076000
1	3.342477000	-2.912112000	-8.088018000
7	5.483240000	-3.167659000	-5.616315000
6	6.330379000	-4.124734000	-4.922508000
6	5.618814000	-4.891754000	-3.806152000
8	6.290157000	-5.364720000	-2.839886000
1	5.379005000	-2.261098000	-5.166296000
1	6.697092000	-4.852510000	-5.655655000
7	4.291794000	-5.074616000	-3.941547000
6	3.503763000	-5.902470000	-3.018310000
6	2.019037000	-5.887222000	-3.393032000
8	1.512478000	-4.518157000	-3.236153000
6	1.194699000	-6.815231000	-2.506323000
1	3.804791000	-4.595622000	-4.694932000
1	3.601378000	-5.524611000	-1.995179000
1	1.899191000	-6.177984000	-4.445677000
1	1.512499000	-4.047260000	-4.109982000
1	0.142575000	-6.774023000	-2.801120000
1	1.541198000	-7.850723000	-2.598311000
1	1.268909000	-6.513512000	-1.456688000
6	-5.576660000	-6.278604000	3.238926000
6	-6.726554000	-5.542637000	3.912500000
8	-7.817890000	-5.352893000	3.304572000
6	-6.102166000	-7.495671000	2.450546000
6	-5.036849000	-8.181298000	1.554741000
6	-4.421395000	-7.194003000	0.581369000
8	-3.350706000	-6.580808000	0.865671000
7	-5.096213000	-6.915615000	-0.552469000
1	-5.082014000	-5.576994000	2.554388000
1	-6.948112000	-7.160527000	1.840730000
1	-6.498920000	-8.242672000	3.150022000
1	-5.503099000	-9.016275000	1.017209000
1	-4.226374000	-8.589102000	2.166918000
1	-4.763870000	-6.128024000	-1.121910000
1	-5.956242000	-7.384799000	-0.788343000
7	-6.517530000	-5.100037000	5.180997000
6	-7.596037000	-4.478119000	5.947663000
6	-8.152337000	-3.163821000	5.391330000
8	-9.312984000	-2.807140000	5.701832000
1	-5.568378000	-5.091713000	5.561485000
1	-8.452239000	-5.155330000	6.010552000
7	-7.318769000	-2.437889000	4.598607000
6	-7.750410000	-1.202071000	3.966921000
6	-7.852075000	-1.278094000	2.443638000
8	-7.881193000	-0.208106000	1.776590000
1	-6.342921000	-2.710965000	4.453247000
1	-8.740471000	-0.954921000	4.366328000
7	-7.948915000	-2.513900000	1.891105000
6	-8.148444000	-2.724017000	0.456254000
6	-6.946065000	-3.326327000	-0.266715000
8	-5.865537000	-2.357073000	-0.357120000
1	-7.960621000	-3.331197000	2.498540000
1	-8.393547000	-1.757473000	0.010913000
1	-6.598220000	-4.231801000	0.248491000
1	-7.254358000	-3.609897000	-1.281495000
1	-5.192063000	-2.616577000	-1.070611000
6	-9.509386000	4.651287000	-3.018979000
6	-8.790844000	3.437979000	-2.466340000
6	-9.148284000	2.892038000	-1.220903000
6	-7.771627000	2.807510000	-3.202329000
6	-8.515661000	1.744598000	-0.726149000
6	-7.133982000	1.661288000	-2.711818000
6	-7.502362000	1.124849000	-1.471546000
1	-10.375631000	4.358385000	-3.628337000
1	-9.883165000	5.297621000	-2.216676000
1	-9.935411000	3.366252000	-0.639004000
1	-7.481082000	3.212392000	-4.168624000
1	-8.795444000	1.322390000	0.233135000
1	-6.350773000	1.190266000	-3.297466000
1	-7.012871000	0.236173000	-1.083850000
26	-0.355705000	-2.937120000	1.046957000
26	-2.059348000	-2.572052000	-1.703093000
8	0.188711000	-5.003632000	0.722439000
1	1.174841000	-5.051378000	0.672675000
1	-0.283857000	-5.615976000	1.421525000
8	-4.496608000	-2.553708000	4.020384000
1	-4.306760000	-1.702218000	4.499271000
1	-4.419656000	-2.391709000	3.015063000
8	-4.216213000	-1.851955000	1.556008000
1	-4.961131000	-2.007554000	0.872261000
1	-4.048648000	-0.884176000	1.700232000
8	-2.188534000	-3.174125000	0.305603000
1	-3.264016000	-4.348400000	-0.408051000
1	-2.888653000	-2.664541000	0.813098000
8	-0.832582000	-4.142261000	8.668339000
1	-0.465878000	-4.767354000	7.955328000
1	-0.816783000	-4.525666000	9.558148000
8	-3.917887000	-3.196362000	-1.904791000
8	-3.899591000	-4.536577000	-1.171993000
8	-0.560520000	-3.957380000	-1.673743000
1	0.113866000	-4.178399000	-2.381303000
8	-1.163631000	-6.353416000	2.356820000
1	-2.006097000	-6.545045000	1.864255000
1	-1.348836000	-6.136130000	3.303639000
8	-2.490142000	-2.796724000	7.290212000
1	-1.908974000	-3.210085000	8.014992000
1	-2.537982000	-1.802824000	7.263513000
8	-1.981902000	-6.128465000	5.014694000
1	-2.312520000	-7.039303000	5.117777000
1	-2.732469000	-5.419848000	5.239884000
8	-0.008109000	-5.565335000	6.678170000
1	-0.740251000	-5.834343000	6.037018000
1	0.712197000	-5.131596000	6.188287000
8	-3.713687000	-4.403688000	5.632449000
1	-4.012822000	-3.757677000	4.899765000
1	-3.365906000	-3.838797000	6.400774000
1	3.717877000	1.143309000	7.012040000
1	3.631124000	4.592386000	3.577229000
1	-0.226714000	6.389358000	5.938345000
1	-1.378095000	7.366634000	1.312383000
1	-5.307490000	5.422560000	2.401499000
1	-5.147100000	7.112606000	-3.776204000
1	-1.946952000	6.314951000	-7.847780000
1	0.745115000	9.822059000	-5.446719000
1	6.879271000	4.308538000	-4.114558000
1	4.065530000	3.811727000	0.194321000
1	8.319259000	-1.685226000	-2.391340000

1	9.864701000	2.038230000	0.312482000
1	6.269714000	0.759821000	3.462720000
1	9.132047000	-2.704067000	4.136841000
1	4.641047000	-1.708245000	-8.103769000
1	7.184439000	-3.611016000	-4.477831000
1	-7.227394000	-4.301637000	6.963276000
1	-7.069885000	-0.381232000	4.210228000
1	-4.821982000	-6.580376000	3.974045000
1	-9.003758000	-3.398476000	0.320199000
1	8.398187000	-4.968796000	0.627209000
1	-8.853614000	5.250712000	-3.660637000
1	2.108158000	-0.118893000	-4.650532000
1	3.888930000	-6.929846000	-3.038689000
1	-0.441530000	-4.533832000	-0.869312000
1	4.545346000	-2.205141000	3.920398000

^{11}IM2$_B$:

6	8.772489000	-3.005139000	3.086527000
6	8.054966000	-1.871483000	2.367434000
8	8.080054000	-1.813338000	1.102683000
6	7.855507000	-4.247080000	3.321839000
6	6.986810000	-4.608202000	2.157546000
7	7.446683000	-4.964025000	0.881173000
6	5.619947000	-4.596045000	2.053811000
6	6.399236000	-5.139843000	0.036572000
7	5.284757000	-4.921959000	0.745361000
1	9.626118000	-3.282203000	2.460441000
1	8.491087000	-5.091599000	3.618181000
1	7.191824000	-4.046155000	4.169306000
1	4.870881000	-4.373645000	2.791868000
1	6.439688000	-5.375400000	-1.028642000
1	4.273881000	-4.824668000	0.374377000
7	7.394734000	-0.968572000	3.134186000
6	6.472361000	0.035564000	2.593860000
6	6.969498000	0.837484000	1.422548000
8	6.162873000	1.206240000	0.521339000
1	7.271224000	-1.113127000	4.136041000
1	5.523421000	-0.410454000	2.283090000
7	8.271594000	1.194960000	1.330327000
6	8.723370000	1.979730000	0.178968000
6	8.307907000	1.374021000	-1.168671000
8	8.034910000	2.123323000	-2.139511000
1	8.916995000	0.938641000	2.062342000
1	8.304140000	2.988926000	0.201436000
7	8.289032000	0.014012000	-1.258660000
6	7.927764000	-0.639943000	-2.510430000
6	6.432712000	-0.663025000	-2.849798000
8	6.076604000	-1.078344000	-3.986367000
1	8.391790000	-0.562360000	-0.422996000
1	8.421192000	-0.134394000	-3.344970000
7	5.574083000	-0.243150000	-1.896607000
6	4.127644000	-0.105027000	-2.132633000
6	3.745416000	1.384977000	-2.146063000
8	3.186227000	1.904501000	-3.155444000
6	3.279062000	-0.885416000	-1.109448000
6	3.501615000	-2.404077000	-1.153112000
6	2.569222000	-3.142269000	-0.201922000
8	1.526038000	-2.528186000	0.191463000
8	2.853022000	-4.366115000	0.156748000
1	5.941967000	0.104665000	-1.012224000
1	3.947335000	-0.477435000	-3.141638000
1	2.222008000	-0.681568000	-1.311368000
1	3.463562000	-0.504960000	-0.097830000
1	4.542813000	-2.660634000	-0.924695000
1	3.290177000	-2.805452000	-2.155108000
7	4.055446000	2.084133000	-1.028679000
6	3.719477000	3.487009000	-0.834820000
6	4.433222000	4.469566000	-1.749075000
8	3.969251000	5.643026000	-1.860345000
1	4.507239000	1.621487000	-0.241036000
1	2.649379000	3.669926000	-0.989451000
7	5.476437000	4.048463000	-2.497515000
6	5.953684000	4.874979000	-3.605731000
6	4.848379000	5.225245000	-4.615872000
8	4.979883000	6.228243000	-5.354135000
1	5.946432000	3.169935000	-2.305553000
1	6.351353000	5.829925000	-3.252795000
7	3.754584000	4.404592000	-4.654401000
6	2.609287000	4.697301000	-5.511778000
6	1.417878000	5.301970000	-4.745442000
8	0.299118000	5.423738000	-5.321259000
6	2.160112000	3.450735000	-6.292872000
1	3.698976000	3.560316000	-4.082272000
1	2.943340000	5.471484000	-6.214442000
1	1.259625000	3.676707000	-6.867735000
1	2.957219000	3.137115000	-6.973668000
1	1.940059000	2.628593000	-5.602832000
7	1.615375000	5.753256000	-3.477714000
6	0.486347000	6.236070000	-2.675313000
6	-0.181239000	7.482912000	-3.271250000
8	-1.378656000	7.751360000	-2.982513000
6	0.895463000	6.506237000	-1.218200000
8	0.786360000	5.254535000	-0.478982000
1	2.522698000	5.656719000	-3.015734000
1	-0.303900000	5.477324000	-2.662197000
1	1.918619000	6.893252000	-1.172490000
1	0.204575000	7.250478000	-0.801960000
1	1.001541000	5.382132000	0.477221000
7	0.571557000	8.257907000	-4.089681000
6	0.017951000	9.415712000	-4.784373000
6	-1.040406000	9.102571000	-5.852081000
8	-1.696765000	10.049922000	-6.340251000
1	1.520113000	7.965725000	-4.283581000
1	-0.468135000	10.085039000	-4.068829000
7	-1.218879000	7.795181000	-6.194863000
6	-2.299887000	7.389399000	-7.081069000
6	-3.506556000	6.771577000	-6.371181000
8	-4.387236000	6.179431000	-7.048062000
1	-0.626769000	7.055921000	-5.816330000
1	-2.645742000	8.274763000	-7.624213000
7	-3.569330000	6.910633000	-5.019349000
6	-4.720674000	6.458216000	-4.239052000
6	-4.372051000	5.383969000	-3.194956000
6	-3.961032000	4.015874000	-3.696301000
6	-4.064131000	3.621653000	-5.058188000
6	-3.481787000	3.072682000	-2.741097000
6	-3.692393000	2.350116000	-5.448074000
6	-3.114953000	1.796107000	-3.103600000
6	-3.197029000	1.398840000	-4.487241000
8	-2.823275000	0.214789000	-4.872422000
1	-2.813050000	7.370396000	-4.519329000
1	-5.472905000	6.104186000	-4.948162000
1	-5.250601000	5.250753000	-2.542246000
1	-3.571691000	5.775050000	-2.548479000
1	-4.420285000	4.323476000	-5.805863000
1	-3.418036000	3.372645000	-1.698946000
1	-3.755082000	2.034641000	-6.483352000
1	-2.756910000	1.065202000	-2.383368000
1	-2.207135000	-1.011653000	-4.053694000
6	-4.701723000	4.529447000	2.587023000
6	-3.235260000	4.836011000	2.420709000
8	-2.342124000	3.959489000	2.587890000
6	-5.218551000	3.507366000	1.518501000
6	-5.971026000	2.324538000	2.155523000
6	-4.986157000	1.408152000	2.891587000
8	-4.062382000	0.823978000	2.260417000
8	-5.184692000	1.373471000	4.195011000
1	-4.818831000	4.080804000	3.581580000
1	-5.881585000	4.019022000	0.812567000
1	-4.377250000	3.101158000	0.946944000
1	-6.456656000	1.709733000	1.392732000
1	-6.749566000	2.669888000	2.840918000
7	-2.861503000	6.114190000	2.094231000
6	-1.476647000	6.393592000	1.699155000
6	-0.440135000	6.010851000	2.754494000
8	0.686291000	5.563559000	2.398386000
1	-3.573252000	6.798278000	1.880494000
1	-1.204157000	5.835699000	0.797190000
7	-0.753823000	6.212328000	4.058828000
6	0.171297000	5.855503000	5.138668000

6	0.268815000	4.345564000	5.425250000
8	1.340935000	3.870805000	5.875119000
1	-1.664614000	6.589195000	4.282306000
1	1.178202000	6.192549000	4.886746000
7	-0.849606000	3.619409000	5.191251000
6	-0.919080000	2.147823000	5.337437000
6	0.054088000	1.507955000	4.324206000
8	0.972345000	0.732639000	4.697190000
6	-2.388153000	1.737333000	5.121624000
6	-2.797663000	0.397517000	5.709522000
8	-2.129055000	-0.158491000	6.632547000
8	-3.904693000	-0.125557000	5.237060000
1	-1.592052000	4.041530000	4.647136000
1	-0.575212000	1.855075000	6.330832000
1	-2.638298000	1.723058000	4.059736000
1	-3.038230000	2.487334000	5.591833000
7	-0.093658000	1.906375000	3.033735000
6	0.798903000	1.423567000	1.972526000
6	2.245552000	1.871888000	2.222492000
8	3.219757000	1.144804000	1.888926000
6	0.310098000	1.903071000	0.584257000
6	-1.066332000	1.367774000	0.116270000
6	-1.115612000	-0.141203000	-0.071039000
8	-0.667460000	-0.849723000	0.893443000
8	-1.614999000	-0.620098000	-1.173377000
1	-0.882834000	2.491104000	2.774657000
1	0.805361000	0.332998000	1.979642000
1	1.072473000	1.604035000	-0.144626000
1	0.272968000	2.999188000	0.562916000
1	-1.854281000	1.626265000	0.837208000
1	-1.312505000	1.856235000	-0.829841000
7	2.426196000	3.094503000	2.796962000
6	3.744731000	3.591907000	3.170299000
6	4.486839000	2.704827000	4.172167000
8	5.731089000	2.540628000	4.049624000
1	1.633024000	3.707931000	2.935803000
1	4.412157000	3.663098000	2.306845000
7	3.766856000	2.133196000	5.166053000
6	4.394525000	1.183913000	6.085767000
6	4.574542000	-0.224637000	5.493836000
8	5.705763000	-0.799100000	5.526446000
1	2.785772000	2.368803000	5.288822000
1	5.390561000	1.536657000	6.357397000
7	3.476049000	-0.809239000	4.968378000
6	3.552146000	-2.150106000	4.372613000
6	2.453306000	-2.401301000	3.334671000
6	1.086579000	-2.600918000	3.914822000
7	-0.030186000	-2.819566000	3.101390000
6	0.665162000	-2.583792000	5.230875000
6	-1.091498000	-2.914802000	3.915861000
7	-0.705799000	-2.781034000	5.206862000
1	2.597872000	-0.290934000	4.888779000
1	3.513401000	-2.911255000	5.165943000
1	2.724288000	-3.291155000	2.749376000
1	2.437599000	-1.568462000	2.620765000
1	1.191469000	-2.383016000	6.147810000
1	-2.112301000	-3.054668000	3.606056000
6	1.549026000	-1.094481000	-4.905406000
6	2.393148000	-2.078652000	-5.688667000
8	2.220824000	-3.331309000	-5.603105000
6	0.247407000	-0.673905000	-5.644390000
6	-0.744723000	-1.816812000	-5.973263000
6	-1.182817000	-2.619363000	-4.761829000
8	-1.828125000	-1.910650000	-3.761792000
8	-0.956493000	-3.818041000	-4.613852000
1	1.290444000	-1.565255000	-3.950761000
1	-0.255810000	0.072994000	-5.022255000
1	0.508240000	-0.171759000	-6.584881000
1	-0.307456000	-2.529650000	-6.676089000
1	-1.629663000	-1.365751000	-6.436939000
7	3.336458000	-1.572928000	-6.529217000
6	4.066152000	-2.418930000	-7.479637000
6	4.970411000	-3.496168000	-6.869529000
8	5.219846000	-4.529666000	-7.529379000
1	3.492857000	-0.575305000	-6.543953000
1	3.363283000	-2.956857000	-8.121651000
7	5.471890000	-3.250967000	-5.627785000
6	6.308866000	-4.216530000	-4.932982000
6	5.588438000	-4.980060000	-3.819734000
8	6.254719000	-5.460982000	-2.854328000
1	5.371223000	-2.344133000	-5.176163000
1	6.673589000	-4.944841000	-5.666521000
7	4.259898000	-5.148161000	-3.956757000
6	3.457353000	-5.958338000	-3.029683000
6	1.975171000	-5.927088000	-3.413134000
8	1.490532000	-4.546115000	-3.285742000
6	1.131825000	-6.825973000	-2.514510000
1	3.781154000	-4.667656000	-4.713681000
1	3.555244000	-5.572242000	-2.009748000
1	1.857698000	-6.235494000	-4.461239000
1	1.480082000	-4.100428000	-4.170828000
1	0.081259000	-6.770699000	-2.812320000
1	1.460021000	-7.868873000	-2.589430000
1	1.207947000	-6.509921000	-1.469429000
6	-5.669898000	-6.262978000	3.332819000
6	-6.789625000	-5.482080000	4.006203000
8	-7.884036000	-5.275538000	3.408638000
6	-6.239770000	-7.490080000	2.591264000
6	-5.193743000	-8.254388000	1.741495000
6	-4.477678000	-7.325792000	0.774515000
8	-3.312807000	-6.903285000	1.020015000
7	-5.160702000	-6.900705000	-0.308463000
1	-5.174920000	-5.593128000	2.616703000
1	-7.065308000	-7.145224000	1.959266000
1	-6.677218000	-8.187171000	3.317606000
1	-5.694742000	-9.064958000	1.196590000
1	-4.431478000	-8.706542000	2.383176000
1	-4.760715000	-6.127742000	-0.862472000
1	-6.092877000	-7.230098000	-0.504761000
7	-6.553306000	-5.023888000	5.264202000
6	-7.607903000	-4.370402000	6.038216000
6	-8.158886000	-3.061020000	5.464538000
8	-9.314794000	-2.689989000	5.777248000
1	-5.597795000	-5.028999000	5.629732000
1	-8.471672000	-5.034207000	6.133342000
7	-7.325098000	-2.356052000	4.654292000
6	-7.750427000	-1.130722000	3.998320000
6	-7.842606000	-1.237304000	2.475580000
8	-7.837681000	-0.181730000	1.784211000
1	-6.350950000	-2.638913000	4.509888000
1	-8.742113000	-0.874377000	4.388045000
7	-7.971093000	-2.482844000	1.954590000
6	-8.159989000	-2.734855000	0.524982000
6	-6.963881000	-3.393188000	-0.161214000
8	-5.858403000	-2.461150000	-0.282433000
1	-8.000535000	-3.283651000	2.583531000
1	-8.376923000	-1.779203000	0.043400000
1	-6.648619000	-4.286050000	0.396649000
1	-7.271064000	-3.712715000	-1.166167000
1	-5.171875000	-2.769566000	-0.989701000
6	-9.067792000	4.058857000	-3.658816000
6	-8.401701000	2.874195000	-2.989902000
6	-8.724588000	2.514011000	-1.669488000
6	-7.471133000	2.083371000	-3.687102000
6	-8.142349000	1.394474000	-1.061316000
6	-6.885098000	0.963835000	-3.083740000
6	-7.214516000	0.615380000	-1.767801000
1	-9.986138000	3.757891000	-4.181815000
1	-9.350014000	4.826271000	-2.928971000
1	-9.447735000	3.110764000	-1.117778000
1	-7.209808000	2.344256000	-4.709680000
1	-8.403359000	1.111314000	-0.046493000
1	-6.174907000	0.360276000	-3.641828000
1	-6.760489000	-0.250312000	-1.293466000
26	-0.362804000	-2.947610000	1.000986000
26	-2.140532000	-2.600188000	-1.655215000
8	0.190953000	-5.030557000	0.730696000
1	1.175209000	-5.084479000	0.677333000
1	-0.271560000	-5.637258000	1.439275000
8	-4.518900000	-2.513167000	4.088901000

1	-4.323508000	-1.663289000	4.565203000
1	-4.452258000	-2.350734000	3.078605000
8	-4.270053000	-1.827963000	1.629577000
1	-4.994810000	-2.032024000	0.932753000
1	-4.102204000	-0.857033000	1.727329000
8	-2.233669000	-3.202923000	0.416041000
1	-3.225129000	-4.353200000	-0.313166000
1	-2.914726000	-2.672073000	0.917723000
8	-0.757335000	-4.116154000	8.638060000
1	-0.413341000	-4.753489000	7.922904000
1	-0.725559000	-4.491863000	9.530803000
8	-4.002398000	-3.319210000	-1.841174000
8	-3.834280000	-4.641461000	-1.074144000
8	-0.561357000	-3.945195000	-1.648809000
1	0.101028000	-4.163266000	-2.361103000
8	-1.099357000	-6.411096000	2.397878000
1	-1.921900000	-6.705459000	1.926584000
1	-1.313223000	-6.139282000	3.323331000
8	-2.460329000	-2.786118000	7.307910000
1	-1.858058000	-3.195694000	8.017591000
1	-2.505397000	-1.792895000	7.281320000
8	-2.005655000	-6.101278000	5.029035000
1	-2.343103000	-7.011509000	5.112372000
1	-2.752994000	-5.394008000	5.258841000
8	0.005508000	-5.559296000	6.644000000
1	-0.746974000	-5.824707000	6.023822000
1	0.711809000	-5.127881000	6.131833000
8	-3.732246000	-4.378007000	5.673003000
1	-4.040872000	-3.721960000	4.951756000
1	-3.368347000	-3.821450000	6.438940000
1	3.774379000	1.123171000	6.985000000
1	3.613469000	4.593322000	3.590793000
1	-0.145345000	6.371741000	6.049887000
1	-1.394066000	7.463657000	1.482159000
1	-5.301388000	5.446297000	2.571935000
1	-5.149254000	7.318463000	-3.709390000
1	-1.942955000	6.657785000	-7.810539000
1	0.837048000	9.965681000	-5.255007000
1	6.758567000	4.331874000	-4.108332000
1	3.939396000	3.742251000	0.204288000
1	8.283871000	-1.673850000	-2.482199000
1	9.814223000	2.055756000	0.221014000
1	6.254171000	0.750762000	3.395158000
1	9.163827000	-2.677063000	4.055490000
1	4.673172000	-1.764893000	-8.113132000
1	7.166002000	-3.711195000	-4.483689000
1	-7.215737000	-4.177434000	7.042034000
1	-7.069212000	-0.307337000	4.230111000
1	-4.907479000	-6.560446000	4.061480000
1	-9.031127000	-3.391797000	0.402816000
1	8.412799000	-5.001503000	0.596150000
1	-8.411894000	4.522867000	-4.404449000
1	2.120883000	-0.186842000	-4.675127000
1	3.829456000	-6.990532000	-3.039470000
1	-0.455896000	-4.555268000	-0.870484000
1	4.536765000	-2.239070000	3.901947000

^{11}TS3$_B$:

6	8.615394000	-2.751054000	2.475686000
6	7.842055000	-1.594555000	1.858917000
8	7.765566000	-1.501515000	0.598461000
6	7.771764000	-4.063804000	2.581915000
6	6.849808000	-4.315205000	1.429876000
7	7.240636000	-4.551270000	0.103611000
6	5.480310000	-4.284931000	1.398472000
6	6.150888000	-4.638434000	-0.700122000
7	5.077277000	-4.482205000	0.084285000
1	9.481716000	-2.921568000	1.828751000
1	8.462610000	-4.904056000	2.726749000
1	7.151422000	-4.016525000	3.482936000
1	4.771689000	-4.129025000	2.191536000
1	6.122933000	-4.777081000	-1.783491000
1	4.053438000	-4.361740000	-0.212650000
7	7.255287000	-0.704058000	2.696860000
6	6.327610000	0.339863000	2.243662000
6	6.787033000	1.174762000	1.077437000
8	5.943414000	1.621444000	0.248704000
1	7.205281000	-0.874183000	3.700147000
1	5.355837000	-0.075086000	1.961200000
7	8.098971000	1.468416000	0.916626000
6	8.527026000	2.259622000	-0.238653000
6	8.011150000	1.711538000	-1.576740000
8	7.738733000	2.503840000	-2.513650000
1	8.772934000	1.145306000	1.594316000
1	8.161156000	3.287242000	-0.169446000
7	7.898712000	0.359978000	-1.695845000
6	7.435972000	-0.245361000	-2.939522000
6	5.921713000	-0.234521000	-3.180354000
8	5.483910000	-0.680232000	-4.273154000
1	8.014860000	-0.243015000	-0.880943000
1	7.881944000	0.281386000	-3.787861000
7	5.133365000	0.256354000	-2.196551000
6	3.684731000	0.461184000	-2.376011000
6	3.383804000	1.965695000	-2.274546000
8	2.873501000	2.602201000	-3.239733000
6	2.833787000	-0.366213000	-1.387486000
6	3.060764000	-1.880609000	-1.503644000
6	2.232127000	-2.681626000	-0.507067000
8	1.205986000	-2.126830000	0.011542000
8	2.591562000	-3.906742000	-0.231627000
1	5.559967000	0.600875000	-1.337905000
1	3.458172000	0.176501000	-3.405141000
1	1.776831000	-0.150864000	-1.580472000
1	3.027378000	-0.042340000	-0.358576000
1	4.120112000	-2.127083000	-1.367208000
1	2.790939000	-2.241509000	-2.507481000
7	3.721747000	2.569559000	-1.109459000
6	3.472046000	3.983403000	-0.871520000
6	4.296268000	4.952512000	-1.704723000
8	3.915484000	6.158725000	-1.783317000
1	4.160661000	2.045998000	-0.354486000
1	2.424751000	4.235633000	-1.074581000
7	5.363752000	4.494446000	-2.391837000
6	6.032044000	5.338828000	-3.380459000
6	5.093697000	5.850358000	-4.481184000
8	5.406429000	6.872256000	-5.133458000
1	5.729062000	3.560483000	-2.243740000
1	6.461757000	6.230733000	-2.916500000
7	3.936552000	5.151873000	-4.691411000
6	2.929180000	5.613820000	-5.644362000
6	1.636892000	6.070059000	-4.943674000
8	0.557819000	6.164116000	-5.592065000
6	2.634106000	4.560603000	-6.723894000
1	3.744452000	4.284619000	-4.192916000
1	3.362803000	6.504970000	-6.118532000
1	1.822469000	4.901195000	-7.370341000
1	3.532240000	4.396766000	-7.326232000
1	2.338116000	3.607055000	-6.275451000
7	1.706637000	6.433658000	-3.629614000
6	0.479746000	6.702326000	-2.869789000
6	-0.302396000	7.922749000	-3.368831000
8	-1.528811000	8.021266000	-3.093080000
6	0.740395000	6.818435000	-1.357165000
8	0.515068000	5.503411000	-0.755034000
1	2.581596000	6.341157000	-3.108679000
1	-0.214616000	5.868318000	-3.013647000
1	1.765221000	7.152403000	-1.166695000
1	0.032861000	7.541283000	-0.933483000
1	0.660702000	5.535061000	0.222796000
7	0.363319000	8.848306000	-4.099432000
6	-0.327639000	9.976198000	-4.718466000
6	-1.353503000	9.604945000	-5.800651000
8	-2.170664000	10.476512000	-6.174632000
1	1.343023000	8.689679000	-4.291080000
1	-0.883177000	10.539360000	-3.963779000
7	-1.314788000	8.333713000	-6.289271000
6	-2.327170000	7.844670000	-7.216024000
6	-3.232960000	6.757893000	-6.634510000
8	-3.890522000	6.015085000	-7.406934000
1	-0.598793000	7.670382000	-5.991773000

1	-2.943476000	8.699228000	-7.514660000
7	-3.288364000	6.664555000	-5.276516000
6	-4.106294000	5.658406000	-4.604241000
6	-3.274924000	4.632080000	-3.807066000
6	-2.262922000	3.819474000	-4.587990000
6	-2.448811000	3.479744000	-5.955893000
6	-1.095203000	3.353196000	-3.921489000
6	-1.528584000	2.691545000	-6.618232000
6	-0.157930000	2.571607000	-4.565697000
6	-0.356527000	2.189696000	-5.943620000
8	0.481959000	1.413373000	-6.559506000
1	-2.734466000	7.291186000	-4.699064000
1	-4.711850000	5.175384000	-5.375075000
1	-3.982297000	3.939463000	-3.319455000
1	-2.754255000	5.157199000	-2.995256000
1	-3.298622000	3.875399000	-6.500994000
1	-0.932153000	3.638511000	-2.885301000
1	-1.658044000	2.428720000	-7.662226000
1	0.758589000	2.261553000	-4.074814000
1	-3.767966000	-3.466518000	-2.861420000
6	-4.988864000	4.360160000	2.438804000
6	-3.541800000	4.720918000	2.215450000
8	-2.607690000	3.887374000	2.384557000
6	-5.497923000	3.257807000	1.451675000
6	-6.217082000	2.109272000	2.184197000
6	-5.200554000	1.232931000	2.924375000
8	-4.312148000	0.603050000	2.287556000
8	-5.334552000	1.276439000	4.236457000
1	-5.056391000	3.967396000	3.461745000
1	-6.182335000	3.703381000	0.722291000
1	-4.660185000	2.831158000	0.890955000
1	-6.730862000	1.454192000	1.475958000
1	-6.967989000	2.488789000	2.882514000
7	-3.234142000	6.005775000	1.852506000
6	-1.869880000	6.353359000	1.439360000
6	-0.807811000	6.023379000	2.487410000
8	0.329166000	5.605305000	2.128680000
1	-3.982037000	6.648016000	1.631891000
1	-1.578983000	5.819913000	0.528603000
7	-1.110051000	6.245165000	3.791671000
6	-0.152748000	5.959281000	4.864910000
6	0.020668000	4.463582000	5.186848000
8	1.128010000	4.044932000	5.606191000
1	-2.028293000	6.600440000	4.019988000
1	0.833540000	6.336226000	4.588874000
7	-1.073438000	3.684328000	5.014297000
6	-1.072312000	2.214731000	5.192646000
6	-0.099468000	1.595442000	4.165933000
8	0.849136000	0.849360000	4.523458000
6	-2.526478000	1.735048000	5.021877000
6	-2.871201000	0.407952000	5.676658000
8	-2.145186000	-0.096445000	6.585820000
8	-3.983139000	-0.161521000	5.273881000
1	-1.856388000	4.064563000	4.497369000
1	-0.692368000	1.957707000	6.182757000
1	-2.792371000	1.661773000	3.965756000
1	-3.201707000	2.477292000	5.467794000
7	-0.289173000	1.979329000	2.876513000
6	0.594595000	1.519055000	1.800337000
6	2.036579000	2.001779000	2.033415000
8	3.021621000	1.289793000	1.696495000
6	0.068340000	1.984330000	0.419639000
6	-1.314330000	1.427290000	-0.016995000
6	-1.378644000	-0.085089000	-0.088120000
8	-1.076532000	-0.739079000	0.963246000
8	-1.751631000	-0.643302000	-1.212255000
1	-1.102024000	2.537279000	2.631034000
1	0.634758000	0.429065000	1.814183000
1	0.819221000	1.698129000	-0.324210000
1	0.010977000	3.079881000	0.399691000
1	-2.093935000	1.744991000	0.687219000
1	-1.562999000	1.839915000	-0.998199000
7	2.194550000	3.227952000	2.602070000
6	3.500441000	3.764605000	2.970813000
6	4.288499000	2.898775000	3.954148000
8	5.533031000	2.770623000	3.801575000
1	1.385694000	3.819451000	2.746082000
1	4.158077000	3.867120000	2.103918000
7	3.612425000	2.306373000	4.966882000
6	4.302258000	1.375173000	5.860501000
6	4.538739000	-0.008632000	5.232727000
8	5.691272000	-0.535835000	5.229681000
1	2.626872000	2.507173000	5.111919000
1	5.284437000	1.771654000	6.122015000
7	3.455237000	-0.626759000	4.711247000
6	3.576618000	-1.949790000	4.086014000
6	2.419879000	-2.243044000	3.127309000
6	1.103770000	-2.466966000	3.805649000
7	-0.070102000	-2.666233000	3.072854000
6	0.777585000	-2.474277000	5.148191000
6	-1.071529000	-2.771607000	3.959536000
7	-0.592056000	-2.667313000	5.219254000
1	2.558020000	-0.140634000	4.653146000
1	3.641096000	-2.723647000	4.865242000
1	2.672532000	-3.127961000	2.526032000
1	2.332458000	-1.413459000	2.414820000
1	1.370436000	-2.288296000	6.026718000
1	-2.113783000	-2.898361000	3.723185000
6	0.030113000	-1.448699000	-4.751658000
6	1.061257000	-2.016478000	-5.702177000
8	1.249597000	-3.277934000	-5.796648000
6	-1.372603000	-1.320629000	-5.411895000
6	-2.011381000	-2.640286000	-5.868035000
6	-2.637678000	-3.527977000	-4.798801000
8	-2.950984000	-2.919109000	-3.623681000
8	-2.923776000	-4.710853000	-5.030915000
1	-0.026450000	-2.104026000	-3.877387000
1	-2.033905000	-0.832150000	-4.689291000
1	-1.280473000	-0.652333000	-6.275739000
1	-1.308768000	-3.260473000	-6.434540000
1	-2.832116000	-2.405665000	-6.562335000
7	1.750066000	-1.155535000	-6.479050000
6	2.596308000	-1.615217000	-7.589810000
6	3.654188000	-2.666299000	-7.235177000
8	3.892083000	-3.601089000	-8.031736000
1	1.494385000	-0.163689000	-6.442026000
1	1.987488000	-2.073233000	-8.374967000
7	4.319568000	-2.505290000	-6.053535000
6	5.318968000	-3.460087000	-5.595434000
6	4.845872000	-4.330075000	-4.429338000
8	5.676468000	-4.750723000	-3.566981000
1	4.250778000	-1.650057000	-5.511445000
1	5.570430000	-4.113538000	-6.438924000
7	3.537624000	-4.655753000	-4.409026000
6	2.963374000	-5.575427000	-3.416548000
6	1.530897000	-5.987716000	-3.784492000
8	0.658236000	-4.816375000	-3.760638000
6	0.964928000	-7.008475000	-2.801393000
1	2.915908000	-4.222287000	-5.090570000
1	2.938761000	-5.104212000	-2.426348000
1	1.517406000	-6.401967000	-4.802148000
1	0.729619000	-4.268998000	-4.596540000
1	-0.069069000	-7.242355000	-3.068079000
1	1.550489000	-7.934312000	-2.819084000
1	0.973367000	-6.610926000	-1.780149000
6	-5.435364000	-6.314460000	3.505144000
6	-6.593155000	-5.608557000	4.195231000
8	-7.709386000	-5.482974000	3.615100000
6	-5.914230000	-7.629583000	2.855632000
6	-4.842348000	-8.317987000	1.971601000
6	-4.313557000	-7.371669000	0.909378000
8	-3.215291000	-6.762778000	1.058251000
7	-5.097594000	-7.129139000	-0.161877000
1	-5.045748000	-5.639245000	2.731887000
1	-6.808546000	-7.406589000	2.263480000
1	-6.225601000	-8.334046000	3.637574000
1	-5.276708000	-9.212699000	1.507803000
1	-3.990448000	-8.638596000	2.578845000
1	-4.808619000	-6.380470000	-0.799572000
1	-5.971680000	-7.610004000	-0.302367000

7	-6.365009000	-5.127438000	5.445301000
6	-7.443441000	-4.538021000	6.237795000
6	-8.086401000	-3.267450000	5.671865000
8	-9.251650000	-2.962025000	6.017142000
1	-5.404722000	-5.078338000	5.793912000
1	-8.262031000	-5.254141000	6.351509000
7	-7.321795000	-2.522466000	4.828335000
6	-7.841557000	-1.329366000	4.179296000
6	-8.029535000	-1.470558000	2.667492000
8	-8.118180000	-0.433939000	1.954456000
1	-6.339385000	-2.753939000	4.655779000
1	-8.816890000	-1.108339000	4.627484000
7	-8.136646000	-2.734336000	2.182564000
6	-8.406310000	-3.022516000	0.775306000
6	-7.252551000	-3.716384000	0.056392000
8	-6.133404000	-2.800825000	-0.099605000
1	-8.079647000	-3.522733000	2.825297000
1	-8.629901000	-2.075573000	0.279447000
1	-6.936139000	-4.606771000	0.616660000
1	-7.595951000	-4.035815000	-0.936374000
1	-5.501450000	-3.077559000	-0.833697000
6	-8.209443000	4.107719000	-3.427593000
6	-7.690466000	2.828641000	-2.804553000
6	-8.281409000	2.299017000	-1.643628000
6	-6.628421000	2.119238000	-3.394429000
6	-7.835369000	1.090977000	-1.091026000
6	-6.175803000	0.913055000	-2.845107000
6	-6.777989000	0.393829000	-1.691938000
1	-8.955666000	3.897598000	-4.206032000
1	-8.691780000	4.750067000	-2.682480000
1	-9.106704000	2.831889000	-1.176991000
1	-6.166187000	2.506575000	-4.299756000
1	-8.298320000	0.687254000	-0.195030000
1	-5.358818000	0.376215000	-3.319262000
1	-6.441960000	-0.544784000	-1.261992000
26	-0.520703000	-2.808551000	1.024132000
26	-2.192504000	-2.550356000	-1.576940000
8	0.169851000	-4.812795000	0.636708000
1	1.146219000	-4.779406000	0.479771000
1	-0.198120000	-5.458473000	1.367299000
8	-4.508389000	-2.577426000	4.176134000
1	-4.342892000	-1.698227000	4.612382000
1	-4.492646000	-2.466154000	3.162107000
8	-4.414579000	-2.020916000	1.657847000
1	-5.193683000	-2.275009000	1.054249000
1	-4.297882000	-1.037830000	1.736458000
8	-2.350978000	-3.246910000	0.387920000
1	-3.239641000	-4.507034000	-0.353533000
1	-3.083106000	-2.791860000	0.900980000
8	-0.581389000	-3.983969000	8.692170000
1	-0.224427000	-4.613871000	7.979204000
1	-0.536375000	-4.354059000	9.586596000
8	-4.222212000	-3.509917000	-1.743856000
8	-3.848977000	-4.838098000	-1.101966000
8	-0.740087000	-3.970262000	-1.755763000
1	-0.256884000	-4.326097000	-2.567192000
8	-0.955441000	-6.256008000	2.364700000
1	-1.797870000	-6.554188000	1.929831000
1	-1.126614000	-6.003899000	3.305010000
8	-2.300348000	-2.709761000	7.339484000
1	-1.696462000	-3.087236000	8.065463000
1	-2.403669000	-1.720983000	7.298818000
8	-1.769012000	-6.000963000	5.042917000
1	-2.061919000	-6.925147000	5.143249000
1	-2.540025000	-5.329375000	5.303790000
8	0.209775000	-5.414930000	6.694256000
1	-0.525458000	-5.685640000	6.057367000
1	0.942757000	-5.014338000	6.195932000
8	-3.553471000	-4.365400000	5.759728000
1	-3.928713000	-3.734982000	5.048586000
1	-3.186011000	-3.784957000	6.507236000
1	3.701436000	1.266126000	6.768120000
1	3.337494000	4.755913000	3.403549000
1	-0.480306000	6.483595000	5.767650000
1	-1.844821000	7.427163000	1.225598000
1	-5.628016000	5.248912000	2.391996000
1	-4.795143000	6.153783000	-3.908431000
1	-1.867826000	7.425944000	-8.115765000
1	0.419361000	10.644307000	-5.155753000
1	6.843730000	4.752631000	-3.818710000
1	3.657682000	4.187683000	0.184394000
1	7.771244000	-1.285691000	-2.973567000
1	9.620987000	2.282309000	-0.254537000
1	6.157489000	1.025262000	3.081899000
1	8.990617000	-2.499128000	3.473046000
1	3.088308000	-0.731610000	-8.008851000
1	6.214300000	-2.931055000	-5.268650000
1	-7.045712000	-4.317249000	7.233529000
1	-7.183899000	-0.473877000	4.355003000
1	-4.612799000	-6.498333000	4.204322000
1	-9.291578000	-3.668631000	0.708710000
1	8.190947000	-4.562153000	-0.232197000
1	-7.405819000	4.682785000	-3.902090000
1	0.342114000	-0.457269000	-4.409069000
1	3.604334000	-6.462836000	-3.352653000
1	-0.513226000	-4.478359000	-0.927677000
1	4.526982000	-1.975189000	3.542425000

$^{11}Pr_B$:

6	8.624518000	-2.663661000	2.301934000
6	7.856080000	-1.513365000	1.666133000
8	7.770363000	-1.449321000	0.403388000
6	7.776226000	-3.973564000	2.417117000
6	6.829214000	-4.200940000	1.280874000
7	7.194449000	-4.363523000	-0.062897000
6	5.459014000	-4.186450000	1.280136000
6	6.090934000	-4.414429000	-0.848156000
7	5.031199000	-4.315828000	-0.035115000
1	9.494640000	-2.843849000	1.662565000
1	8.464108000	-4.819928000	2.537901000
1	7.175487000	-3.929943000	3.331307000
1	4.765559000	-4.080031000	2.094551000
1	6.051416000	-4.473085000	-1.938710000
1	4.005701000	-4.182699000	-0.307921000
7	7.288478000	-0.601914000	2.492359000
6	6.363339000	0.444672000	2.039258000
6	6.793469000	1.243110000	0.836452000
8	5.921868000	1.710766000	0.047899000
1	7.239376000	-0.762526000	3.499097000
1	5.375480000	0.038300000	1.803558000
7	8.103952000	1.484875000	0.600679000
6	8.503621000	2.228036000	-0.595818000
6	7.890295000	1.684439000	-1.895023000
8	7.605386000	2.477894000	-2.826866000
1	8.801260000	1.129637000	1.237134000
1	8.193721000	3.274203000	-0.530646000
7	7.704393000	0.338441000	-1.989649000
6	7.148379000	-0.253921000	-3.203630000
6	5.633893000	-0.106050000	-3.399496000
8	5.118335000	-0.491704000	-4.481341000
1	7.851312000	-0.260509000	-1.176611000
1	7.616699000	0.200289000	-4.081107000
7	4.923722000	0.414169000	-2.372029000
6	3.475286000	0.666204000	-2.463237000
6	3.218379000	2.171595000	-2.288854000
8	2.685105000	2.864170000	-3.201909000
6	2.670113000	-0.172471000	-1.447477000
6	2.889342000	-1.684630000	-1.594697000
6	2.172188000	-2.483000000	-0.518572000
8	1.222331000	-1.916802000	0.119957000
8	2.540991000	-3.715923000	-0.294143000
1	5.410558000	0.719252000	-1.531103000
1	3.184110000	0.417718000	-3.485320000
1	1.605338000	0.050226000	-1.578793000
1	2.922863000	0.131826000	-0.425068000
1	3.957413000	-1.930841000	-1.571684000
1	2.512856000	-2.046035000	-2.563418000
7	3.628185000	2.706697000	-1.112880000
6	3.459333000	4.114859000	-0.787569000
6	4.286202000	5.083485000	-1.618827000

8	3.955516000	6.306598000	-1.639800000
1	4.092081000	2.135436000	-0.409485000
1	2.415900000	4.426995000	-0.907700000
7	5.306635000	4.605130000	-2.362916000
6	5.975669000	5.450628000	-3.349932000
6	5.018640000	6.044752000	-4.390938000
8	5.346052000	7.080318000	-5.013558000
1	5.620450000	3.646454000	-2.267771000
1	6.466540000	6.304305000	-2.874894000
7	3.826697000	5.401478000	-4.580876000
6	2.796823000	5.948015000	-5.462403000
6	1.523764000	6.315368000	-4.680672000
8	0.405031000	6.373028000	-5.262683000
6	2.479490000	5.010122000	-6.636337000
1	3.617489000	4.522022000	-4.111194000
1	3.217736000	6.884403000	-5.854792000
1	1.665352000	5.418485000	-7.238765000
1	3.370376000	4.894569000	-7.259867000
1	2.174210000	4.023282000	-6.275283000
7	1.650336000	6.644019000	-3.361942000
6	0.455768000	6.843098000	-2.529761000
6	-0.441543000	7.984838000	-3.020442000
8	-1.673298000	7.961855000	-2.760177000
6	0.812794000	7.042587000	-1.042103000
8	0.633582000	5.758365000	-0.366594000
1	2.553257000	6.556066000	-2.889681000
1	-0.182070000	5.954238000	-2.584708000
1	1.845889000	7.388450000	-0.936737000
1	0.133006000	7.785168000	-0.606030000
1	0.838358000	5.821532000	0.598478000
7	0.145886000	8.999074000	-3.703460000
6	-0.634337000	10.102531000	-4.257498000
6	-1.621764000	9.730080000	-5.374127000
8	-2.489876000	10.568886000	-5.706490000
1	1.134299000	8.930760000	-3.903634000
1	-1.238017000	10.566090000	-3.472407000
7	-1.493285000	8.497758000	-5.939095000
6	-2.458484000	8.001090000	-6.911419000
6	-3.250868000	6.782271000	-6.436013000
8	-3.863624000	6.072804000	-7.274022000
1	-0.747905000	7.860265000	-5.658964000
1	-3.154765000	8.816786000	-7.132509000
7	-3.258558000	6.540095000	-5.096508000
6	-3.957773000	5.396586000	-4.517383000
6	-3.011642000	4.323369000	-3.941153000
6	-2.080903000	3.619622000	-4.905708000
6	-2.425682000	3.375427000	-6.264036000
6	-0.840967000	3.118826000	-4.418538000
6	-1.605705000	2.614573000	-7.074367000
6	-0.006691000	2.358029000	-5.209701000
6	-0.382151000	2.040443000	-6.567810000
8	0.336993000	1.253597000	-7.308379000
1	-2.755260000	7.157368000	-4.466085000
1	-4.603199000	4.985082000	-5.296367000
1	-3.643224000	3.555550000	-3.461626000
1	-2.416328000	4.773362000	-3.135799000
1	-3.320893000	3.822629000	-6.681264000
1	-0.544231000	3.343688000	-3.398626000
1	-1.861613000	2.414850000	-8.108927000
1	0.948881000	2.011329000	-4.831258000
1	-3.772038000	-4.722763000	-2.476070000
6	-4.774575000	4.434906000	2.703050000
6	-3.331602000	4.840322000	2.540683000
8	-2.387837000	4.008039000	2.649783000
6	-5.241682000	3.436785000	1.592531000
6	-6.022461000	2.240226000	2.176002000
6	-5.066091000	1.311510000	2.936516000
8	-4.263078000	0.565804000	2.314781000
8	-5.141263000	1.447362000	4.248112000
1	-4.850774000	3.928718000	3.673973000
1	-5.873790000	3.963161000	0.869095000
1	-4.377065000	3.048433000	1.044124000
1	-6.477518000	1.639589000	1.383808000
1	-6.821113000	2.584353000	2.839524000
7	-3.039651000	6.155587000	2.291920000
6	-1.680687000	6.552006000	1.903923000
6	-0.608142000	6.163620000	2.920713000
8	0.532511000	5.791235000	2.526173000
1	-3.796276000	6.801333000	2.115550000
1	-1.386396000	6.087419000	0.956942000
7	-0.906343000	6.284028000	4.239898000
6	0.059931000	5.928332000	5.284516000
6	0.229243000	4.413352000	5.505845000
8	1.344661000	3.956003000	5.856836000
1	-1.826747000	6.612853000	4.497449000
1	1.044704000	6.318672000	5.022653000
7	-0.881284000	3.660046000	5.322112000
6	-0.904392000	2.180730000	5.396401000
6	0.073344000	1.620306000	4.340233000
8	1.004977000	0.833943000	4.649573000
6	-2.364858000	1.743449000	5.164094000
6	-2.770948000	0.400792000	5.747695000
8	-2.084862000	-0.177772000	6.641653000
8	-3.904244000	-0.096390000	5.303623000
1	-1.677474000	4.090719000	4.870581000
1	-0.546900000	1.845376000	6.371640000
1	-2.603504000	1.728063000	4.099588000
1	-3.036704000	2.481100000	5.622646000
7	-0.107315000	2.095732000	3.081240000
6	0.746001000	1.682740000	1.963913000
6	2.193086000	2.154891000	2.164539000
8	3.164883000	1.465235000	1.750989000
6	0.172661000	2.214149000	0.628573000
6	-1.229268000	1.672379000	0.245652000
6	-1.275274000	0.166044000	0.100784000
8	-1.060430000	-0.545896000	1.139787000
8	-1.528330000	-0.326846000	-1.080159000
1	-0.910304000	2.684808000	2.878596000
1	0.785120000	0.592525000	1.929937000
1	0.886064000	1.958625000	-0.161214000
1	0.118802000	3.309467000	0.659877000
1	-1.966548000	1.948660000	1.007521000
1	-1.531723000	2.126939000	-0.701100000
7	2.370400000	3.352588000	2.785063000
6	3.690892000	3.867988000	3.128360000
6	4.501404000	2.941282000	4.035320000
8	5.738001000	2.804929000	3.836432000
1	1.568176000	3.933879000	2.992605000
1	4.315056000	4.013979000	2.242749000
7	3.844498000	2.302308000	5.033289000
6	4.542248000	1.314997000	5.857767000
6	4.708506000	-0.052039000	5.172500000
8	5.854211000	-0.582376000	5.051584000
1	2.866115000	2.508166000	5.216388000
1	5.546956000	1.673138000	6.086557000
7	3.578733000	-0.655894000	4.740429000
6	3.636347000	-1.967789000	4.081676000
6	2.441734000	-2.211243000	3.155160000
6	1.146969000	-2.476415000	3.861347000
7	-0.049332000	-2.635158000	3.151307000
6	0.859746000	-2.586715000	5.206529000
6	-1.021662000	-2.822380000	4.058767000
7	-0.503366000	-2.806842000	5.305865000
1	2.682859000	-0.163957000	4.764620000
1	3.706997000	-2.761645000	4.839592000
1	2.674521000	-3.064628000	2.502184000
1	2.332057000	-1.349323000	2.486259000
1	1.485609000	-2.487919000	6.075721000
1	-2.070733000	-2.939730000	3.848503000
6	-0.231770000	-1.527101000	-5.424484000
6	0.884122000	-2.139397000	-6.238758000
8	1.174715000	-3.383355000	-6.147377000
6	-1.639367000	-1.782244000	-6.048334000
6	-2.247278000	-3.163421000	-5.750553000
6	-2.736383000	-3.351240000	-4.317971000
8	-2.562567000	-2.337770000	-3.495959000
8	-3.274325000	-4.444901000	-3.978015000
1	-0.205835000	-1.941757000	-4.413741000
1	-2.311104000	-1.012051000	-5.657955000
1	-1.578619000	-1.635659000	-7.134182000

1	-1.530860000	-3.967986000	-5.961523000
1	-3.104534000	-3.347875000	-6.410932000
7	1.543982000	-1.333914000	-7.095951000
6	2.460744000	-1.853776000	-8.117654000
6	3.625305000	-2.714731000	-7.621344000
8	4.017147000	-3.678204000	-8.317711000
1	1.251642000	-0.351594000	-7.158819000
1	1.922629000	-2.485895000	-8.830196000
7	4.214499000	-2.365299000	-6.440143000
6	5.308708000	-3.141775000	-5.874925000
6	4.904721000	-3.945347000	-4.640565000
8	5.758779000	-4.202378000	-3.737214000
1	3.982044000	-1.507680000	-5.952495000
1	5.655743000	-3.837138000	-6.647748000
7	3.633967000	-4.395189000	-4.603316000
6	3.131995000	-5.270403000	-3.537253000
6	1.713384000	-5.772340000	-3.836869000
8	0.789637000	-4.641158000	-3.887373000
6	1.217053000	-6.738055000	-2.764431000
1	2.986322000	-4.085429000	-5.326253000
1	3.107532000	-4.733962000	-2.581259000
1	1.699264000	-6.264767000	-4.819320000
1	0.797542000	-4.187412000	-4.783486000
1	0.188030000	-7.035990000	-2.981856000
1	1.842353000	-7.637068000	-2.728940000
1	1.234256000	-6.263237000	-1.777244000
6	-5.833891000	-6.263473000	3.779463000
6	-6.940648000	-5.370048000	4.318912000
8	-8.028721000	-5.217080000	3.694127000
6	-6.394780000	-7.457539000	2.987775000
6	-5.290838000	-8.445885000	2.528206000
6	-4.147943000	-7.724076000	1.841157000
8	-3.069815000	-7.489785000	2.481671000
7	-4.345916000	-7.286662000	0.589188000
1	-5.194727000	-5.652035000	3.126762000
1	-6.957547000	-7.080243000	2.127041000
1	-7.116432000	-8.005956000	3.604553000
1	-5.727288000	-9.197500000	1.858470000
1	-4.870460000	-8.969848000	3.391011000
1	-3.695114000	-6.628048000	0.126800000
1	-5.205934000	-7.492380000	0.103107000
7	-6.677241000	-4.750967000	5.499760000
6	-7.700201000	-3.974327000	6.195518000
6	-8.234679000	-2.748150000	5.452248000
8	-9.383973000	-2.318469000	5.707197000
1	-5.708108000	-4.737589000	5.836287000
1	-8.574951000	-4.597675000	6.401066000
7	-7.392404000	-2.172152000	4.551399000
6	-7.806141000	-1.038193000	3.744355000
6	-7.852467000	-1.332052000	2.245806000
8	-7.751551000	-0.384201000	1.419272000
1	-6.420045000	-2.474975000	4.450360000
1	-8.811406000	-0.751613000	4.075327000
7	-8.056073000	-2.626570000	1.888446000
6	-8.244176000	-3.045168000	0.500048000
6	-7.171274000	-4.003276000	-0.008301000
8	-5.908213000	-3.303707000	-0.182834000
1	-8.142182000	-3.336012000	2.615287000
1	-8.257338000	-2.146078000	-0.119682000
1	-7.048281000	-4.836496000	0.697898000
1	-7.492629000	-4.411234000	-0.975329000
1	-5.255949000	-3.838600000	-0.723231000
6	-7.363502000	3.181673000	-4.611876000
6	-6.885912000	2.056899000	-3.716795000
6	-7.558942000	1.761788000	-2.516445000
6	-5.782724000	1.262702000	-4.074611000
6	-7.154252000	0.696870000	-1.704199000
6	-5.371347000	0.192482000	-3.267369000
6	-6.060023000	-0.094895000	-2.083283000
1	-8.329215000	2.939313000	-5.075252000
1	-7.504313000	4.115004000	-4.052335000
1	-8.414661000	2.366429000	-2.223877000
1	-5.252336000	1.474350000	-5.000239000
1	-7.678423000	0.472141000	-0.779918000
1	-4.522435000	-0.418019000	-3.562368000
1	-5.766069000	-0.936452000	-1.464082000
26	-0.497032000	-2.609689000	1.118220000
26	-1.894367000	-2.190584000	-1.678918000
8	0.159720000	-4.600888000	0.604969000
1	1.137079000	-4.551034000	0.442223000
1	-0.177895000	-5.277495000	1.316238000
8	-4.557630000	-2.375235000	3.970045000
1	-4.327442000	-1.512566000	4.404571000
1	-4.484438000	-2.303359000	2.963627000
8	-4.394539000	-1.946311000	1.390470000
1	-5.134848000	-2.353316000	0.831074000
1	-4.405547000	-0.957533000	1.465048000
8	-2.260167000	-2.970562000	0.191361000
1	-2.672394000	-4.373571000	-0.257851000
1	-3.036684000	-2.547431000	0.682563000
8	-1.418700000	-4.602976000	8.791522000
1	-1.172454000	-5.318810000	8.104570000
1	-1.798283000	-4.987694000	9.596879000
8	-4.155684000	-4.821603000	-1.528994000
8	-2.970376000	-5.268846000	-0.702336000
8	-0.481818000	-3.680486000	-1.835474000
1	0.001873000	-4.048724000	-2.639113000
8	-0.801724000	-6.094503000	2.372526000
1	-1.553063000	-6.695865000	2.132564000
1	-1.000982000	-5.769737000	3.281724000
8	-2.341026000	-2.842717000	7.247253000
1	-2.011143000	-3.384922000	8.046989000
1	-2.398178000	-1.854299000	7.301299000
8	-2.222973000	-6.059860000	4.778026000
1	-2.632649000	-6.763130000	4.224028000
1	-2.905699000	-5.341101000	5.056535000
8	-0.857716000	-6.314561000	6.963662000
1	-1.391520000	-6.307241000	6.096776000
1	0.058268000	-6.594889000	6.810186000
8	-3.845791000	-4.241326000	5.614232000
1	-4.134907000	-3.572427000	4.906714000
1	-3.365460000	-3.732062000	6.346041000
1	3.980032000	1.190014000	6.787827000
1	3.550119000	4.835668000	3.618451000
1	-0.257494000	6.394917000	6.221951000
1	-1.672235000	7.638736000	1.768360000
1	-5.431686000	5.310892000	2.738622000
1	-4.606655000	5.747036000	-3.705886000
1	-1.967271000	7.714066000	-7.845855000
1	0.056766000	10.858243000	-4.640920000
1	6.738893000	4.840911000	-3.840342000
1	3.718243000	4.247758000	0.264977000
1	7.374725000	-1.324694000	-3.211131000
1	9.594927000	2.195965000	-0.670881000
1	6.231386000	1.152471000	2.867426000
1	8.994205000	-2.400235000	3.298061000
1	2.859631000	-0.991906000	-8.662313000
1	6.124577000	-2.480965000	-5.586207000
1	-7.281897000	-3.651329000	7.154428000
1	-7.141739000	-0.185707000	3.902667000
1	-5.198312000	-6.607646000	4.602176000
1	-9.218113000	-3.543967000	0.407243000
1	8.135942000	-4.317518000	-0.419783000
1	-6.654816000	3.379165000	-5.422953000
1	-0.074810000	-0.449851000	-5.340065000
1	3.813515000	-6.123494000	-3.429560000
1	-0.387307000	-4.252697000	-1.027353000
1	4.566396000	-2.001800000	3.504883000

[9]Re$_B$:

6	8.695296000	-0.844356000	3.703449000
6	8.068828000	0.207349000	2.797302000
8	8.130099000	0.051528000	1.539720000
6	7.810003000	-2.125753000	3.869428000
6	7.000872000	-2.481034000	2.661646000
7	7.516011000	-2.809472000	1.400647000
6	5.642710000	-2.427616000	2.490110000
6	6.513905000	-2.911411000	0.494908000
7	5.368891000	-2.687820000	1.153321000
1	9.649905000	-1.117278000	3.241329000

1	8.457925000	-2.956127000	4.176335000
1	7.103764000	-1.962132000	4.689498000
1	4.864427000	-2.207388000	3.196625000
1	6.603539000	-3.086879000	-0.578465000
1	4.395788000	-2.540786000	0.738865000
7	7.479732000	1.275894000	3.382999000
6	6.698425000	2.279981000	2.648182000
6	7.342769000	2.866482000	1.417781000
8	6.603731000	3.296354000	0.484942000
1	7.285980000	1.278506000	4.388879000
1	5.728726000	1.884767000	2.328214000
7	8.688789000	2.935950000	1.312068000
6	9.317375000	3.401546000	0.074190000
6	8.820691000	2.686788000	-1.193463000
8	8.821220000	3.298762000	-2.285510000
1	9.260132000	2.602755000	2.073953000
1	9.129181000	4.465917000	-0.087659000
7	8.416529000	1.387080000	-1.071567000
6	7.911244000	0.670448000	-2.237740000
6	6.473840000	1.011246000	-2.653501000
8	6.041435000	0.629642000	-3.774354000
1	8.395457000	0.929103000	-0.159608000
1	8.541501000	0.884718000	-3.104753000
7	5.714436000	1.677020000	-1.749172000
6	4.320794000	2.040746000	-2.006976000
6	4.110186000	3.557722000	-2.082706000
8	3.064198000	4.009353000	-2.641233000
6	3.358528000	1.403246000	-0.968456000
6	3.500500000	-0.126970000	-0.929019000
6	2.646787000	-0.797627000	0.131337000
8	1.641320000	-0.171109000	0.603402000
8	2.959892000	-2.015058000	0.503926000
1	6.124012000	1.948984000	-0.858783000
1	4.083728000	1.654502000	-3.002996000
1	2.331956000	1.670502000	-1.236596000
1	3.548123000	1.828638000	0.024687000
1	4.545342000	-0.419504000	-0.773234000
1	3.199265000	-0.572855000	-1.889615000
7	5.049287000	4.375328000	-1.554168000
6	4.855732000	5.823764000	-1.480018000
6	4.713901000	6.518962000	-2.834075000
8	4.018658000	7.558016000	-2.942581000
1	5.798640000	3.989098000	-0.983564000
1	3.958346000	6.071768000	-0.908129000
7	5.379895000	5.957794000	-3.880461000
6	5.267083000	6.504958000	-5.231940000
6	3.869991000	6.368733000	-5.860109000
8	3.546914000	7.120307000	-6.804349000
1	5.967951000	5.152946000	-3.708630000
1	5.491348000	7.574664000	-5.230963000
7	3.062285000	5.396813000	-5.345312000
6	1.640283000	5.300606000	-5.694618000
6	0.764065000	6.140139000	-4.744130000
8	-0.198396000	6.808120000	-5.195698000
6	1.182310000	3.832457000	-5.714933000
1	3.383931000	4.842303000	-4.560204000
1	1.515763000	5.758504000	-6.677818000
1	0.106926000	3.763630000	-5.897120000
1	1.712042000	3.297351000	-6.511528000
1	1.395668000	3.345176000	-4.756882000
7	1.091278000	6.110304000	-3.424184000
6	0.321563000	6.789781000	-2.373379000
6	-0.047011000	8.234419000	-2.731723000
8	-1.182960000	8.695677000	-2.454887000
6	1.173155000	6.755883000	-1.090771000
8	0.330015000	7.164293000	0.009624000
1	1.833013000	5.485607000	-3.118724000
1	-0.627797000	6.275161000	-2.191276000
1	1.537390000	5.731670000	-0.938652000
1	2.035448000	7.424197000	-1.205822000
1	0.800534000	7.221010000	0.873459000
7	0.929421000	8.981738000	-3.313218000
6	0.705821000	10.379510000	-3.677295000
6	-0.382862000	10.646820000	-4.728848000
8	-0.794284000	11.823487000	-4.869562000

1	1.836156000	8.562308000	-3.489772000
1	0.410255000	10.960332000	-2.798059000
7	-0.828602000	9.587776000	-5.452334000
6	-1.932120000	9.701671000	-6.395628000
6	-3.188448000	8.917792000	-6.002338000
8	-4.053614000	8.658699000	-6.877728000
1	-0.448527000	8.653491000	-5.307448000
1	-2.190794000	10.762756000	-6.475077000
7	-3.313622000	8.565662000	-4.695728000
6	-4.500292000	7.870684000	-4.190092000
6	-4.169077000	6.773103000	-3.166422000
6	-3.588986000	5.465997000	-3.695198000
6	-3.248935000	5.244763000	-5.040019000
6	-3.397774000	4.401391000	-2.792579000
6	-2.729413000	4.014404000	-5.460015000
6	-2.878238000	3.167657000	-3.196103000
6	-2.531294000	2.976496000	-4.541647000
8	-1.975925000	1.794252000	-5.013673000
1	-2.568625000	8.775559000	-4.036857000
1	-5.035774000	7.472025000	-5.056391000
1	-5.099751000	6.542661000	-2.626768000
1	-3.480368000	7.198337000	-2.420672000
1	-3.381245000	6.030588000	-5.774400000
1	-3.678828000	4.534621000	-1.749770000
1	-2.477969000	3.847833000	-6.501881000
1	-2.752337000	2.361378000	-2.483076000
1	-1.891532000	1.062221000	-4.349585000
6	-4.742304000	6.218749000	2.833671000
6	-3.282750000	6.585980000	2.729357000
8	-2.367370000	5.731105000	2.911877000
6	-5.179080000	5.205672000	1.724452000
6	-5.930740000	3.989617000	2.299124000
6	-4.964890000	3.063736000	3.042395000
8	-4.032952000	2.472520000	2.427786000
8	-5.178171000	3.018352000	4.342936000
1	-4.877496000	5.745236000	3.814469000
1	-5.826976000	5.709765000	1.000263000
1	-4.302303000	4.841616000	1.178850000
1	-6.376823000	3.400668000	1.494025000
1	-6.746519000	4.292464000	2.958810000
7	-2.952028000	7.883746000	2.459075000
6	-1.571738000	8.249338000	2.102148000
6	-0.545324000	7.809991000	3.143338000
8	0.588221000	7.374871000	2.790764000
1	-3.688414000	8.533778000	2.222112000
1	-1.279409000	7.805341000	1.144510000
7	-0.867822000	7.954585000	4.455566000
6	0.058135000	7.566671000	5.521958000
6	0.190398000	6.049245000	5.741149000
8	1.267529000	5.581981000	6.190713000
1	-1.779065000	8.323286000	4.689940000
1	1.059435000	7.936935000	5.294294000
7	-0.902706000	5.303841000	5.457952000
6	-0.922155000	3.825195000	5.535342000
6	0.090616000	3.275398000	4.507726000
8	1.027471000	2.507709000	4.850777000
6	-2.373515000	3.370923000	5.276302000
6	-2.782616000	2.019354000	5.838326000
8	-2.132136000	1.440661000	6.761248000
8	-3.883510000	1.500843000	5.341836000
1	-1.639300000	5.724325000	4.901523000
1	-0.579857000	3.500485000	6.519391000
1	-2.593104000	3.359786000	4.207377000
1	-3.057215000	4.101562000	5.728270000
7	-0.052999000	3.733460000	3.237943000
6	0.855783000	3.318180000	2.163097000
6	2.287513000	3.814935000	2.416038000
8	3.285368000	3.100356000	2.129801000
6	0.344847000	3.821181000	0.792625000
6	-1.008629000	3.237168000	0.308450000
6	-0.992527000	1.729700000	0.202524000
8	-0.872013000	1.077840000	1.306058000
8	-1.082801000	1.162887000	-0.955775000
1	-0.853566000	4.313162000	2.999455000
1	0.916032000	2.230110000	2.151004000

1	1.116850000	3.582509000	0.051450000
1	0.240174000	4.912347000	0.817640000
1	-1.810663000	3.507828000	1.005431000
1	-1.243693000	3.665905000	-0.668599000
7	2.426054000	5.067240000	2.933410000
6	3.741641000	5.621650000	3.227578000
6	4.525985000	4.837220000	4.281171000
8	5.773511000	4.705527000	4.156869000
1	1.617296000	5.664085000	3.073934000
1	4.378565000	5.624552000	2.338383000
7	3.841016000	4.314561000	5.327735000
6	4.526582000	3.451730000	6.290035000
6	4.749760000	2.022688000	5.768617000
8	5.908364000	1.503080000	5.768692000
1	2.858384000	4.538122000	5.474898000
1	5.513379000	3.858592000	6.515907000
7	3.657344000	1.357251000	5.335366000
6	3.775907000	0.008348000	4.769701000
6	2.662921000	-0.299194000	3.761761000
6	1.333982000	-0.623221000	4.371384000
7	0.198722000	-0.806229000	3.574181000
6	0.952025000	-0.766296000	5.689702000
6	-0.833483000	-1.032425000	4.401239000
7	-0.410274000	-1.027177000	5.681606000
1	2.749851000	1.824872000	5.263797000
1	3.790434000	-0.738837000	5.577474000
1	2.975822000	-1.141819000	3.129911000
1	2.561387000	0.555616000	3.082995000
1	1.502870000	-0.639810000	6.605045000
1	-1.858851000	-1.171808000	4.108180000
6	1.050214000	-0.158041000	-5.592949000
6	2.201813000	-1.052079000	-5.972359000
8	2.355027000	-2.217663000	-5.482860000
6	-0.281948000	-0.569212000	-6.297604000
6	-0.966487000	-1.823168000	-5.730091000
6	-1.468636000	-1.736445000	-4.288035000
8	-1.390485000	-0.573629000	-3.667946000
8	-1.917161000	-2.785011000	-3.735033000
1	0.892664000	-0.195628000	-4.511282000
1	-0.954622000	0.288747000	-6.215737000
1	-0.088295000	-0.727166000	-7.368037000
1	-0.306893000	-2.698206000	-5.784603000
1	-1.837124000	-2.066384000	-6.354675000
7	3.072224000	-0.595443000	-6.913878000
6	4.034512000	-1.460650000	-7.607023000
6	5.076261000	-2.177933000	-6.746883000
8	5.521139000	-3.279354000	-7.141442000
1	2.918053000	0.330498000	-7.287161000
1	3.511474000	-2.256815000	-8.145074000
7	5.501405000	-1.571787000	-5.601806000
6	6.463278000	-2.219201000	-4.721241000
6	5.836135000	-2.808857000	-3.459740000
8	6.543841000	-2.965598000	-2.417559000
1	5.206873000	-0.640183000	-5.332450000
1	6.934188000	-3.033992000	-5.282397000
7	4.547581000	-3.190919000	-3.545988000
6	3.841931000	-3.889552000	-2.464927000
6	2.394570000	-4.206403000	-2.860939000
8	1.658741000	-2.954189000	-3.042957000
6	1.680440000	-5.033151000	-1.796329000
1	4.026029000	-2.956576000	-4.387857000
1	3.818366000	-3.269213000	-1.562538000
1	2.386600000	-4.747925000	-3.817407000
1	1.749476000	-2.626640000	-3.981321000
1	0.642471000	-5.203229000	-2.094773000
1	2.169877000	-6.004429000	-1.664353000
1	1.680416000	-4.509598000	-0.834986000
6	-5.122168000	-4.097689000	2.192351000
6	-6.353514000	-3.665237000	2.984088000
8	-7.447320000	-3.422109000	2.402227000
6	-5.531494000	-4.884585000	0.929015000
6	-4.364539000	-5.491437000	0.103812000
6	-3.405940000	-4.461051000	-0.460653000
8	-2.628275000	-3.841533000	0.351977000
7	-3.399775000	-4.231279000	-1.773366000
1	-4.559693000	-3.200458000	1.905254000
1	-6.139444000	-4.234417000	0.291808000
1	-6.186498000	-5.713712000	1.223231000
1	-4.788136000	-6.100162000	-0.703355000
1	-3.776560000	-6.163431000	0.741817000
1	-2.798268000	-3.535765000	-2.238736000
1	-4.051526000	-4.709221000	-2.377924000
7	-6.219685000	-3.551701000	4.334825000
6	-7.370167000	-3.252180000	5.188789000
6	-8.014002000	-1.872542000	5.016601000
8	-9.200120000	-1.703509000	5.382752000
1	-5.299230000	-3.557461000	4.771665000
1	-8.170406000	-3.973453000	5.002899000
7	-7.235318000	-0.894686000	4.479317000
6	-7.768753000	0.424185000	4.172252000
6	-8.016360000	0.665815000	2.680981000
8	-8.173499000	1.845706000	2.267150000
1	-6.243043000	-1.048610000	4.287212000
1	-8.722040000	0.534601000	4.699863000
7	-8.080677000	-0.432609000	1.886830000
6	-8.335123000	-0.360585000	0.450369000
6	-7.167459000	-0.872621000	-0.387640000
8	-6.019717000	0.016979000	-0.263707000
1	-7.970154000	-1.357579000	2.295804000
1	-8.544756000	0.681028000	0.200756000
1	-6.887434000	-1.883400000	-0.063293000
1	-7.472144000	-0.917319000	-1.440483000
1	-5.479410000	0.063279000	-1.113120000
6	-9.867567000	6.765992000	-2.505335000
6	-9.027080000	5.588576000	-2.058157000
6	-9.311750000	4.908265000	-0.862122000
6	-7.953340000	5.133421000	-2.845616000
6	-8.554955000	3.798680000	-0.462011000
6	-7.191477000	4.026833000	-2.453036000
6	-7.490722000	3.355272000	-1.259522000
1	-10.613337000	6.463093000	-3.253258000
1	-10.411163000	7.211410000	-1.665099000
1	-10.136109000	5.248720000	-0.239940000
1	-7.715993000	5.646728000	-3.774125000
1	-8.780254000	3.281079000	0.465975000
1	-6.364114000	3.694327000	-3.072023000
1	-6.907450000	2.491129000	-0.953552000
26	-0.142727000	-0.824030000	1.531503000
26	-1.623171000	-0.703575000	-1.611713000
8	0.507173000	-2.821744000	1.162366000
1	1.498764000	-2.793592000	1.088787000
1	0.041245000	-3.547187000	1.745534000
8	-4.376992000	-0.729302000	3.931843000
1	-4.235854000	0.106451000	4.458773000
1	-4.289949000	-0.541598000	2.943226000
8	-4.063685000	0.009846000	1.430754000
1	-4.885703000	-0.048266000	0.820459000
1	-3.901952000	0.962921000	1.682489000
8	-1.993232000	-1.316795000	0.662729000
1	-2.248028000	-2.275397000	0.502475000
1	-2.812160000	-0.745539000	0.936267000
8	-1.400430000	-2.774871000	8.936551000
1	-0.953000000	-3.362190000	8.243120000
1	-1.599591000	-3.237953000	9.763895000
8	-3.633248000	-1.159920000	-1.564846000
8	-4.161511000	0.010460000	-2.114215000
8	-0.114621000	-2.065245000	-1.317379000
1	0.514461000	-2.388373000	-2.028290000
8	-0.968430000	-4.401706000	2.397199000
1	-1.721878000	-4.382942000	1.756296000
1	-1.263121000	-4.383277000	3.348874000
8	-2.547238000	-1.208676000	7.329466000
1	-2.218087000	-1.684675000	8.163646000
1	-2.611563000	-0.215614000	7.343520000
8	-1.972047000	-4.451697000	4.914621000
1	-2.367718000	-5.336253000	5.024276000
1	-2.692457000	-3.677379000	5.093203000
8	-0.326437000	-4.068894000	6.967685000
1	-0.908030000	-4.238662000	6.163213000
1	0.559789000	-3.780254000	6.694242000

8	-3.601254000	-2.651571000	5.429443000
1	-3.903510000	-1.954610000	4.745096000
1	-3.322605000	-2.150292000	6.270966000
1	3.927402000	3.419857000	7.204799000
1	3.601135000	6.654769000	3.560606000
1	-0.274288000	8.031358000	6.455210000
1	-1.528673000	9.338378000	1.993706000
1	-5.376901000	7.111534000	2.815060000
1	-5.170176000	8.600129000	-3.710820000
1	-1.637416000	9.349011000	-7.388342000
1	1.649655000	10.788171000	-4.048950000
1	6.000290000	6.003675000	-5.870320000
1	5.717828000	6.241176000	-0.950318000
1	7.947165000	-0.406832000	-2.041685000
1	10.398270000	3.258263000	0.166846000
1	6.481571000	3.109521000	3.336440000
1	8.911800000	-0.441342000	4.697457000
1	4.558299000	-0.844397000	-8.344885000
1	7.224451000	-1.503498000	-4.415910000
1	-7.048990000	-3.361431000	6.229432000
1	-7.088730000	1.205912000	4.521819000
1	-4.453420000	-4.694075000	2.826429000
1	-9.223074000	-0.960878000	0.210274000
1	8.494269000	-2.818289000	1.158420000
1	-9.252606000	7.548449000	-2.964805000
1	1.277145000	0.881613000	-5.852719000
1	4.377388000	-4.818122000	-2.228178000
1	0.027651000	-2.536443000	-0.453410000
1	4.750356000	-0.048157000	4.274685000

^{9}TS1$_{B}$:

6	8.807846000	-1.286417000	3.257449000
6	8.100051000	-0.147475000	2.537499000
8	8.120712000	-0.097181000	1.272398000
6	7.894578000	-2.538611000	3.455170000
6	7.030113000	-2.874606000	2.280092000
7	7.492593000	-3.219622000	1.001762000
6	5.664236000	-2.846285000	2.169778000
6	6.449341000	-3.373311000	0.148818000
7	5.333222000	-3.151867000	0.855080000
1	9.676071000	-1.550302000	2.645785000
1	8.532534000	-3.387955000	3.731533000
1	7.227187000	-2.363590000	4.305383000
1	4.913984000	-2.624763000	2.906738000
1	6.491378000	-3.598175000	-0.919107000
1	4.332756000	-3.046893000	0.481683000
7	7.453148000	0.769280000	3.299232000
6	6.546923000	1.783140000	2.749028000
6	7.065011000	2.579931000	1.582627000
8	6.271185000	2.958804000	0.674876000
1	7.335237000	0.637585000	4.302886000
1	5.597458000	1.346801000	2.426350000
7	8.372917000	2.918661000	1.501855000
6	8.846445000	3.696094000	0.353789000
6	8.446726000	3.086453000	-0.996879000
8	8.201546000	3.831338000	-1.977658000
1	9.007412000	2.657262000	2.241628000
1	8.433472000	4.707965000	0.366476000
7	8.410969000	1.725862000	-1.076529000
6	8.063038000	1.066944000	-2.328996000
6	6.574076000	1.059508000	-2.693151000
8	6.234351000	0.645373000	-3.834203000
1	8.491752000	1.154425000	-0.235672000
1	8.575575000	1.560799000	-3.158988000
7	5.702234000	1.491102000	-1.755901000
6	4.265616000	1.662858000	-2.027229000
6	3.916971000	3.160698000	-2.071426000
8	3.358852000	3.665691000	-3.086645000
6	3.373976000	0.918960000	-1.012498000
6	3.574556000	-0.602320000	-1.023511000
6	2.629399000	-1.321792000	-0.074460000
8	1.595672000	-0.691490000	0.332762000
8	2.888216000	-2.553974000	0.272234000
1	6.059467000	1.835320000	-0.866292000
1	4.097037000	1.281502000	-3.035305000
1	2.328377000	1.138597000	-1.254348000
1	3.536235000	1.314360000	-0.003060000
1	4.610019000	-0.865691000	-0.776996000
1	3.376047000	-1.019318000	-2.021744000
7	4.250367000	3.879492000	-0.972488000
6	3.936514000	5.292761000	-0.812298000
6	4.646497000	6.239608000	-1.766965000
8	4.188601000	7.410750000	-1.912389000
1	4.705083000	3.427250000	-0.181108000
1	2.865499000	5.484967000	-0.951935000
7	5.685345000	5.787217000	-2.504345000
6	6.172186000	6.576263000	-3.635404000
6	5.079999000	6.899309000	-4.668938000
8	5.237516000	7.867702000	-5.447043000
1	6.149257000	4.912783000	-2.281969000
1	6.569754000	7.540854000	-3.309157000
7	3.974050000	6.095770000	-4.682408000
6	2.836393000	6.370403000	-5.557240000
6	1.630089000	6.969215000	-4.808835000
8	0.516490000	7.064709000	-5.394318000
6	2.405380000	5.113661000	-6.332297000
1	3.902439000	5.276284000	-4.078062000
1	3.174422000	7.139388000	-6.263453000
1	1.510876000	5.328527000	-6.920429000
1	3.213819000	4.797836000	-6.998890000
1	2.177169000	4.298270000	-5.636743000
7	1.814217000	7.440515000	-3.544395000
6	0.672854000	7.893375000	-2.741600000
6	-0.013103000	9.145008000	-3.310129000
8	-1.187647000	9.426965000	-2.952895000
6	1.065459000	8.141839000	-1.277549000
8	0.981787000	6.876025000	-0.553214000
1	2.724494000	7.374974000	-3.085991000
1	-0.106765000	7.122890000	-2.753653000
1	2.079867000	8.549477000	-1.216189000
1	0.354340000	8.861851000	-0.854947000
1	1.142669000	7.009796000	0.412178000
7	0.694414000	9.900545000	-4.185317000
6	0.096788000	11.034821000	-4.881319000
6	-0.983167000	10.682499000	-5.915852000
8	-1.655958000	11.613535000	-6.414194000
1	1.629655000	9.602091000	-4.425689000
1	-0.384630000	11.702008000	-4.160819000
7	-1.156954000	9.365933000	-6.219800000
6	-2.247402000	8.928384000	-7.081688000
6	-3.473276000	8.373716000	-6.347802000
8	-4.402411000	7.855118000	-7.017692000
1	-0.542292000	8.644982000	-5.842734000
1	-2.577506000	9.783349000	-7.678981000
7	-3.490099000	8.489048000	-4.993945000
6	-4.626913000	8.052635000	-4.183015000
6	-4.274118000	6.969942000	-3.148475000
6	-3.889429000	5.592306000	-3.673337000
6	-4.134789000	5.164053000	-4.990891000
6	-3.316542000	4.667880000	-2.781946000
6	-3.823236000	3.860909000	-5.393864000
6	-3.005861000	3.361461000	-3.168545000
6	-3.261036000	2.956431000	-4.486598000
8	-2.971411000	1.676675000	-4.940535000
1	-2.702646000	8.910771000	-4.512358000
1	-5.398211000	7.708830000	-4.876254000
1	-5.152061000	6.861565000	-2.490571000
1	-3.461245000	7.354753000	-2.514049000
1	-4.551905000	5.845910000	-5.725024000
1	-3.122813000	4.971781000	-1.754780000
1	-4.009137000	3.534522000	-6.410799000
1	-2.579372000	2.656905000	-2.461462000
1	-2.551100000	1.090254000	-4.262079000
6	-4.697992000	6.239422000	2.431099000
6	-3.217871000	6.486520000	2.282293000
8	-2.354120000	5.603488000	2.549559000
6	-5.229808000	5.131118000	1.460622000
6	-5.950503000	3.992511000	2.203084000
6	-4.951203000	3.079074000	2.917993000
8	-4.028508000	2.503570000	2.274561000

8	-5.147657000	3.011853000	4.219858000
1	-4.859623000	5.908966000	3.465306000
1	-5.925211000	5.579012000	0.743828000
1	-4.406526000	4.697172000	0.884261000
1	-6.497413000	3.355019000	1.502210000
1	-6.681773000	4.380792000	2.917208000
7	-2.806339000	7.727630000	1.873294000
6	-1.406873000	7.965100000	1.506632000
6	-0.409765000	7.637662000	2.615124000
8	0.729583000	7.176290000	2.320054000
1	-3.498844000	8.398412000	1.571106000
1	-1.112927000	7.358189000	0.644059000
7	-0.764082000	7.898431000	3.897853000
6	0.131085000	7.596025000	5.019004000
6	0.246844000	6.098016000	5.356672000
8	1.325195000	5.648875000	5.818783000
1	-1.680789000	8.285236000	4.075316000
1	1.139931000	7.943536000	4.790125000
7	-0.863093000	5.349592000	5.152377000
6	-0.903892000	3.882513000	5.344786000
6	0.075510000	3.227840000	4.347483000
8	0.982452000	2.446480000	4.736031000
6	-2.366791000	3.441147000	5.147759000
6	-2.744687000	2.085917000	5.723363000
8	-2.080388000	1.547220000	6.660374000
8	-3.823740000	1.530834000	5.224291000
1	-1.615106000	5.741441000	4.597508000
1	-0.550265000	3.626773000	6.344895000
1	-2.628793000	3.439654000	4.088902000
1	-3.022992000	4.173971000	5.636472000
7	-0.058557000	3.610349000	3.050461000
6	0.844754000	3.108115000	2.008248000
6	2.292115000	3.557546000	2.266729000
8	3.263377000	2.803177000	1.989687000
6	0.368548000	3.549099000	0.603265000
6	-1.020194000	3.025150000	0.150902000
6	-1.100011000	1.516190000	0.057947000
8	-0.742770000	0.835639000	1.071734000
8	-1.534877000	0.981385000	-1.056873000
1	-0.838014000	4.202327000	2.777380000
1	0.859348000	2.018869000	2.050154000
1	1.125954000	3.214338000	-0.114637000
1	0.348651000	4.643592000	0.539968000
1	-1.805681000	3.344026000	0.849266000
1	-1.248105000	3.458558000	-0.824939000
7	2.470549000	4.799346000	2.795058000
6	3.785212000	5.305342000	3.170984000
6	4.507144000	4.461566000	4.222820000
8	5.753044000	4.292149000	4.128805000
1	1.682126000	5.429305000	2.886795000
1	4.468005000	5.334721000	2.317412000
7	3.771990000	3.929140000	5.227935000
6	4.390052000	3.012587000	6.187098000
6	4.607499000	1.595571000	5.628498000
8	5.743006000	1.035529000	5.701708000
1	2.793024000	4.181412000	5.337174000
1	5.373143000	3.385925000	6.478228000
7	3.531585000	0.987738000	5.080744000
6	3.647201000	-0.355484000	4.498942000
6	2.558960000	-0.639645000	3.458435000
6	1.196795000	-0.864026000	4.038999000
7	0.078048000	-1.095488000	3.230461000
6	0.777773000	-0.852776000	5.354966000
6	-0.980566000	-1.201122000	4.049327000
7	-0.590616000	-1.065655000	5.335635000
1	2.649746000	1.493306000	4.967528000
1	3.625316000	-1.111081000	5.298323000
1	2.849440000	-1.526297000	2.877870000
1	2.528245000	0.190165000	2.741717000
1	1.305437000	-0.641913000	6.268620000
1	-2.002765000	-1.344336000	3.745922000
6	1.577345000	0.682978000	-4.788314000
6	2.460327000	-0.291136000	-5.533470000
8	2.340180000	-1.548395000	-5.392109000
6	0.250167000	0.991154000	-5.540872000
6	-0.641835000	-0.228695000	-5.850700000
6	-1.075505000	-1.039509000	-4.636956000
8	-1.515645000	-0.306495000	-3.583869000
8	-1.031772000	-2.282076000	-4.617482000
1	1.336580000	0.242499000	-3.815147000
1	-0.315551000	1.698735000	-4.929244000
1	0.479178000	1.502125000	-6.485303000
1	-0.155350000	-0.924506000	-6.539673000
1	-1.551434000	0.150021000	-6.334206000
7	3.372999000	0.208979000	-6.406851000
6	4.105761000	-0.641928000	-7.352623000
6	5.008595000	-1.717452000	-6.738682000
8	5.237149000	-2.764087000	-7.385701000
1	3.468263000	1.211690000	-6.483681000
1	3.405927000	-1.182409000	-7.996232000
7	5.533399000	-1.461560000	-5.508798000
6	6.359965000	-2.433212000	-4.810089000
6	5.624873000	-3.198588000	-3.707328000
8	6.275382000	-3.684161000	-2.733180000
1	5.448052000	-0.550759000	-5.063305000
1	6.727281000	-3.161863000	-5.542135000
7	4.298088000	-3.365504000	-3.863753000
6	3.482936000	-4.183391000	-2.955546000
6	1.998930000	-4.100869000	-3.323787000
8	1.552871000	-2.710682000	-3.141011000
6	1.134826000	-5.013953000	-2.460948000
1	3.829746000	-2.880227000	-4.625349000
1	3.602559000	-3.832173000	-1.925296000
1	1.860645000	-4.356667000	-4.382513000
1	1.619997000	-2.221102000	-4.007557000
1	0.084217000	-4.903838000	-2.742991000
1	1.423319000	-6.061668000	-2.600249000
1	1.239160000	-4.764172000	-1.399984000
6	-5.451106000	-4.513528000	3.168489000
6	-6.596324000	-3.817772000	3.892116000
8	-7.692411000	-3.599050000	3.301851000
6	-5.990482000	-5.667844000	2.298377000
6	-4.924227000	-6.337713000	1.393564000
6	-4.210746000	-5.323703000	0.518543000
8	-3.103502000	-4.824971000	0.872773000
7	-4.827674000	-4.905181000	-0.606009000
1	-4.951320000	-3.770739000	2.532198000
1	-6.811786000	-5.274438000	1.690051000
1	-6.427749000	-6.439596000	2.944572000
1	-5.408365000	-7.106251000	0.778147000
1	-4.162382000	-6.831660000	2.004446000
1	-4.402725000	-4.129658000	-1.128642000
1	-5.701962000	-5.302322000	-0.911389000
7	-6.381990000	-3.446975000	5.181836000
6	-7.458142000	-2.878066000	5.992962000
6	-8.036876000	-1.543711000	5.511775000
8	-9.198656000	-1.219612000	5.850894000
1	-5.432317000	-3.453509000	5.560283000
1	-8.305445000	-3.568144000	6.031924000
7	-7.221406000	-0.767543000	4.748019000
6	-7.680694000	0.484470000	4.169059000
6	-7.822467000	0.453791000	2.647256000
8	-7.868551000	1.541151000	2.011181000
1	-6.246694000	-1.025384000	4.574809000
1	-8.661199000	0.709788000	4.603071000
7	-7.928870000	-0.766844000	2.060873000
6	-8.148015000	-0.930074000	0.624742000
6	-6.969274000	-1.555729000	-0.116094000
8	-5.841756000	-0.633805000	-0.144382000
1	-7.927076000	-1.604887000	2.640260000
1	-8.359404000	0.056285000	0.206763000
1	-6.671901000	-2.498001000	0.364448000
1	-7.277080000	-1.773526000	-1.146812000
1	-5.195280000	-0.838720000	-0.881840000
6	-9.505758000	6.295926000	-2.962890000
6	-8.766275000	5.124838000	-2.350466000
6	-9.202461000	4.546088000	-1.145437000
6	-7.642150000	4.570748000	-2.988482000
6	-8.543770000	3.438821000	-0.596474000
6	-6.978400000	3.464255000	-2.444807000

6	-7.427945000	2.892985000	-1.247294000
1	-10.291463000	5.955457000	-3.651640000
1	-9.992068000	6.910073000	-2.196380000
1	-10.068067000	4.964593000	-0.636923000
1	-7.283689000	5.004533000	-3.918130000
1	-8.879740000	2.997062000	0.335888000
1	-6.110747000	3.057398000	-2.953867000
1	-6.920727000	2.032464000	-0.820839000
26	-0.255465000	-1.215634000	1.155908000
26	-1.921216000	-0.843266000	-1.754865000
8	0.277684000	-3.258725000	0.813406000
1	1.266111000	-3.289204000	0.743002000
1	-0.169275000	-3.889791000	1.520428000
8	-4.393807000	-0.853538000	4.133032000
1	-4.219474000	-0.000610000	4.619383000
1	-4.302813000	-0.680791000	3.136097000
8	-4.083435000	-0.105544000	1.663305000
1	-4.876788000	-0.272196000	1.044426000
1	-3.952270000	0.869169000	1.827328000
8	-2.153307000	-1.464385000	0.450300000
1	-2.827386000	-2.281536000	-0.211928000
1	-2.813773000	-0.874212000	0.945692000
8	-0.767710000	-2.407112000	8.765832000
1	-0.390768000	-3.040352000	8.067026000
1	-0.744420000	-2.768515000	9.664475000
8	-3.807537000	-1.249977000	-1.736876000
8	-3.355564000	-2.615070000	-1.246994000
8	-0.385382000	-2.139946000	-1.537144000
1	0.319910000	-2.347212000	-2.242585000
8	-1.002097000	-4.656063000	2.446346000
1	-1.835637000	-4.862033000	1.944247000
1	-1.194502000	-4.447542000	3.394500000
8	-2.401749000	-1.078934000	7.362280000
1	-1.836517000	-1.478758000	8.107563000
1	-2.464502000	-0.086059000	7.326417000
8	-1.849070000	-4.424540000	5.087330000
1	-2.169390000	-5.336332000	5.214605000
1	-2.603804000	-3.720696000	5.313435000
8	0.090270000	-3.837451000	6.793486000
1	-0.622423000	-4.110897000	6.134232000
1	0.830207000	-3.417270000	6.321832000
8	-3.593669000	-2.718331000	5.709522000
1	-3.909157000	-2.065927000	4.989915000
1	-3.263189000	-2.153071000	6.484649000
1	3.746054000	2.961404000	7.069918000
1	3.651612000	6.325109000	3.544138000
1	-0.226812000	8.135504000	5.901013000
1	-1.304105000	9.019445000	1.229241000
1	-5.261511000	7.169950000	2.300929000
1	-5.032123000	8.921953000	-3.647441000
1	-1.897265000	8.148063000	-7.762748000
1	0.889320000	11.596479000	-5.382595000
1	6.979842000	6.016333000	-4.114542000
1	4.183183000	5.575380000	0.214080000
1	8.406755000	0.029258000	-2.288031000
1	9.937000000	3.765508000	0.411313000
1	6.324622000	2.500778000	3.546765000
1	9.177166000	-0.970859000	4.239029000
1	4.713902000	0.011350000	-7.985891000
1	7.213985000	-1.931553000	-4.352052000
1	-7.077488000	-2.750223000	7.011317000
1	-6.999916000	1.303373000	4.417736000
1	-4.698082000	-4.871231000	3.880282000
1	-9.026615000	-1.570114000	0.471025000
1	8.459957000	-3.262976000	0.721518000
1	-8.831780000	6.940476000	-3.538149000
1	2.106374000	1.625563000	-4.599184000
1	3.824774000	-5.225410000	-3.000326000
1	-0.324543000	-2.784085000	-0.777039000
1	4.635015000	-0.425780000	4.031633000

^{9}IM1$_{B}$:

6	8.749477000	-3.037038000	3.198787000
6	8.046148000	-1.888788000	2.489740000
8	8.065122000	-1.831413000	1.224613000
6	7.840444000	-4.297030000	3.364660000
6	6.974437000	-4.609668000	2.184020000
7	7.432613000	-4.914290000	0.893942000
6	5.607478000	-4.598995000	2.083814000
6	6.385647000	-5.061103000	0.044250000
7	5.271437000	-4.875460000	0.764081000
1	9.625703000	-3.286885000	2.592493000
1	8.481883000	-5.150447000	3.619686000
1	7.173214000	-4.145825000	4.219547000
1	4.859209000	-4.407290000	2.831261000
1	6.424700000	-5.257036000	-1.029701000
1	4.266007000	-4.772749000	0.402073000
7	7.407709000	-0.972293000	3.258096000
6	6.511116000	0.055403000	2.716290000
6	7.032612000	0.844780000	1.546037000
8	6.241362000	1.222702000	0.635919000
1	7.288787000	-1.110789000	4.260631000
1	5.553156000	-0.366893000	2.400532000
7	8.341679000	1.179481000	1.463632000
6	8.815960000	1.952154000	0.312400000
6	8.409147000	1.342711000	-1.036390000
8	8.162238000	2.088036000	-2.016525000
1	8.975940000	0.921078000	2.204663000
1	8.408520000	2.966309000	0.324411000
7	8.371176000	-0.017538000	-1.116241000
6	8.020056000	-0.675160000	-2.368584000
6	6.532299000	-0.669113000	-2.736841000
8	6.195007000	-1.059554000	-3.886638000
1	8.450189000	-0.588934000	-0.275221000
1	8.538945000	-0.188259000	-3.198581000
7	5.657836000	-0.256222000	-1.792774000
6	4.223383000	-0.073478000	-2.067802000
6	3.881695000	1.426030000	-2.108739000
8	3.311891000	1.930231000	-3.118074000
6	3.324485000	-0.817104000	-1.059719000
6	3.497289000	-2.342098000	-1.095916000
6	2.559205000	-3.054128000	-0.134142000
8	1.531219000	-2.415735000	0.274906000
8	2.818292000	-4.283106000	0.223084000
1	6.013920000	0.074095000	-0.897604000
1	4.054710000	-0.448542000	-3.078270000
1	2.281319000	-0.575434000	-1.289801000
1	3.502548000	-0.440453000	-0.045520000
1	4.532968000	-2.627714000	-0.877849000
1	3.266971000	-2.741680000	-2.094764000
7	4.231601000	2.146468000	-1.016433000
6	3.929677000	3.563067000	-0.859985000
6	4.636105000	4.498510000	-1.829021000
8	4.179483000	5.668682000	-1.985411000
1	4.695034000	1.695496000	-0.229285000
1	2.858841000	3.762414000	-0.990243000
7	5.670572000	4.036437000	-2.566868000
6	6.153865000	4.813505000	-3.707821000
6	5.058953000	5.125786000	-4.741826000
8	5.215539000	6.083834000	-5.532841000
1	6.137088000	3.165356000	-2.335964000
1	6.552776000	5.781380000	-3.393047000
7	3.951809000	4.324111000	-4.741105000
6	2.811059000	4.587471000	-5.615021000
6	1.607616000	5.194929000	-4.868640000
8	0.489449000	5.276891000	-5.447482000
6	2.378269000	3.320619000	-6.372564000
1	3.879413000	3.514255000	-4.124142000
1	3.145925000	5.347739000	-6.332149000
1	1.479301000	3.526035000	-6.957245000
1	3.183362000	2.999375000	-7.040716000
1	2.157157000	2.513560000	-5.664893000
7	1.800376000	5.689182000	-3.614263000
6	0.664756000	6.153605000	-2.810186000
6	-0.021539000	7.401379000	-3.386720000
8	-1.187417000	7.697729000	-3.012472000
6	1.064707000	6.412740000	-1.350213000
8	0.982337000	5.151972000	-0.617358000
1	2.713080000	5.628745000	-3.159990000
1	-0.117264000	5.385256000	-2.811845000

1	2.080099000	6.818872000	-1.296253000
1	0.356582000	7.136920000	-0.929894000
1	1.134712000	5.291809000	0.348682000
7	0.672946000	8.134289000	-4.290759000
6	0.066743000	9.254452000	-5.002102000
6	-1.033904000	8.881330000	-6.007224000
8	-1.715275000	9.802243000	-6.512510000
1	1.602845000	7.826988000	-4.540302000
1	-0.398692000	9.940618000	-4.289006000
7	-1.215540000	7.558680000	-6.278958000
6	-2.324060000	7.105319000	-7.109203000
6	-3.548710000	6.597743000	-6.339471000
8	-4.500787000	6.082920000	-6.979205000
1	-0.592895000	6.845316000	-5.900717000
1	-2.651002000	7.940960000	-7.734781000
7	-3.537939000	6.748141000	-4.989113000
6	-4.670484000	6.364186000	-4.146541000
6	-4.326028000	5.302989000	-3.087045000
6	-3.993963000	3.899149000	-3.577598000
6	-4.273536000	3.442412000	-4.878441000
6	-3.435873000	2.981877000	-2.669122000
6	-4.011227000	2.118496000	-5.248406000
6	-3.175761000	1.655312000	-3.021995000
6	-3.465314000	1.220856000	-4.323803000
8	-3.226393000	-0.078752000	-4.743396000
1	-2.733083000	7.165592000	-4.533330000
1	-5.462787000	6.019735000	-4.815368000
1	-5.192447000	5.241596000	-2.408024000
1	-3.488468000	5.682945000	-2.482191000
1	-4.679858000	4.116571000	-5.625629000
1	-3.210359000	3.309187000	-1.655632000
1	-4.222453000	1.770415000	-6.253162000
1	-2.755334000	0.956828000	-2.306055000
1	-2.795473000	-0.658009000	-4.063091000
6	-4.714803000	4.453141000	2.263216000
6	-3.236356000	4.723482000	2.143666000
8	-2.365925000	3.850530000	2.422787000
6	-5.213519000	3.363838000	1.254006000
6	-5.956372000	2.210481000	1.951728000
6	-4.976021000	1.298395000	2.694614000
8	-4.052966000	0.701264000	2.074135000
8	-5.185288000	1.262317000	3.996859000
1	-4.886511000	4.090722000	3.284821000
1	-5.885577000	3.823985000	0.522837000
1	-4.371264000	2.941423000	0.696853000
1	-6.469056000	1.579081000	1.220573000
1	-6.718107000	2.585656000	2.640380000
7	-2.832783000	5.970729000	1.746052000
6	-1.429991000	6.222768000	1.400997000
6	-0.446337000	5.903241000	2.524183000
8	0.701021000	5.450725000	2.246677000
1	-3.527688000	6.633910000	1.432670000
1	-1.117866000	5.619396000	0.542370000
7	-0.821896000	6.161784000	3.801507000
6	0.057775000	5.864959000	4.936435000
6	0.172755000	4.367779000	5.278948000
8	1.245713000	3.920930000	5.755215000
1	-1.743999000	6.542009000	3.964707000
1	1.068692000	6.214968000	4.721003000
7	-0.933907000	3.618021000	5.060305000
6	-0.975349000	2.150092000	5.246182000
6	0.038539000	1.504239000	4.277781000
8	0.935701000	0.723113000	4.690101000
6	-2.429995000	1.703455000	4.998538000
6	-2.826836000	0.353048000	5.572651000
8	-2.202378000	-0.164848000	6.548684000
8	-3.877249000	-0.216705000	5.032460000
1	-1.677377000	4.010023000	4.494554000
1	-0.654641000	1.890948000	6.256513000
1	-2.654430000	1.693830000	3.931296000
1	-3.104918000	2.438409000	5.457851000
7	-0.058618000	1.894068000	2.980519000
6	0.863757000	1.389844000	1.957056000
6	2.304764000	1.838734000	2.238181000
8	3.280054000	1.081248000	1.983613000
6	0.412014000	1.829229000	0.544425000
6	-0.975401000	1.315152000	0.082858000
6	-1.075432000	-0.195085000	0.017800000
8	-0.773057000	-0.857263000	1.067032000
8	-1.464049000	-0.736560000	-1.105234000
1	-0.834227000	2.482449000	2.689402000
1	0.874503000	0.300652000	2.001294000
1	1.174567000	1.482944000	-0.162300000
1	0.403384000	2.923474000	0.475861000
1	-1.764849000	1.658413000	0.764037000
1	-1.183409000	1.731568000	-0.904719000
7	2.475125000	3.084240000	2.760627000
6	3.784597000	3.588714000	3.155636000
6	4.490863000	2.734292000	4.209438000
8	5.737448000	2.562664000	4.132351000
1	1.684791000	3.713805000	2.838852000
1	4.477777000	3.625427000	2.310538000
7	3.738778000	2.194426000	5.198471000
6	4.339001000	1.265262000	6.156889000
6	4.554240000	-0.148664000	5.589198000
8	5.688041000	-0.712217000	5.660449000
1	2.756953000	2.442077000	5.290419000
1	5.320881000	1.630186000	6.462218000
7	3.477301000	-0.751505000	5.037476000
6	3.591189000	-2.092692000	4.450609000
6	2.497332000	-2.374431000	3.415600000
6	1.135869000	-2.588860000	4.001864000
7	0.011986000	-2.809135000	3.197346000
6	0.721101000	-2.573634000	5.319139000
6	-1.045155000	-2.903667000	4.018976000
7	-0.649727000	-2.772614000	5.303954000
1	2.597914000	-0.242014000	4.921485000
1	3.574108000	-2.850609000	5.247954000
1	2.781894000	-3.263700000	2.836014000
1	2.468113000	-1.546117000	2.697081000
1	1.254540000	-2.365078000	6.229963000
1	-2.071092000	-3.032457000	3.719933000
6	1.368328000	-1.088283000	-4.697451000
6	2.244878000	-2.005122000	-5.520093000
8	2.149563000	-3.270577000	-5.455657000
6	0.012400000	-0.755273000	-5.386742000
6	-0.902757000	-1.961930000	-5.679851000
6	-1.305516000	-2.773453000	-4.452687000
8	-1.748869000	-2.020975000	-3.413900000
8	-1.231239000	-4.013459000	-4.424737000
1	1.167495000	-1.582552000	-3.741129000
1	-0.520229000	-0.057237000	-4.735617000
1	0.205523000	-0.226119000	-6.329140000
1	-0.440871000	-2.662359000	-6.381194000
1	-1.821123000	-1.571568000	-6.135348000
7	3.136571000	-1.438535000	-6.375815000
6	3.873754000	-2.226931000	-7.370815000
6	4.801695000	-3.313509000	-6.815651000
8	5.019503000	-4.340178000	-7.497254000
1	3.209936000	-0.431257000	-6.401772000
1	3.177362000	-2.749493000	-8.032455000
7	5.363081000	-3.086423000	-5.595614000
6	6.229556000	-4.062659000	-4.952459000
6	5.549894000	-4.843161000	-3.825121000
8	6.236205000	-5.296006000	-2.859813000
1	5.291721000	-2.186786000	-5.126558000
1	6.561680000	-4.778699000	-5.713230000
7	4.226223000	-5.058702000	-3.952915000
6	3.459088000	-5.899221000	-3.024750000
6	1.969925000	-5.896822000	-3.383871000
8	1.456462000	-4.531571000	-3.228496000
6	1.159339000	-6.826815000	-2.486854000
1	3.726502000	-4.591926000	-4.705528000
1	3.565491000	-5.523943000	-2.001321000
1	1.840046000	-6.191667000	-4.434076000
1	1.489804000	-4.043377000	-4.097868000
1	0.102915000	-6.780438000	-2.765051000
1	1.502046000	-7.862366000	-2.590433000
1	1.254521000	-6.532012000	-1.436457000
6	-5.469330000	-6.329308000	3.212541000

6	-6.650854000	-5.617623000	3.856718000
8	-7.731525000	-5.453500000	3.223927000
6	-5.945215000	-7.551969000	2.402661000
6	-4.848419000	-8.186423000	1.504167000
6	-4.307733000	-7.175742000	0.514202000
8	-3.352127000	-6.402419000	0.833271000
7	-4.927628000	-7.048439000	-0.674696000
1	-4.968422000	-5.615340000	2.546255000
1	-6.797961000	-7.240010000	1.789949000
1	-6.317407000	-8.324783000	3.087143000
1	-5.267737000	-9.055678000	0.983678000
1	-4.010165000	-8.535941000	2.115826000
1	-4.645182000	-6.268995000	-1.275670000
1	-5.697068000	-7.641285000	-0.942436000
7	-6.477179000	-5.163592000	5.126732000
6	-7.582774000	-4.555999000	5.864970000
6	-8.128463000	-3.239641000	5.304465000
8	-9.290447000	-2.879437000	5.603646000
1	-5.536570000	-5.132100000	5.526171000
1	-8.435595000	-5.239608000	5.893630000
7	-7.285560000	-2.515205000	4.519114000
6	-7.707042000	-1.273760000	3.891824000
6	-7.808755000	-1.339556000	2.368939000
8	-7.848188000	-0.264990000	1.712102000
1	-6.312398000	-2.792995000	4.377856000
1	-8.695861000	-1.021384000	4.290776000
7	-7.892057000	-2.571695000	1.801628000
6	-8.103239000	-2.758063000	0.365113000
6	-6.910911000	-3.353069000	-0.378551000
8	-5.824980000	-2.382941000	-0.451470000
1	-7.903408000	-3.395319000	2.399757000
1	-8.345057000	-1.782265000	-0.061648000
1	-6.559922000	-4.269868000	0.112696000
1	-7.225515000	-3.608859000	-1.398029000
1	-5.159626000	-2.624696000	-1.159448000
6	-9.887702000	4.691708000	-2.828624000
6	-9.065499000	3.515414000	-2.345315000
6	-9.418207000	2.819415000	-1.175899000
6	-7.944354000	3.074638000	-3.071286000
6	-8.681566000	1.708045000	-0.747262000
6	-7.203382000	1.964249000	-2.649178000
6	-7.571180000	1.275497000	-1.485771000
1	-10.655307000	4.373570000	-3.547806000
1	-10.405924000	5.186775000	-1.999617000
1	-10.279325000	3.149856000	-0.599398000
1	-7.650217000	3.600295000	-3.976166000
1	-8.950445000	1.178494000	0.160466000
1	-6.341674000	1.644177000	-3.225539000
1	-7.006128000	0.409726000	-1.152487000
26	-0.310929000	-2.907857000	1.126578000
26	-1.964881000	-2.623604000	-1.623867000
8	0.211405000	-4.959542000	0.801994000
1	1.198937000	-5.009253000	0.742119000
1	-0.273735000	-5.575703000	1.493631000
8	-4.440623000	-2.599393000	3.916983000
1	-4.272498000	-1.732228000	4.379777000
1	-4.355549000	-2.460138000	2.916028000
8	-4.124286000	-1.895275000	1.416888000
1	-4.902900000	-2.046001000	0.770824000
1	-3.988138000	-0.923334000	1.594546000
8	-2.123785000	-3.126045000	0.210483000
1	-3.279821000	-4.476348000	-0.406822000
1	-2.858490000	-2.613677000	0.697154000
8	-0.980472000	-4.106891000	8.748333000
1	-0.581224000	-4.741214000	8.064095000
1	-0.991339000	-4.467091000	9.647597000
8	-3.747708000	-3.251011000	-1.921337000
8	-3.887927000	-4.572952000	-1.195934000
8	-0.476375000	-3.946673000	-1.598590000
1	0.171273000	-4.188924000	-2.334379000
8	-1.199975000	-6.279230000	2.388150000
1	-2.048252000	-6.415504000	1.887383000
1	-1.362334000	-6.123810000	3.352834000
8	-2.529536000	-2.764418000	7.267477000
1	-2.006727000	-3.164222000	8.042517000
1	-2.587854000	-1.770626000	7.216428000
8	-1.965906000	-6.125331000	5.051021000
1	-2.291133000	-7.033411000	5.191678000
1	-2.720190000	-5.411396000	5.261484000
8	-0.061552000	-5.537690000	6.800590000
1	-0.755528000	-5.806981000	6.120671000
1	0.694893000	-5.121551000	6.352197000
8	-3.700468000	-4.396962000	5.612874000
1	-3.995951000	-3.777929000	4.858744000
1	-3.374280000	-3.809210000	6.376765000
1	3.684212000	1.210865000	7.031522000
1	3.646252000	4.605126000	3.536196000
1	-0.314491000	6.405600000	5.811774000
1	-1.333651000	7.278413000	1.126333000
1	-5.290502000	5.378254000	2.149158000
1	-5.044196000	7.258705000	-3.629821000
1	-1.993282000	6.294546000	-7.763803000
1	0.851778000	9.800602000	-5.531506000
1	6.959760000	4.248235000	-4.183634000
1	4.189654000	3.848828000	0.162480000
1	8.353533000	-1.716136000	-2.324506000
1	9.907053000	2.015706000	0.366614000
1	6.303973000	0.774288000	3.517285000
1	9.106868000	-2.737403000	4.189650000
1	4.463272000	-1.531785000	-7.976633000
1	7.101506000	-3.561478000	-4.530041000
1	-7.248728000	-4.387028000	6.893642000
1	-7.021859000	-0.458113000	4.139731000
1	-4.730303000	-6.621536000	3.967157000
1	-8.963102000	-3.425056000	0.221557000
1	8.398256000	-4.928703000	0.604904000
1	-9.264451000	5.438011000	-3.334245000
1	1.891888000	-0.149053000	-4.478066000
1	3.853783000	-6.922944000	-3.053087000
1	-0.359088000	-4.518948000	-0.790266000
1	4.576113000	-2.161970000	3.977005000

^{9}TS2$_{B}$:

6	8.754052000	-3.021834000	3.156196000
6	8.050826000	-1.878758000	2.438202000
8	8.083167000	-1.817746000	1.173681000
6	7.831404000	-4.265077000	3.362631000
6	6.966750000	-4.602633000	2.188152000
7	7.429914000	-4.937015000	0.907310000
6	5.600301000	-4.586416000	2.080358000
6	6.385561000	-5.095906000	0.055959000
7	5.268816000	-4.888456000	0.765062000
1	9.616893000	-3.293788000	2.540523000
1	8.462777000	-5.117029000	3.646383000
1	7.163978000	-4.078121000	4.210311000
1	4.849349000	-4.375527000	2.819808000
1	6.431572000	-5.312697000	-1.013603000
1	4.260923000	-4.784228000	0.393886000
7	7.395885000	-0.970988000	3.203439000
6	6.487623000	0.044827000	2.660327000
6	7.000926000	0.846697000	1.495826000
8	6.204143000	1.228093000	0.591250000
1	7.266110000	-1.116702000	4.204162000
1	5.536381000	-0.389829000	2.340793000
7	8.307419000	1.189796000	1.411805000
6	8.773477000	1.970790000	0.263337000
6	8.361275000	1.367456000	-1.086631000
8	8.101244000	2.117873000	-2.060020000
1	8.946354000	0.924269000	2.146201000
1	8.362464000	2.983370000	0.283038000
7	8.331907000	0.007704000	-1.174747000
6	7.975908000	-0.645664000	-2.428231000
6	6.484947000	-0.649626000	-2.784700000
8	6.139236000	-1.045852000	-3.930526000
1	8.423875000	-0.568330000	-0.337752000
1	8.485000000	-0.150318000	-3.259367000
7	5.617483000	-0.236495000	-1.835256000
6	4.177336000	-0.073876000	-2.093217000
6	3.817024000	1.421346000	-2.116127000
8	3.254531000	1.937518000	-3.124618000

6	3.299436000	-0.839361000	-1.083131000
6	3.497263000	-2.361426000	-1.127352000
6	2.555589000	-3.089798000	-0.179454000
8	1.511745000	-2.469604000	0.208047000
8	2.831813000	-4.313351000	0.186028000
1	5.978566000	0.098069000	-0.943493000
1	4.005516000	-0.444156000	-3.104674000
1	2.249923000	-0.616212000	-1.303314000
1	3.476563000	-0.463747000	-0.068287000
1	4.534416000	-2.633347000	-0.898717000
1	3.281193000	-2.758651000	-2.130110000
7	4.144851000	2.128964000	-1.008714000
6	3.824439000	3.538723000	-0.833048000
6	4.529777000	4.497602000	-1.779404000
8	4.059616000	5.664667000	-1.924321000
1	4.604277000	1.671470000	-0.222548000
1	2.753074000	3.727966000	-0.971296000
7	5.578274000	4.060540000	-2.511734000
6	6.064222000	4.860465000	-3.635572000
6	4.973143000	5.180193000	-4.670752000
8	5.119732000	6.157390000	-5.440099000
1	6.049915000	3.188806000	-2.293528000
1	6.452798000	5.826186000	-3.301957000
7	3.877108000	4.363047000	-4.695289000
6	2.740295000	4.631111000	-5.571832000
6	1.524675000	5.206322000	-4.820904000
8	0.403655000	5.273301000	-5.399486000
6	2.331364000	3.377796000	-6.363732000
1	3.810765000	3.538612000	-4.096368000
1	3.070939000	5.413441000	-6.267376000
1	1.436294000	3.585523000	-6.953667000
1	3.147335000	3.082957000	-7.030530000
1	2.114994000	2.550482000	-5.678641000
7	1.704784000	5.695372000	-3.563508000
6	0.556829000	6.146498000	-2.768922000
6	-0.155288000	7.363115000	-3.377464000
8	-1.352102000	7.606794000	-3.066460000
6	0.949049000	6.443555000	-1.312584000
8	0.864175000	5.198715000	-0.556212000
1	2.613954000	5.637576000	-3.099410000
1	-0.206328000	5.360333000	-2.752730000
1	1.962911000	6.853960000	-1.263974000
1	0.237770000	7.175709000	-0.910285000
1	1.054776000	5.345993000	0.402058000
7	0.556088000	8.135337000	-4.234730000
6	-0.049320000	9.250977000	-4.954643000
6	-1.108379000	8.868032000	-5.999215000
8	-1.799701000	9.777980000	-6.510166000
1	1.508395000	7.865306000	-4.440564000
1	-0.551349000	9.921805000	-4.251638000
7	-1.247421000	7.545332000	-6.297297000
6	-2.322678000	7.075961000	-7.159869000
6	-3.525302000	6.482679000	-6.420225000
8	-4.418620000	5.885550000	-7.074763000
1	-0.624176000	6.839813000	-5.904860000
1	-2.681085000	7.923212000	-7.752703000
7	-3.568388000	6.653061000	-5.071798000
6	-4.709310000	6.223988000	-4.263978000
6	-4.354017000	5.170116000	-3.201429000
6	-3.966012000	3.784763000	-3.679598000
6	-4.165002000	3.332780000	-5.007729000
6	-3.429320000	2.874272000	-2.731989000
6	-3.853882000	2.028880000	-5.363034000
6	-3.121336000	1.566647000	-3.062698000
6	-3.335264000	1.115117000	-4.399593000
8	-3.080806000	-0.132866000	-4.776084000
1	-2.805185000	7.124110000	-4.594546000
1	-5.473623000	5.859098000	-4.954313000
1	-5.229651000	5.062578000	-2.539509000
1	-3.544388000	5.570568000	-2.572955000
1	-4.545349000	4.009119000	-5.766712000
1	-3.274245000	3.213322000	-1.711081000
1	-3.998877000	1.671732000	-6.375998000
1	-2.717728000	0.870693000	-2.333604000
1	-2.478360000	-0.913153000	-4.140139000
6	-4.721347000	4.507652000	2.470869000
6	-3.248438000	4.787754000	2.311348000
8	-2.367821000	3.908620000	2.526535000
6	-5.246808000	3.449196000	1.442537000
6	-5.982820000	2.283119000	2.126032000
6	-4.988215000	1.369130000	2.849550000
8	-4.064100000	0.793521000	2.210587000
8	-5.186878000	1.314793000	4.152175000
1	-4.856915000	4.107229000	3.483591000
1	-5.926872000	3.933461000	0.734048000
1	-4.414174000	3.031964000	0.866634000
1	-6.496983000	1.657941000	1.390511000
1	-6.739505000	2.645983000	2.826951000
7	-2.858217000	6.049726000	1.944218000
6	-1.465695000	6.309946000	1.564930000
6	-0.449531000	5.958194000	2.649830000
8	0.686810000	5.510302000	2.326292000
1	-3.561684000	6.728197000	1.688779000
1	-1.179335000	5.726211000	0.683913000
7	-0.790126000	6.186687000	3.942726000
6	0.115529000	5.860095000	5.048338000
6	0.220919000	4.356961000	5.366510000
8	1.295148000	3.896673000	5.826262000
1	-1.707358000	6.563011000	4.139076000
1	1.124558000	6.200766000	4.810250000
7	-0.892761000	3.618273000	5.146687000
6	-0.944141000	2.148041000	5.313688000
6	0.048899000	1.507494000	4.320557000
8	0.957660000	0.729486000	4.711420000
6	-2.405250000	1.714883000	5.085653000
6	-2.802720000	0.368319000	5.667555000
8	-2.145998000	-0.173064000	6.608044000
8	-3.888386000	-0.176653000	5.171139000
1	-1.639069000	4.023806000	4.594931000
1	-0.608176000	1.873350000	6.314900000
1	-2.645244000	1.701109000	4.021441000
1	-3.069436000	2.455201000	5.551624000
7	-0.074637000	1.904086000	3.027359000
6	0.832174000	1.413779000	1.982303000
6	2.277120000	1.855728000	2.250456000
8	3.249817000	1.109827000	1.956558000
6	0.365088000	1.887659000	0.585665000
6	-1.017190000	1.369689000	0.116623000
6	-1.089406000	-0.140514000	-0.027475000
8	-0.719677000	-0.837097000	0.977009000
8	-1.526123000	-0.636176000	-1.149811000
1	-0.859416000	2.488106000	2.753660000
1	0.835303000	0.323528000	1.998794000
1	1.127086000	1.569211000	-0.135091000
1	0.345247000	2.983447000	0.551138000
1	-1.805783000	1.663616000	0.822326000
1	-1.242676000	1.833249000	-0.846509000
7	2.456032000	3.089808000	2.799007000
6	3.771289000	3.585320000	3.185539000
6	4.491809000	2.708909000	4.211892000
8	5.738123000	2.543232000	4.116562000
1	1.665760000	3.713976000	2.906398000
1	4.452556000	3.638932000	2.331586000
7	3.751416000	2.145274000	5.195632000
6	4.358990000	1.195774000	6.128855000
6	4.552279000	-0.211852000	5.537993000
8	5.683779000	-0.784052000	5.585615000
1	2.768604000	2.383278000	5.297830000
1	5.348825000	1.548336000	6.422524000
7	3.462975000	-0.798218000	4.994951000
6	3.553785000	-2.133743000	4.389990000
6	2.457890000	-2.387779000	3.349216000
6	1.092654000	-2.599134000	3.928391000
7	-0.026561000	-2.820461000	3.117829000
6	0.674335000	-2.587641000	5.245185000
6	-1.085095000	-2.921210000	3.935705000
7	-0.695702000	-2.790364000	5.224831000
1	2.585182000	-0.281172000	4.903185000
1	3.522803000	-2.901013000	5.177871000
1	2.735029000	-3.272087000	2.758738000

1	2.438193000	-1.550460000	2.640839000
1	1.203157000	-2.383674000	6.159898000
1	-2.107972000	-3.058215000	3.631050000
6	1.538540000	-1.049882000	-4.860896000
6	2.393950000	-2.025447000	-5.641822000
8	2.254715000	-3.281152000	-5.538783000
6	0.224556000	-0.657281000	-5.595777000
6	-0.740598000	-1.825543000	-5.901966000
6	-1.172729000	-2.600102000	-4.663063000
8	-1.809477000	-1.870451000	-3.702793000
8	-0.928457000	-3.806499000	-4.523310000
1	1.288783000	-1.519022000	-3.903470000
1	-0.289534000	0.079238000	-4.970061000
1	0.471877000	-0.153308000	-6.539406000
1	-0.284936000	-2.547494000	-6.584153000
1	-1.633693000	-1.408373000	-6.381404000
7	3.316071000	-1.510709000	-6.501394000
6	4.044773000	-2.352302000	-7.456582000
6	4.967201000	-3.417518000	-6.852957000
8	5.220305000	-4.450779000	-7.512133000
1	3.437386000	-0.508965000	-6.543054000
1	3.341818000	-2.900951000	-8.089647000
7	5.480744000	-3.164342000	-5.617716000
6	6.331215000	-4.121324000	-4.927797000
6	5.623484000	-4.890942000	-3.810726000
8	6.297477000	-5.361071000	-2.844742000
1	5.374291000	-2.259507000	-5.164840000
1	6.696928000	-4.847107000	-5.663422000
7	4.297279000	-5.078331000	-3.945470000
6	3.510555000	-5.906098000	-3.021180000
6	2.026239000	-5.891611000	-3.397638000
8	1.524057000	-4.521142000	-3.249132000
6	1.199487000	-6.813195000	-2.506224000
1	3.808122000	-4.599595000	-4.697546000
1	3.606632000	-5.525902000	-1.998714000
1	1.907770000	-6.189126000	-4.448623000
1	1.502974000	-4.060998000	-4.127905000
1	0.147626000	-6.770258000	-2.801727000
1	1.543252000	-7.850213000	-2.592222000
1	1.274037000	-6.505569000	-1.458278000
6	-5.579553000	-6.281489000	3.238371000
6	-6.728383000	-5.541244000	3.908504000
8	-7.818254000	-5.349401000	3.298316000
6	-6.106511000	-7.499224000	2.451798000
6	-5.039510000	-8.191387000	1.563224000
6	-4.413304000	-7.207586000	0.593024000
8	-3.346814000	-6.592758000	0.888321000
7	-5.075178000	-6.933477000	-0.549386000
1	-5.081998000	-5.582864000	2.553002000
1	-6.948442000	-7.163017000	1.837021000
1	-6.509160000	-8.242359000	3.152040000
1	-5.506183000	-9.024931000	1.023869000
1	-4.234708000	-8.602044000	2.181018000
1	-4.733571000	-6.150135000	-1.119705000
1	-5.931849000	-7.404302000	-0.793901000
7	-6.520182000	-5.097062000	5.176541000
6	-7.598440000	-4.472099000	5.940950000
6	-8.152760000	-3.158923000	5.380268000
8	-9.314000000	-2.800578000	5.686603000
1	-5.570984000	-5.087519000	5.557389000
1	-8.455341000	-5.148280000	6.005355000
7	-7.316492000	-2.435514000	4.588002000
6	-7.745533000	-1.201534000	3.951345000
6	-7.839354000	-1.281333000	2.427729000
8	-7.863527000	-0.213071000	1.757374000
1	-6.340382000	-2.709259000	4.445786000
1	-8.737756000	-0.954122000	4.345343000
7	-7.935278000	-2.518165000	1.877316000
6	-8.132484000	-2.730468000	0.442328000
6	-6.930933000	-3.336490000	-0.279759000
8	-5.849177000	-2.368482000	-0.377559000
1	-7.951671000	-3.334297000	2.486490000
1	-8.376344000	-1.764504000	-0.004976000
1	-6.582897000	-4.239621000	0.239298000
1	-7.241325000	-3.624610000	-1.292547000
1	-5.171212000	-2.639901000	-1.082859000
6	-9.508525000	4.654441000	-3.021196000
6	-8.790339000	3.440508000	-2.469545000
6	-9.144198000	2.896987000	-1.221985000
6	-7.775704000	2.806413000	-3.208698000
6	-8.512404000	1.748771000	-0.728051000
6	-7.139232000	1.659138000	-2.718985000
6	-7.503555000	1.125421000	-1.476418000
1	-10.380316000	4.362408000	-3.622979000
1	-9.874099000	5.304998000	-2.218537000
1	-9.928215000	3.373583000	-0.637893000
1	-7.489238000	3.208680000	-4.177379000
1	-8.789679000	1.328092000	0.232590000
1	-6.361053000	1.183697000	-3.307864000
1	-7.014780000	0.236209000	-1.089230000
26	-0.363954000	-2.924818000	1.023808000
26	-2.060589000	-2.555324000	-1.718144000
8	0.188382000	-4.992757000	0.741139000
1	1.174055000	-5.040561000	0.685839000
1	-0.279514000	-5.605638000	1.440494000
8	-4.495046000	-2.549736000	4.011010000
1	-4.305948000	-1.698239000	4.489545000
1	-4.421385000	-2.387217000	3.005639000
8	-4.220697000	-1.832372000	1.544879000
1	-4.962525000	-1.995942000	0.860457000
1	-4.057085000	-0.863806000	1.688066000
8	-2.205877000	-3.147834000	0.283015000
1	-3.256682000	-4.340043000	-0.407209000
1	-2.899051000	-2.628694000	0.790624000
8	-0.836638000	-4.136912000	8.662269000
1	-0.467261000	-4.766209000	7.954218000
1	-0.818311000	-4.512198000	9.555427000
8	-3.914402000	-3.241890000	-1.935091000
8	-3.873544000	-4.560987000	-1.179250000
8	-0.535816000	-3.935220000	-1.661487000
1	0.124433000	-4.166140000	-2.375131000
8	-1.156934000	-6.358455000	2.370312000
1	-1.997864000	-6.556917000	1.878892000
1	-1.344034000	-6.140425000	3.316380000
8	-2.489668000	-2.795668000	7.284217000
1	-1.912171000	-3.207280000	8.013230000
1	-2.535442000	-1.801830000	7.255301000
8	-1.990621000	-6.124649000	5.023655000
1	-2.322038000	-7.034573000	5.131931000
1	-2.740178000	-5.414977000	5.245020000
8	-0.009041000	-5.561439000	6.676850000
1	-0.743306000	-5.830323000	6.038139000
1	0.709565000	-5.127569000	6.184507000
8	-3.721157000	-4.396490000	5.632069000
1	-4.022272000	-3.753178000	4.898618000
1	-3.373610000	-3.830153000	6.400027000
1	3.718889000	1.134048000	7.013856000
1	3.639816000	4.593763000	3.588879000
1	-0.224737000	6.391940000	5.941914000
1	-1.374669000	7.372525000	1.316480000
1	-5.305250000	5.432701000	2.408891000
1	-5.125138000	7.099376000	-3.748497000
1	-1.951833000	6.309676000	-7.845594000
1	0.742350000	9.817483000	-5.451826000
1	6.878298000	4.309217000	-4.113892000
1	4.069019000	3.811026000	0.196672000
1	8.317614000	-1.684249000	-2.391884000
1	9.864616000	2.037704000	0.312464000
1	6.269971000	0.758639000	3.463000000
1	9.131056000	-2.706057000	4.134840000
1	4.638049000	-1.694211000	-8.098911000
1	7.185813000	-3.607340000	-4.484360000
1	-7.229985000	-4.293208000	6.956225000
1	-7.066911000	-0.379651000	4.196453000
1	-4.826558000	-6.583360000	3.975132000
1	-8.988597000	-3.404047000	0.306685000
1	8.396755000	-4.967424000	0.623813000
1	-8.855433000	5.249231000	-3.669864000
1	2.100216000	-0.133746000	-4.638321000
1	3.897244000	-6.932916000	-3.039459000

1	-0.433700000	-4.528275000	-0.870324000
1	4.538966000	-2.210529000	3.918269000
1	-1.336935000	-2.809578000	6.041177000

$^{9}IM2_{B}$:

6	8.718566000	-2.873856000	2.643819000
6	7.941880000	-1.723509000	2.019570000
8	7.857851000	-1.643707000	0.758554000
6	7.878189000	-4.187480000	2.762340000
6	6.958493000	-4.453593000	1.611814000
7	7.352063000	-4.698516000	0.287959000
6	5.588826000	-4.431762000	1.578970000
6	6.263671000	-4.798651000	-0.515892000
7	5.188503000	-4.642346000	0.266062000
1	9.584184000	-3.047790000	1.996740000
1	8.571111000	-5.024349000	2.916776000
1	7.256618000	-4.132525000	3.662078000
1	4.878484000	-4.273546000	2.370044000
1	6.234287000	-4.945661000	-1.597308000
1	4.165959000	-4.529598000	-0.034972000
7	7.361282000	-0.824273000	2.852202000
6	6.433053000	0.217674000	2.394999000
6	6.887553000	1.039386000	1.217222000
8	6.040761000	1.477602000	0.387300000
1	7.316352000	-0.984980000	3.857558000
1	5.458168000	-0.197383000	2.123678000
7	8.199393000	1.330122000	1.048984000
6	8.625374000	2.109693000	-0.114759000
6	8.102896000	1.552833000	-1.446534000
8	7.831371000	2.338973000	-2.388764000
1	8.874930000	1.013525000	1.728187000
1	8.263613000	3.139289000	-0.053039000
7	7.983276000	0.200817000	-1.554855000
6	7.512842000	-0.410726000	-2.792546000
6	5.997056000	-0.401422000	-3.024820000
8	5.552662000	-0.858612000	-4.110454000
1	8.099907000	-0.396546000	-0.735855000
1	7.954024000	0.112086000	-3.645819000
7	5.214851000	0.100373000	-2.041932000
6	3.763838000	0.298526000	-2.212201000
6	3.459114000	1.802288000	-2.110458000
8	2.955912000	2.440515000	-3.078468000
6	2.923888000	-0.530763000	-1.216032000
6	3.158435000	-2.043999000	-1.329711000
6	2.346412000	-2.846292000	-0.321245000
8	1.335387000	-2.288218000	0.223199000
8	2.702552000	-4.075487000	-0.061711000
1	5.646163000	0.453682000	-1.188927000
1	3.531717000	0.010750000	-3.239220000
1	1.864533000	-0.320904000	-1.402195000
1	3.122367000	-0.203517000	-0.189107000
1	4.220718000	-2.284354000	-1.204532000
1	2.879310000	-2.410467000	-2.328903000
7	3.788743000	2.402380000	-0.941374000
6	3.541309000	3.815355000	-0.697281000
6	4.375773000	4.786560000	-1.517230000
8	4.003447000	5.995965000	-1.587239000
1	4.218207000	1.875623000	-0.183356000
1	2.496368000	4.073077000	-0.905409000
7	5.442719000	4.326986000	-2.204254000
6	6.117859000	5.172819000	-3.186904000
6	5.182734000	5.696788000	-4.284329000
8	5.499864000	6.722063000	-4.929083000
1	5.798821000	3.388821000	-2.062503000
1	6.552718000	6.059153000	-2.717147000
7	4.022149000	5.005206000	-4.499780000
6	3.016220000	5.480406000	-5.447541000
6	1.723281000	5.926466000	-4.741584000
8	0.642463000	6.020262000	-5.387177000
6	2.722553000	4.443069000	-6.542579000
1	3.826511000	4.134003000	-4.009390000
1	3.450737000	6.378093000	-5.908552000
1	1.911715000	4.792568000	-7.185234000
1	3.621531000	4.287526000	-7.145805000
1	2.426094000	3.483420000	-6.107717000
7	1.793861000	6.282429000	-3.425653000
6	0.567632000	6.547285000	-2.663134000
6	-0.223312000	7.759029000	-3.169599000
8	-1.454097000	7.842828000	-2.910190000
6	0.835296000	6.679731000	-1.152620000
8	0.622674000	5.369091000	-0.537965000
1	2.669571000	6.188750000	-2.905948000
1	-0.122088000	5.707162000	-2.794276000
1	1.858519000	7.023458000	-0.970899000
1	0.124824000	7.401571000	-0.731834000
1	0.776004000	5.408300000	0.438438000
7	0.442960000	8.697838000	-3.883261000
6	-0.249518000	9.827963000	-4.496704000
6	-1.259063000	9.464894000	-5.596865000
8	-2.074442000	10.337730000	-5.971804000
1	1.426185000	8.549718000	-4.065174000
1	-0.818880000	10.376621000	-3.741572000
7	-1.209129000	8.199481000	-6.098998000
6	-2.208061000	7.716202000	-7.043311000
6	-3.106770000	6.609983000	-6.488035000
8	-3.749727000	5.873234000	-7.278515000
1	-0.496283000	7.534768000	-5.797262000
1	-2.830366000	8.569591000	-7.332689000
7	-3.171723000	6.493362000	-5.132591000
6	-3.981477000	5.466894000	-4.481591000
6	-3.142158000	4.431503000	-3.705255000
6	-2.136064000	3.628249000	-4.503156000
6	-2.326380000	3.312657000	-5.876301000
6	-0.970603000	3.141648000	-3.846820000
6	-1.415655000	2.524325000	-6.551483000
6	-0.042804000	2.359631000	-4.503546000
6	-0.249062000	1.997898000	-5.886069000
8	0.575817000	1.216119000	-6.513762000
1	-2.630126000	7.117248000	-4.540717000
1	-4.584194000	4.994871000	-5.261373000
1	-3.844718000	3.731751000	-3.220837000
1	-2.616414000	4.946772000	-2.890365000
1	-3.171930000	3.726543000	-6.414460000
1	-0.803857000	3.408571000	-2.806461000
1	-1.549292000	2.278932000	-7.599213000
1	0.870683000	2.034250000	-4.016643000
1	-3.508249000	-3.584707000	-3.089373000
6	-4.875528000	4.239460000	2.659490000
6	-3.426883000	4.595442000	2.437874000
8	-2.495032000	3.759325000	2.605219000
6	-5.387524000	3.137853000	1.672925000
6	-6.098497000	1.985813000	2.407332000
6	-5.075040000	1.113130000	3.142573000
8	-4.170909000	0.508179000	2.502197000
8	-5.222811000	1.128578000	4.453015000
1	-4.946210000	3.848805000	3.683043000
1	-6.077312000	3.583517000	0.948683000
1	-4.552140000	2.714465000	1.106444000
1	-6.613075000	1.328105000	1.701383000
1	-6.847275000	2.361987000	3.110014000
7	-3.116058000	5.880829000	2.078461000
6	-1.751464000	6.225673000	1.664996000
6	-0.688852000	5.890283000	2.710553000
8	0.448889000	5.477836000	2.347553000
1	-3.862521000	6.525016000	1.858732000
1	-1.462903000	5.693473000	0.752696000
7	-0.991184000	6.101108000	4.016477000
6	-0.033903000	5.807739000	5.087650000
6	0.141916000	4.309799000	5.398224000
8	1.251504000	3.889989000	5.810897000
1	-1.910805000	6.451114000	4.247392000
1	0.951975000	6.187988000	4.814733000
7	-0.951443000	3.530014000	5.224329000
6	-0.946042000	2.058727000	5.391807000
6	0.029086000	1.451840000	4.360071000
8	0.980598000	0.706102000	4.709786000
6	-2.398009000	1.573666000	5.215790000
6	-2.747344000	0.254503000	5.884052000
8	-2.019307000	-0.252353000	6.790113000
8	-3.867705000	-0.307484000	5.493204000

1	-1.736798000	3.911772000	4.712005000
1	-0.565482000	1.795586000	6.380123000
1	-2.656293000	1.483287000	4.158641000
1	-3.079618000	2.319627000	5.645159000
7	-0.163633000	1.845919000	3.073888000
6	0.717057000	1.391299000	1.993114000
6	2.156395000	1.885157000	2.214307000
8	3.144030000	1.184395000	1.860413000
6	0.176832000	1.844932000	0.613978000
6	-1.205691000	1.275988000	0.193386000
6	-1.264325000	-0.237549000	0.139489000
8	-0.925387000	-0.879819000	1.187434000
8	-1.673518000	-0.808997000	-0.965068000
1	-0.979223000	2.402370000	2.834122000
1	0.765701000	0.301808000	2.010602000
1	0.923597000	1.557455000	-0.133600000
1	0.113263000	2.940118000	0.586264000
1	-1.982882000	1.597222000	0.899050000
1	-1.464995000	1.676523000	-0.790180000
7	2.310936000	3.107981000	2.790496000
6	3.616588000	3.650380000	3.151498000
6	4.414320000	2.783783000	4.126361000
8	5.657368000	2.655712000	3.962694000
1	1.499635000	3.693244000	2.945556000
1	4.267653000	3.759381000	2.280426000
7	3.746740000	2.190589000	5.144471000
6	4.445517000	1.263474000	6.035288000
6	4.679854000	-0.122551000	5.411974000
8	5.834270000	-0.645871000	5.401258000
1	2.762497000	2.391910000	5.298566000
1	5.429208000	1.662600000	6.287004000
7	3.593770000	-0.748220000	4.905026000
6	3.715093000	-2.076985000	4.291867000
6	2.554625000	-2.383378000	3.342330000
6	1.242800000	-2.612708000	4.027037000
7	0.070378000	-2.821585000	3.295771000
6	0.917881000	-2.619305000	5.370044000
6	-0.929923000	-2.931981000	4.182798000
7	-0.450616000	-2.821548000	5.442173000
1	2.693743000	-0.266575000	4.851528000
1	3.786182000	-2.843119000	5.078091000
1	2.809914000	-3.270532000	2.745182000
1	2.457265000	-1.559523000	2.624498000
1	1.510647000	-2.427944000	6.247511000
1	-1.971063000	-3.066919000	3.946308000
6	0.035029000	-1.622644000	-4.770639000
6	1.126374000	-2.202668000	-5.643964000
8	1.327855000	-3.464804000	-5.693413000
6	-1.324138000	-1.536847000	-5.522100000
6	-1.919979000	-2.884245000	-5.960532000
6	-2.564382000	-3.743145000	-4.888793000
8	-2.942855000	-3.050316000	-3.764958000
8	-2.797017000	-4.946675000	-5.035185000
1	-0.066292000	-2.251022000	-3.881405000
1	-2.034846000	-1.023832000	-4.867567000
1	-1.183863000	-0.907249000	-6.408447000
1	-1.181598000	-3.509771000	-6.472513000
1	-2.715077000	-2.690252000	-6.695909000
7	1.844100000	-1.352330000	-6.405225000
6	2.737768000	-1.818565000	-7.475800000
6	3.772389000	-2.877011000	-7.078860000
8	4.019253000	-3.824911000	-7.857172000
1	1.584617000	-0.360565000	-6.388794000
1	2.161444000	-2.270737000	-8.288491000
7	4.412742000	-2.706153000	-5.885089000
6	5.398982000	-3.660774000	-5.398788000
6	4.901222000	-4.520667000	-4.235973000
8	5.716093000	-4.947356000	-3.362061000
1	4.341113000	-1.843388000	-5.355174000
1	5.665180000	-4.321918000	-6.231661000
7	3.589491000	-4.831905000	-4.230797000
6	2.993354000	-5.738080000	-3.238477000
6	1.552367000	-6.116717000	-3.608097000
8	0.710331000	-4.920841000	-3.599149000
6	0.956630000	-7.115855000	-2.620678000
1	2.982126000	-4.398725000	-4.925230000
1	2.981544000	-5.265841000	-2.248767000
1	1.531494000	-6.537474000	-4.622959000
1	0.790102000	-4.399980000	-4.450438000
1	-0.082112000	-7.325292000	-2.889256000
1	1.518544000	-8.056338000	-2.631704000
1	0.972324000	-6.714139000	-1.601307000
6	-5.321637000	-6.494706000	3.672600000
6	-6.483622000	-5.777662000	4.344236000
8	-7.592460000	-5.651263000	3.750937000
6	-5.805229000	-7.803701000	3.013763000
6	-4.733491000	-8.497934000	2.133680000
6	-4.190111000	-7.548945000	1.081525000
8	-3.095758000	-6.938137000	1.251018000
7	-4.957715000	-7.302911000	-0.000497000
1	-4.911931000	-5.823079000	2.906744000
1	-6.693058000	-7.570221000	2.415982000
1	-6.128379000	-8.508839000	3.790296000
1	-5.173230000	-9.386003000	1.662371000
1	-3.888628000	-8.829223000	2.745030000
1	-4.666244000	-6.543355000	-0.625436000
1	-5.832957000	-7.778647000	-0.151462000
7	-6.268370000	-5.287429000	5.593348000
6	-7.356452000	-4.689974000	6.367199000
6	-8.001150000	-3.432907000	5.773546000
8	-9.178395000	-3.138834000	6.086380000
1	-5.313093000	-5.239936000	5.954475000
1	-8.172394000	-5.408117000	6.486706000
7	-7.222686000	-2.684489000	4.946069000
6	-7.735342000	-1.497807000	4.280401000
6	-7.870184000	-1.636494000	2.762702000
8	-7.944474000	-0.597274000	2.051635000
1	-6.231779000	-2.903276000	4.806075000
1	-8.727790000	-1.288998000	4.695805000
7	-7.949657000	-2.898406000	2.268445000
6	-8.174896000	-3.181233000	0.851233000
6	-6.988454000	-3.840483000	0.152194000
8	-5.890087000	-2.898150000	0.021070000
1	-7.910873000	-3.688348000	2.910505000
1	-8.408969000	-2.235578000	0.357696000
1	-6.659978000	-4.727599000	0.710801000
1	-7.305073000	-4.162809000	-0.848622000
1	-5.218127000	-3.177840000	-0.689034000
6	-8.073406000	3.954123000	-3.303684000
6	-7.535593000	2.684720000	-2.676762000
6	-8.111309000	2.156226000	-1.507717000
6	-6.470997000	1.982718000	-3.270790000
6	-7.647914000	0.956692000	-0.950946000
6	-6.000604000	0.785285000	-2.716939000
6	-6.587518000	0.267128000	-1.555430000
1	-8.823741000	3.731241000	-4.074646000
1	-8.556482000	4.596000000	-2.558637000
1	-8.938833000	2.683107000	-1.038099000
1	-6.021323000	2.368422000	-4.183214000
1	-8.101393000	0.551465000	-0.050812000
1	-5.182016000	0.253602000	-3.194252000
1	-6.238673000	-0.665703000	-1.122896000
26	-0.381486000	-2.956341000	1.249024000
26	-2.156708000	-2.720005000	-1.261137000
8	0.273828000	-4.963993000	0.799579000
1	1.250235000	-4.940270000	0.640692000
1	-0.093412000	-5.613160000	1.528423000
8	-4.401049000	-2.718442000	4.384701000
1	-4.236099000	-1.846375000	4.834368000
1	-4.388299000	-2.588499000	3.371527000
8	-4.303926000	-2.120854000	1.886411000
1	-5.040956000	-2.374031000	1.226750000
1	-4.176409000	-1.139657000	1.965017000
8	-2.234628000	-3.388418000	0.703072000
1	-3.158296000	-4.695764000	-0.079305000
1	-2.961875000	-2.927778000	1.217208000
8	-0.510908000	-4.177919000	8.908560000
1	-0.148122000	-4.799393000	8.191035000
1	-0.490423000	-4.566157000	9.796181000
8	-3.964423000	-3.647015000	-1.566111000

8	-3.740589000	-4.982829000	-0.854399000
8	-0.673874000	-4.079966000	-1.574718000
1	-0.191464000	-4.412949000	-2.395835000
8	-0.830704000	-6.415411000	2.536489000
1	-1.675250000	-6.720431000	2.110498000
1	-0.999715000	-6.150600000	3.473505000
8	-2.189405000	-2.873841000	7.534935000
1	-1.604022000	-3.260387000	8.270970000
1	-2.290867000	-1.885104000	7.500917000
8	-1.655268000	-6.149381000	5.214161000
1	-1.949798000	-7.074673000	5.298765000
1	-2.429282000	-5.481462000	5.476373000
8	0.299791000	-5.583601000	6.899969000
1	-0.426453000	-5.845915000	6.249065000
1	1.040433000	-5.177844000	6.417421000
8	-3.445138000	-4.524250000	5.941028000
1	-3.823198000	-3.886514000	5.237201000
1	-3.077582000	-3.950119000	6.691951000
1	3.852538000	1.156899000	6.948364000
1	3.451607000	4.639392000	3.588678000
1	-0.362228000	6.324674000	5.994370000
1	-1.723512000	7.299982000	1.454097000
1	-5.511104000	5.130704000	2.610782000
1	-4.673277000	5.942972000	-3.775493000
1	-1.736956000	7.317833000	-7.946285000
1	0.497531000	10.507745000	-4.915495000
1	6.926154000	4.583721000	-3.627449000
1	3.718992000	4.011835000	0.361364000
1	7.847836000	-1.451258000	-2.823722000
1	9.719358000	2.128531000	-0.135103000
1	6.271162000	0.911290000	3.228212000
1	9.095149000	-2.613330000	3.638429000
1	3.253982000	-0.937792000	-7.871266000
1	6.289381000	-3.131520000	-5.058994000
1	-6.967217000	-4.449884000	7.361893000
1	-7.094591000	-0.634320000	4.478792000
1	-4.512750000	-6.687811000	4.385266000
1	-9.042803000	-3.847287000	0.758630000
1	8.302875000	-4.706141000	-0.046410000
1	-7.279858000	4.534846000	-3.788145000
1	0.311464000	-0.617235000	-4.440190000
1	3.612624000	-6.640812000	-3.173566000
1	-0.428987000	-4.601919000	-0.759577000
1	4.662521000	-2.104781000	3.743371000

^{9}TS3$_{B}$:

6	8.615337000	-2.751134000	2.476744000
6	7.842325000	-1.594550000	1.859733000
8	7.765952000	-1.501691000	0.599254000
6	7.771537000	-4.063796000	2.582802000
6	6.849411000	-4.314934000	1.430841000
7	7.240052000	-4.550941000	0.104510000
6	5.479906000	-4.284739000	1.399655000
6	6.150187000	-4.638144000	-0.699051000
7	5.076680000	-4.482002000	0.085524000
1	9.481827000	-2.921776000	1.830067000
1	8.462298000	-4.904162000	2.727390000
1	7.151314000	-4.016623000	3.483908000
1	4.771415000	-4.128917000	2.192848000
1	6.122042000	-4.776802000	-1.782421000
1	4.052835000	-4.361529000	-0.211439000
7	7.255793000	-0.703728000	2.697501000
6	6.328389000	0.340325000	2.244060000
6	6.788107000	1.174876000	1.077713000
8	5.944654000	1.621619000	0.248852000
1	7.205500000	-0.873805000	3.700780000
1	5.356555000	-0.074466000	1.961561000
7	8.100116000	1.468177000	0.916838000
6	8.528219000	2.259166000	-0.238574000
6	8.012214000	1.710967000	-1.576569000
8	7.739849000	2.503194000	-2.513559000
1	8.774060000	1.144920000	1.594475000
1	8.162418000	3.286817000	-0.169497000
7	7.899642000	0.359392000	-1.695531000
6	7.436907000	-0.245980000	-2.939201000
6	5.922650000	-0.235152000	-3.179965000
8	5.484735000	-0.680702000	-4.272798000
1	8.015628000	-0.243519000	-0.880548000
1	7.882811000	0.280867000	-3.787513000
7	5.134341000	0.255710000	-2.196120000
6	3.685786000	0.460731000	-2.375840000
6	3.384941000	1.965276000	-2.274725000
8	2.873567000	2.601372000	-3.239621000
6	2.834618000	-0.366357000	-1.387245000
6	3.060538000	-1.880892000	-1.503836000
6	2.231597000	-2.681563000	-0.507220000
8	1.205300000	-2.126576000	0.010890000
8	2.590940000	-3.906575000	-0.231188000
1	5.560953000	0.600246000	-1.337495000
1	3.459447000	0.175833000	-3.404958000
1	1.777724000	-0.150250000	-1.579682000
1	3.028896000	-0.042810000	-0.358359000
1	4.119741000	-2.128145000	-1.367731000
1	2.790180000	-2.241372000	-2.507684000
7	3.723905000	2.569582000	-1.110182000
6	3.473980000	3.983388000	-0.872367000
6	4.297650000	4.952503000	-1.706069000
8	3.916655000	6.158650000	-1.784615000
1	4.162819000	2.046186000	-0.355093000
1	2.426531000	4.235353000	-1.074978000
7	5.364917000	4.494576000	-2.393650000
6	6.032515000	5.339233000	-3.382509000
6	5.093521000	5.850234000	-4.482953000
8	5.405534000	6.872117000	-5.135573000
1	5.730794000	3.560863000	-2.245289000
1	6.461928000	6.231360000	-2.918699000
7	3.936644000	5.151129000	-4.692522000
6	2.928567000	5.612008000	-5.645199000
6	1.636864000	6.069539000	-4.944247000
8	0.557811000	6.164419000	-5.592557000
6	2.632437000	4.557173000	-6.722885000
1	3.745415000	4.283777000	-4.193898000
1	3.361869000	6.502364000	-6.121122000
1	1.819531000	4.896403000	-7.368447000
1	3.529686000	4.393113000	-7.326484000
1	2.337851000	3.604020000	-6.272703000
7	1.707034000	6.433163000	-3.630240000
6	0.480437000	6.702390000	-2.870080000
6	-0.301285000	7.923213000	-3.368791000
8	-1.527568000	8.022236000	-3.092626000
6	0.741597000	6.818072000	-1.357502000
8	0.515754000	5.503011000	-0.755645000
1	2.582090000	6.340415000	-3.109526000
1	-0.214339000	5.868735000	-3.013906000
1	1.766688000	7.151379000	-1.167297000
1	0.034644000	7.541248000	-0.933415000
1	0.660806000	5.534465000	0.222289000
7	0.364538000	8.848483000	-4.099661000
6	-0.326308000	9.976501000	-4.718604000
6	-1.352373000	9.605335000	-5.800655000
8	-2.169411000	10.476997000	-6.174692000
1	1.344096000	8.689450000	-4.291723000
1	-0.881670000	10.539704000	-3.963821000
7	-1.313905000	8.334022000	-6.289050000
6	-2.326292000	7.844854000	-7.215709000
6	-3.231827000	6.757933000	-6.634085000
8	-3.889081000	6.014759000	-7.406412000
1	-0.597992000	7.670622000	-5.991491000
1	-2.942748000	8.699309000	-7.514338000
7	-3.287573000	6.665048000	-5.276056000
6	-4.105408000	5.658863000	-4.603739000
6	-3.274144000	4.632282000	-3.806755000
6	-2.262427000	3.819327000	-4.587665000
6	-2.448862000	3.478726000	-5.955281000
6	-1.094380000	3.353580000	-3.921373000
6	-1.528759000	2.690318000	-6.617554000
6	-0.157312000	2.571657000	-4.565471000
6	-0.356334000	2.189043000	-5.943130000
8	0.482181000	1.412683000	-6.558935000
1	-2.734233000	7.292239000	-4.698686000

1	-4.711142000	5.176045000	-5.374566000
1	-3.981738000	3.939973000	-3.319027000
1	-2.753197000	5.157239000	-2.995020000
1	-3.298822000	3.874169000	-6.500302000
1	-0.930935000	3.639512000	-2.885419000
1	-1.658550000	2.426984000	-7.661379000
1	0.759369000	2.261871000	-4.074728000
1	-3.767890000	-3.464702000	-2.862799000
6	-4.989901000	4.359860000	2.437512000
6	-3.542778000	4.719993000	2.213314000
8	-2.608838000	3.886250000	2.382222000
6	-5.499761000	3.255769000	1.452789000
6	-6.218996000	2.109105000	2.188115000
6	-5.202273000	1.232916000	2.928189000
8	-4.313487000	0.603565000	2.291278000
8	-5.336511000	1.275675000	4.240215000
1	-5.057247000	3.969112000	3.461259000
1	-6.184277000	3.700160000	0.722788000
1	-4.662428000	2.827604000	0.892641000
1	-6.734766000	1.453217000	1.482025000
1	-6.968343000	2.490554000	2.887093000
7	-3.234938000	6.004841000	1.850384000
6	-1.870609000	6.352298000	1.437440000
6	-0.808849000	6.022757000	2.485932000
8	0.328621000	5.605608000	2.127661000
1	-3.982746000	6.647178000	1.629761000
1	-1.579432000	5.818576000	0.526937000
7	-1.111964000	6.243935000	3.790089000
6	-0.155168000	5.958228000	4.863824000
6	0.018814000	4.462508000	5.185337000
8	1.126403000	4.044122000	5.604324000
1	-2.030564000	6.598580000	4.017940000
1	0.831098000	6.335740000	4.588495000
7	-1.075009000	3.682851000	5.012785000
6	-1.072994000	2.213181000	5.190599000
6	-0.099275000	1.595047000	4.164088000
8	0.849315000	0.848926000	4.521526000
6	-2.526572000	1.732020000	5.018811000
6	-2.871679000	0.407046000	5.677656000
8	-2.144614000	-0.096367000	6.586509000
8	-3.985020000	-0.162104000	5.278120000
1	-1.858302000	4.062901000	4.496260000
1	-0.693355000	1.956028000	6.180803000
1	-2.790588000	1.654318000	3.962450000
1	-3.203394000	2.475266000	5.460539000
7	-0.288497000	1.979729000	2.874829000
6	0.595508000	1.519781000	1.798731000
6	2.037394000	2.002616000	2.032138000
8	3.022555000	1.290802000	1.695208000
6	0.069347000	1.985081000	0.418020000
6	-1.313403000	1.428192000	-0.018570000
6	-1.378174000	-0.084198000	-0.089062000
8	-1.076449000	-0.737863000	0.962629000
8	-1.751156000	-0.642706000	-1.213046000
1	-1.101441000	2.537568000	2.629379000
1	0.635827000	0.429795000	1.812504000
1	0.820205000	1.698791000	-0.325813000
1	0.012074000	3.080627000	0.398016000
1	-2.092969000	1.746495000	0.685414000
1	-1.561843000	1.840475000	-0.999971000
7	2.195173000	3.228648000	2.601139000
6	3.500981000	3.765218000	2.970275000
6	4.288666000	2.899305000	3.953849000
8	5.533245000	2.771095000	3.801709000
1	1.386202000	3.819878000	2.745562000
1	4.158869000	3.867628000	2.103565000
7	3.612188000	2.306859000	4.966292000
6	4.301677000	1.375600000	5.860124000
6	4.538241000	-0.008231000	5.232433000
8	5.690755000	-0.535483000	5.229633000
1	2.626578000	2.507679000	5.110961000
1	5.283813000	1.772006000	6.121911000
7	3.454821000	-0.626317000	4.710756000
6	3.576234000	-1.949346000	4.085562000
6	2.419438000	-2.242657000	3.126938000
6	1.103372000	-2.466575000	3.805368000
7	-0.070605000	-2.665595000	3.072676000
6	0.777355000	-2.474151000	5.147947000
6	-1.071927000	-2.771130000	3.959472000
7	-0.592287000	-2.667121000	5.219157000
1	2.557612000	-0.140181000	4.652481000
1	3.640779000	-2.723184000	4.864808000
1	2.672085000	-3.127588000	2.525680000
1	2.331947000	-1.413108000	2.414420000
1	1.370334000	-2.288365000	6.026430000
1	-2.114213000	-2.897819000	3.723211000
6	0.030434000	-1.448582000	-4.751969000
6	1.061617000	-2.016612000	-5.702325000
8	1.250271000	-3.278076000	-5.796199000
6	-1.372087000	-1.319642000	-5.412446000
6	-2.011149000	-2.638991000	-5.869108000
6	-2.638871000	-3.526226000	-4.800301000
8	-2.951431000	-2.917507000	-3.624962000
8	-2.926512000	-4.708637000	-5.032967000
1	-0.026817000	-2.104184000	-3.877958000
1	-2.033341000	-0.831321000	-4.689690000
1	-1.279683000	-0.650963000	-6.275959000
1	-1.308275000	-3.259659000	-6.434785000
1	-2.831061000	-2.404170000	-6.564301000
7	1.750229000	-1.155921000	-6.479624000
6	2.596462000	-1.615986000	-7.590214000
6	3.654772000	-2.666534000	-7.235257000
8	3.892778000	-3.601553000	-8.031539000
1	1.494900000	-0.163988000	-6.442542000
1	1.987649000	-2.074775000	-8.374924000
7	4.320306000	-2.505074000	-6.053770000
6	5.319768000	-3.459771000	-5.595559000
6	4.846800000	-4.329357000	-4.429111000
8	5.677316000	-4.749057000	-3.566220000
1	4.251355000	-1.649867000	-5.511659000
1	5.570881000	-4.113529000	-6.438916000
7	3.538746000	-4.655904000	-4.409156000
6	2.964739000	-5.575456000	-3.416423000
6	1.531756000	-5.986895000	-3.783368000
8	0.659912000	-4.814987000	-3.759179000
6	0.965832000	-7.007133000	-2.799699000
1	2.916983000	-4.223104000	-5.091066000
1	2.941146000	-5.104389000	-2.426134000
1	1.517367000	-6.401370000	-4.800927000
1	0.730886000	-4.268047000	-4.595443000
1	-0.068338000	-7.240822000	-3.065897000
1	1.551093000	-7.933163000	-2.817287000
1	0.974808000	-6.609192000	-1.778617000
6	-5.435669000	-6.315385000	3.506729000
6	-6.593660000	-5.610021000	4.197078000
8	-7.710211000	-5.485120000	3.617444000
6	-5.914468000	-7.629199000	2.854604000
6	-4.842681000	-8.316146000	1.969176000
6	-4.315096000	-7.368201000	0.907909000
8	-3.220375000	-6.753667000	1.060232000
7	-5.096766000	-7.129861000	-0.165971000
1	-5.044861000	-5.639041000	2.735078000
1	-6.808731000	-7.405106000	2.262800000
1	-6.225833000	-8.335208000	3.635152000
1	-5.276938000	-9.210541000	1.504718000
1	-3.990331000	-8.636973000	2.575697000
1	-4.808847000	-6.380447000	-0.803251000
1	-5.968077000	-7.614930000	-0.309201000
7	-6.365119000	-5.128226000	5.446838000
6	-7.443426000	-4.538784000	6.239479000
6	-8.086994000	-3.268746000	5.673074000
8	-9.252311000	-2.963574000	6.018346000
1	-5.404692000	-5.078541000	5.794981000
1	-8.261724000	-5.255120000	6.353917000
7	-7.322766000	-2.523892000	4.829091000
6	-7.842927000	-1.331176000	4.179689000
6	-8.029229000	-1.472563000	2.667719000
8	-8.115970000	-0.436070000	1.954273000
1	-6.340293000	-2.755037000	4.656509000
1	-8.818882000	-1.111102000	4.627014000

7	-8.137004000	-2.736369000	2.183000000
6	-8.405843000	-3.024709000	0.775604000
6	-7.251146000	-3.717092000	0.056735000
8	-6.133285000	-2.800036000	-0.099557000
1	-8.080898000	-3.524622000	2.825981000
1	-8.630415000	-2.078005000	0.279731000
1	-6.933443000	-4.606972000	0.617060000
1	-7.594195000	-4.037170000	-0.935946000
1	-5.501359000	-3.075840000	-0.834007000
6	-8.214640000	4.109559000	-3.420579000
6	-7.692739000	2.830247000	-2.800466000
6	-8.282965000	2.296225000	-1.641171000
6	-6.628657000	2.124972000	-3.391596000
6	-7.834157000	1.087932000	-1.091400000
6	-6.173230000	0.918568000	-2.845056000
6	-6.774708000	0.394979000	-1.693520000
1	-8.961510000	3.899563000	-4.198433000
1	-8.697118000	4.749784000	-2.673725000
1	-9.109813000	2.825842000	-1.173578000
1	-6.167084000	2.515633000	-4.295833000
1	-8.296583000	0.680520000	-0.196808000
1	-5.354710000	0.384936000	-3.320181000
1	-6.436763000	-0.543919000	-1.265733000
26	-0.521385000	-2.807623000	1.023950000
26	-2.192938000	-2.549663000	-1.577043000
8	0.168902000	-4.812221000	0.636717000
1	1.145329000	-4.778850000	0.480141000
1	-0.199378000	-5.457716000	1.367296000
8	-4.509424000	-2.576971000	4.176494000
1	-4.344576000	-1.697916000	4.613227000
1	-4.493514000	-2.465339000	3.162507000
8	-4.415230000	-2.019663000	1.658357000
1	-5.194057000	-2.273882000	1.054413000
1	-4.298992000	-1.036579000	1.737475000
8	-2.351691000	-3.245419000	0.387906000
1	-3.241717000	-4.506026000	-0.353616000
1	-3.083729000	-2.790459000	0.901148000
8	-0.581236000	-3.984588000	8.692534000
1	-0.224101000	-4.614238000	7.979424000
1	-0.536551000	-4.355009000	9.586839000
8	-4.222262000	-3.507671000	-1.744712000
8	-3.850748000	-4.836215000	-1.102532000
8	-0.741304000	-3.970285000	-1.755729000
1	-0.257088000	-4.325580000	-2.566759000
8	-0.957619000	-6.254655000	2.364636000
1	-1.801359000	-6.549816000	1.930200000
1	-1.127468000	-6.002812000	3.305268000
8	-2.299827000	-2.709840000	7.339908000
1	-1.696073000	-3.087775000	8.065784000
1	-2.403098000	-1.721038000	7.299865000
8	-1.769175000	-6.000165000	5.043351000
1	-2.062177000	-6.924341000	5.143455000
1	-2.540221000	-5.328566000	5.304185000
8	0.210035000	-5.414752000	6.694189000
1	-0.525430000	-5.685197000	6.057427000
1	0.942826000	-5.013877000	6.195813000
8	-3.553735000	-4.364704000	5.760019000
1	-3.929305000	-3.734518000	5.048863000
1	-3.186322000	-3.784085000	6.507429000
1	3.700585000	1.266618000	6.767573000
1	3.337999000	4.756559000	3.402921000
1	-0.483554000	6.482098000	5.766521000
1	-1.845500000	7.426022000	1.223276000
1	-5.628835000	5.248701000	2.389298000
1	-4.794075000	6.154179000	-3.907709000
1	-1.866957000	7.426160000	-8.115472000
1	0.420712000	10.644528000	-5.155977000
1	6.844356000	4.753478000	-3.821071000
1	3.660110000	4.187909000	0.183416000
1	7.772248000	-1.286284000	-2.973326000
1	9.622180000	2.281767000	-0.254494000
1	6.158287000	1.025925000	3.082150000
1	8.990311000	-2.499212000	3.474196000
1	3.088001000	-0.732462000	-8.009965000
1	6.215235000	-2.930740000	-5.269176000
1	-7.045353000	-4.317325000	7.234924000
1	-7.186224000	-0.475092000	4.356051000
1	-4.613843000	-6.500781000	4.206393000
1	-9.290310000	-3.671879000	0.708807000
1	8.190310000	-4.561674000	-0.231453000
1	-7.412552000	4.686774000	-3.895042000
1	0.342956000	-0.457496000	-4.408864000
1	3.605188000	-6.463294000	-3.353141000
1	-0.514240000	-4.477955000	-0.927422000
1	4.526570000	-1.974747000	3.541919000

$^{9}Pr_B$:

6	8.623765000	-2.662597000	2.301446000
6	7.855351000	-1.512467000	1.665366000
8	7.769622000	-1.448790000	0.402601000
6	7.775588000	-3.972633000	2.416377000
6	6.828778000	-4.200291000	1.279996000
7	7.194108000	-4.361646000	-0.063888000
6	5.458560000	-4.187808000	1.279451000
6	6.090563000	-4.413704000	-0.849051000
7	5.030797000	-4.317304000	-0.035846000
1	9.494202000	-2.842700000	1.662472000
1	8.463552000	-4.818906000	2.537303000
1	7.174661000	-3.929065000	3.330443000
1	4.765012000	-4.082863000	2.093987000
1	6.051051000	-4.471688000	-1.939614000
1	4.005112000	-4.185291000	-0.308578000
7	7.287854000	-0.600713000	2.491348000
6	6.362617000	0.445703000	2.038004000
6	6.792908000	1.244478000	0.835481000
8	5.921359000	1.712363000	0.046999000
1	7.238829000	-0.761066000	3.498123000
1	5.374990000	0.039016000	1.801886000
7	8.103366000	1.486430000	0.599890000
6	8.502979000	2.229234000	-0.596801000
6	7.889603000	1.685012000	-1.895713000
8	7.604485000	2.477990000	-2.827873000
1	8.800638000	1.131136000	1.236349000
1	8.192962000	3.275394000	-0.532019000
7	7.703757000	0.338939000	-1.989640000
6	7.147672000	-0.254001000	-3.203301000
6	5.633213000	-0.106138000	-3.399331000
8	5.117829000	-0.491473000	-4.481366000
1	7.851192000	-0.259576000	-1.176381000
1	7.616060000	0.199589000	-4.081072000
7	4.922757000	0.413655000	-2.371771000
6	3.474196000	0.665141000	-2.463242000
6	3.216399000	2.170348000	-2.288855000
8	2.684474000	2.862995000	-3.202642000
6	2.669143000	-0.173860000	-1.447703000
6	2.889094000	-1.685939000	-1.594742000
6	2.172422000	-2.484407000	-0.518464000
8	1.222806000	-1.918237000	0.120427000
8	2.541069000	-3.717473000	-0.294434000
1	5.409448000	0.719099000	-1.530916000
1	3.183425000	0.416576000	-3.485414000
1	1.604314000	0.048418000	-1.579341000
1	2.921452000	0.130588000	-0.425223000
1	3.957316000	-1.931572000	-1.571916000
1	2.512646000	-2.047715000	-2.563340000
7	3.623749000	2.705307000	-1.111946000
6	3.453660000	4.113336000	-0.786745000
6	4.282114000	5.082223000	-1.616178000
8	3.951474000	6.305299000	-1.637600000
1	4.086801000	2.134154000	-0.407907000
1	2.410439000	4.425211000	-0.909358000
7	5.303578000	4.603733000	-2.358830000
6	5.973958000	5.448922000	-3.345148000
6	5.018256000	6.042850000	-4.387470000
8	5.346310000	7.078365000	-5.009826000
1	5.616694000	3.644864000	-2.263417000
1	6.464326000	6.302670000	-2.869698000
7	3.826607000	5.399403000	-4.578709000
6	2.797456000	5.945753000	-5.461158000
6	1.524158000	6.313974000	-4.680284000

8	0.405937000	6.372860000	-5.263175000
6	2.480444000	5.007151000	-6.634632000
1	3.616991000	4.519904000	-4.109303000
1	3.218821000	6.881792000	-5.853888000
1	1.666589000	5.415243000	-7.237626000
1	3.371537000	4.891078000	-7.257773000
1	2.174959000	4.020556000	-6.273058000
7	1.650037000	6.641938000	-3.361340000
6	0.455183000	6.842041000	-2.529924000
6	-0.440781000	7.984667000	-3.021173000
8	-1.672837000	7.962513000	-2.762309000
6	0.811509000	7.041401000	-1.042098000
8	0.632688000	5.757187000	-0.366665000
1	2.552616000	6.553418000	-2.888547000
1	-0.183458000	5.953782000	-2.585213000
1	1.844345000	7.387949000	-0.936328000
1	0.131094000	7.783651000	-0.606380000
1	0.838587000	5.820109000	0.598247000
7	0.148255000	8.998731000	-3.703049000
6	-0.630224000	10.103192000	-4.257525000
6	-1.616603000	9.732352000	-5.375653000
8	-2.483501000	10.572212000	-5.708540000
1	1.136889000	8.929757000	-3.901928000
1	-1.234403000	10.567065000	-3.472993000
7	-1.488561000	8.500305000	-5.941250000
6	-2.452858000	8.005161000	-6.915272000
6	-3.247619000	6.787272000	-6.441449000
8	-3.860011000	6.078845000	-7.280618000
1	-0.744017000	7.862000000	-5.660732000
1	-3.147688000	8.821817000	-7.137356000
7	-3.258184000	6.545093000	-5.101990000
6	-3.959952000	5.402468000	-4.524222000
6	-3.016110000	4.328642000	-3.945438000
6	-2.083887000	3.623703000	-4.907719000
6	-2.425405000	3.380334000	-6.267007000
6	-0.845952000	3.121053000	-4.417371000
6	-1.604286000	2.618634000	-7.075392000
6	-0.010523000	2.359477000	-5.206553000
6	-0.382776000	2.042870000	-6.565781000
8	0.337327000	1.255238000	-7.304557000
1	-2.754905000	7.161414000	-4.470598000
1	-4.603857000	4.991387000	-5.304693000
1	-3.649319000	3.561561000	-3.466899000
1	-2.422138000	4.778534000	-3.139039000
1	-3.318957000	3.828756000	-6.686484000
1	-0.551846000	3.345055000	-3.396498000
1	-1.857758000	2.419478000	-8.110660000
1	0.943614000	2.011338000	-4.825803000
1	-3.770671000	-4.724490000	-2.474187000
6	-4.774689000	4.433435000	2.699681000
6	-3.331575000	4.838960000	2.539239000
8	-2.387908000	4.006659000	2.649495000
6	-5.239754000	3.435700000	1.587846000
6	-6.020216000	2.237895000	2.169124000
6	-5.064751000	1.310961000	2.932811000
8	-4.259527000	0.565429000	2.313657000
8	-5.142815000	1.447731000	4.244141000
1	-4.852234000	3.926690000	3.670184000
1	-5.871452000	3.962093000	0.864063000
1	-4.374105000	3.048578000	1.040193000
1	-6.471058000	1.636180000	1.375384000
1	-6.821835000	2.580483000	2.829793000
7	-3.039309000	6.154127000	2.290436000
6	-1.680137000	6.550455000	1.902916000
6	-0.607692000	6.161791000	2.919706000
8	0.532644000	5.788446000	2.525094000
1	-3.795785000	6.799882000	2.113452000
1	-1.385714000	6.086117000	0.955851000
7	-0.905369000	6.283266000	4.238904000
6	0.061045000	5.927666000	5.283417000
6	0.229810000	4.412762000	5.505544000
8	1.345095000	3.955186000	5.856663000
1	-1.825627000	6.612395000	4.496604000
1	1.045889000	6.317478000	5.021031000
7	-0.881013000	3.659764000	5.322202000
6	-0.904604000	2.180484000	5.397154000
6	0.072756000	1.619311000	4.340957000
8	1.004234000	0.832846000	4.650566000
6	-2.365479000	1.744071000	5.165640000
6	-2.771831000	0.400755000	5.747433000
8	-2.086549000	-0.178765000	6.641331000
8	-3.904969000	-0.095904000	5.302203000
1	-1.676678000	4.090289000	4.869533000
1	-0.546825000	1.845482000	6.372400000
1	-2.605102000	1.730677000	4.101373000
1	-3.036251000	2.481474000	5.626200000
7	-0.107683000	2.094585000	3.081882000
6	0.745848000	1.681758000	1.964636000
6	2.192848000	2.154097000	2.165234000
8	3.164711000	1.464300000	1.752052000
6	0.172689000	2.213446000	0.629313000
6	-1.229033000	1.671537000	0.245974000
6	-1.274597000	0.165217000	0.100948000
8	-1.060122000	-0.546733000	1.139985000
8	-1.526634000	-0.327667000	-1.080243000
1	-0.910686000	2.683567000	2.879050000
1	0.785026000	0.591562000	1.930447000
1	0.886298000	1.958225000	-0.160388000
1	0.118731000	3.308755000	0.660999000
1	-1.966608000	1.947404000	1.007704000
1	-1.531302000	2.126176000	-0.700800000
7	2.370046000	3.352028000	2.785331000
6	3.690489000	3.867680000	3.128372000
6	4.501060000	2.941559000	4.035927000
8	5.737658000	2.805172000	3.837005000
1	1.567708000	3.933123000	2.992934000
1	4.314688000	4.012843000	2.242648000
7	3.844365000	2.302943000	5.034223000
6	4.542248000	1.315628000	5.858579000
6	4.708934000	-0.051128000	5.172827000
8	5.854704000	-0.581331000	5.052068000
1	2.866115000	2.509072000	5.217698000
1	5.546810000	1.673930000	6.087752000
7	3.579306000	-0.655015000	4.740368000
6	3.637236000	-1.966803000	4.081502000
6	2.442654000	-2.210727000	3.155094000
6	1.147921000	-2.475738000	3.861413000
7	-0.048293000	-2.635629000	3.151428000
6	0.860641000	-2.584922000	5.206668000
6	-1.020579000	-2.822422000	4.059078000
7	-0.502411000	-2.805464000	5.306141000
1	2.683384000	-0.163161000	4.764614000
1	3.708228000	-2.760689000	4.839378000
1	2.675587000	-3.064443000	2.502588000
1	2.332884000	-1.349204000	2.485706000
1	1.486341000	-2.484819000	6.075830000
1	-2.069592000	-2.940331000	3.848873000
6	-0.232525000	-1.527489000	-5.424316000
6	0.883922000	-2.139091000	-6.238486000
8	1.175106000	-3.382960000	-6.147257000
6	-1.639907000	-1.783784000	-6.048339000
6	-2.247287000	-3.165035000	-5.749615000
6	-2.735961000	-3.352198000	-4.316779000
8	-2.562844000	-2.338075000	-3.495395000
8	-3.272734000	-4.446064000	-3.975688000
1	-0.206294000	-1.941846000	-4.413455000
1	-2.312081000	-1.013612000	-5.658686000
1	-1.578998000	-1.637959000	-7.134268000
1	-1.530640000	-3.969463000	-5.960270000
1	-3.104651000	-3.350254000	-6.409630000
7	1.543380000	-1.332894000	-7.095265000
6	2.460587000	-1.851126000	-8.117399000
6	3.625219000	-2.712543000	-7.621985000
8	4.017031000	-3.675431000	-8.319103000
1	1.250828000	-0.350592000	-7.156893000
1	1.922825000	-2.482173000	-8.831149000
7	4.214525000	-2.363848000	-6.440570000
6	5.309222000	-3.140294000	-5.876267000
6	4.905996000	-3.943361000	-4.641331000
8	5.760112000	-4.199467000	-3.737780000

1	3.981775000	-1.506652000	-5.952288000
1	5.655027000	-3.836024000	-6.649326000
7	3.635512000	-4.394062000	-4.604187000
6	3.133641000	-5.269227000	-3.538100000
6	1.714802000	-5.770519000	-3.837581000
8	0.791756000	-4.638507000	-3.887556000
6	1.218086000	-6.736279000	-2.765451000
1	2.987806000	-4.084375000	-5.327098000
1	3.109402000	-4.732943000	-2.582026000
1	1.700122000	-6.262420000	-4.820269000
1	0.799252000	-4.185236000	-4.784017000
1	0.188930000	-7.033751000	-2.982888000
1	1.843001000	-7.635585000	-2.730253000
1	1.235594000	-6.261771000	-1.778129000
6	-5.835782000	-6.262839000	3.779141000
6	-6.941937000	-5.369365000	4.319765000
8	-8.030205000	-5.215561000	3.695500000
6	-6.397934000	-7.456956000	2.988275000
6	-5.295031000	-8.446628000	2.528879000
6	-4.150076000	-7.725968000	1.843903000
8	-3.072133000	-7.495083000	2.485953000
7	-4.346203000	-7.286221000	0.592464000
1	-5.197635000	-5.651586000	3.125276000
1	-6.960528000	-7.079533000	2.127495000
1	-7.119941000	-8.004389000	3.605509000
1	-5.732092000	-9.196881000	1.858021000
1	-4.876372000	-8.972038000	3.391610000
1	-3.694533000	-6.627966000	0.130840000
1	-5.206368000	-7.489673000	0.105654000
7	-6.677710000	-4.751068000	5.500808000
6	-7.699881000	-3.974188000	6.197480000
6	-8.234261000	-2.747525000	5.454934000
8	-9.383377000	-2.317652000	5.710441000
1	-5.708208000	-4.737641000	5.836334000
1	-8.574811000	-4.597207000	6.403285000
7	-7.392246000	-2.171738000	4.553790000
6	-7.806125000	-1.038207000	3.746233000
6	-7.851083000	-1.332822000	2.247819000
8	-7.748878000	-0.385432000	1.420926000
1	-6.420133000	-2.475078000	4.452115000
1	-8.811845000	-0.752342000	4.076462000
7	-8.054999000	-2.627387000	1.890797000
6	-8.242135000	-3.046303000	0.502342000
6	-7.169475000	-4.005240000	-0.005097000
8	-5.905455000	-3.307394000	-0.178625000
1	-8.142077000	-3.336648000	2.617742000
1	-8.254569000	-2.147370000	-0.117619000
1	-7.048074000	-4.838695000	0.701123000
1	-7.490633000	-4.412851000	-0.972348000
1	-5.254425000	-3.842304000	-0.720740000
6	-7.361808000	3.181216000	-4.606122000
6	-6.884548000	2.054973000	-3.712711000
6	-7.556140000	1.760178000	-2.511392000
6	-5.783310000	1.259235000	-4.072805000
6	-7.151938000	0.694099000	-1.700500000
6	-5.372420000	0.187730000	-3.266923000
6	-6.059610000	-0.099316000	-2.081962000
1	-8.328153000	2.940156000	-5.068886000
1	-7.501351000	4.114056000	-4.045468000
1	-8.410275000	2.366184000	-2.217027000
1	-5.253877000	1.470682000	-4.999028000
1	-7.675152000	0.469604000	-0.775593000
1	-4.524959000	-0.423895000	-3.563743000
1	-5.765954000	-0.941815000	-1.463836000
26	-0.496718000	-2.610353000	1.118478000
26	-1.895019000	-2.190815000	-1.678903000
8	0.159082000	-4.601880000	0.604158000
1	1.136554000	-4.553271000	0.442220000
1	-0.180309000	-5.278488000	1.314291000
8	-4.559386000	-2.376260000	3.971057000
1	-4.329412000	-1.513763000	4.406015000
1	-4.485005000	-2.304048000	2.964743000
8	-4.392405000	-1.947068000	1.391601000
1	-5.133007000	-2.354389000	0.832724000
1	-4.402795000	-0.958316000	1.466844000
8	-2.259956000	-2.971028000	0.190906000
1	-2.671304000	-4.373994000	-0.256870000
1	-3.036250000	-2.548608000	0.683179000
8	-1.418111000	-4.602530000	8.789119000
1	-1.169164000	-5.317452000	8.102369000
1	-1.796797000	-4.987876000	9.594538000
8	-4.155345000	-4.824898000	-1.527710000
8	-2.970474000	-5.270252000	-0.699089000
8	-0.481307000	-3.679752000	-1.836377000
1	0.003300000	-4.047627000	-2.639835000
8	-0.808245000	-6.093913000	2.369420000
1	-1.559371000	-6.696535000	2.131835000
1	-1.004979000	-5.769511000	3.279192000
8	-2.341624000	-2.843288000	7.246586000
1	-2.011158000	-3.385981000	8.046249000
1	-2.400091000	-1.854920000	7.300782000
8	-2.223411000	-6.061660000	4.779013000
1	-2.632685000	-6.766429000	4.226677000
1	-2.906707000	-5.343237000	5.056922000
8	-0.850493000	-6.309986000	6.959570000
1	-1.387252000	-6.304829000	6.094200000
1	0.067098000	-6.582873000	6.801960000
8	-3.846656000	-4.242459000	5.613668000
1	-4.135866000	-3.572860000	4.906534000
1	-3.366641000	-3.733771000	6.346076000
1	3.979786000	1.190163000	6.788420000
1	3.549768000	4.835801000	3.617592000
1	-0.255879000	6.394897000	6.220703000
1	-1.671546000	7.637228000	1.767682000
1	-5.431965000	5.309315000	2.734611000
1	-4.610507000	5.753753000	-3.714411000
1	-1.960417000	7.717661000	-7.848909000
1	0.062123000	10.858379000	-4.639743000
1	6.737774000	4.839044000	-3.834447000
1	3.710051000	4.246200000	0.266414000
1	7.373801000	-1.324822000	-3.210191000
1	9.594286000	2.197261000	-0.671902000
1	6.230114000	1.153375000	2.866162000
1	8.993021000	-2.399105000	3.297707000
1	2.859528000	-0.988292000	-8.660502000
1	6.125750000	-2.479801000	-5.588694000
1	-7.280937000	-3.651788000	7.156312000
1	-7.142481000	-0.185105000	3.904656000
1	-5.199200000	-6.606860000	4.601068000
1	-9.216197000	-3.544777000	0.409038000
1	8.135416000	-4.313109000	-0.420927000
1	-6.653688000	3.378884000	-5.417646000
1	-0.076279000	-0.450109000	-5.340158000
1	3.814920000	-6.122550000	-3.430659000
1	-0.387034000	-4.252504000	-1.028603000
1	4.567212000	-2.000538000	3.504566000

$^{7}Re_{B}$:

6	8.740826000	-2.974798000	3.195099000
6	8.030447000	-1.850032000	2.455519000
8	8.040200000	-1.829779000	1.189590000
6	7.826241000	-4.222090000	3.417833000
6	6.952999000	-4.571153000	2.253072000
7	7.405642000	-4.933774000	0.976335000
6	5.586920000	-4.535166000	2.150209000
6	6.357542000	-5.089657000	0.130459000
7	5.246337000	-4.852770000	0.840490000
1	9.606865000	-3.251017000	2.585837000
1	8.463679000	-5.069216000	3.701954000
1	7.165148000	-4.032271000	4.269788000
1	4.842946000	-4.299363000	2.888774000
1	6.397885000	-5.327227000	-0.936535000
1	4.246771000	-4.747327000	0.472761000
7	7.391923000	-0.915037000	3.201988000
6	6.480616000	0.086913000	2.637487000
6	6.989110000	0.859043000	1.449861000
8	6.185335000	1.231606000	0.548232000
1	7.284573000	-1.024497000	4.209748000
1	5.528521000	-0.356069000	2.332084000
7	8.299196000	1.182742000	1.344992000

6	8.766795000	1.933034000	0.176714000
6	8.350247000	1.302066000	-1.159238000
8	8.112223000	2.031973000	-2.152553000
1	8.941677000	0.922573000	2.078215000
1	8.361770000	2.948183000	0.173552000
7	8.291730000	-0.059324000	-1.211177000
6	7.926882000	-0.739344000	-2.447788000
6	6.434504000	-0.742231000	-2.800431000
8	6.079049000	-1.195387000	-3.921297000
1	8.375303000	-0.614767000	-0.360015000
1	8.436778000	-0.265229000	-3.290979000
7	5.577337000	-0.261113000	-1.873409000
6	4.139658000	-0.081001000	-2.136374000
6	3.802613000	1.419792000	-2.178135000
8	3.256913000	1.935590000	-3.192861000
6	3.249190000	-0.812518000	-1.111479000
6	3.444717000	-2.334599000	-1.099168000
6	2.525620000	-3.025386000	-0.107215000
8	1.503760000	-2.381550000	0.312152000
8	2.789199000	-4.248592000	0.269451000
1	5.947998000	0.103848000	-0.997840000
1	3.961052000	-0.463988000	-3.141987000
1	2.203393000	-0.592806000	-1.352733000
1	3.418181000	-0.406939000	-0.107006000
1	4.485629000	-2.597945000	-0.876080000
1	3.214177000	-2.774114000	-2.080795000
7	4.134874000	2.129703000	-1.071139000
6	3.826748000	3.542678000	-0.900337000
6	4.543111000	4.497283000	-1.842128000
8	4.101261000	5.677543000	-1.960614000
1	4.594652000	1.673058000	-0.285681000
1	2.756715000	3.740080000	-1.041040000
7	5.568793000	4.044322000	-2.597303000
6	6.052994000	4.844536000	-3.721619000
6	4.953083000	5.197801000	-4.737459000
8	5.114093000	6.178225000	-5.499787000
1	6.013363000	3.154356000	-2.401174000
1	6.466081000	5.798715000	-3.384488000
7	3.838289000	4.407126000	-4.752835000
6	2.695819000	4.706787000	-5.613841000
6	1.510864000	5.330054000	-4.851634000
8	0.400855000	5.472727000	-5.433483000
6	2.228039000	3.457169000	-6.379898000
1	3.767074000	3.574894000	-4.165807000
1	3.039902000	5.467939000	-6.325160000
1	1.330559000	3.689573000	-6.956675000
1	3.020497000	3.122583000	-7.056339000
1	1.992461000	2.647131000	-5.680855000
7	1.709724000	5.764287000	-3.575727000
6	0.581651000	6.213419000	-2.752713000
6	-0.093702000	7.486789000	-3.282444000
8	-1.261028000	7.773323000	-2.904739000
6	0.991148000	6.423446000	-1.287293000
8	0.896394000	5.143391000	-0.589982000
1	2.621163000	5.668938000	-3.125868000
1	-0.207720000	5.452920000	-2.774872000
1	2.011867000	6.815207000	-1.226816000
1	0.294554000	7.143767000	-0.841679000
1	1.056476000	5.258198000	0.377751000
7	0.613441000	8.256848000	-4.144616000
6	0.021154000	9.416239000	-4.802774000
6	-1.085243000	9.104926000	-5.822516000
8	-1.763094000	10.056644000	-6.273158000
1	1.540703000	7.951399000	-4.406300000
1	-0.434741000	10.075070000	-4.058474000
7	-1.272414000	7.800951000	-6.168771000
6	-2.383579000	7.399091000	-7.021782000
6	-3.584099000	6.794871000	-6.285195000
8	-4.524592000	6.299541000	-6.956869000
1	-0.653884000	7.064231000	-5.829550000
1	-2.734852000	8.281392000	-7.564688000
7	-3.565714000	6.839504000	-4.927306000
6	-4.665796000	6.327873000	-4.109262000
6	-4.250496000	5.206009000	-3.141150000
6	-3.848252000	3.865829000	-3.744951000

6	-4.104160000	3.504356000	-5.080232000
6	-3.242859000	2.905801000	-2.914329000
6	-3.771583000	2.232336000	-5.557191000
6	-2.909420000	1.629339000	-3.375179000
6	-3.177027000	1.287307000	-4.711058000
8	-2.871175000	0.047502000	-5.237979000
1	-2.774551000	7.253878000	-4.445398000
1	-5.445244000	5.994332000	-4.798411000
1	-5.103003000	5.044971000	-2.460326000
1	-3.427533000	5.583431000	-2.514714000
1	-4.548548000	4.214931000	-5.769856000
1	-3.039948000	3.158119000	-1.874663000
1	-3.966592000	1.957277000	-6.587661000
1	-2.454047000	0.899018000	-2.713157000
1	-2.477129000	-0.602583000	-4.586203000
6	-4.752267000	4.378872000	2.419357000
6	-3.282050000	4.662860000	2.249451000
8	-2.398576000	3.791086000	2.490918000
6	-5.279607000	3.309920000	1.404841000
6	-6.057198000	2.176473000	2.100561000
6	-5.097523000	1.237169000	2.836215000
8	-4.250127000	0.554484000	2.198828000
8	-5.223554000	1.287085000	4.149424000
1	-4.878015000	3.986441000	3.436485000
1	-5.935955000	3.791602000	0.673014000
1	-4.448047000	2.865559000	0.848124000
1	-6.584990000	1.565326000	1.364850000
1	-6.807504000	2.568482000	2.791381000
7	-2.897285000	5.915366000	1.853013000
6	-1.503988000	6.179561000	1.478143000
6	-0.496615000	5.858108000	2.579774000
8	0.646524000	5.410699000	2.278734000
1	-3.603672000	6.579109000	1.567722000
1	-1.205770000	5.584206000	0.608980000
7	-0.848156000	6.107951000	3.865966000
6	0.054157000	5.806695000	4.981704000
6	0.171730000	4.308875000	5.320721000
8	1.245678000	3.863620000	5.796393000
1	-1.766931000	6.486899000	4.049665000
1	1.061220000	6.154374000	4.745474000
7	-0.934001000	3.558599000	5.102616000
6	-0.978087000	2.090403000	5.286999000
6	0.014107000	1.442571000	4.296529000
8	0.925961000	0.670445000	4.692714000
6	-2.441396000	1.654955000	5.073968000
6	-2.840608000	0.308924000	5.655497000
8	-2.180518000	-0.237096000	6.591603000
8	-3.932669000	-0.228416000	5.164932000
1	-1.677849000	3.951400000	4.537862000
1	-0.635285000	1.829449000	6.289508000
1	-2.692471000	1.650443000	4.012518000
1	-3.099142000	2.395982000	5.547960000
7	-0.114717000	1.824928000	2.999868000
6	0.793369000	1.331112000	1.958044000
6	2.236379000	1.789239000	2.222876000
8	3.214661000	1.037106000	1.964835000
6	0.320858000	1.785454000	0.556035000
6	-1.072711000	1.274812000	0.104659000
6	-1.164189000	-0.233991000	0.006972000
8	-0.934224000	-0.903460000	1.080377000
8	-1.459582000	-0.778370000	-1.126722000
1	-0.898180000	2.409444000	2.722639000
1	0.811733000	0.241435000	1.993035000
1	1.074196000	1.449247000	-0.165292000
1	0.310087000	2.880663000	0.502048000
1	-1.850200000	1.602791000	0.805994000
1	-1.294234000	1.710481000	-0.871615000
7	2.402223000	3.041632000	2.730760000
6	3.712217000	3.569304000	3.093079000
6	4.448848000	2.750195000	4.153446000
8	5.694983000	2.588282000	4.051987000
1	1.607974000	3.666292000	2.810106000
1	4.390838000	3.593853000	2.235895000
7	3.726964000	2.227532000	5.173718000
6	4.364919000	1.327631000	6.135808000

6	4.591592000	-0.090756000	5.584720000
8	5.734094000	-0.637704000	5.642627000
1	2.747705000	2.475873000	5.292771000
1	5.346905000	1.714200000	6.412988000
7	3.513853000	-0.714834000	5.058626000
6	3.637768000	-2.057219000	4.477950000
6	2.535040000	-2.353508000	3.457024000
6	1.184099000	-2.584456000	4.059856000
7	0.050948000	-2.782498000	3.264357000
6	0.786289000	-2.607166000	5.382212000
6	-0.996198000	-2.900602000	4.095657000
7	-0.584926000	-2.806946000	5.378526000
1	2.625636000	-0.219471000	4.949700000
1	3.639516000	-2.811966000	5.278677000
1	2.820718000	-3.238382000	2.871323000
1	2.486395000	-1.524456000	2.740563000
1	1.331322000	-2.423543000	6.291562000
1	-2.024505000	-3.021088000	3.802971000
6	1.573206000	-1.151207000	-5.055221000
6	2.450926000	-2.195737000	-5.706083000
8	2.341745000	-3.433244000	-5.447888000
6	0.285508000	-0.850270000	-5.875182000
6	-0.647650000	-2.060309000	-6.094751000
6	-1.094201000	-2.730657000	-4.800315000
8	-1.624870000	-1.919546000	-3.877676000
8	-0.948227000	-3.962793000	-4.618378000
1	1.284415000	-1.519969000	-4.065917000
1	-0.269768000	-0.070989000	-5.346754000
1	0.564155000	-0.435464000	-6.853230000
1	-0.175929000	-2.828012000	-6.714376000
1	-1.540495000	-1.694469000	-6.616436000
7	3.354543000	-1.771151000	-6.632489000
6	4.071077000	-2.697253000	-7.515462000
6	5.018348000	-3.689745000	-6.835511000
8	5.296330000	-4.762830000	-7.417278000
1	3.437921000	-0.779233000	-6.803025000
1	3.360775000	-3.310870000	-8.077280000
7	5.522849000	-3.339118000	-5.620702000
6	6.368632000	-4.239658000	-4.855955000
6	5.641364000	-4.965726000	-3.723494000
8	6.305130000	-5.423455000	-2.743245000
1	5.377549000	-2.416662000	-5.219128000
1	6.769677000	-4.997213000	-5.539108000
7	4.312710000	-5.133678000	-3.855077000
6	3.519243000	-5.937306000	-2.916593000
6	2.035438000	-5.927745000	-3.295346000
8	1.523729000	-4.560313000	-3.147004000
6	1.212497000	-6.854363000	-2.405154000
1	3.826606000	-4.662685000	-4.616099000
1	3.613511000	-5.537629000	-1.901110000
1	1.916657000	-6.223585000	-4.345957000
1	1.522610000	-4.098439000	-4.027424000
1	0.159825000	-6.807338000	-2.696972000
1	1.554816000	-7.890879000	-2.500365000
1	1.295513000	-6.555113000	-1.354790000
6	-5.461359000	-6.071357000	2.996822000
6	-6.636284000	-5.523709000	3.800362000
8	-7.762115000	-5.339153000	3.256317000
6	-5.941720000	-7.075998000	1.931979000
6	-4.853807000	-7.513512000	0.912140000
6	-4.219365000	-6.329203000	0.215495000
8	-3.194779000	-5.771768000	0.742631000
7	-4.781214000	-5.840829000	-0.895473000
1	-4.968807000	-5.217395000	2.512801000
1	-6.794448000	-6.633048000	1.407412000
1	-6.322106000	-7.979255000	2.425041000
1	-5.304861000	-8.193970000	0.180417000
1	-4.053552000	-8.058019000	1.423375000
1	-4.408617000	-4.983708000	-1.345161000
1	-5.578959000	-6.295021000	-1.314207000
7	-6.402854000	-5.220367000	5.103801000
6	-7.474781000	-4.724819000	5.966561000
6	-8.059847000	-3.356857000	5.598060000
8	-9.200575000	-3.045901000	6.011633000
1	-5.444359000	-5.202991000	5.461158000
1	-8.318929000	-5.419374000	5.953613000
7	-7.277780000	-2.543216000	4.836709000
6	-7.758749000	-1.261779000	4.343007000
6	-8.027516000	-1.238080000	2.837434000
8	-8.094899000	-0.131626000	2.238311000
1	-6.315386000	-2.794835000	4.603346000
1	-8.695611000	-1.035501000	4.863888000
7	-8.217543000	-2.438271000	2.230056000
6	-8.543964000	-2.560528000	0.811854000
6	-7.492967000	-3.334069000	0.019292000
8	-6.234797000	-2.605020000	-0.048475000
1	-8.171165000	-3.293207000	2.780476000
1	-8.652451000	-1.552756000	0.406977000
1	-7.316144000	-4.308803000	0.490727000
1	-7.864785000	-3.499455000	-0.999802000
1	-5.823246000	-2.574127000	-0.961816000
6	-9.314731000	4.178896000	-3.278312000
6	-8.619459000	3.034300000	-2.571622000
6	-9.117545000	2.525421000	-1.359131000
6	-7.472719000	2.437653000	-3.126099000
6	-8.497714000	1.443488000	-0.721172000
6	-6.848419000	1.355177000	-2.495031000
6	-7.360665000	0.853280000	-1.291676000
1	-10.052190000	3.809985000	-4.004714000
1	-9.850788000	4.821848000	-2.571014000
1	-9.998995000	2.980168000	-0.912776000
1	-7.060802000	2.821639000	-4.055089000
1	-8.878862000	1.064975000	0.222431000
1	-5.961165000	0.914535000	-2.938071000
1	-6.881599000	0.011704000	-0.799754000
26	-0.316607000	-2.866162000	1.200944000
26	-1.921794000	-2.573680000	-1.977627000
8	0.212209000	-4.923412000	0.868189000
1	1.201544000	-4.946068000	0.773453000
1	-0.220056000	-5.562959000	1.558104000
8	-4.455903000	-2.606969000	4.052718000
1	-4.287589000	-1.735290000	4.510725000
1	-4.410606000	-2.476333000	3.050560000
8	-4.281672000	-2.004553000	1.507289000
1	-5.120923000	-2.227735000	0.970672000
1	-4.188422000	-1.019927000	1.653195000
8	-2.248990000	-3.298176000	0.583891000
1	-2.575262000	-4.241527000	0.515368000
1	-3.012256000	-2.695454000	0.918561000
8	-0.943215000	-4.191637000	8.803989000
1	-0.551211000	-4.821264000	8.112269000
1	-0.941154000	-4.554083000	9.702331000
8	-4.659597000	-2.410076000	-2.163240000
8	-3.762387000	-3.461243000	-1.988436000
8	-0.515045000	-4.004175000	-1.587563000
1	0.185638000	-4.232514000	-2.275234000
8	-1.186066000	-6.306938000	2.440589000
1	-2.028016000	-6.282850000	1.920832000
1	-1.350506000	-6.203346000	3.414614000
8	-2.482019000	-2.839781000	7.327524000
1	-1.965211000	-3.238478000	8.106944000
1	-2.553005000	-1.847174000	7.277091000
8	-1.949641000	-6.198306000	5.077286000
1	-2.282677000	-7.097855000	5.251661000
1	-2.689781000	-5.466539000	5.289118000
8	-0.041623000	-5.603996000	6.833154000
1	-0.732145000	-5.871376000	6.150840000
1	0.721391000	-5.195057000	6.389661000
8	-3.646512000	-4.446017000	5.650921000
1	-3.957170000	-3.794614000	4.927958000
1	-3.317706000	-3.881097000	6.432353000
1	3.730375000	1.277184000	7.025450000
1	3.567813000	4.593420000	3.450199000
1	-0.297327000	6.346972000	5.865805000
1	-1.420668000	7.237535000	1.208437000
1	-5.341161000	5.300246000	2.352421000
1	-5.081940000	7.156389000	-3.519794000
1	-2.047852000	6.657678000	-7.751983000
1	0.813356000	9.975503000	-5.307399000
1	6.848160000	4.282274000	-4.218575000

1	4.070192000	3.815679000	0.129420000
1	8.262201000	-1.778967000	-2.389816000
1	9.858333000	1.995497000	0.222456000
1	6.263964000	0.820379000	3.422711000
1	9.113031000	-2.641283000	4.169569000
1	4.642979000	-2.101247000	-8.233339000
1	7.199235000	-3.685702000	-4.415819000
1	-7.089045000	-4.678753000	6.989725000
1	-7.042701000	-0.465153000	4.562181000
1	-4.713685000	-6.522747000	3.659673000
1	-9.505370000	-3.081667000	0.704229000
1	8.370776000	-4.983355000	0.689385000
1	-8.603217000	4.801188000	-3.832269000
1	2.128308000	-0.215892000	-4.907297000
1	3.902623000	-6.965670000	-2.911923000
1	-0.390962000	-4.532469000	-0.758010000
1	4.617670000	-2.116068000	3.992946000

^{7}TS1$_{B}$:

6	8.805352000	-1.320639000	3.242079000
6	8.101638000	-0.172791000	2.532822000
8	8.121039000	-0.116802000	1.267487000
6	7.900435000	-2.585488000	3.395644000
6	7.031819000	-2.885352000	2.213691000
7	7.487077000	-3.163767000	0.916902000
6	5.664441000	-2.879952000	2.118353000
6	6.438091000	-3.299234000	0.067923000
7	5.325271000	-3.133329000	0.794817000
1	9.686493000	-1.564220000	2.640333000
1	8.545028000	-3.440070000	3.638508000
1	7.235176000	-2.446443000	4.254073000
1	4.917340000	-2.705851000	2.871384000
1	6.478858000	-3.471621000	-1.010393000
1	4.316140000	-3.026392000	0.440722000
7	7.463583000	0.744028000	3.300621000
6	6.566743000	1.772548000	2.760300000
6	7.083292000	2.556300000	1.583903000
8	6.286933000	2.935698000	0.678928000
1	7.344658000	0.604926000	4.303150000
1	5.605287000	1.352297000	2.452594000
7	8.393136000	2.884961000	1.490063000
6	8.862183000	3.649810000	0.331546000
6	8.440189000	3.038540000	-1.011907000
8	8.190372000	3.783150000	-1.992127000
1	9.032906000	2.622814000	2.224956000
1	8.461247000	4.666566000	0.342921000
7	8.391836000	1.678822000	-1.088126000
6	8.026164000	1.021115000	-2.336756000
6	6.539015000	1.060778000	-2.706255000
8	6.194295000	0.695023000	-3.861681000
1	8.476539000	1.108937000	-0.246490000
1	8.557560000	1.487586000	-3.170436000
7	5.671478000	1.469593000	-1.753815000
6	4.237652000	1.668660000	-2.021903000
6	3.913423000	3.172170000	-2.055656000
8	3.343996000	3.688667000	-3.058511000
6	3.338860000	0.929020000	-1.010283000
6	3.520057000	-0.594831000	-1.037523000
6	2.601267000	-1.308375000	-0.059589000
8	1.580118000	-0.675990000	0.372722000
8	2.870590000	-2.539518000	0.286329000
1	6.033857000	1.784420000	-0.855711000
1	4.060260000	1.298276000	-3.032631000
1	2.294907000	1.165390000	-1.243233000
1	3.516395000	1.310933000	0.002091000
1	4.560716000	-0.871010000	-0.830596000
1	3.279608000	-1.004265000	-2.029937000
7	4.281416000	3.883243000	-0.962570000
6	4.004962000	5.304037000	-0.799895000
6	4.716427000	6.230217000	-1.774239000
8	4.275190000	7.407258000	-1.925288000
1	4.748313000	3.423571000	-0.182727000
1	2.936248000	5.521241000	-0.918781000
7	5.738168000	5.754964000	-2.521013000
6	6.225397000	6.527772000	-3.663084000
6	5.129752000	6.859877000	-4.689998000
8	5.298560000	7.816260000	-5.480659000
1	6.195378000	4.877410000	-2.295236000
1	6.640866000	7.488705000	-3.348326000
7	4.008583000	6.077909000	-4.684131000
6	2.867711000	6.363362000	-5.551191000
6	1.677429000	6.983147000	-4.793987000
8	0.555999000	7.083141000	-5.363617000
6	2.412255000	5.107226000	-6.313367000
1	3.925949000	5.267558000	-4.068867000
1	3.210685000	7.122611000	-6.265534000
1	1.513601000	5.328900000	-6.892616000
1	3.209413000	4.777677000	-6.986961000
1	2.182910000	4.299590000	-5.609071000
7	1.886399000	7.466021000	-3.537624000
6	0.762529000	7.927322000	-2.715809000
6	0.074599000	9.184068000	-3.268517000
8	-1.084850000	9.480485000	-2.873807000
6	1.179909000	8.165722000	-1.257025000
8	1.081600000	6.898826000	-0.536794000
1	2.802528000	7.390457000	-3.092556000
1	-0.022898000	7.162399000	-2.717341000
1	2.202790000	8.552974000	-1.208032000
1	0.488700000	8.897699000	-0.822473000
1	1.233027000	7.027840000	0.431141000
7	0.758150000	9.923667000	-4.174831000
6	0.145193000	11.053132000	-4.865450000
6	-0.982950000	10.694201000	-5.844747000
8	-1.678617000	11.622014000	-6.316805000
1	1.682715000	9.614760000	-4.441647000
1	-0.296593000	11.740456000	-4.138649000
7	-1.170310000	9.375840000	-6.133547000
6	-2.300740000	8.936526000	-6.941727000
6	-3.505875000	8.417154000	-6.149400000
8	-4.478314000	7.921919000	-6.773808000
1	-0.537831000	8.657010000	-5.782569000
1	-2.642846000	9.782159000	-7.545263000
7	-3.455131000	8.533714000	-4.796519000
6	-4.560187000	8.124038000	-3.930315000
6	-4.187464000	7.018894000	-2.926034000
6	-3.882077000	5.634189000	-3.483925000
6	-4.230234000	5.226284000	-4.784804000
6	-3.278035000	4.683467000	-2.641763000
6	-3.993760000	3.916833000	-5.215783000
6	-3.040720000	3.370085000	-3.056569000
6	-3.404420000	2.981830000	-4.355762000
8	-3.202488000	1.699318000	-4.834654000
1	-2.638928000	8.944028000	-4.354459000
1	-5.377980000	7.810369000	-4.583545000
1	-5.031656000	6.935951000	-2.221316000
1	-3.327926000	7.369431000	-2.334340000
1	-4.668342000	5.929683000	-5.485627000
1	-2.993775000	4.973461000	-1.631661000
1	-4.258795000	3.606304000	-6.220103000
1	-2.577236000	2.650285000	-2.389186000
1	-2.752912000	1.079904000	-4.196147000
6	-4.628595000	6.243121000	2.314831000
6	-3.146602000	6.493402000	2.190408000
8	-2.287065000	5.608135000	2.464100000
6	-5.150045000	5.162860000	1.308028000
6	-5.909202000	4.023132000	2.011579000
6	-4.937948000	3.089158000	2.739579000
8	-4.039843000	2.471759000	2.103659000
8	-5.129772000	3.057726000	4.044548000
1	-4.800095000	5.882066000	3.336996000
1	-5.816902000	5.634432000	0.579283000
1	-4.317883000	4.724946000	0.747585000
1	-6.442403000	3.402127000	1.285940000
1	-6.651575000	4.415268000	2.712537000
7	-2.729113000	7.737823000	1.798370000
6	-1.323294000	7.976209000	1.456728000
6	-0.346737000	7.651759000	2.584628000
8	0.798471000	7.189334000	2.314176000
1	-3.416307000	8.409862000	1.486895000
1	-1.013880000	7.367683000	0.600891000

7	-0.725778000	7.915584000	3.859708000
6	0.148066000	7.615267000	4.998262000
6	0.252035000	6.117729000	5.342537000
8	1.316894000	5.664871000	5.830257000
1	-1.644998000	8.304539000	4.018466000
1	1.161836000	7.959032000	4.786178000
7	-0.857352000	5.374975000	5.112060000
6	-0.913275000	3.908421000	5.302474000
6	0.104254000	3.249809000	4.346377000
8	0.997774000	2.471131000	4.770636000
6	-2.370052000	3.474976000	5.043762000
6	-2.781191000	2.129650000	5.617826000
8	-2.165933000	1.609412000	6.597941000
8	-3.832939000	1.565498000	5.073504000
1	-1.591999000	5.771648000	4.538253000
1	-0.604292000	3.650047000	6.316639000
1	-2.587775000	3.466767000	3.975081000
1	-3.041475000	4.216655000	5.497312000
7	0.015884000	3.628829000	3.045096000
6	0.943489000	3.115718000	2.030888000
6	2.384559000	3.557612000	2.321237000
8	3.358032000	2.795751000	2.074317000
6	0.507442000	3.561118000	0.615828000
6	-0.872053000	3.040595000	0.139811000
6	-0.958722000	1.530133000	0.069082000
8	-0.735118000	0.864194000	1.134381000
8	-1.258189000	0.986892000	-1.079604000
1	-0.755233000	4.218315000	2.744748000
1	0.946975000	2.025915000	2.076004000
1	1.278715000	3.222897000	-0.084919000
1	0.493839000	4.655799000	0.554208000
1	-1.669260000	3.377402000	0.814767000
1	-1.071615000	3.456943000	-0.849566000
7	2.556578000	4.804912000	2.838960000
6	3.866999000	5.307190000	3.233442000
6	4.571758000	4.449105000	4.285011000
8	5.818573000	4.278876000	4.210406000
1	1.768023000	5.437094000	2.912466000
1	4.559330000	5.344388000	2.387570000
7	3.816823000	3.903935000	5.269002000
6	4.412993000	2.967776000	6.222951000
6	4.617173000	1.554051000	5.650576000
8	5.750314000	0.987556000	5.707470000
1	2.833071000	4.144807000	5.354465000
1	5.398064000	3.325907000	6.525701000
7	3.531522000	0.954334000	5.112388000
6	3.633046000	-0.388644000	4.526374000
6	2.537667000	-0.662504000	3.491051000
6	1.172456000	-0.859578000	4.075087000
7	0.047955000	-1.072332000	3.269231000
6	0.756078000	-0.834228000	5.391896000
6	-1.010573000	-1.153455000	4.089897000
7	-0.616421000	-1.020820000	5.376128000
1	2.654016000	1.468051000	5.003591000
1	3.608818000	-1.145361000	5.324640000
1	2.814260000	-1.557425000	2.916330000
1	2.518808000	0.162211000	2.768325000
1	1.290440000	-0.626474000	6.302452000
1	-2.037105000	-1.274356000	3.789711000
6	1.419123000	0.661234000	-4.753009000
6	2.299419000	-0.275868000	-5.550329000
8	2.221137000	-1.538070000	-5.441939000
6	0.080185000	1.006638000	-5.468578000
6	-0.843472000	-0.193515000	-5.771505000
6	-1.249117000	-0.990142000	-4.535074000
8	-1.808521000	-0.253601000	-3.548407000
8	-1.051826000	-2.216308000	-4.447146000
1	1.199254000	0.179315000	-3.794933000
1	-0.457579000	1.714493000	-4.831660000
1	0.295973000	1.528097000	-6.410461000
1	-0.376130000	-0.897223000	-6.465634000
1	-1.753905000	0.203593000	-6.235910000
7	3.177608000	0.274792000	-6.432297000
6	3.911509000	-0.534306000	-7.412173000
6	4.877494000	-1.578155000	-6.840734000
8	5.129808000	-2.606593000	-7.507962000
1	3.236922000	1.281463000	-6.491568000
1	3.213135000	-1.096953000	-8.038042000
7	5.430948000	-1.314608000	-5.624815000
6	6.325613000	-2.253449000	-4.965549000
6	5.670375000	-3.025347000	-3.818558000
8	6.375069000	-3.450543000	-2.853451000
1	5.319074000	-0.416573000	-5.161874000
1	6.671095000	-2.977296000	-5.712744000
7	4.349319000	-3.264858000	-3.928974000
6	3.608503000	-4.107777000	-2.982997000
6	2.115655000	-4.136450000	-3.323640000
8	1.579499000	-2.780247000	-3.170120000
6	1.336352000	-5.076468000	-2.408909000
1	3.831244000	-2.813078000	-4.678596000
1	3.718037000	-3.718801000	-1.965093000
1	1.978511000	-4.440815000	-4.370038000
1	1.551906000	-2.310819000	-4.047878000
1	0.275376000	-5.053853000	-2.672176000
1	1.698445000	-6.105761000	-2.509567000
1	1.440665000	-4.771542000	-1.362262000
6	-5.455402000	-4.607703000	3.326927000
6	-6.623115000	-3.833531000	3.922060000
8	-7.681976000	-3.647692000	3.257207000
6	-5.964628000	-5.837242000	2.547351000
6	-4.861180000	-6.597288000	1.763975000
6	-4.133003000	-5.677183000	0.804439000
8	-3.115303000	-5.024381000	1.173739000
7	-4.655334000	-5.499731000	-0.427964000
1	-4.910495000	-3.936643000	2.650291000
1	-6.752068000	-5.502740000	1.863403000
1	-6.437211000	-6.541988000	3.243254000
1	-5.316086000	-7.438939000	1.227641000
1	-4.117739000	-7.005721000	2.456036000
1	-4.238896000	-4.778259000	-1.017376000
1	-5.466203000	-6.006472000	-0.744962000
7	-6.466032000	-3.357586000	5.185540000
6	-7.571868000	-2.711979000	5.892561000
6	-8.104194000	-1.415913000	5.274844000
8	-9.277690000	-1.052326000	5.520103000
1	-5.533027000	-3.342476000	5.604025000
1	-8.430456000	-3.387050000	5.944517000
7	-7.235712000	-0.713270000	4.498541000
6	-7.638604000	0.498514000	3.805127000
6	-7.681600000	0.359745000	2.284127000
8	-7.641751000	1.394761000	1.566042000
1	-6.256076000	-0.990922000	4.402437000
1	-8.643556000	0.761038000	4.155204000
7	-7.808086000	-0.897117000	1.781400000
6	-7.999739000	-1.151140000	0.354278000
6	-6.818379000	-1.830721000	-0.331658000
8	-5.697781000	-0.896849000	-0.431491000
1	-7.860152000	-1.689688000	2.419632000
1	-8.196353000	-0.192186000	-0.130647000
1	-6.508683000	-2.726169000	0.223054000
1	-7.121891000	-2.130719000	-1.342221000
1	-5.048404000	-1.178965000	-1.117904000
6	-9.819869000	6.408748000	-2.949659000
6	-9.018034000	5.256235000	-2.382315000
6	-9.437513000	4.590693000	-1.216610000
6	-7.849341000	4.807925000	-3.022976000
6	-8.717503000	3.502114000	-0.710225000
6	-7.124784000	3.719402000	-2.521806000
6	-7.558500000	3.061020000	-1.363955000
1	-10.586546000	6.053911000	-3.652504000
1	-10.338147000	6.965083000	-2.160186000
1	-10.336448000	4.928179000	-0.705744000
1	-7.504391000	5.310513000	-3.922749000
1	-9.039061000	2.993794000	0.192545000
1	-6.225825000	3.392767000	-3.034320000
1	-7.010488000	2.213371000	-0.964966000
26	-0.277211000	-1.201261000	1.187842000
26	-1.845453000	-0.829587000	-1.673148000
8	0.295833000	-3.247609000	0.934476000
1	1.282823000	-3.266853000	0.843261000

1	-0.127051000	-3.857855000	1.663579000
8	-4.394240000	-0.831155000	3.992723000
1	-4.218979000	0.035702000	4.453926000
1	-4.319417000	-0.688199000	2.991525000
8	-4.121733000	-0.127498000	1.492824000
1	-4.855205000	-0.336773000	0.829619000
1	-4.009255000	0.852568000	1.641396000
8	-2.124585000	-1.451447000	0.341580000
1	-2.851614000	-2.559961000	-0.196163000
1	-2.806825000	-0.888583000	0.833353000
8	-0.980212000	-2.263167000	8.898729000
1	-0.556149000	-2.909876000	8.240630000
1	-1.021910000	-2.605196000	9.804145000
8	-3.505592000	-1.939851000	-1.931128000
8	-3.395536000	-3.044023000	-1.011618000
8	-0.352437000	-2.230189000	-1.498710000
1	0.316416000	-2.459064000	-2.216670000
8	-0.948261000	-4.617399000	2.638730000
1	-1.773394000	-4.896763000	2.164740000
1	-1.151297000	-4.383578000	3.578362000
8	-2.504702000	-0.979623000	7.340867000
1	-1.993232000	-1.359617000	8.134148000
1	-2.558898000	0.013129000	7.270493000
8	-1.888717000	-4.345175000	5.242234000
1	-2.212453000	-5.256264000	5.364415000
1	-2.660412000	-3.642732000	5.400545000
8	-0.020343000	-3.736043000	7.010856000
1	-0.709863000	-4.013148000	6.328335000
1	0.752879000	-3.353286000	6.561979000
8	-3.677455000	-2.631161000	5.708253000
1	-3.957437000	-2.021662000	4.943384000
1	-3.354295000	-2.031914000	6.465321000
1	3.760607000	2.915249000	7.099501000
1	3.730507000	6.323031000	3.616099000
1	-0.224296000	8.158935000	5.871657000
1	-1.216824000	9.029926000	1.178550000
1	-5.191305000	7.176784000	2.205334000
1	-4.908723000	8.999867000	-3.366308000
1	-1.987762000	8.136079000	-7.617523000
1	0.922567000	11.594212000	-5.411250000
1	7.019753000	5.951718000	-4.145312000
1	4.280384000	5.582837000	0.220664000
1	8.332632000	-0.027842000	-2.282774000
1	9.954073000	3.706925000	0.376288000
1	6.366874000	2.494387000	3.560649000
1	9.155112000	-1.023879000	4.236462000
1	4.471404000	0.149340000	-8.057867000
1	7.186769000	-1.721997000	-4.558818000
1	-7.240499000	-2.505012000	6.915151000
1	-6.966907000	1.327233000	4.044437000
1	-4.745966000	-4.904147000	4.107656000
1	-8.879948000	-1.794387000	0.227556000
1	8.451377000	-3.162070000	0.623158000
1	-9.181636000	7.110825000	-3.497522000
1	1.951870000	1.595581000	-4.533370000
1	4.021776000	-5.124410000	-3.003415000
1	-0.276655000	-2.837016000	-0.717088000
1	4.617706000	-0.466352000	4.053633000

^{7}IM1$_{B}$:

6	8.750765000	-3.039334000	3.200637000
6	8.047724000	-1.891063000	2.491301000
8	8.067753000	-1.833259000	1.226212000
6	7.841582000	-4.299342000	3.365376000
6	6.975460000	-4.610915000	2.184532000
7	7.433411000	-4.914223000	0.894126000
6	5.608454000	-4.600213000	2.084595000
6	6.386320000	-5.060278000	0.044471000
7	5.272210000	-4.875231000	0.764588000
1	9.627525000	-3.288950000	2.595038000
1	8.482845000	-5.153126000	3.619716000
1	7.174347000	-4.148810000	4.220378000
1	4.860293000	-4.409313000	2.832355000
1	6.425337000	-5.255136000	-1.029636000
1	4.266735000	-4.771649000	0.402837000
7	7.408133000	-0.975225000	3.259416000
6	6.511036000	0.051903000	2.717406000
6	7.032387000	0.841975000	1.547568000
8	6.241389000	1.218647000	0.636758000
1	7.289117000	-1.113829000	4.261905000
1	5.553498000	-0.371043000	2.401202000
7	8.341105000	1.178193000	1.466121000
6	8.815288000	1.951756000	0.315401000
6	8.409685000	1.342406000	-1.033790000
8	8.162175000	2.087775000	-2.013746000
1	8.975108000	0.920617000	2.207655000
1	8.406940000	2.965532000	0.327604000
7	8.372815000	-0.017797000	-1.114082000
6	8.022735000	-0.675197000	-2.366820000
6	6.535372000	-0.667884000	-2.736544000
8	6.199068000	-1.055768000	-3.887520000
1	8.452154000	-0.589458000	-0.273289000
1	8.542919000	-0.188867000	-3.196313000
7	5.660031000	-0.257598000	-1.792146000
6	4.225752000	-0.074331000	-2.067986000
6	3.884071000	1.425177000	-2.109301000
8	3.313856000	1.929015000	-3.118555000
6	3.326221000	-0.817392000	-1.060083000
6	3.498708000	-2.342399000	-1.095391000
6	2.560309000	-3.053819000	-0.133646000
8	1.532591000	-2.415064000	0.275402000
8	2.819118000	-4.282967000	0.223529000
1	6.015456000	0.071235000	-0.896182000
1	4.057523000	-0.449563000	-3.078452000
1	2.283284000	-0.575344000	-1.290894000
1	3.503805000	-0.440411000	-0.045944000
1	4.534337000	-2.627792000	-0.876678000
1	3.268955000	-2.742647000	-2.094134000
7	4.234159000	2.145933000	-1.017270000
6	3.931495000	3.562408000	-0.860946000
6	4.636435000	4.498083000	-1.830810000
8	4.178639000	5.667765000	-1.987392000
1	4.697579000	1.695149000	-0.230022000
1	2.860412000	3.761054000	-0.990391000
7	5.671007000	4.036720000	-2.568975000
6	6.153431000	4.814211000	-3.710026000
6	5.058198000	5.125868000	-4.743865000
8	5.214139000	6.084028000	-5.534872000
1	6.138826000	3.166422000	-2.337594000
1	6.551686000	5.782353000	-3.395208000
7	3.951463000	4.323635000	-4.743028000
6	2.810240000	4.586565000	-5.616474000
6	1.606450000	5.192347000	-4.869288000
8	0.487667000	5.272270000	-5.447263000
6	2.378451000	3.319876000	-6.374856000
1	3.879575000	3.513799000	-4.126026000
1	3.144282000	5.347644000	-6.333119000
1	1.479243000	3.524884000	-6.959302000
1	3.183783000	2.999789000	-7.043287000
1	2.158083000	2.512220000	-5.667619000
7	1.799553000	5.687612000	-3.615385000
6	0.663886000	6.150710000	-2.810483000
6	-0.024412000	7.397618000	-3.386428000
8	-1.190688000	7.691983000	-3.011996000
6	1.064591000	6.410266000	-1.350765000
8	0.982950000	5.149531000	-0.617728000
1	2.712619000	5.628306000	-3.161659000
1	-0.117138000	5.381360000	-2.811445000
1	2.079887000	6.816696000	-1.297321000
1	0.356412000	7.134220000	-0.930209000
1	1.135602000	5.289460000	0.348187000
7	0.668658000	8.131689000	-4.290689000
6	0.060190000	9.250675000	-5.001952000
6	-1.039753000	8.875172000	-6.006921000
8	-1.723019000	9.794517000	-6.512480000
1	1.598915000	7.825836000	-4.540627000
1	-0.406567000	9.935830000	-4.288764000
7	-1.219130000	7.552033000	-6.277737000
6	-2.326698000	7.096205000	-7.107885000
6	-3.552695000	6.591690000	-6.338257000

8	-4.505462000	6.078260000	-6.978028000
1	-0.595280000	6.839998000	-5.899034000
1	-2.652838000	7.929579000	-7.736875000
7	-3.541718000	6.742031000	-4.987836000
6	-4.675178000	6.360163000	-4.145582000
6	-4.333349000	5.298177000	-3.086030000
6	-4.000493000	3.894627000	-3.576774000
6	-4.283448000	3.436649000	-4.876492000
6	-3.438251000	2.978666000	-2.669557000
6	-4.020257000	2.112982000	-5.246509000
6	-3.177334000	1.652271000	-3.022442000
6	-3.470132000	1.216546000	-4.323138000
8	-3.230479000	-0.082808000	-4.742588000
1	-2.736152000	7.158145000	-4.532148000
1	-5.467909000	6.017309000	-4.814725000
1	-5.201292000	5.236765000	-2.408907000
1	-3.496950000	5.677201000	-2.478999000
1	-4.692528000	4.109877000	-5.623007000
1	-3.209973000	3.306977000	-1.656997000
1	-4.233942000	1.764023000	-6.250460000
1	-2.753744000	0.954822000	-2.307322000
1	-2.796438000	-0.660844000	-4.063197000
6	-4.714171000	4.455511000	2.262968000
6	-3.235558000	4.724932000	2.143653000
8	-2.365666000	3.851553000	2.423104000
6	-5.213196000	3.364766000	1.255491000
6	-5.954960000	2.212050000	1.955233000
6	-4.974006000	1.299414000	2.696696000
8	-4.051987000	0.701696000	2.075461000
8	-5.182439000	1.263179000	3.999191000
1	-4.886512000	4.094911000	3.285120000
1	-5.886143000	3.823699000	0.524377000
1	-4.371270000	2.942139000	0.697989000
1	-6.469652000	1.580418000	1.225689000
1	-6.714972000	2.588208000	2.645336000
7	-2.831250000	5.971965000	1.746110000
6	-1.428362000	6.223237000	1.400961000
6	-0.444593000	5.903215000	2.523860000
8	0.702562000	5.450400000	2.245867000
1	-3.525800000	6.635410000	1.432471000
1	-1.116737000	5.619766000	0.542237000
7	-0.819524000	6.161845000	3.801300000
6	0.060682000	5.864953000	4.935791000
6	0.175407000	4.367788000	5.278589000
8	1.248257000	3.920891000	5.755110000
1	-1.741407000	6.542421000	3.964928000
1	1.071604000	6.214528000	4.719669000
7	-0.931342000	3.618137000	5.060275000
6	-0.973102000	2.150331000	5.247258000
6	0.039704000	1.503266000	4.278572000
8	0.936740000	0.721979000	4.690826000
6	-2.428112000	1.704098000	5.001217000
6	-2.824102000	0.353231000	5.574786000
8	-2.199102000	-0.164390000	6.550734000
8	-3.874212000	-0.217031000	5.034812000
1	-1.674812000	4.009842000	4.494314000
1	-0.651676000	1.891866000	6.257516000
1	-2.653868000	1.695385000	3.934266000
1	-3.102318000	2.438849000	5.461901000
7	-0.058102000	1.892250000	2.981105000
6	0.863510000	1.387491000	1.957262000
6	2.304950000	1.835598000	2.238084000
8	3.279796000	1.077476000	1.983687000
6	0.411389000	1.827469000	0.544856000
6	-0.976561000	1.314522000	0.083865000
6	-1.077933000	-0.195631000	0.017946000
8	-0.773387000	-0.858628000	1.065994000
8	-1.471541000	-0.735498000	-1.104039000
1	-0.833556000	2.480881000	2.690136000
1	0.873756000	0.298272000	2.001182000
1	1.173365000	1.480852000	-0.162319000
1	0.403643000	2.921746000	0.476464000
1	-1.765475000	1.657983000	0.765589000
1	-1.185130000	1.731510000	-0.903371000
7	2.476098000	3.081481000	2.759294000
6	3.785939000	3.585902000	3.153155000
6	4.492260000	2.732815000	4.207990000
8	5.738853000	2.561254000	4.131033000
1	1.686169000	3.711650000	2.836910000
1	4.478860000	3.620864000	2.307763000
7	3.740302000	2.193920000	5.197630000
6	4.340488000	1.265315000	6.156587000
6	4.555341000	-0.149002000	5.589732000
8	5.688893000	-0.712966000	5.661607000
1	2.758579000	2.441910000	5.289646000
1	5.322467000	1.630210000	6.461630000
7	3.478329000	-0.751617000	5.037906000
6	3.591836000	-2.093085000	4.451558000
6	2.498201000	-2.374586000	3.416245000
6	1.136492000	-2.588740000	4.002076000
7	0.012786000	-2.808036000	3.197120000
6	0.721181000	-2.573909000	5.319190000
6	-1.044782000	-2.902479000	4.018320000
7	-0.649668000	-2.772225000	5.303450000
1	2.599161000	-0.241818000	4.921544000
1	3.574242000	-2.850718000	5.249152000
1	2.782744000	-3.263885000	2.836672000
1	2.469313000	-1.546226000	2.697771000
1	1.254582000	-2.366308000	6.230264000
1	-2.070677000	-3.030492000	3.718831000
6	1.370791000	-1.084686000	-4.697629000
6	2.247558000	-2.001260000	-5.520338000
8	2.152180000	-3.266831000	-5.456168000
6	0.014673000	-0.752864000	-5.386966000
6	-0.899236000	-1.960368000	-5.680712000
6	-1.300508000	-2.772888000	-4.454070000
8	-1.743959000	-2.021314000	-3.414855000
8	-1.225458000	-4.012956000	-4.426765000
1	1.170441000	-1.578719000	-3.741064000
1	-0.518811000	-0.055556000	-4.735628000
1	0.207424000	-0.223115000	-6.329145000
1	-0.436821000	-2.659435000	-6.382974000
1	-1.818254000	-1.570518000	-6.135544000
7	3.139731000	-1.434336000	-6.375163000
6	3.877358000	-2.221900000	-7.370574000
6	4.804567000	-3.309285000	-6.815780000
8	5.021221000	-4.336222000	-7.497404000
1	3.213021000	-0.427017000	-6.400570000
1	3.181367000	-2.743611000	-8.033322000
7	5.366599000	-3.082688000	-5.595979000
6	6.233017000	-4.059508000	-4.953625000
6	5.553450000	-4.840625000	-3.826692000
8	6.239727000	-5.293485000	-2.861277000
1	5.296130000	-2.182985000	-5.126845000
1	6.564712000	-4.775120000	-5.714985000
7	4.229871000	-5.056318000	-3.954641000
6	3.462926000	-5.897382000	-3.026876000
6	1.973732000	-5.894714000	-3.385691000
8	1.460521000	-4.529503000	-3.229547000
6	1.163316000	-6.825068000	-2.488876000
1	3.730034000	-4.589346000	-4.707065000
1	3.569487000	-5.522689000	-2.003252000
1	1.843534000	-6.188970000	-4.436004000
1	1.494256000	-4.040609000	-4.098438000
1	0.106780000	-6.778322000	-2.766539000
1	1.505769000	-7.860617000	-2.593369000
1	1.259214000	-6.530999000	-1.438349000
6	-5.471933000	-6.326187000	3.212478000
6	-6.653253000	-5.613530000	3.855962000
8	-7.734166000	-5.450453000	3.223264000
6	-5.947881000	-7.549751000	2.404034000
6	-4.851007000	-8.184715000	1.506068000
6	-4.310530000	-7.175000000	0.514941000
8	-3.352842000	-6.403417000	0.831729000
7	-4.932576000	-7.046891000	-0.672813000
1	-4.971114000	-5.612941000	2.545297000
1	-6.800827000	-7.238548000	1.791194000
1	-6.319790000	-8.321944000	3.089366000
1	-5.269994000	-9.054634000	0.986416000
1	-4.012537000	-8.533221000	2.117987000

1	-4.649365000	-6.268501000	-1.274832000
1	-5.703466000	-7.638527000	-0.938997000
7	-6.479109000	-5.157177000	5.125063000
6	-7.584183000	-4.547411000	5.862409000
6	-8.130476000	-3.232941000	5.297977000
8	-9.292875000	-2.872669000	5.595491000
1	-5.538440000	-5.125773000	5.524290000
1	-8.436920000	-5.230993000	5.893868000
7	-7.287649000	-2.510368000	4.510933000
6	-7.709637000	-1.271617000	3.878521000
6	-7.805031000	-1.343077000	2.355459000
8	-7.835846000	-0.271558000	1.693182000
1	-6.314036000	-2.787671000	4.371538000
1	-8.700475000	-1.020371000	4.273229000
7	-7.892081000	-2.577174000	1.793146000
6	-8.099099000	-2.768238000	0.356645000
6	-6.902893000	-3.361289000	-0.382183000
8	-5.819227000	-2.388609000	-0.453006000
1	-7.907364000	-3.398546000	2.394282000
1	-8.343344000	-1.794571000	-0.073567000
1	-6.551371000	-4.276359000	0.111835000
1	-7.213219000	-3.619588000	-1.402327000
1	-5.152786000	-2.628116000	-1.160565000
6	-9.882623000	4.703263000	-2.834763000
6	-9.068855000	3.525693000	-2.340391000
6	-9.431725000	2.837775000	-1.169275000
6	-7.945656000	3.075648000	-3.057439000
6	-8.702903000	1.725325000	-0.730354000
6	-7.212472000	1.964085000	-2.624878000
6	-7.590315000	1.283365000	-1.459922000
1	-10.647332000	4.385056000	-3.557006000
1	-10.403534000	5.204959000	-2.011442000
1	-10.294616000	3.175609000	-0.599753000
1	-7.643972000	3.594816000	-3.963549000
1	-8.980103000	1.201730000	0.178330000
1	-6.349042000	1.636596000	-3.194544000
1	-7.031795000	0.416687000	-1.118452000
26	-0.310119000	-2.907889000	1.126945000
26	-1.961652000	-2.625690000	-1.625415000
8	0.212126000	-4.959679000	0.801778000
1	1.199617000	-5.009903000	0.742824000
1	-0.274365000	-5.574990000	1.493149000
8	-4.441422000	-2.596733000	3.914996000
1	-4.272621000	-1.730124000	4.378463000
1	-4.354627000	-2.457841000	2.914185000
8	-4.121365000	-1.895708000	1.414459000
1	-4.900188000	-2.047918000	0.768444000
1	-3.986203000	-0.923640000	1.591357000
8	-2.122343000	-3.128895000	0.208329000
1	-3.277924000	-4.477714000	-0.409538000
1	-2.856981000	-2.615714000	0.694920000
8	-0.985710000	-4.110102000	8.750329000
1	-0.584925000	-4.742970000	8.065650000
1	-0.999635000	-4.472476000	9.648659000
8	-3.744043000	-3.254267000	-1.926712000
8	-3.884731000	-4.575692000	-1.199633000
8	-0.470810000	-3.944738000	-1.598920000
1	0.177302000	-4.187073000	-2.334447000
8	-1.201538000	-6.277466000	2.387792000
1	-2.049516000	-6.414875000	1.886890000
1	-1.364379000	-6.123079000	3.352602000
8	-2.528329000	-2.763653000	7.266530000
1	-2.008180000	-3.164906000	8.042630000
1	-2.585777000	-1.769659000	7.216326000
8	-1.967976000	-6.124054000	5.050232000
1	-2.293026000	-7.031869000	5.193310000
1	-2.722082000	-5.409878000	5.261226000
8	-0.064330000	-5.537383000	6.801375000
1	-0.757801000	-5.805427000	6.120480000
1	0.693521000	-5.122616000	6.354166000
8	-3.700557000	-4.393719000	5.611075000
1	-3.995313000	-3.775018000	4.856432000
1	-3.373960000	-3.805633000	6.374765000
1	3.685749000	1.211582000	7.031309000
1	3.648230000	4.603005000	3.532079000
1	-0.310808000	6.405930000	5.811240000
1	-1.331446000	7.278807000	1.126215000
1	-5.289283000	5.380785000	2.147185000
1	-5.047400000	7.255355000	-3.628972000
1	-1.994932000	6.283111000	-7.759090000
1	0.844068000	9.798430000	-5.531416000
1	6.959670000	4.249661000	-4.186117000
1	4.192221000	3.848484000	0.161257000
1	8.355349000	-1.716445000	-2.322121000
1	9.906299000	2.016260000	0.370232000
1	6.302925000	0.770434000	3.518414000
1	9.107272000	-2.739932000	4.191888000
1	4.467437000	-1.526125000	-7.975064000
1	7.105299000	-3.558965000	-4.531125000
1	-7.249210000	-4.375228000	6.890231000
1	-7.027127000	-0.453779000	4.126467000
1	-4.732779000	-6.617542000	3.967287000
1	-8.956205000	-3.438812000	0.213260000
1	8.398998000	-4.928199000	0.604879000
1	-9.252841000	5.444096000	-3.340333000
1	1.893803000	-0.145065000	-4.478610000
1	3.857535000	-6.921127000	-3.055867000
1	-0.355505000	-4.517566000	-0.790745000
1	4.576869000	-2.162907000	3.978260000

^{7}TS2$_{B}$:

6	8.770807000	-3.040586000	3.151948000
6	8.066148000	-1.893088000	2.442660000
8	8.098704000	-1.823223000	1.178513000
6	7.851985000	-4.289301000	3.342634000
6	6.985093000	-4.612980000	2.165899000
7	7.445313000	-4.924207000	0.878259000
6	5.618119000	-4.603210000	2.063490000
6	6.398717000	-5.075648000	0.028314000
7	5.283402000	-4.886443000	0.744872000
1	9.636671000	-3.303366000	2.536500000
1	8.486444000	-5.143355000	3.612863000
1	7.185980000	-4.116468000	4.194388000
1	4.868496000	-4.408214000	2.808754000
1	6.444226000	-5.273948000	-1.045055000
1	4.272455000	-4.780596000	0.380425000
7	7.409975000	-0.991471000	3.213861000
6	6.501209000	0.027702000	2.677744000
6	7.012444000	0.834672000	1.515816000
8	6.214883000	1.216960000	0.612559000
1	7.280971000	-1.143652000	4.213542000
1	5.548814000	-0.404595000	2.358525000
7	8.317918000	1.181861000	1.431998000
6	8.779963000	1.968379000	0.285576000
6	8.365883000	1.369605000	-1.065933000
8	8.100661000	2.123342000	-2.035491000
1	8.958508000	0.916243000	2.164904000
1	8.367163000	2.980128000	0.310295000
7	8.341955000	0.010392000	-1.160423000
6	7.986493000	-0.637919000	-2.416794000
6	6.497000000	-0.629770000	-2.778748000
8	6.154434000	-0.999093000	-3.934291000
1	8.437240000	-0.569192000	-0.326254000
1	8.502194000	-0.145612000	-3.245539000
7	5.626445000	-0.239265000	-1.822192000
6	4.187704000	-0.068770000	-2.082148000
6	3.832783000	1.427801000	-2.103680000
8	3.256014000	1.942389000	-3.105051000
6	3.305836000	-0.833073000	-1.074395000
6	3.502109000	-2.355266000	-1.120867000
6	2.565299000	-3.085193000	-0.169552000
8	1.523992000	-2.466313000	0.226169000
8	2.843593000	-4.310309000	0.190123000
1	5.985487000	0.077947000	-0.923471000
1	4.015267000	-0.436167000	-3.094585000
1	2.257213000	-0.607786000	-1.296647000
1	3.482320000	-0.459051000	-0.058844000
1	4.540427000	-2.627282000	-0.897657000
1	3.281244000	-2.751662000	-2.122959000
7	4.179511000	2.138098000	-1.003939000

6	3.866851000	3.549839000	-0.829304000
6	4.556091000	4.500689000	-1.795625000
8	4.079210000	5.664119000	-1.948319000
1	4.646442000	1.680788000	-0.222026000
1	2.793559000	3.741812000	-0.947183000
7	5.598552000	4.060265000	-2.534532000
6	6.070856000	4.852185000	-3.669944000
6	4.969846000	5.160703000	-4.698013000
8	5.108340000	6.130817000	-5.477772000
1	6.079281000	3.194833000	-2.309497000
1	6.460212000	5.821706000	-3.348315000
7	3.874997000	4.341813000	-4.705361000
6	2.729848000	4.599636000	-5.574132000
6	1.517371000	5.170528000	-4.814683000
8	0.388123000	5.220071000	-5.379011000
6	2.321878000	3.340808000	-6.357683000
1	3.814241000	3.524042000	-4.096756000
1	3.050839000	5.380814000	-6.275531000
1	1.420860000	3.540579000	-6.941285000
1	3.134321000	3.048203000	-7.029797000
1	2.115759000	2.515416000	-5.667084000
7	1.709436000	5.674780000	-3.565047000
6	0.567864000	6.119441000	-2.757867000
6	-0.167610000	7.322283000	-3.365184000
8	-1.361921000	7.554879000	-3.035999000
6	0.976739000	6.430127000	-1.308694000
8	0.895918000	5.191975000	-0.540901000
1	2.624707000	5.626767000	-3.111790000
1	-0.185853000	5.324767000	-2.724671000
1	1.992427000	6.837224000	-1.274388000
1	0.272230000	7.168005000	-0.905042000
1	1.088175000	5.346844000	0.416006000
7	0.519691000	8.093905000	-4.242140000
6	-0.110958000	9.195273000	-4.962447000
6	-1.189787000	8.789936000	-5.977606000
8	-1.905892000	9.686320000	-6.478092000
1	1.471557000	7.832939000	-4.461160000
1	-0.602589000	9.871770000	-4.257572000
7	-1.317231000	7.463417000	-6.264520000
6	-2.405598000	6.976518000	-7.100622000
6	-3.596934000	6.397634000	-6.331861000
8	-4.509506000	5.805435000	-6.964033000
1	-0.675946000	6.768878000	-5.881699000
1	-2.775071000	7.810880000	-7.704639000
7	-3.607887000	6.572936000	-4.983544000
6	-4.732760000	6.155498000	-4.148029000
6	-4.366526000	5.083324000	-3.107800000
6	-4.007304000	3.700423000	-3.612849000
6	-4.270089000	3.259108000	-4.934117000
6	-3.432312000	2.781215000	-2.696627000
6	-3.985811000	1.955573000	-5.310833000
6	-3.147759000	1.474240000	-3.048764000
6	-3.428717000	1.034493000	-4.376958000
8	-3.198552000	-0.213311000	-4.773622000
1	-2.832518000	7.043941000	-4.526107000
1	-5.523517000	5.811517000	-4.819035000
1	-5.228504000	4.976618000	-2.427917000
1	-3.537892000	5.464495000	-2.492455000
1	-4.675482000	3.944985000	-5.671244000
1	-3.226331000	3.113402000	-1.682726000
1	-4.178644000	1.605273000	-6.318137000
1	-2.709861000	0.774403000	-2.343104000
1	-2.577031000	-0.962621000	-4.159156000
6	-4.689674000	4.541951000	2.462134000
6	-3.215064000	4.814048000	2.303759000
8	-2.339957000	3.927325000	2.510057000
6	-5.223324000	3.496808000	1.424521000
6	-5.970296000	2.331737000	2.098158000
6	-4.983311000	1.404298000	2.815460000
8	-4.068205000	0.820148000	2.172069000
8	-5.179239000	1.350307000	4.118753000
1	-4.826450000	4.132385000	3.471018000
1	-5.897936000	3.993039000	0.718965000
1	-4.393428000	3.076488000	0.846897000
1	-6.489231000	1.715750000	1.358135000
1	-6.722940000	2.697280000	2.802315000
7	-2.816906000	6.076867000	1.947891000
6	-1.422570000	6.330092000	1.570763000
6	-0.409886000	5.970276000	2.656308000
8	0.725027000	5.518003000	2.333741000
1	-3.515786000	6.762191000	1.698269000
1	-1.138927000	5.745537000	0.689479000
7	-0.752559000	6.195467000	3.949163000
6	0.148305000	5.859661000	5.055888000
6	0.241854000	4.355010000	5.370463000
8	1.307892000	3.887362000	5.841326000
1	-1.668449000	6.575548000	4.144609000
1	1.160180000	6.193951000	4.820837000
7	-0.873634000	3.623807000	5.134994000
6	-0.937178000	2.153946000	5.299225000
6	0.058858000	1.507687000	4.312890000
8	0.963774000	0.728329000	4.710011000
6	-2.399605000	1.731489000	5.059801000
6	-2.810375000	0.389031000	5.641389000
8	-2.165806000	-0.152000000	6.590377000
8	-3.893445000	-0.152666000	5.135895000
1	-1.610742000	4.035383000	4.575470000
1	-0.610867000	1.875611000	6.302622000
1	-2.631923000	1.717893000	3.993910000
1	-3.061946000	2.477241000	5.519617000
7	-0.056481000	1.901823000	3.017998000
6	0.854913000	1.405389000	1.979847000
6	2.299433000	1.843081000	2.256997000
8	3.271589000	1.093725000	1.971191000
6	0.398934000	1.876417000	0.578572000
6	-0.980457000	1.358944000	0.099976000
6	-1.058855000	-0.152547000	-0.032803000
8	-0.709583000	-0.842357000	0.983508000
8	-1.481796000	-0.655619000	-1.157025000
1	-0.837247000	2.488449000	2.738679000
1	0.854273000	0.315125000	2.000043000
1	1.166096000	1.555317000	-0.135396000
1	0.380654000	2.972249000	0.540855000
1	-1.774227000	1.662020000	0.796024000
1	-1.194606000	1.815533000	-0.869186000
7	2.478880000	3.077406000	2.805525000
6	3.793977000	3.569444000	3.196798000
6	4.509555000	2.690944000	4.224833000
8	5.756574000	2.526806000	4.135672000
1	1.690196000	3.704347000	2.907839000
1	4.478046000	3.622110000	2.344990000
7	3.764433000	2.124698000	5.203446000
6	4.367247000	1.174022000	6.138539000
6	4.556712000	-0.234966000	5.549730000
8	5.687387000	-0.808816000	5.595308000
1	2.780432000	2.359804000	5.299600000
1	5.357724000	1.523914000	6.433076000
7	3.464664000	-0.820844000	5.011370000
6	3.550591000	-2.159090000	4.411364000
6	2.456821000	-2.410624000	3.368067000
6	1.087688000	-2.608403000	3.943054000
7	-0.030089000	-2.818919000	3.128052000
6	0.663735000	-2.591104000	5.258077000
6	-1.093262000	-2.907611000	3.941075000
7	-0.708318000	-2.779766000	5.231973000
1	2.588606000	-0.301427000	4.918126000
1	3.512783000	-2.923377000	5.201767000
1	2.730073000	-3.299848000	2.783071000
1	2.445331000	-1.576783000	2.655422000
1	1.190803000	-2.391417000	6.174756000
1	-2.116255000	-3.033735000	3.631814000
6	1.515073000	-1.045560000	-4.837175000
6	2.380079000	-1.998494000	-5.635852000
8	2.258889000	-3.256997000	-5.550887000
6	0.196920000	-0.653340000	-5.565046000
6	-0.755093000	-1.828362000	-5.887335000
6	-1.177464000	-2.614046000	-4.651624000
8	-1.844770000	-1.901345000	-3.703317000
8	-0.891577000	-3.811529000	-4.506708000
1	1.271108000	-1.534441000	-3.888317000

1	-0.324189000	0.066735000	-4.925707000
1	0.438468000	-0.131483000	-6.500369000
1	-0.287826000	-2.539248000	-6.573277000
1	-1.651399000	-1.418655000	-6.367211000
7	3.291399000	-1.457305000	-6.491441000
6	4.027076000	-2.274079000	-7.462552000
6	4.963779000	-3.337745000	-6.878465000
8	5.223012000	-4.360294000	-7.551768000
1	3.397394000	-0.453329000	-6.518960000
1	3.329083000	-2.820826000	-8.102725000
7	5.483391000	-3.093826000	-5.643867000
6	6.349903000	-4.048601000	-4.971081000
6	5.662363000	-4.825336000	-3.846439000
8	6.350095000	-5.282724000	-2.883831000
1	5.371813000	-2.195142000	-5.180367000
1	6.707509000	-4.769032000	-5.715912000
7	4.337871000	-5.032532000	-3.971106000
6	3.571349000	-5.870560000	-3.039548000
6	2.084002000	-5.881741000	-3.403877000
8	1.560762000	-4.519827000	-3.254046000
6	1.280852000	-6.814033000	-2.501880000
1	3.835305000	-4.560315000	-4.718553000
1	3.668065000	-5.486193000	-2.018692000
1	1.961984000	-6.184741000	-4.452884000
1	1.511225000	-4.065211000	-4.133980000
1	0.225692000	-6.789159000	-2.787476000
1	1.640544000	-7.845717000	-2.587176000
1	1.360640000	-6.500790000	-1.455926000
6	-5.603720000	-6.270255000	3.220762000
6	-6.752305000	-5.505423000	3.862971000
8	-7.831055000	-5.309295000	3.234456000
6	-6.134536000	-7.500121000	2.455538000
6	-5.058757000	-8.236730000	1.615016000
6	-4.376842000	-7.288339000	0.646167000
8	-3.293469000	-6.712123000	0.953379000
7	-5.009581000	-7.002118000	-0.509978000
1	-5.092406000	-5.591652000	2.525388000
1	-6.954675000	-7.166310000	1.810706000
1	-6.568837000	-8.215082000	3.166139000
1	-5.529842000	-9.068852000	1.077040000
1	-4.284606000	-8.655569000	2.265543000
1	-4.640128000	-6.228497000	-1.076296000
1	-5.880599000	-7.442123000	-0.761266000
7	-6.557291000	-5.047066000	5.128120000
6	-7.640321000	-4.404740000	5.871421000
6	-8.180970000	-3.096715000	5.286149000
8	-9.345799000	-2.730603000	5.568915000
1	-5.613040000	-5.041427000	5.521279000
1	-8.501916000	-5.074933000	5.936041000
7	-7.329354000	-2.386101000	4.498916000
6	-7.743990000	-1.160086000	3.837831000
6	-7.812117000	-1.263654000	2.314161000
8	-7.804764000	-0.207519000	1.623926000
1	-6.351975000	-2.665043000	4.375981000
1	-8.742243000	-0.904879000	4.211318000
7	-7.923394000	-2.508001000	1.785082000
6	-8.104115000	-2.744967000	0.351932000
6	-6.895886000	-3.368914000	-0.342690000
8	-5.810645000	-2.405584000	-0.443565000
1	-7.956256000	-3.313421000	2.407912000
1	-8.338083000	-1.786557000	-0.116562000
1	-6.558132000	-4.263128000	0.198276000
1	-7.193334000	-3.675139000	-1.354100000
1	-5.121005000	-2.700170000	-1.127304000
6	-9.542154000	4.767800000	-3.021180000
6	-8.839954000	3.545760000	-2.466885000
6	-9.185943000	3.023208000	-1.207929000
6	-7.849277000	2.883354000	-3.213365000
6	-8.569198000	1.868551000	-0.710690000
6	-7.227488000	1.729521000	-2.719469000
6	-7.583439000	1.217207000	-1.465816000
1	-10.449139000	4.489266000	-3.575431000
1	-9.850460000	5.452158000	-2.222439000
1	-9.951842000	3.521595000	-0.618005000
1	-7.570638000	3.268347000	-4.191321000
1	-8.840822000	1.462259000	0.257534000
1	-6.467739000	1.232235000	-3.314362000
1	-7.106678000	0.324797000	-1.072232000
26	-0.355891000	-2.931020000	1.032680000
26	-2.036161000	-2.576788000	-1.716212000
8	0.209424000	-4.996331000	0.768751000
1	1.195095000	-5.038887000	0.707832000
1	-0.249459000	-5.608951000	1.473476000
8	-4.501896000	-2.521868000	3.966345000
1	-4.311745000	-1.670116000	4.443610000
1	-4.426725000	-2.362083000	2.960628000
8	-4.226820000	-1.808652000	1.498060000
1	-4.955151000	-1.989302000	0.803698000
1	-4.071674000	-0.838230000	1.635056000
8	-2.199159000	-3.155941000	0.294669000
1	-3.209440000	-4.351658000	-0.371040000
1	-2.891512000	-2.620814000	0.786412000
8	-0.914857000	-4.106989000	8.688928000
1	-0.534211000	-4.741554000	7.991459000
1	-0.918286000	-4.478247000	9.583907000
8	-3.866761000	-3.340834000	-1.950868000
8	-3.818112000	-4.620263000	-1.138244000
8	-0.499911000	-3.953580000	-1.645054000
1	0.156369000	-4.182859000	-2.361270000
8	-1.106827000	-6.374334000	2.415131000
1	-1.936343000	-6.618949000	1.926564000
1	-1.314038000	-6.132949000	3.351036000
8	-2.529506000	-2.768145000	7.267414000
1	-1.971400000	-3.178747000	8.012148000
1	-2.569607000	-1.774135000	7.233166000
8	-2.017246000	-6.098944000	5.042769000
1	-2.355469000	-7.007278000	5.142811000
1	-2.769458000	-5.387045000	5.243069000
8	-0.061276000	-5.543621000	6.725429000
1	-0.788168000	-5.809382000	6.076679000
1	0.670887000	-5.123415000	6.241436000
8	-3.757007000	-4.364042000	5.609226000
1	-4.047790000	-3.725354000	4.868523000
1	-3.413712000	-3.794694000	6.377312000
1	3.725704000	1.115410000	7.022730000
1	3.663719000	4.577900000	3.600574000
1	-0.189958000	6.391453000	5.950247000
1	-1.325098000	7.392484000	1.324117000
1	-5.267712000	5.471292000	2.409971000
1	-5.116961000	7.032280000	-3.611252000
1	-2.043710000	6.197236000	-7.776439000
1	0.664880000	9.761027000	-5.484876000
1	6.881644000	4.299261000	-4.151946000
1	4.133959000	3.826627000	0.193816000
1	8.320107000	-1.679203000	-2.380040000
1	9.871094000	2.037056000	0.332543000
1	6.286159000	0.737811000	3.484311000
1	9.143600000	-2.733398000	4.134910000
1	4.610686000	-1.598331000	-8.095383000
1	7.208483000	-3.532127000	-4.538491000
1	-7.281741000	-4.213558000	6.888000000
1	-7.067448000	-0.336893000	4.083497000
1	-4.861457000	-6.563637000	3.971575000
1	-8.961237000	-3.417582000	0.218759000
1	8.411067000	-4.941457000	0.589994000
1	-8.899345000	5.322230000	-3.714080000
1	2.068126000	-0.128125000	-4.598737000
1	3.974992000	-6.890866000	-3.057674000
1	-0.398969000	-4.548030000	-0.855704000
1	4.537023000	-2.242565000	3.943413000

^{7}IM2$_{B}$:

6	8.716072000	-2.873393000	2.645240000
6	7.940122000	-1.722704000	2.020748000
8	7.857763000	-1.641960000	0.759706000
6	7.875210000	-4.186741000	2.763249000
6	6.956209000	-4.452655000	1.612121000
7	7.350762000	-4.698996000	0.288749000
6	5.586619000	-4.429644000	1.578008000
6	6.262861000	-4.799012000	-0.515926000

7	5.187247000	-4.641048000	0.264924000
1	9.581894000	-3.047567000	1.998503000
1	8.567801000	-5.023798000	2.918127000
1	7.253118000	-4.131598000	3.662617000
1	4.875677000	-4.270151000	2.368270000
1	6.234098000	-4.947403000	-1.597153000
1	4.164668000	-4.528550000	-0.036744000
7	7.358565000	-0.823737000	2.853098000
6	6.431375000	0.218346000	2.394430000
6	6.888491000	1.040364000	1.217995000
8	6.043319000	1.480363000	0.387268000
1	7.312324000	-0.984513000	3.858413000
1	5.457005000	-0.196435000	2.120848000
7	8.200868000	1.329532000	1.051577000
6	8.629313000	2.109022000	-0.111338000
6	8.106916000	1.553516000	-1.443710000
8	7.837047000	2.340345000	-2.385823000
1	8.875108000	1.012041000	1.731663000
1	8.269366000	3.139236000	-0.049436000
7	7.985982000	0.201645000	-1.552716000
6	7.515868000	-0.408991000	-2.790935000
6	6.000058000	-0.400620000	-3.023266000
8	5.556135000	-0.858797000	-4.108693000
1	8.101152000	-0.396176000	-0.733843000
1	7.956571000	0.115179000	-3.643645000
7	5.217506000	0.101729000	-2.040923000
6	3.766638000	0.300125000	-2.211691000
6	3.461775000	1.803891000	-2.109878000
8	2.958176000	2.441980000	-3.077774000
6	2.925996000	-0.528989000	-1.215824000
6	3.159045000	-2.042416000	-1.330300000
6	2.346106000	-2.844507000	-0.322325000
8	1.334691000	-2.286390000	0.221194000
8	2.701000000	-4.074291000	-0.063539000
1	5.648669000	0.455133000	-1.187887000
1	3.534769000	0.012490000	-3.238806000
1	1.866822000	-0.318092000	-1.401778000
1	3.124945000	-0.202329000	-0.188795000
1	4.221049000	-2.284028000	-1.205430000
1	2.879323000	-2.407916000	-2.329697000
7	3.791575000	2.404154000	-0.940938000
6	3.543918000	3.817157000	-0.697042000
6	4.378058000	4.788328000	-1.517449000
8	4.005287000	5.997549000	-1.588068000
1	4.222128000	1.877756000	-0.183273000
1	2.498935000	4.074593000	-0.905200000
7	5.444913000	4.328740000	-2.204620000
6	6.119716000	5.174541000	-3.187487000
6	5.184656000	5.697546000	-4.285468000
8	5.501733000	6.722549000	-4.930692000
1	5.801533000	3.390796000	-2.062618000
1	6.553863000	6.061381000	-2.718008000
7	4.024269000	5.005643000	-4.500778000
6	3.018411000	5.480269000	-5.448930000
6	1.725222000	5.926308000	-4.743416000
8	0.644823000	6.020948000	-5.389558000
6	2.725279000	4.442602000	-6.543791000
1	3.828686000	4.134688000	-4.009952000
1	3.452866000	6.377922000	-5.910076000
1	1.914636000	4.791836000	-7.186838000
1	3.624505000	4.287058000	-7.146649000
1	2.428773000	3.483012000	-6.108834000
7	1.795242000	6.281605000	-3.427268000
6	0.568712000	6.546748000	-2.665352000
6	-0.221198000	7.759322000	-3.171459000
8	-1.451605000	7.844733000	-2.910824000
6	0.835460000	6.677978000	-1.154598000
8	0.621667000	5.366994000	-0.541054000
1	2.670599000	6.187221000	-2.907094000
1	-0.121482000	5.707182000	-2.797554000
1	1.858752000	7.020975000	-0.971917000
1	0.125089000	7.399883000	-0.733777000
1	0.775354000	5.405203000	0.435251000
7	0.445482000	8.696985000	-3.886239000
6	-0.246336000	9.827464000	-4.499777000
6	-1.258269000	9.464519000	-5.597768000
8	-2.073989000	10.337607000	-5.971376000
1	1.428371000	8.547629000	-4.068952000
1	-0.813601000	10.377916000	-3.744380000
7	-1.209661000	8.199028000	-6.099874000
6	-2.210738000	7.715897000	-7.042001000
6	-3.108734000	6.610270000	-6.484466000
8	-3.753295000	5.873221000	-7.273352000
1	-0.496511000	7.534107000	-5.799331000
1	-2.833294000	8.569475000	-7.330305000
7	-3.171453000	6.494667000	-5.128807000
6	-3.980430000	5.468871000	-4.475774000
6	-3.140246000	4.434037000	-3.699519000
6	-2.134502000	3.630819000	-4.497899000
6	-2.326448000	3.313765000	-5.870512000
6	-0.967670000	3.145956000	-3.842765000
6	-1.415803000	2.525949000	-6.546350000
6	-0.039923000	2.364409000	-4.500172000
6	-0.247652000	2.001448000	-5.882157000
8	0.577488000	1.220441000	-6.510492000
1	-2.628654000	7.118788000	-4.538271000
1	-4.584157000	4.996143000	-5.254340000
1	-3.842317000	3.734326000	-3.214345000
1	-2.614003000	4.949752000	-2.885245000
1	-3.173205000	3.726275000	-6.407817000
1	-0.799724000	3.413936000	-2.802865000
1	-1.550584000	2.279589000	-7.593704000
1	0.874606000	2.040382000	-4.014313000
1	-3.510637000	-3.582399000	-3.088151000
6	-4.877350000	4.238427000	2.658290000
6	-3.428489000	4.593335000	2.436223000
8	-2.497043000	3.756962000	2.604654000
6	-5.390108000	3.135117000	1.673866000
6	-6.100050000	1.984181000	2.410992000
6	-5.075601000	1.112427000	3.145866000
8	-4.171213000	0.508189000	2.505132000
8	-5.222818000	1.127890000	4.456346000
1	-4.948313000	3.849787000	3.682589000
1	-6.080704000	3.579577000	0.949679000
1	-4.555216000	2.710978000	1.107241000
1	-6.615910000	1.325252000	1.707074000
1	-6.847647000	2.361487000	3.114327000
7	-3.117058000	5.878224000	2.075610000
6	-1.752247000	6.222371000	1.662259000
6	-0.689935000	5.887795000	2.708372000
8	0.447800000	5.474698000	2.346101000
1	-3.863225000	6.522479000	1.855065000
1	-1.463566000	5.689439000	0.750434000
7	-0.992409000	6.100113000	4.014031000
6	-0.035171000	5.807855000	5.085556000
6	0.141372000	4.310164000	5.396821000
8	1.251316000	3.890983000	5.809226000
1	-1.911905000	6.450729000	4.244502000
1	0.950580000	6.188514000	4.812754000
7	-0.951691000	3.529851000	5.223512000
6	-0.945568000	2.058675000	5.391619000
6	0.029031000	1.451706000	4.359461000
8	0.980684000	0.706030000	4.709000000
6	-2.397569000	1.573179000	5.217167000
6	-2.745573000	0.253399000	5.884765000
8	-2.015982000	-0.254111000	6.789184000
8	-3.866457000	-0.308591000	5.495220000
1	-1.737355000	3.911079000	4.711239000
1	-0.564000000	1.796147000	6.379713000
1	-2.657216000	1.483760000	4.160278000
1	-3.078701000	2.318615000	5.648244000
7	-0.164242000	1.845383000	3.073286000
6	0.716246000	1.390840000	1.992330000
6	2.155737000	1.884440000	2.213560000
8	3.143231000	1.182859000	1.860948000
6	0.176079000	1.844871000	0.613339000
6	-1.206411000	1.276332000	0.192280000
6	-1.266097000	-0.237148000	0.138577000
8	-0.926697000	-0.879580000	1.186321000
8	-1.676996000	-0.808331000	-0.965355000

1	-0.980301000	2.401189000	2.833622000
1	0.764680000	0.301350000	2.009408000
1	0.922878000	1.557496000	-0.134260000
1	0.112625000	2.940051000	0.585917000
1	-1.983917000	1.598190000	0.897376000
1	-1.465067000	1.676838000	-0.791487000
7	2.310432000	3.107641000	2.788885000
6	3.616075000	3.649789000	3.150333000
6	4.412942000	2.783700000	4.126385000
8	5.656101000	2.655113000	3.963703000
1	1.499246000	3.693320000	2.943147000
1	4.267721000	3.757966000	2.279577000
7	3.744663000	2.191099000	5.144353000
6	4.442853000	1.263721000	6.035405000
6	4.678397000	-0.121691000	5.411074000
8	5.833190000	-0.644152000	5.399858000
1	2.760418000	2.392850000	5.297985000
1	5.426083000	1.663050000	6.288601000
7	3.592787000	-0.747497000	4.903351000
6	3.714812000	-2.075343000	4.288317000
6	2.554205000	-2.380689000	3.338530000
6	1.242943000	-2.612305000	4.023445000
7	0.070794000	-2.824396000	3.292681000
6	0.918328000	-2.617984000	5.366536000
6	-0.929077000	-2.935699000	4.180114000
7	-0.449684000	-2.822892000	5.439263000
1	2.692579000	-0.266134000	4.849990000
1	3.786362000	-2.842510000	5.073462000
1	2.809577000	-3.266369000	2.739305000
1	2.456355000	-1.555192000	2.622601000
1	1.511007000	-2.424655000	6.243614000
1	-1.969959000	-3.073076000	3.943998000
6	0.036516000	-1.620214000	-4.768612000
6	1.127801000	-2.199182000	-5.642514000
8	1.328565000	-3.461425000	-5.694136000
6	-1.323247000	-1.534925000	-5.519409000
6	-1.918431000	-2.882397000	-5.958144000
6	-2.569019000	-3.739431000	-4.888585000
8	-2.941302000	-3.048507000	-3.761287000
8	-2.811718000	-4.940366000	-5.039602000
1	-0.064120000	-2.249075000	-3.879633000
1	-2.033694000	-1.022450000	-4.864146000
1	-1.183576000	-0.904750000	-6.405429000
1	-1.178099000	-3.509501000	-6.465497000
1	-2.709654000	-2.689647000	-6.697956000
7	1.846387000	-1.348186000	-6.402176000
6	2.740896000	-1.813544000	-7.472618000
6	3.773185000	-2.874390000	-7.076061000
8	4.018368000	-3.822349000	-7.854776000
1	1.586518000	-0.356539000	-6.385465000
1	2.164951000	-2.263129000	-8.286982000
7	4.413200000	-2.705702000	-5.881752000
6	5.397294000	-3.662514000	-5.395664000
6	4.897320000	-4.523523000	-4.234506000
8	5.710934000	-4.952478000	-3.360554000
1	4.342927000	-1.843180000	-5.351261000
1	5.663678000	-4.322886000	-6.229095000
7	3.585241000	-4.833189000	-4.230883000
6	2.987039000	-5.738785000	-3.239350000
6	1.546182000	-6.116607000	-3.610000000
8	0.704404000	-4.920154000	-3.603044000
6	0.948950000	-7.114326000	-2.622010000
1	2.979213000	-4.398864000	-4.925779000
1	2.974915000	-5.266550000	-2.249625000
1	1.525577000	-6.538309000	-4.624435000
1	0.787666000	-4.398628000	-4.453713000
1	-0.089805000	-7.323176000	-2.891014000
1	1.509986000	-8.055348000	-2.631860000
1	0.964432000	-6.711793000	-1.602986000
6	-5.323200000	-6.496065000	3.676563000
6	-6.484177000	-5.777834000	4.348941000
8	-7.593783000	-5.651320000	3.757081000
6	-5.808205000	-7.802570000	3.014301000
6	-4.736899000	-8.496052000	2.133052000
6	-4.193887000	-7.546237000	1.081490000
8	-3.100000000	-6.934509000	1.251710000
7	-4.961113000	-7.300000000	-0.000664000
1	-4.910851000	-5.823356000	2.913014000
1	-6.695555000	-7.566752000	2.416724000
1	-6.132566000	-8.509215000	3.788967000
1	-5.176941000	-9.383615000	1.661068000
1	-3.891811000	-8.828039000	2.743717000
1	-4.668678000	-6.540330000	-0.625389000
1	-5.835622000	-7.776717000	-0.152709000
7	-6.266481000	-5.286099000	5.596960000
6	-7.352304000	-4.686703000	6.372406000
6	-7.998140000	-3.430575000	5.777987000
8	-9.174586000	-3.135705000	6.093104000
1	-5.310566000	-5.239713000	5.956583000
1	-8.168223000	-5.404214000	6.495933000
7	-7.221547000	-2.683876000	4.947194000
6	-7.735606000	-1.497797000	4.281488000
6	-7.872181000	-1.637596000	2.764131000
8	-7.948177000	-0.599177000	2.052081000
1	-6.230933000	-2.903094000	4.805360000
1	-8.727769000	-1.289592000	4.697974000
7	-7.951439000	-2.900041000	2.271172000
6	-8.176678000	-3.184521000	0.854341000
6	-6.989123000	-3.842702000	0.156103000
8	-5.891711000	-2.899522000	0.024131000
1	-7.912069000	-3.689220000	2.914130000
1	-8.412655000	-2.239794000	0.359929000
1	-6.659973000	-4.728771000	0.716038000
1	-7.304890000	-4.166770000	-0.844421000
1	-5.220550000	-3.178506000	-0.687229000
6	-8.069690000	3.943976000	-3.312949000
6	-7.532742000	2.675964000	-2.682463000
6	-8.109583000	2.150349000	-1.512742000
6	-6.467721000	1.972361000	-3.273958000
6	-7.646935000	0.951958000	-0.952867000
6	-5.998052000	0.776138000	-2.716969000
6	-6.586180000	0.260767000	-1.554779000
1	-8.816578000	3.719259000	-4.086715000
1	-8.556597000	4.586019000	-2.570559000
1	-8.937462000	2.678440000	-1.045119000
1	-6.017196000	2.355944000	-4.186860000
1	-8.101181000	0.548633000	-0.052317000
1	-5.179163000	0.243156000	-3.192316000
1	-6.238120000	-0.671174000	-1.119634000
26	-0.383353000	-2.957093000	1.246087000
26	-2.159540000	-2.718811000	-1.264615000
8	0.271900000	-4.963266000	0.795341000
1	1.248452000	-4.938068000	0.637163000
1	-0.094688000	-5.612990000	1.523900000
8	-4.401388000	-2.718631000	4.384583000
1	-4.235492000	-1.846721000	4.834204000
1	-4.388014000	-2.588762000	3.371517000
8	-4.303284000	-2.119735000	1.885719000
1	-5.041531000	-2.374073000	1.227492000
1	-4.176985000	-1.138441000	1.965205000
8	-2.236769000	-3.387866000	0.699633000
1	-3.161559000	-4.696366000	-0.081491000
1	-2.963390000	-2.926592000	1.214377000
8	-0.505479000	-4.179081000	8.905580000
1	-0.143232000	-4.800438000	8.187670000
1	-0.484162000	-4.567456000	9.793121000
8	-3.969737000	-3.646554000	-1.566570000
8	-3.742000000	-4.983228000	-0.858094000
8	-0.676566000	-4.079040000	-1.577213000
1	-0.194645000	-4.413229000	-2.398236000
8	-0.831654000	-6.416481000	2.532101000
1	-1.677273000	-6.720364000	2.107333000
1	-0.999314000	-6.150849000	3.469121000
8	-2.185900000	-2.875238000	7.533747000
1	-1.599720000	-3.261879000	8.269102000
1	-2.287467000	-1.886405000	7.500188000
8	-1.652691000	-6.149275000	5.212384000
1	-1.946557000	-7.074818000	5.296384000
1	-2.426887000	-5.481966000	5.474894000
8	0.303317000	-5.584621000	6.896339000

1	-0.423585000	-5.846341000	6.245744000
1	1.044285000	-5.179867000	6.413484000
8	-3.442750000	-4.524426000	5.940232000
1	-3.821753000	-3.887055000	5.236704000
1	-3.074661000	-3.949930000	6.690826000
1	3.848804000	1.156141000	6.947659000
1	3.451153000	4.639156000	3.586751000
1	-0.364091000	6.324993000	5.991945000
1	-1.724020000	7.296505000	1.450462000
1	-5.512456000	5.129903000	2.607848000
1	-4.671299000	5.945658000	-3.769244000
1	-1.741659000	7.317044000	-7.945804000
1	0.501047000	10.505607000	-4.920652000
1	6.928511000	4.585846000	-3.627634000
1	3.721863000	4.013880000	0.361529000
1	7.851773000	-1.449165000	-2.823353000
1	9.723349000	2.125772000	-0.130724000
1	6.267568000	0.912180000	3.227146000
1	9.092302000	-2.613083000	3.640045000
1	3.259015000	-0.932674000	-7.865320000
1	6.288099000	-3.135061000	-5.054060000
1	-6.960104000	-4.444518000	7.365438000
1	-7.095380000	-0.633686000	4.478881000
1	-4.515631000	-6.692469000	4.389887000
1	-9.043366000	-3.852287000	0.762624000
1	8.301852000	-4.708222000	-0.044780000
1	-7.274989000	4.525268000	-3.794874000
1	0.312332000	-0.614708000	-4.438010000
1	3.605374000	-6.642122000	-3.173760000
1	-0.432361000	-4.600627000	-0.761380000
1	4.662129000	-2.101868000	3.739567000

^{7}TS3$_{B}$:

6	8.615153000	-2.750750000	2.475810000
6	7.841875000	-1.594265000	1.858935000
8	7.765403000	-1.501334000	0.598468000
6	7.771488000	-4.063472000	2.582101000
6	6.849549000	-4.314932000	1.430062000
7	7.240391000	-4.551092000	0.103818000
6	5.480053000	-4.284647000	1.398639000
6	6.150652000	-4.638299000	-0.699924000
7	5.077033000	-4.482003000	0.084460000
1	9.481494000	-2.921333000	1.828916000
1	8.462311000	-4.903731000	2.727006000
1	7.151128000	-4.016118000	3.483105000
1	4.771425000	-4.128683000	2.191684000
1	6.122703000	-4.777035000	-1.783281000
1	4.053213000	-4.361538000	-0.212495000
7	7.255165000	-0.703659000	2.696799000
6	6.327575000	0.340306000	2.243513000
6	6.787088000	1.175081000	1.077231000
8	5.943519000	1.621781000	0.248454000
1	7.205094000	-0.873729000	3.700095000
1	5.355772000	-0.074586000	1.961069000
7	8.099057000	1.468598000	0.916424000
6	8.527215000	2.259656000	-0.238918000
6	8.011292000	1.711511000	-1.576962000
8	7.738969000	2.503763000	-2.513941000
1	8.772976000	1.145469000	1.594149000
1	8.161460000	3.287323000	-0.169806000
7	7.898710000	0.359952000	-1.695949000
6	7.435916000	-0.245451000	-2.939572000
6	5.921647000	-0.234644000	-3.180340000
8	5.483795000	-0.680544000	-4.273043000
1	8.014790000	-0.242983000	-0.880993000
1	7.881843000	0.281273000	-3.787950000
7	5.133337000	0.256435000	-2.196607000
6	3.684708000	0.461293000	-2.376091000
6	3.383834000	1.965817000	-2.274694000
8	2.873683000	2.602327000	-3.239960000
6	2.833709000	-0.366018000	-1.387540000
6	3.060602000	-1.880431000	-1.503600000
6	2.231909000	-2.681346000	-0.506985000
8	1.205772000	-2.126486000	0.011578000
8	2.591304000	-3.906455000	-0.231467000
1	5.559964000	0.601089000	-1.338025000
1	3.458154000	0.176570000	-3.405212000
1	1.776768000	-0.150626000	-1.580563000
1	3.027297000	-0.042099000	-0.358646000
1	4.119934000	-2.126952000	-1.367128000
1	2.790777000	-2.241381000	-2.507419000
7	3.721653000	2.569684000	-1.109575000
6	3.471970000	3.983536000	-0.871674000
6	4.296340000	4.952610000	-1.704767000
8	3.915613000	6.158837000	-1.783417000
1	4.160452000	2.046122000	-0.354537000
1	2.424711000	4.235800000	-1.074884000
7	5.363893000	4.494492000	-2.391739000
6	6.032352000	5.338822000	-3.380293000
6	5.094166000	5.850378000	-4.481142000
8	5.407022000	6.872252000	-5.133393000
1	5.729100000	3.560497000	-2.243609000
1	6.462049000	6.230712000	-2.916294000
7	3.937017000	5.151938000	-4.691506000
6	2.929790000	5.613912000	-5.644595000
6	1.637451000	6.070280000	-4.944091000
8	0.558489000	6.164465000	-5.592650000
6	2.634753000	4.560652000	-6.724098000
1	3.744812000	4.284698000	-4.193027000
1	3.363531000	6.505001000	-6.118768000
1	1.823224000	4.901276000	-7.370664000
1	3.532944000	4.396694000	-7.326316000
1	2.338624000	3.607161000	-6.275625000
7	1.707016000	6.433834000	-3.630009000
6	0.480012000	6.702581000	-2.870397000
6	-0.301881000	7.923156000	-3.369465000
8	-1.528319000	8.021829000	-3.093868000
6	0.740347000	6.818471000	-1.357707000
8	0.514917000	5.503358000	-0.755805000
1	2.581885000	6.341274000	-3.108932000
1	-0.214425000	5.868682000	-3.014513000
1	1.765130000	7.152429000	-1.166980000
1	0.032714000	7.541250000	-0.934075000
1	0.660260000	5.534902000	0.222071000
7	0.364048000	8.848637000	-4.099961000
6	-0.326690000	9.976615000	-4.719088000
6	-1.352452000	9.605471000	-5.801411000
8	-2.169487000	10.477114000	-6.175487000
1	1.343760000	8.689894000	-4.291473000
1	-0.882266000	10.539842000	-3.964478000
7	-1.313790000	8.334238000	-6.290032000
6	-2.326117000	7.845264000	-7.216882000
6	-3.232000000	6.758522000	-6.635447000
8	-3.889488000	6.015699000	-7.407921000
1	-0.597887000	7.670844000	-5.992451000
1	-2.942354000	8.699858000	-7.515557000
7	-3.287571000	6.665241000	-5.277456000
6	-4.105613000	5.659154000	-4.605227000
6	-3.274373000	4.632822000	-3.807927000
6	-2.262427000	3.820019000	-4.588716000
6	-2.448247000	3.480226000	-5.956612000
6	-1.094847000	3.353589000	-3.922074000
6	-1.528106000	2.691803000	-6.618803000
6	-0.157659000	2.571778000	-4.566135000
6	-0.356206000	2.189784000	-5.944044000
8	0.482184000	1.413252000	-6.559794000
1	-2.733734000	7.291886000	-4.699961000
1	-4.711111000	5.176132000	-5.376109000
1	-3.981831000	3.940326000	-3.320268000
1	-2.753687000	5.157973000	-2.996147000
1	-3.297930000	3.876003000	-6.501825000
1	-0.931828000	3.638969000	-2.885897000
1	-1.657517000	2.428921000	-7.662789000
1	0.758766000	2.261613000	-4.075150000
1	-3.768180000	-3.466118000	-2.861232000
6	-4.989889000	4.359304000	2.438580000
6	-3.542828000	4.719754000	2.214568000
8	-2.608742000	3.886206000	2.383714000
6	-5.499434000	3.255600000	1.453229000
6	-6.218298000	2.108250000	2.187866000

6	-5.201340000	1.232332000	2.927957000
8	-4.312932000	0.602488000	2.291055000
8	-5.335055000	1.275891000	4.240032000
1	-5.057221000	3.968029000	3.462118000
1	-6.184127000	3.700175000	0.723507000
1	-4.661995000	2.828053000	0.892757000
1	-6.733361000	1.452411000	1.481216000
1	-6.968178000	2.488972000	2.886651000
7	-3.235194000	6.004538000	1.851294000
6	-1.870951000	6.352111000	1.438124000
6	-0.808950000	6.022574000	2.486381000
8	0.328207000	5.604772000	2.127896000
1	-3.983111000	6.646727000	1.630604000
1	-1.579919000	5.818452000	0.527537000
7	-1.111478000	6.244449000	3.790558000
6	-0.154283000	5.959007000	4.864012000
6	0.019663000	4.463394000	5.186046000
8	1.127225000	4.045121000	5.605194000
1	-2.029862000	6.599501000	4.018652000
1	0.831918000	6.336316000	4.588163000
7	-1.074213000	3.683748000	5.013778000
6	-1.072428000	2.214161000	5.192214000
6	-0.099324000	1.595292000	4.165518000
8	0.849394000	0.849330000	4.522980000
6	-2.526318000	1.733635000	5.021438000
6	-2.870884000	0.407432000	5.678083000
8	-2.144061000	-0.096565000	6.586807000
8	-3.983575000	-0.161957000	5.277151000
1	-1.857460000	4.063674000	4.497081000
1	-0.692353000	1.957366000	6.182338000
1	-2.791672000	1.658411000	3.965281000
1	-3.202199000	2.476243000	5.465719000
7	-0.289036000	1.979320000	2.876140000
6	0.594808000	1.519251000	1.799945000
6	2.036733000	2.002118000	2.033090000
8	3.021857000	1.290262000	1.696123000
6	0.068539000	1.984566000	0.419269000
6	-1.314180000	1.427627000	-0.017333000
6	-1.378687000	-0.084753000	-0.088239000
8	-1.076703000	-0.738638000	0.963230000
8	-1.751718000	-0.643057000	-1.212311000
1	-1.102020000	2.537099000	2.630703000
1	0.635111000	0.429266000	1.813713000
1	0.819379000	1.698325000	-0.324600000
1	0.011239000	3.080116000	0.399332000
1	-2.093742000	1.745581000	0.686814000
1	-1.562772000	1.840146000	-0.998599000
7	2.194558000	3.228255000	2.601846000
6	3.500377000	3.764995000	2.970719000
6	4.288434000	2.899139000	3.954035000
8	5.532988000	2.771098000	3.801569000
1	1.385621000	3.819624000	2.745933000
1	4.158057000	3.867656000	2.103875000
7	3.612305000	2.306603000	4.966657000
6	4.302121000	1.375409000	5.860298000
6	4.538672000	-0.008389000	5.232532000
8	5.691236000	-0.535528000	5.229467000
1	2.626691000	2.507222000	5.111519000
1	5.284273000	1.771926000	6.121848000
7	3.455192000	-0.626578000	4.711087000
6	3.576602000	-1.949603000	4.085858000
6	2.419806000	-2.242903000	3.127235000
6	1.103742000	-2.466829000	3.805662000
7	-0.070215000	-2.665911000	3.072947000
6	0.777684000	-2.474323000	5.148232000
6	-1.071560000	-2.771367000	3.959713000
7	-0.591959000	-2.667296000	5.219403000
1	2.557949000	-0.140494000	4.652998000
1	3.641172000	-2.723454000	4.865085000
1	2.672433000	-3.127822000	2.525952000
1	2.332319000	-1.413333000	2.414739000
1	1.370631000	-2.288518000	6.026732000
1	-2.113841000	-2.898038000	3.723436000
6	0.030021000	-1.448627000	-4.751549000
6	1.061109000	-2.016530000	-5.702058000

8	1.249349000	-3.278006000	-5.796473000
6	-1.372693000	-1.320483000	-5.411772000
6	-2.011576000	-2.640110000	-5.867851000
6	-2.637967000	-3.527715000	-4.798599000
8	-2.951187000	-2.918807000	-3.623470000
8	-2.924215000	-4.710555000	-5.030701000
1	-0.026574000	-2.103906000	-3.877244000
1	-2.033948000	-0.831922000	-4.689180000
1	-1.280523000	-0.652230000	-6.275646000
1	-1.309005000	-3.260379000	-6.434318000
1	-2.832282000	-2.405456000	-6.562173000
7	1.749976000	-1.155678000	-6.478976000
6	2.596174000	-1.615475000	-7.589722000
6	3.653999000	-2.666595000	-7.235042000
8	3.891848000	-3.601435000	-8.031556000
1	1.494374000	-0.163808000	-6.441999000
1	1.987317000	-2.073503000	-8.374843000
7	4.319379000	-2.505563000	-6.053404000
6	5.318721000	-3.460388000	-5.595239000
6	4.845560000	-4.330288000	-4.429105000
8	5.676125000	-4.750958000	-3.566729000
1	4.250621000	-1.650303000	-5.511355000
1	5.570163000	-4.113898000	-6.438689000
7	3.537287000	-4.655869000	-4.408776000
6	2.962974000	-5.575453000	-3.416252000
6	1.530500000	-5.987742000	-3.784216000
8	0.657846000	-4.816392000	-3.760462000
6	0.964488000	-7.008436000	-2.801075000
1	2.915603000	-4.222401000	-5.090347000
1	2.938338000	-5.104162000	-2.426088000
1	1.517037000	-6.402051000	-4.801849000
1	0.729283000	-4.269047000	-4.596381000
1	-0.069508000	-7.242308000	-3.067771000
1	1.550031000	-7.934286000	-2.818703000
1	0.972913000	-6.610833000	-1.779852000
6	-5.435037000	-6.314667000	3.504946000
6	-6.592896000	-5.609047000	4.195211000
8	-7.709225000	-5.483710000	3.615214000
6	-5.913731000	-7.629877000	2.855475000
6	-4.841851000	-8.318096000	1.971296000
6	-4.313292000	-7.371676000	0.909050000
8	-3.215079000	-6.762662000	1.057825000
7	-5.097458000	-7.129198000	-0.162119000
1	-5.045677000	-5.639354000	2.731645000
1	-6.808166000	-7.407038000	2.263446000
1	-6.224875000	-8.334402000	3.637451000
1	-5.276144000	-9.212849000	1.507516000
1	-3.989841000	-8.638612000	2.578433000
1	-4.808638000	-6.380456000	-0.799795000
1	-5.971499000	-7.610163000	-0.302554000
7	-6.364725000	-5.127926000	5.445271000
6	-7.443221000	-4.538842000	6.237933000
6	-8.086818000	-3.268625000	5.671946000
8	-9.252258000	-2.963810000	6.017108000
1	-5.404421000	-5.078588000	5.793809000
1	-8.261495000	-5.255275000	6.351956000
7	-7.322518000	-2.523221000	4.828501000
6	-7.842855000	-1.330368000	4.179478000
6	-8.030627000	-1.471578000	2.667651000
8	-8.119606000	-0.434975000	1.954626000
1	-6.339957000	-2.754154000	4.656079000
1	-8.818346000	-1.109889000	4.627595000
7	-8.137245000	-2.735367000	2.182668000
6	-8.406665000	-3.023543000	0.775359000
6	-7.252598000	-3.716972000	0.056527000
8	-6.133722000	-2.801049000	-0.099256000
1	-8.080025000	-3.523780000	2.825368000
1	-8.630510000	-2.076631000	0.279553000
1	-6.935967000	-4.607311000	0.616746000
1	-7.595772000	-4.036424000	-0.936311000
1	-5.501698000	-3.077498000	-0.833397000
6	-8.207438000	4.109540000	-3.425435000
6	-7.688607000	2.830052000	-2.803096000
6	-8.279392000	2.300160000	-1.642191000
6	-6.627116000	2.120430000	-3.393666000

6	-7.833747000	1.091653000	-1.090333000
6	-6.174868000	0.913762000	-2.845064000
6	-6.776898000	0.394285000	-1.691945000
1	-8.956633000	3.900051000	-4.201190000
1	-8.686220000	4.753294000	-2.679235000
1	-9.104296000	2.833183000	-1.175028000
1	-6.165022000	2.507960000	-4.298979000
1	-8.296663000	0.687650000	-0.194451000
1	-5.358297000	0.376769000	-3.319757000
1	-6.441199000	-0.544705000	-1.262565000
26	-0.520926000	-2.808141000	1.024234000
26	-2.192703000	-2.550111000	-1.576809000
8	0.169626000	-4.812394000	0.636855000
1	1.145996000	-4.778982000	0.479897000
1	-0.198314000	-5.458120000	1.367430000
8	-4.508792000	-2.576919000	4.176687000
1	-4.343455000	-1.697809000	4.613141000
1	-4.493007000	-2.465653000	3.162677000
8	-4.415106000	-2.020712000	1.658210000
1	-5.194148000	-2.275076000	1.054656000
1	-4.298890000	-1.037606000	1.737024000
8	-2.351254000	-3.246452000	0.388114000
1	-3.239920000	-4.506712000	-0.353368000
1	-3.083375000	-2.791408000	0.901180000
8	-0.580794000	-3.984808000	8.692210000
1	-0.223899000	-4.614536000	7.979059000
1	-0.535764000	-4.355131000	9.586539000
8	-4.222533000	-3.509611000	-1.743684000
8	-3.849202000	-4.837784000	-1.101825000
8	-0.740333000	-3.970039000	-1.755594000
1	-0.257197000	-4.325961000	-2.567029000
8	-0.955561000	-6.255796000	2.364714000
1	-1.797898000	-6.554054000	1.929711000
1	-1.126878000	-6.003742000	3.305013000
8	-2.299731000	-2.710043000	7.340021000
1	-1.695812000	-3.087860000	8.065805000
1	-2.402901000	-1.721237000	7.299771000
8	-1.769209000	-6.000737000	5.042843000
1	-2.062249000	-6.924872000	5.143233000
1	-2.540082000	-5.329027000	5.303810000
8	0.210038000	-5.415272000	6.693837000
1	-0.525372000	-5.685728000	6.057050000
1	0.942971000	-5.014660000	6.195460000
8	-3.553385000	-4.364994000	5.759943000
1	-3.928801000	-3.734465000	5.048995000
1	-3.185804000	-3.784747000	6.507543000
1	3.701267000	1.266339000	6.767893000
1	3.337310000	4.756239000	3.403553000
1	-0.482246000	6.483244000	5.766649000
1	-1.845969000	7.425853000	1.224035000
1	-5.628975000	5.248050000	2.390706000
1	-4.794506000	6.154589000	-3.909506000
1	-1.866719000	7.426527000	-8.116591000
1	0.420449000	10.644634000	-5.156273000
1	6.844062000	4.752573000	-3.818427000
1	3.657467000	4.187807000	0.184266000
1	7.771202000	-1.285776000	-2.973598000
1	9.621179000	2.282219000	-0.254790000
1	6.157494000	1.025772000	3.081703000
1	8.990348000	-2.498768000	3.473165000
1	3.088216000	-0.731920000	-8.008824000
1	6.214074000	-2.931383000	-5.268468000
1	-7.045332000	-4.317752000	7.233533000
1	-7.185681000	-0.474540000	4.355335000
1	-4.612330000	-6.498382000	4.203999000
1	-9.291713000	-3.669945000	0.708626000
1	8.190705000	-4.562006000	-0.231978000
1	-7.404436000	4.682850000	-3.903065000
1	0.342099000	-0.457202000	-4.409015000
1	3.603911000	-6.462871000	-3.352266000
1	-0.513425000	-4.478075000	-0.927477000
1	4.526927000	-1.974962000	3.542199000

[7]Pr$_B$:

6	8.624518000	-2.663665000	2.301958000
6	7.856084000	-1.513370000	1.666151000
8	7.770379000	-1.449322000	0.403406000
6	7.776220000	-3.973562000	2.417148000
6	6.829219000	-4.200945000	1.280898000
7	7.194469000	-4.363567000	-0.062865000
6	5.459019000	-4.186431000	1.280142000
6	6.090961000	-4.414469000	-0.848136000
7	5.031219000	-4.315828000	-0.035112000
1	9.494639000	-2.843861000	1.662589000
1	8.464096000	-4.819928000	2.537950000
1	7.175471000	-3.929927000	3.331331000
1	4.765557000	-4.079980000	2.094546000
1	6.051450000	-4.473152000	-1.938690000
1	4.005726000	-4.182681000	-0.307934000
7	7.288478000	-0.601918000	2.492373000
6	6.363349000	0.444672000	2.039261000
6	6.793496000	1.243098000	0.836454000
8	5.921908000	1.710751000	0.047886000
1	7.239370000	-0.762526000	3.499113000
1	5.375489000	0.038306000	1.803557000
7	8.103986000	1.484843000	0.600692000
6	8.503681000	2.227999000	-0.595800000
6	7.890365000	1.684413000	-1.895014000
8	7.605488000	2.477875000	-2.826862000
1	8.801281000	1.129603000	1.237160000
1	8.193796000	3.274171000	-0.530631000
7	7.704443000	0.338418000	-1.989645000
6	7.148435000	-0.253934000	-3.203633000
6	5.633950000	-0.106059000	-3.399503000
8	5.118393000	-0.491708000	-4.481349000
1	7.851330000	-0.260535000	-1.176603000
1	7.616758000	0.200290000	-4.081101000
7	4.923779000	0.414159000	-2.372036000
6	3.475345000	0.666202000	-2.463246000
6	3.218451000	2.171594000	-2.288849000
8	2.685146000	2.864173000	-3.201881000
6	2.670163000	-0.172478000	-1.447498000
6	2.889370000	-1.684637000	-1.594744000
6	2.172207000	-2.483019000	-0.518632000
8	1.222352000	-1.916824000	0.119903000
8	2.541001000	-3.715948000	-0.294222000
1	5.410611000	0.719225000	-1.531101000
1	3.184169000	0.417730000	-3.485334000
1	1.605391000	0.050237000	-1.578806000
1	2.922920000	0.131798000	-0.425085000
1	3.957437000	-1.930861000	-1.571734000
1	2.512883000	-2.046018000	-2.563474000
7	3.628295000	2.706688000	-1.112886000
6	3.459442000	4.114845000	-0.787552000
6	4.286269000	5.083495000	-1.618826000
8	3.955556000	6.306601000	-1.639781000
1	4.092214000	2.135423000	-0.409509000
1	2.416002000	4.426971000	-0.907641000
7	5.306701000	4.605173000	-2.362937000
6	5.975699000	5.450697000	-3.349954000
6	5.018641000	6.044812000	-4.390938000
8	5.346031000	7.080378000	-5.013568000
1	5.620546000	3.646506000	-2.267802000
1	6.466559000	6.304380000	-2.874914000
7	3.826697000	5.401529000	-4.580848000
6	2.796810000	5.948050000	-5.462372000
6	1.523748000	6.315393000	-4.680639000
8	0.405012000	6.373025000	-5.262646000
6	2.479483000	5.010149000	-6.636300000
1	3.617504000	4.522075000	-4.111159000
1	3.217710000	6.884441000	-5.854768000
1	1.665338000	5.418500000	-7.238726000
1	3.370367000	4.894604000	-7.259833000
1	2.174216000	4.023307000	-6.275240000
7	1.650319000	6.644066000	-3.361915000
6	0.455749000	6.843133000	-2.529733000
6	-0.441585000	7.984848000	-3.020426000
8	-1.673339000	7.961846000	-2.760154000
6	0.812773000	7.042647000	-1.042079000
8	0.633586000	5.758426000	-0.366559000

1	2.553242000	6.556133000	-2.889654000
1	-0.182071000	5.954259000	-2.584668000
1	1.845863000	7.388529000	-0.936717000
1	0.132973000	7.785219000	-0.606011000
1	0.838350000	5.821602000	0.598514000
7	0.145822000	8.999086000	-3.703460000
6	-0.634425000	10.102522000	-4.257508000
6	-1.621860000	9.730039000	-5.374119000
8	-2.489988000	10.568826000	-5.706484000
1	1.134234000	8.930786000	-3.903643000
1	-1.238104000	10.566084000	-3.472416000
7	-1.493366000	8.497711000	-5.939070000
6	-2.458571000	8.001012000	-6.911373000
6	-3.250923000	6.782183000	-6.435941000
8	-3.863681000	6.072697000	-7.273933000
1	-0.747971000	7.860236000	-5.658942000
1	-3.154872000	8.816691000	-7.132461000
7	-3.258576000	6.540013000	-5.096435000
6	-3.957760000	5.396495000	-4.517289000
6	-3.011599000	4.323291000	-3.941086000
6	-2.080877000	3.619558000	-4.905666000
6	-2.425691000	3.375356000	-6.263984000
6	-0.840918000	3.118784000	-4.418533000
6	-1.605722000	2.614520000	-7.074340000
6	-0.006650000	2.358002000	-5.209719000
6	-0.382143000	2.040411000	-6.567818000
8	0.336997000	1.253585000	-7.308413000
1	-2.755278000	7.157301000	-4.466027000
1	-4.603203000	4.984982000	-5.296253000
1	-3.643159000	3.555464000	-3.461541000
1	-2.416269000	4.773291000	-3.135748000
1	-3.320922000	3.822541000	-6.681188000
1	-0.544155000	3.343656000	-3.398630000
1	-1.861655000	2.414793000	-8.108892000
1	0.948941000	2.011325000	-4.831305000
1	-3.772072000	-4.722775000	-2.476097000
6	-4.774555000	4.434938000	2.703137000
6	-3.331590000	4.840366000	2.540730000
8	-2.387815000	4.008089000	2.649790000
6	-5.241701000	3.436823000	1.592630000
6	-6.022466000	2.240272000	2.176134000
6	-5.066068000	1.311541000	2.936596000
8	-4.263122000	0.565797000	2.314826000
8	-5.141149000	1.447433000	4.248198000
1	-4.850717000	3.928742000	3.674059000
1	-5.873828000	3.963203000	0.869214000
1	-4.377104000	3.048465000	1.044195000
1	-6.477584000	1.639653000	1.383958000
1	-6.821074000	2.584412000	2.839703000
7	-3.039655000	6.155637000	2.291983000
6	-1.680699000	6.552069000	1.903977000
6	-0.608148000	6.163676000	2.920758000
8	0.532511000	5.791311000	2.526214000
1	-3.796288000	6.801381000	2.115641000
1	-1.386413000	6.087492000	0.956990000
7	-0.906354000	6.284050000	4.239945000
6	0.059917000	5.928337000	5.284560000
6	0.229236000	4.413352000	5.505857000
8	1.344650000	3.956003000	5.856857000
1	-1.826761000	6.612865000	4.497499000
1	1.044689000	6.318689000	5.022713000
7	-0.881285000	3.660045000	5.322091000
6	-0.904394000	2.180727000	5.396354000
6	0.073347000	1.620320000	4.340181000
8	1.004989000	0.833965000	4.649511000
6	-2.364857000	1.743449000	5.164031000
6	-2.770962000	0.400813000	5.747661000
8	-2.084853000	-0.177771000	6.641587000
8	-3.904295000	-0.096337000	5.303642000
1	-1.677473000	4.090725000	4.870564000
1	-0.546906000	1.845354000	6.371588000
1	-2.603481000	1.728028000	4.099519000
1	-3.036716000	2.481116000	5.622539000
7	-0.107325000	2.095750000	3.081191000
6	0.745985000	1.682760000	1.963859000
6	2.193071000	2.154910000	2.164474000
8	3.164865000	1.465247000	1.750930000
6	0.172637000	2.214164000	0.628519000
6	-1.229282000	1.672374000	0.245591000
6	-1.275269000	0.166039000	0.100727000
8	-1.060389000	-0.545902000	1.139719000
8	-1.528383000	-0.326853000	-1.080205000
1	-0.910315000	2.684826000	2.878555000
1	0.785106000	0.592545000	1.929884000
1	0.886046000	1.958651000	-0.161266000
1	0.118760000	3.309480000	0.659824000
1	-1.966572000	1.948651000	1.007453000
1	-1.531739000	2.126928000	-0.701164000
7	2.370388000	3.352611000	2.784992000
6	3.690879000	3.868006000	3.128301000
6	4.501375000	2.941300000	4.035276000
8	5.737974000	2.804944000	3.836409000
1	1.568166000	3.933908000	2.992521000
1	4.315055000	4.013997000	2.242699000
7	3.844447000	2.302324000	5.033231000
6	4.542182000	1.315017000	5.857728000
6	4.708454000	-0.052025000	5.172474000
8	5.854162000	-0.582360000	5.051582000
1	2.866057000	2.508177000	5.216304000
1	5.546885000	1.673160000	6.086535000
7	3.578688000	-0.655889000	4.740399000
6	3.636309000	-1.967793000	4.081661000
6	2.441702000	-2.211252000	3.155140000
6	1.146932000	-2.476428000	3.861316000
7	-0.049354000	-2.635206000	3.151259000
6	0.859688000	-2.586713000	5.206495000
6	-1.021698000	-2.822427000	4.058703000
7	-0.503422000	-2.806861000	5.305812000
1	2.682811000	-0.163956000	4.764575000
1	3.706952000	-2.761641000	4.839585000
1	2.674491000	-3.064636000	2.502164000
1	2.332030000	-1.349331000	2.486240000
1	1.485537000	-2.487901000	6.075695000
1	-2.070764000	-2.939788000	3.848428000
6	-0.231780000	-1.527123000	-5.424505000
6	0.884114000	-2.139411000	-6.238779000
8	1.174701000	-3.383372000	-6.147417000
6	-1.639375000	-1.782280000	-6.048351000
6	-2.247264000	-3.163466000	-5.750569000
6	-2.736410000	-3.351273000	-4.318000000
8	-2.562643000	-2.337789000	-3.495995000
8	-3.274347000	-4.444940000	-3.978053000
1	-0.205841000	-1.941780000	-4.413762000
1	-2.311122000	-1.012095000	-5.657971000
1	-1.578630000	-1.635695000	-7.134200000
1	-1.530818000	-3.968015000	-5.961501000
1	-3.104494000	-3.347952000	-6.410972000
7	1.543987000	-1.333922000	-7.095956000
6	2.460762000	-1.853787000	-8.117648000
6	3.625308000	-2.714753000	-7.621325000
8	4.017144000	-3.678232000	-8.317686000
1	1.251650000	-0.351602000	-7.158828000
1	1.922653000	-2.485901000	-8.830199000
7	4.214498000	-2.365326000	-6.440120000
6	5.308694000	-3.141814000	-5.874892000
6	4.904690000	-3.945402000	-4.640548000
8	5.758745000	-4.202456000	-3.737201000
1	3.982060000	-1.507698000	-5.952482000
1	5.655747000	-3.837168000	-6.647716000
7	3.633931000	-4.395230000	-4.603305000
6	3.131948000	-5.270453000	-3.537253000
6	1.713341000	-5.772394000	-3.836883000
8	0.789589000	-4.641217000	-3.887402000
6	1.217000000	-6.738108000	-2.764448000
1	2.986292000	-4.085462000	-5.326244000
1	3.107479000	-4.734018000	-2.581255000
1	1.699233000	-6.264825000	-4.819332000
1	0.797502000	-4.187473000	-4.783517000
1	0.187982000	-7.036050000	-2.981886000
1	1.842305000	-7.637117000	-2.728944000

1	1.234187000	-6.263288000	-1.777262000
6	-5.833841000	-6.263472000	3.779480000
6	-6.940619000	-5.370054000	4.318901000
8	-8.028680000	-5.217102000	3.694093000
6	-6.394703000	-7.457545000	2.987785000
6	-5.290745000	-8.445878000	2.528227000
6	-4.147873000	-7.724054000	1.841159000
8	-3.069749000	-7.489721000	2.481664000
7	-4.345864000	-7.286678000	0.589178000
1	-5.194668000	-5.652030000	3.126792000
1	-6.957467000	-7.080257000	2.127045000
1	-7.116353000	-8.005971000	3.604555000
1	-5.727185000	-9.197509000	1.858503000
1	-4.870352000	-8.969824000	3.391035000
1	-3.695075000	-6.628061000	0.126777000
1	-5.205878000	-7.492432000	0.103106000
7	-6.677244000	-4.750967000	5.499753000
6	-7.700229000	-3.974341000	6.195488000
6	-8.234711000	-2.748170000	5.452209000
8	-9.384014000	-2.318505000	5.707143000
1	-5.708121000	-4.737583000	5.836308000
1	-8.574974000	-4.597700000	6.401020000
7	-7.392427000	-2.172154000	4.551379000
6	-7.806167000	-1.038191000	3.744342000
6	-7.852481000	-1.332029000	2.245788000
8	-7.751578000	-0.384165000	1.419268000
1	-6.420060000	-2.474959000	4.450360000
1	-8.811437000	-0.751623000	4.075310000
7	-8.056079000	-2.626546000	1.888412000
6	-8.244192000	-3.045133000	0.500011000
6	-7.171292000	-4.003237000	-0.008351000
8	-5.908240000	-3.303654000	-0.182888000
1	-8.142171000	-3.335996000	2.615247000
1	-8.257357000	-2.146038000	-0.119713000
1	-7.048290000	-4.836456000	0.697848000
1	-7.492648000	-4.411195000	-0.975378000
1	-5.255956000	-3.838542000	-0.723265000
6	-7.363470000	3.181725000	-4.611907000
6	-6.885877000	2.056969000	-3.716804000
6	-7.558930000	1.761856000	-2.516468000
6	-5.782676000	1.262780000	-4.074596000
6	-7.154250000	0.696940000	-1.704214000
6	-5.371308000	0.192564000	-3.267345000
6	-6.060007000	-0.094817000	-2.083273000
1	-8.329178000	2.939350000	-5.075287000
1	-7.504293000	4.115063000	-4.052381000
1	-8.414661000	2.366488000	-2.223920000
1	-5.252276000	1.474427000	-5.000217000
1	-7.678441000	0.472209000	-0.779945000
1	-4.522388000	-0.417934000	-3.562328000
1	-5.766064000	-0.936371000	-1.464064000
26	-0.497028000	-2.609718000	1.118152000
26	-1.894388000	-2.190590000	-1.678967000
8	0.159726000	-4.600920000	0.604906000
1	1.137082000	-4.551047000	0.442153000
1	-0.177862000	-5.277502000	1.316213000
8	-4.557608000	-2.375212000	3.970031000
1	-4.327412000	-1.512539000	4.404534000
1	-4.484425000	-2.303365000	2.963612000
8	-4.394571000	-1.946319000	1.390458000
1	-5.134880000	-2.353308000	0.831059000
1	-4.405588000	-0.957543000	1.465032000
8	-2.260179000	-2.970570000	0.191329000
1	-2.672421000	-4.373580000	-0.257900000
1	-3.036694000	-2.547443000	0.682519000
8	-1.418734000	-4.602950000	8.791578000
1	-1.172522000	-5.318803000	8.104634000
1	-1.798337000	-4.987645000	9.596937000
8	-4.155721000	-4.821588000	-1.529019000
8	-2.970392000	-5.268843000	-0.702410000
8	-0.481850000	-3.680505000	-1.835505000
1	0.001831000	-4.048746000	-2.639147000
8	-0.801618000	-6.094486000	2.372570000
1	-1.552940000	-6.695873000	2.132617000
1	-1.000907000	-5.769668000	3.281743000
8	-2.341019000	-2.842701000	7.247253000
1	-2.011155000	-3.384890000	8.047005000
1	-2.398160000	-1.854283000	7.301277000
8	-2.222931000	-6.059833000	4.778041000
1	-2.632596000	-6.763083000	4.224008000
1	-2.905663000	-5.341078000	5.056543000
8	-0.857797000	-6.314591000	6.963756000
1	-1.391556000	-6.307262000	6.096844000
1	0.058179000	-6.594977000	6.810331000
8	-3.845766000	-4.241302000	5.614224000
1	-4.134880000	-3.572410000	4.906703000
1	-3.365436000	-3.732033000	6.346032000
1	3.979948000	1.190040000	6.787778000
1	3.550101000	4.835687000	3.618391000
1	-0.257517000	6.394898000	6.222004000
1	-1.672253000	7.638800000	1.768423000
1	-5.431670000	5.310920000	2.738739000
1	-4.606623000	5.746938000	-3.705773000
1	-1.967368000	7.713989000	-7.845815000
1	0.056661000	10.858240000	-4.640948000
1	6.738929000	4.841003000	-3.840382000
1	3.718387000	4.247733000	0.264986000
1	7.374787000	-1.324706000	-3.211149000
1	9.594988000	2.195912000	-0.670848000
1	6.231394000	1.152475000	2.867424000
1	8.994207000	-2.400235000	3.298083000
1	2.859662000	-0.991918000	-8.662299000
1	6.124555000	-2.481005000	-5.586151000
1	-7.281948000	-3.651338000	7.154407000
1	-7.141774000	-0.185700000	3.902670000
1	-5.198277000	-6.607635000	4.602210000
1	-9.218129000	-3.543931000	0.407208000
1	8.135968000	-4.317600000	-0.419740000
1	-6.654780000	3.379207000	-5.422983000
1	-0.074830000	-0.449871000	-5.340084000
1	3.813471000	-6.123542000	-3.429558000
1	-0.387348000	-4.252733000	-1.027394000
1	4.566361000	-2.001810000	3.504874000

$^{5}Re_{B}$:

6	8.734820000	-3.036110000	3.202034000
6	8.046196000	-1.896132000	2.465229000
8	8.074103000	-1.863206000	1.199654000
6	7.801096000	-4.272303000	3.406620000
6	6.922787000	-4.589627000	2.236563000
7	7.369209000	-4.923525000	0.949933000
6	5.556608000	-4.543164000	2.139127000
6	6.317692000	-5.052545000	0.103814000
7	5.209890000	-4.826729000	0.823262000
1	9.602231000	-3.318992000	2.597896000
1	8.425070000	-5.133418000	3.678228000
1	7.142262000	-4.084590000	4.260768000
1	4.816431000	-4.321257000	2.885801000
1	6.357396000	-5.264876000	-0.968878000
1	4.208685000	-4.702488000	0.463921000
7	7.407699000	-0.962800000	3.213208000
6	6.513681000	0.055347000	2.650010000
6	7.039408000	0.830056000	1.471830000
8	6.245371000	1.216979000	0.567718000
1	7.279307000	-1.087526000	4.216754000
1	5.558142000	-0.373023000	2.334527000
7	8.352954000	1.141484000	1.376324000
6	8.833813000	1.891718000	0.213099000
6	8.411931000	1.273203000	-1.127358000
8	8.181330000	2.011580000	-2.116184000
1	8.989867000	0.867619000	2.109398000
1	8.442365000	2.912153000	0.214296000
7	8.339708000	-0.087048000	-1.186949000
6	7.971504000	-0.757550000	-2.427961000
6	6.482237000	-0.732890000	-2.791449000
8	6.129654000	-1.162035000	-3.922469000
1	8.417385000	-0.647692000	-0.338621000
1	8.495833000	-0.292106000	-3.267049000
7	5.623225000	-0.258858000	-1.861856000
6	4.191436000	-0.054819000	-2.136384000

6	3.870900000	1.449710000	-2.166719000
8	3.309535000	1.972193000	-3.169724000
6	3.278830000	-0.782344000	-1.129140000
6	3.449139000	-2.308447000	-1.126231000
6	2.491662000	-2.983579000	-0.161503000
8	1.437231000	-2.343013000	0.171020000
8	2.756831000	-4.183136000	0.286684000
1	5.992017000	0.088254000	-0.978483000
1	4.016950000	-0.424946000	-3.147681000
1	2.239295000	-0.546559000	-1.380682000
1	3.444487000	-0.387427000	-0.119619000
1	4.479178000	-2.592047000	-0.881758000
1	3.230407000	-2.735304000	-2.115939000
7	4.228859000	2.154045000	-1.064878000
6	3.937764000	3.570150000	-0.886991000
6	4.635040000	4.515009000	-1.853301000
8	4.184574000	5.690632000	-1.985168000
1	4.691406000	1.690932000	-0.284760000
1	2.865825000	3.776532000	-0.996408000
7	5.656762000	4.057239000	-2.611419000
6	6.129970000	4.846059000	-3.748710000
6	5.024014000	5.181018000	-4.764275000
8	5.179578000	6.148316000	-5.544276000
1	6.108359000	3.172552000	-2.405758000
1	6.539798000	5.806513000	-3.425514000
7	3.910673000	4.388961000	-4.758192000
6	2.759823000	4.669241000	-5.613937000
6	1.560444000	5.242217000	-4.834630000
8	0.427791000	5.308514000	-5.386125000
6	2.329925000	3.422031000	-6.404830000
1	3.843584000	3.567370000	-4.155711000
1	3.082709000	5.453464000	-6.310707000
1	1.421791000	3.635382000	-6.972223000
1	3.129946000	3.129089000	-7.091502000
1	2.126040000	2.590662000	-5.720926000
7	1.771187000	5.724641000	-3.577761000
6	0.643417000	6.139509000	-2.735548000
6	-0.103438000	7.370305000	-3.270846000
8	-1.267143000	7.617835000	-2.856504000
6	1.075810000	6.388888000	-1.282411000
8	0.997975000	5.123982000	-0.554793000
1	2.695308000	5.673095000	-3.146301000
1	-0.111662000	5.344760000	-2.726321000
1	2.095677000	6.785340000	-1.246662000
1	0.383429000	7.115648000	-0.841247000
1	1.155504000	5.260539000	0.410763000
7	0.537153000	8.144160000	-4.180567000
6	-0.131807000	9.252254000	-4.853336000
6	-1.238334000	8.854423000	-5.842201000
8	-1.966690000	9.756931000	-6.314377000
1	1.468065000	7.873229000	-4.465645000
1	-0.608952000	9.901883000	-4.114360000
7	-1.371451000	7.531396000	-6.138105000
6	-2.471227000	7.051783000	-6.965307000
6	-3.664533000	6.475566000	-6.195135000
8	-4.607908000	5.949392000	-6.838351000
1	-0.713756000	6.836228000	-5.785686000
1	-2.840631000	7.887468000	-7.566635000
7	-3.636795000	6.578102000	-4.840471000
6	-4.741280000	6.124032000	-3.994573000
6	-4.343428000	5.044483000	-2.973380000
6	-3.992633000	3.660636000	-3.506328000
6	-4.252443000	3.241522000	-4.823508000
6	-3.437980000	2.719530000	-2.619991000
6	-3.971130000	1.932476000	-5.230920000
6	-3.159240000	1.407718000	-3.010241000
6	-3.425419000	1.009319000	-4.329980000
8	-3.161596000	-0.267651000	-4.787501000
1	-2.841269000	7.012301000	-4.384624000
1	-5.529422000	5.770153000	-4.663233000
1	-5.190746000	4.945530000	-2.274932000
1	-3.500579000	5.430037000	-2.378693000
1	-4.662425000	3.932588000	-5.553171000
1	-3.231848000	3.016700000	-1.592960000
1	-4.167326000	1.613827000	-6.248524000
1	-2.742292000	0.690779000	-2.311593000
1	-2.747892000	-0.868690000	-4.111932000
6	-4.693187000	4.455368000	2.329069000
6	-3.214721000	4.722256000	2.204069000
8	-2.346826000	3.845538000	2.480546000
6	-5.192855000	3.358511000	1.329862000
6	-5.947952000	2.217971000	2.034858000
6	-4.980036000	1.297429000	2.782553000
8	-4.055217000	0.696491000	2.165944000
8	-5.196442000	1.257090000	4.082336000
1	-4.863519000	4.102156000	3.354221000
1	-5.857720000	3.815112000	0.589958000
1	-4.350139000	2.925106000	0.782052000
1	-6.468183000	1.589495000	1.306919000
1	-6.707453000	2.602266000	2.720311000
7	-2.809424000	5.968207000	1.806970000
6	-1.406544000	6.218602000	1.460925000
6	-0.423426000	5.893163000	2.582525000
8	0.722082000	5.437223000	2.303230000
1	-3.503251000	6.632497000	1.493392000
1	-1.096962000	5.617025000	0.600160000
7	-0.795984000	6.148119000	3.861445000
6	0.084927000	5.841306000	4.992800000
6	0.195847000	4.341459000	5.324723000
8	1.267471000	3.887525000	5.797137000
1	-1.715359000	6.533221000	4.028357000
1	1.096522000	6.189832000	4.778192000
7	-0.913888000	3.596512000	5.103553000
6	-0.958626000	2.128608000	5.285577000
6	0.040686000	1.480530000	4.303855000
8	0.935631000	0.692301000	4.707273000
6	-2.418295000	1.687678000	5.058949000
6	-2.813891000	0.337083000	5.633399000
8	-2.173181000	-0.198993000	6.588041000
8	-3.885661000	-0.215893000	5.115649000
1	-1.654940000	3.991790000	4.536618000
1	-0.623694000	1.866724000	6.290574000
1	-2.657862000	1.683064000	3.994877000
1	-3.083112000	2.424315000	5.530187000
7	-0.062004000	1.873725000	3.007836000
6	0.856478000	1.365746000	1.982246000
6	2.298284000	1.823322000	2.246758000
8	3.273031000	1.070205000	1.976952000
6	0.392004000	1.777263000	0.566172000
6	-0.975428000	1.203074000	0.111576000
6	-1.007696000	-0.307547000	0.048099000
8	-0.763675000	-0.931292000	1.159137000
8	-1.255945000	-0.896286000	-1.069113000
1	-0.833955000	2.469606000	2.721120000
1	0.874859000	0.277458000	2.043090000
1	1.166619000	1.452568000	-0.137323000
1	0.342399000	2.869913000	0.486440000
1	-1.774184000	1.516472000	0.795936000
1	-1.203224000	1.601128000	-0.879094000
7	2.469277000	3.066373000	2.773312000
6	3.781402000	3.573528000	3.156448000
6	4.496973000	2.717785000	4.202966000
8	5.742187000	2.544042000	4.114163000
1	1.678986000	3.694921000	2.859993000
1	4.466477000	3.611766000	2.304915000
7	3.752996000	2.175104000	5.197125000
6	4.358975000	1.233823000	6.140068000
6	4.554696000	-0.176133000	5.555432000
8	5.685870000	-0.747898000	5.598966000
1	2.773967000	2.428206000	5.302861000
1	5.347724000	1.588773000	6.434682000
7	3.463871000	-0.765934000	5.017253000
6	3.561026000	-2.094862000	4.401211000
6	2.447932000	-2.351960000	3.379424000
6	1.097510000	-2.587797000	3.982429000
7	-0.038373000	-2.798261000	3.192167000
6	0.703832000	-2.602685000	5.306554000
6	-1.080215000	-2.916553000	4.027186000
7	-0.664952000	-2.811671000	5.310070000
1	2.585508000	-0.250001000	4.922576000

1	3.555741000	-2.869556000	5.182688000
1	2.723950000	-3.222517000	2.768986000
1	2.406327000	-1.503159000	2.685621000
1	1.251527000	-2.405935000	6.211594000
1	-2.108676000	-3.044906000	3.741784000
6	1.421875000	-1.190616000	-4.857633000
6	2.301952000	-2.182448000	-5.583355000
8	2.240643000	-3.432176000	-5.369775000
6	0.069180000	-0.924716000	-5.582129000
6	-0.835825000	-2.161586000	-5.761221000
6	-1.142087000	-2.896898000	-4.458126000
8	-1.558885000	-2.099171000	-3.464969000
8	-0.962244000	-4.129628000	-4.352165000
1	1.209441000	-1.589213000	-3.860745000
1	-0.472041000	-0.178489000	-4.994660000
1	0.263227000	-0.479751000	-6.567447000
1	-0.393125000	-2.893816000	-6.442484000
1	-1.783415000	-1.815789000	-6.190985000
7	3.162611000	-1.698163000	-6.521203000
6	3.880167000	-2.573302000	-7.454772000
6	4.856506000	-3.575961000	-6.831534000
8	5.116876000	-4.635421000	-7.445460000
1	3.204760000	-0.699416000	-6.665497000
1	3.172064000	-3.177265000	-8.029519000
7	5.407615000	-3.248064000	-5.630475000
6	6.294008000	-4.155727000	-4.920413000
6	5.624048000	-4.897123000	-3.762559000
8	6.325997000	-5.316114000	-2.791457000
1	5.282017000	-2.332083000	-5.208741000
1	6.662052000	-4.902263000	-5.633745000
7	4.299894000	-5.120378000	-3.862967000
6	3.560679000	-5.942918000	-2.897358000
6	2.061654000	-5.965808000	-3.213610000
8	1.530788000	-4.609583000	-3.065958000
6	1.295936000	-6.885853000	-2.267550000
1	3.779282000	-4.673329000	-4.615172000
1	3.687094000	-5.540249000	-1.886653000
1	1.904540000	-6.289066000	-4.251503000
1	1.527489000	-4.146419000	-3.947094000
1	0.232303000	-6.871323000	-2.520732000
1	1.658724000	-7.917073000	-2.343284000
1	1.412532000	-6.548937000	-1.231624000
6	-5.385702000	-6.054413000	2.895196000
6	-6.608980000	-5.511595000	3.624688000
8	-7.697818000	-5.325905000	3.012130000
6	-5.798868000	-7.043562000	1.788738000
6	-4.655837000	-7.452072000	0.818856000
6	-4.041987000	-6.256509000	0.124736000
8	-3.118733000	-5.598669000	0.733853000
7	-4.503012000	-5.860467000	-1.059615000
1	-4.859012000	-5.198501000	2.453406000
1	-6.627446000	-6.598413000	1.228188000
1	-6.194614000	-7.959396000	2.245160000
1	-5.050600000	-8.160836000	0.081996000
1	-3.856427000	-7.957738000	1.371012000
1	-4.101556000	-5.014536000	-1.540215000
1	-5.216162000	-6.390943000	-1.538547000
7	-6.459015000	-5.209278000	4.941284000
6	-7.586877000	-4.716762000	5.731560000
6	-8.162858000	-3.361550000	5.306582000
8	-9.332749000	-3.060481000	5.636971000
1	-5.527535000	-5.197735000	5.360648000
1	-8.420704000	-5.421702000	5.677841000
7	-7.337464000	-2.544877000	4.595908000
6	-7.792868000	-1.268162000	4.069798000
6	-7.912804000	-1.221953000	2.546414000
8	-7.965850000	-0.101795000	1.972389000
1	-6.358291000	-2.787217000	4.433232000
1	-8.779830000	-1.065565000	4.499893000
7	-7.989590000	-2.409682000	1.890365000
6	-8.178837000	-2.491070000	0.442318000
6	-6.998169000	-3.112584000	-0.300627000
8	-5.824612000	-2.263152000	-0.204286000
1	-7.988892000	-3.277440000	2.422854000
1	-8.351812000	-1.478112000	0.074096000

1	-6.773784000	-4.106774000	0.110803000
1	-7.268390000	-3.227925000	-1.357645000
1	-5.172897000	-2.335971000	-0.978269000
6	-9.932805000	4.425681000	-3.087914000
6	-9.078783000	3.323984000	-2.496376000
6	-9.410742000	2.734950000	-1.264230000
6	-7.945430000	2.847837000	-3.180387000
6	-8.641011000	1.692958000	-0.731122000
6	-7.171186000	1.806903000	-2.655052000
6	-7.518237000	1.225077000	-1.428296000
1	-10.672306000	4.022380000	-3.793683000
1	-10.486267000	4.965069000	-2.311063000
1	-10.281053000	3.094604000	-0.720035000
1	-7.666722000	3.293350000	-4.131850000
1	-8.898525000	1.245417000	0.223449000
1	-6.299542000	1.458058000	-3.198594000
1	-6.923987000	0.413077000	-1.018577000
26	-0.300841000	-2.799561000	1.150405000
26	-1.980913000	-2.676578000	-1.681622000
8	0.213668000	-4.736015000	0.967354000
1	1.205188000	-4.784635000	0.875414000
1	-0.242250000	-5.369873000	1.661820000
8	-4.478768000	-2.576388000	4.022198000
1	-4.307117000	-1.718913000	4.506424000
1	-4.343290000	-2.422136000	3.034301000
8	-4.042795000	-1.873523000	1.520921000
1	-4.884481000	-2.005362000	0.930162000
1	-3.905126000	-0.901794000	1.721431000
8	-2.227202000	-3.182620000	0.331519000
1	-2.548955000	-4.144570000	0.391704000
1	-2.939365000	-2.569338000	0.809694000
8	-0.973954000	-4.148293000	8.783908000
1	-0.575230000	-4.782558000	8.100714000
1	-0.970698000	-4.498619000	9.686978000
8	-3.854519000	-2.351836000	-1.902511000
8	-3.428724000	-3.726563000	-2.354332000
8	-0.537660000	-3.993089000	-1.541945000
1	0.058442000	-4.266752000	-2.300021000
8	-1.192804000	-6.135451000	2.480980000
1	-2.024466000	-6.102925000	1.941859000
1	-1.374899000	-6.092379000	3.457530000
8	-2.523339000	-2.809375000	7.303284000
1	-1.997312000	-3.208825000	8.076957000
1	-2.572758000	-1.815478000	7.252613000
8	-1.996855000	-6.161073000	5.089203000
1	-2.321618000	-7.065582000	5.252365000
1	-2.746084000	-5.436688000	5.301376000
8	-0.067617000	-5.569296000	6.823697000
1	-0.769108000	-5.836685000	6.153312000
1	0.682969000	-5.151303000	6.367302000
8	-3.709207000	-4.421724000	5.646876000
1	-4.008546000	-3.776773000	4.916875000
1	-3.382899000	-3.851054000	6.425015000
1	3.715897000	1.176764000	7.023169000
1	3.644436000	4.589553000	3.538452000
1	-0.284416000	6.376759000	5.872535000
1	-1.308573000	7.274555000	1.188403000
1	-5.268504000	5.379815000	2.208362000
1	-5.140849000	6.987830000	-3.445697000
1	-2.115130000	6.271226000	-7.643116000
1	0.616738000	9.842903000	-5.387853000
1	6.925446000	4.282199000	-4.243369000
1	4.215768000	3.840961000	0.135093000
1	8.286712000	-1.803462000	-2.369335000
1	9.925956000	1.939111000	0.262116000
1	6.299832000	0.784783000	3.439970000
1	9.103171000	-2.715264000	4.182164000
1	4.428563000	-1.936467000	-8.155845000
1	7.142138000	-3.603395000	-4.514060000
1	-7.263803000	-4.651274000	6.775179000
1	-7.117662000	-0.459859000	4.365138000
1	-4.684807000	-6.516317000	3.600956000
1	-9.071837000	-3.094255000	0.230807000
1	8.333012000	-4.965973000	0.657301000
1	-9.325932000	5.152436000	-3.639784000

1	1.946680000	-0.235418000	-4.725859000
1	3.966511000	-6.962814000	-2.907501000
1	-0.303648000	-4.446153000	-0.692568000
1	4.536753000	-2.156070000	3.908119000

^{5}TS1$_{B}$:

6	8.867116000	-1.270304000	3.263077000
6	8.145353000	-0.136662000	2.548703000
8	8.154807000	-0.089128000	1.283268000
6	7.969549000	-2.535135000	3.454477000
6	7.090409000	-2.862333000	2.287850000
7	7.534918000	-3.184219000	0.997262000
6	5.722795000	-2.843204000	2.199749000
6	6.479368000	-3.332701000	0.158609000
7	5.373001000	-3.131721000	0.886130000
1	9.737709000	-1.520584000	2.649034000
1	8.619981000	-3.381897000	3.708697000
1	7.312932000	-2.379942000	4.316806000
1	4.982828000	-2.636525000	2.951354000
1	6.507438000	-3.538860000	-0.913758000
1	4.364881000	-3.024087000	0.532196000
7	7.500217000	0.778146000	3.313885000
6	6.587389000	1.789199000	2.768593000
6	7.097531000	2.585261000	1.597642000
8	6.298663000	2.964325000	0.694739000
1	7.392943000	0.648603000	4.318761000
1	5.636451000	1.350854000	2.453465000
7	8.405382000	2.922834000	1.507242000
6	8.871128000	3.698337000	0.354731000
6	8.460852000	3.088587000	-0.992903000
8	8.210324000	3.834150000	-1.971929000
1	9.045049000	2.662033000	2.242705000
1	8.459611000	4.710792000	0.369445000
7	8.422904000	1.728256000	-1.072727000
6	8.066597000	1.069654000	-2.323400000
6	6.575813000	1.067320000	-2.681277000
8	6.230472000	0.658429000	-3.822280000
1	8.509293000	1.156955000	-0.232360000
1	8.577405000	1.560653000	-3.156090000
7	5.709744000	1.495801000	-1.737160000
6	4.271534000	1.671689000	-1.997957000
6	3.930926000	3.171482000	-2.046807000
8	3.364874000	3.675446000	-3.057165000
6	3.387066000	0.939995000	-0.968053000
6	3.601621000	-0.578723000	-0.942912000
6	2.677545000	-1.279250000	0.039750000
8	1.676757000	-0.628831000	0.493438000
8	2.920596000	-2.521460000	0.368990000
1	6.073099000	1.835030000	-0.848305000
1	4.090443000	1.286445000	-3.002492000
1	2.339648000	1.144367000	-1.216063000
1	3.546333000	1.356654000	0.033178000
1	4.643294000	-0.825302000	-0.703415000
1	3.392980000	-1.025936000	-1.925678000
7	4.282023000	3.894604000	-0.955172000
6	3.978487000	5.311072000	-0.801949000
6	4.677731000	6.246186000	-1.776485000
8	4.213924000	7.413087000	-1.936906000
1	4.746148000	3.444670000	-0.168090000
1	2.906585000	5.507314000	-0.927852000
7	5.714819000	5.787038000	-2.512274000
6	6.195047000	6.561755000	-3.656063000
6	5.100117000	6.863759000	-4.693122000
8	5.254755000	7.817213000	-5.490164000
1	6.182633000	4.916909000	-2.280406000
1	6.588157000	7.533128000	-3.344732000
7	3.995871000	6.058638000	-4.687375000
6	2.853617000	6.311806000	-5.562227000
6	1.639494000	6.891986000	-4.811594000
8	0.516218000	6.943460000	-5.383736000
6	2.443120000	5.046148000	-6.333687000
1	3.926939000	5.251030000	-4.066919000
1	3.179104000	7.084282000	-6.270802000
1	1.542262000	5.242440000	-6.918533000
1	3.255231000	4.744497000	-7.002418000
1	2.233293000	4.228730000	-5.634667000
7	1.827245000	7.398865000	-3.561158000
6	0.684238000	7.839189000	-2.753175000
6	-0.041558000	9.061719000	-3.336300000
8	-1.213656000	9.326497000	-2.959513000
6	1.087135000	8.123564000	-1.298382000
8	1.022764000	6.871624000	-0.547376000
1	2.743023000	7.359010000	-3.110680000
1	-0.076057000	7.049559000	-2.741252000
1	2.097899000	8.542136000	-1.253965000
1	0.371842000	8.844085000	-0.884060000
1	1.200767000	7.021800000	0.412356000
7	0.628597000	9.809916000	-4.246933000
6	-0.013049000	10.908272000	-4.960863000
6	-1.089215000	10.498970000	-5.978244000
8	-1.786140000	11.398455000	-6.500900000
1	1.565082000	9.527112000	-4.500456000
1	-0.510488000	11.572736000	-4.248818000
7	-1.233408000	9.170151000	-6.241249000
6	-2.312754000	8.682548000	-7.090406000
6	-3.540986000	8.150331000	-6.343415000
8	-4.471860000	7.620148000	-7.001287000
1	-0.598578000	8.476014000	-5.847858000
1	-2.647438000	9.504677000	-7.729546000
7	-3.556388000	8.296855000	-4.992652000
6	-4.696031000	7.888080000	-4.171306000
6	-4.351813000	6.830419000	-3.108706000
6	-3.976263000	5.436547000	-3.595554000
6	-4.240562000	4.966198000	-4.894738000
6	-3.395484000	4.538400000	-2.682254000
6	-3.942412000	3.647689000	-5.257862000
6	-3.097701000	3.218195000	-3.028509000
6	-3.373193000	2.768480000	-4.328714000
8	-3.095237000	1.475323000	-4.741014000
1	-2.766902000	8.727709000	-4.523420000
1	-5.468596000	7.530762000	-4.856163000
1	-5.232258000	6.746438000	-2.450523000
1	-3.537749000	7.225774000	-2.482094000
1	-4.661042000	5.626794000	-5.646306000
1	-3.184293000	4.875878000	-1.668878000
1	-4.142110000	3.289763000	-6.261625000
1	-2.656699000	2.535465000	-2.310376000
1	-2.651019000	0.910590000	-4.054591000
6	-4.633990000	6.261610000	2.445211000
6	-3.151671000	6.497494000	2.300723000
8	-2.295021000	5.607246000	2.567699000
6	-5.169430000	5.157238000	1.472806000
6	-5.900143000	4.023601000	2.212184000
6	-4.909312000	3.106034000	2.932882000
8	-3.970009000	2.546727000	2.298344000
8	-5.128519000	3.016802000	4.229375000
1	-4.801910000	5.933245000	3.479124000
1	-5.858938000	5.609849000	0.753371000
1	-4.346652000	4.717772000	0.900076000
1	-6.447483000	3.388901000	1.509029000
1	-6.634730000	4.413907000	2.921459000
7	-2.730759000	7.736817000	1.896242000
6	-1.329835000	7.965455000	1.530142000
6	-0.334157000	7.626107000	2.636083000
8	0.805048000	7.167420000	2.335553000
1	-3.418360000	8.412429000	1.593541000
1	-1.041162000	7.359853000	0.664804000
7	-0.689746000	7.872751000	3.921127000
6	0.200124000	7.554363000	5.041644000
6	0.298538000	6.054471000	5.377202000
8	1.362717000	5.600016000	5.866774000
1	-1.607354000	8.256493000	4.100817000
1	1.213012000	7.891206000	4.814635000
7	-0.809042000	5.311790000	5.143370000
6	-0.858983000	3.844707000	5.334305000
6	0.122476000	3.188425000	4.339390000
8	1.026326000	2.405326000	4.731954000
6	-2.323862000	3.410610000	5.136019000
6	-2.722045000	2.068917000	5.730017000
8	-2.066727000	1.524027000	6.669648000

8	-3.814574000	1.529836000	5.240578000
1	-1.543579000	5.704673000	4.565194000
1	-0.507293000	3.588201000	6.334927000
1	-2.580693000	3.393557000	4.075729000
1	-2.976719000	4.157113000	5.608076000
7	-0.000604000	3.573939000	3.041952000
6	0.919937000	3.082737000	2.008548000
6	2.360879000	3.535462000	2.296152000
8	3.339343000	2.780399000	2.049456000
6	0.472370000	3.540516000	0.599012000
6	-0.905072000	3.024719000	0.107156000
6	-1.000629000	1.515445000	0.018749000
8	-0.739048000	0.845149000	1.072548000
8	-1.362033000	0.990504000	-1.123253000
1	-0.780069000	4.162708000	2.762417000
1	0.938851000	1.993242000	2.040221000
1	1.245729000	3.215653000	-0.105999000
1	0.453048000	4.635786000	0.549173000
1	-1.705613000	3.352141000	0.784338000
1	-1.101938000	3.456252000	-0.876327000
7	2.527173000	4.784591000	2.811794000
6	3.837166000	5.299483000	3.192505000
6	4.559663000	4.463175000	4.249582000
8	5.806659000	4.299772000	4.159825000
1	1.738465000	5.417965000	2.877090000
1	4.523769000	5.330509000	2.341797000
7	3.824500000	3.931969000	5.255581000
6	4.446471000	3.025742000	6.222067000
6	4.658755000	1.600990000	5.680839000
8	5.798095000	1.047127000	5.738605000
1	2.844969000	4.183315000	5.364326000
1	5.431869000	3.401608000	6.501749000
7	3.573147000	0.979152000	5.168194000
6	3.680154000	-0.373982000	4.608446000
6	2.597613000	-0.662430000	3.563061000
6	1.228376000	-0.874306000	4.132616000
7	0.106230000	-1.062274000	3.315686000
6	0.803344000	-0.885884000	5.446305000
6	-0.958078000	-1.163556000	4.128547000
7	-0.569580000	-1.069197000	5.417097000
1	2.690486000	1.482712000	5.053269000
1	3.647191000	-1.117591000	5.418632000
1	2.889020000	-1.554075000	2.990630000
1	2.575577000	0.162043000	2.840831000
1	1.332078000	-0.708064000	6.366327000
1	-1.982812000	-1.273967000	3.819487000
6	1.440066000	0.622957000	-4.811067000
6	2.360937000	-0.321254000	-5.546102000
8	2.285085000	-1.581974000	-5.397340000
6	0.063005000	0.797336000	-5.519630000
6	-0.783160000	-0.482784000	-5.677123000
6	-1.144622000	-1.197125000	-4.374947000
8	-1.527088000	-0.371729000	-3.384459000
8	-1.083918000	-2.439497000	-4.271072000
1	1.267451000	0.222196000	-3.806751000
1	-0.508203000	1.527293000	-4.941454000
1	0.223580000	1.234593000	-6.514239000
1	-0.291481000	-1.221437000	-6.316017000
1	-1.724244000	-0.188917000	-6.159464000
7	3.260886000	0.203219000	-6.418436000
6	4.021813000	-0.628030000	-7.359792000
6	4.946336000	-1.684490000	-6.744657000
8	5.188826000	-2.729956000	-7.388843000
1	3.315485000	1.207933000	-6.507920000
1	3.340653000	-1.183401000	-8.010764000
7	5.478217000	-1.414988000	-5.520520000
6	6.334322000	-2.367284000	-4.829343000
6	5.630473000	-3.137341000	-3.709350000
8	6.300903000	-3.590317000	-2.732970000
1	5.381433000	-0.505501000	-5.075738000
1	6.701960000	-3.093446000	-5.563611000
7	4.307638000	-3.340893000	-3.854941000
6	3.510125000	-4.150641000	-2.925088000
6	2.018056000	-4.037836000	-3.250105000
8	1.620955000	-2.632040000	-3.075371000

6	1.151472000	-4.918489000	-2.357629000
1	3.824774000	-2.877848000	-4.621172000
1	3.667854000	-3.802552000	-1.898994000
1	1.842854000	-4.300205000	-4.301599000
1	1.647747000	-2.176476000	-3.964193000
1	0.098505000	-4.768715000	-2.611181000
1	1.398690000	-5.976007000	-2.503539000
1	1.298116000	-4.671661000	-1.301091000
6	-5.429675000	-4.422963000	2.952897000
6	-6.586868000	-3.777704000	3.705776000
8	-7.680679000	-3.534925000	3.121348000
6	-5.958492000	-5.472833000	1.953740000
6	-4.884643000	-6.065396000	1.002540000
6	-4.189399000	-4.991478000	0.189896000
8	-3.202355000	-4.356930000	0.670132000
7	-4.688656000	-4.673012000	-1.021969000
1	-4.888838000	-3.633904000	2.413802000
1	-6.763737000	-5.010890000	1.372960000
1	-6.415523000	-6.303262000	2.506668000
1	-5.359997000	-6.803097000	0.345036000
1	-4.114674000	-6.585607000	1.582263000
1	-4.266693000	-3.892050000	-1.530272000
1	-5.466376000	-5.170062000	-1.425958000
7	-6.385080000	-3.472520000	5.015070000
6	-7.471280000	-2.950230000	5.844413000
6	-8.046543000	-1.590863000	5.434259000
8	-9.208565000	-1.284501000	5.788297000
1	-5.441241000	-3.490768000	5.405918000
1	-8.318002000	-3.641949000	5.834118000
7	-7.229640000	-0.775441000	4.713764000
6	-7.689805000	0.504705000	4.199903000
6	-7.840194000	0.551035000	2.679396000
8	-7.905774000	1.670045000	2.103658000
1	-6.255023000	-1.024069000	4.529908000
1	-8.666876000	0.710659000	4.650415000
7	-7.928022000	-0.638489000	2.029197000
6	-8.128013000	-0.728185000	0.584262000
6	-6.935106000	-1.312916000	-0.167477000
8	-5.797879000	-0.406359000	-0.093922000
1	-7.919371000	-1.505209000	2.564070000
1	-8.335404000	0.277904000	0.214438000
1	-6.662288000	-2.291245000	0.252409000
1	-7.214085000	-1.452034000	-1.219482000
1	-5.140574000	-0.535048000	-0.838873000
6	-9.837401000	6.263336000	-2.900041000
6	-8.993726000	5.145373000	-2.324307000
6	-9.323123000	4.551923000	-1.093503000
6	-7.874051000	4.657331000	-3.022227000
6	-8.564385000	3.494471000	-0.575319000
6	-7.110477000	3.601240000	-2.511188000
6	-7.454504000	3.015362000	-1.285577000
1	-9.226878000	6.979719000	-3.461382000
1	-10.596281000	5.874688000	-3.593364000
1	-10.183366000	4.919872000	-0.538937000
1	-7.598154000	5.104367000	-3.973834000
1	-8.820550000	3.041706000	0.377182000
1	-6.249319000	3.244009000	-3.066061000
1	-6.869529000	2.191989000	-0.885342000
26	-0.207955000	-1.157167000	1.250632000
26	-1.809801000	-0.871540000	-1.554962000
8	0.310104000	-3.190719000	0.886472000
1	1.299797000	-3.243792000	0.851380000
1	-0.206512000	-3.831551000	1.538577000
8	-4.386132000	-0.829343000	4.122322000
1	-4.224974000	0.016333000	4.627502000
1	-4.250915000	-0.642214000	3.134242000
8	-3.972822000	-0.044129000	1.675461000
1	-4.795379000	-0.162255000	1.081665000
1	-3.822821000	0.924401000	1.867396000
8	-2.113111000	-1.399173000	0.395891000
1	-2.868191000	-2.173553000	-0.305850000
1	-2.759233000	-0.792085000	0.904046000
8	-0.886044000	-2.508814000	8.778867000
1	-0.512339000	-3.132585000	8.070934000
1	-0.875552000	-2.888258000	9.670223000

8	-3.730693000	-0.823762000	-1.706535000
8	-3.261024000	-2.256562000	-1.458528000
8	-0.229525000	-1.983274000	-1.414866000
1	0.432562000	-2.204210000	-2.157899000
8	-1.172918000	-4.535842000	2.362321000
1	-2.009686000	-4.569136000	1.825464000
1	-1.347198000	-4.423178000	3.331903000
8	-2.450925000	-1.109221000	7.371923000
1	-1.917827000	-1.530837000	8.128251000
1	-2.504759000	-0.116193000	7.348969000
8	-1.925090000	-4.451501000	5.019838000
1	-2.256785000	-5.358215000	5.155397000
1	-2.664866000	-3.731667000	5.265436000
8	-0.015151000	-3.906199000	6.783361000
1	-0.708180000	-4.158631000	6.096670000
1	0.747905000	-3.490673000	6.345832000
8	-3.621347000	-2.722405000	5.669585000
1	-3.925040000	-2.051363000	4.960256000
1	-3.299998000	-2.174991000	6.460406000
1	3.808006000	2.986889000	7.109585000
1	3.695607000	6.319716000	3.561620000
1	-0.150991000	8.095049000	5.925675000
1	-1.219235000	9.020178000	1.257219000
1	-5.190179000	7.196192000	2.312674000
1	-5.096775000	8.773698000	-3.659434000
1	-1.947355000	7.875428000	-7.731082000
1	0.754825000	11.486546000	-5.481200000
1	7.004809000	5.999024000	-4.128294000
1	4.242836000	5.600659000	0.218382000
1	8.406277000	0.030597000	-2.282726000
1	9.962185000	3.766488000	0.404025000
1	6.369433000	2.507102000	3.567292000
1	9.233922000	-0.955601000	4.245833000
1	4.619057000	0.041920000	-7.986147000
1	7.185245000	-1.846044000	-4.388594000
1	-7.102451000	-2.880517000	6.872672000
1	-7.005071000	1.309262000	4.482915000
1	-4.711321000	-4.868504000	3.651573000
1	-9.002655000	-1.360526000	0.383073000
1	8.497756000	-3.210168000	0.699826000
1	-10.367581000	6.811706000	-2.113293000
1	1.908435000	1.607692000	-4.690886000
1	3.831847000	-5.198745000	-2.981383000
1	-0.170077000	-2.651438000	-0.671946000
1	4.670480000	-0.459047000	4.148998000

^{5}IM1$_B$:

6	8.719687000	-3.057376000	3.210699000
6	8.038091000	-1.901383000	2.492884000
8	8.074997000	-1.842045000	1.228255000
6	7.784907000	-4.296645000	3.387577000
6	6.917540000	-4.602997000	2.206259000
7	7.375389000	-4.915867000	0.918176000
6	5.551034000	-4.575951000	2.102216000
6	6.329053000	-5.051822000	0.066021000
7	5.214735000	-4.850740000	0.781999000
1	9.590608000	-3.329409000	2.606485000
1	8.407852000	-5.160271000	3.653926000
1	7.117634000	-4.122282000	4.238031000
1	4.803644000	-4.373824000	2.847633000
1	6.372170000	-5.251738000	-1.007289000
1	4.211197000	-4.733251000	0.414968000
7	7.396800000	-0.981496000	3.254835000
6	6.509929000	0.050016000	2.704986000
6	7.044798000	0.840256000	1.541806000
8	6.261176000	1.226131000	0.628170000
1	7.261043000	-1.123715000	4.255040000
1	5.553923000	-0.368804000	2.378568000
7	8.355955000	1.167929000	1.470008000
6	8.843680000	1.941770000	0.325140000
6	8.447176000	1.337391000	-1.029074000
8	8.213022000	2.085834000	-2.009687000
1	8.983630000	0.900017000	2.213278000
1	8.439885000	2.957347000	0.336320000
7	8.403259000	-0.022796000	-1.111349000
6	8.061898000	-0.677825000	-2.367803000
6	6.577174000	-0.669160000	-2.747569000
8	6.246461000	-1.066924000	-3.896900000
1	8.473444000	-0.595863000	-0.270701000
1	8.588324000	-0.189876000	-3.192498000
7	5.698367000	-0.243561000	-1.813342000
6	4.267130000	-0.055180000	-2.098703000
6	3.926368000	1.444472000	-2.134852000
8	3.357938000	1.952189000	-3.143098000
6	3.356306000	-0.795018000	-1.099906000
6	3.512242000	-2.322272000	-1.133936000
6	2.529007000	-3.013185000	-0.201693000
8	1.482869000	-2.364654000	0.132979000
8	2.773338000	-4.231250000	0.209747000
1	6.050125000	0.087892000	-0.917024000
1	4.104378000	-0.426633000	-3.111518000
1	2.317068000	-0.544602000	-1.336942000
1	3.529320000	-0.421743000	-0.083444000
1	4.535610000	-2.621805000	-0.881100000
1	3.309718000	-2.716852000	-2.140710000
7	4.271345000	2.158889000	-1.037114000
6	3.964140000	3.573026000	-0.869688000
6	4.665934000	4.517186000	-1.833430000
8	4.205700000	5.686913000	-1.982110000
1	4.731621000	1.703631000	-0.250659000
1	2.891924000	3.768954000	-0.994851000
7	5.701185000	4.062870000	-2.575305000
6	6.180589000	4.848193000	-3.712352000
6	5.083416000	5.163945000	-4.743188000
8	5.236584000	6.127466000	-5.528186000
1	6.167490000	3.190170000	-2.350692000
1	6.577222000	5.815177000	-3.392074000
7	3.978685000	4.359124000	-4.745953000
6	2.835637000	4.623753000	-5.616633000
6	1.628105000	5.212382000	-4.861681000
8	0.506708000	5.286863000	-5.435296000
6	2.412751000	3.362469000	-6.388840000
1	3.910357000	3.544169000	-4.135061000
1	3.164490000	5.394994000	-6.324843000
1	1.510459000	3.566562000	-6.968872000
1	3.219141000	3.056746000	-7.062563000
1	2.201187000	2.544627000	-5.690651000
7	1.820661000	5.699008000	-3.604218000
6	0.683420000	6.141388000	-2.789887000
6	-0.019221000	7.388334000	-3.347824000
8	-1.185694000	7.668339000	-2.962610000
6	1.086333000	6.384755000	-1.327832000
8	1.003426000	5.116223000	-0.608343000
1	2.736246000	5.645246000	-3.155032000
1	-0.090050000	5.364514000	-2.798272000
1	2.102449000	6.788639000	-1.271297000
1	0.380558000	7.105609000	-0.898200000
1	1.141536000	5.249614000	0.360966000
7	0.661170000	8.137547000	-4.249093000
6	0.037793000	9.256845000	-4.946682000
6	-1.063762000	8.878842000	-5.948838000
8	-1.760077000	9.795182000	-6.441802000
1	1.592764000	7.842812000	-4.507269000
1	-0.431784000	9.930998000	-4.224927000
7	-1.229819000	7.556399000	-6.231798000
6	-2.336955000	7.097255000	-7.060729000
6	-3.556684000	6.579507000	-6.289974000
8	-4.509993000	6.067363000	-6.929942000
1	-0.595799000	6.847607000	-5.863997000
1	-2.671728000	7.931654000	-7.683765000
7	-3.540345000	6.717901000	-4.938269000
6	-4.668849000	6.324918000	-4.094573000
6	-4.320085000	5.253149000	-3.047252000
6	-3.991145000	3.854053000	-3.553132000
6	-4.276705000	3.410435000	-4.857256000
6	-3.431317000	2.926907000	-2.655893000
6	-4.019184000	2.089532000	-5.240697000
6	-3.175522000	1.603252000	-3.022467000
6	-3.471920000	1.181451000	-4.327015000
8	-3.238872000	-0.114422000	-4.759951000

1	-2.735094000	7.134648000	-4.482636000
1	-5.463915000	5.986633000	-4.763288000
1	-5.184025000	5.185214000	-2.365643000
1	-3.479948000	5.626511000	-2.441721000
1	-4.684639000	4.092489000	-5.596344000
1	-3.200875000	3.244270000	-1.640328000
1	-4.235410000	1.751355000	-6.247770000
1	-2.752654000	0.897824000	-2.314746000
1	-2.809606000	-0.702632000	-4.085871000
6	-4.722642000	4.455966000	2.241736000
6	-3.242624000	4.721293000	2.129521000
8	-2.375700000	3.851190000	2.427812000
6	-5.216350000	3.355011000	1.242517000
6	-5.951127000	2.202923000	1.950310000
6	-4.965935000	1.303684000	2.701897000
8	-4.036740000	0.708252000	2.087832000
8	-5.177937000	1.273759000	4.003267000
1	-4.903690000	4.107965000	3.266693000
1	-5.892956000	3.804864000	0.509163000
1	-4.372643000	2.933709000	0.686719000
1	-6.460273000	1.562249000	1.224710000
1	-6.715754000	2.578979000	2.635184000
7	-2.835099000	5.961771000	1.715355000
6	-1.430277000	6.207993000	1.374997000
6	-0.453530000	5.900884000	2.507446000
8	0.692198000	5.436478000	2.243207000
1	-3.527345000	6.620265000	1.386535000
1	-1.114309000	5.594463000	0.525140000
7	-0.831758000	6.182879000	3.778954000
6	0.045396000	5.901875000	4.920103000
6	0.165582000	4.408329000	5.276271000
8	1.242956000	3.965630000	5.745634000
1	-1.751575000	6.572009000	3.933615000
1	1.055678000	6.254241000	4.705480000
7	-0.943571000	3.656084000	5.075899000
6	-0.985459000	2.190808000	5.280374000
6	0.024383000	1.529399000	4.318748000
8	0.923728000	0.755979000	4.741185000
6	-2.441939000	1.745403000	5.041155000
6	-2.825958000	0.381979000	5.592296000
8	-2.199877000	-0.139920000	6.565054000
8	-3.864908000	-0.195333000	5.038386000
1	-1.692832000	4.043067000	4.514564000
1	-0.662871000	1.943174000	6.292919000
1	-2.677317000	1.758583000	3.976579000
1	-3.111906000	2.469992000	5.523841000
7	-0.078526000	1.895271000	3.015044000
6	0.837872000	1.371502000	1.995489000
6	2.281955000	1.826203000	2.252153000
8	3.254983000	1.072350000	1.977959000
6	0.369801000	1.780857000	0.580703000
6	-1.008523000	1.225036000	0.143353000
6	-1.046896000	-0.283620000	0.022370000
8	-0.680437000	-0.964662000	1.047238000
8	-1.440136000	-0.797623000	-1.105070000
1	-0.849854000	2.486916000	2.718163000
1	0.848095000	0.282830000	2.059337000
1	1.133926000	1.442285000	-0.127773000
1	0.335681000	2.873874000	0.497756000
1	-1.795045000	1.514514000	0.852315000
1	-1.260209000	1.657433000	-0.827011000
7	2.456304000	3.069772000	2.776861000
6	3.768382000	3.574526000	3.162731000
6	4.478981000	2.718692000	4.212713000
8	5.725506000	2.548772000	4.132952000
1	1.665577000	3.696743000	2.867439000
1	4.455987000	3.610493000	2.313206000
7	3.728374000	2.172884000	5.199685000
6	4.325189000	1.228798000	6.145397000
6	4.529367000	-0.178238000	5.556727000
8	5.657618000	-0.753750000	5.622979000
1	2.745220000	2.414231000	5.289131000
1	5.309860000	1.583966000	6.452990000
7	3.449493000	-0.759383000	4.988546000
6	3.553155000	-2.085941000	4.367695000
6	2.458881000	-2.334445000	3.324059000
6	1.095012000	-2.548643000	3.903997000
7	-0.026200000	-2.769532000	3.097085000
6	0.678024000	-2.533654000	5.221501000
6	-1.084066000	-2.865383000	3.915092000
7	-0.692065000	-2.733080000	5.203636000
1	2.573473000	-0.240875000	4.887842000
1	3.527971000	-2.863622000	5.145769000
1	2.736325000	-3.214224000	2.727175000
1	2.439908000	-1.491935000	2.621729000
1	1.208530000	-2.321263000	6.133269000
1	-2.108082000	-2.996974000	3.612908000
6	1.363663000	-1.109984000	-4.680269000
6	2.246055000	-2.025333000	-5.498657000
8	2.159798000	-3.291024000	-5.428518000
6	0.014605000	-0.772048000	-5.380514000
6	-0.895717000	-1.977462000	-5.692931000
6	-1.306097000	-2.799949000	-4.475830000
8	-1.767819000	-2.059390000	-3.436766000
8	-1.220849000	-4.039490000	-4.455970000
1	1.154667000	-1.606843000	-3.727128000
1	-0.525371000	-0.080250000	-4.728648000
1	0.216730000	-0.234565000	-6.316317000
1	-0.426117000	-2.670602000	-6.396364000
1	-1.810670000	-1.584735000	-6.153242000
7	3.132818000	-1.455700000	-6.357630000
6	3.872172000	-2.241483000	-7.353370000
6	4.797273000	-3.329999000	-6.797821000
8	5.002448000	-4.363686000	-7.472680000
1	3.197198000	-0.447933000	-6.388820000
1	3.177679000	-2.761959000	-8.018647000
7	5.371400000	-3.096958000	-5.584792000
6	6.236536000	-4.074907000	-4.942391000
6	5.558056000	-4.850101000	-3.811142000
8	6.245402000	-5.297967000	-2.844086000
1	5.312312000	-2.192968000	-5.122464000
1	6.563600000	-4.794055000	-5.702415000
7	4.234116000	-5.067940000	-3.935287000
6	3.475381000	-5.908759000	-3.000685000
6	1.987050000	-5.936698000	-3.362268000
8	1.445386000	-4.582227000	-3.220364000
6	1.195978000	-6.869943000	-2.450808000
1	3.730253000	-4.604986000	-4.687633000
1	3.570867000	-5.519558000	-1.981309000
1	1.862742000	-6.247760000	-4.408479000
1	1.490167000	-4.088386000	-4.085826000
1	0.137290000	-6.844532000	-2.722848000
1	1.555255000	-7.900840000	-2.542989000
1	1.294211000	-6.559451000	-1.404867000
6	-5.462314000	-6.314299000	3.202564000
6	-6.641657000	-5.602563000	3.850855000
8	-7.723839000	-5.438817000	3.220446000
6	-5.941361000	-7.551728000	2.416575000
6	-4.847778000	-8.203659000	1.526882000
6	-4.299922000	-7.208422000	0.525149000
8	-3.322158000	-6.455859000	0.824517000
7	-4.936818000	-7.070054000	-0.653267000
1	-4.975508000	-5.606349000	2.519634000
1	-6.795840000	-7.250161000	1.801127000
1	-6.311908000	-8.311316000	3.116570000
1	-5.272237000	-9.076324000	1.016222000
1	-4.012262000	-8.551049000	2.143218000
1	-4.654096000	-6.291674000	-1.255669000
1	-5.724896000	-7.644983000	-0.905492000
7	-6.466017000	-5.151787000	5.121659000
6	-7.571877000	-4.551588000	5.865868000
6	-8.126001000	-3.235670000	5.312370000
8	-9.289429000	-2.883336000	5.615403000
1	-5.524260000	-5.117740000	5.518225000
1	-8.421364000	-5.239312000	5.894852000
7	-7.288719000	-2.503620000	4.528458000
6	-7.718149000	-1.263369000	3.904398000
6	-7.820330000	-1.327893000	2.381433000
8	-7.861424000	-0.252865000	1.725325000
1	-6.314326000	-2.775464000	4.382582000

1	-8.707903000	-1.017126000	4.304835000
7	-7.901448000	-2.559605000	1.813529000
6	-8.111901000	-2.746416000	0.377016000
6	-6.921530000	-3.348083000	-0.364064000
8	-5.833408000	-2.380666000	-0.445957000
1	-7.909930000	-3.383825000	2.410937000
1	-8.349183000	-1.770167000	-0.051203000
1	-6.571366000	-4.262188000	0.132552000
1	-7.237808000	-3.610108000	-1.381435000
1	-5.171092000	-2.632019000	-1.153781000
6	-9.870762000	4.705839000	-2.821134000
6	-9.056328000	3.523338000	-2.339818000
6	-9.407902000	2.833880000	-1.166168000
6	-7.944245000	3.069888000	-3.071919000
6	-8.679246000	1.716535000	-0.739389000
6	-7.211192000	1.953513000	-2.651633000
6	-7.577910000	1.271433000	-1.483999000
1	-10.648129000	4.392198000	-3.531736000
1	-10.376358000	5.210036000	-1.989816000
1	-10.262050000	3.174066000	-0.584984000
1	-7.651345000	3.590116000	-3.980340000
1	-8.947476000	1.191882000	0.171346000
1	-6.356504000	1.623510000	-3.232852000
1	-7.018724000	0.401216000	-1.152330000
26	-0.299511000	-2.896091000	1.041384000
26	-1.981247000	-2.667404000	-1.645198000
8	0.181525000	-4.815556000	0.860723000
1	1.169286000	-4.886675000	0.788837000
1	-0.324787000	-5.448867000	1.549323000
8	-4.454989000	-2.572172000	3.913442000
1	-4.276911000	-1.707726000	4.376956000
1	-4.363393000	-2.435810000	2.912594000
8	-4.118241000	-1.883498000	1.409637000
1	-4.898904000	-2.040147000	0.767407000
1	-3.988860000	-0.911024000	1.587253000
8	-2.123933000	-3.143442000	0.196415000
1	-3.287233000	-4.485932000	-0.395163000
1	-2.840697000	-2.610406000	0.682611000
8	-0.874205000	-4.068370000	8.652506000
1	-0.480278000	-4.696284000	7.959552000
1	-0.836539000	-4.416926000	9.555594000
8	-3.773792000	-3.277854000	-1.916697000
8	-3.920013000	-4.586067000	-1.164966000
8	-0.524886000	-4.022405000	-1.631025000
1	0.136122000	-4.265965000	-2.354078000
8	-1.195685000	-6.196806000	2.375525000
1	-2.028324000	-6.395014000	1.867250000
1	-1.372577000	-6.068376000	3.343941000
8	-2.510249000	-2.749673000	7.239141000
1	-1.946041000	-3.149368000	7.984486000
1	-2.568951000	-1.755142000	7.199799000
8	-1.958490000	-6.092553000	5.010506000
1	-2.271101000	-7.000536000	5.177731000
1	-2.712757000	-5.379372000	5.236140000
8	0.017488000	-5.479389000	6.677794000
1	-0.703127000	-5.759492000	6.032193000
1	0.725065000	-5.015572000	6.196383000
8	-3.691434000	-4.375609000	5.596035000
1	-3.993006000	-3.754012000	4.846369000
1	-3.361143000	-3.789006000	6.360500000
1	3.672275000	1.166898000	7.020886000
1	3.632619000	4.590760000	3.544369000
1	-0.333549000	6.449009000	5.788488000
1	-1.331474000	7.260075000	1.087904000
1	-5.294636000	5.381101000	2.110640000
1	-5.040732000	7.214145000	-3.567499000
1	-2.001251000	6.290446000	-7.717696000
1	0.813241000	9.817056000	-5.475536000
1	6.987348000	4.288406000	-4.193193000
1	4.226162000	3.851834000	0.154351000
1	8.393640000	-1.719278000	-2.323137000
1	9.934435000	2.001122000	0.389716000
1	6.296129000	0.768282000	3.504864000
1	9.082928000	-2.756198000	4.198988000
1	4.463766000	-1.544368000	-7.954926000
1	7.111257000	-3.575377000	-4.524068000
1	-7.234901000	-4.385104000	6.893987000
1	-7.037010000	-0.444348000	4.152503000
1	-4.711716000	-6.591007000	3.951438000
1	-8.974634000	-3.409724000	0.233309000
1	8.341442000	-4.941773000	0.631215000
1	-9.244590000	5.443329000	-3.335965000
1	1.887018000	-0.171846000	-4.455095000
1	3.887412000	-6.925889000	-3.014831000
1	-0.387511000	-4.537607000	-0.792715000
1	4.538377000	-2.150093000	3.894279000

^{5}TS2$_B$:

6	8.785000000	-3.040057000	3.150372000
6	8.079138000	-1.892230000	2.442827000
8	8.110537000	-1.821723000	1.178614000
6	7.868880000	-4.291362000	3.337105000
6	7.000790000	-4.613003000	2.160660000
7	7.458907000	-4.919051000	0.870997000
6	5.633555000	-4.606832000	2.061467000
6	6.410687000	-5.070911000	0.023067000
7	5.296580000	-4.887116000	0.742867000
1	9.652151000	-3.299543000	2.535307000
1	8.505408000	-5.145083000	3.603508000
1	7.203539000	-4.123107000	4.190289000
1	4.885080000	-4.416036000	2.808994000
1	6.453112000	-5.266021000	-1.050824000
1	4.284603000	-4.781863000	0.381074000
7	7.423903000	-0.990904000	3.215003000
6	6.516166000	0.029941000	2.679962000
6	7.029037000	0.837095000	1.518760000
8	6.233041000	1.220657000	0.614617000
1	7.295767000	-1.143388000	4.214746000
1	5.562934000	-0.400401000	2.360794000
7	8.334857000	1.183291000	1.436255000
6	8.798403000	1.970253000	0.290655000
6	8.385574000	1.372407000	-1.061722000
8	8.121535000	2.126654000	-2.031047000
1	8.974466000	0.917478000	2.169960000
1	8.385972000	2.982143000	0.315526000
7	8.362101000	0.013080000	-1.156907000
6	8.008751000	-0.634850000	-2.414017000
6	6.519349000	-0.630736000	-2.775974000
8	6.177942000	-1.001687000	-3.931370000
1	8.455970000	-0.566830000	-0.322836000
1	8.523006000	-0.139796000	-3.242028000
7	5.647861000	-0.241455000	-1.819713000
6	4.209272000	-0.071838000	-2.080230000
6	3.852225000	1.423871000	-2.104444000
8	3.265697000	1.931828000	-3.103593000
6	3.327750000	-0.834862000	-1.071582000
6	3.519028000	-2.357496000	-1.118309000
6	2.582685000	-3.082025000	-0.162408000
8	1.547559000	-2.457251000	0.239500000
8	2.856189000	-4.308912000	0.195674000
1	6.006438000	0.075642000	-0.920838000
1	4.036836000	-0.440285000	-3.092346000
1	2.279229000	-0.606724000	-1.291119000
1	3.507917000	-0.461835000	-0.056269000
1	4.557199000	-2.633428000	-0.899465000
1	3.292376000	-2.753612000	-2.119219000
7	4.204175000	2.140057000	-1.010306000
6	3.885750000	3.551148000	-0.839886000
6	4.564397000	4.500830000	-1.814957000
8	4.080204000	5.660454000	-1.971687000
1	4.679203000	1.688014000	-0.230066000
1	2.810913000	3.737136000	-0.953372000
7	5.605222000	4.061812000	-2.557494000
6	6.067613000	4.850822000	-3.699001000
6	4.960266000	5.146720000	-4.724270000
8	5.090522000	6.112562000	-5.510696000
1	6.092383000	3.201411000	-2.327084000
1	6.451705000	5.824614000	-3.383994000
7	3.870356000	4.321444000	-4.722065000
6	2.719809000	4.567101000	-5.587402000

6	1.507342000	5.137467000	-4.827112000
8	0.376258000	5.178980000	-5.387788000
6	2.314964000	3.300299000	-6.359713000
1	3.816619000	3.507983000	-4.107269000
1	3.033418000	5.344571000	-6.296150000
1	1.409199000	3.490633000	-6.939094000
1	3.125144000	3.007854000	-7.034695000
1	2.117746000	2.478881000	-5.661711000
7	1.702939000	5.651438000	-3.581959000
6	0.564351000	6.098962000	-2.772021000
6	-0.172174000	7.301815000	-3.379229000
8	-1.360578000	7.543913000	-3.036526000
6	0.979396000	6.413545000	-1.325834000
8	0.911715000	5.176122000	-0.555177000
1	2.620152000	5.609534000	-3.132498000
1	-0.190299000	5.305155000	-2.734801000
1	1.992459000	6.827585000	-1.297558000
1	0.271809000	7.147035000	-0.919846000
1	1.109354000	5.334204000	0.399998000
7	0.507748000	8.061545000	-4.272429000
6	-0.128803000	9.155804000	-4.998037000
6	-1.213966000	8.740707000	-6.002490000
8	-1.929354000	9.633309000	-6.510929000
1	1.455944000	7.794935000	-4.500041000
1	-0.616795000	9.837188000	-4.295343000
7	-1.348111000	7.411158000	-6.271155000
6	-2.440635000	6.918087000	-7.098278000
6	-3.641684000	6.370301000	-6.321602000
8	-4.562489000	5.783252000	-6.946055000
1	-0.706018000	6.719598000	-5.884523000
1	-2.799358000	7.742140000	-7.722494000
7	-3.651366000	6.566582000	-4.976117000
6	-4.782282000	6.175267000	-4.136212000
6	-4.424960000	5.127733000	-3.068317000
6	-4.046006000	3.738456000	-3.541780000
6	-4.315339000	3.257409000	-4.847184000
6	-3.441711000	2.854415000	-2.610955000
6	-4.007889000	1.950788000	-5.196705000
6	-3.125523000	1.548728000	-2.939099000
6	-3.405507000	1.064693000	-4.253403000
8	-3.114457000	-0.173154000	-4.621965000
1	-2.868477000	7.031035000	-4.525412000
1	-5.569971000	5.817281000	-4.803403000
1	-5.297268000	5.030422000	-2.400266000
1	-3.608101000	5.527318000	-2.449057000
1	-4.743362000	3.916440000	-5.595854000
1	-3.229707000	3.215826000	-1.607972000
1	-4.208195000	1.573180000	-6.192815000
1	-2.662465000	0.872502000	-2.227937000
1	-2.477778000	-1.004351000	-3.964026000
6	-4.671078000	4.550203000	2.440392000
6	-3.194663000	4.814675000	2.287203000
8	-2.324504000	3.924347000	2.499777000
6	-5.207824000	3.514950000	1.394354000
6	-5.956801000	2.345863000	2.058692000
6	-4.972077000	1.409156000	2.766877000
8	-4.064264000	0.821105000	2.117360000
8	-5.162666000	1.350928000	4.071010000
1	-4.813011000	4.135399000	3.446301000
1	-5.882441000	4.018619000	0.694207000
1	-4.380102000	3.098178000	0.811097000
1	-6.478415000	1.737826000	1.313962000
1	-6.707180000	2.707927000	2.767122000
7	-2.789915000	6.074352000	1.928080000
6	-1.393875000	6.319837000	1.552569000
6	-0.384826000	5.960737000	2.641530000
8	0.749823000	5.505024000	2.322658000
1	-3.485218000	6.761135000	1.672452000
1	-1.111373000	5.729933000	0.674530000
7	-0.730471000	6.189754000	3.932934000
6	0.165792000	5.854008000	5.043273000
6	0.252851000	4.350058000	5.363303000
8	1.314583000	3.880844000	5.842166000
1	-1.646137000	6.572324000	4.124592000
1	1.179603000	6.183751000	4.810102000
7	-0.863360000	3.621328000	5.123010000
6	-0.930783000	2.151777000	5.288923000
6	0.074350000	1.503207000	4.313335000
8	0.974731000	0.723242000	4.719570000
6	-2.391907000	1.732018000	5.035100000
6	-2.807820000	0.385345000	5.602818000
8	-2.180202000	-0.153852000	6.564230000
8	-3.876675000	-0.161524000	5.073423000
1	-1.595344000	4.033056000	4.556714000
1	-0.615211000	1.874159000	6.295921000
1	-2.616538000	1.729061000	3.967644000
1	-3.056326000	2.474774000	5.496882000
7	-0.028940000	1.896163000	3.017176000
6	0.887680000	1.394042000	1.986760000
6	2.330418000	1.833806000	2.267223000
8	3.303973000	1.082254000	1.991892000
6	0.437563000	1.856124000	0.581115000
6	-0.945039000	1.344982000	0.106404000
6	-1.038860000	-0.167030000	-0.004927000
8	-0.703388000	-0.846780000	1.024258000
8	-1.457911000	-0.681139000	-1.124125000
1	-0.806619000	2.483310000	2.730738000
1	0.886803000	0.304008000	2.014410000
1	1.203180000	1.523058000	-0.128901000
1	0.426928000	2.951736000	0.533676000
1	-1.737610000	1.666708000	0.795235000
1	-1.151210000	1.791658000	-0.868766000
7	2.507119000	3.072192000	2.807463000
6	3.820843000	3.566106000	3.200744000
6	4.532656000	2.690221000	4.233546000
8	5.780201000	2.527104000	4.150493000
1	1.718347000	3.700632000	2.900881000
1	4.507480000	3.616179000	2.350733000
7	3.783252000	2.124099000	5.209105000
6	4.382073000	1.173202000	6.146571000
6	4.572697000	-0.236125000	5.558736000
8	5.704750000	-0.807439000	5.601248000
1	2.798539000	2.358111000	5.300538000
1	5.371806000	1.522574000	6.444138000
7	3.480096000	-0.825673000	5.025115000
6	3.567784000	-2.165236000	4.428063000
6	2.475259000	-2.420560000	3.384519000
6	1.105487000	-2.618031000	3.958052000
7	-0.010815000	-2.828690000	3.141215000
6	0.679153000	-2.598894000	5.272290000
6	-1.075383000	-2.915277000	3.952243000
7	-0.693081000	-2.786751000	5.243942000
1	2.603143000	-0.307818000	4.931687000
1	3.530419000	-2.927809000	5.220141000
1	2.749798000	-3.310979000	2.801896000
1	2.463890000	-1.588513000	2.669817000
1	1.205260000	-2.397172000	6.189079000
1	-2.098404000	-3.038195000	3.641503000
6	1.454694000	-1.063729000	-4.769067000
6	2.318104000	-1.997467000	-5.591249000
8	2.200707000	-3.258256000	-5.531381000
6	0.124374000	-0.670568000	-5.473919000
6	-0.836574000	-1.841463000	-5.788619000
6	-1.230685000	-2.647872000	-4.559790000
8	-1.858545000	-1.939669000	-3.569657000
8	-0.960828000	-3.847911000	-4.437916000
1	1.227451000	-1.566569000	-3.823198000
1	-0.386120000	0.048164000	-4.825369000
1	0.350139000	-0.145529000	-6.411291000
1	-0.386910000	-2.544316000	-6.494130000
1	-1.741703000	-1.421093000	-6.240928000
7	3.224822000	-1.437321000	-6.438858000
6	3.961817000	-2.234064000	-7.425866000
6	4.898878000	-3.308154000	-6.861375000
8	5.144115000	-4.327063000	-7.545271000
1	3.325240000	-0.432343000	-6.449846000
1	3.264931000	-2.768988000	-8.077013000
7	5.435989000	-3.076497000	-5.631672000
6	6.310739000	-4.038753000	-4.979538000
6	5.637890000	-4.820641000	-3.849425000

8	6.334509000	-5.272818000	-2.891028000
1	5.340498000	-2.179052000	-5.162212000
1	6.654057000	-4.754733000	-5.735300000
7	4.313907000	-5.037173000	-3.965982000
6	3.556436000	-5.877341000	-3.029032000
6	2.066699000	-5.892113000	-3.384174000
8	1.542377000	-4.530506000	-3.238501000
6	1.270042000	-6.820711000	-2.472873000
1	3.805350000	-4.569629000	-4.711914000
1	3.658966000	-5.492077000	-2.009127000
1	1.939088000	-6.201094000	-4.430929000
1	1.501321000	-4.074654000	-4.118954000
1	0.213344000	-6.798866000	-2.752931000
1	1.630891000	-7.852277000	-2.554650000
1	1.354959000	-6.501716000	-1.429112000
6	-5.578395000	-6.285415000	3.174730000
6	-6.732186000	-5.509213000	3.793146000
8	-7.799712000	-5.311869000	3.146271000
6	-6.103399000	-7.536067000	2.438925000
6	-5.017507000	-8.299651000	1.636010000
6	-4.302088000	-7.371362000	0.671953000
8	-3.205188000	-6.826069000	0.984695000
7	-4.923150000	-7.065374000	-0.485901000
1	-5.068078000	-5.623016000	2.463219000
1	-6.912999000	-7.217902000	1.773160000
1	-6.550424000	-8.228369000	3.163834000
1	-5.486329000	-9.133136000	1.098037000
1	-4.264447000	-8.718880000	2.310352000
1	-4.550476000	-6.279435000	-1.031326000
1	-5.807946000	-7.477208000	-0.737046000
7	-6.555532000	-5.046023000	5.059404000
6	-7.649113000	-4.401674000	5.784991000
6	-8.178081000	-3.092798000	5.191984000
8	-9.344671000	-2.722098000	5.460831000
1	-5.616698000	-5.037470000	5.465291000
1	-8.512609000	-5.070617000	5.835779000
7	-7.315174000	-2.385642000	4.413762000
6	-7.718177000	-1.157099000	3.750613000
6	-7.777157000	-1.258399000	2.226829000
8	-7.766490000	-0.201531000	1.538126000
1	-6.338258000	-2.668414000	4.299923000
1	-8.717933000	-0.897094000	4.116783000
7	-7.885291000	-2.501612000	1.693474000
6	-8.073160000	-2.730505000	0.259891000
6	-6.870190000	-3.346605000	-0.449245000
8	-5.796481000	-2.367127000	-0.572373000
1	-7.920508000	-3.309069000	2.313540000
1	-8.314429000	-1.770278000	-0.201322000
1	-6.508014000	-4.231243000	0.089451000
1	-7.179531000	-3.659984000	-1.454689000
1	-5.104908000	-2.684282000	-1.225575000
6	-9.655109000	4.852654000	-2.976760000
6	-8.932107000	3.634978000	-2.440020000
6	-9.273733000	3.085105000	-1.191425000
6	-7.924729000	3.004947000	-3.191916000
6	-8.636454000	1.934986000	-0.710438000
6	-7.282626000	1.855237000	-2.714348000
6	-7.635039000	1.315153000	-1.471570000
1	-9.031580000	5.411737000	-3.683188000
1	-10.572809000	4.568615000	-3.510223000
1	-10.051346000	3.559132000	-0.596736000
1	-7.648212000	3.412383000	-4.161359000
1	-8.903553000	1.508950000	0.250554000
1	-6.509037000	1.384708000	-3.312994000
1	-7.143946000	0.425638000	-1.089730000
26	-0.334196000	-2.921586000	1.042785000
26	-2.014833000	-2.567350000	-1.655676000
8	0.228593000	-4.989775000	0.794974000
1	1.213963000	-5.027846000	0.720730000
1	-0.214255000	-5.604468000	1.506799000
8	-4.481782000	-2.520733000	3.884518000
1	-4.290512000	-1.668183000	4.360543000
1	-4.411807000	-2.363260000	2.881100000
8	-4.211417000	-1.790299000	1.406230000
1	-4.931472000	-1.959278000	0.706848000
1	-4.063468000	-0.820708000	1.562128000
8	-2.167957000	-3.136022000	0.257917000
1	-3.150802000	-4.347774000	-0.303437000
1	-2.865190000	-2.592611000	0.735744000
8	-1.009830000	-4.105916000	8.723053000
1	-0.605994000	-4.739389000	8.038090000
1	-1.046946000	-4.478709000	9.616560000
8	-3.725522000	-3.442596000	-1.945816000
8	-3.866911000	-4.590968000	-0.987127000
8	-0.501757000	-3.953061000	-1.610273000
1	0.140805000	-4.185178000	-2.336563000
8	-1.046126000	-6.390112000	2.457606000
1	-1.859051000	-6.680734000	1.967847000
1	-1.272869000	-6.142178000	3.387239000
8	-2.559790000	-2.763019000	7.237580000
1	-2.032870000	-3.174168000	8.004571000
1	-2.595284000	-1.768592000	7.198048000
8	-2.019940000	-6.099002000	5.057859000
1	-2.363849000	-7.005149000	5.158043000
1	-2.773868000	-5.382888000	5.231484000
8	-0.102169000	-5.541185000	6.783756000
1	-0.813598000	-5.806001000	6.118045000
1	0.647419000	-5.132636000	6.317112000
8	-3.766800000	-4.352009000	5.564564000
1	-4.044478000	-3.724167000	4.812243000
1	-3.431801000	-3.775883000	6.332978000
1	3.737392000	1.115358000	7.028509000
1	3.689323000	4.575818000	3.601001000
1	-0.172864000	6.390031000	5.934961000
1	-1.291350000	7.380604000	1.301092000
1	-5.243383000	5.483277000	2.392002000
1	-5.167346000	7.066492000	-3.623980000
1	-2.085234000	6.119473000	-7.754658000
1	0.642410000	9.718514000	-5.530448000
1	6.879910000	4.300874000	-4.181875000
1	4.156838000	3.833809000	0.180703000
1	8.345535000	-1.675149000	-2.378883000
1	9.889509000	2.038472000	0.338849000
1	6.302539000	0.739439000	3.487461000
1	9.156070000	-2.734544000	4.134499000
1	4.545169000	-1.544905000	-8.044347000
1	7.176888000	-3.526962000	-4.556926000
1	-7.306828000	-4.211045000	6.807267000
1	-7.039570000	-0.337691000	4.002897000
1	-4.837438000	-6.558087000	3.934355000
1	-8.928387000	-3.405696000	0.128241000
1	8.424055000	-4.931790000	0.580485000
1	-9.949776000	5.534493000	-2.170570000
1	2.003449000	-0.144903000	-4.526052000
1	3.962513000	-6.896656000	-3.049564000
1	-0.397895000	-4.549835000	-0.823905000
1	4.554622000	-2.248466000	3.960966000

^{5}IM2$_{B}$:

6	8.712281000	-2.900966000	2.652744000
6	7.943023000	-1.741638000	2.035739000
8	7.868116000	-1.644904000	0.775400000
6	7.857978000	-4.204410000	2.779861000
6	6.935187000	-4.466496000	1.631000000
7	7.326473000	-4.728804000	0.309735000
6	5.565984000	-4.425022000	1.596387000
6	6.237172000	-4.821617000	-0.493988000
7	5.163446000	-4.642045000	0.285084000
1	9.570215000	-3.084487000	1.998269000
1	8.541932000	-5.047870000	2.938447000
1	7.237769000	-4.137765000	3.679744000
1	4.857899000	-4.248858000	2.385584000
1	6.207213000	-4.980961000	-1.573773000
1	4.142133000	-4.514502000	-0.021164000
7	7.357610000	-0.851497000	2.875218000
6	6.433149000	0.194963000	2.422363000
6	6.901019000	1.035138000	1.263761000
8	6.063869000	1.486433000	0.430858000
1	7.304282000	-1.023532000	3.878212000
1	5.462804000	-0.217751000	2.131751000

7	8.214264000	1.328936000	1.114613000
6	8.651524000	2.130617000	-0.029889000
6	8.153389000	1.590300000	-1.377619000
8	7.888962000	2.387882000	-2.312274000
1	8.882131000	1.002711000	1.796889000
1	8.279944000	3.155922000	0.043080000
7	8.047281000	0.238770000	-1.506738000
6	7.600073000	-0.360903000	-2.758819000
6	6.086701000	-0.368866000	-3.005169000
8	5.655243000	-0.837806000	-4.091459000
1	8.153488000	-0.368879000	-0.693894000
1	8.042607000	0.182481000	-3.598620000
7	5.294105000	0.138261000	-2.033896000
6	3.845112000	0.333507000	-2.217445000
6	3.533399000	1.835615000	-2.115824000
8	3.019603000	2.468648000	-3.081573000
6	2.995046000	-0.496212000	-1.231733000
6	3.255056000	-2.007355000	-1.316902000
6	2.362459000	-2.806540000	-0.375904000
8	1.316792000	-2.238155000	0.082092000
8	2.688379000	-4.038993000	-0.082987000
1	5.716018000	0.490303000	-1.176038000
1	3.622544000	0.046584000	-3.246780000
1	1.937881000	-0.306409000	-1.448599000
1	3.159946000	-0.153849000	-0.203885000
1	4.304337000	-2.234217000	-1.093925000
1	3.072514000	-2.380178000	-2.335661000
7	3.865856000	2.439783000	-0.949547000
6	3.609524000	3.851941000	-0.708767000
6	4.423433000	4.825660000	-1.546738000
8	4.033317000	6.028819000	-1.626174000
1	4.304616000	1.916221000	-0.194446000
1	2.559124000	4.097997000	-0.903884000
7	5.491568000	4.374975000	-2.238065000
6	6.144993000	5.222895000	-3.233791000
6	5.194316000	5.721389000	-4.330589000
8	5.494131000	6.743034000	-4.989289000
1	5.867672000	3.445682000	-2.087251000
1	6.568587000	6.120620000	-2.775514000
7	4.041688000	5.012417000	-4.529267000
6	3.022387000	5.462037000	-5.475390000
6	1.733327000	5.914079000	-4.765820000
8	0.648935000	6.000928000	-5.406411000
6	2.722835000	4.399264000	-6.544439000
1	3.860101000	4.145783000	-4.025421000
1	3.445442000	6.352620000	-5.959935000
1	1.904528000	4.731687000	-7.186699000
1	3.616640000	4.234094000	-7.152822000
1	2.434235000	3.448324000	-6.085520000
7	1.810853000	6.280535000	-3.453068000
6	0.588005000	6.541334000	-2.684300000
6	-0.198933000	7.765098000	-3.166997000
8	-1.420165000	7.865454000	-2.868810000
6	0.854223000	6.640196000	-1.171617000
8	0.629008000	5.318871000	-0.582996000
1	2.690722000	6.196594000	-2.939021000
1	-0.106249000	5.708359000	-2.835160000
1	1.880226000	6.970429000	-0.980992000
1	0.149372000	7.359545000	-0.737866000
1	0.765643000	5.344857000	0.396652000
7	0.453323000	8.686972000	-3.913670000
6	-0.250666000	9.808677000	-4.529358000
6	-1.295974000	9.423715000	-5.587881000
8	-2.127605000	10.286444000	-5.949988000
1	1.429976000	8.528661000	-4.120398000
1	-0.792779000	10.378286000	-3.769853000
7	-1.256440000	8.150188000	-6.070895000
6	-2.283001000	7.648277000	-6.974730000
6	-3.168141000	6.557107000	-6.370010000
8	-3.834614000	5.804963000	-7.125715000
1	-0.528436000	7.494598000	-5.785565000
1	-2.912122000	8.496309000	-7.265180000
7	-3.196155000	6.470001000	-5.010734000
6	-3.995467000	5.462847000	-4.317749000
6	-3.145433000	4.439448000	-3.536441000

6	-2.147967000	3.628160000	-4.336870000
6	-2.363894000	3.285088000	-5.699747000
6	-0.964485000	3.165645000	-3.695420000
6	-1.458204000	2.496164000	-6.380435000
6	-0.041061000	2.383689000	-4.359089000
6	-0.270995000	1.996880000	-5.730913000
8	0.551975000	1.216916000	-6.363357000
1	-2.639082000	7.106338000	-4.447062000
1	-4.616681000	4.976120000	-5.073628000
1	-3.842330000	3.745154000	-3.036419000
1	-2.610239000	4.965204000	-2.734629000
1	-3.225473000	3.679544000	-6.226965000
1	-0.777241000	3.455275000	-2.664509000
1	-1.610397000	2.231547000	-7.420921000
1	0.887132000	2.077097000	-3.888288000
1	-3.561416000	-3.643242000	-3.106426000
6	-4.891319000	4.262884000	2.616639000
6	-3.438802000	4.605981000	2.401068000
8	-2.512284000	3.769718000	2.596416000
6	-5.403512000	3.152827000	1.638483000
6	-6.095426000	1.996561000	2.383578000
6	-5.058361000	1.136574000	3.114175000
8	-4.145230000	0.547173000	2.471491000
8	-5.208470000	1.142979000	4.424287000
1	-4.973289000	3.886021000	3.644316000
1	-6.105864000	3.590042000	0.921317000
1	-4.570901000	2.736081000	1.062959000
1	-6.610662000	1.330559000	1.685783000
1	-6.842049000	2.367938000	3.091018000
7	-3.119416000	5.880385000	2.011893000
6	-1.751768000	6.209314000	1.595866000
6	-0.693323000	5.885600000	2.649106000
8	0.438737000	5.449041000	2.296503000
1	-3.861541000	6.522768000	1.772980000
1	-1.465078000	5.660065000	0.693269000
7	-0.990618000	6.134473000	3.949380000
6	-0.032531000	5.861701000	5.025368000
6	0.138508000	4.370928000	5.370873000
8	1.245770000	3.956952000	5.794208000
1	-1.905513000	6.500992000	4.173213000
1	0.954404000	6.232041000	4.742719000
7	-0.957983000	3.590725000	5.214657000
6	-0.960272000	2.125136000	5.424609000
6	-0.000315000	1.478525000	4.402612000
8	0.958728000	0.750204000	4.769100000
6	-2.419484000	1.654130000	5.279351000
6	-2.742510000	0.296110000	5.879286000
8	-2.013141000	-0.224266000	6.776724000
8	-3.838701000	-0.281809000	5.447656000
1	-1.740169000	3.961643000	4.689166000
1	-0.570545000	1.887777000	6.415619000
1	-2.716814000	1.635009000	4.229984000
1	-3.075338000	2.376596000	5.783529000
7	-0.209534000	1.822312000	3.104615000
6	0.663347000	1.337293000	2.029258000
6	2.106786000	1.831311000	2.227987000
8	3.089084000	1.123179000	1.874702000
6	0.110034000	1.763645000	0.647118000
6	-1.263769000	1.160851000	0.245522000
6	-1.258620000	-0.347452000	0.110957000
8	-0.853634000	-1.020801000	1.122807000
8	-1.668807000	-0.882522000	-1.005508000
1	-1.023295000	2.376836000	2.854182000
1	0.706086000	0.247637000	2.067914000
1	0.857062000	1.482492000	-0.102928000
1	0.025317000	2.857390000	0.609917000
1	-2.032905000	1.414806000	0.986857000
1	-1.570411000	1.592087000	-0.711164000
7	2.269236000	3.060427000	2.788744000
6	3.577618000	3.604314000	3.136402000
6	4.379900000	2.750910000	4.119228000
8	5.624345000	2.631437000	3.957584000
1	1.460849000	3.650102000	2.941006000
1	4.224804000	3.699881000	2.261150000
7	3.716107000	2.159411000	5.140185000

6	4.415903000	1.233492000	6.031250000
6	4.649077000	-0.150806000	5.403731000
8	5.801608000	-0.678373000	5.396241000
1	2.728226000	2.345985000	5.285762000
1	5.399150000	1.633328000	6.283076000
7	3.563264000	-0.765270000	4.883280000
6	3.678843000	-2.083877000	4.247460000
6	2.538660000	-2.352439000	3.261572000
6	1.209669000	-2.581601000	3.911930000
7	0.058748000	-2.811435000	3.153803000
6	0.853867000	-2.573587000	5.247911000
6	-0.960052000	-2.920969000	4.017413000
7	-0.514022000	-2.786431000	5.289398000
1	2.665685000	-0.278886000	4.837197000
1	3.714451000	-2.866478000	5.019789000
1	2.796131000	-3.230324000	2.651984000
1	2.470613000	-1.510923000	2.560435000
1	1.421203000	-2.365311000	6.138439000
1	-1.992181000	-3.073483000	3.756382000
6	0.026273000	-1.665623000	-4.701267000
6	1.126969000	-2.211855000	-5.584472000
8	1.334720000	-3.470880000	-5.675119000
6	-1.326821000	-1.556996000	-5.461184000
6	-1.923431000	-2.890223000	-5.939566000
6	-2.614364000	-3.760574000	-4.907163000
8	-2.970578000	-3.101352000	-3.753127000
8	-2.903061000	-4.943802000	-5.103966000
1	-0.077919000	-2.324714000	-3.834912000
1	-2.041180000	-1.056808000	-4.800651000
1	-1.176206000	-0.905489000	-6.329661000
1	-1.175226000	-3.515697000	-6.437937000
1	-2.691007000	-2.677995000	-6.698575000
7	1.849409000	-1.334569000	-6.309796000
6	2.758834000	-1.762866000	-7.383779000
6	3.780605000	-2.841253000	-7.006975000
8	4.011823000	-3.782763000	-7.797017000
1	1.582761000	-0.345496000	-6.268005000
1	2.194062000	-2.181537000	-8.222051000
7	4.426405000	-2.695055000	-5.811757000
6	5.398150000	-3.672088000	-5.340828000
6	4.884422000	-4.551340000	-4.199594000
8	5.691597000	-5.012907000	-3.336423000
1	4.385192000	-1.829723000	-5.282413000
1	5.662803000	-4.318871000	-6.185486000
7	3.566947000	-4.838607000	-4.200760000
6	2.954997000	-5.756591000	-3.229111000
6	1.549595000	-6.197041000	-3.663192000
8	0.647423000	-5.045969000	-3.685906000
6	0.958958000	-7.227358000	-2.704999000
1	2.967792000	-4.383356000	-4.888486000
1	2.878814000	-5.277509000	-2.245129000
1	1.592622000	-6.613041000	-4.679201000
1	0.766133000	-4.485607000	-4.506977000
1	-0.061768000	-7.471804000	-3.010097000
1	1.555599000	-8.146128000	-2.703785000
1	0.925781000	-6.834696000	-1.682242000
6	-5.335601000	-6.488587000	3.665498000
6	-6.484437000	-5.758701000	4.346122000
8	-7.599133000	-5.627570000	3.764657000
6	-5.835325000	-7.802296000	3.028439000
6	-4.772240000	-8.522682000	2.159551000
6	-4.210834000	-7.594826000	1.097472000
8	-3.096418000	-7.018611000	1.251823000
7	-4.982596000	-7.330032000	0.022978000
1	-4.933217000	-5.827656000	2.886345000
1	-6.721634000	-7.568925000	2.428320000
1	-6.165632000	-8.490781000	3.816823000
1	-5.224076000	-9.409263000	1.696716000
1	-3.934130000	-8.859850000	2.776815000
1	-4.681633000	-6.572703000	-0.600781000
1	-5.874794000	-7.777743000	-0.114081000
7	-6.250932000	-5.265081000	5.590425000
6	-7.325295000	-4.660552000	6.377762000
6	-7.972625000	-3.401389000	5.791252000
8	-9.144555000	-3.102307000	6.119540000
1	-5.290002000	-5.220552000	5.937412000
1	-8.142935000	-5.374526000	6.510120000
7	-7.202582000	-2.657418000	4.952338000
6	-7.720267000	-1.471231000	4.289558000
6	-7.868942000	-1.615197000	2.773746000
8	-7.947679000	-0.579313000	2.057984000
1	-6.214142000	-2.879555000	4.797637000
1	-8.708347000	-1.260210000	4.714130000
7	-7.954374000	-2.879078000	2.286504000
6	-8.183889000	-3.170131000	0.872048000
6	-7.000907000	-3.839263000	0.176990000
8	-5.900133000	-2.901999000	0.028856000
1	-7.912814000	-3.665951000	2.932197000
1	-8.415584000	-2.227242000	0.372145000
1	-6.672832000	-4.720077000	0.745751000
1	-7.321415000	-4.173804000	-0.818654000
1	-5.237324000	-3.197497000	-0.683702000
6	-8.075929000	3.949296000	-3.306539000
6	-7.534400000	2.681192000	-2.680219000
6	-8.111375000	2.147710000	-1.514103000
6	-6.464745000	1.985104000	-3.272329000
6	-7.644794000	0.948773000	-0.958612000
6	-5.991584000	0.788068000	-2.719915000
6	-6.580173000	0.264526000	-1.561643000
1	-8.818208000	3.724023000	-4.084523000
1	-8.569715000	4.584726000	-2.563010000
1	-8.942649000	2.669910000	-1.045908000
1	-6.013522000	2.374910000	-4.182206000
1	-8.098635000	0.540540000	-0.060088000
1	-5.169573000	0.260783000	-3.196252000
1	-6.229872000	-0.668519000	-1.130581000
26	-0.348705000	-2.948109000	1.080372000
26	-2.183683000	-2.786776000	-1.336148000
8	0.233826000	-4.836751000	0.784125000
1	1.213382000	-4.843086000	0.631093000
1	-0.163378000	-5.497663000	1.512545000
8	-4.396145000	-2.695471000	4.354545000
1	-4.215802000	-1.828302000	4.807407000
1	-4.374102000	-2.560129000	3.341924000
8	-4.278957000	-2.090154000	1.856960000
1	-5.022828000	-2.357094000	1.211680000
1	-4.165340000	-1.107938000	1.933732000
8	-2.218771000	-3.405854000	0.649060000
1	-3.167189000	-4.697952000	-0.072687000
1	-2.909674000	-2.902403000	1.167450000
8	-0.347789000	-4.111014000	8.718247000
1	-0.007292000	-4.737388000	7.993722000
1	-0.247925000	-4.470854000	9.612474000
8	-4.010392000	-3.695896000	-1.570982000
8	-3.763729000	-5.012187000	-0.829933000
8	-0.707636000	-4.152001000	-1.665154000
1	-0.236680000	-4.515206000	-2.480530000
8	-0.846254000	-6.327425000	2.457787000
1	-1.670989000	-6.693838000	2.041502000
1	-1.021921000	-6.086862000	3.402218000
8	-2.168473000	-2.859405000	7.468650000
1	-1.516262000	-3.237587000	8.150368000
1	-2.266533000	-1.869660000	7.443312000
8	-1.641170000	-6.119827000	5.119455000
1	-1.922802000	-7.047341000	5.220928000
1	-2.412943000	-5.457179000	5.406711000
8	0.402364000	-5.520896000	6.689741000
1	-0.358208000	-5.801617000	6.088803000
1	1.071687000	-5.049281000	6.163228000
8	-3.426380000	-4.512854000	5.890013000
1	-3.808549000	-3.870448000	5.191958000
1	-3.055710000	-3.940738000	6.641962000
1	3.823339000	1.125720000	6.944409000
1	3.416702000	4.599257000	3.561351000
1	-0.357879000	6.400297000	5.920474000
1	-1.716727000	7.279523000	1.366110000
1	-5.519558000	5.158222000	2.551182000
1	-4.670135000	5.958131000	-3.608285000
1	-1.838363000	7.228761000	-7.881474000
1	0.486993000	10.473372000	-4.987268000

1	6.959916000	4.644111000	-3.676007000
1	3.801838000	4.056429000	0.345982000
1	7.951623000	-1.395249000	-2.802908000
1	9.745454000	2.158535000	-0.033399000
1	6.258897000	0.876312000	3.262927000
1	9.100172000	-2.644016000	3.644032000
1	3.284643000	-0.869505000	-7.736002000
1	6.292391000	-3.159744000	-4.985332000
1	-6.921008000	-4.420626000	7.366485000
1	-7.076255000	-0.608210000	4.479272000
1	-4.517926000	-6.678455000	4.368864000
1	-9.054416000	-3.833648000	0.785676000
1	8.277402000	-4.759075000	-0.023089000
1	-7.282736000	4.537490000	-3.782444000
1	0.296676000	-0.671201000	-4.334408000
1	3.605977000	-6.632642000	-3.127061000
1	-0.448243000	-4.627968000	-0.830651000
1	4.641069000	-2.114859000	3.725879000

^{5}TS3$_{B}$:

6	8.609257000	-2.765093000	2.488892000
6	7.845474000	-1.601747000	1.872856000
8	7.776857000	-1.499683000	0.612694000
6	7.748633000	-4.064907000	2.611405000
6	6.824838000	-4.317598000	1.461246000
7	7.215345000	-4.575303000	0.138941000
6	5.455904000	-4.269006000	1.426139000
6	6.126144000	-4.658545000	-0.665937000
7	5.052719000	-4.477385000	0.113499000
1	9.467598000	-2.950815000	1.835645000
1	8.428302000	-4.912391000	2.766812000
1	7.128603000	-3.998437000	3.511432000
1	4.748291000	-4.093052000	2.215758000
1	6.099275000	-4.812873000	-1.747154000
1	4.031362000	-4.344024000	-0.190822000
7	7.256129000	-0.714822000	2.713201000
6	6.331972000	0.331718000	2.260425000
6	6.802087000	1.178167000	1.107499000
8	5.965641000	1.634752000	0.276688000
1	7.198332000	-0.891292000	3.715021000
1	5.363752000	-0.081756000	1.963832000
7	8.115223000	1.472395000	0.960228000
6	8.552329000	2.279186000	-0.180904000
6	8.053267000	1.744288000	-1.530612000
8	7.786126000	2.545471000	-2.461373000
1	8.783305000	1.141336000	1.639948000
1	8.180739000	3.304105000	-0.103268000
7	7.949517000	0.393132000	-1.665052000
6	7.503632000	-0.203343000	-2.919236000
6	5.990760000	-0.208335000	-3.168566000
8	5.560794000	-0.671374000	-4.257681000
1	8.058281000	-0.217418000	-0.854798000
1	7.948646000	0.340435000	-3.757456000
7	5.196756000	0.294787000	-2.196071000
6	3.749339000	0.496651000	-2.383191000
6	3.441760000	1.999324000	-2.278165000
8	2.920833000	2.633463000	-3.239196000
6	2.892543000	-0.332056000	-1.402368000
6	3.141459000	-1.844773000	-1.494643000
6	2.246552000	-2.640310000	-0.552426000
8	1.194945000	-2.073707000	-0.106254000
8	2.577607000	-3.868439000	-0.245990000
1	5.618174000	0.641903000	-1.336189000
1	3.528266000	0.213608000	-3.413950000
1	1.836895000	-0.134103000	-1.619088000
1	3.059854000	0.004356000	-0.372923000
1	4.189981000	-2.080247000	-1.278071000
1	2.950701000	-2.211822000	-2.514049000
7	3.782940000	2.603040000	-1.113982000
6	3.526910000	4.015239000	-0.872206000
6	4.329231000	4.989164000	-1.721451000
8	3.931154000	6.189395000	-1.804778000
1	4.229193000	2.079660000	-0.363085000
1	2.473982000	4.258708000	-1.056608000
7	5.396632000	4.541596000	-2.416035000
6	6.040394000	5.389722000	-3.418004000
6	5.082660000	5.878217000	-4.513411000
8	5.374862000	6.898468000	-5.177737000
1	5.780061000	3.615511000	-2.262616000
1	6.460461000	6.291803000	-2.965080000
7	3.933758000	5.161758000	-4.704862000
6	2.908973000	5.599593000	-5.650681000
6	1.619862000	6.049060000	-4.939654000
8	0.532977000	6.126866000	-5.577087000
6	2.611990000	4.527902000	-6.711536000
1	3.758199000	4.297412000	-4.195066000
1	3.325295000	6.489385000	-6.142414000
1	1.789755000	4.851447000	-7.353316000
1	3.504732000	4.363997000	-7.321840000
1	2.330296000	3.578340000	-6.245620000
7	1.699906000	6.423169000	-3.629118000
6	0.478326000	6.681223000	-2.857360000
6	-0.316553000	7.898869000	-3.342683000
8	-1.536879000	7.995284000	-3.039525000
6	0.749610000	6.788319000	-1.346157000
8	0.529657000	5.469123000	-0.750123000
1	2.581862000	6.345820000	-3.117822000
1	-0.211993000	5.844000000	-3.002157000
1	1.775521000	7.121705000	-1.160650000
1	0.044523000	7.507909000	-0.913200000
1	0.670453000	5.499446000	0.228667000
7	0.328627000	8.820242000	-4.096155000
6	-0.382354000	9.936364000	-4.713805000
6	-1.428887000	9.543154000	-5.767973000
8	-2.264230000	10.401638000	-6.131780000
1	1.304926000	8.664674000	-4.306656000
1	-0.924772000	10.506154000	-3.954646000
7	-1.386576000	8.267623000	-6.245370000
6	-2.412910000	7.759427000	-7.145938000
6	-3.299666000	6.673738000	-6.533890000
8	-3.968198000	5.918427000	-7.284425000
1	-0.655877000	7.615449000	-5.959057000
1	-3.041391000	8.605496000	-7.443336000
7	-3.327150000	6.594997000	-5.173946000
6	-4.128260000	5.593069000	-4.475533000
6	-3.279813000	4.577456000	-3.682389000
6	-2.272929000	3.767069000	-4.472038000
6	-2.480556000	3.410943000	-5.832721000
6	-1.088002000	3.320712000	-3.822387000
6	-1.563597000	2.627289000	-6.504539000
6	-0.153449000	2.544281000	-4.476985000
6	-0.372818000	2.146839000	-5.847344000
8	0.463971000	1.375752000	-6.471904000
1	-2.767152000	7.232443000	-4.614539000
1	-4.745327000	5.099356000	-5.230305000
1	-3.977411000	3.883350000	-3.183107000
1	-2.751958000	5.110275000	-2.880343000
1	-3.345044000	3.791129000	-6.365593000
1	-0.908043000	3.619457000	-2.792755000
1	-1.709287000	2.352741000	-7.543352000
1	0.776113000	2.250316000	-4.001130000
1	-3.807014000	-3.483588000	-2.880690000
6	-4.981714000	4.398410000	2.432673000
6	-3.530768000	4.750550000	2.222818000
8	-2.601588000	3.915914000	2.413427000
6	-5.485310000	3.300084000	1.437220000
6	-6.190410000	2.137839000	2.160974000
6	-5.165820000	1.269746000	2.899428000
8	-4.265724000	0.655689000	2.262560000
8	-5.308575000	1.300993000	4.210611000
1	-5.062767000	4.005397000	3.454295000
1	-6.177884000	3.746400000	0.716055000
1	-4.646588000	2.887443000	0.867459000
1	-6.694567000	1.481117000	1.447420000
1	-6.948411000	2.503088000	2.859043000
7	-3.215070000	6.028159000	1.842308000
6	-1.847709000	6.363157000	1.429384000
6	-0.789920000	6.036390000	2.482455000
8	0.341941000	5.599347000	2.130141000
1	-3.958851000	6.669927000	1.606862000

1	-1.558695000	5.818908000	0.524485000
7	-1.088181000	6.282414000	3.783222000
6	-0.130982000	6.005908000	4.859086000
6	0.034993000	4.514079000	5.202291000
8	1.140108000	4.094962000	5.625897000
1	-2.002256000	6.650853000	4.007308000
1	0.857050000	6.373625000	4.576890000
7	-1.064388000	3.738490000	5.043281000
6	-1.073897000	2.272721000	5.249721000
6	-0.113866000	1.623158000	4.229631000
8	0.839981000	0.889188000	4.598512000
6	-2.534972000	1.809209000	5.098372000
6	-2.863022000	0.446790000	5.685493000
8	-2.142525000	-0.075377000	6.589096000
8	-3.952359000	-0.131488000	5.237937000
1	-1.844988000	4.114927000	4.519771000
1	-0.688917000	2.030932000	6.241525000
1	-2.831038000	1.803235000	4.048768000
1	-3.187899000	2.529495000	5.609678000
7	-0.317394000	1.969864000	2.931815000
6	0.556759000	1.484523000	1.857719000
6	2.002109000	1.970664000	2.063988000
8	2.982237000	1.256197000	1.718674000
6	0.010588000	1.921684000	0.476411000
6	-1.364024000	1.324740000	0.067888000
6	-1.356490000	-0.181663000	-0.083025000
8	-0.991546000	-0.867691000	0.935132000
8	-1.719959000	-0.700997000	-1.222872000
1	-1.126250000	2.531088000	2.680198000
1	0.594306000	0.394364000	1.892035000
1	0.758897000	1.643966000	-0.273572000
1	-0.071816000	3.015704000	0.446236000
1	-2.132210000	1.571011000	0.812161000
1	-1.668092000	1.765627000	-0.885009000
7	2.167444000	3.200660000	2.622301000
6	3.476767000	3.739474000	2.974507000
6	4.272900000	2.882696000	3.959411000
8	5.517535000	2.759935000	3.802206000
1	1.361210000	3.794665000	2.769433000
1	4.126872000	3.833047000	2.101171000
7	3.603886000	2.291151000	4.977024000
6	4.297264000	1.358356000	5.866029000
6	4.527691000	-0.023517000	5.231831000
8	5.677701000	-0.556359000	5.227008000
1	2.616681000	2.482239000	5.121219000
1	5.281012000	1.753263000	6.123655000
7	3.441577000	-0.630146000	4.702734000
6	3.554612000	-1.944038000	4.056810000
6	2.414882000	-2.202587000	3.067301000
6	1.084500000	-2.430066000	3.715512000
7	-0.069431000	-2.652045000	2.958824000
6	0.730589000	-2.425532000	5.051921000
6	-1.087149000	-2.760295000	3.824067000
7	-0.637885000	-2.632674000	5.095469000
1	2.546671000	-0.139037000	4.655115000
1	3.587536000	-2.732723000	4.823099000
1	2.670257000	-3.077580000	2.453052000
1	2.351505000	-1.356479000	2.371357000
1	1.300488000	-2.221572000	5.941758000
1	-2.120784000	-2.905992000	3.565056000
6	-0.004990000	-1.514971000	-4.754108000
6	1.056446000	-2.059121000	-5.684441000
8	1.261049000	-3.317213000	-5.792986000
6	-1.390164000	-1.396845000	-5.453464000
6	-2.017030000	-2.724304000	-5.903882000
6	-2.728457000	-3.565198000	-4.848851000
8	-2.983413000	-2.957558000	-3.660319000
8	-3.121555000	-4.712465000	-5.103371000
1	-0.075939000	-2.180533000	-3.888905000
1	-2.070317000	-0.895268000	-4.757954000
1	-1.273255000	-0.741638000	-6.324543000
1	-1.284422000	-3.372783000	-6.397212000
1	-2.783244000	-2.512418000	-6.663706000
7	1.755580000	-1.179567000	-6.430657000
6	2.630504000	-1.610557000	-7.531236000
6	3.676052000	-2.673317000	-7.175459000
8	3.910000000	-3.608263000	-7.972495000
1	1.489850000	-0.190867000	-6.381870000
1	2.041866000	-2.046126000	-8.344042000
7	4.336005000	-2.522557000	-5.988513000
6	5.321237000	-3.491189000	-5.528886000
6	4.826242000	-4.373383000	-4.381415000
8	5.645726000	-4.825714000	-3.524508000
1	4.286294000	-1.661340000	-5.453617000
1	5.580693000	-4.136199000	-6.376490000
7	3.511606000	-4.673225000	-4.370345000
6	2.917394000	-5.598979000	-3.395415000
6	1.523740000	-6.075127000	-3.831227000
8	0.590219000	-4.950983000	-3.849150000
6	0.961271000	-7.123304000	-2.875328000
1	2.900560000	-4.221095000	-5.050014000
1	2.824557000	-5.116483000	-2.414295000
1	1.578519000	-6.487555000	-4.848136000
1	0.701190000	-4.370708000	-4.657854000
1	-0.053570000	-7.391935000	-3.179656000
1	1.580714000	-8.026885000	-2.877592000
1	0.919580000	-6.733774000	-1.851551000
6	-5.439109000	-6.289043000	3.460511000
6	-6.588118000	-5.577146000	4.159207000
8	-7.706782000	-5.443643000	3.585390000
6	-5.928328000	-7.609641000	2.829520000
6	-4.863081000	-8.315990000	1.952357000
6	-4.323395000	-7.386032000	0.880805000
8	-3.203631000	-6.813421000	1.008159000
7	-5.117720000	-7.120118000	-0.177007000
1	-5.059800000	-5.621364000	2.675521000
1	-6.823295000	-7.388789000	2.237476000
1	-6.241962000	-8.301682000	3.621595000
1	-5.305797000	-9.210559000	1.495982000
1	-4.014531000	-8.639653000	2.562507000
1	-4.821281000	-6.374359000	-0.814468000
1	-6.009388000	-7.572404000	-0.301095000
7	-6.350624000	-5.103542000	5.410240000
6	-7.421647000	-4.515301000	6.213676000
6	-8.063084000	-3.237744000	5.661651000
8	-9.225263000	-2.930927000	6.016283000
1	-5.387599000	-5.059299000	5.752193000
1	-8.242490000	-5.228899000	6.327033000
7	-7.300717000	-2.489354000	4.819473000
6	-7.820314000	-1.291402000	4.179261000
6	-8.013761000	-1.425835000	2.667557000
8	-8.098450000	-0.386284000	1.958081000
1	-6.320008000	-2.722708000	4.637843000
1	-8.793089000	-1.069872000	4.632652000
7	-8.128543000	-2.687250000	2.179100000
6	-8.397796000	-2.971277000	0.771151000
6	-7.247414000	-3.673086000	0.054906000
8	-6.122678000	-2.764583000	-0.104681000
1	-8.073171000	-3.478144000	2.818907000
1	-8.613741000	-2.022404000	0.275663000
1	-6.936381000	-4.563226000	0.618571000
1	-7.592087000	-3.994127000	-0.936924000
1	-5.495778000	-3.051369000	-0.838965000
6	-8.225581000	4.113362000	-3.455337000
6	-7.700870000	2.839862000	-2.825771000
6	-8.283620000	2.318125000	-1.657243000
6	-6.641405000	2.127553000	-3.417047000
6	-7.832544000	1.114756000	-1.098491000
6	-6.183930000	0.925949000	-2.861703000
6	-6.778178000	0.414383000	-1.700929000
1	-8.968441000	3.895575000	-4.234855000
1	-8.713578000	4.755805000	-2.714049000
1	-9.106701000	2.853352000	-1.189417000
1	-6.185572000	2.508673000	-4.328202000
1	-8.289398000	0.717318000	-0.196546000
1	-5.369737000	0.386256000	-3.337522000
1	-6.438662000	-0.521063000	-1.266712000
26	-0.477602000	-2.791380000	0.894557000
26	-2.188498000	-2.594291000	-1.635869000
8	0.131770000	-4.674954000	0.633243000

1	1.111894000	-4.672509000	0.483671000
1	-0.269738000	-5.336524000	1.357874000
8	-4.499871000	-2.547905000	4.148649000
1	-4.320948000	-1.674612000	4.590884000
1	-4.473344000	-2.428460000	3.135441000
8	-4.381078000	-1.974605000	1.634752000
1	-5.162952000	-2.230818000	1.037405000
1	-4.267341000	-0.991959000	1.715603000
8	-2.325835000	-3.251578000	0.344060000
1	-3.235886000	-4.488742000	-0.362274000
1	-3.027651000	-2.758252000	0.858003000
8	-0.459636000	-3.947286000	8.530468000
1	-0.117401000	-4.574345000	7.807660000
1	-0.352056000	-4.300626000	9.426336000
8	-4.233534000	-3.508530000	-1.755854000
8	-3.855733000	-4.830746000	-1.102152000
8	-0.745537000	-4.018782000	-1.836390000
1	-0.284627000	-4.413248000	-2.643310000
8	-0.979580000	-6.162254000	2.285850000
1	-1.803012000	-6.511574000	1.851615000
1	-1.156808000	-5.942285000	3.235141000
8	-2.283660000	-2.703788000	7.281072000
1	-1.629575000	-3.079157000	7.962946000
1	-2.385466000	-1.714167000	7.253722000
8	-1.762982000	-5.974956000	4.946933000
1	-2.043707000	-6.900346000	5.068748000
1	-2.530450000	-5.305587000	5.232045000
8	0.289727000	-5.362596000	6.505147000
1	-0.471980000	-5.646869000	5.908168000
1	0.958123000	-4.894373000	5.974407000
8	-3.538105000	-4.353827000	5.706196000
1	-3.916148000	-3.715152000	5.002850000
1	-3.167920000	-3.778564000	6.457356000
1	3.700824000	1.247989000	6.776332000
1	3.318145000	4.734846000	3.399346000
1	-0.454786000	6.544375000	5.754823000
1	-1.815443000	7.434591000	1.205079000
1	-5.614801000	5.291056000	2.379075000
1	-4.806872000	6.093221000	-3.773158000
1	-1.967576000	7.332709000	-8.048944000
1	0.350805000	10.602883000	-5.176261000
1	6.856957000	4.814392000	-3.861799000
1	3.731059000	4.221827000	0.180054000
1	7.853434000	-1.238254000	-2.964213000
1	9.646254000	2.306872000	-0.184626000
1	6.152304000	1.009581000	3.102632000
1	8.996239000	-2.511852000	3.481496000
1	3.134200000	-0.716484000	-7.912634000
1	6.215610000	-2.972263000	-5.183571000
1	-7.016771000	-4.303929000	7.208569000
1	-7.159130000	-0.438733000	4.355490000
1	-4.607689000	-6.467679000	4.150400000
1	-9.287538000	-3.610962000	0.702006000
1	8.166152000	-4.607715000	-0.194111000
1	-7.424238000	4.691179000	-3.930270000
1	0.286437000	-0.523949000	-4.393145000
1	3.588987000	-6.458286000	-3.286284000
1	-0.513184000	-4.485811000	-0.989544000
1	4.517311000	-1.973398000	3.536010000

^{5}Pr$_B$:

6	8.618298000	-2.648171000	2.202997000
6	7.812969000	-1.510325000	1.591948000
8	7.703334000	-1.435790000	0.332155000
6	7.803610000	-3.978450000	2.322644000
6	6.858701000	-4.237905000	1.191325000
7	7.222563000	-4.425784000	-0.149926000
6	5.488574000	-4.249764000	1.195143000
6	6.117096000	-4.520851000	-0.929395000
7	5.059060000	-4.421837000	-0.114498000
1	9.480100000	-2.802769000	1.545960000
1	8.513832000	-4.806116000	2.444229000
1	7.205011000	-3.948368000	3.238892000
1	4.795754000	-4.137316000	2.009457000
1	6.069896000	-4.618971000	-2.016647000
1	4.029599000	-4.319476000	-0.384750000
7	7.237309000	-0.617707000	2.434101000
6	6.286840000	0.411427000	1.994062000
6	6.710654000	1.248418000	0.815175000
8	5.838621000	1.706106000	0.021278000
1	7.215136000	-0.775878000	3.441787000
1	5.314336000	-0.017614000	1.736446000
7	8.020007000	1.527010000	0.614428000
6	8.431841000	2.307005000	-0.552843000
6	7.876167000	1.768068000	-1.878536000
8	7.614463000	2.566239000	-2.812482000
1	8.707920000	1.188273000	1.270061000
1	8.087000000	3.341474000	-0.476974000
7	7.713103000	0.420338000	-1.990544000
6	7.197620000	-0.168515000	-3.222090000
6	5.675180000	-0.132527000	-3.410900000
8	5.188873000	-0.567638000	-4.487381000
1	7.842886000	-0.185983000	-1.180069000
1	7.624532000	0.357550000	-4.080486000
7	4.930884000	0.360583000	-2.393912000
6	3.471818000	0.544488000	-2.500565000
6	3.156842000	2.041242000	-2.341560000
8	2.693750000	2.727720000	-3.295810000
6	2.686013000	-0.318046000	-1.489872000
6	2.921435000	-1.827807000	-1.645344000
6	2.189575000	-2.639869000	-0.586981000
8	1.224864000	-2.080522000	0.037203000
8	2.554746000	-3.873488000	-0.363142000
1	5.392460000	0.703642000	-1.552579000
1	3.202790000	0.279445000	-3.524636000
1	1.617974000	-0.109060000	-1.617960000
1	2.929220000	-0.016795000	-0.464572000
1	3.990961000	-2.065636000	-1.604559000
1	2.564810000	-2.186685000	-2.622398000
7	3.441677000	2.583493000	-1.131659000
6	3.180436000	3.981097000	-0.824802000
6	4.088977000	4.990136000	-1.509773000
8	3.760342000	6.213639000	-1.499791000
1	3.863813000	2.025849000	-0.392988000
1	2.159129000	4.259823000	-1.106160000
7	5.172784000	4.544618000	-2.180552000
6	5.922888000	5.430395000	-3.069507000
6	5.053776000	6.064745000	-4.161654000
8	5.422311000	7.127637000	-4.710606000
1	5.468016000	3.579375000	-2.102953000
1	6.365742000	6.264123000	-2.518164000
7	3.886777000	5.423999000	-4.478875000
6	2.933284000	6.000867000	-5.424250000
6	1.607317000	6.374746000	-4.738765000
8	0.546075000	6.484561000	-5.412640000
6	2.697806000	5.090575000	-6.638520000
1	3.653882000	4.520905000	-4.070718000
1	3.393992000	6.939510000	-5.762593000
1	1.934843000	5.520074000	-7.291141000
1	3.631992000	4.980101000	-7.196251000
1	2.359086000	4.098144000	-6.325645000
7	1.627570000	6.653703000	-3.402497000
6	0.373756000	6.868315000	-2.667844000
6	-0.448251000	8.049689000	-3.198356000
8	-1.696677000	8.058275000	-3.035642000
6	0.612119000	7.026184000	-1.153372000
8	0.450028000	5.714758000	-0.529043000
1	2.483607000	6.528785000	-2.856864000
1	-0.282499000	6.001357000	-2.800170000
1	1.615140000	7.420387000	-0.961846000
1	-0.136665000	7.719169000	-0.749884000
1	0.688693000	5.741727000	0.429138000
7	0.220194000	9.060320000	-3.807602000
6	-0.481312000	10.198775000	-4.395839000
6	-1.397810000	9.879992000	-5.587530000
8	-2.211299000	10.754757000	-5.962983000
1	1.219041000	8.966735000	-3.930323000
1	-1.124531000	10.670537000	-3.647837000
7	-1.270560000	8.654837000	-6.167940000
6	-2.180048000	8.210292000	-7.216808000

6	-3.048703000	7.014426000	-6.821961000
8	-3.604018000	6.325309000	-7.715032000
1	-0.566682000	7.987955000	-5.851152000
1	-2.828051000	9.055394000	-7.471823000
7	-3.184835000	6.771172000	-5.489663000
6	-3.960909000	5.644016000	-4.979008000
6	-3.091611000	4.565896000	-4.301281000
6	-2.072949000	3.851466000	-5.164796000
6	-2.277302000	3.607091000	-6.551066000
6	-0.887235000	3.353452000	-4.555863000
6	-1.372553000	2.856720000	-7.277231000
6	0.034662000	2.610366000	-5.262714000
6	-0.197726000	2.296253000	-6.653682000
8	0.604250000	1.522055000	-7.319076000
1	-2.714000000	7.366287000	-4.814221000
1	-4.527586000	5.236696000	-5.819446000
1	-3.772348000	3.808518000	-3.875887000
1	-2.575828000	5.019675000	-3.444773000
1	-3.130201000	4.046561000	-7.055874000
1	-0.704505000	3.570063000	-3.507193000
1	-1.522715000	2.657986000	-8.332586000
1	0.953140000	2.271846000	-4.794191000
1	-3.704927000	-4.423672000	-2.449138000
6	-4.868679000	4.406766000	2.773190000
6	-3.426923000	4.796326000	2.573850000
8	-2.485457000	3.963252000	2.699738000
6	-5.370100000	3.402371000	1.681576000
6	-6.096401000	2.191401000	2.300554000
6	-5.088576000	1.268596000	2.999323000
8	-4.284980000	0.565264000	2.330875000
8	-5.134076000	1.363332000	4.316484000
1	-4.928207000	3.912159000	3.750820000
1	-6.049735000	3.918364000	0.995028000
1	-4.529579000	3.033012000	1.084933000
1	-6.591539000	1.586834000	1.536279000
1	-6.859643000	2.519653000	3.012140000
7	-3.133718000	6.098484000	2.266671000
6	-1.783233000	6.474060000	1.832037000
6	-0.685207000	6.100080000	2.826442000
8	0.437605000	5.703966000	2.405989000
1	-3.891502000	6.740828000	2.082883000
1	-1.518673000	5.987068000	0.887480000
7	-0.940788000	6.262821000	4.150464000
6	0.055175000	5.935373000	5.175783000
6	0.223572000	4.429049000	5.449964000
8	1.345391000	3.982564000	5.794963000
1	-1.850516000	6.606306000	4.426197000
1	1.033806000	6.308110000	4.868946000
7	-0.890599000	3.670381000	5.319776000
6	-0.909928000	2.195052000	5.453751000
6	0.011655000	1.587524000	4.372824000
8	0.956957000	0.812346000	4.670293000
6	-2.383464000	1.765760000	5.317198000
6	-2.731910000	0.369715000	5.806697000
8	-2.029606000	-0.222491000	6.679728000
8	-3.832995000	-0.152408000	5.318052000
1	-1.695732000	4.081569000	4.864797000
1	-0.501933000	1.898279000	6.421216000
1	-2.714008000	1.855072000	4.282577000
1	-3.003442000	2.456102000	5.906083000
7	-0.227787000	2.012851000	3.105611000
6	0.583866000	1.565676000	1.969599000
6	2.034082000	2.056534000	2.102388000
8	2.997991000	1.361377000	1.677505000
6	-0.058490000	2.031024000	0.638379000
6	-1.481839000	1.481894000	0.352230000
6	-1.535649000	-0.024945000	0.207982000
8	-1.052622000	-0.734523000	1.158091000
8	-2.065110000	-0.514003000	-0.878136000
1	-1.032290000	2.604042000	2.916085000
1	0.630062000	0.475946000	1.972283000
1	0.614317000	1.730728000	-0.172076000
1	-0.105414000	3.127823000	0.618899000
1	-2.172640000	1.754069000	1.158502000
1	-1.856047000	1.933039000	-0.570355000

7	2.225227000	3.271311000	2.685761000
6	3.549799000	3.807148000	2.983190000
6	4.388995000	2.930094000	3.912368000
8	5.623118000	2.802435000	3.691484000
1	1.426370000	3.857608000	2.892463000
1	4.157547000	3.919783000	2.081522000
7	3.766595000	2.321771000	4.950003000
6	4.502404000	1.379174000	5.793511000
6	4.706844000	0.004200000	5.136256000
8	5.863785000	-0.505746000	5.041855000
1	2.793978000	2.530023000	5.159040000
1	5.497132000	1.772584000	6.008177000
7	3.596605000	-0.627459000	4.692437000
6	3.696274000	-1.946488000	4.053463000
6	2.506741000	-2.243657000	3.137341000
6	1.226853000	-2.536587000	3.858533000
7	0.039517000	-2.773360000	3.157333000
6	0.944145000	-2.604322000	5.207531000
6	-0.925072000	-2.964021000	4.072654000
7	-0.408894000	-2.875960000	5.317103000
1	2.689314000	-0.156782000	4.705060000
1	3.795958000	-2.725750000	4.823038000
1	2.762754000	-3.102711000	2.500302000
1	2.366714000	-1.397455000	2.453001000
1	1.564871000	-2.443383000	6.071023000
1	-1.967388000	-3.135837000	3.868881000
6	0.027770000	-1.110093000	-5.296661000
6	1.024977000	-1.856540000	-6.145013000
8	1.192640000	-3.120612000	-6.024023000
6	-1.425726000	-1.191924000	-5.853493000
6	-2.136344000	-2.535891000	-5.624643000
6	-2.477893000	-2.834812000	-4.170236000
8	-2.010236000	-1.986742000	-3.281245000
8	-3.165899000	-3.863261000	-3.884560000
1	0.031049000	-1.522440000	-4.285819000
1	-2.001078000	-0.401270000	-5.361757000
1	-1.412155000	-0.962842000	-6.926784000
1	-1.529426000	-3.372061000	-5.997551000
1	-3.074316000	-2.569473000	-6.192560000
7	1.717657000	-1.145273000	-7.059442000
6	2.543280000	-1.777894000	-8.093883000
6	3.649106000	-2.717692000	-7.605909000
8	3.962827000	-3.709183000	-8.303211000
1	1.501281000	-0.147021000	-7.148153000
1	1.925627000	-2.385045000	-8.762569000
7	4.270938000	-2.408808000	-6.431287000
6	5.306412000	-3.261721000	-5.867176000
6	4.834792000	-4.100461000	-4.679657000
8	5.667048000	-4.485317000	-3.802361000
1	4.096380000	-1.540190000	-5.937829000
1	5.647609000	-3.943160000	-6.654855000
7	3.531723000	-4.444253000	-4.657633000
6	2.972389000	-5.353167000	-3.649411000
6	1.493727000	-5.658200000	-3.918210000
8	0.706655000	-4.429670000	-3.790336000
6	0.941331000	-6.680034000	-2.928385000
1	2.904895000	-4.039912000	-5.352426000
1	3.050840000	-4.909774000	-2.650380000
1	1.375553000	-6.031668000	-4.944969000
1	0.740187000	-3.891251000	-4.636622000
1	-0.125269000	-6.838150000	-3.109469000
1	1.458757000	-7.640012000	-3.032201000
1	1.067343000	-6.328050000	-1.898525000
6	-5.851991000	-6.316433000	3.934806000
6	-6.937308000	-5.412889000	4.499899000
8	-8.042080000	-5.258671000	3.906327000
6	-6.446221000	-7.527456000	3.194883000
6	-5.361479000	-8.504036000	2.668500000
6	-4.285624000	-7.769428000	1.892499000
8	-3.177480000	-7.488579000	2.456052000
7	-4.581620000	-7.368421000	0.646007000
1	-5.243360000	-5.718218000	3.242137000
1	-7.073634000	-7.167039000	2.372479000
1	-7.113787000	-8.080752000	3.866082000
1	-5.832102000	-9.271049000	2.040796000

1	-4.870671000	-9.010073000	3.504397000
1	-3.985997000	-6.701678000	0.126561000
1	-5.464612000	-7.620363000	0.227294000
7	-6.635848000	-4.785779000	5.667547000
6	-7.634569000	-3.998183000	6.386069000
6	-8.189749000	-2.778521000	5.646663000
8	-9.330650000	-2.345724000	5.933017000
1	-5.658499000	-4.779681000	5.976509000
1	-8.503983000	-4.616818000	6.625759000
7	-7.373534000	-2.210299000	4.717951000
6	-7.807259000	-1.076889000	3.919989000
6	-7.867548000	-1.365853000	2.421004000
8	-7.781930000	-0.415232000	1.596074000
1	-6.403925000	-2.515346000	4.591012000
1	-8.810730000	-0.800442000	4.264735000
7	-8.065180000	-2.660601000	2.061468000
6	-8.258812000	-3.074342000	0.672317000
6	-7.174960000	-4.012395000	0.148803000
8	-5.929258000	-3.291765000	-0.043019000
1	-8.144111000	-3.370868000	2.787912000
1	-8.284599000	-2.171364000	0.058629000
1	-7.027128000	-4.846330000	0.850304000
1	-7.501596000	-4.424218000	-0.814735000
1	-5.321944000	-3.752113000	-0.689738000
6	-7.379400000	2.219210000	-5.014351000
6	-6.726980000	1.614666000	-3.788646000
6	-7.483530000	1.326719000	-2.638858000
6	-5.357673000	1.292415000	-3.783755000
6	-6.895962000	0.727552000	-1.517713000
6	-4.758506000	0.699672000	-2.664022000
6	-5.529169000	0.417610000	-1.528781000
1	-7.815818000	1.442616000	-5.657722000
1	-8.189516000	2.905169000	-4.742020000
1	-8.544287000	1.566352000	-2.627184000
1	-4.761041000	1.494214000	-4.670953000
1	-7.493336000	0.486005000	-0.643099000
1	-3.704219000	0.441901000	-2.671176000
1	-5.069152000	-0.046169000	-0.663725000
26	-0.430461000	-2.756589000	1.134544000
26	-1.933507000	-2.400110000	-1.416679000
8	0.171998000	-4.763685000	0.584417000
1	1.143376000	-4.724200000	0.388059000
1	-0.161781000	-5.431983000	1.308100000
8	-4.566291000	-2.446634000	4.090974000
1	-4.306612000	-1.589850000	4.522066000
1	-4.484080000	-2.378361000	3.085208000
8	-4.357374000	-2.012241000	1.521005000
1	-5.107356000	-2.400563000	0.952959000
1	-4.353244000	-1.023912000	1.593589000
8	-2.269025000	-3.139905000	0.346699000
1	-2.829099000	-4.525973000	-0.168454000
1	-3.005547000	-2.654262000	0.846674000
8	-1.340992000	-4.689006000	8.786842000
1	-1.120035000	-5.406669000	8.094610000
1	-1.715371000	-5.069571000	9.596579000
8	-4.218112000	-4.643969000	-1.588627000
8	-3.227106000	-5.330321000	-0.679012000
8	-0.821457000	-3.953012000	-1.743797000
1	-0.282095000	-4.142634000	-2.574916000
8	-0.825365000	-6.229541000	2.349214000
1	-1.613716000	-6.772061000	2.092817000
1	-1.005143000	-5.913010000	3.266117000
8	-2.230784000	-2.896215000	7.262657000
1	-1.907949000	-3.443453000	8.060967000
1	-2.300275000	-1.908198000	7.318820000
8	-2.209762000	-6.147272000	4.769354000
1	-2.653883000	-6.830911000	4.217651000
1	-2.870221000	-5.416780000	5.076888000
8	-0.828614000	-6.414166000	6.949970000
1	-1.367907000	-6.410122000	6.087945000
1	0.084940000	-6.699383000	6.790436000
8	-3.773203000	-4.316418000	5.674157000
1	-4.099683000	-3.641564000	4.985681000
1	-3.270296000	-3.807321000	6.389128000
1	3.949453000	1.257888000	6.729500000
1	3.410315000	4.794688000	3.432322000
1	-0.228054000	6.438426000	6.105331000
1	-1.769889000	7.557555000	1.672524000
1	-5.515741000	5.289953000	2.813453000
1	-4.685096000	6.010472000	-4.241614000
1	-1.632524000	7.919506000	-8.118031000
1	0.260702000	10.936114000	-4.714337000
1	6.727755000	4.840246000	-3.514865000
1	3.258065000	4.114099000	0.254855000
1	7.511398000	-1.215143000	-3.274217000
1	9.525362000	2.311355000	-0.595408000
1	6.130658000	1.099193000	2.833384000
1	9.000323000	-2.385334000	3.194750000
1	2.993879000	-0.975086000	-8.686258000
1	6.142834000	-2.651518000	-5.528221000
1	-7.185901000	-3.665785000	7.327923000
1	-7.148213000	-0.219075000	4.072904000
1	-5.180528000	-6.641797000	4.736225000
1	-9.227175000	-3.584901000	0.584772000
1	8.162945000	-4.383080000	-0.510210000
1	-6.657451000	2.774286000	-5.623688000
1	0.317906000	-0.059809000	-5.232698000
1	3.551471000	-6.285334000	-3.650804000
1	-0.557059000	-4.491173000	-0.947164000
1	4.624399000	-1.960297000	3.472901000

^{3}Re$_B$:

6	8.749537000	-2.935030000	3.217854000
6	8.042161000	-1.827111000	2.449119000
8	8.084548000	-1.810492000	1.184065000
6	7.821808000	-4.157586000	3.506929000
6	6.968456000	-4.569142000	2.348350000
7	7.445948000	-5.008592000	1.104507000
6	5.605749000	-4.521775000	2.210491000
6	6.412797000	-5.198564000	0.245583000
7	5.290411000	-4.906095000	0.913050000
1	9.600929000	-3.246606000	2.605263000
1	8.447438000	-4.989681000	3.854800000
1	7.148145000	-3.908272000	4.333535000
1	4.849023000	-4.238709000	2.918447000
1	6.451391000	-5.499258000	-0.801488000
1	4.297896000	-4.797815000	0.509473000
7	7.371825000	-0.898334000	3.176648000
6	6.456032000	0.083794000	2.588049000
6	6.983612000	0.884116000	1.429226000
8	6.191768000	1.274724000	0.523431000
1	7.226083000	-1.017914000	4.179111000
1	5.528885000	-0.382585000	2.242639000
7	8.294619000	1.209399000	1.353900000
6	8.785998000	1.974323000	0.205563000
6	8.399685000	1.355029000	-1.144747000
8	8.184056000	2.092674000	-2.137142000
1	8.923149000	0.929839000	2.092090000
1	8.377759000	2.988087000	0.204321000
7	8.337113000	-0.006512000	-1.206200000
6	7.989077000	-0.678839000	-2.451217000
6	6.493925000	-0.748200000	-2.785615000
8	6.143558000	-1.287120000	-3.870920000
1	8.408733000	-0.568603000	-0.357964000
1	8.465689000	-0.159473000	-3.287707000
7	5.634100000	-0.217115000	-1.890927000
6	4.188163000	-0.092746000	-2.143441000
6	3.803152000	1.397127000	-2.183255000
8	3.277930000	1.909138000	-3.211337000
6	3.326975000	-0.853045000	-1.114463000
6	3.608357000	-2.361038000	-1.051546000
6	2.607518000	-3.093352000	-0.168441000
8	1.529467000	-2.481667000	0.123879000
8	2.874186000	-4.306813000	0.245344000
1	6.000182000	0.197462000	-1.035180000
1	4.016879000	-0.481653000	-3.148376000
1	2.274943000	-0.703494000	-1.380007000
1	3.445034000	-0.410073000	-0.118871000
1	4.625245000	-2.556918000	-0.690701000
1	3.535238000	-2.817719000	-2.048475000

7	4.080558000	2.102656000	-1.059041000
6	3.736614000	3.505749000	-0.880883000
6	4.483417000	4.488340000	-1.768088000
8	4.047634000	5.672720000	-1.864059000
1	4.522978000	1.648160000	-0.262475000
1	2.671461000	3.686802000	-1.071811000
7	5.527678000	4.055229000	-2.509524000
6	6.037697000	4.884063000	-3.600859000
6	4.957846000	5.260555000	-4.629682000
8	5.125639000	6.265217000	-5.358006000
1	5.963245000	3.158481000	-2.328907000
1	6.440778000	5.829899000	-3.230145000
7	3.849328000	4.462281000	-4.694177000
6	2.725017000	4.783676000	-5.571061000
6	1.537519000	5.426933000	-4.829476000
8	0.452947000	5.620807000	-5.444038000
6	2.247427000	3.543224000	-6.345803000
1	3.772906000	3.611720000	-4.135043000
1	3.091033000	5.540369000	-6.275800000
1	1.365505000	3.793994000	-6.938714000
1	3.045053000	3.192501000	-7.007802000
1	1.983367000	2.737831000	-5.651443000
7	1.706467000	5.816915000	-3.535774000
6	0.568151000	6.285650000	-2.738980000
6	-0.037086000	7.598992000	-3.255200000
8	-1.206715000	7.920667000	-2.914058000
6	0.933289000	6.439941000	-1.255308000
8	0.806530000	5.138758000	-0.604846000
1	2.600949000	5.689764000	-3.060734000
1	-0.247988000	5.557108000	-2.810839000
1	1.954712000	6.820619000	-1.150320000
1	0.229126000	7.152468000	-0.808691000
1	0.954303000	5.222802000	0.368196000
7	0.731662000	8.364722000	-4.066939000
6	0.210695000	9.565854000	-4.710704000
6	-0.875839000	9.329086000	-5.770949000
8	-1.504483000	10.319253000	-6.209833000
1	1.656524000	8.031525000	-4.302074000
1	-0.240024000	10.224831000	-3.963366000
7	-1.102367000	8.044317000	-6.163010000
6	-2.208614000	7.710530000	-7.050941000
6	-3.435316000	7.107101000	-6.358212000
8	-4.365783000	6.638625000	-7.062678000
1	-0.522416000	7.274124000	-5.830050000
1	-2.525926000	8.626054000	-7.558918000
7	-3.450212000	7.124771000	-4.999808000
6	-4.574430000	6.610313000	-4.217447000
6	-4.189587000	5.463783000	-3.265496000
6	-3.799299000	4.129991000	-3.891991000
6	-4.019487000	3.810808000	-5.244172000
6	-3.236556000	3.136752000	-3.070485000
6	-3.692461000	2.546998000	-5.746868000
6	-2.910026000	1.868244000	-3.557069000
6	-3.140953000	1.569752000	-4.909341000
8	-2.839613000	0.337168000	-5.466195000
1	-2.664232000	7.514675000	-4.489191000
1	-5.343656000	6.301293000	-4.929221000
1	-5.050828000	5.302788000	-2.596099000
1	-3.368340000	5.817366000	-2.623421000
1	-4.431788000	4.547085000	-5.926521000
1	-3.062462000	3.354886000	-2.018106000
1	-3.858346000	2.304651000	-6.790570000
1	-2.494057000	1.114735000	-2.894671000
1	-2.503430000	-0.339730000	-4.815796000
6	-4.837135000	4.329891000	2.434460000
6	-3.371993000	4.636484000	2.259644000
8	-2.474176000	3.784373000	2.516873000
6	-5.343953000	3.234832000	1.436820000
6	-6.089244000	2.090153000	2.149491000
6	-5.104881000	1.180148000	2.889682000
8	-4.255108000	0.497581000	2.253347000
8	-5.214702000	1.253365000	4.202558000
1	-4.957369000	3.952571000	3.457869000
1	-6.016654000	3.691086000	0.703402000
1	-4.505491000	2.804997000	0.879060000
1	-6.605760000	1.459665000	1.422456000
1	-6.844523000	2.471513000	2.840897000
7	-3.008684000	5.886646000	1.837025000
6	-1.620492000	6.167639000	1.454039000
6	-0.605556000	5.872301000	2.556231000
8	0.533266000	5.411254000	2.261560000
1	-3.726596000	6.534135000	1.543456000
1	-1.316023000	5.568465000	0.589737000
7	-0.945559000	6.161748000	3.837656000
6	-0.028573000	5.896912000	4.951122000
6	0.120562000	4.406673000	5.309182000
8	1.215189000	3.978734000	5.750737000
1	-1.860608000	6.550119000	4.019714000
1	0.970127000	6.260458000	4.703278000
7	-0.984315000	3.640399000	5.141412000
6	-1.009272000	2.176931000	5.357628000
6	-0.037753000	1.507924000	4.362077000
8	0.891851000	0.758170000	4.760001000
6	-2.472901000	1.728086000	5.185472000
6	-2.802958000	0.336452000	5.695664000
8	-2.086762000	-0.226653000	6.578124000
8	-3.883833000	-0.223766000	5.207926000
1	-1.753136000	4.018783000	4.601667000
1	-0.642690000	1.938267000	6.357031000
1	-2.770888000	1.787375000	4.138331000
1	-3.119713000	2.423902000	5.737090000
7	-0.206861000	1.844839000	3.057921000
6	0.675806000	1.331597000	2.001856000
6	2.124135000	1.803740000	2.212458000
8	3.099699000	1.073762000	1.887321000
6	0.148294000	1.758892000	0.611987000
6	-1.246707000	1.206962000	0.229804000
6	-1.263135000	-0.279993000	-0.059464000
8	-0.705976000	-1.067168000	0.773569000
8	-1.858298000	-0.670658000	-1.148448000
1	-0.988829000	2.435593000	2.788199000
1	0.696052000	0.241054000	2.047157000
1	0.882443000	1.432474000	-0.133011000
1	0.111682000	2.853453000	0.551896000
1	-1.981457000	1.394284000	1.024128000
1	-1.597910000	1.728905000	-0.663417000
7	2.298642000	3.041054000	2.752592000
6	3.612999000	3.561968000	3.110950000
6	4.360789000	2.713447000	4.141123000
8	5.606156000	2.554429000	4.025209000
1	1.502589000	3.654932000	2.875445000
1	4.280985000	3.611009000	2.246852000
7	3.645333000	2.159345000	5.148256000
6	4.275177000	1.210250000	6.066535000
6	4.495972000	-0.179255000	5.444235000
8	5.626595000	-0.750792000	5.516841000
1	2.663175000	2.390299000	5.267094000
1	5.257503000	1.577005000	6.368242000
7	3.430648000	-0.745777000	4.837001000
6	3.552020000	-2.048386000	4.170893000
6	2.500839000	-2.267445000	3.075300000
6	1.122555000	-2.527931000	3.598478000
7	0.015313000	-2.803244000	2.778601000
6	0.695649000	-2.524755000	4.912851000
6	-1.038928000	-2.943929000	3.598531000
7	-0.662947000	-2.784799000	4.888997000
1	2.549342000	-0.233370000	4.754842000
1	3.501328000	-2.853794000	4.919551000
1	2.814848000	-3.123256000	2.465616000
1	2.498494000	-1.400712000	2.404308000
1	1.208364000	-2.292084000	5.829603000
1	-2.041146000	-3.152182000	3.283061000
6	1.456882000	-0.993198000	-5.105011000
6	2.355977000	-2.114124000	-5.571816000
8	2.196127000	-3.306386000	-5.166012000
6	0.222743000	-0.818660000	-6.038017000
6	-0.669586000	-2.072138000	-6.190702000
6	-1.206908000	-2.616327000	-4.877458000
8	-1.765730000	-1.717534000	-4.055230000
8	-1.126067000	-3.828149000	-4.562691000

1	1.110232000	-1.231333000	-4.094677000
1	-0.384952000	0.000267000	-5.645235000
1	0.562166000	-0.511049000	-7.036031000
1	-0.143566000	-2.888325000	-6.692978000
1	-1.528611000	-1.787891000	-6.812534000
7	3.303789000	-1.817325000	-6.500284000
6	4.008617000	-2.831936000	-7.295018000
6	4.809479000	-3.884826000	-6.525464000
8	4.966334000	-5.016669000	-7.037851000
1	3.418017000	-0.848815000	-6.763731000
1	3.298040000	-3.398001000	-7.904990000
7	5.332363000	-3.527229000	-5.321970000
6	6.062628000	-4.481394000	-4.503314000
6	5.214915000	-5.210605000	-3.457279000
8	5.785174000	-5.790337000	-2.483773000
1	5.304789000	-2.569667000	-4.978197000
1	6.501395000	-5.244241000	-5.156556000
7	3.885120000	-5.242952000	-3.654669000
6	2.968619000	-6.001670000	-2.791721000
6	1.509783000	-5.679736000	-3.125872000
8	1.260406000	-4.251456000	-2.828490000
6	0.525544000	-6.534240000	-2.334884000
1	3.484341000	-4.702816000	-4.419012000
1	3.159218000	-5.754320000	-1.743825000
1	1.329277000	-5.814004000	-4.199227000
1	1.375472000	-3.741084000	-3.674361000
1	-0.495494000	-6.314417000	-2.662021000
1	0.715319000	-7.598485000	-2.513355000
1	0.608226000	-6.340748000	-1.260485000
6	-5.411099000	-6.125024000	3.076047000
6	-6.565023000	-5.588095000	3.918465000
8	-7.711385000	-5.423589000	3.410530000
6	-5.918566000	-7.144864000	2.037877000
6	-4.854211000	-7.612362000	1.006589000
6	-4.172297000	-6.447465000	0.321589000
8	-3.069924000	-6.000452000	0.788031000
7	-4.772757000	-5.859651000	-0.721782000
1	-4.953900000	-5.265337000	2.566828000
1	-6.777378000	-6.704731000	1.521222000
1	-6.298862000	-8.035037000	2.554113000
1	-5.336957000	-8.263904000	0.268271000
1	-4.074002000	-8.195335000	1.505020000
1	-4.377283000	-5.008353000	-1.152652000
1	-5.642932000	-6.216226000	-1.087168000
7	-6.293383000	-5.272965000	5.210639000
6	-7.343192000	-4.781843000	6.104244000
6	-7.973926000	-3.435207000	5.728674000
8	-9.120589000	-3.155208000	6.148781000
1	-5.324467000	-5.247302000	5.539886000
1	-8.171373000	-5.494508000	6.140502000
7	-7.221234000	-2.605858000	4.955637000
6	-7.739710000	-1.342836000	4.451640000
6	-8.021748000	-1.347914000	2.947403000
8	-8.082867000	-0.256896000	2.318953000
1	-6.249605000	-2.828697000	4.721917000
1	-8.677420000	-1.133018000	4.978258000
7	-8.232609000	-2.561127000	2.374000000
6	-8.576103000	-2.718869000	0.964458000
6	-7.552157000	-3.548349000	0.192788000
8	-6.266003000	-2.870347000	0.093953000
1	-8.174792000	-3.403267000	2.942959000
1	-8.664466000	-1.721827000	0.529276000
1	-7.400994000	-4.508198000	0.700174000
1	-7.934773000	-3.738804000	-0.818151000
1	-5.911921000	-2.802516000	-0.838152000
6	-8.517794000	3.679666000	-3.737014000
6	-8.038402000	2.560589000	-2.836456000
6	-8.699567000	2.269296000	-1.629742000
6	-6.931831000	1.772632000	-3.201778000
6	-8.273890000	1.215861000	-0.810469000
6	-6.504195000	0.716745000	-2.389305000
6	-7.172993000	0.435833000	-1.191273000
1	-9.225116000	3.309355000	-4.492107000
1	-9.032485000	4.462132000	-3.167907000
1	-9.555169000	2.871598000	-1.332646000
1	-6.396284000	1.992652000	-4.120712000
1	-8.780874000	1.000269000	0.125418000
1	-5.648719000	0.118674000	-2.686838000
1	-6.839579000	-0.378376000	-0.556271000
26	-0.266006000	-3.013966000	0.792862000
26	-2.149847000	-2.298895000	-2.190103000
8	0.280511000	-4.946559000	0.851827000
1	1.266336000	-4.972913000	0.754444000
1	-0.113824000	-5.533554000	1.594928000
8	-4.445076000	-2.639914000	4.175322000
1	-4.243309000	-1.772884000	4.626135000
1	-4.427170000	-2.508338000	3.172680000
8	-4.330069000	-2.086555000	1.622779000
1	-5.147548000	-2.368665000	1.091946000
1	-4.261282000	-1.095623000	1.718245000
8	-2.208827000	-3.474287000	0.982823000
1	-2.523450000	-4.409761000	0.903427000
1	-2.968999000	-2.837030000	1.175753000
8	-0.218521000	-3.964459000	8.303803000
1	0.094749000	-4.627405000	7.600237000
1	0.024066000	-4.213668000	9.208041000
8	-4.867188000	-2.585806000	-2.155821000
8	-3.792969000	-3.445228000	-1.911148000
8	-0.696656000	-3.577707000	-1.300188000
1	0.123061000	-3.797333000	-1.897791000
8	-1.005248000	-6.408547000	2.485209000
1	-1.826296000	-6.494223000	1.945618000
1	-1.223662000	-6.293837000	3.447923000
8	-2.210793000	-2.859458000	7.174267000
1	-1.483780000	-3.203236000	7.796321000
1	-2.314002000	-1.867837000	7.148979000
8	-1.816679000	-6.246570000	5.077447000
1	-2.098103000	-7.137662000	5.352356000
1	-2.556538000	-5.520442000	5.298418000
8	0.406063000	-5.471198000	6.310508000
1	-0.413716000	-5.821511000	5.843596000
1	0.950648000	-4.966342000	5.679509000
8	-3.521701000	-4.507902000	5.681112000
1	-3.865664000	-3.848622000	4.980876000
1	-3.134119000	-3.935633000	6.430771000
1	3.636300000	1.117719000	6.949556000
1	3.471023000	4.574730000	3.499147000
1	-0.386314000	6.443079000	5.829039000
1	-1.554359000	7.224092000	1.173665000
1	-5.441806000	5.239653000	2.351054000
1	-4.992645000	7.430950000	-3.618717000
1	-1.882129000	6.993166000	-7.808714000
1	1.041887000	10.102531000	-5.175574000
1	6.845383000	4.335408000	-4.092756000
1	3.920436000	3.765716000	0.163958000
1	8.375576000	-1.701350000	-2.424393000
1	9.876020000	2.039388000	0.276017000
1	6.190743000	0.803620000	3.371034000
1	9.142500000	-2.567232000	4.171848000
1	4.686869000	-2.304253000	-7.972988000
1	6.867521000	-3.963114000	-3.979194000
1	-6.917056000	-4.704215000	7.109388000
1	-7.040720000	-0.526698000	4.652818000
1	-4.630544000	-6.558186000	3.712124000
1	-9.551210000	-3.219090000	0.880369000
1	8.416919000	-5.100839000	0.850185000
1	-7.684561000	4.141470000	-4.277831000
1	2.005343000	-0.045113000	-5.043022000
1	3.148751000	-7.076395000	-2.926044000
1	-1.060082000	-4.427080000	-0.973886000
1	4.554204000	-2.091665000	3.732979000

^{3}TS1$_{B}$:

6	8.866680000	-1.270220000	3.262680000
6	8.144947000	-0.136557000	2.548310000
8	8.154418000	-0.089008000	1.282876000
6	7.969088000	-2.535038000	3.454054000
6	7.089956000	-2.862208000	2.287414000
7	7.534476000	-3.184088000	0.996827000
6	5.722344000	-2.843054000	2.199296000

6	6.478933000	-3.332542000	0.158160000
7	5.372560000	-3.131550000	0.885670000
1	9.737277000	-1.520509000	2.648645000
1	8.619503000	-3.381814000	3.708273000
1	7.312464000	-2.379843000	4.316378000
1	4.982371000	-2.636370000	2.950895000
1	6.507013000	-3.538690000	-0.914209000
1	4.364447000	-3.023895000	0.531725000
7	7.499818000	0.778254000	3.313495000
6	6.587016000	1.789332000	2.768203000
6	7.097190000	2.585396000	1.597268000
8	6.298343000	2.964487000	0.694357000
1	7.392530000	0.648701000	4.318368000
1	5.636074000	1.351009000	2.453057000
7	8.405050000	2.922942000	1.506892000
6	8.870834000	3.698448000	0.354398000
6	8.460567000	3.088724000	-0.993251000
8	8.210075000	3.834305000	-1.972272000
1	9.044699000	2.662119000	2.242363000
1	8.459340000	4.710912000	0.369117000
7	8.422588000	1.728395000	-1.073091000
6	8.066290000	1.069817000	-2.323780000
6	6.575514000	1.067522000	-2.681689000
8	6.230189000	0.658654000	-3.822705000
1	8.508947000	1.157082000	-0.232729000
1	8.577126000	1.560813000	-3.156453000
7	5.709431000	1.496013000	-1.737589000
6	4.271232000	1.671938000	-1.998424000
6	3.930671000	3.171741000	-2.047273000
8	3.364663000	3.675725000	-3.057647000
6	3.386719000	0.940246000	-0.968555000
6	3.601263000	-0.578474000	-0.943459000
6	2.677169000	-1.279061000	0.039145000
8	1.676354000	-0.628699000	0.492856000
8	2.920234000	-2.521287000	0.368314000
1	6.072770000	1.835224000	-0.848721000
1	4.090158000	1.286710000	-3.002968000
1	2.339311000	1.144639000	-1.216587000
1	3.545963000	1.356878000	0.032691000
1	4.642930000	-0.825062000	-0.703944000
1	3.392645000	-1.025645000	-1.926248000
7	4.281757000	3.894854000	-0.955630000
6	3.978258000	5.311333000	-0.802433000
6	4.677570000	6.246398000	-1.776968000
8	4.213817000	7.413314000	-1.937434000
1	4.745845000	3.444912000	-0.168532000
1	2.906366000	5.507610000	-0.928373000
7	5.714662000	5.787180000	-2.512708000
6	6.194973000	6.561833000	-3.656507000
6	5.100097000	6.863856000	-4.693617000
8	5.254815000	7.817272000	-5.490689000
1	6.182426000	4.917036000	-2.280794000
1	6.588122000	7.533197000	-3.345196000
7	3.995809000	6.058794000	-4.687880000
6	2.853601000	6.311992000	-5.562783000
6	1.639484000	6.892268000	-4.812214000
8	0.516230000	6.943785000	-5.384394000
6	2.443061000	5.046328000	-6.334212000
1	3.926809000	5.251213000	-4.067397000
1	3.179155000	7.084424000	-6.271375000
1	1.542235000	5.242649000	-6.919098000
1	3.255180000	4.744608000	-7.002904000
1	2.233164000	4.228948000	-5.635170000
7	1.827225000	7.399182000	-3.561790000
6	0.684221000	7.839603000	-2.753857000
6	-0.041484000	9.062156000	-3.337047000
8	-1.213577000	9.327019000	-2.960303000
6	1.087093000	8.124008000	-1.299062000
8	1.022626000	6.872099000	-0.548012000
1	2.742987000	7.359289000	-3.111283000
1	-0.076121000	7.050020000	-2.741926000
1	2.097882000	8.542521000	-1.254631000
1	0.371832000	8.844587000	-0.884787000
1	1.200610000	7.022300000	0.411720000
7	0.628742000	9.810279000	-4.247688000
6	-0.012816000	10.908647000	-4.961678000
6	-1.088979000	10.499372000	-5.979072000
8	-1.785835000	11.398879000	-6.501782000
1	1.565217000	9.527408000	-4.501175000
1	-0.510234000	11.573169000	-4.249672000
7	-1.233246000	9.170552000	-6.242028000
6	-2.312599000	8.682980000	-7.091195000
6	-3.540881000	8.150869000	-6.344210000
8	-4.471772000	7.620715000	-7.002080000
1	-0.598468000	8.476392000	-5.848593000
1	-2.647217000	9.505103000	-7.730376000
7	-3.556302000	8.297452000	-4.993453000
6	-4.695986000	7.888783000	-4.172112000
6	-4.351854000	6.831149000	-3.109458000
6	-3.976379000	5.437233000	-3.596237000
6	-4.240682000	4.966841000	-4.895405000
6	-3.395672000	4.539090000	-2.682886000
6	-3.942605000	3.648298000	-5.258465000
6	-3.097964000	3.218852000	-3.029077000
6	-3.373458000	2.769095000	-4.329267000
8	-3.095572000	1.475904000	-4.741504000
1	-2.766799000	8.728279000	-4.524224000
1	-5.468560000	7.531482000	-4.856969000
1	-5.232317000	6.747251000	-2.451289000
1	-3.537777000	7.226482000	-2.482848000
1	-4.661107000	5.627430000	-5.647011000
1	-3.184480000	4.876600000	-1.669521000
1	-4.142305000	3.290340000	-6.262216000
1	-2.657017000	2.536127000	-2.310904000
1	-2.651402000	0.911174000	-4.055047000
6	-4.633756000	6.261440000	2.446204000
6	-3.151464000	6.497411000	2.301591000
8	-2.294746000	5.607161000	2.568343000
6	-5.169317000	5.157208000	1.473702000
6	-5.899909000	4.023446000	2.213010000
6	-4.908969000	3.105756000	2.933405000
8	-3.969785000	2.546551000	2.298596000
8	-5.127961000	3.016302000	4.229919000
1	-4.801533000	5.932912000	3.480088000
1	-5.858932000	5.609930000	0.754440000
1	-4.346621000	4.717836000	0.900780000
1	-6.447352000	3.388850000	1.509841000
1	-6.634390000	4.413643000	2.922455000
7	-2.730652000	7.736816000	1.897253000
6	-1.329771000	7.965568000	1.531058000
6	-0.334011000	7.626111000	2.636891000
8	0.805197000	7.167506000	2.336246000
1	-3.418316000	8.412438000	1.594716000
1	-1.041144000	7.360099000	0.665612000
7	-0.689561000	7.872591000	3.921979000
6	0.200336000	7.554116000	5.042449000
6	0.298799000	6.054193000	5.377844000
8	1.362992000	5.599709000	5.867358000
1	-1.607178000	8.256280000	4.101736000
1	1.213211000	7.891018000	4.815465000
7	-0.808769000	5.311520000	5.143934000
6	-0.858692000	3.844419000	5.334704000
6	0.122764000	3.188260000	4.339719000
8	1.026622000	2.405121000	4.732184000
6	-2.323564000	3.410319000	5.136355000
6	-2.721764000	2.068555000	5.730185000
8	-2.066475000	1.523551000	6.669770000
8	-3.814292000	1.529545000	5.240654000
1	-1.543312000	5.704459000	4.565802000
1	-0.506999000	3.587806000	6.335298000
1	-2.580377000	3.393387000	4.076058000
1	-2.976434000	4.156762000	5.608489000
7	-0.000341000	3.573923000	3.042332000
6	0.920175000	3.082824000	2.008869000
6	2.361124000	3.535500000	2.296467000
8	3.339563000	2.780439000	2.049666000
6	0.472593000	3.540723000	0.599393000
6	-0.904865000	3.024949000	0.107602000
6	-1.000359000	1.515681000	0.019209000
8	-0.738703000	0.845432000	1.073019000

8	-1.361769000	0.990663000	-1.122732000
1	-0.779813000	4.162719000	2.762874000
1	0.939072000	1.993326000	2.040440000
1	1.245933000	3.215897000	-0.105656000
1	0.453273000	4.635996000	0.549602000
1	-1.705386000	3.352332000	0.784828000
1	-1.101789000	3.456509000	-0.875857000
7	2.527471000	4.784568000	2.812224000
6	3.837508000	5.299330000	3.192926000
6	4.559921000	4.462778000	4.249854000
8	5.806896000	4.299236000	4.160060000
1	1.738795000	5.417971000	2.877625000
1	4.524090000	5.330411000	2.342204000
7	3.824689000	3.931500000	5.255759000
6	4.446546000	3.025027000	6.222074000
6	4.658635000	1.600345000	5.680576000
8	5.797909000	1.046335000	5.738233000
1	2.845190000	4.182952000	5.364548000
1	5.431995000	3.400716000	6.501815000
7	3.572950000	0.978709000	5.167824000
6	3.679847000	-0.374346000	4.607855000
6	2.597309000	-0.662660000	3.562410000
6	1.228071000	-0.874570000	4.131956000
7	0.105870000	-1.062485000	3.315053000
6	0.803130000	-0.886228000	5.445670000
6	-0.958391000	-1.163826000	4.128014000
7	-0.569789000	-1.069513000	5.416571000
1	2.690335000	1.482372000	5.052990000
1	3.646828000	-1.118060000	5.417941000
1	2.888716000	-1.554255000	2.989903000
1	2.575284000	0.161876000	2.840251000
1	1.331936000	-0.708461000	6.365660000
1	-1.983150000	-1.274214000	3.819028000
6	1.440350000	0.622688000	-4.810489000
6	2.361254000	-0.321597000	-5.545388000
8	2.285447000	-1.582299000	-5.396442000
6	0.063282000	0.796915000	-5.519075000
6	-0.782838000	-0.483259000	-5.676380000
6	-1.144272000	-1.197422000	-4.374099000
8	-1.526765000	-0.371895000	-3.383730000
8	-1.083524000	-2.439778000	-4.270043000
1	1.267751000	0.222067000	-3.806114000
1	-0.507951000	1.526936000	-4.941004000
1	0.223839000	1.234032000	-6.513748000
1	-0.291134000	-1.221987000	-6.315167000
1	-1.723933000	-0.189496000	-6.158762000
7	3.261182000	0.202780000	-6.417800000
6	4.022138000	-0.628579000	-7.359036000
6	4.946699000	-1.684917000	-6.743749000
8	5.189225000	-2.730468000	-7.387783000
1	3.315746000	1.207483000	-6.507431000
1	3.340996000	-1.184069000	-8.009926000
7	5.478572000	-1.415218000	-5.519652000
6	6.334713000	-2.367382000	-4.828338000
6	5.630894000	-3.137301000	-3.708231000
8	6.301342000	-3.590110000	-2.731786000
1	5.381758000	-0.505669000	-5.075003000
1	6.702376000	-3.093638000	-5.562500000
7	4.308066000	-3.340921000	-3.853790000
6	3.510583000	-4.150561000	-2.923818000
6	2.018509000	-4.037857000	-3.248848000
8	1.621358000	-2.632049000	-3.074319000
6	1.151958000	-4.918410000	-2.356242000
1	3.825183000	-2.878005000	-4.620088000
1	3.668302000	-3.802317000	-1.897775000
1	1.843315000	-4.300386000	-4.300304000
1	1.648132000	-2.176614000	-3.963207000
1	0.098985000	-4.768711000	-2.609814000
1	1.399213000	-5.975941000	-2.501998000
1	1.298595000	-4.671423000	-1.299741000
6	-5.429708000	-4.423115000	2.952728000
6	-6.586969000	-3.777898000	3.705548000
8	-7.680734000	-3.535091000	3.121047000
6	-5.958431000	-5.472934000	1.953463000
6	-4.884497000	-6.065448000	1.002321000
6	-4.189172000	-4.991489000	0.189799000
8	-3.202168000	-4.356989000	0.670180000
7	-4.688311000	-4.672941000	-1.022092000
1	-4.888827000	-3.634027000	2.413722000
1	-6.763625000	-5.010956000	1.372639000
1	-6.415511000	-6.303395000	2.506301000
1	-5.359799000	-6.803110000	0.344735000
1	-4.114581000	-6.585695000	1.582082000
1	-4.266291000	-3.891951000	-1.530306000
1	-5.465998000	-5.169956000	-1.426187000
7	-6.385282000	-3.472784000	5.014877000
6	-7.471533000	-2.950546000	5.844192000
6	-8.046801000	-1.591155000	5.434109000
8	-9.208844000	-1.284843000	5.788121000
1	-5.441468000	-3.491051000	5.405786000
1	-8.318250000	-3.642270000	5.833808000
7	-7.229892000	-0.775662000	4.713706000
6	-7.690070000	0.504511000	4.199916000
6	-7.840401000	0.550904000	2.679407000
8	-7.905987000	1.669927000	2.103695000
1	-6.255262000	-1.024253000	4.529866000
1	-8.667164000	0.710418000	4.650402000
7	-7.928173000	-0.638615000	2.029201000
6	-8.128102000	-0.728363000	0.584263000
6	-6.935126000	-1.313092000	-0.167359000
8	-5.797928000	-0.406500000	-0.093784000
1	-7.919519000	-1.505328000	2.564086000
1	-8.335508000	0.277702000	0.214381000
1	-6.662315000	-2.291387000	0.252609000
1	-7.214018000	-1.452280000	-1.219377000
1	-5.140513000	-0.535166000	-0.838567000
6	-9.837534000	6.263292000	-2.899967000
6	-8.993854000	5.145336000	-2.324228000
6	-9.323284000	4.551848000	-1.093451000
6	-7.874141000	4.657339000	-3.022117000
6	-8.564540000	3.494402000	-0.575264000
6	-7.110560000	3.601254000	-2.511074000
6	-7.454620000	3.015337000	-1.285491000
1	-9.227008000	6.979704000	-3.461267000
1	-10.596379000	5.874642000	-3.593329000
1	-10.183557000	4.919762000	-0.538908000
1	-7.598218000	5.104404000	-3.973702000
1	-8.820731000	3.041608000	0.377216000
1	-6.249372000	3.244057000	-3.065923000
1	-6.869640000	2.191969000	-0.885253000
26	-0.208352000	-1.157207000	1.249948000
26	-1.809457000	-0.871449000	-1.554160000
8	0.310377000	-3.190756000	0.887307000
1	1.300075000	-3.243819000	0.852346000
1	-0.206349000	-3.831701000	1.539181000
8	-4.386050000	-0.829503000	4.122166000
1	-4.224777000	0.016129000	4.627398000
1	-4.250826000	-0.642366000	3.134075000
8	-3.972869000	-0.044205000	1.675291000
1	-4.795470000	-0.162231000	1.081680000
1	-3.822750000	0.924284000	1.867359000
8	-2.113463000	-1.399203000	0.395035000
1	-2.868488000	-2.173621000	-0.306938000
1	-2.759525000	-0.792149000	0.903401000
8	-0.886152000	-2.509017000	8.778800000
1	-0.512476000	-3.132888000	8.070961000
1	-0.875715000	-2.888351000	9.670204000
8	-3.730342000	-0.823803000	-1.705729000
8	-3.260456000	-2.256486000	-1.457294000
8	-0.229157000	-1.983138000	-1.413961000
1	0.432918000	-2.204127000	-2.156988000
8	-1.172927000	-4.536114000	2.362590000
1	-2.009625000	-4.569328000	1.825617000
1	-1.347324000	-4.423561000	3.332117000
8	-2.450922000	-1.109430000	7.371667000
1	-1.917841000	-1.531042000	8.128022000
1	-2.504673000	-0.116399000	7.348656000
8	-1.925323000	-4.451836000	5.019971000
1	-2.257095000	-5.358517000	5.155560000
1	-2.665070000	-3.731955000	5.265468000

8	-0.015347000	-3.906619000	6.783456000
1	-0.708402000	-4.159032000	6.096798000
1	0.747740000	-3.491176000	6.345904000
8	-3.621485000	-2.722596000	5.669483000
1	-3.925097000	-2.051566000	4.960106000
1	-3.300106000	-2.175184000	6.460267000
1	3.808086000	2.986094000	7.109593000
1	3.696050000	6.319528000	3.562176000
1	-0.150786000	8.094695000	5.926542000
1	-1.219231000	9.020333000	1.258273000
1	-5.189991000	7.196024000	2.313876000
1	-5.096686000	8.774448000	-3.660287000
1	-1.947233000	7.875812000	-7.731828000
1	0.755108000	11.486854000	-5.482015000
1	7.004723000	5.999042000	-4.128686000
1	4.242584000	5.600928000	0.217902000
1	8.405944000	0.030751000	-2.283110000
1	9.961891000	3.766572000	0.403713000
1	6.369064000	2.507230000	3.566907000
1	9.233480000	-0.955535000	4.245444000
1	4.619356000	0.041300000	-7.985491000
1	7.185618000	-1.846047000	-4.387667000
1	-7.102755000	-2.880898000	6.872473000
1	-7.005367000	1.309068000	4.483002000
1	-4.711414000	-4.868691000	3.651445000
1	-9.002712000	-1.360740000	0.383053000
1	8.497316000	-3.210051000	0.699403000
1	-10.367759000	6.811630000	-2.113225000
1	1.908685000	1.607457000	-4.690453000
1	3.832342000	-5.198662000	-2.979960000
1	-0.169670000	-2.651258000	-0.671037000
1	4.670174000	-0.459395000	4.148406000

^{3}IM1$_B$:

6	8.742136000	-3.016914000	3.194625000
6	8.037025000	-1.873152000	2.479952000
8	8.050669000	-1.825496000	1.214378000
6	7.836672000	-4.278778000	3.365436000
6	6.973070000	-4.600603000	2.185544000
7	7.433385000	-4.915536000	0.898669000
6	5.606318000	-4.590612000	2.083017000
6	6.387551000	-5.068830000	0.048500000
7	5.272459000	-4.877268000	0.765017000
1	9.619354000	-3.267342000	2.589917000
1	8.480455000	-5.128809000	3.625887000
1	7.167887000	-4.125186000	4.218683000
1	4.856620000	-4.393342000	2.827588000
1	6.426013000	-5.273207000	-1.023701000
1	4.267254000	-4.777144000	0.400937000
7	7.404863000	-0.948625000	3.244009000
6	6.511198000	0.079391000	2.697499000
6	7.033265000	0.855552000	1.518346000
8	6.241519000	1.230259000	0.607352000
1	7.288974000	-1.079481000	4.247998000
1	5.549862000	-0.340448000	2.388769000
7	8.344034000	1.181885000	1.428737000
6	8.819094000	1.942102000	0.269540000
6	8.404100000	1.325326000	-1.073491000
8	8.159851000	2.065452000	-2.058196000
1	8.979575000	0.924632000	2.169061000
1	8.417979000	2.958824000	0.274812000
7	8.356115000	-0.035158000	-1.143348000
6	7.997514000	-0.699651000	-2.390013000
6	6.508265000	-0.690780000	-2.752764000
8	6.164171000	-1.093933000	-3.896127000
1	8.433769000	-0.600849000	-0.298337000
1	8.515258000	-0.220000000	-3.224946000
7	5.640144000	-0.261458000	-1.810163000
6	4.204686000	-0.077621000	-2.080003000
6	3.866360000	1.422558000	-2.123060000
8	3.297538000	1.927340000	-3.132634000
6	3.309327000	-0.819135000	-1.066792000
6	3.488226000	-2.343423000	-1.097141000
6	2.560635000	-3.057074000	-0.127067000
8	1.538884000	-2.420540000	0.299224000
8	2.822106000	-4.288565000	0.220436000
1	6.001875000	0.075925000	-0.919924000
1	4.031944000	-0.455166000	-3.088844000
1	2.264857000	-0.582100000	-1.296454000
1	3.488529000	-0.437551000	-0.054614000
1	4.526582000	-2.623322000	-0.883903000
1	3.254165000	-2.748379000	-2.092812000
7	4.220598000	2.143746000	-1.032617000
6	3.926855000	3.562296000	-0.878769000
6	4.633821000	4.491587000	-1.853223000
8	4.182950000	5.664000000	-2.010148000
1	4.683777000	1.693121000	-0.245104000
1	2.856492000	3.767069000	-1.004607000
7	5.662522000	4.022600000	-2.594694000
6	6.145516000	4.794670000	-3.739087000
6	5.048357000	5.112503000	-4.768980000
8	5.208811000	6.067559000	-5.562887000
1	6.124409000	3.148955000	-2.364577000
1	6.551859000	5.760531000	-3.427623000
7	3.935423000	4.318811000	-4.762347000
6	2.793244000	4.589323000	-5.632367000
6	1.594894000	5.199497000	-4.880041000
8	0.473387000	5.283222000	-5.452258000
6	2.352846000	3.326783000	-6.392603000
1	3.860424000	3.509756000	-4.144498000
1	3.129721000	5.350194000	-6.348156000
1	1.454068000	3.538168000	-6.975458000
1	3.155359000	3.003196000	-7.062675000
1	2.128636000	2.518777000	-5.687063000
7	1.796062000	5.693955000	-3.627005000
6	0.666037000	6.156982000	-2.814304000
6	-0.022920000	7.407512000	-3.380864000
8	-1.184153000	7.705665000	-2.993142000
6	1.074849000	6.408459000	-1.355398000
8	0.987727000	5.145423000	-0.627208000
1	2.711462000	5.630905000	-3.178389000
1	-0.116837000	5.389395000	-2.814565000
1	2.093227000	6.807398000	-1.304826000
1	0.373919000	7.135743000	-0.928620000
1	1.131781000	5.283030000	0.340578000
7	0.662209000	8.139372000	-4.292563000
6	0.048776000	9.259168000	-4.998351000
6	-1.068678000	8.884789000	-5.984227000
8	-1.761200000	9.804463000	-6.476367000
1	1.588876000	7.830688000	-4.552170000
1	-0.403307000	9.949833000	-4.281095000
7	-1.251674000	7.561981000	-6.254657000
6	-2.373652000	7.107491000	-7.065948000
6	-3.586711000	6.603861000	-6.275606000
8	-4.549710000	6.088787000	-6.898586000
1	-0.620845000	6.849541000	-5.888259000
1	-2.709821000	7.941433000	-7.688910000
7	-3.554170000	6.757116000	-4.925794000
6	-4.674232000	6.377491000	-4.065147000
6	-4.317201000	5.316131000	-3.009822000
6	-3.992674000	3.912040000	-3.504661000
6	-4.293579000	3.454220000	-4.800576000
6	-3.419547000	2.995270000	-2.605024000
6	-4.034347000	2.131031000	-5.175147000
6	-3.164066000	1.669159000	-2.962736000
6	-3.470282000	1.234397000	-4.260391000
8	-3.232633000	-0.063698000	-4.683369000
1	-2.741974000	7.175076000	-4.483629000
1	-5.477920000	6.034724000	-4.721329000
1	-5.175436000	5.255433000	-2.320320000
1	-3.471501000	5.695221000	-2.415828000
1	-4.712734000	4.127789000	-5.541179000
1	-3.176674000	3.322677000	-1.595654000
1	-4.259763000	1.783233000	-6.176897000
1	-2.735046000	0.969825000	-2.253171000
1	-2.754013000	-0.631974000	-4.024852000
6	-4.737471000	4.454846000	2.218852000
6	-3.256897000	4.716221000	2.099522000
8	-2.390388000	3.841922000	2.386024000
6	-5.245689000	3.358571000	1.221805000

6	-5.975714000	2.207171000	1.936159000
6	-4.983657000	1.294566000	2.662689000
8	-4.074260000	0.693043000	2.027381000
8	-5.176951000	1.257668000	3.967326000
1	-4.912421000	4.105231000	3.244389000
1	-5.929065000	3.812314000	0.497265000
1	-4.409992000	2.935281000	0.655643000
1	-6.502493000	1.571347000	1.218016000
1	-6.720690000	2.589030000	2.640237000
7	-2.848862000	5.960664000	1.696706000
6	-1.444322000	6.209203000	1.357437000
6	-0.467196000	5.902268000	2.489686000
8	0.683672000	5.451405000	2.223828000
1	-3.541140000	6.623173000	1.376185000
1	-1.126806000	5.597731000	0.506634000
7	-0.851265000	6.170036000	3.762415000
6	0.024181000	5.887047000	4.904323000
6	0.149923000	4.392325000	5.253846000
8	1.226393000	3.954063000	5.729811000
1	-1.775285000	6.549172000	3.917006000
1	1.033419000	6.244627000	4.693475000
7	-0.952595000	3.634764000	5.040059000
6	-0.985378000	2.167176000	5.230998000
6	0.041671000	1.525006000	4.274368000
8	0.938409000	0.748159000	4.694871000
6	-2.434642000	1.710596000	4.969055000
6	-2.828414000	0.358932000	5.541431000
8	-2.212931000	-0.150528000	6.527477000
8	-3.866780000	-0.222063000	4.990145000
1	-1.700702000	4.021176000	4.476806000
1	-0.673732000	1.912892000	6.245410000
1	-2.648681000	1.699016000	3.899732000
1	-3.118956000	2.441630000	5.420613000
7	-0.047115000	1.912278000	2.975415000
6	0.880304000	1.404009000	1.959410000
6	2.318676000	1.862467000	2.235833000
8	3.297009000	1.109703000	1.978986000
6	0.426495000	1.823433000	0.542048000
6	-0.955044000	1.287157000	0.086293000
6	-1.033946000	-0.224292000	0.051787000
8	-0.739838000	-0.862702000	1.113636000
8	-1.391629000	-0.795051000	-1.072677000
1	-0.822563000	2.497599000	2.677880000
1	0.898130000	0.315469000	2.016925000
1	1.193285000	1.477889000	-0.160244000
1	0.405115000	2.916600000	0.461002000
1	-1.750070000	1.635980000	0.757983000
1	-1.162439000	1.682222000	-0.909707000
7	2.484026000	3.109294000	2.755915000
6	3.792633000	3.618122000	3.147953000
6	4.501003000	2.765284000	4.201605000
8	5.747657000	2.595353000	4.125363000
1	1.690452000	3.734146000	2.838120000
1	4.484197000	3.655812000	2.301603000
7	3.748417000	2.224619000	5.190133000
6	4.349438000	1.299316000	6.151721000
6	4.567005000	-0.116438000	5.589775000
8	5.704064000	-0.673992000	5.655639000
1	2.764682000	2.465872000	5.276603000
1	5.330861000	1.666924000	6.455189000
7	3.489017000	-0.728448000	5.050046000
6	3.606255000	-2.074313000	4.474085000
6	2.507779000	-2.371357000	3.448912000
6	1.150786000	-2.590799000	4.043516000
7	0.028427000	-2.827682000	3.242245000
6	0.737941000	-2.569385000	5.361205000
6	-1.026455000	-2.926724000	4.065870000
7	-0.630889000	-2.782449000	5.349859000
1	2.606334000	-0.224502000	4.936262000
1	3.598310000	-2.824988000	5.278309000
1	2.794422000	-3.263656000	2.874828000
1	2.468997000	-1.549788000	2.723041000
1	1.272386000	-2.351672000	6.269291000
1	-2.051391000	-3.066698000	3.768446000
6	1.371404000	-1.057574000	-4.728601000
6	2.230253000	-2.011895000	-5.527243000
8	2.125601000	-3.272870000	-5.417909000
6	0.022290000	-0.709669000	-5.423592000
6	-0.914853000	-1.904744000	-5.700153000
6	-1.263125000	-2.726910000	-4.464558000
8	-1.684468000	-1.989664000	-3.407310000
8	-1.132516000	-3.963375000	-4.438388000
1	1.158851000	-1.525187000	-3.761755000
1	-0.497039000	0.004881000	-4.779313000
1	0.223401000	-0.195050000	-6.372508000
1	-0.483319000	-2.597779000	-6.427625000
1	-1.846106000	-1.501056000	-6.115424000
7	3.119454000	-1.481494000	-6.409457000
6	3.837965000	-2.306076000	-7.388160000
6	4.768901000	-3.379891000	-6.814072000
8	4.986562000	-4.418267000	-7.478033000
1	3.199605000	-0.476288000	-6.469543000
1	3.129288000	-2.845482000	-8.022860000
7	5.331794000	-3.131741000	-5.599027000
6	6.195592000	-4.099174000	-4.939572000
6	5.511022000	-4.872911000	-3.810512000
8	6.197586000	-5.335342000	-2.849774000
1	5.256464000	-2.226380000	-5.142253000
1	6.535847000	-4.821934000	-5.690288000
7	4.184810000	-5.074320000	-3.932321000
6	3.414884000	-5.912200000	-3.003786000
6	1.923960000	-5.894115000	-3.353661000
8	1.423470000	-4.524730000	-3.180080000
6	1.110292000	-6.827148000	-2.462672000
1	3.685123000	-4.600412000	-4.680757000
1	3.531382000	-5.543798000	-1.979145000
1	1.784034000	-6.173978000	-4.406394000
1	1.445772000	-4.032956000	-4.047290000
1	0.052836000	-6.770640000	-2.735194000
1	1.444828000	-7.863950000	-2.579311000
1	1.212546000	-6.545113000	-1.409473000
6	-5.492965000	-6.403338000	3.233245000
6	-6.671374000	-5.632258000	3.811242000
8	-7.732687000	-5.472447000	3.144611000
6	-5.981548000	-7.626618000	2.432069000
6	-4.866515000	-8.349298000	1.628445000
6	-4.177471000	-7.395046000	0.673936000
8	-3.193374000	-6.694040000	1.055921000
7	-4.697394000	-7.234189000	-0.558806000
1	-4.934087000	-5.726035000	2.574536000
1	-6.776686000	-7.293058000	1.756642000
1	-6.438770000	-8.353996000	3.114850000
1	-5.305039000	-9.195647000	1.086193000
1	-4.105304000	-8.746557000	2.307842000
1	-4.317178000	-6.474942000	-1.132051000
1	-5.487459000	-7.769970000	-0.880915000
7	-6.517099000	-5.121335000	5.061875000
6	-7.627170000	-4.460637000	5.747183000
6	-8.163030000	-3.185716000	5.089699000
8	-9.339748000	-2.821681000	5.318158000
1	-5.585686000	-5.093270000	5.482554000
1	-8.483383000	-5.137096000	5.816735000
7	-7.293923000	-2.498471000	4.299603000
6	-7.700496000	-1.303442000	3.579612000
6	-7.707646000	-1.460359000	2.059559000
8	-7.671334000	-0.431580000	1.331720000
1	-6.310210000	-2.767405000	4.224070000
1	-8.716915000	-1.053492000	3.904941000
7	-7.800163000	-2.723663000	1.568156000
6	-7.951944000	-2.997866000	0.138476000
6	-6.735317000	-3.645300000	-0.516776000
8	-5.649075000	-2.677514000	-0.617706000
1	-7.858209000	-3.507717000	2.215744000
1	-8.166951000	-2.049345000	-0.358937000
1	-6.402836000	-4.522352000	0.053383000
1	-7.012986000	-3.973370000	-1.526353000
1	-4.956053000	-2.971343000	-1.264476000
6	-9.859780000	5.088570000	-2.555627000
6	-9.093736000	3.848920000	-2.143667000
6	-9.481852000	3.101236000	-1.017788000

6	-7.993770000	3.399479000	-2.896291000
6	-8.799594000	1.932173000	-0.659464000
6	-7.306850000	2.231374000	-2.542774000
6	-7.709594000	1.492390000	-1.422856000
1	-10.645859000	4.847343000	-3.284596000
1	-10.348912000	5.562434000	-1.697054000
1	-10.328095000	3.436478000	-0.422454000
1	-7.676284000	3.962611000	-3.770369000
1	-9.098419000	1.358772000	0.211609000
1	-6.461476000	1.904290000	-3.139269000
1	-7.189374000	0.584695000	-1.135091000
26	-0.295209000	-2.920835000	1.170910000
26	-1.888031000	-2.616967000	-1.623470000
8	0.226980000	-4.976225000	0.821048000
1	1.214091000	-5.009192000	0.732552000
1	-0.205679000	-5.591778000	1.544526000
8	-4.430619000	-2.597793000	3.848444000
1	-4.258903000	-1.731343000	4.309893000
1	-4.352043000	-2.461187000	2.846512000
8	-4.155112000	-1.900905000	1.343998000
1	-4.866362000	-2.127649000	0.653029000
1	-4.064711000	-0.921107000	1.498463000
8	-2.088340000	-3.147088000	0.258912000
1	-2.977265000	-4.509604000	-0.288508000
1	-2.824797000	-2.625565000	0.720525000
8	-1.151628000	-4.071794000	8.867946000
1	-0.715188000	-4.713668000	8.213697000
1	-1.255571000	-4.440114000	9.757988000
8	-3.398964000	-3.603280000	-1.976886000
8	-3.539084000	-4.800569000	-1.078396000
8	-0.497090000	-3.950628000	-1.564955000
1	0.144942000	-4.194534000	-2.313089000
8	-1.026174000	-6.340705000	2.515526000
1	-1.862711000	-6.600767000	2.047075000
1	-1.211677000	-6.114492000	3.460589000
8	-2.570097000	-2.742689000	7.250888000
1	-2.107395000	-3.135342000	8.067270000
1	-2.620512000	-1.749045000	7.186294000
8	-1.942115000	-6.093634000	5.133420000
1	-2.269963000	-7.005786000	5.236327000
1	-2.716775000	-5.391495000	5.287477000
8	-0.145806000	-5.524800000	6.987259000
1	-0.804421000	-5.780284000	6.266462000
1	0.665574000	-5.169510000	6.586774000
8	-3.729368000	-4.378777000	5.587716000
1	-4.008043000	-3.774719000	4.817346000
1	-3.412344000	-3.779532000	6.347173000
1	3.694851000	1.247364000	7.026667000
1	3.652429000	4.634323000	3.528340000
1	-0.358626000	6.428697000	5.774457000
1	-1.346590000	7.261848000	1.071926000
1	-5.306409000	5.382866000	2.094573000
1	-5.037283000	7.273612000	-3.543604000
1	-2.053808000	6.294412000	-7.723074000
1	0.827396000	9.800414000	-5.542133000
1	6.945894000	4.223680000	-4.217386000
1	4.193142000	3.849095000	0.141822000
1	8.327271000	-1.741571000	-2.340510000
1	9.910737000	1.999401000	0.319669000
1	6.310438000	0.805476000	3.493807000
1	9.098222000	-2.712152000	4.184380000
1	4.422127000	-1.634665000	-8.025135000
1	7.062837000	-3.591082000	-4.515865000
1	-7.299420000	-4.221929000	6.764087000
1	-7.048574000	-0.460474000	3.824037000
1	-4.799784000	-6.704948000	4.026826000
1	-8.810384000	-3.667800000	0.001959000
1	8.399567000	-4.933745000	0.611787000
1	-9.203696000	5.830156000	-3.025253000
1	1.915200000	-0.125353000	-4.529033000
1	3.799157000	-6.939623000	-3.040514000
1	-0.405586000	-4.533961000	-0.760594000
1	4.588572000	-2.142248000	3.994897000

[3]TS2$_B$:

6	8.755636000	-3.057212000	3.151745000
6	8.067279000	-1.901035000	2.440548000
8	8.109375000	-1.828217000	1.176709000
6	7.817942000	-4.291130000	3.346388000
6	6.943843000	-4.603102000	2.171661000
7	7.396426000	-4.907319000	0.879792000
6	5.576386000	-4.587169000	2.075939000
6	6.345294000	-5.048649000	0.033962000
7	5.233669000	-4.859724000	0.756957000
1	9.617012000	-3.334285000	2.536366000
1	8.439559000	-5.154548000	3.616803000
1	7.156121000	-4.106436000	4.198913000
1	4.831502000	-4.394035000	2.826258000
1	6.388984000	-5.240621000	-1.040831000
1	4.221916000	-4.741535000	0.396323000
7	7.413391000	-0.996354000	3.209807000
6	6.512828000	0.028813000	2.671278000
6	7.034065000	0.836589000	1.514520000
8	6.242679000	1.226566000	0.608854000
1	7.276533000	-1.150308000	4.208469000
1	5.559573000	-0.397402000	2.346475000
7	8.341583000	1.176993000	1.438399000
6	8.814341000	1.965347000	0.297516000
6	8.409501000	1.371170000	-1.058896000
8	8.154723000	2.127656000	-2.028774000
1	8.976859000	0.904160000	2.173319000
1	8.403901000	2.978031000	0.322017000
7	8.382292000	0.011848000	-1.155787000
6	8.036770000	-0.634192000	-2.416118000
6	6.550065000	-0.625136000	-2.788365000
8	6.214187000	-0.998094000	-3.944740000
1	8.469125000	-0.569299000	-0.321836000
1	8.558553000	-0.139887000	-3.239898000
7	5.674615000	-0.228394000	-1.838740000
6	4.239230000	-0.051238000	-2.108237000
6	3.884825000	1.445007000	-2.130183000
8	3.299625000	1.956372000	-3.128124000
6	3.345614000	-0.804249000	-1.104100000
6	3.516489000	-2.329500000	-1.141697000
6	2.549301000	-3.026286000	-0.195898000
8	1.514215000	-2.378462000	0.164585000
8	2.799797000	-4.249605000	0.198574000
1	6.029503000	0.086458000	-0.937768000
1	4.070449000	-0.418425000	-3.121468000
1	2.301120000	-0.564995000	-1.329968000
1	3.523152000	-0.431425000	-0.088247000
1	4.545956000	-2.619387000	-0.901621000
1	3.302117000	-2.728182000	-2.144235000
7	4.233615000	2.155398000	-1.031210000
6	3.911720000	3.564497000	-0.851367000
6	4.588934000	4.520764000	-1.820938000
8	4.102800000	5.680251000	-1.972435000
1	4.708133000	1.699340000	-0.253086000
1	2.836049000	3.748189000	-0.962002000
7	5.631611000	4.087214000	-2.564295000
6	6.092630000	4.881650000	-3.702832000
6	4.985413000	5.179025000	-4.727968000
8	5.113776000	6.148279000	-5.510428000
1	6.117029000	3.224185000	-2.340467000
1	6.474418000	5.854977000	-3.383707000
7	3.897827000	4.350929000	-4.729232000
6	2.746648000	4.594586000	-5.594170000
6	1.529078000	5.147631000	-4.829220000
8	0.396896000	5.180536000	-5.388147000
6	2.353268000	3.331171000	-6.377934000
1	3.846403000	3.534516000	-4.118051000
1	3.054757000	5.380751000	-6.295750000
1	1.445031000	3.517971000	-6.954603000
1	3.165566000	3.052737000	-7.056218000
1	2.165158000	2.501509000	-5.687140000
7	1.720699000	5.654959000	-3.580562000
6	0.578401000	6.082514000	-2.765104000
6	-0.170532000	7.285101000	-3.357585000
8	-1.358609000	7.515482000	-3.005479000
6	0.990654000	6.382928000	-1.314971000

8	0.923005000	5.137744000	-0.556236000
1	2.639640000	5.621674000	-3.134148000
1	-0.168278000	5.280821000	-2.737271000
1	2.003393000	6.797314000	-1.280796000
1	0.281991000	7.111779000	-0.902826000
1	1.109936000	5.290095000	0.402224000
7	0.497693000	8.056312000	-4.249607000
6	-0.151486000	9.149937000	-4.964832000
6	-1.235452000	8.731228000	-5.969177000
8	-1.960895000	9.619995000	-6.469901000
1	1.446726000	7.798958000	-4.484139000
1	-0.643676000	9.821310000	-4.255497000
7	-1.357334000	7.402076000	-6.245860000
6	-2.448178000	6.903592000	-7.072188000
6	-3.643871000	6.346240000	-6.293671000
8	-4.565157000	5.759573000	-6.917685000
1	-0.706410000	6.714557000	-5.866887000
1	-2.814324000	7.726443000	-7.693617000
7	-3.647985000	6.534608000	-4.947080000
6	-4.774791000	6.137206000	-4.104439000
6	-4.412498000	5.084889000	-3.043038000
6	-4.039159000	3.696482000	-3.523973000
6	-4.309613000	3.223744000	-4.831844000
6	-3.440036000	2.804306000	-2.597665000
6	-4.008031000	1.917499000	-5.188394000
6	-3.130227000	1.498900000	-2.933139000
6	-3.410924000	1.022165000	-4.250243000
8	-3.125660000	-0.214683000	-4.624286000
1	-2.864813000	6.999336000	-4.497348000
1	-5.564721000	5.781312000	-4.770098000
1	-5.281155000	4.986754000	-2.370461000
1	-3.591311000	5.480986000	-2.427144000
1	-4.735029000	3.888357000	-5.577080000
1	-3.227745000	3.159104000	-1.592294000
1	-4.209701000	1.546634000	-6.186798000
1	-2.671005000	0.817435000	-2.224983000
1	-2.484097000	-1.067792000	-3.968015000
6	-4.677981000	4.554476000	2.437970000
6	-3.199596000	4.809229000	2.287090000
8	-2.333729000	3.918831000	2.516539000
6	-5.216377000	3.508211000	1.403553000
6	-5.957275000	2.342275000	2.081715000
6	-4.966962000	1.416971000	2.796582000
8	-4.053960000	0.830419000	2.152045000
8	-5.159123000	1.363841000	4.100148000
1	-4.827083000	4.155061000	3.449030000
1	-5.897132000	4.003263000	0.703266000
1	-4.390520000	3.090040000	0.818672000
1	-6.479855000	1.724499000	1.345486000
1	-6.706727000	2.708283000	2.789153000
7	-2.789558000	6.061803000	1.909648000
6	-1.392389000	6.297926000	1.533208000
6	-0.386430000	5.947804000	2.627599000
8	0.745386000	5.479810000	2.316312000
1	-3.482137000	6.746392000	1.641049000
1	-1.110900000	5.697726000	0.661937000
7	-0.730202000	6.197941000	3.915478000
6	0.166398000	5.874807000	5.029316000
6	0.254746000	4.374314000	5.364578000
8	1.319014000	3.908738000	5.840814000
1	-1.643363000	6.588721000	4.102337000
1	1.180049000	6.202954000	4.793210000
7	-0.864292000	3.644149000	5.139999000
6	-0.934875000	2.177307000	5.327320000
6	0.065038000	1.510487000	4.358936000
8	0.970112000	0.741486000	4.775445000
6	-2.398481000	1.760740000	5.082951000
6	-2.807370000	0.405946000	5.635742000
8	-2.177013000	-0.138334000	6.592382000
8	-3.871675000	-0.143806000	5.100003000
1	-1.599732000	4.050426000	4.574195000
1	-0.617171000	1.912839000	6.337126000
1	-2.632567000	1.773347000	4.017755000
1	-3.057304000	2.497930000	5.561522000
7	-0.047878000	1.875244000	3.055227000

6	0.861868000	1.354124000	2.027685000
6	2.307074000	1.803334000	2.283544000
8	3.279959000	1.055783000	1.995290000
6	0.394266000	1.788376000	0.620111000
6	-0.983640000	1.242918000	0.170908000
6	-1.025983000	-0.265559000	0.011150000
8	-0.665470000	-0.960773000	1.028959000
8	-1.417156000	-0.757469000	-1.123135000
1	-0.823135000	2.463226000	2.763367000
1	0.864032000	0.264207000	2.075642000
1	1.158424000	1.460137000	-0.093407000
1	0.362925000	2.883300000	0.559018000
1	-1.769750000	1.519140000	0.885965000
1	-1.230240000	1.703316000	-0.788104000
7	2.484640000	3.043768000	2.818017000
6	3.799906000	3.545172000	3.196302000
6	4.521730000	2.679589000	4.230949000
8	5.769654000	2.522513000	4.142747000
1	1.694385000	3.668886000	2.918771000
1	4.479539000	3.589647000	2.340523000
7	3.779437000	2.113302000	5.211809000
6	4.383713000	1.161167000	6.144470000
6	4.568864000	-0.246140000	5.550012000
8	5.699548000	-0.820826000	5.585641000
1	2.793580000	2.340877000	5.304689000
1	5.375334000	1.509579000	6.436588000
7	3.473056000	-0.827815000	5.014946000
6	3.553861000	-2.159464000	4.399625000
6	2.464306000	-2.391810000	3.347360000
6	1.090849000	-2.583450000	3.913099000
7	-0.022228000	-2.781680000	3.089909000
6	0.659471000	-2.568984000	5.226424000
6	-1.090407000	-2.865672000	3.894598000
7	-0.713716000	-2.746792000	5.190804000
1	2.596276000	-0.307612000	4.934524000
1	3.506183000	-2.932829000	5.180766000
1	2.732450000	-3.277532000	2.754817000
1	2.463855000	-1.551626000	2.642045000
1	1.181829000	-2.373445000	6.146813000
1	-2.111844000	-2.981576000	3.577241000
6	1.442302000	-1.088926000	-4.760015000
6	2.312022000	-2.016576000	-5.582832000
8	2.209734000	-3.278340000	-5.517422000
6	0.113969000	-0.694791000	-5.468022000
6	-0.841160000	-1.866469000	-5.798343000
6	-1.232275000	-2.688453000	-4.579526000
8	-1.875285000	-1.995876000	-3.586094000
8	-0.948603000	-3.885231000	-4.464613000
1	1.213455000	-1.596440000	-3.817121000
1	-0.402782000	0.016165000	-4.815889000
1	0.341841000	-0.160607000	-6.399664000
1	-0.387003000	-2.558924000	-6.511258000
1	-1.747399000	-1.444647000	-6.246906000
7	3.208334000	-1.449078000	-6.436602000
6	3.948989000	-2.240224000	-7.425665000
6	4.890390000	-3.310873000	-6.862222000
8	5.129195000	-4.334780000	-7.540897000
1	3.295207000	-0.442877000	-6.453207000
1	3.254983000	-2.777368000	-8.078068000
7	5.438796000	-3.071008000	-5.639102000
6	6.317425000	-4.030432000	-4.987889000
6	5.650918000	-4.804757000	-3.849333000
8	6.351010000	-5.241872000	-2.886297000
1	5.349142000	-2.170579000	-5.174402000
1	6.654843000	-4.750782000	-5.742149000
7	4.327992000	-5.032576000	-3.961239000
6	3.583919000	-5.871373000	-3.012839000
6	2.093909000	-5.920501000	-3.363842000
8	1.540079000	-4.570105000	-3.235937000
6	1.322390000	-6.848377000	-2.430070000
1	3.812687000	-4.576052000	-4.709476000
1	3.678556000	-5.468887000	-1.998721000
1	1.968094000	-6.251017000	-4.404238000
1	1.509449000	-4.113743000	-4.116636000
1	0.261968000	-6.847274000	-2.696208000

1	1.698451000	-7.875253000	-2.500135000
1	1.417764000	-6.510554000	-1.392819000
6	-5.572478000	-6.272739000	3.132286000
6	-6.719792000	-5.492888000	3.758077000
8	-7.788910000	-5.291748000	3.114801000
6	-6.107487000	-7.544103000	2.438631000
6	-5.029183000	-8.338669000	1.657088000
6	-4.285779000	-7.440761000	0.684516000
8	-3.149289000	-6.968999000	0.970316000
7	-4.919310000	-7.085503000	-0.452343000
1	-5.087270000	-5.623591000	2.391380000
1	-6.918621000	-7.241713000	1.767378000
1	-6.555790000	-8.210024000	3.187048000
1	-5.508396000	-9.171597000	1.127026000
1	-4.289393000	-8.761783000	2.343242000
1	-4.530292000	-6.301523000	-0.989916000
1	-5.834254000	-7.441645000	-0.680050000
7	-6.539845000	-5.037676000	5.026558000
6	-7.632773000	-4.402065000	5.761286000
6	-8.172565000	-3.093718000	5.177006000
8	-9.342227000	-2.734505000	5.448057000
1	-5.599904000	-5.028263000	5.429997000
1	-8.492328000	-5.075885000	5.814433000
7	-7.315327000	-2.374418000	4.403844000
6	-7.729257000	-1.147045000	3.745379000
6	-7.785746000	-1.244513000	2.221120000
8	-7.775756000	-0.186086000	1.534493000
1	-6.336073000	-2.648477000	4.287538000
1	-8.731527000	-0.897544000	4.112014000
7	-7.891070000	-2.486553000	1.685579000
6	-8.073389000	-2.714493000	0.251231000
6	-6.870715000	-3.339874000	-0.449735000
8	-5.790568000	-2.367647000	-0.574029000
1	-7.924293000	-3.295768000	2.303636000
1	-8.306012000	-1.753027000	-0.211732000
1	-6.515847000	-4.223710000	0.095079000
1	-7.177447000	-3.656729000	-1.454919000
1	-5.098456000	-2.697245000	-1.220772000
6	-9.645717000	4.882406000	-2.971024000
6	-8.929072000	3.662181000	-2.431536000
6	-9.268828000	3.120259000	-1.179088000
6	-7.929654000	3.021603000	-3.185383000
6	-8.637591000	1.967543000	-0.695959000
6	-7.293526000	1.869608000	-2.705664000
6	-7.644075000	1.337550000	-1.458799000
1	-9.008855000	5.453400000	-3.655871000
1	-10.548589000	4.599838000	-3.529969000
1	-10.040525000	3.602311000	-0.583177000
1	-7.654816000	3.422645000	-4.157973000
1	-8.904029000	1.546846000	0.267527000
1	-6.526069000	1.390861000	-3.305638000
1	-7.157515000	0.446283000	-1.075416000
26	-0.310843000	-2.899916000	1.003300000
26	-2.017339000	-2.621237000	-1.668222000
8	0.181075000	-4.829981000	0.839438000
1	1.167635000	-4.894270000	0.765630000
1	-0.288772000	-5.464659000	1.541209000
8	-4.483424000	-2.488239000	3.874855000
1	-4.289887000	-1.638383000	4.354138000
1	-4.404084000	-2.332575000	2.871298000
8	-4.196501000	-1.769860000	1.394909000
1	-4.916964000	-1.949902000	0.699132000
1	-4.062677000	-0.799162000	1.552839000
8	-2.145472000	-3.147862000	0.250663000
1	-3.143381000	-4.346880000	-0.268750000
1	-2.822124000	-2.577330000	0.720991000
8	-0.949093000	-4.098840000	8.654493000
1	-0.549108000	-4.722946000	7.959144000
1	-0.953273000	-4.470884000	9.549029000
8	-3.731191000	-3.478059000	-1.925357000
8	-3.873473000	-4.603765000	-0.935304000
8	-0.534205000	-4.016523000	-1.646558000
1	0.118714000	-4.254413000	-2.360906000
8	-1.062865000	-6.283105000	2.426656000
1	-1.839032000	-6.659099000	1.934849000
1	-1.306918000	-6.073068000	3.363320000
8	-2.552991000	-2.756393000	7.222944000
1	-2.000624000	-3.174013000	7.967986000
1	-2.587643000	-1.761445000	7.197623000
8	-2.021647000	-6.065940000	5.006591000
1	-2.360681000	-6.971632000	5.126508000
1	-2.773576000	-5.347391000	5.197359000
8	-0.056766000	-5.506398000	6.686791000
1	-0.783891000	-5.771396000	6.039819000
1	0.662290000	-5.061634000	6.204527000
8	-3.759914000	-4.327356000	5.541218000
1	-4.039762000	-3.692868000	4.794410000
1	-3.424362000	-3.757592000	6.315289000
1	3.744516000	1.101634000	7.030241000
1	3.668366000	4.557803000	3.588890000
1	-0.173431000	6.419532000	5.915254000
1	-1.285937000	7.355420000	1.269886000
1	-5.244415000	5.490178000	2.373856000
1	-5.158936000	7.025517000	-3.586452000
1	-2.088319000	6.109073000	-7.731076000
1	0.612675000	9.723906000	-5.495395000
1	6.906500000	4.335661000	-4.187579000
1	4.183763000	3.840848000	0.170821000
1	8.370175000	-1.675495000	-2.379357000
1	9.905224000	2.031164000	0.353197000
1	6.297365000	0.737852000	3.478683000
1	9.133652000	-2.753176000	4.133696000
1	4.529090000	-1.546647000	-8.042289000
1	7.186247000	-3.516697000	-4.573647000
1	-7.284976000	-4.214064000	6.782192000
1	-7.058178000	-0.322561000	4.001155000
1	-4.811006000	-6.522293000	3.879324000
1	-8.932499000	-3.383973000	0.115371000
1	8.360500000	-4.923167000	0.585748000
1	-9.963176000	5.552674000	-2.163961000
1	1.988232000	-0.169566000	-4.512098000
1	4.008933000	-6.883143000	-3.017301000
1	-0.406397000	-4.546178000	-0.819496000
1	4.542656000	-2.242261000	3.937024000

$^{3}IM2_{B}$:

6	8.697180000	-2.898846000	2.628005000
6	7.919529000	-1.744215000	2.013322000
8	7.844145000	-1.648718000	0.752953000
6	7.863244000	-4.218034000	2.727548000
6	6.950702000	-4.480956000	1.570346000
7	7.349871000	-4.718278000	0.246552000
6	5.580972000	-4.470613000	1.533657000
6	6.263825000	-4.826024000	-0.560235000
7	5.185516000	-4.680945000	0.219346000
1	9.566748000	-3.061153000	1.983242000
1	8.560297000	-5.052175000	2.878306000
1	7.236192000	-4.175696000	3.624198000
1	4.867223000	-4.321465000	2.323505000
1	6.237418000	-4.971932000	-1.641690000
1	4.160911000	-4.572052000	-0.083668000
7	7.329052000	-0.856400000	2.851614000
6	6.401734000	0.186959000	2.395730000
6	6.870516000	1.028914000	1.238028000
8	6.035796000	1.470094000	0.397595000
1	7.277716000	-1.026566000	3.854826000
1	5.434487000	-0.230064000	2.101359000
7	8.183407000	1.331407000	1.100110000
6	8.626997000	2.129757000	-0.043982000
6	8.140679000	1.581131000	-1.392452000
8	7.885158000	2.372817000	-2.334398000
1	8.846345000	1.015918000	1.792203000
1	8.252095000	3.154404000	0.021323000
7	8.034034000	0.229225000	-1.514058000
6	7.591513000	-0.375397000	-2.764605000
6	6.079225000	-0.392617000	-3.015647000
8	5.660147000	-0.847095000	-4.112575000
1	8.133810000	-0.374482000	-0.697570000
1	8.032650000	0.167611000	-3.605154000
7	5.272594000	0.084836000	-2.040298000

6	3.821692000	0.262308000	-2.234262000
6	3.496752000	1.763104000	-2.146746000
8	3.017814000	2.391715000	-3.133036000
6	2.973353000	-0.572964000	-1.250280000
6	3.210183000	-2.086383000	-1.362860000
6	2.355810000	-2.892480000	-0.390362000
8	1.321839000	-2.336676000	0.109720000
8	2.701036000	-4.122237000	-0.116686000
1	5.682817000	0.441987000	-1.178331000
1	3.609766000	-0.031238000	-3.263943000
1	1.915806000	-0.366312000	-1.449597000
1	3.155312000	-0.246607000	-0.220267000
1	4.266420000	-2.328693000	-1.197271000
1	2.970414000	-2.446488000	-2.374528000
7	3.781300000	2.372615000	-0.970321000
6	3.502677000	3.782737000	-0.744048000
6	4.368660000	4.764347000	-1.517367000
8	3.999921000	5.974989000	-1.584239000
1	4.191094000	1.854006000	-0.196024000
1	2.468474000	4.025496000	-1.014436000
7	5.456991000	4.312044000	-2.174504000
6	6.163296000	5.168197000	-3.125598000
6	5.262677000	5.701745000	-4.246732000
8	5.597209000	6.734371000	-4.870541000
1	5.807693000	3.372385000	-2.031106000
1	6.581259000	6.050132000	-2.632837000
7	4.110759000	5.010007000	-4.506501000
6	3.136484000	5.490849000	-5.484008000
6	1.833357000	5.969868000	-4.818889000
8	0.778991000	6.097716000	-5.501669000
6	2.851574000	4.442677000	-6.571277000
1	3.903259000	4.132188000	-4.033385000
1	3.597136000	6.374889000	-5.945636000
1	2.061633000	4.796895000	-7.237002000
1	3.761434000	4.264870000	-7.151703000
1	2.529660000	3.493753000	-6.130794000
7	1.867948000	6.315670000	-3.498721000
6	0.622998000	6.605542000	-2.777803000
6	-0.102094000	7.862819000	-3.271282000
8	-1.329812000	8.001873000	-3.018076000
6	0.825852000	6.670946000	-1.254285000
8	0.573431000	5.339268000	-0.701749000
1	2.722801000	6.199067000	-2.949862000
1	-0.090409000	5.799179000	-2.974740000
1	1.843561000	6.994699000	-1.014478000
1	0.104429000	7.383760000	-0.837327000
1	0.695627000	5.341206000	0.279844000
7	0.609031000	8.770243000	-3.980735000
6	-0.031912000	9.922828000	-4.608148000
6	-1.049893000	9.586460000	-5.709358000
8	-1.836985000	10.481648000	-6.091656000
1	1.587082000	8.581169000	-4.151936000
1	-0.582222000	10.502514000	-3.862247000
7	-1.038530000	8.316727000	-6.203863000
6	-2.051240000	7.858585000	-7.145864000
6	-2.990690000	6.792224000	-6.580135000
8	-3.642604000	6.055436000	-7.363343000
1	-0.343735000	7.633421000	-5.901163000
1	-2.641670000	8.730335000	-7.447450000
7	-3.080599000	6.708900000	-5.223374000
6	-3.933987000	5.723995000	-4.563351000
6	-3.141153000	4.700010000	-3.725496000
6	-2.117115000	3.860109000	-4.460763000
6	-2.240230000	3.537686000	-5.839573000
6	-1.000347000	3.354189000	-3.736918000
6	-1.306766000	2.731314000	-6.460625000
6	-0.051981000	2.552921000	-4.339340000
6	-0.187125000	2.190427000	-5.729875000
8	0.658434000	1.393010000	-6.308668000
1	-2.526703000	7.326820000	-4.636320000
1	-4.522405000	5.238153000	-5.345586000
1	-3.870252000	4.025055000	-3.244910000
1	-2.636774000	5.232339000	-2.907892000
1	-3.050664000	3.958924000	-6.424407000
1	-0.883498000	3.627191000	-2.691211000

1	-1.387000000	2.482113000	-7.512893000
1	0.829589000	2.211682000	-3.807337000
1	-3.599095000	-3.618478000	-2.991646000
6	-4.961193000	4.154368000	2.539170000
6	-3.516881000	4.511471000	2.290791000
8	-2.579086000	3.683496000	2.464673000
6	-5.477684000	3.010114000	1.605651000
6	-6.181851000	1.890315000	2.394740000
6	-5.150406000	1.033539000	3.136718000
8	-4.253602000	0.414878000	2.499953000
8	-5.282792000	1.075864000	4.448364000
1	-5.019012000	3.806381000	3.579090000
1	-6.172602000	3.422538000	0.866974000
1	-4.645741000	2.562858000	1.052715000
1	-6.716442000	1.212691000	1.723252000
1	-6.912707000	2.296141000	3.099883000
7	-3.215810000	5.791873000	1.905601000
6	-1.854575000	6.139975000	1.483748000
6	-0.788840000	5.828915000	2.533897000
8	0.349709000	5.411726000	2.179426000
1	-3.967144000	6.429013000	1.682146000
1	-1.562713000	5.597101000	0.579063000
7	-1.090585000	6.066877000	3.835368000
6	-0.130206000	5.801837000	4.911003000
6	0.059005000	4.311659000	5.248315000
8	1.170658000	3.910410000	5.674036000
1	-2.010960000	6.418779000	4.060219000
1	0.852131000	6.186455000	4.631317000
7	-1.025301000	3.517793000	5.082521000
6	-1.005489000	2.051022000	5.280611000
6	-0.035493000	1.428722000	4.253605000
8	0.923245000	0.696478000	4.613646000
6	-2.454059000	1.549048000	5.132675000
6	-2.778010000	0.241602000	5.835087000
8	-2.021271000	-0.242712000	6.730052000
8	-3.903366000	-0.336711000	5.484583000
1	-1.812089000	3.881452000	4.559250000
1	-0.612046000	1.813053000	6.270200000
1	-2.723646000	1.431481000	4.080735000
1	-3.138339000	2.297370000	5.553175000
7	-0.236039000	1.795367000	2.960646000
6	0.651219000	1.330904000	1.888747000
6	2.088441000	1.829284000	2.120690000
8	3.081368000	1.127750000	1.782894000
6	0.121803000	1.769188000	0.499276000
6	-1.255634000	1.197012000	0.062435000
6	-1.320228000	-0.319564000	0.019734000
8	-0.957320000	-0.940069000	1.077852000
8	-1.747557000	-0.906391000	-1.063015000
1	-1.057435000	2.338854000	2.711246000
1	0.699424000	0.241819000	1.915203000
1	0.878287000	1.475471000	-0.236288000
1	0.059073000	2.864425000	0.462850000
1	-2.041837000	1.527759000	0.753926000
1	-1.499778000	1.592114000	-0.927526000
7	2.235803000	3.055462000	2.691979000
6	3.534806000	3.603531000	3.070369000
6	4.325716000	2.747900000	4.060400000
8	5.571312000	2.625797000	3.910652000
1	1.420703000	3.638114000	2.835681000
1	4.198130000	3.712156000	2.208745000
7	3.652605000	2.159343000	5.077102000
6	4.348885000	1.242595000	5.980501000
6	4.606612000	-0.141436000	5.362268000
8	5.762068000	-0.661770000	5.383686000
1	2.665138000	2.353113000	5.218120000
1	5.324315000	1.652754000	6.246305000
7	3.537834000	-0.766747000	4.819988000
6	3.678681000	-2.091661000	4.203241000
6	2.526057000	-2.405110000	3.246497000
6	1.212112000	-2.633318000	3.926936000
7	0.042193000	-2.853275000	3.194920000
6	0.884375000	-2.625889000	5.269227000
6	-0.959660000	-2.956698000	4.081260000
7	-0.483351000	-2.830595000	5.340760000

1	2.638164000	-0.287243000	4.746748000
1	3.752544000	-2.859336000	4.987689000
1	2.786755000	-3.293871000	2.654332000
1	2.431303000	-1.583284000	2.525712000
1	1.473437000	-2.423619000	6.146716000
1	-1.999925000	-3.096565000	3.844626000
6	0.181616000	-1.489182000	-4.400856000
6	1.165112000	-2.054759000	-5.403478000
8	1.299610000	-3.318159000	-5.548483000
6	-1.226085000	-1.253569000	-5.016494000
6	-1.891719000	-2.487373000	-5.647111000
6	-2.527728000	-3.501666000	-4.723590000
8	-2.966619000	-2.987473000	-3.514390000
8	-2.742927000	-4.672967000	-5.037922000
1	0.113675000	-2.186428000	-3.560558000
1	-1.867985000	-0.850352000	-4.227354000
1	-1.133280000	-0.484917000	-5.790432000
1	-1.204437000	-3.037035000	-6.297415000
1	-2.712022000	-2.139394000	-6.293498000
7	1.866110000	-1.194373000	-6.168920000
6	2.676648000	-1.653168000	-7.307734000
6	3.695210000	-2.756754000	-6.997554000
8	3.870915000	-3.688726000	-7.813274000
1	1.638459000	-0.196281000	-6.110713000
1	2.037974000	-2.062949000	-8.095790000
7	4.392949000	-2.644408000	-5.829514000
6	5.363415000	-3.645983000	-5.409047000
6	4.870931000	-4.535952000	-4.265496000
8	5.688915000	-4.993522000	-3.410455000
1	4.384796000	-1.790047000	-5.280680000
1	5.590503000	-4.281667000	-6.272872000
7	3.556404000	-4.837465000	-4.257080000
6	2.962242000	-5.767236000	-3.285203000
6	1.513501000	-6.123512000	-3.647700000
8	0.674580000	-4.928731000	-3.575471000
6	0.929950000	-7.157182000	-2.688697000
1	2.945318000	-4.374723000	-4.928072000
1	2.965956000	-5.324207000	-2.282256000
1	1.475233000	-6.506024000	-4.677320000
1	0.770241000	-4.346028000	-4.384018000
1	-0.113289000	-7.355132000	-2.948699000
1	1.489006000	-8.097851000	-2.741639000
1	0.961920000	-6.790663000	-1.656489000
6	-5.300134000	-6.547352000	3.803240000
6	-6.459055000	-5.836272000	4.486590000
8	-7.583035000	-5.739623000	3.916777000
6	-5.770848000	-7.880019000	3.184870000
6	-4.702854000	-8.568904000	2.295632000
6	-4.210166000	-7.631231000	1.209104000
8	-3.125707000	-6.991833000	1.334433000
7	-5.013641000	-7.426765000	0.144815000
1	-4.921009000	-5.884911000	3.013865000
1	-6.677052000	-7.679481000	2.602795000
1	-6.060293000	-8.574874000	3.983638000
1	-5.130550000	-9.478625000	1.855536000
1	-3.835759000	-8.864328000	2.894073000
1	-4.758376000	-6.671915000	-0.501283000
1	-5.883619000	-7.922476000	0.032656000
7	-6.221124000	-5.316171000	5.719292000
6	-7.296917000	-4.715026000	6.506657000
6	-7.965593000	-3.473119000	5.907736000
8	-9.133929000	-3.177861000	6.251518000
1	-5.257257000	-5.248381000	6.054344000
1	-8.103660000	-5.437851000	6.656734000
7	-7.217914000	-2.738340000	5.040390000
6	-7.758095000	-1.566237000	4.369960000
6	-7.928445000	-1.731495000	2.858662000
8	-8.032695000	-0.706726000	2.130633000
1	-6.231642000	-2.956480000	4.871263000
1	-8.741941000	-1.361086000	4.807038000
7	-8.003488000	-3.003459000	2.389699000
6	-8.249769000	-3.315606000	0.982776000
6	-7.065881000	-3.973110000	0.278225000
8	-5.981803000	-3.020647000	0.111067000
1	-7.940837000	-3.780225000	3.045608000
1	-8.503389000	-2.382129000	0.475838000
1	-6.717883000	-4.844973000	0.848900000
1	-7.393451000	-4.319453000	-0.711120000
1	-5.320494000	-3.307081000	-0.607426000
6	-8.010751000	3.863271000	-3.229121000
6	-7.497510000	2.589448000	-2.590484000
6	-8.136654000	2.037356000	-1.465829000
6	-6.392048000	1.907319000	-3.130104000
6	-7.694835000	0.833493000	-0.901103000
6	-5.943789000	0.705208000	-2.568104000
6	-6.593987000	0.162877000	-1.452395000
1	-8.731166000	3.645927000	-4.029632000
1	-8.522172000	4.500788000	-2.499248000
1	-8.995523000	2.549444000	-1.038043000
1	-5.891155000	2.313103000	-4.006463000
1	-8.192954000	0.412699000	-0.032333000
1	-5.091119000	0.189857000	-3.001515000
1	-6.262547000	-0.773246000	-1.013551000
26	-0.404033000	-2.981375000	1.139514000
26	-2.295989000	-2.845964000	-1.307514000
8	0.254841000	-5.007477000	0.767036000
1	1.227466000	-4.982863000	0.588345000
1	-0.094232000	-5.636485000	1.519363000
8	-4.406008000	-2.763423000	4.395194000
1	-4.250166000	-1.882055000	4.830496000
1	-4.388438000	-2.652611000	3.381045000
8	-4.303869000	-2.209770000	1.876697000
1	-5.076111000	-2.480134000	1.264581000
1	-4.205723000	-1.224575000	1.954903000
8	-2.264018000	-3.415376000	0.588573000
1	-3.215946000	-4.827456000	-0.060339000
1	-2.981267000	-2.957301000	1.122969000
8	-0.354772000	-4.080980000	8.824219000
1	-0.011315000	-4.722819000	8.114930000
1	-0.282674000	-4.432426000	9.724432000
8	-4.088638000	-3.781588000	-1.491922000
8	-3.862091000	-5.110663000	-0.778541000
8	-0.813793000	-4.201353000	-1.578802000
1	-0.303940000	-4.504172000	-2.392858000
8	-0.826283000	-6.425525000	2.550398000
1	-1.673629000	-6.747333000	2.143951000
1	-0.983170000	-6.158730000	3.488988000
8	-2.134497000	-2.849011000	7.506824000
1	-1.501861000	-3.210988000	8.215880000
1	-2.245990000	-1.861243000	7.458662000
8	-1.613501000	-6.153717000	5.236334000
1	-1.897827000	-7.080293000	5.338398000
1	-2.387834000	-5.489250000	5.504735000
8	0.398103000	-5.543995000	6.835879000
1	-0.352551000	-5.825517000	6.221673000
1	1.111049000	-5.137912000	6.313267000
8	-3.405521000	-4.532546000	5.971048000
1	-3.799749000	-3.910493000	5.262095000
1	-3.030115000	-3.942579000	6.706515000
1	3.743752000	1.130904000	6.884944000
1	3.358886000	4.593256000	3.501531000
1	-0.463261000	6.331731000	5.808450000
1	-1.834803000	7.211051000	1.255978000
1	-5.603218000	5.038997000	2.461324000
1	-4.637176000	6.238816000	-3.896494000
1	-1.591933000	7.432372000	-8.042003000
1	0.744983000	10.566359000	-5.029860000
1	6.986271000	4.584104000	-3.544812000
1	3.613920000	3.985679000	0.321886000
1	7.947619000	-1.408469000	-2.805761000
1	9.720867000	2.160909000	-0.039374000
1	6.221843000	0.867632000	3.235438000
1	9.067749000	-2.648470000	3.627526000
1	3.196974000	-0.777015000	-7.707471000
1	6.276120000	-3.153766000	-5.072795000
1	-6.888056000	-4.454638000	7.488279000
1	-7.121603000	-0.693634000	4.539964000
1	-4.471504000	-6.709205000	4.500559000
1	-9.110773000	-3.993738000	0.916983000
1	8.301693000	-4.718868000	-0.085118000

1	-7.198881000	4.446575000	-3.679066000
1	0.546538000	-0.533399000	-4.011040000
1	3.574218000	-6.676666000	-3.254744000
1	-0.533900000	-4.694631000	-0.758745000
1	4.629412000	-2.109241000	3.659934000

^{3}TS3$_{B}$:

6	8.590754000	-2.778246000	2.448533000
6	7.820951000	-1.614728000	1.840706000
8	7.749049000	-1.507733000	0.581159000
6	7.739483000	-4.085759000	2.556595000
6	6.812791000	-4.332195000	1.407205000
7	7.197970000	-4.576349000	0.080750000
6	5.443387000	-4.292438000	1.379502000
6	6.104792000	-4.660187000	-0.719070000
7	5.034386000	-4.492703000	0.067544000
1	9.451875000	-2.951937000	1.795643000
1	8.425759000	-4.930112000	2.699426000
1	7.121950000	-4.035149000	3.459378000
1	4.738755000	-4.127849000	2.174299000
1	6.073787000	-4.806167000	-1.801324000
1	4.008647000	-4.362749000	-0.228743000
7	7.230463000	-0.732821000	2.685458000
6	6.303075000	0.313161000	2.237202000
6	6.769757000	1.163393000	1.085414000
8	5.932292000	1.613347000	0.252209000
1	7.176930000	-0.911480000	3.686998000
1	5.335681000	-0.101997000	1.940389000
7	8.081667000	1.466550000	0.943710000
6	8.519051000	2.276952000	-0.194529000
6	8.030789000	1.739402000	-1.546870000
8	7.767060000	2.539291000	-2.479759000
1	8.748487000	1.141905000	1.627777000
1	8.140273000	3.299379000	-0.118280000
7	7.931790000	0.388217000	-1.681710000
6	7.492274000	-0.208072000	-2.937912000
6	5.980755000	-0.215212000	-3.194313000
8	5.559726000	-0.664589000	-4.292504000
1	8.037014000	-0.222509000	-0.871145000
1	7.939911000	0.336445000	-3.774115000
7	5.177507000	0.267833000	-2.219035000
6	3.729817000	0.463306000	-2.413646000
6	3.416981000	1.965299000	-2.309629000
8	2.917804000	2.602531000	-3.280028000
6	2.870885000	-0.369689000	-1.438426000
6	3.105756000	-1.884445000	-1.544356000
6	2.216645000	-2.676396000	-0.592271000
8	1.161203000	-2.111631000	-0.154062000
8	2.556055000	-3.898536000	-0.269701000
1	5.591089000	0.611886000	-1.353903000
1	3.515407000	0.181497000	-3.446112000
1	1.815893000	-0.161910000	-1.649037000
1	3.042879000	-0.043251000	-0.406582000
1	4.155340000	-2.130585000	-1.346549000
1	2.893755000	-2.243657000	-2.562550000
7	3.730835000	2.565399000	-1.135737000
6	3.465635000	3.975911000	-0.895500000
6	4.299269000	4.956212000	-1.705885000
8	3.916706000	6.162491000	-1.775080000
1	4.162022000	2.040506000	-0.377443000
1	2.420670000	4.220560000	-1.119441000
7	5.375105000	4.508707000	-2.386687000
6	6.047328000	5.364762000	-3.362523000
6	5.114916000	5.878835000	-4.467419000
8	5.427185000	6.907048000	-5.109977000
1	5.743568000	3.575334000	-2.243290000
1	6.467436000	6.255574000	-2.887879000
7	3.963360000	5.175415000	-4.691688000
6	2.961280000	5.637049000	-5.650279000
6	1.669847000	6.108473000	-4.957847000
8	0.598299000	6.221861000	-5.615818000
6	2.660241000	4.575008000	-6.719774000
1	3.772134000	4.303006000	-4.201603000
1	3.401248000	6.521053000	-6.131602000
1	1.852046000	4.915491000	-7.370588000
1	3.557894000	4.398596000	-7.319315000
1	2.356709000	3.628278000	-6.261837000
7	1.732319000	6.461948000	-3.640697000
6	0.501744000	6.738473000	-2.890567000
6	-0.246400000	7.990241000	-3.363220000
8	-1.466750000	8.121181000	-3.072322000
6	0.738531000	6.799910000	-1.371736000
8	0.493649000	5.467272000	-0.816798000
1	2.601884000	6.358689000	-3.112935000
1	-0.208504000	5.926153000	-3.074749000
1	1.762557000	7.118954000	-1.153540000
1	0.029207000	7.514020000	-0.936765000
1	0.621468000	5.470591000	0.164276000
7	0.435550000	8.899792000	-4.097930000
6	-0.234220000	10.042355000	-4.713305000
6	-1.278178000	9.688247000	-5.784204000
8	-2.086770000	10.571671000	-6.148599000
1	1.409657000	8.717749000	-4.296996000
1	-0.769899000	10.620428000	-3.955661000
7	-1.263897000	8.416471000	-6.273349000
6	-2.295051000	7.941227000	-7.186375000
6	-3.208569000	6.868677000	-6.590591000
8	-3.878299000	6.125641000	-7.352437000
1	-0.552042000	7.743501000	-5.987394000
1	-2.902564000	8.804320000	-7.478637000
7	-3.257062000	6.787318000	-5.231540000
6	-4.084979000	5.798462000	-4.545586000
6	-3.264236000	4.776885000	-3.731805000
6	-2.257987000	3.942539000	-4.497403000
6	-2.428856000	3.607626000	-5.868226000
6	-1.110352000	3.453412000	-3.811673000
6	-1.511417000	2.804942000	-6.517284000
6	-0.176881000	2.656890000	-4.443043000
6	-0.359243000	2.282153000	-5.824925000
8	0.475106000	1.492205000	-6.429193000
1	-2.693643000	7.413076000	-4.662359000
1	-4.693832000	5.309832000	-5.310409000
1	-3.977611000	4.097739000	-3.234104000
1	-2.739579000	5.309715000	-2.927452000
1	-3.263653000	4.017493000	-6.426161000
1	-0.956846000	3.736115000	-2.773248000
1	-1.628540000	2.546052000	-7.563714000
1	0.727110000	2.329499000	-3.940928000
1	-3.837029000	-3.506550000	-2.839531000
6	-5.031147000	4.341950000	2.384820000
6	-3.582411000	4.698719000	2.166602000
8	-2.648647000	3.871100000	2.364749000
6	-5.532973000	3.222349000	1.412466000
6	-6.234403000	2.073903000	2.161597000
6	-5.205996000	1.217456000	2.908264000
8	-4.311105000	0.589856000	2.278229000
8	-5.340218000	1.273088000	4.220036000
1	-5.107800000	3.968067000	3.414043000
1	-6.227920000	3.652282000	0.683709000
1	-4.695219000	2.799823000	0.848676000
1	-6.744776000	1.404328000	1.464498000
1	-6.986392000	2.453174000	2.858901000
7	-3.274053000	5.974179000	1.772824000
6	-1.909084000	6.314668000	1.356208000
6	-0.848058000	6.002382000	2.410491000
8	0.284451000	5.564822000	2.061281000
1	-4.021799000	6.609918000	1.533695000
1	-1.616985000	5.766927000	0.454542000
7	-1.144135000	6.262629000	3.709112000
6	-0.182452000	6.002952000	4.785211000
6	-0.007190000	4.515753000	5.143165000
8	1.100690000	4.106797000	5.569453000
1	-2.058738000	6.630742000	3.931514000
1	0.802677000	6.373682000	4.496809000
7	-1.102624000	3.732782000	4.993556000
6	-1.104856000	2.269824000	5.217385000
6	-0.148584000	1.610688000	4.199764000
8	0.809845000	0.884145000	4.572841000
6	-2.564919000	1.799330000	5.080547000
6	-2.882113000	0.442246000	5.684841000

8	-2.148142000	-0.067373000	6.584978000
8	-3.974224000	-0.144777000	5.256341000
1	-1.885691000	4.099631000	4.466934000
1	-0.713263000	2.041670000	6.209788000
1	-2.867479000	1.780323000	4.032950000
1	-3.217681000	2.522578000	5.587769000
7	-0.360056000	1.941463000	2.899441000
6	0.509509000	1.446404000	1.825776000
6	1.954413000	1.938274000	2.022282000
8	2.935899000	1.226604000	1.673898000
6	-0.047069000	1.864941000	0.442147000
6	-1.427776000	1.269633000	0.051905000
6	-1.434332000	-0.240287000	-0.088544000
8	-1.015768000	-0.909681000	0.926217000
8	-1.858492000	-0.765558000	-1.197554000
1	-1.171986000	2.497051000	2.645273000
1	0.547675000	0.356807000	1.870079000
1	0.694622000	1.572165000	-0.308763000
1	-0.123261000	2.959323000	0.398118000
1	-2.187853000	1.527018000	0.800948000
1	-1.740420000	1.705012000	-0.901062000
7	2.119030000	3.169490000	2.578772000
6	3.426883000	3.712973000	2.930153000
6	4.227001000	2.863612000	3.918069000
8	5.471913000	2.744527000	3.759694000
1	1.311202000	3.760767000	2.727250000
1	4.077589000	3.806211000	2.057397000
7	3.562596000	2.275074000	4.940321000
6	4.262545000	1.350624000	5.832762000
6	4.503496000	-0.030646000	5.201670000
8	5.654473000	-0.560980000	5.207651000
1	2.573997000	2.459196000	5.083093000
1	5.242999000	1.753642000	6.090388000
7	3.423982000	-0.639365000	4.661491000
6	3.545991000	-1.952855000	4.016740000
6	2.402200000	-2.221733000	3.035086000
6	1.076612000	-2.444620000	3.694340000
7	-0.084313000	-2.665719000	2.948480000
6	0.733390000	-2.432505000	5.033488000
6	-1.095634000	-2.765744000	3.822286000
7	-0.635203000	-2.634264000	5.089334000
1	2.528127000	-0.150739000	4.607948000
1	3.590381000	-2.739928000	4.784128000
1	2.656194000	-3.101020000	2.426471000
1	2.333002000	-1.380861000	2.333158000
1	1.311036000	-2.225415000	5.917544000
1	-2.132138000	-2.907387000	3.573103000
6	0.022885000	-1.404970000	-4.579636000
6	1.023115000	-1.959751000	-5.570729000
8	1.190896000	-3.220395000	-5.704584000
6	-1.383289000	-1.192412000	-5.209353000
6	-2.057028000	-2.453884000	-5.767907000
6	-2.723966000	-3.401259000	-4.780626000
8	-3.030160000	-2.885437000	-3.557344000
8	-3.057637000	-4.547374000	-5.109737000
1	-0.043451000	-2.101184000	-3.738394000
1	-2.023566000	-0.748392000	-4.440690000
1	-1.283797000	-0.460491000	-6.018195000
1	-1.367979000	-3.051561000	-6.374231000
1	-2.862422000	-2.142682000	-6.449926000
7	1.708608000	-1.088891000	-6.339804000
6	2.526590000	-1.534618000	-7.478037000
6	3.571105000	-2.612536000	-7.165954000
8	3.763412000	-3.546548000	-7.975447000
1	1.463395000	-0.095098000	-6.282380000
1	1.895607000	-1.963148000	-8.262304000
7	4.273891000	-2.477460000	-6.002536000
6	5.263459000	-3.459590000	-5.581099000
6	4.788905000	-4.354867000	-4.434680000
8	5.619301000	-4.806187000	-3.587839000
1	4.252319000	-1.619716000	-5.460009000
1	5.499080000	-4.094143000	-6.443473000
7	3.477234000	-4.667742000	-4.414268000
6	2.901784000	-5.609028000	-3.443170000
6	1.479257000	-6.034686000	-3.834509000
8	0.580055000	-4.885599000	-3.773534000
6	0.929664000	-7.100181000	-2.890310000
1	2.854229000	-4.209624000	-5.078166000
1	2.860319000	-5.154045000	-2.445752000
1	1.479780000	-6.414190000	-4.865737000
1	0.666192000	-4.283719000	-4.569768000
1	-0.102849000	-7.335655000	-3.161129000
1	1.526044000	-8.017392000	-2.946528000
1	0.939390000	-6.742503000	-1.854217000
6	-5.414080000	-6.310612000	3.566560000
6	-6.569695000	-5.606542000	4.262519000
8	-7.694766000	-5.500487000	3.695691000
6	-5.883029000	-7.647452000	2.955823000
6	-4.813824000	-8.338126000	2.070198000
6	-4.328479000	-7.413726000	0.968964000
8	-3.235550000	-6.784417000	1.069166000
7	-5.144014000	-7.214701000	-0.087287000
1	-5.049628000	-5.646589000	2.771497000
1	-6.790873000	-7.453192000	2.374125000
1	-6.169055000	-8.338750000	3.758891000
1	-5.236732000	-9.255578000	1.641560000
1	-3.943079000	-8.621814000	2.668987000
1	-4.882819000	-6.480446000	-0.752142000
1	-6.015488000	-7.709886000	-0.188844000
7	-6.328287000	-5.105699000	5.502081000
6	-7.399042000	-4.510019000	6.300173000
6	-8.050568000	-3.246155000	5.728447000
8	-9.210496000	-2.936553000	6.087890000
1	-5.363066000	-5.045970000	5.835976000
1	-8.215038000	-5.226345000	6.429943000
7	-7.299925000	-2.512683000	4.862747000
6	-7.830812000	-1.329901000	4.202996000
6	-8.026340000	-1.495303000	2.694466000
8	-8.103421000	-0.473324000	1.959082000
1	-6.320214000	-2.745724000	4.676865000
1	-8.804201000	-1.109031000	4.655536000
7	-8.152445000	-2.767404000	2.236741000
6	-8.421694000	-3.081493000	0.835647000
6	-7.267203000	-3.792927000	0.136099000
8	-6.140469000	-2.888220000	-0.026430000
1	-8.094293000	-3.543719000	2.893610000
1	-8.641185000	-2.143515000	0.321217000
1	-6.960907000	-4.675701000	0.713682000
1	-7.604526000	-4.126499000	-0.854091000
1	-5.528548000	-3.161424000	-0.777937000
6	-7.849409000	3.795129000	-3.814523000
6	-7.423865000	2.602274000	-2.984167000
6	-8.178521000	2.198060000	-1.867559000
6	-6.289049000	1.847117000	-3.330083000
6	-7.821510000	1.064276000	-1.127187000
6	-5.924693000	0.714097000	-2.590530000
6	-6.691392000	0.316632000	-1.488282000
1	-8.600278000	3.510672000	-4.564344000
1	-8.297196000	4.579849000	-3.193777000
1	-9.060223000	2.769500000	-1.586388000
1	-5.697698000	2.138862000	-4.195159000
1	-8.409253000	0.752836000	-0.268725000
1	-5.047560000	0.140284000	-2.876557000
1	-6.428358000	-0.569502000	-0.919367000
26	-0.489958000	-2.813874000	0.896332000
26	-2.268262000	-2.700791000	-1.575549000
8	0.126089000	-4.703279000	0.676617000
1	1.101891000	-4.700604000	0.503135000
1	-0.263417000	-5.352316000	1.414232000
8	-4.495045000	-2.568709000	4.175621000
1	-4.326334000	-1.687668000	4.607323000
1	-4.459905000	-2.465511000	3.162224000
8	-4.367574000	-2.037947000	1.644454000
1	-5.162432000	-2.322439000	1.076953000
1	-4.283705000	-1.051424000	1.718430000
8	-2.333423000	-3.275872000	0.313009000
1	-3.280649000	-4.638929000	-0.361851000
1	-3.030317000	-2.773883000	0.829689000
8	-0.403477000	-3.894177000	8.545496000
1	-0.066577000	-4.532201000	7.830050000

1	-0.281684000	-4.229717000	9.446368000
8	-4.278019000	-3.609824000	-1.710720000
8	-3.910885000	-4.949169000	-1.093366000
8	-0.846108000	-4.107815000	-1.763827000
1	-0.348403000	-4.446453000	-2.573319000
8	-0.992601000	-6.166423000	2.349840000
1	-1.823206000	-6.499244000	1.916684000
1	-1.160489000	-5.953732000	3.302679000
8	-2.250393000	-2.681829000	7.298285000
1	-1.586199000	-3.044198000	7.977315000
1	-2.360212000	-1.692738000	7.260087000
8	-1.748772000	-5.976815000	5.015273000
1	-2.025373000	-6.900583000	5.156822000
1	-2.515307000	-5.303628000	5.296541000
8	0.325456000	-5.341695000	6.535667000
1	-0.444907000	-5.633010000	5.953575000
1	0.988379000	-4.884609000	5.988689000
8	-3.519292000	-4.346181000	5.760104000
1	-3.901721000	-3.719321000	5.048905000
1	-3.141710000	-3.759692000	6.500146000
1	3.666153000	1.237924000	6.742808000
1	3.264571000	4.708974000	3.352081000
1	-0.507312000	6.548567000	5.676225000
1	-1.883893000	7.384740000	1.124418000
1	-5.669148000	5.230120000	2.316305000
1	-4.770822000	6.310013000	-3.858748000
1	-1.853535000	7.514092000	-8.090997000
1	0.524024000	10.691472000	-5.159735000
1	6.866402000	4.787051000	-3.798245000
1	3.626853000	4.177049000	0.164781000
1	7.843542000	-1.242608000	-2.981762000
1	9.612775000	2.312166000	-0.192703000
1	6.123027000	0.988383000	3.081244000
1	8.973645000	-2.531718000	3.444397000
1	3.025541000	-0.648746000	-7.883775000
1	6.168012000	-2.950158000	-5.248998000
1	-6.991122000	-4.278463000	7.289293000
1	-7.176504000	-0.468986000	4.363078000
1	-4.575961000	-6.466482000	4.253615000
1	-9.308654000	-3.726323000	0.779920000
1	8.147195000	-4.598590000	-0.257645000
1	-7.004427000	4.233049000	-4.357264000
1	0.373960000	-0.443369000	-4.191041000
1	3.554297000	-6.488221000	-3.386540000
1	-0.583665000	-4.561588000	-0.920113000
1	4.505455000	-1.975752000	3.489670000

^3Pr_B:

6	8.624018000	-2.660688000	2.216897000
6	7.824532000	-1.518749000	1.605655000
8	7.720016000	-1.441041000	0.345531000
6	7.802759000	-3.987586000	2.331483000
6	6.853170000	-4.233596000	1.201109000
7	7.212403000	-4.411158000	-0.142686000
6	5.483012000	-4.236769000	1.208452000
6	6.104558000	-4.490984000	-0.920240000
7	5.049038000	-4.392978000	-0.101786000
1	9.486812000	-2.817732000	1.561802000
1	8.508668000	-4.819937000	2.445576000
1	7.206985000	-3.959864000	3.249613000
1	4.793025000	-4.127488000	2.025554000
1	6.056152000	-4.576286000	-2.008788000
1	4.019446000	-4.281812000	-0.368386000
7	7.248102000	-0.627088000	2.448002000
6	6.300029000	0.404759000	2.009178000
6	6.721269000	1.237393000	0.826546000
8	5.847276000	1.694498000	0.034562000
1	7.222157000	-0.788493000	3.455060000
1	5.324658000	-0.020997000	1.756909000
7	8.029604000	1.515227000	0.618591000
6	8.434201000	2.293192000	-0.552887000
6	7.867642000	1.754164000	-1.874066000
8	7.594706000	2.552604000	-2.804872000
1	8.721643000	1.175949000	1.269538000
1	8.091540000	3.328276000	-0.475656000
7	7.708392000	0.406247000	-1.987136000
6	7.187251000	-0.181817000	-3.217089000
6	5.666356000	-0.121051000	-3.411194000
8	5.177502000	-0.534391000	-4.494971000
1	7.845094000	-0.200490000	-1.178093000
1	7.625466000	0.329877000	-4.078381000
7	4.925363000	0.365218000	-2.388480000
6	3.468902000	0.566552000	-2.495487000
6	3.168189000	2.065781000	-2.333572000
8	2.688574000	2.753227000	-3.278981000
6	2.675294000	-0.288792000	-1.484833000
6	2.899918000	-1.800102000	-1.640492000
6	2.169854000	-2.606172000	-0.576767000
8	1.197625000	-2.048407000	0.037198000
8	2.544908000	-3.834288000	-0.339094000
1	5.389991000	0.694368000	-1.543442000
1	3.196858000	0.305612000	-3.519753000
1	1.608901000	-0.071472000	-1.612543000
1	2.922845000	0.010852000	-0.459966000
1	3.968125000	-2.044285000	-1.606215000
1	2.534628000	-2.157867000	-2.614918000
7	3.481517000	2.606971000	-1.130345000
6	3.238859000	4.006993000	-0.819158000
6	4.131556000	5.007949000	-1.535853000
8	3.803516000	6.231797000	-1.530349000
1	3.914668000	2.046546000	-0.399944000
1	2.210611000	4.291931000	-1.067618000
7	5.201555000	4.556171000	-2.224280000
6	5.935860000	5.435189000	-3.132661000
6	5.049021000	6.061369000	-4.215241000
8	5.410174000	7.118733000	-4.779675000
1	5.500843000	3.592019000	-2.145973000
1	6.388254000	6.273029000	-2.595439000
7	3.875720000	5.420200000	-4.506694000
6	2.906333000	5.991236000	-5.439417000
6	1.592077000	6.367330000	-4.733155000
8	0.518902000	6.472599000	-5.388677000
6	2.650708000	5.073633000	-6.644096000
1	3.646861000	4.521708000	-4.085892000
1	3.360659000	6.928082000	-5.791099000
1	1.876544000	5.498769000	-7.286296000
1	3.575383000	4.960041000	-7.216831000
1	2.317767000	4.083183000	-6.319004000
7	1.635548000	6.653699000	-3.399025000
6	0.393861000	6.867540000	-2.643763000
6	-0.437679000	8.048078000	-3.160755000
8	-1.682657000	8.057783000	-2.973105000
6	0.656310000	7.023885000	-1.132962000
8	0.485200000	5.714427000	-0.506001000
1	2.501607000	6.533567000	-2.868357000
1	-0.263610000	5.999962000	-2.766033000
1	1.667581000	7.403408000	-0.955840000
1	-0.076581000	7.727175000	-0.718405000
1	0.718018000	5.742934000	0.453663000
7	0.218743000	9.055962000	-3.787241000
6	-0.494267000	10.191816000	-4.366412000
6	-1.432521000	9.867239000	-5.539469000
8	-2.255244000	10.738662000	-5.902236000
1	1.215064000	8.961749000	-3.928721000
1	-1.123797000	10.665958000	-3.608361000
7	-1.313391000	8.640302000	-6.117926000
6	-2.241767000	8.189763000	-7.147513000
6	-3.096474000	6.990458000	-6.733394000
8	-3.667576000	6.298414000	-7.614227000
1	-0.601848000	7.976255000	-5.812452000
1	-2.898647000	9.031431000	-7.390946000
7	-3.203150000	6.747319000	-5.398460000
6	-3.962901000	5.617068000	-4.870235000
6	-3.074983000	4.541086000	-4.213791000
6	-2.078436000	3.824632000	-5.100914000
6	-2.312480000	3.586246000	-6.483508000
6	-0.883986000	3.316549000	-4.517369000
6	-1.427795000	2.831888000	-7.229952000
6	0.017270000	2.567983000	-5.244623000
6	-0.245011000	2.260667000	-6.631739000

8	0.537697000	1.483699000	-7.316561000	6	3.681586000	-1.960981000	4.058203000
1	-2.722885000	7.346662000	-4.733498000	6	2.489127000	-2.248919000	3.142792000
1	-4.548530000	5.208060000	-5.696703000	6	1.206916000	-2.531219000	3.864313000
1	-3.744312000	3.783482000	-3.771107000	7	0.014185000	-2.741653000	3.163820000
1	-2.538013000	4.996250000	-3.371104000	6	0.926708000	-2.610860000	5.213171000
1	-3.172175000	4.033479000	-6.969757000	6	-0.950895000	-2.928641000	4.079265000
1	-0.677519000	3.529721000	-3.472452000	7	-0.430027000	-2.863485000	5.323140000
1	-1.600253000	2.637718000	-8.282735000	1	2.684078000	-0.168483000	4.714423000
1	0.942333000	2.221939000	-4.795138000	1	3.778605000	-2.743074000	4.825267000
1	-3.704023000	-4.469639000	-2.475259000	1	2.738315000	-3.109314000	2.504800000
6	-4.849253000	4.422679000	2.760538000	1	2.355351000	-1.401014000	2.459431000
6	-3.406244000	4.810446000	2.566889000	1	1.551974000	-2.469767000	6.076873000
8	-2.467109000	3.974093000	2.688606000	1	-1.996400000	-3.081636000	3.876031000
6	-5.350664000	3.428874000	1.659591000	6	-0.045331000	-1.188099000	-5.337393000
6	-6.089427000	2.218499000	2.265470000	6	0.989306000	-1.899909000	-6.171821000
6	-5.092139000	1.285483000	2.965811000	8	1.192247000	-3.159255000	-6.053758000
8	-4.293953000	0.574938000	2.298715000	6	-1.486371000	-1.319410000	-5.918517000
8	-5.138137000	1.381333000	4.282955000	6	-2.165502000	-2.676472000	-5.672437000
1	-4.911491000	3.919637000	3.733690000	6	-2.532778000	-2.945186000	-4.217999000
1	-6.022109000	3.953994000	0.971829000	8	-2.146895000	-2.044265000	-3.345255000
1	-4.508673000	3.057401000	1.066284000	8	-3.168889000	-4.003922000	-3.918026000
1	-6.581746000	1.623638000	1.492089000	1	-0.041139000	-1.599351000	-4.326023000
1	-6.856217000	2.546616000	2.973206000	1	-2.091535000	-0.535105000	-5.453675000
7	-3.108899000	6.113942000	2.269575000	1	-1.458607000	-1.112058000	-6.995941000
6	-1.755701000	6.488323000	1.842143000	1	-1.528559000	-3.505463000	-6.009024000
6	-0.663191000	6.107483000	2.840036000	1	-3.088537000	-2.749975000	-6.261041000
8	0.460847000	5.710210000	2.423808000	7	1.675125000	-1.164573000	-7.071825000
1	-3.864301000	6.759555000	2.087443000	6	2.525297000	-1.768758000	-8.103977000
1	-1.488421000	6.004285000	0.896850000	6	3.643271000	-2.693976000	-7.616037000
7	-0.924773000	6.264712000	4.163488000	8	3.973370000	-3.678708000	-8.315231000
6	0.065410000	5.928830000	5.191719000	1	1.437947000	-0.170037000	-7.154180000
6	0.228615000	4.420320000	5.456569000	1	1.926775000	-2.378900000	-8.787135000
8	1.346844000	3.968664000	5.806354000	7	4.259418000	-2.378391000	-6.440006000
1	-1.834535000	6.610151000	4.436619000	6	5.309684000	-3.215378000	-5.879019000
1	1.046399000	6.301529000	4.892448000	6	4.855642000	-4.051075000	-4.682965000
7	-0.886785000	3.665896000	5.313106000	8	5.694848000	-4.408299000	-3.800365000
6	-0.912553000	2.189894000	5.437478000	1	4.072270000	-1.512824000	-5.946093000
6	0.016599000	1.586236000	4.360733000	1	5.651595000	-3.897258000	-6.666048000
8	0.958180000	0.807865000	4.661664000	7	3.559697000	-4.421002000	-4.658299000
6	-2.386217000	1.765512000	5.285924000	6	3.015936000	-5.327299000	-3.639583000
6	-2.746936000	0.375934000	5.784637000	6	1.543716000	-5.663364000	-3.907709000
8	-2.053579000	-0.214793000	6.665748000	8	0.737082000	-4.445853000	-3.808929000
8	-3.849416000	-0.141835000	5.294265000	6	1.004036000	-6.673875000	-2.899415000
1	-1.687203000	4.082718000	4.855024000	1	2.926235000	-4.035317000	-5.357608000
1	-0.514062000	1.885683000	6.406654000	1	3.083217000	-4.869154000	-2.646223000
1	-2.704220000	1.846205000	4.246589000	1	1.436362000	-6.059602000	-4.927087000
1	-3.011135000	2.463397000	5.860435000	1	0.757116000	-3.927596000	-4.668740000
7	-0.211748000	2.018464000	3.093887000	1	-0.059212000	-6.851966000	-3.081449000
6	0.607841000	1.576187000	1.961676000	1	1.536839000	-7.627517000	-2.982308000
6	2.058733000	2.060744000	2.109539000	1	1.120322000	-6.299657000	-1.876278000
8	3.023567000	1.364155000	1.689808000	6	-5.860258000	-6.301959000	3.910656000
6	-0.018931000	2.056918000	0.629007000	6	-6.948127000	-5.400637000	4.474227000
6	-1.440347000	1.515042000	0.322144000	8	-8.050583000	-5.245066000	3.876550000
6	-1.501743000	0.008324000	0.174880000	6	-6.450782000	-7.510697000	3.163991000
8	-1.073420000	-0.705335000	1.148165000	6	-5.363396000	-8.488237000	2.645209000
8	-1.983008000	-0.475444000	-0.935303000	6	-4.278092000	-7.754861000	1.881084000
1	-1.015037000	2.610179000	2.901030000	8	-3.173124000	-7.481946000	2.454853000
1	0.649829000	0.486197000	1.956523000	7	-4.561774000	-7.347801000	0.633756000
1	0.661222000	1.763586000	-0.177662000	1	-5.249094000	-5.701303000	3.222308000
1	-0.062584000	3.153920000	0.620112000	1	-7.070681000	-7.147745000	2.337011000
1	-2.139939000	1.790076000	1.119877000	1	-7.124999000	-8.063835000	3.828618000
1	-1.798189000	1.970294000	-0.604895000	1	-5.829759000	-9.252767000	2.011285000
7	2.248495000	3.272724000	2.699176000	1	-4.881642000	-8.997437000	3.484463000
6	3.572450000	3.803827000	3.006600000	1	-3.957917000	-6.683098000	0.120248000
6	4.405190000	2.919085000	3.934414000	1	-5.442571000	-7.594007000	0.207064000
8	5.640196000	2.790319000	3.719119000	7	-6.651749000	-4.777034000	5.645027000
1	1.449739000	3.860227000	2.902972000	6	-7.653503000	-3.991396000	6.361395000
1	4.184971000	3.920392000	2.108687000	6	-8.202183000	-2.767013000	5.624923000
7	3.775967000	2.306303000	4.965298000	8	-9.342211000	-2.331043000	5.909896000
6	4.504575000	1.356212000	5.806651000	1	-5.675378000	-4.770704000	5.957541000
6	4.702688000	-0.017547000	5.144690000	1	-8.525321000	-4.609871000	6.592648000
8	5.857371000	-0.532072000	5.047121000	7	-7.381805000	-2.199143000	4.699691000
1	2.802591000	2.515507000	5.170068000	6	-7.809529000	-1.062729000	3.902842000
1	5.500942000	1.743500000	6.024729000	6	-7.870676000	-1.349026000	2.403321000
7	3.589132000	-0.643492000	4.701208000	8	-7.787509000	-0.395842000	1.581196000

1	-6.413661000	-2.508323000	4.573291000
1	-8.811828000	-0.781743000	4.247243000
7	-8.066366000	-2.643456000	2.041092000
6	-8.260796000	-3.055890000	0.651554000
6	-7.180880000	-3.998914000	0.128396000
8	-5.933352000	-3.282150000	-0.066256000
1	-8.145896000	-3.354961000	2.766305000
1	-8.282582000	-2.152783000	0.037899000
1	-7.035291000	-4.831851000	0.831522000
1	-7.510512000	-4.411424000	-0.833885000
1	-5.319229000	-3.752750000	-0.698701000
6	-7.398513000	2.178640000	-5.013540000
6	-6.716878000	1.589977000	-3.796019000
6	-7.451504000	1.291225000	-2.634615000
6	-5.341805000	1.294460000	-3.809590000
6	-6.836545000	0.708732000	-1.519575000
6	-4.715014000	0.718355000	-2.696014000
6	-5.463751000	0.426978000	-1.548740000
1	-7.864898000	1.395552000	-5.627303000
1	-8.191094000	2.881010000	-4.731028000
1	-8.516686000	1.509248000	-2.608928000
1	-4.762018000	1.504272000	-4.705979000
1	-7.417258000	0.457533000	-0.636353000
1	-3.656640000	0.479432000	-2.717815000
1	-4.981986000	-0.021722000	-0.687390000
26	-0.453896000	-2.732097000	1.141906000
26	-1.951845000	-2.387029000	-1.459842000
8	0.174123000	-4.732600000	0.602456000
1	1.147374000	-4.687183000	0.416785000
1	-0.164653000	-5.404720000	1.319492000
8	-4.574749000	-2.440954000	4.070469000
1	-4.319876000	-1.582327000	4.500712000
1	-4.495053000	-2.372042000	3.064106000
8	-4.368615000	-2.001820000	1.502780000
1	-5.117582000	-2.387665000	0.931689000
1	-4.361793000	-1.013467000	1.579336000
8	-2.275635000	-3.126963000	0.331914000
1	-2.797720000	-4.515074000	-0.172289000
1	-3.018312000	-2.649083000	0.829099000
8	-1.368855000	-4.669521000	8.793346000
1	-1.143156000	-5.387747000	8.102941000
1	-1.743419000	-5.049719000	9.603146000
8	-4.190381000	-4.632202000	-1.582768000
8	-3.188763000	-5.322978000	-0.687570000
8	-0.747546000	-3.898884000	-1.751407000
1	-0.220356000	-4.118328000	-2.582969000
8	-0.828974000	-6.207859000	2.357042000
1	-1.612346000	-6.756359000	2.098165000
1	-1.014147000	-5.892579000	3.273306000
8	-2.260479000	-2.885396000	7.261741000
1	-1.939050000	-3.430174000	8.062622000
1	-2.328184000	-1.897093000	7.313207000
8	-2.219318000	-6.133944000	4.773716000
1	-2.659174000	-6.819877000	4.221426000
1	-2.881986000	-5.402914000	5.074863000
8	-0.847878000	-6.395607000	6.960742000
1	-1.383796000	-6.392137000	6.096420000
1	0.065938000	-6.682230000	6.805256000
8	-3.792927000	-4.305871000	5.666658000
1	-4.114334000	-3.634240000	4.973071000
1	-3.294847000	-3.793459000	6.383102000
1	3.949106000	1.234469000	6.741103000
1	3.433031000	4.788867000	3.461236000
1	-0.221703000	6.426254000	6.123088000
1	-1.739219000	7.572389000	1.687011000
1	-5.494611000	5.306801000	2.807216000
1	-4.670282000	5.980983000	-4.115497000
1	-1.711291000	7.899928000	-8.059198000
1	0.241163000	10.928358000	-4.701579000
1	6.733241000	4.841977000	-3.587211000
1	3.354189000	4.141271000	0.257222000
1	7.482741000	-1.234312000	-3.259839000
1	9.527387000	2.295477000	-0.603420000
1	6.148924000	1.094851000	2.847733000
1	9.004542000	-2.401066000	3.210058000

1	2.966891000	-0.949695000	-8.680709000
1	6.141689000	-2.593953000	-5.550153000
1	-7.210186000	-3.664434000	7.307647000
1	-7.146395000	-0.208215000	4.057014000
1	-5.192020000	-6.629773000	4.713752000
1	-9.231333000	-3.562186000	0.563124000
1	8.151886000	-4.368413000	-0.505332000
1	-6.688154000	2.711645000	-5.655144000
1	0.207410000	-0.128550000	-5.267783000
1	3.612737000	-6.248154000	-3.627084000
1	-0.503787000	-4.441096000	-0.951372000
1	4.608784000	-1.977623000	3.476122000

$^{1}Re_{B}$:

6	8.738108000	-3.026746000	3.192952000
6	8.037422000	-1.891276000	2.460810000
8	8.054621000	-1.860310000	1.195071000
6	7.819138000	-4.275652000	3.388542000
6	6.941767000	-4.594994000	2.218240000
7	7.389269000	-4.917199000	0.929148000
6	5.574914000	-4.563256000	2.123633000
6	6.337950000	-5.053607000	0.084177000
7	5.228899000	-4.844209000	0.806853000
1	9.610031000	-3.296088000	2.589016000
1	8.454001000	-5.131416000	3.651738000
1	7.160226000	-4.103221000	4.245833000
1	4.833513000	-4.353065000	2.872563000
1	6.380888000	-5.257851000	-0.990524000
1	4.226273000	-4.731091000	0.449989000
7	7.399481000	-0.960105000	3.211977000
6	6.495147000	0.051532000	2.653280000
6	7.007327000	0.827734000	1.469883000
8	6.204390000	1.208179000	0.570968000
1	7.285151000	-1.080006000	4.217644000
1	5.540063000	-0.383119000	2.345313000
7	8.318314000	1.147635000	1.365819000
6	8.787446000	1.900595000	0.199655000
6	8.366564000	1.276248000	-1.138318000
8	8.128869000	2.010997000	-2.128146000
1	8.961012000	0.880743000	2.096418000
1	8.386425000	2.917319000	0.200991000
7	8.304397000	-0.084511000	-1.195358000
6	7.936915000	-0.759570000	-2.434159000
6	6.447380000	-0.737004000	-2.796927000
8	6.094423000	-1.161195000	-3.929377000
1	8.388374000	-0.643206000	-0.346439000
1	8.460739000	-0.297142000	-3.275159000
7	5.587944000	-0.271080000	-1.863386000
6	4.154212000	-0.074298000	-2.133696000
6	3.827985000	1.429047000	-2.165794000
8	3.274043000	1.951571000	-3.172404000
6	3.250026000	-0.805161000	-1.120827000
6	3.428643000	-2.330075000	-1.118578000
6	2.498721000	-3.011911000	-0.131450000
8	1.463871000	-2.366880000	0.254761000
8	2.766079000	-4.222553000	0.280591000
1	5.956941000	0.075457000	-0.979979000
1	3.978324000	-0.447642000	-3.143398000
1	2.208048000	-0.572553000	-1.365710000
1	3.419522000	-0.409603000	-0.112259000
1	4.465905000	-2.606416000	-0.895768000
1	3.192932000	-2.762114000	-2.102374000
7	4.174992000	2.133678000	-1.059816000
6	3.875371000	3.548013000	-0.883140000
6	4.582544000	4.499733000	-1.835405000
8	4.137294000	5.678315000	-1.957331000
1	4.640142000	1.672482000	-0.280338000
1	2.804410000	3.750047000	-1.009293000
7	5.605155000	4.045114000	-2.593980000
6	6.083664000	4.841551000	-3.723548000
6	4.980570000	5.188909000	-4.738045000
8	5.140198000	6.163766000	-5.507834000
1	6.052510000	3.156779000	-2.395664000
1	6.496467000	5.797765000	-3.391775000
7	3.865487000	4.399040000	-4.743584000

6	2.719468000	4.690809000	-5.602479000
6	1.529669000	5.296321000	-4.833453000
8	0.409112000	5.407211000	-5.401772000
6	2.265219000	3.441032000	-6.376032000
1	3.795374000	3.571536000	-4.149729000
1	3.055541000	5.459873000	-6.309389000
1	1.362888000	3.665615000	-6.948325000
1	3.059930000	3.121585000	-7.057114000
1	2.042319000	2.623408000	-5.681706000
7	1.735932000	5.752418000	-3.566141000
6	0.609074000	6.187030000	-2.733296000
6	-0.099150000	7.442038000	-3.264245000
8	-1.263245000	7.712541000	-2.865578000
6	1.032001000	6.414458000	-1.274198000
8	0.948633000	5.140299000	-0.564189000
1	2.652990000	5.672978000	-3.124578000
1	-0.165536000	5.411209000	-2.739917000
1	2.051905000	6.809867000	-1.226979000
1	0.337217000	7.136196000	-0.828236000
1	1.110326000	5.263776000	0.402168000
7	0.575457000	8.212978000	-4.151489000
6	-0.053567000	9.349533000	-4.815245000
6	-1.165141000	8.999340000	-5.816514000
8	-1.867245000	9.929786000	-6.274403000
1	1.502940000	7.921560000	-4.427717000
1	-0.515303000	10.005690000	-4.072232000
7	-1.329834000	7.686485000	-6.139764000
6	-2.438268000	7.250539000	-6.979629000
6	-3.637286000	6.664395000	-6.226005000
8	-4.584265000	6.163506000	-6.883965000
1	-0.692561000	6.967830000	-5.796597000
1	-2.795614000	8.111641000	-7.551659000
7	-3.610467000	6.730890000	-4.869049000
6	-4.711323000	6.243593000	-4.037575000
6	-4.303181000	5.135876000	-3.050654000
6	-3.908118000	3.783541000	-3.631027000
6	-4.185274000	3.391251000	-4.953244000
6	-3.289205000	2.842627000	-2.788652000
6	-3.861841000	2.107502000	-5.405204000
6	-2.964168000	1.555341000	-3.223814000
6	-3.254086000	1.181226000	-4.546652000
8	-2.956559000	-0.069379000	-5.047443000
1	-2.813804000	7.148475000	-4.399643000
1	-5.495603000	5.903666000	-4.718005000
1	-5.158234000	4.991924000	-2.368853000
1	-3.478692000	5.518300000	-2.429036000
1	-4.637632000	4.087153000	-5.652755000
1	-3.067239000	3.120826000	-1.759308000
1	-4.072593000	1.808931000	-6.426045000
1	-2.495531000	0.839462000	-2.555898000
1	-2.541713000	-0.705491000	-4.391633000
6	-4.709283000	4.369698000	2.374009000
6	-3.238401000	4.665006000	2.231741000
8	-2.354702000	3.796732000	2.485445000
6	-5.208674000	3.308548000	1.337271000
6	-6.003463000	2.166902000	2.001016000
6	-5.065405000	1.227814000	2.763812000
8	-4.225451000	0.514458000	2.152073000
8	-5.195717000	1.313578000	4.075443000
1	-4.849924000	3.964337000	3.383982000
1	-5.847350000	3.795356000	0.593239000
1	-4.362344000	2.872070000	0.796662000
1	-6.504198000	1.560089000	1.243475000
1	-6.778321000	2.551398000	2.668074000
7	-2.853190000	5.919532000	1.843273000
6	-1.456588000	6.187571000	1.481919000
6	-0.457340000	5.866865000	2.591224000
8	0.686908000	5.416074000	2.299569000
1	-3.558323000	6.580021000	1.547281000
1	-1.150046000	5.593680000	0.614680000
7	-0.816034000	6.121110000	3.874789000
6	0.080411000	5.820014000	4.995529000
6	0.192247000	4.321795000	5.334876000
8	1.264075000	3.869427000	5.807987000
1	-1.733813000	6.505259000	4.052543000

1	1.089321000	6.165412000	4.763952000
7	-0.918667000	3.578365000	5.117191000
6	-0.973094000	2.110735000	5.299139000
6	0.024858000	1.455791000	4.319073000
8	0.921677000	0.670337000	4.724749000
6	-2.437285000	1.685929000	5.068557000
6	-2.847452000	0.336204000	5.632203000
8	-2.207857000	-0.208346000	6.583501000
8	-3.924494000	-0.204543000	5.113461000
1	-1.659754000	3.977627000	4.553747000
1	-0.643822000	1.845800000	6.305143000
1	-2.678691000	1.696782000	4.005305000
1	-3.094283000	2.425433000	5.546275000
7	-0.084187000	1.843375000	3.022954000
6	0.825355000	1.338549000	1.987934000
6	2.270845000	1.781028000	2.260759000
8	3.242479000	1.017248000	2.011645000
6	0.367199000	1.797771000	0.583527000
6	-1.035714000	1.315197000	0.134752000
6	-1.159152000	-0.190145000	0.033354000
8	-0.955911000	-0.869020000	1.115487000
8	-1.454347000	-0.732320000	-1.095225000
1	-0.858110000	2.437428000	2.738985000
1	0.829354000	0.248708000	2.023511000
1	1.114112000	1.441808000	-0.134862000
1	0.380296000	2.892600000	0.525152000
1	-1.805097000	1.659194000	0.836868000
1	-1.249125000	1.754652000	-0.841575000
7	2.445730000	3.033129000	2.766214000
6	3.758334000	3.547334000	3.138176000
6	4.480137000	2.712846000	4.196785000
8	5.725703000	2.542263000	4.105046000
1	1.656484000	3.664700000	2.840684000
1	4.441439000	3.571723000	2.284436000
7	3.744412000	2.184387000	5.204474000
6	4.363117000	1.260980000	6.156772000
6	4.576378000	-0.150998000	5.583681000
8	5.711355000	-0.713323000	5.639931000
1	2.765934000	2.438289000	5.315759000
1	5.347478000	1.630633000	6.448117000
7	3.494425000	-0.753433000	5.040946000
6	3.607275000	-2.086069000	4.436007000
6	2.498153000	-2.358746000	3.414803000
6	1.147018000	-2.579762000	4.021506000
7	0.003212000	-2.760126000	3.234549000
6	0.759316000	-2.604100000	5.346416000
6	-1.037809000	-2.868597000	4.074939000
7	-0.614068000	-2.786342000	5.354118000
1	2.613293000	-0.244795000	4.934554000
1	3.607896000	-2.854560000	5.223640000
1	2.773633000	-3.239816000	2.818974000
1	2.458135000	-1.520643000	2.708343000
1	1.314853000	-2.430595000	6.251278000
1	-2.071631000	-2.970265000	3.794216000
6	1.541230000	-1.186457000	-4.960358000
6	2.413046000	-2.197874000	-5.669559000
8	2.341287000	-3.443191000	-5.440505000
6	0.223219000	-0.873783000	-5.726347000
6	-0.709549000	-2.084780000	-5.938639000
6	-1.101414000	-2.781046000	-4.637193000
8	-1.646457000	-2.003924000	-3.700784000
8	-0.882965000	-4.008129000	-4.477052000
1	1.289131000	-1.589736000	-3.974593000
1	-0.316361000	-0.111942000	-5.157390000
1	0.464120000	-0.431895000	-6.702744000
1	-0.252096000	-2.836908000	-6.587665000
1	-1.621768000	-1.715553000	-6.421921000
7	3.279364000	-1.729834000	-6.612194000
6	3.994366000	-2.621392000	-7.530673000
6	4.983764000	-3.599479000	-6.890033000
8	5.274075000	-4.657506000	-7.492998000
1	3.329594000	-0.733202000	-6.767458000
1	3.285563000	-3.244783000	-8.083513000
7	5.509604000	-3.251882000	-5.683271000
6	6.392001000	-4.140402000	-4.946475000

6	5.709719000	-4.868044000	-3.787664000
8	6.404900000	-5.285173000	-2.810157000
1	5.349139000	-2.339741000	-5.265596000
1	6.774998000	-4.895557000	-5.642625000
7	4.385165000	-5.084222000	-3.893224000
6	3.641040000	-5.905849000	-2.931319000
6	2.150348000	-5.964863000	-3.279232000
8	1.580135000	-4.624419000	-3.132915000
6	1.392383000	-6.914531000	-2.355343000
1	3.866321000	-4.636072000	-4.646845000
1	3.735106000	-5.487586000	-1.923243000
1	2.024669000	-6.284248000	-4.322429000
1	1.532943000	-4.169921000	-4.015037000
1	0.331850000	-6.919719000	-2.621096000
1	1.778975000	-7.936386000	-2.440125000
1	1.487724000	-6.589685000	-1.313331000
6	-5.396513000	-5.983530000	2.970168000
6	-6.603354000	-5.471173000	3.747417000
8	-7.719239000	-5.308949000	3.176505000
6	-5.809154000	-7.050514000	1.938304000
6	-4.689662000	-7.437746000	0.932630000
6	-4.163934000	-6.234753000	0.179287000
8	-3.175232000	-5.579298000	0.669453000
7	-4.779515000	-5.837183000	-0.936295000
1	-4.952830000	-5.120585000	2.456128000
1	-6.688984000	-6.682260000	1.400376000
1	-6.125179000	-7.963412000	2.458390000
1	-5.080677000	-8.185829000	0.233080000
1	-3.843672000	-7.887785000	1.461965000
1	-4.482777000	-4.968382000	-1.426044000
1	-5.542119000	-6.373296000	-1.322814000
7	-6.407001000	-5.169470000	5.057502000
6	-7.504942000	-4.690660000	5.895246000
6	-8.068439000	-3.309820000	5.542242000
8	-9.205096000	-2.987028000	5.957089000
1	-5.456481000	-5.133886000	5.434647000
1	-8.351180000	-5.379903000	5.833209000
7	-7.276054000	-2.500075000	4.786410000
6	-7.744602000	-1.212965000	4.295126000
6	-8.018200000	-1.187781000	2.790358000
8	-8.091159000	-0.080644000	2.193725000
1	-6.320918000	-2.764719000	4.544909000
1	-8.677070000	-0.976932000	4.819242000
7	-8.202525000	-2.387806000	2.181735000
6	-8.523454000	-2.514325000	0.762605000
6	-7.474952000	-3.305564000	-0.015236000
8	-6.214086000	-2.581776000	-0.105264000
1	-8.152383000	-3.242976000	2.730753000
1	-8.618875000	-1.508298000	0.350220000
1	-7.297018000	-4.269213000	0.477220000
1	-7.847823000	-3.493472000	-1.029858000
1	-5.834747000	-2.528322000	-1.034307000
6	-9.510947000	4.212316000	-3.277026000
6	-8.769813000	3.081584000	-2.595029000
6	-9.221142000	2.556695000	-1.371286000
6	-7.625086000	2.514735000	-3.183793000
6	-8.557744000	1.487869000	-0.755486000
6	-6.956950000	1.445543000	-2.575780000
6	-7.423827000	0.926997000	-1.360878000
1	-10.259632000	3.829082000	-3.984296000
1	-10.041769000	4.840119000	-2.552373000
1	-10.100004000	2.988737000	-0.898163000
1	-7.249021000	2.912155000	-4.122258000
1	-8.902371000	1.097920000	0.197450000
1	-6.071828000	1.029093000	-3.045347000
1	-6.911598000	0.093934000	-0.887764000
26	-0.344840000	-2.820008000	1.168034000
26	-1.975050000	-2.664501000	-1.773649000
8	0.204767000	-4.871219000	0.926992000
1	1.195034000	-4.895738000	0.836110000
1	-0.245727000	-5.499838000	1.615188000
8	-4.430494000	-2.558699000	3.944826000
1	-4.273004000	-1.678431000	4.391641000
1	-4.355525000	-2.455528000	2.946875000
8	-4.190294000	-2.000503000	1.350632000
1	-5.061381000	-2.224504000	0.857314000
1	-4.118863000	-1.018324000	1.531597000
8	-2.254938000	-3.163563000	0.167267000
1	-2.552357000	-4.129369000	0.243748000
1	-2.997365000	-2.597043000	0.651150000
8	-1.002341000	-4.162455000	8.799343000
1	-0.607530000	-4.796123000	8.113811000
1	-1.008166000	-4.519199000	9.699854000
8	-4.695625000	-2.379156000	-2.233166000
8	-3.863355000	-3.485642000	-2.090189000
8	-0.521128000	-4.136513000	-1.572214000
1	0.133615000	-4.340304000	-2.300996000
8	-1.271479000	-6.199014000	2.457001000
1	-2.092629000	-6.102606000	1.911300000
1	-1.448967000	-6.142770000	3.432992000
8	-2.508365000	-2.796024000	7.304982000
1	-2.004816000	-3.201554000	8.089908000
1	-2.571515000	-1.801649000	7.253676000
8	-2.026530000	-6.162453000	5.087046000
1	-2.373484000	-7.054529000	5.271754000
1	-2.751724000	-5.413023000	5.293930000
8	-0.103915000	-5.589595000	6.837772000
1	-0.796345000	-5.847305000	6.154728000
1	0.674968000	-5.210069000	6.396270000
8	-3.684548000	-4.368085000	5.627330000
1	-3.971078000	-3.731040000	4.885835000
1	-3.351735000	-3.798499000	6.407542000
1	3.720670000	1.203724000	7.040289000
1	3.621267000	4.570022000	3.502133000
1	-0.274918000	6.361509000	5.877331000
1	-1.373630000	7.245965000	1.213871000
1	-5.304092000	5.287119000	2.306152000
1	-5.119059000	7.086356000	-3.462446000
1	-2.095819000	6.487781000	-7.684266000
1	0.717629000	9.921996000	-5.337295000
1	6.877934000	4.278552000	-4.221138000
1	4.134989000	3.818494000	0.143661000
1	8.252376000	-1.805223000	-2.370967000
1	9.879269000	1.958502000	0.244309000
1	6.282843000	0.781099000	3.443376000
1	9.100862000	-2.707395000	4.175633000
1	4.531873000	-1.999292000	-8.252966000
1	7.231527000	-3.577345000	-4.536590000
1	-7.157226000	-4.673299000	6.932678000
1	-7.019226000	-0.424345000	4.512429000
1	-4.627409000	-6.367435000	3.650460000
1	-9.490117000	-3.025340000	0.652854000
1	8.352772000	-4.945818000	0.633825000
1	-8.829373000	4.852974000	-3.847495000
1	2.090488000	-0.248992000	-4.802670000
1	4.068248000	-6.916892000	-2.918111000
1	-0.316950000	-4.633634000	-0.740927000
1	4.584191000	-2.143551000	3.944933000

^{1}TS1$_{B}$:

6	8.803003000	-1.316205000	3.245898000
6	8.099589000	-0.168880000	2.535503000
8	8.119424000	-0.114046000	1.270112000
6	7.898111000	-2.581177000	3.398998000
6	7.029598000	-2.880357000	2.216802000
7	7.485022000	-3.157664000	0.919870000
6	5.662245000	-2.874430000	2.121179000
6	6.436201000	-3.291856000	0.070515000
7	5.323236000	-3.126325000	0.797311000
1	9.684720000	-1.559850000	2.645023000
1	8.542668000	-3.435868000	3.641554000
1	7.232790000	-2.442412000	4.257420000
1	4.915042000	-2.700787000	2.874199000
1	6.477816000	-3.463125000	-1.007995000
1	4.313988000	-3.018810000	0.443488000
7	7.461303000	0.748531000	3.302354000
6	6.564380000	1.776540000	2.761115000
6	7.080656000	2.558967000	1.583693000
8	6.283911000	2.938182000	0.678965000
1	7.342092000	0.610097000	4.304973000

1	5.602779000	1.356053000	2.454184000
7	8.390630000	2.886731000	1.488599000
6	8.859444000	3.649855000	0.328852000
6	8.435878000	3.037655000	-1.013718000
8	8.186142000	3.781652000	-1.994432000
1	9.030880000	2.624319000	2.222972000
1	8.459455000	4.666993000	0.339437000
7	8.385987000	1.677925000	-1.088603000
6	8.018832000	1.019274000	-2.336347000
6	6.531733000	1.061491000	-2.705871000
8	6.186102000	0.695269000	-3.860876000
1	8.471135000	1.108804000	-0.246478000
1	8.551173000	1.483384000	-3.170748000
7	5.665075000	1.472815000	-1.753729000
6	4.231482000	1.673702000	-2.021778000
6	3.909024000	3.177587000	-2.054230000
8	3.340373000	3.695768000	-3.056568000
6	3.331837000	0.934519000	-1.010568000
6	3.512686000	-0.589326000	-1.036974000
6	2.597014000	-1.301621000	-0.055282000
8	1.577266000	-0.669166000	0.380073000
8	2.868355000	-2.532365000	0.291069000
1	6.028171000	1.787671000	-0.855921000
1	4.053822000	1.304140000	-3.032756000
1	2.288049000	1.171197000	-1.244000000
1	3.509357000	1.316420000	0.001821000
1	4.553917000	-0.865371000	-0.832649000
1	3.269453000	-1.000036000	-2.028194000
7	4.277692000	3.887101000	-0.960270000
6	4.002802000	5.308000000	-0.795972000
6	4.714795000	6.234562000	-1.769571000
8	4.274520000	7.412146000	-1.919261000
1	4.744802000	3.426313000	-0.181228000
1	2.934257000	5.526414000	-0.914131000
7	5.736005000	5.759219000	-2.517014000
6	6.223678000	6.532732000	-3.658423000
6	5.128165000	6.866661000	-4.684901000
8	5.297724000	7.823546000	-5.474803000
1	6.191993000	4.880655000	-2.292708000
1	6.639896000	7.493044000	-3.342767000
7	4.006343000	6.085684000	-4.679416000
6	2.865509000	6.372590000	-5.546035000
6	1.675481000	6.991514000	-4.787771000
8	0.553785000	7.091893000	-5.356795000
6	2.409733000	5.117536000	-6.309813000
1	3.923200000	5.274770000	-4.064942000
1	3.208611000	7.132741000	-6.259364000
1	1.510878000	5.340019000	-6.888439000
1	3.206650000	4.788901000	-6.984125000
1	2.180693000	4.308883000	-5.606607000
7	1.884843000	7.473237000	-3.530999000
6	0.761046000	7.933011000	-2.708189000
6	0.071665000	9.189521000	-3.259610000
8	-1.088074000	9.484265000	-2.864506000
6	1.179039000	8.170703000	-1.249432000
8	1.080578000	6.903479000	-0.529730000
1	2.801141000	7.397222000	-3.086361000
1	-0.023717000	7.167413000	-2.709794000
1	2.202070000	8.557580000	-1.200621000
1	0.488237000	8.902659000	-0.814192000
1	1.232217000	7.031910000	0.438233000
7	0.754378000	9.930895000	-4.165119000
6	0.140181000	11.060546000	-4.854331000
6	-0.987417000	10.701666000	-5.834283000
8	-1.683937000	11.629364000	-6.305329000
1	1.679165000	9.623143000	-4.432508000
1	-0.302492000	11.746363000	-4.126648000
7	-1.173323000	9.383479000	-6.124745000
6	-2.303012000	8.943870000	-6.933789000
6	-3.506953000	8.420320000	-6.142424000
8	-4.478071000	7.923520000	-6.767652000
1	-0.540238000	8.664855000	-5.774398000
1	-2.646771000	9.790308000	-7.535265000
7	-3.456761000	8.535241000	-4.789381000
6	-4.560951000	8.121687000	-3.923905000
6	-4.185570000	7.016400000	-2.920810000
6	-3.877159000	5.632892000	-3.479990000
6	-4.223742000	5.225550000	-4.781455000
6	-3.271727000	4.682581000	-2.638365000
6	-3.984519000	3.916928000	-5.213500000
6	-3.031647000	3.370074000	-3.054254000
6	-3.393936000	2.982288000	-4.353948000
8	-3.189286000	1.700501000	-4.833692000
1	-2.641582000	8.946848000	-4.346644000
1	-5.377775000	7.806655000	-4.577688000
1	-5.029483000	6.930850000	-2.216075000
1	-3.326760000	7.368323000	-2.328879000
1	-4.662789000	5.928776000	-5.481870000
1	-2.988503000	4.972252000	-1.627887000
1	-4.248358000	3.606815000	-6.218264000
1	-2.566877000	2.650556000	-2.387476000
1	-2.739410000	1.081349000	-4.195162000
6	-4.628950000	6.238548000	2.316719000
6	-3.147216000	6.490977000	2.193901000
8	-2.286869000	5.606077000	2.466288000
6	-5.148286000	5.160620000	1.306343000
6	-5.908886000	4.019357000	2.005881000
6	-4.938938000	3.084792000	2.734902000
8	-4.039804000	2.467758000	2.100153000
8	-5.132707000	3.052895000	4.039617000
1	-4.800489000	5.874044000	3.337645000
1	-5.813578000	5.633799000	0.577198000
1	-4.314897000	4.723929000	0.746774000
1	-6.439896000	3.399422000	1.277756000
1	-6.653269000	4.409975000	2.705542000
7	-2.730736000	7.736662000	1.804872000
6	-1.324856000	7.977000000	1.464760000
6	-0.348882000	7.650869000	2.592702000
8	0.796933000	7.190051000	2.322040000
1	-3.418347000	8.408697000	1.494306000
1	-1.014339000	7.370501000	0.607877000
7	-0.729204000	7.911508000	3.868061000
6	0.143688000	7.609237000	5.006816000
6	0.247166000	6.111202000	5.349136000
8	1.311161000	5.657907000	5.838365000
1	-1.648911000	8.299311000	4.026843000
1	1.157666000	7.953128000	4.795942000
7	-0.861585000	5.368558000	5.115402000
6	-0.917279000	3.901651000	5.303324000
6	0.102475000	3.245385000	4.347986000
8	0.995713000	2.466457000	4.772315000
6	-2.373262000	3.467614000	5.040966000
6	-2.786294000	2.123260000	5.615974000
8	-2.172707000	1.603114000	6.597184000
8	-3.838120000	1.559813000	5.070991000
1	-1.595037000	5.765799000	4.540447000
1	-0.610141000	3.641802000	6.317676000
1	-2.587960000	3.457342000	3.971661000
1	-3.046252000	4.209893000	5.491161000
7	0.016035000	3.626809000	3.047297000
6	0.945225000	3.116042000	2.033471000
6	2.385516000	3.558871000	2.326130000
8	3.359907000	2.797705000	2.080785000
6	0.510505000	3.563419000	0.618653000
6	-0.868574000	3.043347000	0.140849000
6	-0.955554000	1.532842000	0.071667000
8	-0.744240000	0.869086000	1.140803000
8	-1.242006000	0.987733000	-1.079628000
1	-0.755308000	4.215890000	2.746768000
1	0.949945000	2.026166000	2.077149000
1	1.282372000	3.226235000	-0.081892000
1	0.496723000	4.658180000	0.558576000
1	-1.666448000	3.381132000	0.814409000
1	-1.065906000	3.458919000	-0.849267000
7	2.555820000	4.806356000	2.843973000
6	3.865483000	5.309602000	3.239773000
6	4.570263000	4.451077000	4.290957000
8	5.817147000	4.281380000	4.216638000
1	1.766809000	5.438157000	2.916219000
1	4.558340000	5.348306000	2.394382000

7	3.815209000	3.904918000	5.274354000
6	4.411667000	2.968452000	6.227841000
6	4.616163000	1.554972000	5.654946000
8	5.749839000	0.989371000	5.710159000
1	2.831389000	4.145570000	5.359983000
1	5.396700000	3.326716000	6.530555000
7	3.530195000	0.954250000	5.118502000
6	3.632041000	-0.388765000	4.532653000
6	2.535638000	-0.663609000	3.498702000
6	1.171209000	-0.862165000	4.084083000
7	0.045987000	-1.074092000	3.278923000
6	0.755838000	-0.839050000	5.401167000
6	-1.011897000	-1.156950000	4.100305000
7	-0.616675000	-1.026209000	5.386219000
1	2.652271000	1.467355000	5.010112000
1	3.609451000	-1.145347000	5.331098000
1	2.812390000	-1.558026000	2.923281000
1	2.515122000	0.161236000	2.776192000
1	1.291029000	-0.632786000	6.311562000
1	-2.038596000	-1.277636000	3.800572000
6	1.424193000	0.653571000	-4.761537000
6	2.304240000	-0.283997000	-5.558499000
8	2.227010000	-1.546179000	-5.448768000
6	0.084965000	0.998611000	-5.476633000
6	-0.840259000	-0.201174000	-5.775603000
6	-1.245728000	-0.995063000	-4.537174000
8	-1.796326000	-0.254249000	-3.548503000
8	-1.055508000	-2.222276000	-4.450005000
1	1.204709000	0.172505000	-3.802968000
1	-0.451384000	1.708529000	-4.840903000
1	0.300367000	1.517583000	-6.419995000
1	-0.374811000	-0.907013000	-6.468867000
1	-1.750898000	0.195864000	-6.239749000
7	3.181416000	0.266194000	-6.441782000
6	3.916290000	-0.543888000	-7.420018000
6	4.886630000	-1.582464000	-6.846316000
8	5.144404000	-2.610552000	-7.511982000
1	3.239823000	1.272812000	-6.502606000
1	3.218650000	-1.111023000	-8.042629000
7	5.437224000	-1.314856000	-5.629998000
6	6.334490000	-2.249036000	-4.967762000
6	5.680889000	-3.018896000	-3.818544000
8	6.386355000	-3.439019000	-2.851760000
1	5.319727000	-0.417195000	-5.167824000
1	6.682059000	-2.974158000	-5.712739000
7	4.360475000	-3.262244000	-3.928577000
6	3.621790000	-4.103986000	-2.979963000
6	2.129676000	-4.141708000	-3.323148000
8	1.586178000	-2.787956000	-3.176760000
6	1.353830000	-5.081005000	-2.404681000
1	3.841374000	-2.814044000	-4.679624000
1	3.727168000	-3.709442000	-1.963730000
1	1.995992000	-4.452155000	-4.368241000
1	1.559724000	-2.320462000	-4.055584000
1	0.292947000	-5.064217000	-2.668725000
1	1.720554000	-6.109153000	-2.500132000
1	1.456014000	-4.770454000	-1.359458000
6	-5.455479000	-4.609782000	3.316844000
6	-6.624719000	-3.837148000	3.910950000
8	-7.682717000	-3.651467000	3.244689000
6	-5.962329000	-5.838696000	2.534783000
6	-4.857109000	-6.595782000	1.750991000
6	-4.130614000	-5.673232000	0.792564000
8	-3.115679000	-5.016919000	1.163463000
7	-4.651601000	-5.497537000	-0.440643000
1	-4.909885000	-3.937460000	2.642025000
1	-6.749646000	-5.504137000	1.850720000
1	-6.434465000	-6.545330000	3.229076000
1	-5.310143000	-7.437845000	1.213739000
1	-4.113049000	-7.003376000	2.442897000
1	-4.236314000	-4.775046000	-1.029534000
1	-5.460158000	-6.007086000	-0.759024000
7	-6.469814000	-3.362054000	5.175018000
6	-7.576928000	-2.717124000	5.880592000
6	-8.108052000	-1.420477000	5.263097000
8	-9.281667000	-1.056606000	5.507327000
1	-5.537464000	-3.346671000	5.594977000
1	-8.435621000	-3.392226000	5.930342000
7	-7.238540000	-0.717631000	4.488084000
6	-7.640457000	0.494722000	3.795090000
6	-7.682105000	0.357004000	2.273975000
8	-7.642414000	1.392558000	1.556697000
1	-6.258995000	-0.995621000	4.392627000
1	-8.645669000	0.757247000	4.144406000
7	-7.807190000	-0.899592000	1.770181000
6	-7.997481000	-1.152591000	0.342696000
6	-6.814641000	-1.829658000	-0.343188000
8	-5.695420000	-0.893867000	-0.441523000
1	-7.859578000	-1.692648000	2.407770000
1	-8.195321000	-0.193514000	-0.141489000
1	-6.503756000	-2.725123000	0.210796000
1	-7.117074000	-2.129171000	-1.354213000
1	-5.045937000	-1.173902000	-1.128563000
6	-9.811950000	6.407415000	-2.962548000
6	-9.012159000	5.254502000	-2.393133000
6	-9.433590000	4.590834000	-1.227060000
6	-7.843424000	4.803980000	-3.032156000
6	-8.715461000	3.501900000	-0.718773000
6	-7.120746000	3.715103000	-2.529052000
6	-7.556428000	3.058532000	-1.370919000
1	-9.172332000	7.107867000	-3.510872000
1	-10.578630000	6.052736000	-3.665468000
1	-10.332570000	4.930057000	-0.717421000
1	-7.496933000	5.305162000	-3.932119000
1	-9.038522000	2.994993000	0.184256000
1	-6.221634000	3.386854000	-3.040275000
1	-7.009948000	2.210574000	-0.970505000
26	-0.280296000	-1.194562000	1.199623000
26	-1.832757000	-0.829993000	-1.673043000
8	0.296161000	-3.239102000	0.938259000
1	1.283371000	-3.258121000	0.849020000
1	-0.128545000	-3.852232000	1.663343000
8	-4.395392000	-0.835171000	3.984413000
1	-4.222003000	0.031823000	4.446096000
1	-4.317961000	-0.692513000	2.983432000
8	-4.117488000	-0.130699000	1.484443000
1	-4.851277000	-0.338300000	0.821124000
1	-4.005313000	0.849083000	1.635085000
8	-2.115471000	-1.450349000	0.334990000
1	-2.846805000	-2.563663000	-0.206280000
1	-2.800829000	-0.889108000	0.824673000
8	-1.002806000	-2.273683000	8.907267000
1	-0.576776000	-2.919939000	8.250054000
1	-1.051176000	-2.618059000	9.811461000
8	-3.497501000	-1.933264000	-1.937159000
8	-3.393754000	-3.039747000	-1.020110000
8	-0.344229000	-2.234599000	-1.504166000
1	0.322596000	-2.467341000	-2.222063000
8	-0.951838000	-4.615396000	2.635693000
1	-1.776686000	-4.891909000	2.159508000
1	-1.155539000	-4.384077000	3.575737000
8	-2.514104000	-0.985411000	7.341038000
1	-2.009010000	-1.366472000	8.137853000
1	-2.567918000	0.007338000	7.270483000
8	-1.894628000	-4.348747000	5.240243000
1	-2.219059000	-5.259966000	5.359653000
1	-2.666573000	-3.646283000	5.397280000
8	-0.036552000	-3.745205000	7.021469000
1	-0.721756000	-4.019198000	6.333326000
1	0.743075000	-3.367849000	6.579307000
8	-3.683504000	-2.634266000	5.703208000
1	-3.961661000	-2.024859000	4.937631000
1	-3.361646000	-2.035514000	6.461292000
1	3.759397000	2.915435000	7.104448000
1	3.727714000	6.324961000	3.623266000
1	-0.229119000	8.151723000	5.880759000
1	-1.219051000	9.031397000	1.188923000
1	-5.192870000	7.171774000	2.209768000
1	-4.911888000	8.995962000	-3.358974000
1	-1.988685000	8.145602000	-7.611536000

1	0.916985000	11.603279000	-5.399301000
1	7.017546000	5.956531000	-4.141276000
1	4.278972000	5.585380000	0.224790000
1	8.322945000	-0.030301000	-2.280826000
1	9.951418000	3.706043000	0.372696000
1	6.364725000	2.499180000	3.560840000
1	9.151790000	-1.018994000	4.240479000
1	4.472890000	0.139150000	-8.069179000
1	7.194118000	-1.714099000	-4.562391000
1	-7.247466000	-2.511081000	6.903982000
1	-6.968815000	1.323124000	4.035643000
1	-4.747164000	-4.906711000	4.098415000
1	-8.876573000	-1.797138000	0.214850000
1	8.449341000	-3.155483000	0.626191000
1	-10.330004000	6.965528000	-2.174181000
1	1.956962000	1.588186000	-4.543024000
1	4.040157000	-5.118627000	-2.994489000
1	-0.266270000	-2.833267000	-0.716480000
1	4.616145000	-0.465782000	4.058641000

^{1}IM1$_B$:

6	8.752115000	-3.031982000	3.199121000
6	8.048207000	-1.885246000	2.488128000
8	8.066695000	-1.830187000	1.222907000
6	7.843093000	-4.291706000	3.367552000
6	6.975947000	-4.605211000	2.188006000
7	7.432897000	-4.913071000	0.898321000
6	5.608997000	-4.590967000	2.088185000
6	6.385287000	-5.058318000	0.049152000
7	5.271779000	-4.868500000	0.768962000
1	9.628068000	-3.282916000	2.592914000
1	8.484567000	-5.144982000	3.622953000
1	7.176721000	-4.139308000	4.222888000
1	4.861480000	-4.395996000	2.835501000
1	6.423846000	-5.256126000	-1.024463000
1	4.266236000	-4.764146000	0.407623000
7	7.409636000	-0.967631000	3.255030000
6	6.512080000	0.058389000	2.711712000
6	7.032696000	0.846455000	1.540170000
8	6.240583000	1.224017000	0.630612000
1	7.291494000	-1.104186000	4.257955000
1	5.554364000	-0.365160000	2.396841000
7	8.341865000	1.180162000	1.455899000
6	8.815495000	1.951090000	0.303223000
6	8.407068000	1.340298000	-1.044508000
8	8.160412000	2.084627000	-2.025445000
1	8.976905000	0.921392000	2.196126000
1	8.408628000	2.965470000	0.314348000
7	8.367391000	-0.019999000	-1.122465000
6	8.014829000	-0.679204000	-2.373583000
6	6.526873000	-0.671759000	-2.741042000
8	6.188086000	-1.065046000	-3.889473000
1	8.447191000	-0.590380000	-0.280819000
1	8.534072000	-0.194512000	-3.204661000
7	5.653874000	-0.254265000	-1.797720000
6	4.219458000	-0.070047000	-2.072141000
6	3.879231000	1.429726000	-2.112709000
8	3.309658000	1.934781000	-3.121638000
6	3.320152000	-0.812032000	-1.063251000
6	3.493093000	-2.336997000	-1.095711000
6	2.557274000	-3.046267000	-0.129789000
8	1.529431000	-2.407578000	0.279160000
8	2.818774000	-4.273748000	0.231111000
1	6.011208000	0.077483000	-0.903570000
1	4.050003000	-0.445225000	-3.082442000
1	2.277095000	-0.570737000	-1.294368000
1	3.497810000	-0.433574000	-0.049653000
1	4.529223000	-2.621591000	-0.878366000
1	3.261435000	-2.739765000	-2.092989000
7	4.229825000	2.149327000	-1.019980000
6	3.928609000	3.565943000	-0.862416000
6	4.635034000	4.501882000	-1.830934000
8	4.178943000	5.672411000	-1.986096000
1	4.693301000	1.697695000	-0.233245000
1	2.857782000	3.765834000	-0.992033000
7	5.669107000	4.039968000	-2.569473000
6	6.152282000	4.817688000	-3.710032000
6	5.057202000	5.130778000	-4.743637000
8	5.214188000	6.088783000	-5.534634000
1	6.134769000	3.168138000	-2.339756000
1	6.551275000	5.785320000	-3.394611000
7	3.949556000	4.329840000	-4.742458000
6	2.808377000	4.593760000	-5.615669000
6	1.604858000	5.198986000	-4.867674000
8	0.485936000	5.279687000	-5.445240000
6	2.376344000	3.327807000	-6.375104000
1	3.877231000	3.519611000	-4.125988000
1	3.142526000	5.355450000	-6.331636000
1	1.476769000	3.533274000	-6.958831000
1	3.181322000	3.008489000	-7.044304000
1	2.156608000	2.519281000	-5.668695000
7	1.798225000	5.692914000	-3.613233000
6	0.662639000	6.155020000	-2.807627000
6	-0.026522000	7.401784000	-3.382903000
8	-1.192990000	7.695249000	-3.008400000
6	1.063960000	6.414195000	-1.347980000
8	0.982808000	5.153247000	-0.615172000
1	2.711418000	5.633135000	-3.159863000
1	-0.117996000	5.385279000	-2.808589000
1	2.079279000	6.820631000	-1.294957000
1	0.356011000	7.138020000	-0.926784000
1	1.136348000	5.292875000	0.350662000
7	0.666417000	8.137180000	-4.286217000
6	0.057653000	9.256691000	-4.996372000
6	-1.041314000	8.881948000	-6.002677000
8	-1.723436000	9.801791000	-6.508917000
1	1.596825000	7.831922000	-4.536359000
1	-0.410054000	9.940555000	-4.282556000
7	-1.220714000	7.558981000	-6.274313000
6	-2.327159000	7.103739000	-7.106258000
6	-3.552411000	6.594717000	-6.338508000
8	-4.502812000	6.078689000	-6.979740000
1	-0.597785000	6.846524000	-5.894832000
1	-2.654329000	7.938692000	-7.732649000
7	-3.543937000	6.744883000	-4.988038000
6	-4.677818000	6.360090000	-4.147709000
6	-4.335267000	5.299214000	-3.087335000
6	-3.998308000	3.896261000	-3.576903000
6	-4.275366000	3.437957000	-4.877759000
6	-3.438403000	2.981152000	-2.667401000
6	-4.008678000	2.114661000	-5.246805000
6	-3.174022000	1.655215000	-3.019354000
6	-3.460843000	1.219259000	-4.321181000
8	-3.216641000	-0.079829000	-4.739707000
1	-2.740069000	7.162812000	-4.531023000
1	-5.468418000	6.015045000	-4.818244000
1	-5.204346000	5.235894000	-2.411879000
1	-3.500897000	5.680402000	-2.478877000
1	-4.682815000	4.110592000	-5.625707000
1	-3.214895000	3.309688000	-1.653875000
1	-4.217791000	1.765432000	-6.251614000
1	-2.752498000	0.958180000	-2.302668000
1	-2.785837000	-0.656947000	-4.057867000
6	-4.711888000	4.454103000	2.269050000
6	-3.233620000	4.724797000	2.148799000
8	-2.362918000	3.851450000	2.425914000
6	-5.211099000	3.366119000	1.258711000
6	-5.953381000	2.212022000	1.955656000
6	-4.972454000	1.299127000	2.696996000
8	-4.049462000	0.702967000	2.075721000
8	-5.181730000	1.261456000	3.999324000
1	-4.882662000	4.090062000	3.290236000
1	-5.883542000	3.827136000	0.528450000
1	-4.369102000	2.944374000	0.700645000
1	-6.466719000	1.581211000	1.224460000
1	-6.714522000	2.586574000	2.645334000
7	-2.830344000	5.972712000	1.753103000
6	-1.427794000	6.225160000	1.407425000
6	-0.443263000	5.903133000	2.529103000
8	0.703616000	5.450663000	2.249497000

1	-3.525451000	6.636427000	1.441288000
1	-1.116586000	5.623345000	0.547374000
7	-0.817395000	6.159661000	3.807240000
6	0.063220000	5.860755000	4.940878000
6	0.177137000	4.363180000	5.282164000
8	1.249899000	3.915411000	5.758113000
1	-1.739142000	6.540089000	3.971980000
1	1.074254000	6.209883000	4.724575000
7	-0.929915000	3.614198000	5.063257000
6	-0.972011000	2.146128000	5.248470000
6	0.040955000	1.500435000	4.279027000
8	0.938116000	0.718721000	4.690067000
6	-2.427033000	1.700216000	5.001623000
6	-2.824223000	0.349792000	5.575575000
8	-2.199665000	-0.168492000	6.551422000
8	-3.874864000	-0.219517000	5.035577000
1	-1.673167000	4.006752000	4.497567000
1	-0.650693000	1.886442000	6.258464000
1	-2.652112000	1.690910000	3.934505000
1	-3.101288000	2.435392000	5.461558000
7	-0.057023000	1.891196000	2.982075000
6	0.864430000	1.387743000	1.957541000
6	2.305585000	1.837026000	2.237668000
8	3.280887000	1.079608000	1.982981000
6	0.411539000	1.828456000	0.545689000
6	-0.977128000	1.316730000	0.085399000
6	-1.079860000	-0.193343000	0.020971000
8	-0.783002000	-0.854632000	1.072398000
8	-1.464393000	-0.734792000	-1.103303000
1	-0.832861000	2.479730000	2.691941000
1	0.875936000	0.298510000	2.001205000
1	1.172868000	1.481788000	-0.162141000
1	0.404086000	2.922786000	0.477841000
1	-1.765395000	1.661374000	0.767178000
1	-1.184992000	1.733066000	-0.902192000
7	2.475882000	3.083112000	2.758642000
6	3.785533000	3.588721000	3.151623000
6	4.492973000	2.736496000	4.206394000
8	5.739536000	2.565146000	4.128471000
1	1.685495000	3.712674000	2.836584000
1	4.477980000	3.623973000	2.305855000
7	3.741925000	2.197587000	5.196733000
6	4.343547000	1.269933000	6.155730000
6	4.558679000	-0.144614000	5.589568000
8	5.693213000	-0.706987000	5.658686000
1	2.760271000	2.445671000	5.289845000
1	5.325649000	1.635587000	6.459473000
7	3.480976000	-0.749229000	5.041287000
6	3.594772000	-2.091135000	4.456048000
6	2.499744000	-2.374501000	3.422813000
6	1.139219000	-2.590146000	4.010741000
7	0.014851000	-2.808861000	3.206707000
6	0.725340000	-2.577271000	5.328298000
6	-1.041839000	-2.904892000	4.028791000
7	-0.645526000	-2.776336000	5.313675000
1	2.600890000	-0.240746000	4.926178000
1	3.579085000	-2.848062000	5.254357000
1	2.784306000	-3.263500000	2.842764000
1	2.468507000	-1.546316000	2.704196000
1	1.259672000	-2.370835000	6.239087000
1	-2.067985000	-3.032954000	3.730035000
6	1.370053000	-1.097275000	-4.705354000
6	2.245828000	-2.016932000	-5.525573000
8	2.148760000	-3.282230000	-5.458485000
6	0.013580000	-0.767063000	-5.394568000
6	-0.903021000	-1.974334000	-5.680588000
6	-1.303810000	-2.781706000	-4.450199000
8	-1.735033000	-2.023961000	-3.409430000
8	-1.238691000	-4.021983000	-4.421028000
1	1.170141000	-1.587912000	-3.746980000
1	-0.517804000	-0.065300000	-4.746513000
1	0.205834000	-0.242921000	-6.339986000
1	-0.443757000	-2.677406000	-6.380997000
1	-1.822394000	-1.584814000	-6.135002000
7	3.138276000	-1.453390000	-6.382329000
6	3.875397000	-2.244960000	-7.374853000
6	4.804826000	-3.328480000	-6.816179000
8	5.024894000	-4.356461000	-7.495074000
1	3.212738000	-0.446267000	-6.410738000
1	3.178942000	-2.770782000	-8.033820000
7	5.364703000	-3.097459000	-5.596225000
6	6.231986000	-4.070797000	-4.949889000
6	5.552611000	-4.849172000	-3.821002000
8	6.239426000	-5.300530000	-2.855303000
1	5.290943000	-2.197020000	-5.129091000
1	6.565719000	-4.788370000	-5.708501000
7	4.228813000	-5.064376000	-3.947375000
6	3.462140000	-5.902579000	-3.016734000
6	1.973388000	-5.904025000	-3.377494000
8	1.457913000	-4.538811000	-3.228912000
6	1.163136000	-6.830900000	-2.476943000
1	3.728680000	-4.599229000	-4.700722000
1	3.566628000	-5.523055000	-1.994637000
1	1.845101000	-6.203768000	-4.426509000
1	1.493223000	-4.053242000	-4.099636000
1	0.106804000	-6.786747000	-2.755825000
1	1.506994000	-7.866521000	-2.575858000
1	1.257396000	-6.531435000	-1.427785000
6	-5.470210000	-6.327051000	3.202655000
6	-6.651767000	-5.615646000	3.847125000
8	-7.732260000	-5.450911000	3.214205000
6	-5.946154000	-7.549106000	2.391912000
6	-4.848901000	-8.183600000	1.494066000
6	-4.306349000	-7.172512000	0.505447000
8	-3.348983000	-6.401805000	0.825259000
7	-4.926517000	-7.042222000	-0.683073000
1	-4.969074000	-5.612721000	2.536879000
1	-6.798241000	-7.236422000	1.778642000
1	-6.319351000	-8.322000000	3.075764000
1	-5.268208000	-9.052143000	0.972370000
1	-4.011553000	-8.534142000	2.106365000
1	-4.643767000	-6.261732000	-1.282778000
1	-5.697403000	-7.633042000	-0.951085000
7	-6.478484000	-5.162681000	5.117597000
6	-7.584158000	-4.555413000	5.855967000
6	-8.129771000	-3.238821000	5.295970000
8	-9.292010000	-2.879037000	5.594713000
1	-5.538132000	-5.131671000	5.517634000
1	-8.436993000	-5.239022000	5.884215000
7	-7.286665000	-2.513849000	4.511357000
6	-7.708304000	-1.272433000	3.884014000
6	-7.806130000	-1.337732000	2.360873000
8	-7.841743000	-0.263197000	1.703774000
1	-6.313283000	-2.791186000	4.370640000
1	-8.698359000	-1.021666000	4.280963000
7	-7.890095000	-2.569609000	1.793209000
6	-8.097638000	-2.755253000	0.356061000
6	-6.902329000	-3.347039000	-0.385214000
8	-5.818049000	-2.374659000	-0.454499000
1	-7.903400000	-3.393457000	2.390973000
1	-8.340962000	-1.779761000	-0.070517000
1	-6.550572000	-4.263537000	0.105864000
1	-7.213524000	-3.602394000	-1.405805000
1	-5.151489000	-2.614478000	-1.161829000
6	-9.885859000	4.698562000	-2.835262000
6	-9.067017000	3.522755000	-2.345105000
6	-9.423778000	2.832389000	-1.173580000
6	-7.944999000	3.076821000	-3.066579000
6	-8.690140000	1.721574000	-0.738469000
6	-7.207009000	1.966951000	-2.637894000
6	-7.578760000	1.283827000	-1.472411000
1	-10.651889000	4.378988000	-3.555488000
1	-10.405661000	5.197592000	-2.009625000
1	-10.285677000	3.166916000	-0.600613000
1	-7.647965000	3.597973000	-3.973112000
1	-8.962534000	1.196051000	0.170543000
1	-6.344574000	1.642732000	-3.210887000
1	-7.016157000	0.418527000	-1.133967000
26	-0.311141000	-2.900207000	1.136526000
26	-1.954896000	-2.626024000	-1.622258000

8	0.213019000	-4.952245000	0.806343000
1	1.200685000	-5.001550000	0.749535000
1	-0.274289000	-5.569856000	1.494909000
8	-4.439866000	-2.598678000	3.913208000
1	-4.271970000	-1.732265000	4.377282000
1	-4.352003000	-2.458971000	2.912561000
8	-4.116797000	-1.894767000	1.413723000
1	-4.895988000	-2.043636000	0.766973000
1	-3.980106000	-0.923370000	1.592663000
8	-2.119413000	-3.131402000	0.206414000
1	-3.277774000	-4.473797000	-0.414482000
1	-2.852421000	-2.616665000	0.693399000
8	-0.997622000	-4.119002000	8.757050000
1	-0.596856000	-4.752017000	8.072474000
1	-1.016294000	-4.483377000	9.654486000
8	-3.741862000	-3.244255000	-1.927077000
8	-3.893475000	-4.563834000	-1.198730000
8	-0.470937000	-3.949636000	-1.597104000
1	0.175580000	-4.193821000	-2.332999000
8	-1.201812000	-6.275888000	2.386626000
1	-2.048939000	-6.412800000	1.883930000
1	-1.365594000	-6.123393000	3.351536000
8	-2.531714000	-2.768259000	7.268631000
1	-2.015961000	-3.170465000	8.047103000
1	-2.588978000	-1.774339000	7.218562000
8	-1.970478000	-6.127794000	5.049299000
1	-2.297034000	-7.035574000	5.188927000
1	-2.724199000	-5.413099000	5.259482000
8	-0.073500000	-5.545232000	6.808597000
1	-0.764306000	-5.811898000	6.124374000
1	0.687515000	-5.132205000	6.365196000
8	-3.702403000	-4.396750000	5.609551000
1	-3.996297000	-3.777288000	4.855204000
1	-3.376626000	-3.809746000	6.374318000
1	3.689713000	1.216439000	7.031141000
1	3.647180000	4.605860000	3.530221000
1	-0.307370000	6.401019000	5.817159000
1	-1.331389000	7.281301000	1.134707000
1	-5.287886000	5.379267000	2.156875000
1	-5.053341000	7.254346000	-3.631838000
1	-1.994010000	6.293273000	-7.760028000
1	0.841499000	9.805731000	-5.524548000
1	6.958100000	4.252745000	-4.186358000
1	4.189111000	3.850805000	0.160177000
1	8.346797000	-1.720595000	-2.327787000
1	9.906668000	2.014139000	0.356406000
1	6.304480000	0.778315000	3.511664000
1	9.109833000	-2.730400000	4.189271000
1	4.463684000	-1.551622000	-7.983890000
1	7.103020000	-3.567484000	-4.528106000
1	-7.250192000	-4.386925000	6.884741000
1	-7.024704000	-0.456117000	4.134024000
1	-4.731325000	-6.619907000	3.957188000
1	-8.955529000	-3.424303000	0.210261000
1	8.398407000	-4.930981000	0.609032000
1	-9.259920000	5.441960000	-3.341852000
1	1.893549000	-0.157043000	-4.489994000
1	3.858710000	-6.925666000	-3.040574000
1	-0.351372000	-4.515568000	-0.784303000
1	4.579152000	-2.160422000	3.981319000

^{1}TS2$_{B}$:

6	8.752949000	-3.039266000	3.158650000
6	8.064286000	-1.888533000	2.438799000
8	8.105334000	-1.825267000	1.174453000
6	7.814259000	-4.270249000	3.366121000
6	6.942042000	-4.594358000	2.193326000
7	7.396900000	-4.916627000	0.906580000
6	5.574990000	-4.573869000	2.093631000
6	6.347543000	-5.064122000	0.059678000
7	5.234849000	-4.861283000	0.777147000
1	9.612759000	-3.322725000	2.544003000
1	8.434756000	-5.131050000	3.647181000
1	7.151288000	-4.075265000	4.215471000
1	4.829036000	-4.368480000	2.839533000
1	6.391218000	-5.270550000	-1.012207000
1	4.225787000	-4.744935000	0.411618000
7	7.411391000	-0.977812000	3.201903000
6	6.511260000	0.043825000	2.656093000
6	7.034535000	0.845540000	1.496022000
8	6.243787000	1.234669000	0.589199000
1	7.274046000	-1.125120000	4.201644000
1	5.558933000	-0.384764000	2.331603000
7	8.343140000	1.180973000	1.418211000
6	8.819374000	1.962493000	0.274065000
6	8.413995000	1.363676000	-1.080154000
8	8.164321000	2.116680000	-2.053805000
1	8.977438000	0.907976000	2.153929000
1	8.412105000	2.976552000	0.293550000
7	8.379851000	0.003716000	-1.170223000
6	8.031781000	-0.647319000	-2.427169000
6	6.543357000	-0.647309000	-2.792955000
8	6.202533000	-1.045045000	-3.939806000
1	8.464228000	-0.573660000	-0.333419000
1	8.547518000	-0.151566000	-3.254004000
7	5.672459000	-0.228019000	-1.849194000
6	4.235808000	-0.056681000	-2.115515000
6	3.875949000	1.438282000	-2.138748000
8	3.292821000	1.947456000	-3.138917000
6	3.346249000	-0.810935000	-1.108731000
6	3.521133000	-2.335880000	-1.143597000
6	2.550341000	-3.033111000	-0.201494000
8	1.510432000	-2.386734000	0.148670000
8	2.802333000	-4.253097000	0.200553000
1	6.030929000	0.101922000	-0.954920000
1	4.066622000	-0.426487000	-3.127756000
1	2.300624000	-0.575271000	-1.333133000
1	3.524055000	-0.435721000	-0.093792000
1	4.549765000	-2.623577000	-0.897537000
1	3.313692000	-2.735631000	-2.147178000
7	4.217997000	2.149228000	-1.037929000
6	3.889677000	3.556634000	-0.856575000
6	4.570578000	4.518583000	-1.817607000
8	4.088144000	5.680532000	-1.960234000
1	4.692140000	1.694683000	-0.258648000
1	2.814182000	3.737096000	-0.974175000
7	5.611700000	4.087242000	-2.564626000
6	6.072576000	4.887249000	-3.699232000
6	4.963329000	5.192748000	-4.719844000
8	5.090847000	6.167299000	-5.495755000
1	6.092560000	3.220216000	-2.348044000
1	6.456930000	5.857856000	-3.375074000
7	3.874873000	4.365610000	-4.724565000
6	2.722767000	4.616421000	-5.586406000
6	1.510377000	5.178876000	-4.820157000
8	0.380728000	5.229857000	-5.382661000
6	2.318600000	3.354457000	-6.367163000
1	3.824880000	3.544252000	-4.120002000
1	3.033760000	5.399642000	-6.289851000
1	1.409790000	3.546917000	-6.941060000
1	3.126669000	3.069650000	-7.047836000
1	2.127342000	2.526574000	-5.675143000
7	1.703487000	5.672983000	-3.566603000
6	0.563546000	6.107441000	-2.751372000
6	-0.171124000	7.321606000	-3.338458000
8	-1.359542000	7.559613000	-2.993443000
6	0.977029000	6.397220000	-1.299665000
8	0.911951000	5.147038000	-0.549363000
1	2.621240000	5.629535000	-3.118957000
1	-0.191316000	5.313320000	-2.729139000
1	1.989245000	6.812913000	-1.263814000
1	0.268038000	7.122645000	-0.881998000
1	1.097451000	5.294572000	0.410082000
7	0.511850000	8.096205000	-4.216653000
6	-0.121143000	9.204222000	-4.923968000
6	-1.194435000	8.807518000	-5.948470000
8	-1.901317000	9.709366000	-6.452558000
1	1.459944000	7.832028000	-4.447506000
1	-0.618122000	9.867828000	-4.210637000
7	-1.327932000	7.482332000	-6.237640000

6	-2.409623000	7.002399000	-7.086316000
6	-3.615866000	6.433388000	-6.333451000
8	-4.523567000	5.848890000	-6.979086000
1	-0.691269000	6.783910000	-5.854445000
1	-2.765293000	7.838386000	-7.696326000
7	-3.645607000	6.609452000	-4.985388000
6	-4.789150000	6.202701000	-4.169107000
6	-4.442449000	5.162948000	-3.090614000
6	-4.044324000	3.773779000	-3.550190000
6	-4.228623000	3.303956000	-4.873520000
6	-3.504344000	2.880380000	-2.588782000
6	-3.888220000	2.003691000	-5.217471000
6	-3.162622000	1.580379000	-2.910121000
6	-3.338729000	1.105584000	-4.248976000
8	-2.995826000	-0.115830000	-4.602201000
1	-2.870558000	7.069025000	-4.516818000
1	-5.555656000	5.831736000	-4.853669000
1	-5.324568000	5.063707000	-2.436303000
1	-3.638547000	5.571927000	-2.459987000
1	-4.621022000	3.965238000	-5.639592000
1	-3.364311000	3.231246000	-1.569300000
1	-4.021750000	1.638096000	-6.229389000
1	-2.750074000	0.895907000	-2.177485000
1	-2.360018000	-1.116189000	-3.882730000
6	-4.687049000	4.536045000	2.444220000
6	-3.210106000	4.799353000	2.295117000
8	-2.339660000	3.913316000	2.524411000
6	-5.217480000	3.488094000	1.407415000
6	-5.957061000	2.319374000	2.082107000
6	-4.966012000	1.398682000	2.801841000
8	-4.047694000	0.815547000	2.161483000
8	-5.162057000	1.346200000	4.104876000
1	-4.834842000	4.133935000	3.454403000
1	-5.896643000	3.980608000	0.703832000
1	-4.387444000	3.072730000	0.826432000
1	-6.473417000	1.699584000	1.343331000
1	-6.712152000	2.681603000	2.785306000
7	-2.805938000	6.054180000	1.919152000
6	-1.409696000	6.296745000	1.543128000
6	-0.402272000	5.947574000	2.636472000
8	0.730582000	5.482886000	2.323905000
1	-3.501578000	6.735636000	1.650449000
1	-1.126123000	5.699672000	0.670369000
7	-0.745503000	6.194963000	3.925034000
6	0.153125000	5.872057000	5.037358000
6	0.247089000	4.370886000	5.368079000
8	1.314612000	3.907033000	5.839023000
1	-1.659403000	6.583259000	4.113454000
1	1.165437000	6.204478000	4.801500000
7	-0.870574000	3.638229000	5.145698000
6	-0.936679000	2.170614000	5.328800000
6	0.062517000	1.509595000	4.355724000
8	0.969714000	0.740632000	4.767565000
6	-2.399752000	1.750384000	5.087266000
6	-2.805080000	0.395236000	5.642277000
8	-2.171130000	-0.147604000	6.597511000
8	-3.870224000	-0.155843000	5.109962000
1	-1.609087000	4.044096000	4.583621000
1	-0.615567000	1.903986000	6.336976000
1	-2.635554000	1.761028000	4.022436000
1	-3.059598000	2.486513000	5.566084000
7	-0.053658000	1.878784000	3.053504000
6	0.855330000	1.362723000	2.022822000
6	2.299964000	1.815466000	2.275844000
8	3.273993000	1.070891000	1.982954000
6	0.383032000	1.796924000	0.616644000
6	-0.992062000	1.243686000	0.167678000
6	-1.024177000	-0.264601000	0.006635000
8	-0.656916000	-0.957914000	1.023277000
8	-1.410632000	-0.760516000	-1.128704000
1	-0.831003000	2.465801000	2.765009000
1	0.861270000	0.272820000	2.069291000
1	1.148429000	1.475022000	-0.098560000
1	0.344630000	2.891689000	0.557562000
1	-1.778928000	1.513549000	0.884410000
1	-1.243329000	1.703760000	-0.790235000
7	2.475672000	3.055070000	2.812350000
6	3.790270000	3.559434000	3.189246000
6	4.515586000	2.694450000	4.221909000
8	5.762919000	2.535685000	4.128716000
1	1.683885000	3.677733000	2.916769000
1	4.468563000	3.606626000	2.332575000
7	3.777001000	2.130208000	5.206804000
6	4.385615000	1.179576000	6.138210000
6	4.572867000	-0.227165000	5.543230000
8	5.704389000	-0.800328000	5.578513000
1	2.792209000	2.360668000	5.305326000
1	5.377080000	1.530111000	6.428424000
7	3.477982000	-0.810364000	5.008110000
6	3.561298000	-2.141381000	4.392140000
6	2.469055000	-2.376864000	3.343358000
6	1.098282000	-2.574544000	3.913358000
7	-0.016091000	-2.774605000	3.092668000
6	0.670144000	-2.564694000	5.227737000
6	-1.082227000	-2.864052000	3.899623000
7	-0.702534000	-2.747072000	5.194951000
1	2.599876000	-0.292086000	4.928431000
1	3.518688000	-2.915277000	5.173088000
1	2.738430000	-3.260873000	2.748755000
1	2.463094000	-1.535707000	2.639157000
1	1.194046000	-2.369281000	6.147270000
1	-2.104060000	-2.982767000	3.584481000
6	1.444126000	-1.124310000	-4.753992000
6	2.307139000	-2.061368000	-5.572754000
8	2.195229000	-3.322386000	-5.501016000
6	0.118399000	-0.722628000	-5.461772000
6	-0.851624000	-1.883233000	-5.791311000
6	-1.238771000	-2.717043000	-4.582574000
8	-1.860644000	-2.026330000	-3.560983000
8	-0.980718000	-3.916142000	-4.475391000
1	1.213762000	-1.625180000	-3.807766000
1	-0.392920000	-0.004188000	-4.814264000
1	0.349126000	-0.194725000	-6.396153000
1	-0.414929000	-2.572775000	-6.517665000
1	-1.758645000	-1.443315000	-6.220443000
7	3.207085000	-1.504889000	-6.428897000
6	3.945293000	-2.306136000	-7.411819000
6	4.880024000	-3.377994000	-6.839490000
8	5.114175000	-4.407723000	-7.510826000
1	3.301749000	-0.499458000	-6.449891000
1	3.249458000	-2.843723000	-8.061831000
7	5.427048000	-3.133161000	-5.616709000
6	6.298249000	-4.093888000	-4.957371000
6	5.623361000	-4.860782000	-3.818721000
8	6.318086000	-5.301386000	-2.853710000
1	5.342713000	-2.228828000	-5.158404000
1	6.636582000	-4.818887000	-5.706739000
7	4.298803000	-5.078549000	-3.932246000
6	3.546449000	-5.908015000	-2.981698000
6	2.058425000	-5.956969000	-3.341560000
8	1.503666000	-4.605415000	-3.226063000
6	1.279783000	-6.877570000	-2.406664000
1	3.789504000	-4.623186000	-4.685147000
1	3.636029000	-5.498029000	-1.970134000
1	1.938425000	-6.294144000	-4.380581000
1	1.501314000	-4.146063000	-4.106197000
1	0.220903000	-6.876839000	-2.678881000
1	1.654758000	-7.905316000	-2.468365000
1	1.369360000	-6.534057000	-1.370788000
6	-5.546408000	-6.276850000	3.115505000
6	-6.698683000	-5.512158000	3.750884000
8	-7.770984000	-5.314533000	3.112168000
6	-6.072569000	-7.548280000	2.415076000
6	-4.992689000	-8.324658000	1.617223000
6	-4.274210000	-7.411614000	0.640391000
8	-3.148659000	-6.910203000	0.923309000
7	-4.917246000	-7.073545000	-0.495585000
1	-5.070304000	-5.618016000	2.377406000
1	-6.892218000	-7.249043000	1.752827000
1	-6.507190000	-8.225661000	3.161335000

1	-5.466999000	-9.161422000	1.088934000
1	-4.239404000	-8.741033000	2.292869000
1	-4.546813000	-6.278898000	-1.032960000
1	-5.825388000	-7.448974000	-0.719722000
7	-6.519711000	-5.066001000	5.022913000
6	-7.616169000	-4.444067000	5.763889000
6	-8.162846000	-3.132176000	5.193891000
8	-9.332837000	-2.780216000	5.473024000
1	-5.579800000	-5.053691000	5.425843000
1	-8.472121000	-5.123020000	5.809259000
7	-7.311175000	-2.402013000	4.424781000
6	-7.731498000	-1.168639000	3.781558000
6	-7.797334000	-1.248464000	2.256556000
8	-7.802289000	-0.180867000	1.584263000
1	-6.331571000	-2.671906000	4.302277000
1	-8.731772000	-0.924593000	4.156993000
7	-7.892845000	-2.484428000	1.705743000
6	-8.075215000	-2.696153000	0.268561000
6	-6.873333000	-3.317679000	-0.437087000
8	-5.788947000	-2.350043000	-0.549921000
1	-7.916949000	-3.301316000	2.313799000
1	-8.304008000	-1.728538000	-0.183164000
1	-6.523129000	-4.209355000	0.097842000
1	-7.179619000	-3.622990000	-1.446026000
1	-5.094502000	-2.675457000	-1.202953000
6	-9.644926000	4.816530000	-2.998910000
6	-8.911154000	3.603431000	-2.466361000
6	-9.241798000	3.050972000	-1.216125000
6	-7.904269000	2.980046000	-3.224651000
6	-8.594600000	1.904548000	-0.738980000
6	-7.252128000	1.834424000	-2.751388000
6	-7.593682000	1.291854000	-1.506450000
1	-10.546652000	4.524761000	-3.554990000
1	-9.967197000	5.480279000	-2.188333000
1	-10.019112000	3.519693000	-0.616839000
1	-7.635341000	3.389824000	-4.195235000
1	-8.854111000	1.475874000	0.222933000
1	-6.478771000	1.369302000	-3.354477000
1	-7.093819000	0.404673000	-1.129981000
26	-0.303753000	-2.899453000	1.009339000
26	-2.020195000	-2.637851000	-1.628671000
8	0.180180000	-4.829230000	0.837498000
1	1.166189000	-4.898372000	0.763074000
1	-0.294410000	-5.466459000	1.537405000
8	-4.479038000	-2.503835000	3.890275000
1	-4.288941000	-1.653194000	4.369468000
1	-4.400492000	-2.347920000	2.886115000
8	-4.190254000	-1.788537000	1.412679000
1	-4.915352000	-1.963292000	0.716002000
1	-4.051796000	-0.818536000	1.570589000
8	-2.140453000	-3.153185000	0.266368000
1	-3.180411000	-4.377101000	-0.289671000
1	-2.827385000	-2.597987000	0.741879000
8	-0.924969000	-4.110497000	8.651073000
1	-0.527588000	-4.732315000	7.952355000
1	-0.924964000	-4.485227000	9.544515000
8	-3.754664000	-3.424353000	-1.920450000
8	-3.914201000	-4.586814000	-0.955799000
8	-0.571519000	-4.039995000	-1.652640000
1	0.081027000	-4.281631000	-2.367006000
8	-1.071882000	-6.282773000	2.412446000
1	-1.854851000	-6.638992000	1.914774000
1	-1.309287000	-6.081193000	3.352932000
8	-2.537270000	-2.767647000	7.228865000
1	-1.981853000	-3.184710000	7.971727000
1	-2.576225000	-1.772819000	7.205221000
8	-2.005851000	-6.076272000	5.000193000
1	-2.341966000	-6.982184000	5.126849000
1	-2.757908000	-5.357874000	5.197068000
8	-0.036918000	-5.510816000	6.675130000
1	-0.764500000	-5.777486000	6.029403000
1	0.677982000	-5.059843000	6.192414000
8	-3.741923000	-4.342272000	5.546783000
1	-4.027816000	-3.705893000	4.803020000
1	-3.407803000	-3.773536000	6.321730000
1	3.748369000	1.118422000	7.025265000
1	3.656525000	4.571336000	3.582964000
1	-0.188139000	6.413041000	5.925031000
1	-1.307387000	7.355288000	1.282416000
1	-5.259063000	5.468378000	2.380911000
1	-5.199192000	7.089551000	-3.668394000
1	-2.044415000	6.219366000	-7.755932000
1	0.653082000	9.781359000	-5.436130000
1	6.884370000	4.342487000	-4.188812000
1	4.152714000	3.831220000	0.168302000
1	8.370953000	-1.686684000	-2.390415000
1	9.910396000	2.025189000	0.330518000
1	6.293441000	0.757103000	3.459284000
1	9.133148000	-2.726917000	4.137139000
1	4.529821000	-1.619397000	-8.031848000
1	7.167195000	-3.581983000	-4.541053000
1	-7.269972000	-4.265185000	6.786972000
1	-7.060093000	-0.345586000	4.041481000
1	-4.779736000	-6.524455000	3.857952000
1	-8.936895000	-3.360728000	0.124336000
1	8.361833000	-4.942189000	0.616051000
1	-9.017936000	5.397473000	-3.684527000
1	1.995607000	-0.206802000	-4.511532000
1	3.968963000	-6.920764000	-2.976195000
1	-0.430016000	-4.554593000	-0.817345000
1	4.548818000	-2.220766000	3.926217000

^{1}IM2$_{B}$:

6	8.701024000	-2.895547000	2.635564000
6	7.923830000	-1.740836000	2.020511000
8	7.852154000	-1.643483000	0.760040000
6	7.867480000	-4.215251000	2.732132000
6	6.955482000	-4.476424000	1.574068000
7	7.355063000	-4.710357000	0.249825000
6	5.585737000	-4.467686000	1.537236000
6	6.269348000	-4.817644000	-0.557403000
7	5.190682000	-4.675623000	0.222410000
1	9.571921000	-3.056766000	1.992345000
1	8.564745000	-5.049438000	2.881652000
1	7.239937000	-4.174846000	3.628517000
1	4.871719000	-4.320997000	2.327300000
1	6.244263000	-4.960967000	-1.639386000
1	4.166108000	-4.567549000	-0.080103000
7	7.330109000	-0.854876000	2.858320000
6	6.402170000	0.187512000	2.401645000
6	6.871072000	1.030796000	1.245100000
8	6.036795000	1.471137000	0.403776000
1	7.277265000	-1.025705000	3.861319000
1	5.435748000	-0.230465000	2.105926000
7	8.183575000	1.335567000	1.108533000
6	8.626286000	2.135570000	-0.034813000
6	8.142033000	1.586202000	-1.383745000
8	7.884099000	2.377397000	-2.325507000
1	8.846598000	1.021027000	1.800969000
1	8.248844000	3.159255000	0.030730000
7	8.040113000	0.234086000	-1.506009000
6	7.600047000	-0.371618000	-2.756866000
6	6.088336000	-0.388389000	-3.011093000
8	5.672029000	-0.838987000	-4.110613000
1	8.141331000	-0.369680000	-0.689748000
1	8.043232000	0.169737000	-3.597376000
7	5.279255000	0.083955000	-2.035250000
6	3.828870000	0.262078000	-2.231999000
6	3.503920000	1.763099000	-2.148348000
8	3.021361000	2.388153000	-3.135096000
6	2.978257000	-0.570506000	-1.247595000
6	3.214056000	-2.084147000	-1.357241000
6	2.359264000	-2.889061000	-0.384193000
8	1.325056000	-2.332955000	0.115670000
8	2.705034000	-4.118341000	-0.109553000
1	5.687751000	0.439424000	-1.171823000
1	3.618578000	-0.033543000	-3.261404000
1	1.921281000	-0.363261000	-1.449230000
1	3.158905000	-0.242414000	-0.217928000
1	4.270169000	-2.326399000	-1.190553000

1	2.975068000	-2.445868000	-2.368537000
7	3.791696000	2.376622000	-0.974700000
6	3.511132000	3.786983000	-0.752273000
6	4.373292000	4.767750000	-1.530867000
8	4.001137000	5.977162000	-1.601689000
1	4.205165000	1.860760000	-0.200472000
1	2.475815000	4.026776000	-1.021037000
7	5.461931000	4.316048000	-2.187844000
6	6.165007000	5.171254000	-3.142153000
6	5.262112000	5.699115000	-4.264154000
8	5.593439000	6.730778000	-4.891251000
1	5.816601000	3.378253000	-2.041287000
1	6.581314000	6.055721000	-2.652509000
7	4.111953000	5.003497000	-4.521157000
6	3.135895000	5.478847000	-5.499609000
6	1.832131000	5.957391000	-4.835383000
8	0.777657000	6.082725000	-5.518521000
6	2.852710000	4.426434000	-6.583226000
1	3.907000000	4.126881000	-4.044822000
1	3.594314000	6.362349000	-5.964454000
1	2.061628000	4.776738000	-7.249658000
1	3.762644000	4.248752000	-7.163587000
1	2.533062000	3.478292000	-6.139457000
7	1.866205000	6.305541000	-3.515847000
6	0.620884000	6.595367000	-2.795601000
6	-0.104422000	7.852373000	-3.289519000
8	-1.331678000	7.992255000	-3.034389000
6	0.822679000	6.660582000	-1.272023000
8	0.571428000	5.328522000	-0.719859000
1	2.721281000	6.191502000	-2.966767000
1	-0.092213000	5.788938000	-2.993229000
1	1.839867000	6.985464000	-1.031474000
1	0.100122000	7.372508000	-0.855545000
1	0.692148000	5.330746000	0.261945000
7	0.605621000	8.758158000	-4.002090000
6	-0.036259000	9.909398000	-4.630972000
6	-1.056049000	9.570571000	-5.729771000
8	-1.843512000	10.464988000	-6.113094000
1	1.583406000	8.568657000	-4.174343000
1	-0.585342000	10.490759000	-3.885483000
7	-1.045611000	8.299694000	-6.221422000
6	-2.059524000	7.839798000	-7.161265000
6	-2.999090000	6.775345000	-6.592131000
8	-3.651815000	6.036841000	-7.373046000
1	-0.350378000	7.616987000	-5.918391000
1	-2.649710000	8.711135000	-7.464500000
7	-3.087910000	6.695192000	-5.235092000
6	-3.940912000	5.711830000	-4.572177000
6	-3.147524000	4.690906000	-3.731112000
6	-2.121532000	3.850828000	-4.463517000
6	-2.242202000	3.526054000	-5.841983000
6	-1.004874000	3.348081000	-3.737306000
6	-1.305778000	2.721430000	-6.460837000
6	-0.053656000	2.548554000	-4.337507000
6	-0.185558000	2.184729000	-5.727959000
8	0.663805000	1.390016000	-6.304935000
1	-2.533353000	7.314313000	-4.649932000
1	-4.528883000	5.223323000	-5.353073000
1	-3.876060000	4.016246000	-3.249275000
1	-2.644451000	5.225988000	-2.914496000
1	-3.053061000	3.944222000	-6.428414000
1	-0.889935000	3.622725000	-2.691829000
1	-1.383858000	2.470808000	-7.512926000
1	0.827912000	2.209898000	-3.803876000
1	-3.637654000	-3.580575000	-2.992857000
6	-4.965243000	4.151279000	2.532489000
6	-3.521143000	4.505533000	2.278992000
8	-2.583815000	3.677315000	2.454397000
6	-5.484434000	3.000457000	1.608802000
6	-6.189209000	1.888086000	2.407710000
6	-5.157920000	1.032477000	3.151166000
8	-4.261927000	0.411625000	2.515323000
8	-5.289449000	1.077594000	4.462766000
1	-5.021337000	3.811336000	3.575192000
1	-6.179589000	3.408025000	0.867673000
1	-4.653720000	2.547604000	1.058607000
1	-6.728892000	1.207807000	1.743011000
1	-6.916086000	2.300669000	3.113050000
7	-3.219518000	5.784033000	1.887964000
6	-1.858469000	6.129369000	1.463418000
6	-0.792224000	5.821567000	2.513975000
8	0.346213000	5.403396000	2.160180000
1	-3.970565000	6.421020000	1.663121000
1	-1.567764000	5.582724000	0.560655000
7	-1.093446000	6.063525000	3.814776000
6	-0.133108000	5.801211000	4.891032000
6	0.055349000	4.312089000	5.233258000
8	1.165958000	3.912586000	5.663407000
1	-2.013936000	6.415631000	4.038848000
1	0.849382000	6.184548000	4.610111000
7	-1.028239000	3.517381000	5.067310000
6	-1.007982000	2.051468000	5.271307000
6	-0.039605000	1.425261000	4.245281000
8	0.920032000	0.695127000	4.607430000
6	-2.456470000	1.548099000	5.128947000
6	-2.778958000	0.245211000	5.840357000
8	-2.017442000	-0.237231000	6.732284000
8	-3.907895000	-0.332245000	5.499662000
1	-1.813620000	3.878384000	4.539969000
1	-0.612161000	1.817985000	6.261026000
1	-2.727806000	1.422943000	4.078227000
1	-3.140572000	2.298856000	5.545271000
7	-0.241077000	1.786826000	2.951098000
6	0.647250000	1.319606000	1.881082000
6	2.084213000	1.818869000	2.114496000
8	3.078056000	1.115916000	1.782673000
6	0.120494000	1.754613000	0.489545000
6	-1.256702000	1.183461000	0.050319000
6	-1.327588000	-0.333276000	0.014093000
8	-0.970315000	-0.948899000	1.078603000
8	-1.755635000	-0.922747000	-1.064976000
1	-1.063301000	2.328130000	2.699791000
1	0.695349000	0.230620000	1.910149000
1	0.878262000	1.458117000	-0.243526000
1	0.059209000	2.849878000	0.450054000
1	-2.044250000	1.519763000	0.737692000
1	-1.496302000	1.575255000	-0.942094000
7	2.230308000	3.047714000	2.680645000
6	3.528520000	3.597799000	3.058769000
6	4.319221000	2.747130000	4.053238000
8	5.565396000	2.627246000	3.905758000
1	1.415028000	3.631308000	2.819658000
1	4.193042000	3.703154000	2.197668000
7	3.645953000	2.160564000	5.070860000
6	4.342482000	1.247405000	5.977711000
6	4.602259000	-0.138233000	5.363848000
8	5.757941000	-0.657953000	5.389327000
1	2.658278000	2.354116000	5.210813000
1	5.317164000	1.659420000	6.243390000
7	3.534962000	-0.765449000	4.820914000
6	3.677516000	-2.091884000	4.207856000
6	2.527031000	-2.407910000	3.249340000
6	1.211581000	-2.634004000	3.927606000
7	0.042532000	-2.854856000	3.194254000
6	0.881599000	-2.622592000	5.269331000
6	-0.960774000	-2.954917000	4.079314000
7	-0.486425000	-2.825493000	5.339170000
1	2.635442000	-0.286194000	4.743884000
1	3.749876000	-2.857517000	4.994448000
1	2.788935000	-3.298260000	2.660107000
1	2.433834000	-1.588002000	2.526241000
1	1.469169000	-2.418510000	6.147398000
1	-2.000759000	-3.094535000	3.841451000
6	0.202158000	-1.498471000	-4.392056000
6	1.182404000	-2.056608000	-5.402154000
8	1.318317000	-3.318874000	-5.556758000
6	-1.208645000	-1.260867000	-5.000109000
6	-1.877911000	-2.493449000	-5.629711000
6	-2.537623000	-3.493808000	-4.707923000
8	-2.982807000	-2.966036000	-3.505235000

8	-2.767906000	-4.663001000	-5.018265000
1	0.138631000	-2.199849000	-3.554800000
1	-1.846395000	-0.858659000	-4.207134000
1	-1.118712000	-0.490958000	-5.772991000
1	-1.187950000	-3.054743000	-6.267206000
1	-2.686536000	-2.143082000	-6.289456000
7	1.880454000	-1.190726000	-6.164135000
6	2.689789000	-1.642777000	-7.306441000
6	3.713495000	-2.743220000	-7.001715000
8	3.892409000	-3.671325000	-7.821110000
1	1.650629000	-0.193362000	-6.101456000
1	2.050810000	-2.052849000	-8.094075000
7	4.411879000	-2.632049000	-5.833942000
6	5.386024000	-3.631380000	-5.416707000
6	4.898422000	-4.520810000	-4.270581000
8	5.718613000	-4.973606000	-3.415121000
1	4.400694000	-1.779773000	-5.281888000
1	5.610487000	-4.267191000	-6.281113000
7	3.584789000	-4.826478000	-4.260645000
6	2.993493000	-5.757044000	-3.287992000
6	1.549717000	-6.126843000	-3.657064000
8	0.699961000	-4.939828000	-3.592617000
6	0.970172000	-7.162514000	-2.697740000
1	2.972019000	-4.365851000	-4.931540000
1	2.987490000	-5.310377000	-2.286524000
1	1.520597000	-6.513031000	-4.685632000
1	0.797008000	-4.354362000	-4.399114000
1	-0.069858000	-7.370431000	-2.962745000
1	1.537787000	-8.098400000	-2.744211000
1	0.993014000	-6.791977000	-1.666722000
6	-5.301734000	-6.534336000	3.814457000
6	-6.459279000	-5.828848000	4.505995000
8	-7.586252000	-5.733406000	3.941837000
6	-5.771586000	-7.865247000	3.191824000
6	-4.708794000	-8.542632000	2.287410000
6	-4.237073000	-7.597738000	1.198088000
8	-3.160132000	-6.943704000	1.316012000
7	-5.050753000	-7.402951000	0.139809000
1	-4.927504000	-5.867136000	3.026737000
1	-6.684468000	-7.665805000	2.619915000
1	-6.049292000	-8.567042000	3.988670000
1	-5.134331000	-9.454390000	1.849570000
1	-3.832191000	-8.832858000	2.874479000
1	-4.806046000	-6.649046000	-0.511128000
1	-5.914354000	-7.910687000	0.032329000
7	-6.216452000	-5.311789000	5.738912000
6	-7.289233000	-4.713814000	6.532727000
6	-7.961138000	-3.470159000	5.940839000
8	-9.127344000	-3.175621000	6.292370000
1	-5.250988000	-5.243676000	6.069597000
1	-8.094952000	-5.437593000	6.683657000
7	-7.218745000	-2.733430000	5.070588000
6	-7.763197000	-1.560073000	4.405590000
6	-7.942059000	-1.723141000	2.895025000
8	-8.049620000	-0.697686000	2.168472000
1	-6.233710000	-2.951559000	4.894574000
1	-8.744514000	-1.356110000	4.848831000
7	-8.020415000	-2.994566000	2.425170000
6	-8.271285000	-3.304837000	1.018810000
6	-7.089723000	-3.963131000	0.311143000
8	-6.003711000	-3.012911000	0.144440000
1	-7.954674000	-3.772190000	3.079728000
1	-8.524908000	-2.370473000	0.513556000
1	-6.743237000	-4.836775000	0.880036000
1	-7.419210000	-4.306938000	-0.678428000
1	-5.351850000	-3.290060000	-0.586234000
6	-8.024251000	3.849480000	-3.224469000
6	-7.511536000	2.580568000	-2.575716000
6	-8.155013000	2.034106000	-1.450770000
6	-6.402285000	1.897305000	-3.106060000
6	-7.713647000	0.834443000	-0.876857000
6	-5.954334000	0.699457000	-2.534781000
6	-6.608937000	0.162656000	-1.418933000
1	-8.741662000	3.625877000	-4.025958000
1	-8.539013000	4.491169000	-2.500612000
1	-9.016803000	2.547218000	-1.030170000
1	-5.898073000	2.298903000	-3.982441000
1	-8.215242000	0.417818000	-0.008045000
1	-5.098453000	0.183157000	-2.960675000
1	-6.277743000	-0.770114000	-0.972931000
26	-0.402875000	-2.980838000	1.138373000
26	-2.307597000	-2.866725000	-1.303962000
8	0.257023000	-5.003753000	0.765252000
1	1.231122000	-4.980460000	0.595555000
1	-0.101231000	-5.632828000	1.513218000
8	-4.408250000	-2.758562000	4.409981000
1	-4.253136000	-1.876387000	4.844131000
1	-4.387745000	-2.650003000	3.395869000
8	-4.299447000	-2.211332000	1.889189000
1	-5.079514000	-2.480744000	1.286465000
1	-4.203238000	-1.225797000	1.967848000
8	-2.268721000	-3.416737000	0.585752000
1	-3.254776000	-4.832762000	-0.075453000
1	-2.980573000	-2.954461000	1.124896000
8	-0.331554000	-4.074143000	8.809348000
1	0.004515000	-4.717791000	8.098138000
1	-0.246937000	-4.422170000	9.709808000
8	-4.129842000	-3.750175000	-1.487458000
8	-3.894632000	-5.096300000	-0.805079000
8	-0.784843000	-4.194533000	-1.596331000
1	-0.273139000	-4.501926000	-2.406629000
8	-0.850053000	-6.416710000	2.536924000
1	-1.703019000	-6.719349000	2.127380000
1	-1.000759000	-6.156835000	3.478531000
8	-2.127824000	-2.843177000	7.512811000
1	-1.487104000	-3.204645000	8.214750000
1	-2.239822000	-1.855398000	7.465297000
8	-1.617028000	-6.151096000	5.231008000
1	-1.901854000	-7.076997000	5.337922000
1	-2.387464000	-5.484715000	5.506222000
8	0.406405000	-5.541734000	6.817715000
1	-0.348000000	-5.823945000	6.208686000
1	1.112704000	-5.130055000	6.290253000
8	-3.401072000	-4.527657000	5.980188000
1	-3.798074000	-3.904027000	5.273795000
1	-3.023742000	-3.938826000	6.715538000
1	3.736517000	1.137687000	6.881836000
1	3.351518000	4.589240000	3.485554000
1	-0.465750000	6.334044000	5.786894000
1	-1.838006000	7.199524000	1.231420000
1	-5.606762000	5.035808000	2.449291000
1	-4.644571000	6.228228000	-3.907036000
1	-1.601262000	7.411052000	-8.056729000
1	0.739995000	10.551953000	-5.055349000
1	6.989026000	4.588095000	-3.560643000
1	3.624775000	3.993891000	0.312677000
1	7.955703000	-1.404964000	-2.795618000
1	9.720076000	2.169238000	-0.029371000
1	6.220422000	0.867591000	3.241400000
1	9.069478000	-2.646042000	3.636081000
1	3.205646000	-0.763412000	-7.704880000
1	6.299437000	-3.137907000	-5.084188000
1	-6.876551000	-4.456558000	7.513569000
1	-7.125924000	-0.687607000	4.573346000
1	-4.469650000	-6.697305000	4.507362000
1	-9.133335000	-3.981820000	0.954448000
1	8.306884000	-4.708230000	-0.081879000
1	-7.211618000	4.430850000	-3.675528000
1	0.567622000	-0.544095000	-3.999136000
1	3.613206000	-6.660921000	-3.250775000
1	-0.507379000	-4.686666000	-0.775044000
1	4.629446000	-2.110694000	3.666655000

[1]TS3$_B$:

6	8.608011000	-2.757003000	2.480286000
6	7.830273000	-1.602297000	1.865285000
8	7.766888000	-1.496411000	0.605145000
6	7.770634000	-4.072611000	2.589945000
6	6.863393000	-4.342441000	1.430439000
7	7.269642000	-4.588648000	0.110316000

6	5.493956000	-4.329020000	1.385867000
6	6.187928000	-4.699203000	-0.701992000
7	5.105635000	-4.547107000	0.070606000
1	9.474033000	-2.924077000	1.832070000
1	8.464737000	-4.907223000	2.751482000
1	7.139605000	-4.019887000	3.483265000
1	4.775934000	-4.171996000	2.170380000
1	6.166844000	-4.850303000	-1.782717000
1	4.083306000	-4.432672000	-0.237648000
7	7.226913000	-0.724864000	2.705567000
6	6.299503000	0.317892000	2.250247000
6	6.776792000	1.174953000	1.107614000
8	5.947177000	1.627327000	0.267906000
1	7.162123000	-0.907093000	3.705805000
1	5.337860000	-0.101049000	1.940482000
7	8.089977000	1.480190000	0.982032000
6	8.539908000	2.296323000	-0.147148000
6	8.070065000	1.763264000	-1.507812000
8	7.816973000	2.566305000	-2.440678000
1	8.748759000	1.154908000	1.673546000
1	8.157785000	3.317551000	-0.071585000
7	7.974445000	0.412066000	-1.648311000
6	7.547129000	-0.181303000	-2.909988000
6	6.035642000	-0.219536000	-3.164469000
8	5.620632000	-0.699030000	-4.252411000
1	8.070845000	-0.201074000	-0.838462000
1	7.982597000	0.382939000	-3.739650000
7	5.226967000	0.274829000	-2.199798000
6	3.775234000	0.440114000	-2.391881000
6	3.437462000	1.937840000	-2.298396000
8	2.947606000	2.565773000	-3.279683000
6	2.935463000	-0.400943000	-1.406917000
6	3.211761000	-1.910250000	-1.486386000
6	2.324918000	-2.712646000	-0.540889000
8	1.299829000	-2.130972000	-0.053350000
8	2.630666000	-3.954519000	-0.270233000
1	5.634810000	0.638396000	-1.339777000
1	3.562942000	0.146230000	-3.421384000
1	1.876337000	-0.224054000	-1.625246000
1	3.091403000	-0.053803000	-0.379617000
1	4.263246000	-2.124935000	-1.260270000
1	3.035315000	-2.290503000	-2.503395000
7	3.724182000	2.544987000	-1.121226000
6	3.441046000	3.952949000	-0.886908000
6	4.282701000	4.940373000	-1.680246000
8	3.895897000	6.145027000	-1.752169000
1	4.141535000	2.025319000	-0.351810000
1	2.399140000	4.189538000	-1.132319000
7	5.369763000	4.499041000	-2.347187000
6	6.049496000	5.358249000	-3.314999000
6	5.126483000	5.867992000	-4.429854000
8	5.439024000	6.899355000	-5.067186000
1	5.738873000	3.567040000	-2.199031000
1	6.459916000	6.250910000	-2.835434000
7	3.982031000	5.157362000	-4.668438000
6	2.988292000	5.613522000	-5.638437000
6	1.687103000	6.080617000	-4.960947000
8	0.621742000	6.186332000	-5.630182000
6	2.703852000	4.548756000	-6.709665000
1	3.791813000	4.281738000	-4.183555000
1	3.429573000	6.498946000	-6.115975000
1	1.900823000	4.884448000	-7.369291000
1	3.608586000	4.376304000	-7.299655000
1	2.400296000	3.600988000	-6.253927000
7	1.734796000	6.439831000	-3.644711000
6	0.496331000	6.716742000	-2.907760000
6	-0.253852000	7.960527000	-3.397799000
8	-1.478157000	8.086438000	-3.121802000
6	0.721049000	6.794341000	-1.387788000
8	0.479776000	5.465920000	-0.821678000
1	2.598935000	6.340614000	-3.107352000
1	-0.208482000	5.899191000	-3.089432000
1	1.741421000	7.121472000	-1.164840000
1	0.004174000	7.508403000	-0.965227000
1	0.610762000	5.477445000	0.158830000

7	0.431703000	8.869925000	-4.129381000
6	-0.236467000	10.006798000	-4.756830000
6	-1.269726000	9.644064000	-5.835023000
8	-2.077194000	10.523538000	-6.211319000
1	1.408778000	8.691704000	-4.317041000
1	-0.780726000	10.587266000	-4.007138000
7	-1.248032000	8.369848000	-6.317591000
6	-2.269451000	7.888782000	-7.238475000
6	-3.190322000	6.820436000	-6.646547000
8	-3.852546000	6.074222000	-7.411855000
1	-0.537096000	7.700192000	-6.021853000
1	-2.873428000	8.750140000	-7.542899000
7	-3.253285000	6.745581000	-5.287631000
6	-4.088014000	5.759136000	-4.606318000
6	-3.274889000	4.743918000	-3.776916000
6	-2.252281000	3.914136000	-4.525429000
6	-2.400589000	3.576082000	-5.898016000
6	-1.111544000	3.434194000	-3.822011000
6	-1.467061000	2.780694000	-6.532680000
6	-0.162603000	2.644196000	-4.438401000
6	-0.321164000	2.267806000	-5.822763000
8	0.528508000	1.484901000	-6.414410000
1	-2.694235000	7.372642000	-4.715428000
1	-4.686432000	5.264971000	-5.375699000
1	-3.992580000	4.062528000	-3.288482000
1	-2.765349000	5.281466000	-2.966023000
1	-3.231188000	3.977459000	-6.468218000
1	-0.976343000	3.718928000	-2.781624000
1	-1.566317000	2.519830000	-7.580460000
1	0.735967000	2.322769000	-3.922800000
1	-3.828149000	-3.555180000	-2.813806000
6	-5.046005000	4.367532000	2.376430000
6	-3.596822000	4.722354000	2.157642000
8	-2.664180000	3.894075000	2.358035000
6	-5.547061000	3.244378000	1.407915000
6	-6.240834000	2.093326000	2.159836000
6	-5.207333000	1.235515000	2.897424000
8	-4.307235000	0.622700000	2.259326000
8	-5.344620000	1.271423000	4.209055000
1	-5.123939000	3.998423000	3.407277000
1	-6.246488000	3.670581000	0.681315000
1	-4.709412000	2.824850000	0.841715000
1	-6.753793000	1.424698000	1.463824000
1	-6.989890000	2.468500000	2.862401000
7	-3.287219000	5.996459000	1.760698000
6	-1.921298000	6.334882000	1.345494000
6	-0.862737000	6.024371000	2.402816000
8	0.270442000	5.585994000	2.057148000
1	-4.034187000	6.632289000	1.519419000
1	-1.627982000	5.784324000	0.445899000
7	-1.162588000	6.286115000	3.700272000
6	-0.204475000	6.026792000	4.779613000
6	-0.025756000	4.539216000	5.134020000
8	1.082463000	4.131999000	5.560944000
1	-2.077873000	6.654310000	3.919606000
1	0.780767000	6.401425000	4.496560000
7	-1.118374000	3.752746000	4.980195000
6	-1.113209000	2.289342000	5.199044000
6	-0.154766000	1.636503000	4.179872000
8	0.799526000	0.903758000	4.551689000
6	-2.570314000	1.810705000	5.061401000
6	-2.873355000	0.444236000	5.650924000
8	-2.125260000	-0.074101000	6.534155000
8	-3.967063000	-0.143633000	5.227673000
1	-1.902326000	4.117704000	4.453543000
1	-0.719728000	2.059698000	6.190321000
1	-2.875132000	1.801135000	4.014229000
1	-3.226849000	2.523504000	5.578336000
7	-0.359564000	1.976847000	2.880952000
6	0.516307000	1.488455000	1.808954000
6	1.959253000	1.982314000	2.012263000
8	2.942491000	1.277045000	1.655619000
6	-0.036749000	1.905156000	0.423205000
6	-1.408810000	1.296949000	0.022699000
6	-1.396992000	-0.211616000	-0.124894000

8	-0.920665000	-0.882592000	0.859644000
8	-1.862428000	-0.746158000	-1.214122000
1	-1.168717000	2.536808000	2.627229000
1	0.558050000	0.399012000	1.849976000
1	0.712389000	1.620175000	-0.323607000
1	-0.122385000	2.998706000	0.379851000
1	-2.176787000	1.541629000	0.768611000
1	-1.723488000	1.733740000	-0.929028000
7	2.122982000	3.205645000	2.585971000
6	3.431293000	3.739077000	2.951864000
6	4.221191000	2.869318000	3.930769000
8	5.467139000	2.749282000	3.782061000
1	1.315598000	3.796435000	2.738377000
1	4.086774000	3.844579000	2.083923000
7	3.544165000	2.265718000	4.935760000
6	4.228770000	1.326388000	5.824241000
6	4.461497000	-0.053110000	5.186067000
8	5.609740000	-0.589905000	5.193779000
1	2.553397000	2.445887000	5.066086000
1	5.211157000	1.718375000	6.091226000
7	3.379957000	-0.652805000	4.639859000
6	3.495763000	-1.964468000	3.989474000
6	2.379054000	-2.211606000	2.970682000
6	1.035502000	-2.452135000	3.586250000
7	-0.102481000	-2.710593000	2.813829000
6	0.655890000	-2.433772000	4.914858000
6	-1.133395000	-2.827538000	3.662501000
7	-0.708046000	-2.668758000	4.938560000
1	2.487437000	-0.157978000	4.586788000
1	3.505133000	-2.757156000	4.752350000
1	2.651163000	-3.078641000	2.353357000
1	2.328401000	-1.359164000	2.282332000
1	1.204224000	-2.212168000	5.813828000
1	-2.157161000	-3.001043000	3.385571000
6	0.052239000	-1.409826000	-4.558440000
6	1.049419000	-1.962278000	-5.554314000
8	1.199787000	-3.222837000	-5.708591000
6	-1.360678000	-1.204093000	-5.175628000
6	-2.033407000	-2.466115000	-5.735577000
6	-2.679465000	-3.428870000	-4.749812000
8	-3.028274000	-2.912546000	-3.536367000
8	-2.966401000	-4.589480000	-5.070747000
1	-0.004723000	-2.103840000	-3.714462000
1	-1.996720000	-0.765772000	-4.400030000
1	-1.272481000	-0.468517000	-5.982582000
1	-1.348661000	-3.052661000	-6.356805000
1	-2.850955000	-2.152575000	-6.402218000
7	1.755269000	-1.090424000	-6.303287000
6	2.583381000	-1.534812000	-7.434770000
6	3.609505000	-2.629190000	-7.117451000
8	3.798753000	-3.560864000	-7.930161000
1	1.518575000	-0.094915000	-6.240827000
1	1.958459000	-1.948023000	-8.231964000
7	4.300144000	-2.511164000	-5.944457000
6	5.272718000	-3.508703000	-5.518836000
6	4.773176000	-4.412701000	-4.389562000
8	5.586926000	-4.887403000	-3.539578000
1	4.290958000	-1.652553000	-5.402846000
1	5.515433000	-4.136458000	-6.384263000
7	3.457316000	-4.707905000	-4.387590000
6	2.857572000	-5.649075000	-3.430477000
6	1.430094000	-6.043945000	-3.835900000
8	0.554754000	-4.875550000	-3.785049000
6	0.848176000	-7.097847000	-2.898122000
1	2.848940000	-4.236516000	-5.055452000
1	2.820971000	-5.203357000	-2.428950000
1	1.432244000	-6.422706000	-4.867388000
1	0.656198000	-4.281287000	-4.585371000
1	-0.185163000	-7.313359000	-3.182073000
1	1.427593000	-8.026353000	-2.946327000
1	0.851618000	-6.739933000	-1.862124000
6	-5.425457000	-6.332453000	3.565511000
6	-6.575807000	-5.619594000	4.261015000
8	-7.698465000	-5.501183000	3.691708000
6	-5.904568000	-7.668452000	2.960362000
6	-4.838072000	-8.373183000	2.082856000
6	-4.333370000	-7.454805000	0.985185000
8	-3.228157000	-6.849362000	1.087104000
7	-5.145604000	-7.233737000	-0.069640000
1	-5.058363000	-5.673934000	2.767136000
1	-6.808514000	-7.469059000	2.374289000
1	-6.199949000	-8.352547000	3.766192000
1	-5.269238000	-9.285533000	1.651448000
1	-3.974971000	-8.666990000	2.687654000
1	-4.870442000	-6.498261000	-0.726357000
1	-6.028098000	-7.708522000	-0.172852000
7	-6.333242000	-5.127615000	5.503856000
6	-7.401857000	-4.533962000	6.306437000
6	-8.050508000	-3.264192000	5.744624000
8	-9.210208000	-2.955649000	6.105916000
1	-5.367659000	-5.075034000	5.837468000
1	-8.219770000	-5.248937000	6.431645000
7	-7.297486000	-2.524575000	4.886534000
6	-7.825049000	-1.334922000	4.236982000
6	-8.024584000	-1.487151000	2.727872000
8	-8.114604000	-0.457431000	2.004979000
1	-6.317976000	-2.757803000	4.697668000
1	-8.796454000	-1.112684000	4.692949000
7	-8.137740000	-2.755119000	2.255665000
6	-8.409008000	-3.056795000	0.852241000
6	-7.259285000	-3.767424000	0.144307000
8	-6.135068000	-2.860489000	-0.031035000
1	-8.077498000	-3.537659000	2.905096000
1	-8.625480000	-2.114205000	0.345092000
1	-6.946504000	-4.648945000	0.720346000
1	-7.605358000	-4.103031000	-0.842245000
1	-5.514399000	-3.157483000	-0.764230000
6	-8.189312000	4.021687000	-3.445382000
6	-7.665908000	2.756527000	-2.798077000
6	-8.283441000	2.222261000	-1.653121000
6	-6.572051000	2.064661000	-3.349224000
6	-7.832317000	1.026042000	-1.079449000
6	-6.114468000	0.870192000	-2.778767000
6	-6.743599000	0.345695000	-1.642579000
1	-8.919285000	3.793138000	-4.233981000
1	-8.692527000	4.667133000	-2.716851000
1	-9.132723000	2.742143000	-1.215834000
1	-6.087815000	2.456602000	-4.240961000
1	-8.314249000	0.620591000	-0.194299000
1	-5.271747000	0.346977000	-3.221904000
1	-6.403645000	-0.584029000	-1.196893000
26	-0.431989000	-2.793217000	0.798934000
26	-2.307550000	-2.681675000	-1.562147000
8	0.137607000	-4.714288000	0.594194000
1	1.117516000	-4.731573000	0.452405000
1	-0.261749000	-5.340222000	1.343058000
8	-4.507022000	-2.579596000	4.187199000
1	-4.326161000	-1.703807000	4.624667000
1	-4.476494000	-2.461880000	3.175609000
8	-4.372406000	-2.012415000	1.664865000
1	-5.159505000	-2.289058000	1.084640000
1	-4.282939000	-1.026072000	1.741291000
8	-2.319829000	-3.248557000	0.352158000
1	-3.255200000	-4.618117000	-0.304709000
1	-3.011523000	-2.752408000	0.878559000
8	-0.239634000	-3.823472000	8.359912000
1	0.066346000	-4.488679000	7.654518000
1	-0.041904000	-4.107231000	9.265229000
8	-4.253134000	-3.636946000	-1.682663000
8	-3.885416000	-4.957205000	-1.026376000
8	-0.835806000	-4.051031000	-1.772533000
1	-0.356398000	-4.394971000	-2.592661000
8	-0.966109000	-6.168230000	2.298407000
1	-1.790335000	-6.531159000	1.880101000
1	-1.138728000	-5.945504000	3.247435000
8	-2.216192000	-2.694204000	7.226471000
1	-1.492670000	-3.036218000	7.853561000
1	-2.325895000	-1.704842000	7.186885000
8	-1.735286000	-5.988672000	4.963613000
1	-2.001077000	-6.916710000	5.096238000

1	-2.504522000	-5.327451000	5.257832000
8	0.403725000	-5.343565000	6.378773000
1	-0.396914000	-5.641665000	5.842097000
1	1.035505000	-4.892945000	5.790842000
8	-3.517904000	-4.379358000	5.739730000
1	-3.907728000	-3.746990000	5.037470000
1	-3.130339000	-3.793387000	6.473902000
1	3.625537000	1.212491000	6.729673000
1	3.270580000	4.728142000	3.390266000
1	-0.534979000	6.569004000	5.670622000
1	-1.894844000	7.404336000	1.111085000
1	-5.683215000	5.255929000	2.303536000
1	-4.783126000	6.273155000	-3.930417000
1	-1.818147000	7.455765000	-8.135433000
1	0.523582000	10.656029000	-5.200018000
1	6.876295000	4.784347000	-3.740987000
1	3.577434000	4.154721000	0.176720000
1	7.920903000	-1.207187000	-2.966253000
1	9.633435000	2.334002000	-0.131225000
1	6.106969000	0.989040000	3.094790000
1	8.983832000	-2.503002000	3.476991000
1	3.098972000	-0.651271000	-7.824460000
1	6.177199000	-3.011473000	-5.168248000
1	-6.992761000	-4.310786000	7.297006000
1	-7.166324000	-0.477917000	4.400903000
1	-4.587341000	-6.491515000	4.251689000
1	-9.298675000	-3.697512000	0.791725000
1	8.223413000	-4.595776000	-0.215667000
1	-7.384335000	4.600600000	-3.912815000
1	0.401550000	-0.445863000	-4.174387000
1	3.492716000	-6.541244000	-3.379267000
1	-0.573440000	-4.539836000	-0.944357000
1	4.470324000	-1.998547000	3.491482000

$^{1}Pr_{B}$:

6	8.591944000	-2.679823000	2.211689000
6	7.810122000	-1.529230000	1.593723000
8	7.717799000	-1.448790000	0.332780000
6	7.745062000	-3.988992000	2.342016000
6	6.786663000	-4.223051000	1.216585000
7	7.138142000	-4.402759000	-0.128701000
6	5.416553000	-4.205984000	1.229027000
6	6.026652000	-4.464351000	-0.902275000
7	4.974951000	-4.352054000	-0.080091000
1	9.449023000	-2.858732000	1.554843000
1	8.434323000	-4.834610000	2.460146000
1	7.153614000	-3.940885000	3.262056000
1	4.731899000	-4.089395000	2.049364000
1	5.979565000	-4.547255000	-1.991636000
1	3.945862000	-4.224722000	-0.345278000
7	7.232957000	-0.635371000	2.433179000
6	6.289200000	0.398967000	1.991934000
6	6.714152000	1.232665000	0.811901000
8	5.840288000	1.697074000	0.023430000
1	7.194389000	-0.802777000	3.439105000
1	5.314009000	-0.025083000	1.735770000
7	8.023187000	1.504098000	0.602829000
6	8.431689000	2.280417000	-0.568393000
6	7.862351000	1.744738000	-1.889788000
8	7.591473000	2.545127000	-2.819473000
1	8.714227000	1.155306000	1.249816000
1	8.094534000	3.317245000	-0.490558000
7	7.696286000	0.397604000	-2.002956000
6	7.172907000	-0.188423000	-3.233143000
6	5.653444000	-0.111038000	-3.431685000
8	5.161313000	-0.523554000	-4.514434000
1	7.833751000	-0.210228000	-1.194793000
1	7.619513000	0.315434000	-4.094817000
7	4.915846000	0.389281000	-2.413248000
6	3.461996000	0.603237000	-2.525647000
6	3.167011000	2.100613000	-2.341005000
8	2.687086000	2.802528000	-3.275724000
6	2.654077000	-0.260168000	-1.535040000
6	2.871324000	-1.772552000	-1.701231000
6	2.079628000	-2.570614000	-0.676060000
8	1.036048000	-2.024021000	-0.181973000
8	2.473310000	-3.771530000	-0.346083000
1	5.381907000	0.713512000	-1.567240000
1	3.194506000	0.359701000	-3.555436000
1	1.589895000	-0.039333000	-1.672617000
1	2.892096000	0.027977000	-0.504197000
1	3.933329000	-2.029996000	-1.621555000
1	2.543718000	-2.111601000	-2.695331000
7	3.482837000	2.623086000	-1.130358000
6	3.252053000	4.021032000	-0.800261000
6	4.143434000	5.023225000	-1.517553000
8	3.818624000	6.247789000	-1.505935000
1	3.918506000	2.052368000	-0.409707000
1	2.222439000	4.315141000	-1.031890000
7	5.209982000	4.571527000	-2.211674000
6	5.942778000	5.451672000	-3.120377000
6	5.053090000	6.084317000	-4.197131000
8	5.414979000	7.142738000	-4.759120000
1	5.506097000	3.605968000	-2.138915000
1	6.400023000	6.286291000	-2.582266000
7	3.876765000	5.447686000	-4.485290000
6	2.903690000	6.024605000	-5.410595000
6	1.591422000	6.390717000	-4.695620000
8	0.512200000	6.486394000	-5.342682000
6	2.645076000	5.116102000	-6.621397000
1	3.646016000	4.548747000	-4.066120000
1	3.355159000	6.965077000	-5.756275000
1	1.869827000	5.546095000	-7.259030000
1	3.568551000	5.005707000	-7.196645000
1	2.311592000	4.124034000	-6.301769000
7	1.642468000	6.678042000	-3.361951000
6	0.404296000	6.880692000	-2.597802000
6	-0.439755000	8.056034000	-3.105879000
8	-1.683135000	8.056261000	-2.907545000
6	0.675573000	7.032485000	-1.087889000
8	0.500199000	5.722021000	-0.464059000
1	2.512580000	6.561114000	-2.837301000
1	-0.247637000	6.008633000	-2.718300000
1	1.689996000	7.405296000	-0.914839000
1	-0.050746000	7.738955000	-0.667167000
1	0.728079000	5.749136000	0.497184000
7	0.203450000	9.069608000	-3.736895000
6	-0.524069000	10.199413000	-4.309892000
6	-1.468606000	9.866346000	-5.475618000
8	-2.304861000	10.728452000	-5.829444000
1	1.199334000	8.983709000	-3.886439000
1	-1.151855000	10.667675000	-3.546808000
7	-1.339365000	8.642141000	-6.057673000
6	-2.270719000	8.181388000	-7.080113000
6	-3.101111000	6.966286000	-6.662558000
8	-3.670047000	6.268463000	-7.540129000
1	-0.617902000	7.985977000	-5.758568000
1	-2.943556000	9.013391000	-7.312969000
7	-3.189611000	6.716073000	-5.327611000
6	-3.924395000	5.571395000	-4.795640000
6	-3.011330000	4.499237000	-4.167903000
6	-2.035610000	3.788443000	-5.082085000
6	-2.306565000	3.546764000	-6.457473000
6	-0.825727000	3.281922000	-4.529706000
6	-1.445767000	2.784702000	-7.223855000
6	0.051050000	2.524528000	-5.277202000
6	-0.251522000	2.208174000	-6.653857000
8	0.505154000	1.418574000	-7.353145000
1	-2.714293000	7.322052000	-4.665225000
1	-4.522381000	5.163108000	-5.613613000
1	-3.664265000	3.735886000	-3.710758000
1	-2.454979000	4.954676000	-3.338135000
1	-3.176492000	3.996853000	-6.922364000
1	-0.587332000	3.501262000	-3.493139000
1	-1.647217000	2.586582000	-8.270721000
1	0.986023000	2.177716000	-4.849960000
1	-3.745851000	-4.553938000	-2.454322000
6	-4.836477000	4.437535000	2.745386000
6	-3.393144000	4.832923000	2.571421000
8	-2.451675000	4.000323000	2.700062000

6	-5.321200000	3.458956000	1.623033000
6	-6.054971000	2.230513000	2.199037000
6	-5.055929000	1.290224000	2.887842000
8	-4.287827000	0.557351000	2.211276000
8	-5.068356000	1.408007000	4.204653000
1	-4.907553000	3.919192000	3.709638000
1	-5.991888000	3.990804000	0.939647000
1	-4.471405000	3.106078000	1.029124000
1	-6.534850000	1.647240000	1.409871000
1	-6.829722000	2.539147000	2.906708000
7	-3.098619000	6.138191000	2.279150000
6	-1.744313000	6.517413000	1.859359000
6	-0.655843000	6.141193000	2.863408000
8	0.467792000	5.735115000	2.454959000
1	-3.855307000	6.780506000	2.090725000
1	-1.470535000	6.033466000	0.915906000
7	-0.919078000	6.313226000	4.184874000
6	0.072306000	5.989741000	5.216364000
6	0.239946000	4.483702000	5.491540000
8	1.363025000	4.031376000	5.822986000
1	-1.827173000	6.666301000	4.453706000
1	1.052207000	6.362710000	4.913969000
7	-0.879698000	3.729981000	5.374645000
6	-0.907800000	2.255816000	5.512320000
6	0.006662000	1.635139000	4.433378000
8	0.947791000	0.857383000	4.738641000
6	-2.387431000	1.842831000	5.387388000
6	-2.723736000	0.413147000	5.779732000
8	-2.053840000	-0.195948000	6.666834000
8	-3.774039000	-0.117089000	5.199688000
1	-1.689789000	4.147539000	4.935506000
1	-0.497816000	1.958478000	6.478750000
1	-2.749009000	2.017193000	4.374820000
1	-2.979601000	2.489340000	6.050773000
7	-0.229549000	2.048682000	3.161892000
6	0.579194000	1.580350000	2.031772000
6	2.033761000	2.062266000	2.149611000
8	2.986443000	1.360361000	1.710930000
6	-0.063591000	2.028083000	0.696826000
6	-1.471257000	1.439521000	0.414565000
6	-1.469070000	-0.058646000	0.210490000
8	-1.020882000	-0.781336000	1.182859000
8	-1.895944000	-0.528162000	-0.918544000
1	-1.026402000	2.648632000	2.965371000
1	0.618490000	0.489980000	2.053032000
1	0.618537000	1.744274000	-0.111712000
1	-0.138220000	3.123231000	0.675942000
1	-2.157357000	1.654649000	1.240820000
1	-1.876258000	1.905122000	-0.487357000
7	2.238259000	3.271984000	2.737932000
6	3.567704000	3.793369000	3.038504000
6	4.399386000	2.896839000	3.956211000
8	5.632879000	2.763150000	3.735581000
1	1.445007000	3.862028000	2.954344000
1	4.175142000	3.911119000	2.137465000
7	3.769221000	2.276746000	4.981950000
6	4.487985000	1.302053000	5.803441000
6	4.670150000	-0.057820000	5.108744000
8	5.817975000	-0.585971000	5.002744000
1	2.795494000	2.483023000	5.185422000
1	5.488215000	1.674008000	6.030010000
7	3.550081000	-0.653636000	4.641970000
6	3.625287000	-1.945457000	3.946178000
6	2.427014000	-2.184755000	3.023189000
6	1.148757000	-2.492962000	3.740358000
7	-0.037022000	-2.736704000	3.040809000
6	0.870490000	-2.569500000	5.090817000
6	-0.997231000	-2.938494000	3.956057000
7	-0.478947000	-2.851477000	5.202964000
1	2.651024000	-0.168634000	4.676185000
1	3.712982000	-2.758664000	4.681390000
1	2.667571000	-3.017858000	2.347867000
1	2.298629000	-1.305749000	2.377616000
1	1.492934000	-2.408343000	5.953081000
1	-2.038640000	-3.114472000	3.752194000
6	-0.066273000	-1.248585000	-5.341847000
6	0.972544000	-1.956811000	-6.174596000
8	1.192038000	-3.212332000	-6.046195000
6	-1.506812000	-1.386237000	-5.923049000
6	-2.184506000	-2.742391000	-5.668055000
6	-2.557142000	-3.001434000	-4.212209000
8	-2.157527000	-2.100582000	-3.342425000
8	-3.207852000	-4.047521000	-3.909075000
1	-0.060516000	-1.657863000	-4.329742000
1	-2.113659000	-0.599647000	-5.464248000
1	-1.478531000	-1.185934000	-7.001816000
1	-1.544447000	-3.572818000	-5.995574000
1	-3.105024000	-2.822557000	-6.259566000
7	1.644363000	-1.221296000	-7.085236000
6	2.495642000	-1.823815000	-8.117225000
6	3.631750000	-2.724831000	-7.626728000
8	3.972997000	-3.712764000	-8.315942000
1	1.396444000	-0.229710000	-7.174755000
1	1.901748000	-2.451520000	-8.788373000
7	4.249845000	-2.386177000	-6.458010000
6	5.313374000	-3.202955000	-5.892408000
6	4.872170000	-4.032381000	-4.687748000
8	5.715685000	-4.364315000	-3.799150000
1	4.050961000	-1.519127000	-5.971662000
1	5.662130000	-3.887699000	-6.673919000
7	3.582720000	-4.425138000	-4.660297000
6	3.057312000	-5.332022000	-3.632963000
6	1.598412000	-5.718326000	-3.906057000
8	0.750612000	-4.529812000	-3.814019000
6	1.090661000	-6.740320000	-2.892583000
1	2.941409000	-4.056655000	-5.361829000
1	3.100862000	-4.857268000	-2.645798000
1	1.508553000	-6.124001000	-4.923374000
1	0.765739000	-4.001779000	-4.667878000
1	0.031411000	-6.946362000	-3.068000000
1	1.647640000	-7.679958000	-2.975389000
1	1.204105000	-6.359366000	-1.871310000
6	-5.845172000	-6.290827000	3.914318000
6	-6.938601000	-5.386360000	4.461118000
8	-8.034165000	-5.232100000	3.850297000
6	-6.425731000	-7.523444000	3.198899000
6	-5.328390000	-8.502504000	2.703380000
6	-4.250672000	-7.775478000	1.922508000
8	-3.148013000	-7.477086000	2.489352000
7	-4.538201000	-7.399332000	0.667149000
1	-5.243678000	-5.701229000	3.208213000
1	-7.049595000	-7.187003000	2.363702000
1	-7.093389000	-8.066860000	3.878107000
1	-5.786671000	-9.286060000	2.087102000
1	-4.842392000	-8.987427000	3.554501000
1	-3.941209000	-6.735584000	0.142528000
1	-5.417192000	-7.661156000	0.246057000
7	-6.654688000	-4.758320000	5.632773000
6	-7.662618000	-3.967620000	6.334087000
6	-8.190214000	-2.737395000	5.592037000
8	-9.327489000	-2.287546000	5.866576000
1	-5.681300000	-4.750989000	5.955381000
1	-8.542483000	-4.580672000	6.548686000
7	-7.355888000	-2.180858000	4.672330000
6	-7.764678000	-1.043309000	3.867500000
6	-7.836025000	-1.339328000	2.370046000
8	-7.739429000	-0.392718000	1.541516000
1	-6.390157000	-2.500199000	4.553395000
1	-8.760446000	-0.740215000	4.211973000
7	-8.054598000	-2.632321000	2.017197000
6	-8.260415000	-3.050872000	0.630881000
6	-7.210594000	-4.034322000	0.121276000
8	-5.939542000	-3.360580000	-0.070672000
1	-8.137608000	-3.339308000	2.746407000
1	-8.250370000	-2.152893000	0.009417000
1	-7.097857000	-4.865250000	0.832649000
1	-7.548718000	-4.443594000	-0.839422000
1	-5.332598000	-3.854339000	-0.694326000
6	-7.303541000	2.209267000	-5.051151000
6	-6.645501000	1.600888000	-3.830389000

6	-7.392497000	1.317889000	-2.673827000
6	-5.277622000	1.270255000	-3.838497000
6	-6.797709000	0.716336000	-1.557215000
6	-4.671760000	0.674352000	-2.724503000
6	-5.433352000	0.398705000	-1.581057000
1	-7.624007000	1.433124000	-5.759965000
1	-8.193044000	2.788500000	-4.780540000
1	-8.451630000	1.563511000	-2.652602000
1	-4.687743000	1.468840000	-4.730963000
1	-7.388019000	0.478928000	-0.676654000
1	-3.619923000	0.407620000	-2.743972000
1	-4.968320000	-0.065029000	-0.718572000
26	-0.509571000	-2.665227000	1.048197000
26	-1.876203000	-2.435606000	-1.470181000
8	0.105204000	-4.560314000	0.690290000
1	1.080871000	-4.532368000	0.510718000
1	-0.243030000	-5.236196000	1.411795000
8	-4.551138000	-2.441696000	4.040892000
1	-4.272276000	-1.582830000	4.454559000
1	-4.487255000	-2.386239000	3.032432000
8	-4.388066000	-2.032004000	1.463444000
1	-5.133826000	-2.438640000	0.901416000
1	-4.394910000	-1.043220000	1.525258000
8	-2.302678000	-3.169094000	0.285534000
1	-2.812533000	-4.554056000	-0.195977000
1	-3.042561000	-2.682619000	0.776259000
8	-1.238742000	-4.608132000	8.741609000
1	-1.024717000	-5.336689000	8.058315000
1	-1.580147000	-4.978077000	9.570683000
8	-4.235954000	-4.764870000	-1.578794000
8	-3.196147000	-5.388227000	-0.678020000
8	-0.771827000	-4.010692000	-1.780664000
1	-0.227833000	-4.241157000	-2.597580000
8	-0.863550000	-6.094731000	2.392800000
1	-1.618838000	-6.673356000	2.116788000
1	-1.066392000	-5.815005000	3.318507000
8	-2.244648000	-2.869449000	7.219861000
1	-1.869601000	-3.400156000	8.006571000
1	-2.300460000	-1.879344000	7.265299000
8	-2.221801000	-6.118460000	4.795946000
1	-2.654255000	-6.811925000	4.246345000
1	-2.892033000	-5.394772000	5.100905000
8	-0.763031000	-6.360786000	6.924132000
1	-1.335756000	-6.363579000	6.083487000
1	0.148306000	-6.629833000	6.727802000
8	-3.804760000	-4.295080000	5.674793000
1	-4.111528000	-3.632193000	4.967441000
1	-3.298485000	-3.775873000	6.382463000
1	3.930989000	1.165942000	6.734913000
1	3.437392000	4.776259000	3.500196000
1	-0.217301000	6.493837000	6.143374000
1	-1.731166000	7.601415000	1.703520000
1	-5.485408000	5.318719000	2.797271000
1	-4.619847000	5.919863000	-4.022865000
1	-1.745059000	7.904419000	-7.998595000
1	0.202209000	10.942506000	-4.650577000
1	6.736348000	4.857682000	-3.580713000
1	3.382683000	4.141478000	0.276095000
1	7.456289000	-1.244461000	-3.271349000
1	9.524855000	2.277013000	-0.618873000
1	6.135672000	1.088034000	2.831159000
1	8.980913000	-2.417895000	3.200960000
1	2.919413000	-1.004568000	-8.706905000
1	6.137302000	-2.567099000	-5.571496000
1	-7.233576000	-3.646389000	7.288833000
1	-7.084134000	-0.201081000	4.012751000
1	-5.170677000	-6.593618000	4.721616000
1	-9.247261000	-3.525008000	0.543933000
1	8.076457000	-4.371034000	-0.495568000
1	-6.619463000	2.873888000	-5.591411000
1	0.181845000	-0.187501000	-5.274314000
1	3.682899000	-6.233023000	-3.601451000
1	-0.513933000	-4.483657000	-0.945313000
1	4.551347000	-1.948490000	3.362329000

^{11}TS4$_{B}$:

6	8.771829000	-1.068527000	4.018010000
6	8.250225000	0.032658000	3.104565000
8	8.383309000	-0.089377000	1.848970000
6	7.786010000	-2.277782000	4.136758000
6	7.054432000	-2.591569000	2.869587000
7	7.654823000	-2.927385000	1.648530000
6	5.717763000	-2.477299000	2.591711000
6	6.726831000	-2.975757000	0.664278000
7	5.543409000	-2.710363000	1.233182000
1	9.720171000	-1.406312000	3.587146000
1	8.345637000	-3.146862000	4.504349000
1	7.032342000	-2.045555000	4.895912000
1	4.894273000	-2.235427000	3.237389000
1	6.893749000	-3.138631000	-0.401465000
1	4.619152000	-2.530343000	0.747541000
7	7.660936000	1.102877000	3.687866000
6	6.938662000	2.142131000	2.946059000
6	7.656368000	2.772440000	1.781369000
8	6.972727000	3.292348000	0.852155000
1	7.393359000	1.070446000	4.675585000
1	5.989816000	1.764918000	2.548559000
7	9.004783000	2.778466000	1.714463000
6	9.685030000	3.265046000	0.511780000
6	9.187556000	2.623662000	-0.794586000
8	9.263739000	3.267445000	-1.864399000
1	9.538387000	2.365686000	2.464415000
1	9.546090000	4.341969000	0.390165000
7	8.697515000	1.348507000	-0.727704000
6	8.190105000	0.697228000	-1.929494000
6	6.765380000	1.083308000	-2.351343000
8	6.317820000	0.687203000	-3.461953000
1	8.644540000	0.855186000	0.164183000
1	8.837851000	0.938356000	-2.777009000
7	6.032450000	1.808804000	-1.471201000
6	4.650710000	2.210560000	-1.751068000
6	4.442548000	3.728778000	-1.696875000
8	3.458076000	4.235041000	-2.317087000
6	3.629101000	1.487163000	-0.830121000
6	3.769205000	-0.042452000	-0.927098000
6	2.822778000	-0.808532000	-0.023060000
8	1.685656000	-0.283493000	0.254391000
8	3.185119000	-1.976143000	0.424094000
1	6.455061000	2.079100000	-0.586070000
1	4.458473000	1.918942000	-2.787767000
1	2.620594000	1.785495000	-1.132387000
1	3.759755000	1.822661000	0.206230000
1	4.792547000	-0.355316000	-0.695726000
1	3.567517000	-0.383636000	-1.954194000
7	5.309619000	4.486184000	-0.989043000
6	5.103355000	5.922107000	-0.803669000
6	5.124956000	6.742085000	-2.093547000
8	4.453790000	7.798403000	-2.182861000
1	6.005698000	4.050230000	-0.386307000
1	4.142221000	6.121598000	-0.324022000
7	5.901243000	6.269876000	-3.107826000
6	5.935842000	6.942527000	-4.405874000
6	4.611901000	6.882683000	-5.185536000
8	4.380010000	7.735087000	-6.068933000
1	6.462323000	5.443073000	-2.952148000
1	6.162345000	8.004215000	-4.280722000
7	3.764298000	5.860494000	-4.870400000
6	2.394759000	5.796152000	-5.392509000
6	1.401770000	6.557991000	-4.491759000
8	0.480879000	7.239500000	-5.005196000
6	1.955871000	4.334154000	-5.581693000
1	4.001852000	5.224920000	-4.117602000
1	2.383370000	6.330404000	-6.344502000
1	0.915050000	4.282455000	-5.908773000
1	2.590580000	3.856925000	-6.337169000
1	2.040596000	3.778680000	-4.641302000
7	1.583287000	6.444817000	-3.148481000
6	0.694348000	7.042730000	-2.141201000
6	0.344572000	8.506430000	-2.435110000
8	-0.816082000	8.943576000	-2.232298000

6	1.414637000	6.923620000	-0.785342000
8	0.488388000	7.295904000	0.258951000
1	2.297617000	5.806811000	-2.807952000
1	-0.260664000	6.508413000	-2.099348000
1	1.742577000	5.883338000	-0.656294000
1	2.298755000	7.573100000	-0.786090000
1	0.911449000	7.361032000	1.148069000
7	1.359866000	9.295895000	-2.878381000
6	1.152353000	10.711237000	-3.177903000
6	0.156839000	11.031500000	-4.304574000
8	-0.255044000	12.211179000	-4.414731000
1	2.282202000	8.893359000	-3.001639000
1	0.773282000	11.236317000	-2.295670000
7	-0.213214000	10.011320000	-5.120881000
6	-1.233843000	10.169342000	-6.147194000
6	-2.512074000	9.361812000	-5.900637000
8	-3.291089000	9.128060000	-6.860058000
1	0.168620000	9.074205000	-4.999458000
1	-1.493672000	11.232191000	-6.193985000
7	-2.754969000	8.962051000	-4.624197000
6	-3.979817000	8.245370000	-4.260924000
6	-3.742222000	7.130626000	-3.231688000
6	-3.021971000	5.874560000	-3.706927000
6	-2.590417000	5.668761000	-5.027790000
6	-2.788073000	4.844472000	-2.775990000
6	-1.941325000	4.485043000	-5.399161000
6	-2.137175000	3.658945000	-3.130301000
6	-1.703295000	3.485018000	-4.451035000
8	-1.012280000	2.347054000	-4.864233000
1	-2.073338000	9.144472000	-3.892438000
1	-4.416729000	7.861156000	-5.187055000
1	-4.726061000	6.837330000	-2.834568000
1	-3.186517000	7.557221000	-2.383911000
1	-2.745949000	6.433392000	-5.779973000
1	-3.139546000	4.965119000	-1.753874000
1	-1.617207000	4.330606000	-6.422424000
1	-1.962226000	2.881494000	-2.393783000
1	-0.944563000	1.632111000	-4.185554000
6	-4.561749000	6.392678000	3.225008000
6	-3.107473000	6.762233000	3.063905000
8	-2.182944000	5.915628000	3.231817000
6	-5.032517000	5.333154000	2.173600000
6	-5.757056000	4.139496000	2.823845000
6	-4.755419000	3.242070000	3.559357000
8	-3.800991000	2.696629000	2.939417000
8	-4.980051000	3.175611000	4.859233000
1	-4.663914000	5.958890000	4.228264000
1	-5.704244000	5.807818000	1.450851000
1	-4.174540000	4.946453000	1.614806000
1	-6.233492000	3.513431000	2.064436000
1	-6.542272000	4.469897000	3.508792000
7	-2.787790000	8.056992000	2.763702000
6	-1.415959000	8.418611000	2.371602000
6	-0.367094000	7.989080000	3.395823000
8	0.747399000	7.520419000	3.026032000
1	-3.530579000	8.703827000	2.538677000
1	-1.144853000	7.964815000	1.412659000
7	-0.645855000	8.178800000	4.712215000
6	0.312844000	7.807682000	5.756771000
6	0.454312000	6.292087000	5.979625000
8	1.558704000	5.817133000	6.347982000
1	-1.539851000	8.575482000	4.966161000
1	1.306146000	8.178917000	5.497960000
7	-0.661928000	5.552543000	5.784683000
6	-0.686751000	4.077468000	5.882330000
6	0.252653000	3.495053000	4.807154000
8	1.162130000	2.677736000	5.108865000
6	-2.150928000	3.625568000	5.720336000
6	-2.498692000	2.268132000	6.310338000
8	-1.751547000	1.701112000	7.167911000
8	-3.628082000	1.740084000	5.910698000
1	-1.439465000	5.976404000	5.292563000
1	-0.289341000	3.760434000	6.848040000
1	-2.438547000	3.611192000	4.667716000
1	-2.804773000	4.352696000	6.220003000

7	0.081108000	3.963562000	3.545052000
6	0.943950000	3.519665000	2.443494000
6	2.401626000	3.958509000	2.646511000
8	3.354816000	3.218534000	2.280070000
6	0.402474000	4.021912000	1.086403000
6	-0.913487000	3.357014000	0.602021000
6	-0.725641000	1.890421000	0.301691000
8	-0.356333000	1.146780000	1.287512000
8	-0.904555000	1.438158000	-0.896550000
1	-0.700802000	4.581432000	3.337118000
1	0.964752000	2.429711000	2.432141000
1	1.181745000	3.840910000	0.335800000
1	0.239052000	5.105088000	1.123603000
1	-1.697862000	3.444179000	1.365141000
1	-1.252059000	3.868797000	-0.300927000
7	2.611900000	5.174654000	3.220146000
6	3.955453000	5.639665000	3.543658000
6	4.695463000	4.741142000	4.538840000
8	5.932522000	4.544300000	4.399900000
1	1.831520000	5.795395000	3.406931000
1	4.591510000	5.672108000	2.654791000
7	3.978246000	4.193519000	5.549928000
6	4.596528000	3.222703000	6.452249000
6	4.744708000	1.820104000	5.838598000
8	5.859905000	1.211976000	5.872713000
1	3.014040000	4.477606000	5.709736000
1	5.601507000	3.551383000	6.722325000
7	3.638548000	1.272628000	5.293353000
6	3.696623000	-0.036814000	4.634090000
6	2.592766000	-0.228031000	3.583146000
6	1.234901000	-0.517781000	4.148845000
7	0.122188000	-0.773514000	3.329971000
6	0.800408000	-0.539888000	5.455666000
6	-0.946469000	-0.918122000	4.135381000
7	-0.562074000	-0.792067000	5.421004000
1	2.765140000	1.805633000	5.237718000
1	3.653796000	-0.838311000	5.387311000
1	2.886405000	-1.053176000	2.919747000
1	2.558103000	0.668408000	2.950318000
1	1.307579000	-0.347361000	6.384564000
1	-1.961250000	-1.066193000	3.807524000
6	1.824280000	0.210288000	-5.475959000
6	2.913689000	-0.747239000	-5.881278000
8	2.967049000	-1.948919000	-5.461215000
6	0.501249000	-0.030330000	-6.271770000
6	-0.340744000	-1.229814000	-5.801621000
6	-1.077728000	-1.068827000	-4.475159000
8	-0.616129000	-0.185450000	-3.603427000
8	-2.070276000	-1.827349000	-4.245818000
1	1.606587000	0.096113000	-4.410845000
1	-0.090080000	0.885961000	-6.190302000
1	0.742697000	-0.166873000	-7.335013000
1	0.283804000	-2.130873000	-5.709862000
1	-1.099697000	-1.467731000	-6.554327000
7	3.838367000	-0.305585000	-6.775727000
6	4.768159000	-1.199798000	-7.474188000
6	5.770075000	-1.965163000	-6.608751000
8	6.227292000	-3.049313000	-7.035194000
1	3.767712000	0.649884000	-7.095572000
1	4.215818000	-1.966877000	-8.024845000
7	6.134911000	-1.418557000	-5.414699000
6	7.028712000	-2.120544000	-4.507264000
6	6.312330000	-2.747572000	-3.313276000
8	6.947310000	-2.956114000	-2.234374000
1	5.820492000	-0.504390000	-5.108215000
1	7.520037000	-2.920478000	-5.072618000
7	5.025944000	-3.103822000	-3.495709000
6	4.248190000	-3.830971000	-2.485220000
6	2.840733000	-4.171181000	-2.991284000
8	2.076574000	-2.937455000	-3.183354000
6	2.081545000	-5.046233000	-1.997577000
1	4.560161000	-2.824560000	-4.356133000
1	4.143031000	-3.226036000	-1.577984000
1	2.911806000	-4.683972000	-3.960806000
1	2.268942000	-2.513388000	-4.067580000

1	1.068088000	-5.234745000	-2.361800000
1	2.585860000	-6.009218000	-1.863659000
1	2.009723000	-4.553798000	-1.021679000
6	-4.967464000	-4.285895000	3.453350000
6	-6.169665000	-3.708376000	4.189554000
8	-7.264094000	-3.533711000	3.580969000
6	-5.400775000	-5.510979000	2.619530000
6	-4.268049000	-6.137196000	1.774418000
6	-3.582988000	-5.155212000	0.836061000
8	-2.351578000	-4.894179000	0.946543000
7	-4.338191000	-4.560061000	-0.111375000
1	-4.575629000	-3.503947000	2.787339000
1	-6.239794000	-5.206262000	1.984006000
1	-5.796909000	-6.285290000	3.289072000
1	-4.678390000	-6.971058000	1.188299000
1	-3.487404000	-6.549703000	2.420700000
1	-3.942599000	-3.791064000	-0.663680000
1	-5.320939000	-4.765574000	-0.204755000
7	-6.015350000	-3.410024000	5.505274000
6	-7.148450000	-2.960145000	6.317300000
6	-7.810649000	-1.643394000	5.888821000
8	-9.014295000	-1.440184000	6.172885000
1	-5.078930000	-3.393238000	5.912716000
1	-7.945642000	-3.708116000	6.296940000
7	-7.016176000	-0.756978000	5.233509000
6	-7.528918000	0.494001000	4.697179000
6	-7.619761000	0.529357000	3.169776000
8	-7.675839000	1.642828000	2.580701000
1	-6.006797000	-0.919191000	5.134468000
1	-8.533601000	0.649576000	5.105740000
7	-7.680090000	-0.667347000	2.532773000
6	-7.835301000	-0.787563000	1.085116000
6	-6.715887000	-1.579966000	0.417024000
8	-5.465991000	-0.841462000	0.470205000
1	-7.650617000	-1.527284000	3.077392000
1	-7.876940000	0.220584000	0.669264000
1	-6.605773000	-2.553717000	0.916055000
1	-6.990647000	-1.753077000	-0.632365000
1	-4.767877000	-1.225883000	-0.144109000
6	-8.484704000	5.955913000	-2.900432000
6	-7.791613000	4.795671000	-2.217210000
6	-8.282175000	4.272670000	-1.007808000
6	-6.657496000	4.194480000	-2.792007000
6	-7.668462000	3.174036000	-0.392137000
6	-6.039522000	3.095723000	-2.182631000
6	-6.542424000	2.580620000	-0.981226000
1	-9.240875000	5.603758000	-3.615886000
1	-8.999004000	6.598810000	-2.177210000
1	-9.156310000	4.727456000	-0.547291000
1	-6.255666000	4.586718000	-3.722567000
1	-8.051976000	2.778771000	0.543888000
1	-5.163170000	2.650446000	-2.643115000
1	-6.068051000	1.725261000	-0.508438000
26	-0.048279000	-0.853476000	1.282218000
26	-0.976692000	-0.478199000	-1.561652000
8	0.762179000	-2.768552000	1.149284000
1	1.748836000	-2.750692000	1.050309000
1	0.315098000	-3.455122000	1.829762000
8	-4.212385000	-0.689568000	4.884968000
1	-4.047659000	0.149469000	5.392736000
1	-4.189469000	-0.464288000	3.858581000
8	-4.077621000	0.003818000	2.509089000
1	-4.675019000	-0.292225000	1.755757000
1	-3.858612000	0.964207000	2.484823000
8	-1.519259000	-1.257087000	0.349817000
1	-2.563269000	-1.733969000	-0.083388000
1	-2.866900000	-1.634976000	-2.819101000
8	0.157785000	-2.073873000	8.669223000
1	0.406049000	-2.738136000	7.942694000
1	0.431643000	-2.359185000	9.554462000
8	-2.973890000	-1.095806000	-1.956542000
8	-3.465784000	-2.000567000	-0.834559000
8	0.188535000	-2.166470000	-1.585876000
1	0.885463000	-2.475543000	-2.245497000
8	-0.464907000	-4.301736000	2.654410000
1	-1.193198000	-4.669776000	2.080223000
1	-0.779711000	-4.127276000	3.579109000
8	-1.946232000	-0.953341000	7.739609000
1	-1.173577000	-1.304862000	8.294700000
1	-2.054303000	0.037870000	7.763012000
8	-1.472992000	-4.176789000	5.226455000
1	-1.735144000	-5.106073000	5.362049000
1	-2.234527000	-3.515088000	5.579760000
8	0.685037000	-3.585504000	6.640633000
1	-0.126975000	-3.860406000	6.107966000
1	1.325010000	-3.147304000	6.053250000
8	-3.219956000	-2.632669000	6.144492000
1	-3.631222000	-1.894638000	5.535708000
1	-2.867437000	-2.134233000	6.945253000
1	3.982370000	3.160344000	7.355482000
1	3.869916000	6.651598000	3.950996000
1	0.002063000	8.279590000	6.693743000
1	-1.375376000	9.506398000	2.250645000
1	-5.199989000	7.282643000	3.192470000
1	-4.707250000	8.955389000	-3.839980000
1	-0.853865000	9.868571000	-7.127781000
1	2.119904000	11.148449000	-3.439813000
1	6.730453000	6.492128000	-5.007771000
1	5.893799000	6.280556000	-0.136594000
1	8.202646000	-0.388468000	-1.783613000
1	10.755883000	3.070258000	0.623505000
1	6.678598000	2.946917000	3.648841000
1	8.980251000	-0.691123000	5.023735000
1	5.323300000	-0.599205000	-8.201506000
1	7.786495000	-1.438830000	-4.123578000
1	-6.798946000	-2.862212000	7.350036000
1	-6.897559000	1.333427000	5.001915000
1	-4.163227000	-4.547713000	4.149780000
1	-8.787345000	-1.289115000	0.861359000
1	8.648109000	-2.979142000	1.485491000
1	-7.775858000	6.575276000	-3.461197000
1	2.150828000	1.243370000	-5.640325000
1	4.781486000	-4.752921000	-2.221258000
1	0.220537000	-2.642815000	-0.722602000
1	4.677462000	-0.109891000	4.153731000
1	-1.167257000	-0.863766000	6.267900000

^{9}IM3$_{B}$:

6	8.911637000	-0.814532000	3.779005000
6	8.286697000	0.262623000	2.903753000
8	8.433486000	0.198654000	1.645994000
6	8.046782000	-2.116476000	3.861133000
6	7.308495000	-2.452403000	2.603593000
7	7.894035000	-2.756362000	1.366107000
6	5.959554000	-2.418944000	2.362577000
6	6.935203000	-2.871007000	0.412712000
7	5.757990000	-2.673259000	1.013486000
1	9.884020000	-1.047064000	3.333003000
1	8.696020000	-2.942569000	4.177219000
1	7.295608000	-1.993825000	4.647949000
1	5.140852000	-2.220417000	3.030141000
1	7.053529000	-3.047938000	-0.649992000
1	4.787442000	-2.547616000	0.552854000
7	7.595860000	1.254103000	3.515849000
6	6.786135000	2.240006000	2.791588000
6	7.466141000	2.985689000	1.672564000
8	6.763088000	3.443946000	0.725145000
1	7.334178000	1.184122000	4.502379000
1	5.893593000	1.779693000	2.353994000
7	8.807149000	3.153051000	1.676458000
6	9.486830000	3.755832000	0.528405000
6	9.147408000	3.094896000	-0.818189000
8	9.200400000	3.774866000	-1.867505000
1	9.344390000	2.794553000	2.451655000
1	9.221892000	4.810723000	0.422594000
7	8.818656000	1.768486000	-0.803212000
6	8.439123000	1.092377000	-2.037548000
6	6.974517000	1.253566000	-2.465662000
8	6.603665000	0.809708000	-3.584732000
1	8.760348000	1.252671000	0.075980000

1	9.045145000	1.476309000	-2.862510000
7	6.135737000	1.862478000	-1.588790000
6	4.726796000	2.108773000	-1.897754000
6	4.374406000	3.598612000	-1.926583000
8	3.307885000	3.964803000	-2.509784000
6	3.762622000	1.345166000	-0.952379000
6	3.997932000	-0.172886000	-1.008712000
6	3.000887000	-0.970757000	-0.180140000
8	1.836226000	-0.497949000	-0.007449000
8	3.377756000	-2.132619000	0.299820000
1	6.493181000	2.170931000	-0.688070000
1	4.578904000	1.747669000	-2.920846000
1	2.734379000	1.565391000	-1.253101000
1	3.868312000	1.717917000	0.073610000
1	5.013887000	-0.427133000	-0.691005000
1	3.903247000	-0.530832000	-2.045150000
7	5.206832000	4.490792000	-1.342201000
6	4.862975000	5.910842000	-1.260192000
6	4.700994000	6.601455000	-2.615059000
8	3.899798000	7.559511000	-2.744430000
1	5.964292000	4.166412000	-0.743976000
1	3.922018000	6.056426000	-0.723488000
7	5.463436000	6.130494000	-3.638040000
6	5.337806000	6.666961000	-4.993479000
6	3.992705000	6.368006000	-5.677947000
8	3.635643000	7.061723000	-6.653709000
1	6.126065000	5.389971000	-3.449799000
1	5.433566000	7.755653000	-4.982607000
7	3.270891000	5.328326000	-5.170625000
6	1.878014000	5.076969000	-5.560043000
6	0.903421000	5.864523000	-4.660764000
8	-0.052542000	6.503224000	-5.166837000
6	1.576601000	3.568570000	-5.544138000
1	3.613536000	4.828520000	-4.358200000
1	1.738941000	5.490259000	-6.560732000
1	0.525578000	3.375129000	-5.775935000
1	2.202710000	3.066086000	-6.290542000
1	1.797828000	3.141021000	-4.559668000
7	1.144824000	5.842582000	-3.322026000
6	0.290995000	6.529598000	-2.342226000
6	-0.007162000	7.986421000	-2.727891000
8	-1.153981000	8.474566000	-2.560758000
6	1.001521000	6.457570000	-0.978815000
8	0.065396000	6.876657000	0.038295000
1	1.907242000	5.271203000	-2.965690000
1	-0.683921000	6.037058000	-2.269502000
1	1.328494000	5.423155000	-0.804124000
1	1.885656000	7.107764000	-0.996006000
1	0.479448000	6.982287000	0.928199000
7	1.034935000	8.707189000	-3.221630000
6	0.876158000	10.106032000	-3.613183000
6	-0.084263000	10.386144000	-4.779846000
8	-0.436122000	11.571862000	-4.989348000
1	1.957370000	8.283885000	-3.280608000
1	0.495801000	10.698238000	-2.774651000
7	-0.498073000	9.324492000	-5.519492000
6	-1.504908000	9.448311000	-6.563714000
6	-2.836685000	8.765565000	-6.236149000
8	-3.634170000	8.476621000	-7.166332000
1	-0.169025000	8.382094000	-5.318244000
1	-1.687339000	10.516178000	-6.724108000
7	-3.099441000	8.536252000	-4.923063000
6	-4.357167000	7.939849000	-4.473761000
6	-4.153999000	6.773202000	-3.494991000
6	-3.510543000	5.506911000	-4.044680000
6	-3.328684000	5.250245000	-5.415492000
6	-3.076826000	4.526442000	-3.134071000
6	-2.735557000	4.061942000	-5.855627000
6	-2.475743000	3.336086000	-3.558287000
6	-2.305603000	3.105986000	-4.930557000
8	-1.687550000	1.949574000	-5.412306000
1	-2.389627000	8.727477000	-4.220532000
1	-4.904262000	7.635185000	-5.369998000
1	-5.139626000	6.521712000	-3.074036000
1	-3.545586000	7.134109000	-2.654197000

1	-3.625529000	5.986386000	-6.155091000
1	-3.205497000	4.702564000	-2.068094000
1	-2.588413000	3.874546000	-6.913324000
1	-2.113816000	2.615591000	-2.830808000
1	-1.506007000	1.281701000	-4.702289000
6	-5.148556000	6.685777000	3.278894000
6	-3.656531000	6.811251000	3.067578000
8	-2.844004000	5.883150000	3.338503000
6	-5.814153000	5.573969000	2.397408000
6	-6.353078000	4.389490000	3.217399000
6	-5.227934000	3.466616000	3.695748000
8	-4.336019000	3.066287000	2.894434000
8	-5.318046000	3.168403000	4.974345000
1	-5.305314000	6.441276000	4.337295000
1	-6.643047000	6.018081000	1.836623000
1	-5.100634000	5.186984000	1.662835000
1	-7.017056000	3.759106000	2.610186000
1	-6.938045000	4.734694000	4.075682000
7	-3.188614000	8.008695000	2.590253000
6	-1.806250000	8.153000000	2.116798000
6	-0.755504000	7.771404000	3.153295000
8	0.339157000	7.252263000	2.786762000
1	-3.857404000	8.707030000	2.297844000
1	-1.620494000	7.541587000	1.227663000
7	-0.998489000	8.047151000	4.457500000
6	-0.032098000	7.702411000	5.503609000
6	0.107786000	6.194679000	5.781675000
8	1.211474000	5.743249000	6.178765000
1	-1.885386000	8.463077000	4.705202000
1	0.961333000	8.058197000	5.224283000
7	-1.003208000	5.440663000	5.610417000
6	-1.023060000	3.971358000	5.798877000
6	-0.113899000	3.325737000	4.732462000
8	0.876348000	2.618729000	5.051921000
6	-2.498062000	3.538782000	5.707080000
6	-2.810712000	2.110593000	6.117237000
8	-2.069539000	1.467830000	6.922704000
8	-3.908144000	1.586501000	5.622114000
1	-1.790074000	5.830152000	5.098050000
1	-0.592029000	3.713413000	6.767213000
1	-2.873647000	3.684334000	4.693846000
1	-3.092090000	4.192499000	6.359465000
7	-0.397749000	3.644005000	3.440440000
6	0.423239000	3.154719000	2.322000000
6	1.879322000	3.638951000	2.442581000
8	2.842877000	2.912618000	2.084360000
6	-0.183590000	3.621416000	0.978995000
6	-1.542252000	2.990203000	0.597978000
6	-1.404253000	1.580658000	0.073875000
8	-0.527520000	0.809440000	0.566082000
8	-2.182625000	1.195325000	-0.906838000
1	-1.220408000	4.201213000	3.230027000
1	0.463984000	2.064141000	2.340463000
1	0.546018000	3.382449000	0.197127000
1	-0.298075000	4.711694000	0.988916000
1	-2.238471000	2.971570000	1.450014000
1	-2.021307000	3.598635000	-0.174532000
7	2.070056000	4.901458000	2.919023000
6	3.406643000	5.451883000	3.103251000
6	4.251853000	4.715978000	4.145367000
8	5.496430000	4.614133000	3.970725000
1	1.280531000	5.507583000	3.111946000
1	3.987901000	5.399133000	2.178073000
7	3.623883000	4.200898000	5.228704000
6	4.352997000	3.352309000	6.169305000
6	4.612402000	1.936244000	5.628287000
8	5.771683000	1.421341000	5.695693000
1	2.645903000	4.411602000	5.411414000
1	5.329688000	3.785539000	6.391501000
7	3.553007000	1.284568000	5.105310000
6	3.699578000	-0.050537000	4.509277000
6	2.670153000	-0.321151000	3.404754000
6	1.278874000	-0.551706000	3.908246000
7	0.198147000	-0.709209000	3.031590000
6	0.793694000	-0.602330000	5.200140000

6	-0.906211000	-0.830888000	3.787669000
7	-0.578390000	-0.776942000	5.097546000
1	2.643554000	1.747695000	5.041071000
1	3.634769000	-0.818959000	5.294213000
1	2.990887000	-1.200955000	2.829508000
1	2.671215000	0.522773000	2.702806000
1	1.272169000	-0.457361000	6.152796000
1	-1.906092000	-0.963019000	3.412308000
6	0.674674000	-0.392659000	-6.008174000
6	1.791298000	-1.393995000	-6.162049000
8	1.911017000	-2.399333000	-5.393603000
6	-0.717092000	-0.965250000	-6.422366000
6	-1.342828000	-1.973102000	-5.439969000
6	-1.769049000	-1.395843000	-4.108300000
8	-1.402093000	-0.213888000	-3.730125000
8	-2.480837000	-2.109694000	-3.285848000
1	0.621760000	-0.075717000	-4.962518000
1	-1.382401000	-0.104095000	-6.540022000
1	-0.632904000	-1.443528000	-7.406923000
1	-0.645853000	-2.794395000	-5.224093000
1	-2.224834000	-2.440289000	-5.895112000
7	2.673547000	-1.205574000	-7.183166000
6	3.628602000	-2.230947000	-7.623680000
6	4.736811000	-2.633607000	-6.645480000
8	5.324723000	-3.721404000	-6.824722000
1	2.540005000	-0.399909000	-7.778134000
1	3.100218000	-3.160240000	-7.857888000
7	5.032764000	-1.783100000	-5.620961000
6	6.005655000	-2.132557000	-4.589012000
6	5.410858000	-2.866918000	-3.384003000
8	6.129624000	-3.071954000	-2.362538000
1	4.580692000	-0.881716000	-5.567138000
1	6.760107000	-2.789474000	-5.032916000
7	4.132077000	-3.282552000	-3.483818000
6	3.459300000	-4.050244000	-2.428340000
6	1.966510000	-4.233262000	-2.724421000
8	1.296117000	-2.922030000	-2.807786000
6	1.280414000	-5.053701000	-1.639415000
1	3.608778000	-3.067993000	-4.327260000
1	3.574353000	-3.532227000	-1.473299000
1	1.842611000	-4.722285000	-3.701174000
1	1.401681000	-2.556955000	-3.722692000
1	0.220685000	-5.188830000	-1.867272000
1	1.746633000	-6.041520000	-1.553322000
1	1.350387000	-4.548238000	-0.672018000
6	-4.742636000	-3.863800000	1.986311000
6	-6.025726000	-3.498327000	2.717564000
8	-7.096742000	-3.280737000	2.077581000
6	-5.028180000	-5.006013000	0.983717000
6	-3.768647000	-5.596567000	0.304403000
6	-3.017286000	-4.662578000	-0.627911000
8	-1.805913000	-4.345788000	-0.382458000
7	-3.650547000	-4.229104000	-1.730677000
1	-4.364895000	-2.981000000	1.452701000
1	-5.748398000	-4.646583000	0.239300000
1	-5.527590000	-5.831300000	1.508099000
1	-4.066292000	-6.483951000	-0.272107000
1	-3.056561000	-5.931288000	1.064164000
1	-3.202432000	-3.577450000	-2.378802000
1	-4.620884000	-4.456311000	-1.885343000
7	-5.979579000	-3.462437000	4.076892000
6	-7.187821000	-3.281666000	4.880184000
6	-7.885171000	-1.921496000	4.777606000
8	-9.094447000	-1.826520000	5.096078000
1	-5.077770000	-3.438592000	4.557429000
1	-7.941850000	-4.018792000	4.590398000
7	-7.128063000	-0.880821000	4.339586000
6	-7.708103000	0.427713000	4.085592000
6	-8.005769000	0.703067000	2.609833000
8	-8.216166000	1.888268000	2.228809000
1	-6.115802000	-0.973552000	4.200042000
1	-8.653961000	0.496245000	4.634147000
7	-8.080554000	-0.376621000	1.794986000
6	-8.397995000	-0.285906000	0.374313000
6	-7.259523000	-0.779172000	-0.512493000
8	-6.129770000	0.133584000	-0.388396000
1	-7.869180000	-1.301929000	2.163250000
1	-8.622976000	0.758065000	0.147619000
1	-6.961604000	-1.790955000	-0.209632000
1	-7.603694000	-0.802037000	-1.555968000
1	-5.485842000	0.062711000	-1.136796000
6	-9.803902000	6.989910000	-2.251762000
6	-8.960634000	5.791085000	-1.871505000
6	-9.220242000	5.067524000	-0.694738000
6	-7.914203000	5.355572000	-2.704051000
6	-8.466032000	3.935798000	-0.357696000
6	-7.153454000	4.227753000	-2.372238000
6	-7.427010000	3.513353000	-1.198609000
1	-10.619136000	6.705417000	-2.931428000
1	-10.262863000	7.452163000	-1.370950000
1	-10.026016000	5.390047000	-0.039492000
1	-7.698928000	5.900323000	-3.620051000
1	-8.677232000	3.376654000	0.549091000
1	-6.345764000	3.915193000	-3.028368000
1	-6.851347000	2.630222000	-0.936260000
26	0.045471000	-1.177155000	0.954475000
26	-2.211234000	-0.739867000	-1.584698000
8	1.038500000	-2.926841000	1.325617000
1	1.978997000	-2.930700000	1.023440000
1	0.559334000	-3.757127000	1.699490000
8	-4.293958000	-0.572898000	3.968534000
1	-4.179821000	0.181715000	4.601902000
1	-4.409543000	-0.192983000	3.004147000
8	-4.709316000	0.613370000	1.813091000
1	-5.277396000	0.394599000	1.025405000
1	-4.607609000	1.574164000	1.994167000
8	-4.152945000	0.418336000	-2.229094000
1	-4.278099000	0.594768000	-3.179345000
1	-3.710810000	1.188877000	-1.778592000
8	-0.485234000	-2.655326000	8.147557000
1	-0.125215000	-3.161325000	7.343369000
1	-0.322938000	-3.108697000	8.988433000
8	-2.937473000	-1.625516000	0.169546000
8	-1.956601000	-1.698566000	1.171118000
8	-0.539640000	-2.046213000	-1.109101000
1	0.222146000	-2.266019000	-1.735668000
8	-0.536156000	-4.724556000	2.055202000
1	-1.100743000	-4.682034000	1.244736000
1	-1.039815000	-4.538345000	2.895666000
8	-2.353669000	-1.251472000	7.134295000
1	-1.693837000	-1.732604000	7.736467000
1	-2.450947000	-0.274655000	7.292951000
8	-1.789265000	-4.382495000	4.436449000
1	-2.144472000	-5.254182000	4.689942000
1	-2.479037000	-3.607390000	4.691851000
8	0.317826000	-3.750745000	5.946961000
1	-0.435985000	-4.050469000	5.352777000
1	0.885389000	-3.120569000	5.466124000
8	-3.409456000	-2.627078000	5.159182000
1	-3.733572000	-1.854281000	4.555042000
1	-3.140652000	-2.204945000	6.038933000
1	3.768264000	3.289849000	7.091707000
1	3.296689000	6.502331000	3.389707000
1	-0.331180000	8.207614000	6.426866000
1	-1.654431000	9.198627000	1.827981000
1	-5.634396000	7.652162000	3.101780000
1	-4.965743000	8.705972000	-3.972011000
1	-1.148404000	9.016879000	-7.503074000
1	1.862026000	10.496259000	-3.880500000
1	6.149952000	6.260409000	-5.602681000
1	5.655670000	6.408754000	-0.693184000
1	8.639370000	0.021147000	-1.938103000
1	10.565801000	3.691871000	0.698515000
1	6.428620000	2.989175000	3.510498000
1	9.090997000	-0.451793000	4.796005000
1	4.097102000	-1.874303000	-8.545726000
1	6.489361000	-1.219818000	-4.231842000
1	-6.923169000	-3.461874000	5.927023000
1	-7.041750000	1.213625000	4.448918000
1	-3.965103000	-4.167185000	2.696037000

1	-9.290919000	-0.888830000	0.160469000
1	8.884183000	-2.765466000	1.179233000
1	-9.209174000	7.754161000	-2.764842000
1	0.873977000	0.501673000	-6.608005000
1	3.930194000	-5.038692000	-2.338928000
1	-0.984397000	-2.904505000	-0.824300000
1	4.712503000	-0.105112000	4.098325000
1	-1.244200000	-0.899294000	5.881984000

Model C structures:

$^{11}Re_C$:

6	9.602795000	-3.367367000	1.443402000
6	9.163101000	-1.974935000	1.866772000
8	8.934114000	-1.083350000	1.007652000
6	8.424037000	-4.396892000	1.483077000
6	7.157723000	-3.904940000	0.853114000
7	6.802536000	-4.079922000	-0.494239000
6	6.153078000	-3.140978000	1.391395000
6	5.631556000	-3.444814000	-0.759352000
7	5.240073000	-2.865214000	0.382578000
1	9.992042000	-3.276986000	0.426436000
1	8.763240000	-5.322511000	1.003040000
1	8.204164000	-4.649561000	2.526148000
1	6.008142000	-2.800114000	2.401890000
1	5.070644000	-3.465772000	-1.698810000
1	4.410270000	-2.200030000	0.512145000
7	8.983093000	-1.750455000	3.203749000
6	8.202581000	-0.610624000	3.707034000
6	8.553866000	0.739134000	3.106207000
8	7.633857000	1.541480000	2.798257000
1	9.125231000	-2.509678000	3.862740000
1	7.136712000	-0.756119000	3.509517000
7	9.859386000	1.076690000	2.952935000
6	10.222707000	2.400958000	2.442679000
6	9.571186000	2.746007000	1.100023000
8	9.275314000	3.939405000	0.832145000
1	10.580923000	0.425834000	3.226302000
1	9.920569000	3.186132000	3.140827000
7	9.358988000	1.728531000	0.223115000
6	8.764998000	2.007544000	-1.081037000
6	7.345937000	2.573673000	-1.018895000
8	6.905863000	3.253338000	-1.988201000
1	9.454772000	0.757041000	0.513329000
1	9.366258000	2.738082000	-1.627982000
7	6.623250000	2.313105000	0.091924000
6	5.249190000	2.801825000	0.264724000
6	5.196755000	4.334977000	0.274889000
8	4.146795000	4.934660000	-0.083715000
6	4.646503000	2.224323000	1.564412000
6	4.569568000	0.687244000	1.582382000
6	3.291631000	0.081379000	1.029639000
8	2.230374000	0.783940000	0.953910000
8	3.302630000	-1.195716000	0.745558000
1	7.059763000	1.859054000	0.892066000
1	4.650707000	2.490509000	-0.597015000
1	3.647765000	2.641535000	1.715128000
1	5.267123000	2.558421000	2.400371000
1	4.623239000	0.343576000	2.626143000
1	5.421531000	0.224957000	1.071935000
7	6.302261000	5.002027000	0.697035000
6	6.336858000	6.462958000	0.680906000
6	6.289569000	7.055052000	-0.733929000
8	5.699038000	8.137627000	-0.954927000
1	7.152296000	4.500950000	0.939241000
1	5.475957000	6.873395000	1.212537000
7	6.936770000	6.343409000	-1.701166000
6	6.822912000	6.713083000	-3.110165000
6	5.478225000	6.304144000	-3.721201000
8	4.833381000	7.110024000	-4.446479000
1	7.317953000	5.431549000	-1.481596000
1	6.891445000	7.796955000	-3.210842000
7	5.009518000	5.069792000	-3.408654000
6	3.708826000	4.609748000	-3.917426000
6	2.594800000	5.603807000	-3.570560000
8	1.663465000	5.834434000	-4.395005000
6	3.382921000	3.209947000	-3.370331000
1	5.580746000	4.415307000	-2.877223000
1	3.745115000	4.576863000	-5.009769000
1	2.405011000	2.878549000	-3.724867000
1	4.115696000	2.476173000	-3.716800000
1	3.358573000	3.221838000	-2.275584000
7	2.643817000	6.172155000	-2.342151000
6	1.635121000	7.119545000	-1.856105000
6	1.406397000	8.286600000	-2.830934000
8	0.256686000	8.781440000	-2.978104000
6	2.083496000	7.607036000	-0.462249000
8	0.925835000	8.205094000	0.175754000
1	3.341434000	5.842998000	-1.675500000
1	0.661958000	6.629095000	-1.751694000
1	2.444791000	6.746378000	0.111410000
1	2.906053000	8.326711000	-0.554649000
1	0.972582000	8.202428000	1.158298000
7	2.502094000	8.742945000	-3.490659000
6	2.433217000	9.873624000	-4.407615000
6	1.709056000	9.633696000	-5.735610000
8	1.527754000	10.601655000	-6.511565000
1	3.380290000	8.231712000	-3.441871000
1	1.917987000	10.711903000	-3.928181000
7	1.273817000	8.369155000	-5.997205000
6	0.476422000	8.084066000	-7.178868000
6	-1.041677000	8.175320000	-6.990815000
8	-1.792826000	7.860390000	-7.947061000
1	1.484144000	7.596694000	-5.368565000
1	0.746307000	8.787666000	-7.971906000
7	-1.486790000	8.619492000	-5.783748000
6	-2.909587000	8.743498000	-5.487383000
6	-3.357739000	7.845377000	-4.302032000
6	-3.111781000	6.372117000	-4.552221000
6	-4.090545000	5.549657000	-5.137832000
6	-1.856360000	5.807036000	-4.269956000
6	-3.826773000	4.211036000	-5.439966000
6	-1.575691000	4.472825000	-4.575331000
6	-2.564089000	3.674623000	-5.164792000
8	-2.317595000	2.347430000	-5.524630000
1	-0.817020000	8.836475000	-5.052266000
1	-3.445097000	8.463189000	-6.398635000
1	-4.424769000	8.035665000	-4.124851000
1	-2.815741000	8.165409000	-3.401384000
1	-5.071237000	5.962305000	-5.361103000
1	-1.077122000	6.424522000	-3.835409000
1	-4.586633000	3.570882000	-5.875687000
1	-0.589404000	4.071958000	-4.369301000
1	-1.495878000	2.003883000	-5.079910000
6	-5.149881000	7.404685000	0.589948000
6	-3.711913000	7.606722000	0.999920000
8	-3.008961000	6.669595000	1.475083000
6	-5.351529000	6.175189000	-0.340459000
6	-6.533973000	5.277166000	0.104235000
6	-6.240025000	4.639905000	1.466606000
8	-5.739036000	3.441110000	1.476709000
8	-6.426374000	5.371051000	2.499539000
1	-5.712252000	7.216713000	1.517113000
1	-5.517370000	6.510310000	-1.370361000
1	-4.438619000	5.569424000	-0.350619000
1	-6.694971000	4.488959000	-0.635896000
1	-7.449834000	5.871882000	0.183099000
7	-3.196711000	8.872433000	0.910019000
6	-1.771187000	9.123969000	1.153178000
6	-1.338045000	8.737779000	2.563991000
8	-0.186208000	8.266850000	2.787962000
1	-3.734047000	9.581307000	0.432050000
1	-1.133745000	8.579517000	0.449276000
7	-2.224460000	8.961371000	3.568197000
6	-1.909195000	8.633867000	4.959605000
6	-1.825321000	7.126580000	5.228278000
8	-0.910307000	6.677055000	5.967818000
1	-3.130888000	9.340405000	3.331899000
1	-0.938348000	9.051218000	5.232881000
7	-2.763434000	6.343130000	4.653951000
6	-2.761203000	4.880197000	4.825202000
6	-1.526010000	4.271970000	4.159939000
8	-0.888791000	3.346630000	4.728961000
6	-4.057519000	4.283716000	4.264966000
6	-5.234477000	4.577156000	5.179756000
8	-5.101230000	4.767746000	6.413996000
8	-6.455715000	4.512153000	4.660914000
1	-3.437349000	6.741632000	4.014014000
1	-2.693347000	4.655862000	5.892341000

1	-3.961723000	3.192718000	4.186641000
1	-4.266875000	4.651369000	3.259977000
7	-1.153162000	4.780336000	2.958778000
6	0.060227000	4.289959000	2.291905000
6	1.306178000	4.608655000	3.142405000
8	2.230088000	3.761098000	3.278243000
6	0.155024000	4.841182000	0.853310000
6	-0.998146000	4.407891000	-0.073513000
6	-1.006528000	2.946315000	-0.450929000
8	-0.119960000	2.161314000	0.053804000
8	-1.891722000	2.511694000	-1.294511000
1	-1.719752000	5.493415000	2.503902000
1	0.028937000	3.200047000	2.258072000
1	1.108115000	4.504468000	0.432001000
1	0.173429000	5.936594000	0.882000000
1	-1.974700000	4.659117000	0.360042000
1	-0.952243000	4.972939000	-1.014397000
7	1.354742000	5.810106000	3.777801000
6	2.418030000	6.090639000	4.743106000
6	2.432131000	5.103784000	5.919782000
8	3.522250000	4.570039000	6.283914000
1	0.645964000	6.519677000	3.618472000
1	3.404184000	6.019737000	4.278232000
7	1.251338000	4.837597000	6.511150000
6	1.125314000	3.833533000	7.568778000
6	1.629904000	2.444560000	7.162277000
8	2.336365000	1.771584000	7.960604000
1	0.415399000	5.358372000	6.240236000
1	1.698434000	4.114554000	8.456444000
7	1.304093000	1.999662000	5.925449000
6	1.811345000	0.717172000	5.436538000
6	3.344271000	0.672425000	5.406272000
8	3.941576000	-0.450062000	5.463265000
6	1.319058000	0.434482000	4.000667000
6	-0.129625000	0.088310000	3.884186000
7	-0.813395000	0.235776000	2.664127000
6	-0.991405000	-0.467838000	4.800719000
6	-2.075700000	-0.199573000	2.877329000
7	-2.204780000	-0.651956000	4.145312000
1	0.618952000	2.509134000	5.361300000
1	1.505247000	-0.086597000	6.115172000
1	1.920559000	-0.402591000	3.622402000
1	1.549503000	1.301350000	3.373392000
1	-0.866430000	-0.739613000	5.833975000
1	-2.888942000	-0.173052000	2.176302000
1	-3.063617000	-1.082591000	4.610602000
7	3.994284000	1.837510000	5.233136000
6	5.448495000	1.949750000	5.284634000
6	6.008852000	1.936641000	6.711599000
8	6.980002000	1.181857000	7.006763000
1	3.456786000	2.691140000	5.118706000
1	5.906347000	1.119607000	4.752384000
7	5.414646000	2.774106000	7.590471000
6	5.826452000	2.858240000	8.991529000
6	5.631007000	1.570593000	9.808329000
8	6.348546000	1.355628000	10.810701000
1	4.650957000	3.370254000	7.268505000
1	6.890763000	3.098272000	9.069384000
7	4.683070000	0.707705000	9.348563000
6	4.455066000	-0.611158000	9.948179000
6	5.668612000	-1.549816000	9.817263000
8	5.725857000	-2.597843000	10.503199000
6	3.189028000	-1.215037000	9.271038000
6	2.659451000	-2.438746000	9.941687000
7	2.109841000	-2.382299000	11.230047000
6	2.629022000	-3.739636000	9.479054000
6	1.774144000	-3.628386000	11.539952000
7	2.064940000	-4.491784000	10.504474000
1	3.997729000	1.041564000	8.673710000
1	4.270925000	-0.517933000	11.023729000
1	2.421203000	-0.432831000	9.285246000
1	3.414412000	-1.426648000	8.218936000
1	2.973523000	-4.172384000	8.555022000
1	1.325256000	-3.950190000	12.464747000
1	1.922014000	-5.488393000	10.497927000
7	6.629125000	-1.206181000	8.908222000
6	7.895266000	-1.927470000	8.827704000
6	8.461963000	-1.934340000	7.386147000
6	7.761604000	-2.902950000	6.452483000
6	6.433468000	-2.683822000	6.027918000
6	8.409285000	-4.083452000	6.036181000
6	5.764969000	-3.638708000	5.251363000
6	7.746067000	-5.033805000	5.244594000
6	6.416879000	-4.817271000	4.858396000
1	6.586468000	-0.303016000	8.444798000
1	7.725314000	-2.941814000	9.198130000
1	9.526813000	-2.194543000	7.443760000
1	8.399117000	-0.904664000	7.009094000
1	5.913946000	-1.777831000	6.317664000
1	9.423089000	-4.280924000	6.377001000
1	4.722318000	-3.474456000	4.997229000
1	8.252795000	-5.954621000	4.968330000
1	5.888529000	-5.567192000	4.276952000
6	-4.468878000	2.360918000	-9.762078000
6	-3.354567000	1.380534000	-10.023255000
8	-3.099136000	0.421647000	-9.247551000
1	-4.272995000	2.882054000	-8.818667000
7	-2.575557000	1.586803000	-11.134462000
6	-1.522169000	0.633246000	-11.494051000
6	-0.481742000	0.435460000	-10.388649000
8	0.078639000	-0.685643000	-10.237600000
1	-2.822117000	2.314341000	-11.789545000
1	-1.936393000	-0.356435000	-11.707471000
7	-0.230160000	1.507817000	-9.604736000
6	0.694958000	1.490673000	-8.462106000
6	0.399812000	0.315722000	-7.520933000
8	1.355891000	-0.254291000	-6.920763000
6	0.585429000	2.848941000	-7.724269000
6	1.732790000	3.122595000	-6.749432000
16	3.414131000	3.373506000	-7.610647000
6	3.266954000	5.206371000	-8.057922000
1	-0.731295000	2.366628000	-9.789032000
1	1.721291000	1.349308000	-8.815790000
1	-0.365727000	2.882695000	-7.175826000
1	0.556003000	3.643906000	-8.482990000
1	1.532248000	4.022529000	-6.161263000
1	1.875678000	2.295953000	-6.050834000
1	4.168832000	5.457099000	-8.619452000
1	2.389144000	5.382269000	-8.682346000
1	3.221589000	5.807708000	-7.148146000
7	-0.886902000	-0.058164000	-7.350948000
6	-1.267455000	-1.115667000	-6.406989000
6	-0.873777000	-2.503766000	-6.920551000
8	-0.676631000	-3.469106000	-6.127115000
6	-2.790324000	-1.006169000	-6.118549000
6	-3.227105000	-1.782952000	-4.847535000
6	-2.597657000	-1.134417000	-3.645572000
8	-3.164079000	-0.086963000	-3.101244000
8	-1.444813000	-1.506805000	-3.204264000
1	-1.627013000	0.344173000	-7.921133000
1	-0.705257000	-0.967173000	-5.481397000
1	-3.022658000	0.056415000	-5.979953000
1	-3.359292000	-1.358933000	-6.984872000
1	-2.921029000	-2.830073000	-4.906936000
1	-4.315825000	-1.727618000	-4.760104000
7	-0.751644000	-2.664037000	-8.256394000
6	-0.236619000	-3.908784000	-8.824864000
6	1.256563000	-4.101053000	-8.527946000
8	1.701348000	-5.187372000	-8.090059000
1	-0.813306000	-1.856843000	-8.859890000
1	-0.762340000	-4.766769000	-8.405098000
7	2.051555000	-3.019455000	-8.775868000
6	3.493062000	-3.091777000	-8.542213000
6	3.917062000	-3.119254000	-7.067731000
8	4.978440000	-3.688017000	-6.728937000
1	1.641983000	-2.170667000	-9.152262000
1	3.901004000	-4.001567000	-8.988887000
7	3.105428000	-2.466286000	-6.183637000
6	3.488374000	-2.339575000	-4.775330000
6	3.140151000	-3.605305000	-3.981957000

8	3.769551000	-3.912051000	-2.923842000
6	2.848676000	-1.044743000	-4.183340000
8	3.332436000	0.086101000	-4.955807000
6	3.215878000	-0.780830000	-2.729526000
1	2.338383000	-1.895804000	-6.531440000
1	4.575997000	-2.235332000	-4.718168000
1	1.756730000	-1.117366000	-4.272175000
1	2.780475000	0.188925000	-5.762766000
1	2.712440000	0.135470000	-2.412109000
1	2.905124000	-1.598469000	-2.075724000
1	4.299426000	-0.640943000	-2.636286000
7	2.117002000	-4.350782000	-4.444426000
6	1.699156000	-5.603155000	-3.810087000
6	0.433533000	-6.176279000	-4.461935000
8	-0.732496000	-5.338078000	-4.165771000
6	0.120584000	-7.576210000	-3.943613000
1	1.691270000	-4.084744000	-5.325210000
1	1.514792000	-5.424106000	-2.744872000
1	0.568975000	-6.201327000	-5.552631000
1	-0.808701000	-7.937596000	-4.391644000
1	-0.005999000	-7.564277000	-2.854045000
1	0.925302000	-8.276497000	-4.191508000
1	-0.788280000	-4.585873000	-4.806601000
6	-3.669152000	-4.573016000	1.998815000
6	-4.845580000	-4.946472000	2.882519000
8	-6.033082000	-4.898626000	2.468349000
6	-3.632042000	-5.429705000	0.706322000
6	-2.268202000	-5.441436000	-0.038802000
6	-1.851438000	-4.105641000	-0.618019000
8	-1.827976000	-3.065492000	0.152845000
7	-1.510001000	-4.014087000	-1.894624000
1	-3.769941000	-3.513498000	1.741033000
1	-4.432321000	-5.102202000	0.034536000
1	-3.855218000	-6.472935000	0.963866000
1	-2.303023000	-6.189986000	-0.837598000
1	-1.484673000	-5.758589000	0.664974000
1	-1.276989000	-3.098569000	-2.293880000
1	-1.399388000	-4.792733000	-2.558333000
7	-4.538411000	-5.356005000	4.149491000
6	-5.548076000	-5.884033000	5.065865000
6	-6.629158000	-4.902318000	5.523246000
8	-7.743199000	-5.361828000	5.870444000
1	-3.581028000	-5.303841000	4.462669000
1	-6.088869000	-6.707903000	4.592057000
7	-6.306463000	-3.583017000	5.550458000
6	-7.307741000	-2.579589000	5.878107000
6	-7.672465000	-1.680725000	4.698933000
8	-8.089162000	-0.507305000	4.913077000
1	-5.358302000	-3.215609000	5.392771000
1	-8.207369000	-3.101780000	6.221413000
7	-7.541419000	-2.212131000	3.462180000
6	-7.858904000	-1.459135000	2.249077000
6	-6.823658000	-1.647941000	1.147552000
8	-5.570122000	-0.985465000	1.502719000
1	-7.175378000	-3.154228000	3.356367000
1	-7.936437000	-0.405370000	2.527117000
1	-6.636457000	-2.718077000	0.988731000
1	-7.222697000	-1.215259000	0.221220000
1	-5.049865000	-0.748431000	0.682374000
6	-11.867127000	0.324299000	-0.650605000
6	-10.434946000	0.693288000	-0.973887000
6	-9.588567000	1.225514000	0.015716000
6	-9.924151000	0.535647000	-2.273836000
6	-8.272154000	1.593742000	-0.286261000
6	-8.608908000	0.904518000	-2.581026000
6	-7.776306000	1.436032000	-1.588696000
1	-12.257381000	-0.420940000	-1.352183000
1	-11.957027000	-0.084050000	0.362017000
1	-9.963635000	1.353887000	1.027551000
1	-10.563325000	0.120316000	-3.049043000
1	-7.630300000	2.004503000	0.487466000
1	-8.235225000	0.775254000	-3.593034000
1	-6.753578000	1.724666000	-1.809661000
26	0.151614000	0.338778000	0.796033000
26	-1.460077000	0.635156000	-2.115435000

8	0.785420000	-1.603804000	0.741238000
1	1.793979000	-1.661067000	0.664889000
1	0.389982000	-2.213454000	1.413737000
8	-5.522976000	0.381548000	5.473644000
1	-6.497738000	0.331662000	5.585897000
1	-5.333699000	0.812983000	4.577587000
8	-4.557214000	2.388658000	-0.524264000
1	-3.758451000	2.817599000	-0.897189000
1	-5.034654000	2.869250000	0.241392000
8	-5.137671000	1.230307000	3.031470000
1	-5.398108000	0.440662000	2.486403000
1	-5.435543000	2.083643000	2.629229000
8	-1.677757000	-0.444695000	-0.317184000
1	-1.573696000	-1.445963000	-0.283392000
1	-2.707537000	-0.235409000	-0.203911000
8	0.355392000	-2.355026000	7.370141000
1	1.045670000	-2.496177000	6.636796000
1	0.780791000	-2.487864000	8.238369000
8	0.211776000	1.129806000	-3.064152000
8	0.031317000	1.459664000	-4.394802000
8	-4.143803000	-0.013734000	-0.520097000
1	-4.390261000	1.048623000	-0.480710000
1	-4.218288000	-0.294935000	-1.460153000
8	-0.679970000	-3.105259000	2.477565000
1	-1.272380000	-3.182305000	1.658111000
1	-1.086670000	-2.538824000	3.162099000
8	-2.227700000	-1.986846000	7.490210000
1	-1.248691000	-2.230488000	7.388244000
1	-2.339562000	-1.405245000	8.262063000
8	1.616075000	-4.044161000	3.421423000
1	0.726777000	-3.807527000	3.035909000
1	1.743662000	-5.006421000	3.457441000
8	2.226144000	-2.658267000	5.604217000
1	2.065898000	-3.160116000	4.755951000
1	2.893334000	-1.932635000	5.529559000
8	-4.111269000	-1.742682000	5.599817000
1	-4.715150000	-0.905249000	5.681129000
1	-3.506814000	-1.889600000	6.387059000
1	8.636214000	-1.454512000	9.489457000
1	5.259722000	3.670509000	9.456144000
1	5.731287000	2.881209000	4.787461000
1	0.067181000	3.776203000	7.839522000
1	2.269465000	7.109143000	5.113165000
1	-2.675388000	9.079467000	5.600892000
1	-1.583163000	10.191995000	1.000477000
1	-5.556661000	8.312874000	0.130923000
1	-3.147753000	9.790970000	-5.257181000
1	0.695144000	7.070340000	-7.525703000
1	3.453539000	10.192016000	-4.633556000
1	7.643582000	6.247570000	-3.663411000
1	7.249858000	6.788478000	1.187859000
1	8.748928000	1.077914000	-1.657411000
1	11.310620000	2.438751000	2.331405000
1	8.323271000	-0.556684000	4.792329000
1	10.409616000	-3.739471000	2.086203000
1	-5.407351000	1.812139000	-9.639688000
1	-4.589773000	3.103320000	-10.555114000
1	-1.023061000	0.998327000	-12.397255000
1	-0.397501000	-3.876196000	-9.905927000
1	3.961735000	-2.227431000	-9.023395000
1	-5.030592000	-6.278018000	5.946269000
1	-6.960750000	-1.929405000	6.685295000
1	-2.729340000	-4.692686000	2.549707000
1	2.514096000	-6.335961000	-3.883988000
1	-8.838393000	-1.771058000	1.858640000
1	7.321228000	-4.611121000	-1.177297000
1	-12.526560000	1.201524000	-0.704881000

Model BP structures:

[11]Re_{BP}:

6	8.631143000	-1.413582000	3.551207000
6	8.050633000	-0.327747000	2.655564000
8	8.054085000	-0.487425000	1.398324000
6	7.675396000	-2.635349000	3.753330000
6	6.873621000	-3.000187000	2.543926000
7	7.397124000	-3.416003000	1.312395000
6	5.524491000	-2.884001000	2.337856000
6	6.411650000	-3.512996000	0.388910000
7	5.266200000	-3.196285000	1.008550000
1	9.555084000	-1.748766000	3.067620000
1	8.272703000	-3.484045000	4.108717000
1	6.964552000	-2.398882000	4.551522000
1	4.743905000	-2.592724000	3.015676000
1	6.514783000	-3.754740000	-0.670230000
1	4.316544000	-3.024091000	0.568763000
7	7.566450000	0.790815000	3.250832000
6	6.862530000	1.844347000	2.510987000
6	7.604319000	2.427602000	1.332911000
8	6.958495000	2.982304000	0.394291000
1	7.380905000	0.807262000	4.257285000
1	5.902375000	1.487558000	2.121235000
7	8.949182000	2.342509000	1.266966000
6	9.673607000	2.780149000	0.071462000
6	9.198271000	2.129468000	-1.237572000
8	9.404144000	2.717920000	-2.320070000
1	9.450587000	1.917037000	2.032508000
1	9.568570000	3.857846000	-0.074861000
7	8.577887000	0.911121000	-1.155136000
6	8.045393000	0.276775000	-2.352895000
6	6.587189000	0.611834000	-2.689643000
8	6.055792000	0.112017000	-3.720154000
1	8.449982000	0.446303000	-0.255820000
1	8.650811000	0.579335000	-3.212332000
7	5.918161000	1.420757000	-1.835209000
6	4.501684000	1.744696000	-2.036685000
6	4.189700000	3.243574000	-1.981877000
8	3.265163000	3.708617000	-2.710615000
6	3.565338000	1.035372000	-1.020144000
6	3.698063000	-0.493954000	-1.013118000
6	2.689431000	-1.157930000	-0.089253000
8	1.666535000	-0.482424000	0.275981000
8	2.890166000	-2.392765000	0.288631000
1	6.415394000	1.801210000	-1.032093000
1	4.259171000	1.411376000	-3.047423000
1	2.534561000	1.301297000	-1.275328000
1	3.738550000	1.438007000	-0.015177000
1	4.707669000	-0.804746000	-0.719543000
1	3.526587000	-0.914654000	-2.014946000
7	4.852031000	4.003895000	-1.076219000
6	4.350437000	5.306254000	-0.642485000
6	4.179570000	6.353420000	-1.725396000
8	3.280488000	7.229295000	-1.608210000
1	5.547707000	3.583057000	-0.457016000
1	3.368886000	5.207707000	-0.169635000
7	5.002686000	6.322719000	-2.801979000
6	4.804228000	7.273914000	-3.897096000
6	3.391399000	7.214674000	-4.490089000
8	2.870244000	8.246599000	-4.985497000
1	5.715712000	5.609179000	-2.862108000
1	4.953065000	8.300853000	-3.555068000
7	2.759755000	6.010587000	-4.461959000
6	1.414378000	5.865239000	-5.018383000
6	0.343618000	6.600559000	-4.203849000
8	-0.794390000	6.791939000	-4.718478000
6	1.051997000	4.383501000	-5.192858000
1	3.141017000	5.222784000	-3.937096000
1	1.396051000	6.363790000	-5.994031000
1	0.026264000	4.303432000	-5.560379000
1	1.725044000	3.918556000	-5.920844000
1	1.139839000	3.846792000	-4.243096000
7	0.670828000	7.072292000	-2.978504000
6	-0.264772000	7.911875000	-2.234256000

6	-0.708631000	9.141614000	-3.046224000
8	-1.848918000	9.635496000	-2.840922000
6	0.343080000	8.354259000	-0.899208000
8	0.261983000	7.223069000	0.018881000
1	1.588408000	6.907932000	-2.570725000
1	-1.185277000	7.358149000	-2.025018000
1	1.382747000	8.667200000	-1.036126000
1	-0.245634000	9.198137000	-0.516835000
1	0.797239000	7.364534000	0.833352000
7	0.177848000	9.659076000	-3.934740000
6	-0.159298000	10.829882000	-4.741851000
6	-1.238691000	10.617450000	-5.809819000
8	-1.729988000	11.614760000	-6.383209000
1	1.064646000	9.192729000	-4.115023000
1	-0.526329000	11.635830000	-4.100379000
7	-1.607186000	9.331346000	-6.077893000
6	-2.685282000	9.026771000	-7.005977000
6	-3.860959000	8.299496000	-6.361048000
8	-4.647773000	7.621517000	-7.073060000
1	-1.165388000	8.546511000	-5.604837000
1	-3.034119000	9.976679000	-7.426575000
7	-4.003568000	8.429114000	-5.012896000
6	-5.117682000	7.818993000	-4.289468000
6	-4.686547000	6.751000000	-3.268946000
6	-4.053025000	5.479291000	-3.786470000
6	-4.007994000	5.127158000	-5.164046000
6	-3.504916000	4.572644000	-2.829065000
6	-3.410720000	3.949667000	-5.566509000
6	-2.916199000	3.388110000	-3.205902000
6	-2.831911000	3.047689000	-4.604455000
8	-2.241441000	1.957008000	-5.003623000
1	-3.320921000	8.965285000	-4.484681000
1	-5.797469000	7.405968000	-5.039046000
1	-5.577529000	6.465341000	-2.685234000
1	-3.993450000	7.213557000	-2.549785000
1	-4.426360000	5.793946000	-5.911634000
1	-3.566359000	4.828022000	-1.775139000
1	-3.356103000	3.674367000	-6.613636000
1	-2.499305000	2.693739000	-2.482785000
1	-1.696623000	0.792285000	-4.212976000
6	-4.595402000	6.385945000	3.023831000
6	-3.129817000	6.732604000	3.001226000
8	-2.230919000	5.843134000	3.062975000
6	-5.031395000	5.775862000	1.645599000
6	-5.797473000	4.438146000	1.776968000
6	-4.909044000	3.333591000	2.289093000
8	-4.199356000	2.626027000	1.530558000
8	-4.888767000	3.227002000	3.624627000
1	-4.749362000	5.639282000	3.808967000
1	-5.669409000	6.492460000	1.117546000
1	-4.149222000	5.609586000	1.014999000
1	-6.172336000	4.137681000	0.796952000
1	-6.652724000	4.539286000	2.447813000
7	-2.769450000	8.044842000	2.863818000
6	-1.405181000	8.396528000	2.444211000
6	-0.308278000	7.886963000	3.374276000
8	0.767667000	7.433195000	2.893967000
1	-3.494065000	8.741504000	2.760512000
1	-1.184711000	7.959874000	1.463998000
7	-0.513446000	7.979641000	4.713917000
6	0.489049000	7.515339000	5.675278000
6	0.579274000	5.986388000	5.801333000
8	1.644251000	5.457873000	6.207981000
1	-1.382159000	8.374851000	5.046583000
1	1.478691000	7.870185000	5.381153000
7	-0.539896000	5.298152000	5.482642000
6	-0.642594000	3.825789000	5.513147000
6	0.207614000	3.235168000	4.368454000
8	1.116345000	2.400389000	4.617055000
6	-2.147216000	3.491622000	5.460725000
6	-2.525927000	2.025607000	5.670968000
8	-2.006122000	1.374770000	6.620279000
8	-3.446287000	1.568642000	4.855275000
1	-1.282346000	5.783731000	4.992025000
1	-0.201185000	3.448188000	6.436941000

1	-2.570620000	3.846647000	4.520124000
1	-2.626717000	4.056689000	6.272320000
7	-0.003750000	3.720602000	3.115231000
6	0.867162000	3.295439000	2.007815000
6	2.317768000	3.760020000	2.240434000
8	3.290585000	3.065049000	1.841371000
6	0.354583000	3.788880000	0.634061000
6	-0.995328000	3.200641000	0.142957000
6	-0.998261000	1.695707000	-0.047505000
8	-0.731434000	0.980035000	0.981955000
8	-1.284590000	1.188905000	-1.210472000
1	-0.765086000	4.370765000	2.937494000
1	0.906844000	2.205850000	1.999473000
1	1.130683000	3.547442000	-0.102705000
1	0.257617000	4.881883000	0.633272000
1	-1.797807000	3.431220000	0.855111000
1	-1.243505000	3.683050000	-0.804944000
7	2.495989000	4.953435000	2.872056000
6	3.824978000	5.458018000	3.207028000
6	4.628516000	4.544707000	4.137552000
8	5.840388000	4.312678000	3.887428000
1	1.703543000	5.556060000	3.063259000
1	4.444589000	5.575606000	2.315575000
7	3.999036000	4.036254000	5.225986000
6	4.710531000	3.121068000	6.117160000
6	4.857989000	1.704557000	5.546040000
8	5.973433000	1.102097000	5.588402000
1	3.062754000	4.348465000	5.480011000
1	5.722335000	3.485624000	6.304428000
7	3.749875000	1.134954000	5.023900000
6	3.819251000	-0.193549000	4.404933000
6	2.677022000	-0.437403000	3.412938000
6	1.356340000	-0.747745000	4.045659000
7	0.202366000	-0.942548000	3.274554000
6	1.002761000	-0.872428000	5.372126000
6	-0.811258000	-1.160151000	4.130070000
7	-0.358221000	-1.132626000	5.399161000
1	2.873408000	1.655747000	4.950204000
1	3.832451000	-0.970731000	5.183711000
1	2.957303000	-1.270365000	2.754740000
1	2.583625000	0.439419000	2.760926000
1	1.575357000	-0.741879000	6.273185000
1	-1.844625000	-1.300188000	3.865101000
6	1.963720000	0.376829000	-5.126382000
6	2.764222000	-0.727895000	-5.785608000
8	2.540924000	-1.952328000	-5.540425000
6	0.685458000	0.774156000	-5.916055000
6	-0.370444000	-0.340656000	-6.109257000
6	-0.843542000	-0.986769000	-4.823805000
8	-1.367436000	-0.129386000	-3.861013000
8	-0.761875000	-2.187806000	-4.579611000
1	1.685434000	0.033427000	-4.124425000
1	0.223265000	1.617981000	-5.396006000
1	0.969712000	1.139644000	-6.911010000
1	0.012250000	-1.148303000	-6.737423000
1	-1.236226000	0.107665000	-6.612302000
7	3.713704000	-0.369144000	-6.687718000
6	4.415762000	-1.350860000	-7.523663000
6	5.317359000	-2.345943000	-6.785203000
8	5.575426000	-3.444107000	-7.324843000
1	3.894176000	0.612586000	-6.840563000
1	3.693815000	-1.959964000	-8.074140000
7	5.800928000	-1.966622000	-5.570370000
6	6.607100000	-2.858987000	-4.752734000
6	5.848545000	-3.431548000	-3.555578000
8	6.474921000	-3.765258000	-2.506435000
1	5.647161000	-1.040627000	-5.180655000
1	6.939572000	-3.690653000	-5.384507000
7	4.520216000	-3.604464000	-3.709588000
6	3.678919000	-4.268781000	-2.707755000
6	2.209637000	-4.284638000	-3.139767000
8	1.707735000	-2.903374000	-3.169263000
6	1.341726000	-5.097642000	-2.184838000
1	4.075045000	-3.234327000	-4.544263000
1	3.743782000	-3.742940000	-1.749588000

1	2.126257000	-4.697220000	-4.154565000
1	1.798812000	-2.521823000	-4.082777000
1	0.297449000	-5.058907000	-2.506827000
1	1.660739000	-6.145420000	-2.168992000
1	1.406439000	-4.699199000	-1.167230000
6	-5.317543000	-4.126788000	2.919422000
6	-6.509668000	-3.491735000	3.625721000
8	-7.571317000	-3.229143000	2.991968000
6	-5.771972000	-5.173580000	1.885579000
6	-4.647921000	-5.650385000	0.923818000
6	-4.070374000	-4.505448000	0.121095000
8	-3.110182000	-3.813494000	0.609295000
7	-4.625276000	-4.199043000	-1.058303000
1	-4.769551000	-3.317943000	2.418584000
1	-6.605191000	-4.753303000	1.312477000
1	-6.166914000	-6.055831000	2.403949000
1	-5.050581000	-6.420718000	0.256206000
1	-3.828927000	-6.101425000	1.493646000
1	-4.279747000	-3.399901000	-1.606476000
1	-5.371954000	-4.760111000	-1.439802000
7	-6.351592000	-3.197310000	4.941334000
6	-7.441761000	-2.626001000	5.730452000
6	-7.905716000	-1.224798000	5.321767000
8	-9.043505000	-0.830807000	5.662436000
1	-5.420888000	-3.262298000	5.359995000
1	-8.328919000	-3.261311000	5.661889000
7	-7.029045000	-0.470016000	4.598468000
6	-7.414821000	0.826896000	4.065102000
6	-7.631744000	0.841269000	2.551093000
8	-7.592429000	1.937826000	1.928802000
1	-6.073427000	-0.784209000	4.420594000
1	-8.356603000	1.114285000	4.546476000
7	-7.900397000	-0.352679000	1.962862000
6	-8.226417000	-0.478956000	0.544947000
6	-7.194522000	-1.284295000	-0.239473000
8	-5.912423000	-0.585095000	-0.264731000
1	-7.918135000	-1.200342000	2.527496000
1	-8.309290000	0.526618000	0.129422000
1	-7.054556000	-2.268809000	0.222771000
1	-7.549706000	-1.420279000	-1.267941000
1	-5.429574000	-0.619904000	-1.128626000
6	-9.713449000	5.661284000	-3.742548000
6	-8.830694000	4.672039000	-3.012088000
6	-9.067455000	4.351762000	-1.663404000
6	-7.772279000	4.023726000	-3.674603000
6	-8.278732000	3.407747000	-0.994297000
6	-6.979847000	3.078774000	-3.011284000
6	-7.228326000	2.767933000	-1.668273000
1	-10.580603000	5.160604000	-4.194351000
1	-10.101949000	6.430017000	-3.065623000
1	-9.881569000	4.842751000	-1.136157000
1	-7.575587000	4.255305000	-4.718342000
1	-8.468099000	3.160793000	0.045704000
1	-6.170575000	2.587203000	-3.543119000
1	-6.619536000	2.036342000	-1.145426000
26	-0.140777000	-0.963218000	1.238949000
26	-1.776842000	-0.736785000	-1.828719000
8	0.338572000	-2.995021000	0.851970000
1	1.333063000	-3.033482000	0.780741000
1	-0.137844000	-3.639036000	1.517025000
8	-4.161118000	-0.762315000	3.934041000
1	-3.878935000	0.081783000	4.416016000
1	-4.135986000	-0.595880000	2.944013000
8	-4.026400000	-0.028952000	1.370344000
1	-4.862213000	-0.250102000	0.825320000
1	-3.945791000	0.963990000	1.467044000
8	-2.092617000	-1.346381000	0.355025000
1	-2.427212000	-2.293049000	0.348093000
1	-2.833131000	-0.735139000	0.768377000
8	-1.047912000	-2.658492000	8.866369000
1	-0.652481000	-3.305008000	8.196039000
1	-1.190759000	-3.045329000	9.742975000
8	-3.919439000	-0.637216000	-2.077380000
8	-3.443583000	-1.955219000	-2.309949000
8	-0.267374000	-2.100859000	-1.625344000

1	0.404411000	-2.396396000	-2.315614000
8	-1.139921000	-4.338682000	2.353883000
1	-1.978831000	-4.290157000	1.831983000
1	-1.300801000	-4.356292000	3.336372000
8	-2.322947000	-1.213742000	7.254640000
1	-1.913939000	-1.646503000	8.082002000
1	-2.370087000	-0.217475000	7.211238000
8	-1.908273000	-4.480133000	4.959441000
1	-2.268447000	-5.376764000	5.091899000
1	-2.637964000	-3.732648000	5.179355000
8	-0.153144000	-4.115734000	6.931889000
1	-0.768302000	-4.260826000	6.150421000
1	0.773608000	-4.065028000	6.649216000
8	-3.547194000	-2.685647000	5.534523000
1	-3.800260000	-2.014594000	4.811428000
1	-3.186875000	-2.150337000	6.330298000
1	4.165405000	3.077473000	7.065009000
1	3.694160000	6.440186000	3.671173000
1	0.254518000	7.937422000	6.656916000
1	-1.342818000	9.487244000	2.372071000
1	-5.207421000	7.261274000	3.266555000
1	-5.663168000	8.600916000	-3.747198000
1	-2.339477000	8.399912000	-7.833303000
1	0.751572000	11.177339000	-5.236442000
1	5.538506000	7.060512000	-4.679337000
1	5.049646000	5.685265000	0.109491000
1	8.109661000	-0.812035000	-2.253626000
1	10.735269000	2.559355000	0.215528000
1	6.629794000	2.663511000	3.205078000
1	8.899352000	-1.018893000	4.535915000
1	5.019321000	-0.801456000	-8.252183000
1	7.484971000	-2.336293000	-4.370162000
1	-7.125138000	-2.601835000	6.777646000
1	-6.667676000	1.589344000	4.297241000
1	-4.624763000	-4.567024000	3.645355000
1	-9.198965000	-0.978165000	0.441277000
1	8.377543000	-3.515944000	1.099730000
1	-9.173227000	6.163418000	-4.552423000
1	2.581397000	1.273834000	-4.996680000
1	4.038205000	-5.295056000	-2.558947000
1	-0.146847000	-2.610404000	-0.779778000
1	4.778775000	-0.260563000	3.882350000
1	-0.989814000	-1.221229000	6.221420000
1	-4.251666000	2.503632000	4.057686000

^{11}TS1$_{BP}$:

6	8.628980000	-1.391934000	3.498020000
6	8.041225000	-0.301791000	2.612249000
8	8.036417000	-0.451095000	1.353927000
6	7.664465000	-2.601705000	3.723174000
6	6.866668000	-2.985345000	2.517341000
7	7.394151000	-3.445262000	1.302870000
6	5.521843000	-2.851609000	2.295026000
6	6.414362000	-3.554409000	0.374184000
7	5.270106000	-3.198648000	0.973365000
1	9.539246000	-1.738080000	2.996752000
1	8.254745000	-3.446847000	4.098318000
1	6.952542000	-2.343686000	4.513787000
1	4.740054000	-2.528435000	2.956677000
1	6.514025000	-3.837742000	-0.673484000
1	4.326705000	-3.027568000	0.521580000
7	7.557717000	0.811279000	3.219733000
6	6.849088000	1.868826000	2.490841000
6	7.592542000	2.467006000	1.321766000
8	6.949289000	3.028592000	0.385033000
1	7.375467000	0.819316000	4.226914000
1	5.891819000	1.511516000	2.094669000
7	8.937863000	2.387619000	1.261874000
6	9.667740000	2.844690000	0.077304000
6	9.201754000	2.211481000	-1.243017000
8	9.412281000	2.815492000	-2.316004000
1	9.436413000	1.957672000	2.026896000
1	9.560426000	3.924104000	-0.054239000
7	8.584001000	0.990106000	-1.181357000
6	8.060722000	0.371613000	-2.391035000
6	6.587708000	0.663138000	-2.702497000
8	6.050691000	0.140045000	-3.718878000
1	8.451582000	0.511814000	-0.289655000
1	8.646108000	0.724751000	-3.245452000
7	5.917645000	1.471474000	-1.848603000
6	4.495881000	1.781001000	-2.039273000
6	4.172019000	3.277639000	-1.975528000
8	3.250757000	3.742017000	-2.709330000
6	3.570354000	1.054751000	-1.024742000
6	3.707857000	-0.474362000	-1.041337000
6	2.686624000	-1.162873000	-0.146203000
8	1.641164000	-0.506414000	0.194149000
8	2.901858000	-2.393709000	0.226892000
1	6.419618000	1.865896000	-1.054822000
1	4.251400000	1.451727000	-3.050934000
1	2.536626000	1.320770000	-1.267862000
1	3.749597000	1.444480000	-0.015679000
1	4.713388000	-0.786340000	-0.735908000
1	3.559017000	-0.874830000	-2.055104000
7	4.823050000	4.034081000	-1.059291000
6	4.316972000	5.333068000	-0.621251000
6	4.163115000	6.390686000	-1.696053000
8	3.266994000	7.269872000	-1.580177000
1	5.516848000	3.612443000	-0.437999000
1	3.329343000	5.231955000	-0.162277000
7	4.995927000	6.365508000	-2.765255000
6	4.810958000	7.327576000	-3.853293000
6	3.401473000	7.280253000	-4.455005000
8	2.886583000	8.318206000	-4.943703000
1	5.708969000	5.651851000	-2.823643000
1	4.963188000	8.350716000	-3.501547000
7	2.765147000	6.078005000	-4.441301000
6	1.423524000	5.941939000	-5.008129000
6	0.346382000	6.664931000	-4.190389000
8	-0.790504000	6.855837000	-4.708180000
6	1.062996000	4.463053000	-5.207417000
1	3.141385000	5.284019000	-3.922499000
1	1.411972000	6.455253000	-5.976209000
1	0.038466000	4.387661000	-5.578979000
1	1.738888000	4.009960000	-5.940236000
1	1.148880000	3.912468000	-4.265393000
7	0.666911000	7.127284000	-2.959805000
6	-0.274283000	7.957594000	-2.211489000
6	-0.717434000	9.193154000	-3.015019000
8	-1.861368000	9.680856000	-2.815301000
6	0.328510000	8.391986000	-0.871211000
8	0.247811000	7.255119000	0.039417000
1	1.583750000	6.962961000	-2.549999000
1	-1.193840000	7.399514000	-2.010182000
1	1.367716000	8.708722000	-1.003109000
1	-0.263187000	9.232147000	-0.485125000
1	0.774390000	7.396782000	0.859821000
7	0.176474000	9.723456000	-3.888628000
6	-0.152988000	10.904488000	-4.683829000
6	-1.214311000	10.703174000	-5.771418000
8	-1.693776000	11.705713000	-6.345505000
1	1.065543000	9.260430000	-4.065476000
1	-0.533283000	11.699174000	-4.035996000
7	-1.582728000	9.420017000	-6.053698000
6	-2.646745000	9.125294000	-7.000723000
6	-3.836879000	8.404224000	-6.376329000
8	-4.608652000	7.720410000	-7.099073000
1	-1.151227000	8.630725000	-5.578923000
1	-2.982569000	10.078876000	-7.423753000
7	-4.008874000	8.543855000	-5.032299000
6	-5.145550000	7.947842000	-4.331997000
6	-4.745196000	6.904611000	-3.274725000
6	-4.096634000	5.622775000	-3.744658000
6	-3.960975000	5.265264000	-5.115448000
6	-3.620248000	4.715741000	-2.749482000
6	-3.334291000	4.089606000	-5.473294000
6	-3.007921000	3.531205000	-3.083270000
6	-2.817988000	3.193898000	-4.471441000
8	-2.187744000	2.110124000	-4.830322000
1	-3.333589000	9.077307000	-4.491870000

1	-5.798847000	7.515876000	-5.094493000
1	-5.650449000	6.632759000	-2.707126000
1	-4.070258000	7.384391000	-2.548813000
1	-4.337820000	5.924000000	-5.891294000
1	-3.758417000	4.969570000	-1.702626000
1	-3.207869000	3.811933000	-6.513487000
1	-2.651392000	2.833915000	-2.332497000
1	-1.655976000	0.983582000	-4.058523000
6	-4.611221000	6.406448000	3.003031000
6	-3.146617000	6.759298000	3.000630000
8	-2.244100000	5.874126000	3.063888000
6	-5.027663000	5.806674000	1.613650000
6	-5.758248000	4.446996000	1.719988000
6	-4.846863000	3.363043000	2.237478000
8	-4.093751000	2.692996000	1.486908000
8	-4.859627000	3.232810000	3.570491000
1	-4.772589000	5.652978000	3.780124000
1	-5.682336000	6.515138000	1.095011000
1	-4.139276000	5.673397000	0.983672000
1	-6.107922000	4.143716000	0.731261000
1	-6.626977000	4.519675000	2.377255000
7	-2.791399000	8.074660000	2.876621000
6	-1.426772000	8.435416000	2.466111000
6	-0.330762000	7.923825000	3.396210000
8	0.745230000	7.470502000	2.915487000
1	-3.518538000	8.768800000	2.774214000
1	-1.201089000	8.006297000	1.483951000
7	-0.535861000	8.015597000	4.735848000
6	0.468980000	7.553734000	5.696413000
6	0.566789000	6.024733000	5.816277000
8	1.638871000	5.498277000	6.206185000
1	-1.403912000	8.411578000	5.069343000
1	1.456946000	7.914450000	5.403850000
7	-0.554473000	5.334130000	5.509643000
6	-0.652149000	3.861190000	5.531892000
6	0.203802000	3.279870000	4.387506000
8	1.115784000	2.448359000	4.632831000
6	-2.155151000	3.521090000	5.469327000
6	-2.520132000	2.047025000	5.649083000
8	-2.008480000	1.392858000	6.600410000
8	-3.414866000	1.588086000	4.807483000
1	-1.305160000	5.820929000	5.033788000
1	-0.214494000	3.478780000	6.455471000
1	-2.577815000	3.891561000	4.534257000
1	-2.639837000	4.066166000	6.291274000
7	-0.009512000	3.769697000	3.135865000
6	0.858311000	3.344554000	2.027156000
6	2.309423000	3.810910000	2.249979000
8	3.278953000	3.119814000	1.835618000
6	0.337773000	3.830817000	0.654568000
6	-1.018743000	3.246895000	0.179713000
6	-1.032029000	1.743367000	-0.001740000
8	-0.681683000	1.015836000	0.980072000
8	-1.427027000	1.247790000	-1.157942000
1	-0.772244000	4.419018000	2.960928000
1	0.900222000	2.255040000	2.022461000
1	1.107594000	3.581433000	-0.086135000
1	0.244281000	4.923984000	0.646578000
1	-1.815882000	3.481311000	0.896756000
1	-1.277794000	3.726051000	-0.766572000
7	2.490755000	4.998719000	2.890064000
6	3.820260000	5.498734000	3.230284000
6	4.623246000	4.570428000	4.146581000
8	5.832048000	4.332163000	3.887620000
1	1.697974000	5.597010000	3.092820000
1	4.438842000	5.630211000	2.340128000
7	3.995262000	4.055403000	5.232678000
6	4.704489000	3.126487000	6.111513000
6	4.855320000	1.719277000	5.518633000
8	5.970455000	1.116037000	5.559485000
1	3.060550000	4.368799000	5.490718000
1	5.715468000	3.488394000	6.308435000
7	3.751195000	1.158755000	4.979041000
6	3.825026000	-0.160481000	4.340754000
6	2.680322000	-0.397182000	3.350058000
6	1.363951000	-0.719960000	3.985153000
7	0.215860000	-0.946421000	3.214768000
6	1.010623000	-0.829078000	5.313347000
6	-0.795325000	-1.167597000	4.072330000
7	-0.345376000	-1.111234000	5.342292000
1	2.874123000	1.679447000	4.912262000
1	3.843754000	-0.948057000	5.108810000
1	2.960617000	-1.222743000	2.682673000
1	2.582720000	0.486299000	2.707109000
1	1.579842000	-0.675507000	6.212924000
1	-1.825579000	-1.331297000	3.808085000
6	1.912073000	0.424056000	-5.072884000
6	2.688302000	-0.689066000	-5.746543000
8	2.419807000	-1.912119000	-5.539785000
6	0.638844000	0.854606000	-5.853245000
6	-0.455637000	-0.224845000	-6.024833000
6	-0.937879000	-0.848230000	-4.733968000
8	-1.334771000	0.034466000	-3.728173000
8	-0.970352000	-2.054855000	-4.507291000
1	1.631139000	0.077300000	-4.072609000
1	0.209690000	1.717902000	-5.337468000
1	0.924676000	1.200975000	-6.854441000
1	-0.112542000	-1.049416000	-6.653974000
1	-1.313447000	0.249354000	-6.518437000
7	3.667218000	-0.337599000	-6.618081000
6	4.363403000	-1.319540000	-7.458851000
6	5.221195000	-2.352085000	-6.719300000
8	5.449603000	-3.452819000	-7.266356000
1	3.885630000	0.641210000	-6.736383000
1	3.639164000	-1.897951000	-8.038340000
7	5.701919000	-2.001150000	-5.494108000
6	6.477358000	-2.927643000	-4.684035000
6	5.685481000	-3.539119000	-3.528033000
8	6.286184000	-3.950305000	-2.492835000
1	5.587941000	-1.068741000	-5.104472000
1	6.824970000	-3.740592000	-5.331739000
7	4.354080000	-3.658960000	-3.702917000
6	3.482517000	-4.343772000	-2.740514000
6	2.028959000	-4.350686000	-3.220669000
8	1.531682000	-2.965657000	-3.242023000
6	1.126175000	-5.177862000	-2.311405000
1	3.932504000	-3.243166000	-4.527616000
1	3.519296000	-3.837669000	-1.769876000
1	1.977540000	-4.740480000	-4.246355000
1	1.662028000	-2.549301000	-4.135443000
1	0.091490000	-5.123382000	-2.660694000
1	1.436810000	-6.228119000	-2.313786000
1	1.162410000	-4.809294000	-1.281028000
6	-5.420417000	-4.327954000	3.087580000
6	-6.545461000	-3.530397000	3.730658000
8	-7.587504000	-3.242095000	3.075131000
6	-5.983129000	-5.536687000	2.311977000
6	-4.923066000	-6.281566000	1.457549000
6	-4.237596000	-5.334736000	0.493007000
8	-3.136654000	-4.782016000	0.779482000
7	-4.887166000	-5.020406000	-0.648993000
1	-4.889661000	-3.657489000	2.399079000
1	-6.800806000	-5.181884000	1.675163000
1	-6.424068000	-6.253306000	3.015945000
1	-5.407797000	-7.102862000	0.915865000
1	-4.150689000	-6.716412000	2.099131000
1	-4.501872000	-4.272090000	-1.222395000
1	-5.759728000	-5.454454000	-0.904891000
7	-6.363468000	-3.147459000	5.020898000
6	-7.425347000	-2.488400000	5.779566000
6	-7.875800000	-1.119686000	5.264689000
8	-9.013727000	-0.693744000	5.565430000
1	-5.431511000	-3.227503000	5.433300000
1	-8.325017000	-3.109975000	5.785761000
7	-6.984195000	-0.422871000	4.503940000
6	-7.350953000	0.841790000	3.888820000
6	-7.504919000	0.779489000	2.369393000
8	-7.454281000	1.847226000	1.699373000
1	-6.030121000	-0.757357000	4.353664000
1	-8.313857000	1.146358000	4.315277000

7	-7.735093000	-0.445018000	1.828073000
6	-8.039319000	-0.641375000	0.412368000
6	-6.971067000	-1.417093000	-0.351902000
8	-5.768691000	-0.592272000	-0.495415000
1	-7.780519000	-1.262446000	2.436141000
1	-8.173810000	0.341989000	-0.042429000
1	-6.720261000	-2.348161000	0.171508000
1	-7.355365000	-1.661733000	-1.349686000
1	-5.185073000	-0.897975000	-1.228213000
6	-9.303928000	5.931558000	-3.665900000
6	-8.497313000	4.758769000	-3.149533000
6	-8.588705000	4.353669000	-1.805826000
6	-7.666930000	4.020248000	-4.012565000
6	-7.881692000	3.239690000	-1.334299000
6	-6.957638000	2.906232000	-3.547596000
6	-7.060430000	2.512475000	-2.207668000
1	-10.270620000	5.600756000	-4.069272000
1	-9.517297000	6.654583000	-2.871034000
1	-9.230913000	4.909230000	-1.126738000
1	-7.586438000	4.314574000	-5.055938000
1	-7.966462000	2.927315000	-0.297239000
1	-6.327444000	2.346958000	-4.233544000
1	-6.513926000	1.650334000	-1.836604000
26	-0.150070000	-1.019160000	1.180063000
26	-1.647174000	-0.664516000	-1.718643000
8	0.332934000	-3.051744000	0.760819000
1	1.324898000	-3.091679000	0.707727000
1	-0.156838000	-3.705869000	1.435380000
8	-4.133167000	-0.758054000	3.872061000
1	-3.845809000	0.083083000	4.349646000
1	-4.113548000	-0.594396000	2.879939000
8	-3.995718000	0.009991000	1.324939000
1	-4.776581000	-0.203140000	0.714687000
1	-3.905122000	0.998444000	1.420326000
8	-1.992780000	-1.333975000	0.281002000
1	-2.728410000	-2.256153000	-0.301655000
1	-2.697372000	-0.749585000	0.738882000
8	-1.009128000	-2.631786000	8.828100000
1	-0.601746000	-3.271406000	8.156676000
1	-1.169928000	-3.031351000	9.695965000
8	-3.489194000	-1.383007000	-1.942452000
8	-3.490267000	-2.580748000	-1.125518000
8	-0.300213000	-2.129617000	-1.640914000
1	0.339081000	-2.460876000	-2.361921000
8	-0.996661000	-4.475881000	2.295263000
1	-1.825514000	-4.720786000	1.805609000
1	-1.166674000	-4.365819000	3.266622000
8	-2.296552000	-1.201326000	7.209450000
1	-1.883855000	-1.632968000	8.035604000
1	-2.351886000	-0.205445000	7.171411000
8	-1.824249000	-4.435983000	4.918660000
1	-2.175605000	-5.340438000	5.017887000
1	-2.574003000	-3.711820000	5.123352000
8	-0.093347000	-4.077638000	6.899709000
1	-0.707731000	-4.221313000	6.115176000
1	0.833383000	-4.020929000	6.618277000
8	-3.523080000	-2.681068000	5.488424000
1	-3.783465000	-2.019942000	4.760387000
1	-3.161142000	-2.139990000	6.277488000
1	4.156097000	3.067445000	7.056588000
1	3.689737000	6.473361000	3.709984000
1	0.231277000	7.971525000	6.679113000
1	-1.368966000	9.526881000	2.402190000
1	-5.228965000	7.277836000	3.245257000
1	-5.710582000	8.740180000	-3.825944000
1	-2.291509000	8.498475000	-7.823929000
1	0.763702000	11.263341000	-5.159050000
1	5.549061000	7.116499000	-4.632549000
1	5.006112000	5.703670000	0.143943000
1	8.169758000	-0.715858000	-2.328788000
1	10.729257000	2.624923000	0.224006000
1	6.611769000	2.679772000	3.193062000
1	8.920683000	-0.997231000	4.476135000
1	4.997669000	-0.770485000	-8.161069000
1	7.346123000	-2.421701000	-4.259646000
1	-7.083556000	-2.381309000	6.813818000
1	-6.619877000	1.621497000	4.114642000
1	-4.690163000	-4.649431000	3.837777000
1	-8.983505000	-1.194554000	0.327587000
1	8.373415000	-3.583489000	1.106862000
1	-8.782889000	6.457479000	-4.473899000
1	2.546666000	1.308126000	-4.937339000
1	3.838442000	-5.372327000	-2.603164000
1	-0.168482000	-2.639392000	-0.781409000
1	4.783192000	-0.215576000	3.814192000
1	-0.975574000	-1.200166000	6.166172000
1	-4.213671000	2.515779000	4.009937000

^{11}IM1$_{BP}$:

6	8.665220000	-1.390721000	3.529013000
6	8.075278000	-0.293524000	2.653846000
8	8.069945000	-0.431411000	1.394345000
6	7.706564000	-2.606914000	3.740996000
6	6.915993000	-2.994558000	2.531461000
7	7.449809000	-3.453309000	1.319149000
6	5.568833000	-2.884392000	2.309726000
6	6.470062000	-3.586736000	0.392939000
7	5.320958000	-3.246698000	0.991365000
1	9.577923000	-1.727702000	3.025797000
1	8.300421000	-3.450079000	4.115299000
1	6.989103000	-2.358625000	4.529804000
1	4.782609000	-2.568862000	2.970202000
1	6.568537000	-3.876438000	-0.652587000
1	4.372775000	-3.095907000	0.537935000
7	7.591180000	0.814145000	3.270819000
6	6.886638000	1.878885000	2.548341000
6	7.637990000	2.478055000	1.384563000
8	7.003285000	3.021370000	0.432429000
1	7.410430000	0.817388000	4.277836000
1	5.928836000	1.528266000	2.148144000
7	8.985767000	2.419747000	1.350747000
6	9.732042000	2.893851000	0.183267000
6	9.304150000	2.261368000	-1.149742000
8	9.528451000	2.875755000	-2.214003000
1	9.476490000	2.005437000	2.129480000
1	9.610521000	3.972236000	0.054813000
7	8.706581000	1.029094000	-1.109241000
6	8.219039000	0.411524000	-2.333888000
6	6.740740000	0.655165000	-2.659592000
8	6.240824000	0.131645000	-3.693766000
1	8.557009000	0.544942000	-0.223731000
1	8.801065000	0.800763000	-3.174743000
7	6.029649000	1.424265000	-1.802466000
6	4.603838000	1.700727000	-2.014529000
6	4.251303000	3.193616000	-1.975198000
8	3.326657000	3.631005000	-2.721646000
6	3.679895000	0.972578000	-1.000375000
6	3.813733000	-0.558164000	-1.009864000
6	2.777287000	-1.233957000	-0.118814000
8	1.758796000	-0.545332000	0.235500000
8	2.948884000	-2.475886000	0.238686000
1	6.507345000	1.823230000	-0.996318000
1	4.378915000	1.352586000	-3.024536000
1	2.646242000	1.237415000	-1.243737000
1	3.857128000	1.366017000	0.007588000
1	4.815993000	-0.872725000	-0.695635000
1	3.669200000	-0.962713000	-2.022638000
7	4.887709000	3.972378000	-1.067418000
6	4.359327000	5.264217000	-0.634420000
6	4.193155000	6.318961000	-1.708813000
8	3.293318000	7.194123000	-1.588458000
1	5.583275000	3.566988000	-0.438014000
1	3.373827000	5.145703000	-0.176940000
7	5.018556000	6.294076000	-2.783753000
6	4.820139000	7.248527000	-3.875389000
6	3.405052000	7.192905000	-4.461596000
8	2.885576000	8.225100000	-4.958230000
1	5.734063000	5.583078000	-2.843619000
1	4.972765000	8.274559000	-3.532185000
7	2.767128000	5.992099000	-4.425493000

6	1.419901000	5.853721000	-4.978259000
6	0.354224000	6.605455000	-4.171707000
8	-0.781541000	6.798599000	-4.691301000
6	1.044566000	4.374352000	-5.138282000
1	3.151778000	5.199977000	-3.909566000
1	1.404493000	6.342961000	-5.958754000
1	0.017514000	4.300089000	-5.503004000
1	1.712041000	3.895676000	-5.862308000
1	1.129509000	3.845983000	-4.183477000
7	0.681773000	7.090575000	-2.951813000
6	-0.249358000	7.952645000	-2.225990000
6	-0.675084000	9.173344000	-3.061696000
8	-1.812686000	9.680351000	-2.875863000
6	0.354089000	8.408407000	-0.892948000
8	0.233340000	7.299763000	0.047455000
1	1.598324000	6.926769000	-2.540133000
1	-1.177204000	7.412401000	-2.014341000
1	1.402922000	8.693102000	-1.023935000
1	-0.217102000	9.274735000	-0.535276000
1	0.753671000	7.447168000	0.871101000
7	0.225496000	9.669757000	-3.948671000
6	-0.092618000	10.831401000	-4.776165000
6	-1.143838000	10.607099000	-5.869458000
8	-1.607535000	11.596341000	-6.478606000
1	1.109511000	9.192678000	-4.114981000
1	-0.476318000	11.642894000	-4.151635000
7	-1.519698000	9.319322000	-6.119089000
6	-2.571418000	9.004424000	-7.073216000
6	-3.775347000	8.307423000	-6.447807000
8	-4.545892000	7.617018000	-7.165534000
1	-1.101382000	8.540538000	-5.616023000
1	-2.895551000	9.947600000	-7.527473000
7	-3.962590000	8.479383000	-5.109729000
6	-5.116248000	7.915200000	-4.411233000
6	-4.745957000	6.884470000	-3.330953000
6	-4.116719000	5.581629000	-3.769816000
6	-3.997993000	5.182397000	-5.130193000
6	-3.648717000	4.695628000	-2.752565000
6	-3.400582000	3.982138000	-5.458184000
6	-3.065726000	3.488141000	-3.056204000
6	-2.895902000	3.103211000	-4.434754000
8	-2.293447000	1.995807000	-4.763222000
1	-3.288249000	9.018225000	-4.573977000
1	-5.768969000	7.481029000	-5.172843000
1	-5.663095000	6.639322000	-2.769846000
1	-4.071580000	7.364934000	-2.605313000
1	-4.361559000	5.829237000	-5.922543000
1	-3.769993000	4.985565000	-1.712972000
1	-3.288234000	3.672262000	-6.490936000
1	-2.716906000	2.804993000	-2.289879000
1	-1.743032000	0.839737000	-3.958700000
6	-4.607701000	6.367906000	3.025223000
6	-3.150621000	6.749732000	3.012812000
8	-2.232191000	5.880548000	3.073055000
6	-5.015476000	5.735893000	1.647988000
6	-5.752402000	4.382438000	1.782871000
6	-4.847566000	3.305554000	2.326068000
8	-4.091936000	2.617486000	1.594041000
8	-4.868854000	3.201287000	3.661899000
1	-4.751268000	5.623839000	3.815041000
1	-5.663474000	6.433775000	1.107029000
1	-4.122638000	5.583875000	1.028765000
1	-6.102537000	4.060410000	0.800321000
1	-6.622574000	4.471529000	2.435787000
7	-2.819306000	8.070519000	2.886636000
6	-1.461562000	8.457662000	2.475957000
6	-0.355517000	7.955065000	3.399601000
8	0.725769000	7.520509000	2.913341000
1	-3.559044000	8.751614000	2.786976000
1	-1.231022000	8.043653000	1.488252000
7	-0.557783000	8.034457000	4.740512000
6	0.456468000	7.580449000	5.695048000
6	0.574343000	6.052289000	5.809226000
8	1.653606000	5.539398000	6.198004000
1	-1.430472000	8.415207000	5.079574000
1	1.438562000	7.954683000	5.399705000
7	-0.537414000	5.347648000	5.501078000
6	-0.614446000	3.873251000	5.517424000
6	0.230144000	3.310515000	4.354345000
8	1.146796000	2.478033000	4.580707000
6	-2.113116000	3.512954000	5.483405000
6	-2.471794000	2.042761000	5.710086000
8	-1.923994000	1.398284000	6.648229000
8	-3.411110000	1.579168000	4.921085000
1	-1.294551000	5.825276000	5.025696000
1	-0.153667000	3.493576000	6.430856000
1	-2.552172000	3.853761000	4.544515000
1	-2.593961000	4.076923000	6.295197000
7	0.005775000	3.817244000	3.111208000
6	0.876093000	3.417628000	1.994084000
6	2.322743000	3.895410000	2.218170000
8	3.294918000	3.216426000	1.790405000
6	0.348411000	3.898962000	0.620563000
6	-0.980619000	3.273588000	0.116003000
6	-0.942276000	1.767003000	-0.043438000
8	-0.498386000	1.087791000	0.931003000
8	-1.380863000	1.223643000	-1.166578000
1	-0.761174000	4.465433000	2.950190000
1	0.935126000	2.330058000	1.981589000
1	1.134821000	3.678705000	-0.112252000
1	0.221081000	4.988579000	0.617891000
1	-1.802635000	3.489650000	0.812322000
1	-1.231978000	3.736580000	-0.840588000
7	2.501132000	5.073001000	2.877591000
6	3.831534000	5.568199000	3.222409000
6	4.635016000	4.625626000	4.124295000
8	5.839960000	4.379406000	3.856138000
1	1.708219000	5.669832000	3.084211000
1	4.448077000	5.714903000	2.333258000
7	4.010291000	4.111882000	5.213676000
6	4.723852000	3.193039000	6.098868000
6	4.875225000	1.777010000	5.528502000
8	5.988493000	1.172181000	5.591111000
1	3.077432000	4.428372000	5.474951000
1	5.735478000	3.558042000	6.287328000
7	3.775182000	1.209173000	4.988237000
6	3.851846000	-0.124670000	4.381400000
6	2.726040000	-0.375613000	3.372821000
6	1.397708000	-0.692842000	3.985939000
7	0.264600000	-0.921175000	3.195666000
6	1.019814000	-0.795269000	5.308653000
6	-0.763207000	-1.136617000	4.034257000
7	-0.337652000	-1.073789000	5.312295000
1	2.899217000	1.729628000	4.903979000
1	3.850203000	-0.895153000	5.166782000
1	3.020977000	-1.208571000	2.721004000
1	2.636817000	0.499021000	2.716836000
1	1.569815000	-0.633979000	6.218943000
1	-1.787800000	-1.304500000	3.752391000
6	1.875214000	0.402553000	-4.950608000
6	2.669941000	-0.667673000	-5.669709000
8	2.399381000	-1.900871000	-5.534104000
6	0.586789000	0.833271000	-5.706930000
6	-0.483700000	-0.260408000	-5.928962000
6	-0.980574000	-0.942522000	-4.671561000
8	-1.416410000	-0.098580000	-3.642936000
8	-0.996953000	-2.156271000	-4.499003000
1	1.607094000	0.011064000	-3.963030000
1	0.139862000	1.661803000	-5.151227000
1	0.860975000	1.232763000	-6.691570000
1	-0.115660000	-1.055725000	-6.581146000
1	-1.342188000	0.216310000	-6.418750000
7	3.669721000	-0.274028000	-6.498002000
6	4.386895000	-1.219143000	-7.364308000
6	5.201335000	-2.302208000	-6.646815000
8	5.393637000	-3.397066000	-7.218927000
1	3.889498000	0.709201000	-6.566591000
1	3.679381000	-1.756470000	-8.000869000
7	5.690409000	-1.997695000	-5.412295000
6	6.443944000	-2.963943000	-4.626992000

6	5.635989000	-3.594214000	-3.491563000
8	6.219598000	-4.031009000	-2.457063000
1	5.623611000	-1.065596000	-5.010955000
1	6.779553000	-3.764124000	-5.296658000
7	4.306122000	-3.699819000	-3.685507000
6	3.411862000	-4.384805000	-2.744299000
6	1.959745000	-4.318129000	-3.224113000
8	1.530093000	-2.910610000	-3.228401000
6	1.017642000	-5.118339000	-2.331146000
1	3.901013000	-3.271115000	-4.511597000
1	3.471791000	-3.912965000	-1.757984000
1	1.891997000	-4.689941000	-4.255628000
1	1.658509000	-2.509234000	-4.130330000
1	-0.010792000	-5.013733000	-2.687638000
1	1.284168000	-6.180723000	-2.345743000
1	1.063286000	-4.764171000	-1.296336000
6	-5.354818000	-4.165999000	2.939911000
6	-6.519588000	-3.506804000	3.665887000
8	-7.594863000	-3.253935000	3.050316000
6	-5.846017000	-5.345765000	2.077772000
6	-4.785959000	-5.894161000	1.081746000
6	-4.397433000	-4.865577000	0.047229000
8	-3.547600000	-3.954455000	0.329572000
7	-5.002769000	-4.889544000	-1.155984000
1	-4.896523000	-3.403287000	2.297407000
1	-6.742852000	-5.027016000	1.535414000
1	-6.151943000	-6.174216000	2.728517000
1	-5.179265000	-6.794851000	0.597196000
1	-3.874354000	-6.176933000	1.619753000
1	-4.759094000	-4.184659000	-1.848539000
1	-5.676778000	-5.599010000	-1.397880000
7	-6.333507000	-3.192951000	4.972598000
6	-7.408351000	-2.607609000	5.773513000
6	-7.897879000	-1.225749000	5.329136000
8	-9.046365000	-0.846797000	5.651306000
1	-5.395829000	-3.256185000	5.375808000
1	-8.290423000	-3.253137000	5.750989000
7	-7.027035000	-0.470343000	4.600550000
6	-7.428312000	0.808568000	4.036224000
6	-7.602784000	0.795037000	2.516510000
8	-7.551285000	1.881433000	1.877316000
1	-6.060833000	-0.768236000	4.445307000
1	-8.389090000	1.080847000	4.488211000
7	-7.851210000	-0.409977000	1.942203000
6	-8.135943000	-0.563939000	0.517805000
6	-7.065351000	-1.350987000	-0.233606000
8	-5.808115000	-0.614016000	-0.226813000
1	-7.884261000	-1.247162000	2.522031000
1	-8.232997000	0.433834000	0.086715000
1	-6.920335000	-2.332060000	0.237653000
1	-7.393044000	-1.499875000	-1.269905000
1	-5.217270000	-0.767716000	-1.013518000
6	-9.544314000	5.660564000	-3.775448000
6	-8.695134000	4.616410000	-3.081850000
6	-8.890102000	4.315451000	-1.721715000
6	-7.715748000	3.896236000	-3.789627000
6	-8.137570000	3.320909000	-1.085071000
6	-6.959603000	2.900838000	-3.158508000
6	-7.165213000	2.610208000	-1.804201000
1	-10.491048000	5.230460000	-4.129495000
1	-9.798929000	6.485799000	-3.101002000
1	-9.646046000	4.859573000	-1.160783000
1	-7.553069000	4.111879000	-4.842538000
1	-8.297878000	3.085517000	-0.037367000
1	-6.212753000	2.352486000	-3.725526000
1	-6.583864000	1.839233000	-1.306976000
26	-0.070200000	-0.952022000	1.151165000
26	-1.814904000	-0.701376000	-1.599277000
8	0.302340000	-3.027267000	0.721188000
1	1.285209000	-3.151326000	0.710923000
1	-0.303461000	-3.635638000	1.321996000
8	-4.167457000	-0.754434000	4.026567000
1	-3.872885000	0.085584000	4.503649000
1	-4.118119000	-0.601626000	3.028484000
8	-3.991336000	-0.078002000	1.503102000
1	-4.804280000	-0.295796000	0.928861000
1	-3.889135000	0.912198000	1.562387000
8	-1.897783000	-1.157220000	0.283035000
1	-3.393996000	-2.751185000	-0.893859000
1	-2.697967000	-0.791289000	0.791738000
8	-0.750518000	-2.731163000	8.599595000
1	-0.421783000	-3.334205000	7.856508000
1	-0.750766000	-3.154803000	9.470887000
8	-3.729730000	-0.981067000	-1.860538000
8	-3.431563000	-2.475533000	-1.870589000
8	-0.297096000	-2.012908000	-1.638909000
1	0.329629000	-2.343337000	-2.366470000
8	-1.412756000	-4.240390000	2.053216000
1	-2.243982000	-4.113038000	1.533977000
1	-1.555482000	-4.282631000	3.039063000
8	-2.235159000	-1.222782000	7.238480000
1	-1.732932000	-1.683114000	7.993999000
1	-2.285850000	-0.227023000	7.236168000
8	-1.949677000	-4.446020000	4.718139000
1	-2.273333000	-5.352410000	4.876579000
1	-2.653178000	-3.711605000	5.066333000
8	0.006976000	-4.055704000	6.501972000
1	-0.683112000	-4.224358000	5.791360000
1	0.839524000	-3.749564000	6.105605000
8	-3.515385000	-2.696061000	5.538359000
1	-3.790391000	-1.989660000	4.845933000
1	-3.130401000	-2.187391000	6.334083000
1	4.180369000	3.145764000	7.047602000
1	3.701804000	6.534627000	3.718742000
1	0.218044000	7.991471000	6.680444000
1	-1.422018000	9.550560000	2.423526000
1	-5.243544000	7.229495000	3.255246000
1	-5.672712000	8.726199000	-3.925654000
1	-2.207691000	8.353225000	-7.873594000
1	0.829543000	11.175619000	-5.251628000
1	5.550690000	7.035074000	-4.661091000
1	5.038868000	5.644921000	0.134319000
1	8.367955000	-0.672027000	-2.290602000
1	10.793736000	2.690053000	0.350535000
1	6.652766000	2.686789000	3.254769000
1	8.954139000	-1.005312000	4.511738000
1	5.054768000	-0.640760000	-8.009679000
1	7.318495000	-2.484381000	-4.184492000
1	-7.061168000	-2.545495000	6.809503000
1	-6.705297000	1.592127000	4.275160000
1	-4.582658000	-4.491908000	3.644758000
1	-9.092861000	-1.088524000	0.397452000
1	8.431072000	-3.577853000	1.123914000
1	-9.034108000	6.080357000	-4.649045000
1	2.489093000	1.295792000	-4.782648000
1	3.726993000	-5.430918000	-2.642334000
1	-0.175864000	-2.553950000	-0.798489000
1	4.819972000	-0.198843000	3.875902000
1	-0.975563000	-1.166592000	6.128792000
1	-4.225359000	2.493514000	4.115004000

^{11}TS2$_{BP}$:

6	8.638746000	-1.358426000	3.547418000
6	8.023212000	-0.246144000	2.709294000
8	7.989094000	-0.359273000	1.447529000
6	7.748371000	-2.637266000	3.669783000
6	6.994604000	-3.009335000	2.431997000
7	7.557020000	-3.447727000	1.224424000
6	5.650417000	-2.917001000	2.187227000
6	6.594647000	-3.588157000	0.280054000
7	5.431976000	-3.270494000	0.862162000
1	9.583112000	-1.620235000	3.056982000
1	8.384332000	-3.464423000	4.008736000
1	7.006845000	-2.476782000	4.459213000
1	4.846861000	-2.618890000	2.835006000
1	6.699455000	-3.872631000	-0.763338000
1	4.489846000	-3.134358000	0.407848000
7	7.565270000	0.856616000	3.353766000
6	6.890118000	1.954356000	2.651290000
6	7.674886000	2.565188000	1.514886000

8	7.072961000	3.134530000	0.557100000
1	7.396582000	0.850208000	4.362791000
1	5.930165000	1.637326000	2.229955000
7	9.022949000	2.487082000	1.515902000
6	9.807830000	2.975765000	0.380136000
6	9.420148000	2.360850000	-0.972754000
8	9.691842000	2.980026000	-2.022833000
1	9.487059000	2.064459000	2.306588000
1	9.692049000	4.056053000	0.262588000
7	8.801922000	1.138190000	-0.960843000
6	8.331977000	0.537956000	-2.199567000
6	6.836979000	0.703029000	-2.487657000
8	6.334168000	0.103447000	-3.477741000
1	8.611749000	0.651319000	-0.085127000
1	8.875950000	1.002241000	-3.028763000
7	6.121369000	1.503583000	-1.662738000
6	4.693020000	1.758673000	-1.885683000
6	4.325174000	3.247083000	-1.876875000
8	3.382315000	3.653236000	-2.617358000
6	3.761526000	1.036663000	-0.874151000
6	3.945392000	-0.486109000	-0.840053000
6	2.865624000	-1.198195000	-0.033365000
8	1.833802000	-0.528321000	0.326955000
8	3.024145000	-2.460148000	0.252797000
1	6.598754000	1.945351000	-0.879000000
1	4.478837000	1.392630000	-2.892233000
1	2.729487000	1.263410000	-1.159879000
1	3.895909000	1.464885000	0.125636000
1	4.925549000	-0.752798000	-0.425527000
1	3.925394000	-0.908214000	-1.855313000
7	4.971536000	4.057523000	-1.004331000
6	4.452955000	5.373191000	-0.638470000
6	4.308556000	6.371621000	-1.769766000
8	3.426174000	7.268002000	-1.698283000
1	5.659626000	3.672865000	-0.354110000
1	3.460132000	5.291387000	-0.187164000
7	5.136773000	6.272304000	-2.838200000
6	4.972308000	7.179888000	-3.974958000
6	3.562273000	7.136953000	-4.574708000
8	3.073788000	8.166424000	-5.106457000
1	5.842822000	5.549719000	-2.853644000
1	5.148473000	8.216432000	-3.677498000
7	2.894802000	5.953759000	-4.513698000
6	1.548232000	5.830969000	-5.081160000
6	0.510060000	6.682110000	-4.338329000
8	-0.578981000	6.965306000	-4.907190000
6	1.118640000	4.357962000	-5.138023000
1	3.255883000	5.171206000	-3.969855000
1	1.564483000	6.248613000	-6.093514000
1	0.097557000	4.282981000	-5.518345000
1	1.783286000	3.802961000	-5.809467000
1	1.162205000	3.898947000	-4.145376000
7	0.819484000	7.133475000	-3.097916000
6	-0.082498000	8.036465000	-2.389483000
6	-0.397559000	9.306355000	-3.200463000
8	-1.492224000	9.899996000	-3.007109000
6	0.497137000	8.427179000	-1.027109000
8	0.352634000	7.280584000	-0.133106000
1	1.712710000	6.919750000	-2.662786000
1	-1.050043000	7.550241000	-2.229410000
1	1.550715000	8.705687000	-1.125732000
1	-0.074774000	9.282612000	-0.645624000
1	0.840561000	7.411132000	0.712494000
7	0.547278000	9.748356000	-4.067242000
6	0.334569000	10.952040000	-4.867770000
6	-0.754941000	10.857675000	-5.941631000
8	-1.145625000	11.907064000	-6.499988000
1	1.387624000	9.201604000	-4.244125000
1	0.047816000	11.787599000	-4.223242000
7	-1.243793000	9.617467000	-6.231605000
6	-2.350082000	9.440564000	-7.160185000
6	-3.628413000	8.912339000	-6.514340000
8	-4.524683000	8.400962000	-7.234516000
1	-0.874482000	8.785837000	-5.776664000
1	-2.556935000	10.415926000	-7.613653000
7	-3.733437000	9.028650000	-5.161302000
6	-4.914395000	8.562863000	-4.437419000
6	-4.617390000	7.418771000	-3.449014000
6	-4.065448000	6.127336000	-4.019244000
6	-4.157756000	5.784878000	-5.392728000
6	-3.450837000	5.204514000	-3.125650000
6	-3.622357000	4.598588000	-5.861086000
6	-2.921664000	4.012202000	-3.569881000
6	-2.961344000	3.674175000	-4.973947000
8	-2.407719000	2.590540000	-5.417679000
1	-2.974186000	9.443474000	-4.628194000
1	-5.649354000	8.263849000	-5.188567000
1	-5.554107000	7.190397000	-2.912989000
1	-3.915416000	7.789895000	-2.688112000
1	-4.617378000	6.471834000	-6.095709000
1	-3.400597000	5.458331000	-2.069604000
1	-3.666086000	4.339878000	-6.913321000
1	-2.458720000	3.299499000	-2.894836000
1	-3.082201000	-1.243248000	-2.997888000
6	-4.536303000	6.167302000	2.619776000
6	-3.097928000	6.610448000	2.676452000
8	-2.150422000	5.777454000	2.790320000
6	-4.841194000	5.479048000	1.244853000
6	-5.618717000	4.146884000	1.375455000
6	-4.781380000	3.057105000	1.993701000
8	-4.100813000	2.248383000	1.312624000
8	-4.770252000	3.075680000	3.334671000
1	-4.690456000	5.437863000	3.421003000
1	-5.427354000	6.160605000	0.619260000
1	-3.903899000	5.284118000	0.709066000
1	-5.926793000	3.812023000	0.384157000
1	-6.519841000	4.276304000	1.978491000
7	-2.812304000	7.941710000	2.562055000
6	-1.451034000	8.383010000	2.220499000
6	-0.373505000	7.912541000	3.193696000
8	0.730762000	7.481450000	2.759025000
1	-3.571996000	8.592316000	2.417787000
1	-1.159086000	7.986035000	1.242103000
7	-0.625593000	8.016704000	4.525217000
6	0.363942000	7.602400000	5.523021000
6	0.509217000	6.079390000	5.664748000
8	1.585538000	5.590966000	6.091115000
1	-1.511340000	8.399368000	4.826152000
1	1.347888000	7.993639000	5.257071000
7	-0.580255000	5.350063000	5.335166000
6	-0.633077000	3.876066000	5.372869000
6	0.264077000	3.306624000	4.252934000
8	1.170895000	2.476487000	4.525266000
6	-2.123745000	3.491070000	5.285267000
6	-2.471643000	2.025984000	5.552057000
8	-1.959834000	1.424777000	6.537729000
8	-3.366440000	1.518019000	4.738004000
1	-1.330457000	5.808713000	4.831905000
1	-0.204061000	3.518623000	6.310565000
1	-2.525379000	3.791092000	4.316201000
1	-2.647849000	4.075717000	6.054592000
7	0.090189000	3.801669000	2.997442000
6	1.004315000	3.394509000	1.920304000
6	2.436281000	3.898291000	2.184484000
8	3.430707000	3.219999000	1.809066000
6	0.512341000	3.831014000	0.518788000
6	-0.791828000	3.162047000	-0.000461000
6	-0.740130000	1.651827000	-0.051139000
8	-0.436720000	1.024728000	1.007482000
8	-1.026042000	1.038318000	-1.192072000
1	-0.669496000	4.449619000	2.800439000
1	1.084188000	2.307894000	1.939906000
1	1.321380000	3.609722000	-0.187984000
1	0.359855000	4.916989000	0.481773000
1	-1.638298000	3.420498000	0.649441000
1	-1.007591000	3.550958000	-0.998031000
7	2.576378000	5.089479000	2.826065000
6	3.888052000	5.604838000	3.212702000
6	4.663298000	4.689931000	4.166282000
8	5.883228000	4.461195000	3.957459000

1	1.769557000	5.680481000	2.994103000
1	4.538309000	5.735949000	2.345332000
7	3.997931000	4.177496000	5.232111000
6	4.687678000	3.279803000	6.157959000
6	4.885518000	1.861117000	5.607725000
8	6.007769000	1.279620000	5.706669000
1	3.051429000	4.485596000	5.451008000
1	5.684300000	3.663344000	6.385136000
7	3.811911000	1.264686000	5.043356000
6	3.930204000	-0.077503000	4.462241000
6	2.806082000	-0.380769000	3.467155000
6	1.484691000	-0.696522000	4.095354000
7	0.345825000	-0.943311000	3.317013000
6	1.115160000	-0.783405000	5.420137000
6	-0.675958000	-1.154037000	4.166379000
7	-0.240204000	-1.071150000	5.437348000
1	2.930006000	1.768005000	4.924931000
1	3.957618000	-0.831947000	5.262222000
1	3.113524000	-1.230621000	2.842674000
1	2.700306000	0.470075000	2.783222000
1	1.673008000	-0.613348000	6.323804000
1	-1.702201000	-1.329652000	3.894615000
6	1.405982000	0.144558000	-4.229095000
6	2.223527000	-0.665971000	-5.210280000
8	2.025737000	-1.911647000	-5.383543000
6	0.117041000	0.774194000	-4.831890000
6	-0.851973000	-0.166142000	-5.563995000
6	-1.638539000	-1.174201000	-4.759151000
8	-2.041728000	-0.770431000	-3.488241000
8	-2.013390000	-2.257699000	-5.205789000
1	1.145382000	-0.521580000	-3.401001000
1	-0.417780000	1.282260000	-4.025256000
1	0.407364000	1.554473000	-5.544285000
1	-0.362228000	-0.722304000	-6.368925000
1	-1.607171000	0.487607000	-6.024024000
7	3.190117000	-0.042817000	-5.926946000
6	3.965124000	-0.752112000	-6.956688000
6	4.768057000	-1.953168000	-6.436125000
8	4.899912000	-2.971047000	-7.147749000
1	3.335331000	0.948521000	-5.798934000
1	3.300073000	-1.150653000	-7.726461000
7	5.309626000	-1.826991000	-5.189544000
6	6.043648000	-2.909689000	-4.549637000
6	5.240686000	-3.618426000	-3.456569000
8	5.810217000	-4.106883000	-2.436588000
1	5.345783000	-0.933497000	-4.706134000
1	6.312969000	-3.644030000	-5.317885000
7	3.911661000	-3.732619000	-3.668180000
6	3.029221000	-4.486404000	-2.768737000
6	1.633339000	-4.671342000	-3.376673000
8	0.969099000	-3.369608000	-3.507453000
6	0.745101000	-5.563282000	-2.514735000
1	3.504050000	-3.264295000	-4.471758000
1	2.936171000	-3.966391000	-1.807137000
1	1.719586000	-5.093470000	-4.386052000
1	1.235388000	-2.848430000	-4.323499000
1	-0.256399000	-5.615187000	-2.949315000
1	1.155911000	-6.576703000	-2.457051000
1	0.660690000	-5.167227000	-1.496054000
6	-5.232356000	-4.265901000	3.021026000
6	-6.416209000	-3.606884000	3.711819000
8	-7.490963000	-3.401482000	3.076491000
6	-5.669646000	-5.551626000	2.290725000
6	-4.575628000	-6.151869000	1.364331000
6	-4.179689000	-5.191242000	0.268467000
8	-3.289197000	-4.298830000	0.478730000
7	-4.823553000	-5.254918000	-0.911219000
1	-4.842677000	-3.543096000	2.292902000
1	-6.573796000	-5.332461000	1.712420000
1	-5.944433000	-6.318870000	3.024996000
1	-4.941229000	-7.091797000	0.935611000
1	-3.674763000	-6.378685000	1.944201000
1	-4.590055000	-4.581490000	-1.638619000
1	-5.526713000	-5.953335000	-1.095822000
7	-6.252469000	-3.238038000	5.006714000
6	-7.345626000	-2.627365000	5.763669000
6	-7.842027000	-1.276242000	5.237858000
8	-9.003531000	-0.897702000	5.507179000
1	-5.322621000	-3.284833000	5.428589000
1	-8.220099000	-3.283350000	5.762821000
7	-6.958980000	-0.545540000	4.497533000
6	-7.356393000	0.699441000	3.859158000
6	-7.465018000	0.610746000	2.336371000
8	-7.357284000	1.658620000	1.641111000
1	-5.986298000	-0.841273000	4.385880000
1	-8.338427000	0.976287000	4.260232000
7	-7.708079000	-0.616374000	1.810641000
6	-7.905829000	-0.836789000	0.379135000
6	-7.001227000	-1.939182000	-0.165439000
8	-5.589246000	-1.559020000	-0.112385000
1	-7.788659000	-1.423049000	2.428400000
1	-7.717266000	0.109299000	-0.132935000
1	-7.115182000	-2.844805000	0.437478000
1	-7.276026000	-2.162293000	-1.202809000
1	-5.138384000	-1.450825000	-0.985209000
6	-10.781725000	5.378295000	-3.123674000
6	-9.505289000	4.627603000	-2.810804000
6	-9.357928000	3.924933000	-1.602530000
6	-8.444902000	4.594781000	-3.734736000
6	-8.192037000	3.200823000	-1.321808000
6	-7.276191000	3.874919000	-3.463078000
6	-7.147160000	3.175875000	-2.255671000
1	-11.489619000	4.745653000	-3.676497000
1	-11.287504000	5.708306000	-2.209984000
1	-10.165198000	3.943240000	-0.874821000
1	-8.539353000	5.133611000	-4.673937000
1	-8.094296000	2.668661000	-0.379314000
1	-6.470797000	3.860726000	-4.190876000
1	-6.239751000	2.616526000	-2.044795000
26	0.032334000	-1.002609000	1.296097000
26	-1.537852000	-0.836869000	-1.501875000
8	0.455539000	-3.034388000	0.781076000
1	1.446536000	-3.126062000	0.758011000
1	-0.130209000	-3.657399000	1.403456000
8	-4.079970000	-0.866380000	4.022010000
1	-3.801219000	0.014181000	4.438471000
1	-4.025087000	-0.791280000	3.018119000
8	-3.931648000	-0.408839000	1.445718000
1	-4.705875000	-0.822367000	0.926919000
1	-3.948376000	0.592662000	1.409909000
8	-1.777435000	-1.240598000	0.320283000
1	-3.200574000	-3.116760000	-0.789566000
1	-2.634816000	-0.954660000	0.823394000
8	-0.841220000	-2.625033000	8.807083000
1	-0.486536000	-3.263600000	8.108481000
1	-0.929402000	-3.023840000	9.685714000
8	-3.608176000	-1.485546000	-1.929383000
8	-3.272483000	-2.961728000	-1.787362000
8	-0.109432000	-2.170094000	-1.616739000
1	0.300684000	-2.673795000	-2.437332000
8	-1.165391000	-4.280764000	2.178769000
1	-2.006990000	-4.300961000	1.657060000
1	-1.307184000	-4.291742000	3.165602000
8	-2.201637000	-1.171326000	7.278677000
1	-1.758150000	-1.589843000	8.093608000
1	-2.287603000	-0.180398000	7.225871000
8	-1.778410000	-4.442224000	4.837713000
1	-2.091043000	-5.355322000	4.981745000
1	-2.515087000	-3.726441000	5.158718000
8	-0.006413000	-4.065110000	6.816065000
1	-0.615365000	-4.209575000	6.031114000
1	0.918884000	-3.987420000	6.533608000
8	-3.419102000	-2.735953000	5.606374000
1	-3.700243000	-2.051263000	4.892941000
1	-3.058580000	-2.198028000	6.392916000
1	4.104202000	3.233048000	7.082592000
1	3.730665000	6.581001000	3.681037000
1	0.080679000	8.023236000	6.492245000
1	-1.450893000	9.477019000	2.177456000
1	-5.220981000	7.005328000	2.788507000

1	-5.345118000	9.397949000	-3.871119000
1	-2.088835000	8.744164000	-7.962360000
1	1.279243000	11.207215000	-5.355173000
1	5.704662000	6.911286000	-4.741893000
1	5.130617000	5.787614000	0.114366000
1	8.549457000	-0.534275000	-2.213924000
1	10.863366000	2.767772000	0.578004000
1	6.666944000	2.749608000	3.374675000
1	8.879861000	-1.011056000	4.556819000
1	4.642951000	-0.030169000	-7.422002000
1	6.954232000	-2.518645000	-4.093930000
1	-7.013041000	-2.506746000	6.799213000
1	-6.655351000	1.505825000	4.088383000
1	-4.424159000	-4.477861000	3.728739000
1	-8.949576000	-1.123135000	0.186825000
1	8.542909000	-3.568180000	1.050427000
1	-10.587681000	6.261470000	-3.742105000
1	2.012148000	0.959914000	-3.813754000
1	3.482547000	-5.465238000	-2.573953000
1	0.061587000	-2.648458000	-0.737350000
1	4.895802000	-0.134445000	3.949470000
1	-0.882775000	-1.150967000	6.253486000
1	-4.155541000	2.384248000	3.841623000

$^{11}Pr_{BP}$:

6	8.776302000	-2.792749000	2.270490000
6	7.983708000	-1.632589000	1.686001000
8	7.825755000	-1.556484000	0.431395000
6	7.929087000	-4.105207000	2.384473000
6	6.942832000	-4.296928000	1.275110000
7	7.268524000	-4.443242000	-0.080521000
6	5.574139000	-4.247324000	1.312339000
6	6.150014000	-4.450712000	-0.839949000
7	5.112594000	-4.341296000	0.003661000
1	9.624511000	-2.959325000	1.599092000
1	8.616998000	-4.956405000	2.456465000
1	7.361807000	-4.085317000	3.320416000
1	4.906470000	-4.140031000	2.148170000
1	6.083964000	-4.478919000	-1.931438000
1	4.108046000	-4.211332000	-0.252462000
7	7.458774000	-0.724216000	2.544918000
6	6.510547000	0.317190000	2.133147000
6	6.881357000	1.102459000	0.901878000
8	5.970273000	1.516643000	0.129631000
1	7.475415000	-0.883273000	3.549066000
1	5.517185000	-0.097955000	1.939373000
7	8.171574000	1.392935000	0.620434000
6	8.502193000	2.157877000	-0.586018000
6	7.856150000	1.609133000	-1.866415000
8	7.494050000	2.401389000	-2.772117000
1	8.904249000	1.088359000	1.243947000
1	8.166286000	3.194271000	-0.497367000
7	7.729272000	0.257697000	-1.982062000
6	7.160950000	-0.335503000	-3.191040000
6	5.652178000	-0.144300000	-3.381947000
8	5.121668000	-0.458825000	-4.478832000
1	7.931450000	-0.347421000	-1.187235000
1	7.638692000	0.092407000	-4.076661000
7	4.954073000	0.329481000	-2.321570000
6	3.525128000	0.656036000	-2.409012000
6	3.315162000	2.173809000	-2.249624000
8	2.649206000	2.833841000	-3.096413000
6	2.690026000	-0.148194000	-1.386554000
6	2.866428000	-1.666608000	-1.556303000
6	2.156735000	-2.481565000	-0.499181000
8	1.135588000	-1.928452000	0.087954000
8	2.545347000	-3.672599000	-0.208719000
1	5.453949000	0.580430000	-1.470931000
1	3.216504000	0.411295000	-3.427231000
1	1.634529000	0.112400000	-1.519126000
1	2.963191000	0.147179000	-0.366224000
1	3.927306000	-1.938825000	-1.555499000
1	2.465897000	-2.000443000	-2.526330000
7	3.896025000	2.743889000	-1.167674000
6	3.788600000	4.164982000	-0.868938000
6	4.439070000	5.104108000	-1.875877000
8	4.075248000	6.317360000	-1.892483000
1	4.423440000	2.183889000	-0.500349000
1	2.740194000	4.473262000	-0.789095000
7	5.350875000	4.620174000	-2.745953000
6	5.877907000	5.469214000	-3.815999000
6	4.799624000	6.077623000	-4.721503000
8	5.054618000	7.119888000	-5.363495000
1	5.744159000	3.688781000	-2.643598000
1	6.434940000	6.318781000	-3.411281000
7	3.587992000	5.442784000	-4.779600000
6	2.464010000	6.026894000	-5.517687000
6	1.269269000	6.279030000	-4.580820000
8	0.081672000	6.201767000	-4.996286000
6	2.058131000	5.189852000	-6.737481000
1	3.435126000	4.563212000	-4.292624000
1	2.830309000	7.003936000	-5.863167000
1	1.201841000	5.642821000	-7.242194000
1	2.898956000	5.133894000	-7.434023000
1	1.775849000	4.174886000	-6.441983000
7	1.549952000	6.635566000	-3.290778000
6	0.498028000	6.616293000	-2.264346000
6	-0.656635000	7.579730000	-2.539198000
8	-1.792076000	7.343217000	-2.043243000
6	1.080072000	6.864582000	-0.852161000
8	0.847660000	5.646049000	-0.076411000
1	2.519053000	6.622466000	-2.965827000
1	0.033394000	5.624537000	-2.240158000
1	2.152133000	7.073419000	-0.910548000
1	0.573754000	7.712624000	-0.374427000
1	1.041474000	5.773907000	0.885086000
7	-0.391902000	8.682238000	-3.280249000
6	-1.432137000	9.649485000	-3.621932000
6	-2.563500000	9.120034000	-4.514509000
8	-3.625198000	9.777122000	-4.587617000
1	0.535494000	8.782197000	-3.669861000
1	-1.918151000	10.018573000	-2.714924000
7	-2.340645000	7.953825000	-5.186131000
6	-3.394790000	7.323991000	-5.971852000
6	-3.902134000	6.003194000	-5.392627000
8	-4.590119000	5.231308000	-6.110708000
1	-1.448034000	7.466351000	-5.117284000
1	-4.229761000	8.029582000	-6.032203000
7	-3.570801000	5.735113000	-4.101275000
6	-3.938575000	4.486118000	-3.442917000
6	-2.730364000	3.572228000	-3.157179000
6	-1.926667000	3.078020000	-4.341314000
6	-2.500595000	2.854914000	-5.623848000
6	-0.555265000	2.751801000	-4.144478000
6	-1.766186000	2.261694000	-6.633190000
6	0.192697000	2.152599000	-5.134844000
6	-0.405571000	1.835216000	-6.410170000
8	0.236035000	1.152232000	-7.309280000
1	-3.035554000	6.413208000	-3.566804000
1	-4.674967000	3.980818000	-4.069385000
1	-3.115337000	2.687322000	-2.621332000
1	-2.053973000	4.086483000	-2.460688000
1	-3.515184000	3.180416000	-5.822923000
1	-0.081849000	2.978641000	-3.194582000
1	-2.196164000	2.077003000	-7.611341000
1	1.238865000	1.922699000	-4.967700000
1	-3.615333000	-4.709050000	-2.519647000
6	-4.630655000	4.703344000	2.412321000
6	-3.170734000	5.069744000	2.491736000
8	-2.272454000	4.196726000	2.672026000
6	-4.951124000	3.981441000	1.057691000
6	-5.753966000	2.667395000	1.227881000
6	-4.931794000	1.589095000	1.884381000
8	-4.227315000	0.777125000	1.234839000
8	-4.953615000	1.623141000	3.226133000
1	-4.843088000	4.016772000	3.237915000
1	-5.524907000	4.656993000	0.414534000
1	-4.020711000	3.753258000	0.523983000
1	-6.056774000	2.298806000	0.246207000
1	-6.654502000	2.837386000	1.822807000

7	-2.816386000	6.381373000	2.333950000
6	-1.424842000	6.750906000	2.042499000
6	-0.417056000	6.283610000	3.089255000
8	0.706739000	5.829263000	2.728725000
1	-3.536562000	7.054284000	2.109810000
1	-1.103689000	6.320989000	1.087898000
7	-0.728405000	6.421450000	4.402415000
6	0.220210000	6.019687000	5.449459000
6	0.356009000	4.495264000	5.608778000
8	1.463003000	3.982307000	5.902605000
1	-1.617965000	6.829799000	4.654911000
1	1.215061000	6.402994000	5.216699000
7	-0.785437000	3.785991000	5.433273000
6	-0.864913000	2.312115000	5.483256000
6	0.032204000	1.709363000	4.381634000
8	0.886764000	0.825022000	4.652276000
6	-2.362920000	1.960389000	5.352384000
6	-2.733218000	0.485723000	5.527159000
8	-2.258612000	-0.162387000	6.501547000
8	-3.592031000	0.025596000	4.649669000
1	-1.588055000	4.265113000	5.046937000
1	-0.468816000	1.946144000	6.432453000
1	-2.738487000	2.316525000	4.390476000
1	-2.892664000	2.510181000	6.143295000
7	-0.116158000	2.226177000	3.133651000
6	0.733031000	1.770487000	2.028816000
6	2.200097000	2.184267000	2.223958000
8	3.130291000	1.431151000	1.826559000
6	0.200617000	2.271576000	0.668069000
6	-1.177031000	1.697370000	0.244738000
6	-1.180801000	0.204093000	0.056115000
8	-0.926320000	-0.533118000	1.094337000
8	-1.414155000	-0.304651000	-1.106791000
1	-0.846789000	2.909651000	2.945201000
1	0.743898000	0.680272000	2.032022000
1	0.949013000	2.020616000	-0.090885000
1	0.123194000	3.364915000	0.668997000
1	-1.934321000	1.929257000	1.000006000
1	-1.483448000	2.161633000	-0.694830000
7	2.431337000	3.382933000	2.818121000
6	3.777191000	3.853228000	3.127755000
6	4.565954000	2.905078000	4.030670000
8	5.784835000	2.695020000	3.798751000
1	1.658401000	4.001383000	3.034817000
1	4.384305000	3.963980000	2.225818000
7	3.905400000	2.328554000	5.065840000
6	4.579127000	1.328179000	5.895075000
6	4.688758000	-0.048540000	5.219791000
8	5.807179000	-0.624420000	5.086516000
1	2.957431000	2.619314000	5.292510000
1	5.598921000	1.650910000	6.110667000
7	3.529249000	-0.614964000	4.800563000
6	3.547704000	-1.921286000	4.132675000
6	2.375069000	-2.107319000	3.162062000
6	1.060047000	-2.377999000	3.828946000
7	-0.135175000	-2.524349000	3.101139000
6	0.739399000	-2.505601000	5.160793000
6	-1.133213000	-2.717546000	3.992867000
7	-0.628605000	-2.719556000	5.234720000
1	2.652232000	-0.092522000	4.827461000
1	3.561079000	-2.728524000	4.879324000
1	2.616754000	-2.943042000	2.489688000
1	2.303773000	-1.213269000	2.529705000
1	1.349582000	-2.423393000	6.042319000
1	-2.182762000	-2.815992000	3.766281000
6	-0.158355000	-1.552238000	-5.408893000
6	0.942203000	-2.172081000	-6.238190000
8	1.247958000	-3.410902000	-6.110581000
6	-1.574099000	-1.756761000	-6.030064000
6	-2.203842000	-3.133496000	-5.764927000
6	-2.645912000	-3.367358000	-4.328741000
8	-2.499374000	-2.363393000	-3.481814000
8	-3.128678000	-4.484945000	-3.987303000
1	-0.132876000	-1.992619000	-4.409120000
1	-2.228140000	-0.982520000	-5.618531000

1	-1.516857000	-1.583655000	-7.111282000
1	-1.518198000	-3.947372000	-6.033951000
1	-3.089551000	-3.270861000	-6.399232000
7	1.569272000	-1.385251000	-7.131893000
6	2.491366000	-1.916141000	-8.145502000
6	3.662680000	-2.752064000	-7.623286000
8	4.048325000	-3.747290000	-8.274498000
1	1.253684000	-0.410787000	-7.220809000
1	1.959941000	-2.569443000	-8.843298000
7	4.263096000	-2.350429000	-6.462102000
6	5.367037000	-3.101576000	-5.881033000
6	4.968129000	-3.882088000	-4.631358000
8	5.802839000	-4.075163000	-3.693916000
1	4.031706000	-1.476777000	-6.003888000
1	5.719665000	-3.811625000	-6.638233000
7	3.715884000	-4.384980000	-4.610910000
6	3.232175000	-5.258260000	-3.537848000
6	1.799695000	-5.740058000	-3.798197000
8	0.894266000	-4.590153000	-3.833539000
6	1.316143000	-6.697921000	-2.713520000
1	3.073184000	-4.122986000	-5.356823000
1	3.244125000	-4.723573000	-2.580028000
1	1.749278000	-6.231737000	-4.778808000
1	0.889997000	-4.147926000	-4.742268000
1	0.274661000	-6.974398000	-2.897669000
1	1.920776000	-7.611006000	-2.702410000
1	1.382329000	-6.229234000	-1.724472000
6	-5.900367000	-6.061431000	3.566095000
6	-6.989729000	-5.107780000	4.032765000
8	-8.000063000	-4.860876000	3.315804000
6	-6.481287000	-7.281890000	2.827761000
6	-5.393820000	-8.315673000	2.424495000
6	-4.233717000	-7.648556000	1.717648000
8	-3.161205000	-7.382656000	2.357846000
7	-4.401666000	-7.275416000	0.440253000
1	-5.236138000	-5.503497000	2.891219000
1	-7.029641000	-6.935828000	1.945166000
1	-7.215139000	-7.788444000	3.465259000
1	-5.841526000	-9.086773000	1.785939000
1	-4.993677000	-8.809581000	3.313962000
1	-3.721879000	-6.657930000	-0.020706000
1	-5.248970000	-7.498292000	-0.059810000
7	-6.797729000	-4.542030000	5.254202000
6	-7.830414000	-3.735729000	5.902481000
6	-8.209036000	-2.429519000	5.201233000
8	-9.330694000	-1.916319000	5.411485000
1	-5.873627000	-4.624860000	5.681692000
1	-8.759640000	-4.306954000	5.981174000
7	-7.269565000	-1.882388000	4.378361000
6	-7.557776000	-0.701525000	3.581194000
6	-7.591092000	-0.969717000	2.077042000
8	-7.349531000	-0.030627000	1.268223000
1	-6.331660000	-2.276812000	4.301105000
1	-8.542526000	-0.332875000	3.891999000
7	-7.922793000	-2.229546000	1.694829000
6	-8.117146000	-2.612811000	0.297145000
6	-7.193661000	-3.739098000	-0.152432000
8	-5.819360000	-3.255240000	-0.241904000
1	-8.093310000	-2.940543000	2.405213000
1	-7.954830000	-1.727038000	-0.320359000
1	-7.248407000	-4.574735000	0.557158000
1	-7.511556000	-4.088001000	-1.142196000
1	-5.224922000	-3.841525000	-0.783896000
6	-7.406757000	2.913736000	-5.321425000
6	-6.946524000	1.960323000	-4.237983000
6	-7.607521000	1.887775000	-2.999117000
6	-5.841465000	1.112174000	-4.449343000
6	-7.184266000	0.998753000	-2.001532000
6	-5.410892000	0.222863000	-3.457338000
6	-6.081945000	0.164533000	-2.227820000
1	-7.667949000	2.372363000	-6.239714000
1	-8.293098000	3.473128000	-5.005379000
1	-8.467645000	2.527898000	-2.820856000
1	-5.323891000	1.145536000	-5.404820000
1	-7.705141000	0.940534000	-1.050562000

1	-4.561247000	-0.428953000	-3.643851000
1	-5.764540000	-0.527372000	-1.454919000
26	-0.445416000	-2.487203000	1.127743000
26	-1.799400000	-2.181379000	-1.708547000
8	0.144120000	-4.417625000	0.784068000
1	1.116180000	-4.479836000	0.604688000
1	-0.343110000	-5.141622000	1.480796000
8	-4.404751000	-2.319021000	3.823171000
1	-4.098357000	-1.456070000	4.250256000
1	-4.384088000	-2.240227000	2.823166000
8	-4.255880000	-1.880779000	1.191783000
1	-5.016486000	-2.336128000	0.672057000
1	-4.279360000	-0.881488000	1.163118000
8	-2.126050000	-2.863154000	0.248943000
1	-2.576349000	-4.386793000	-0.229865000
1	-2.988468000	-2.437771000	0.679624000
8	-1.555747000	-4.466647000	8.734841000
1	-1.305919000	-5.216908000	8.094394000
1	-1.880852000	-4.793425000	9.587519000
8	-4.001087000	-4.747068000	-1.560655000
8	-2.847818000	-5.237804000	-0.714326000
8	-0.393206000	-3.662382000	-1.843572000
1	0.107589000	-4.043658000	-2.651591000
8	-1.038741000	-5.869494000	2.307898000
1	-1.756234000	-6.497248000	2.012207000
1	-1.227017000	-5.683282000	3.269872000
8	-2.442878000	-2.774051000	7.127632000
1	-2.133997000	-3.294664000	7.956375000
1	-2.536290000	-1.783548000	7.148769000
8	-2.259842000	-6.005296000	4.675729000
1	-2.700010000	-6.740202000	4.189365000
1	-2.954612000	-5.291367000	4.978441000
8	-0.998528000	-6.251314000	6.970625000
1	-1.459477000	-6.246558000	6.072774000
1	-0.229460000	-6.841206000	6.985717000
8	-3.891040000	-4.222662000	5.498880000
1	-4.148815000	-3.555488000	4.780379000
1	-3.446181000	-3.705420000	6.247949000
1	4.024570000	1.232970000	6.833183000
1	3.681996000	4.831904000	3.606981000
1	-0.107161000	6.456060000	6.397685000
1	-1.378376000	7.841903000	1.960280000
1	-5.268285000	5.585273000	2.534668000
1	-4.427721000	4.712075000	-2.487775000
1	-3.054996000	7.115287000	-6.990410000
1	-0.961571000	10.501141000	-4.121191000
1	6.564200000	4.862728000	-4.413117000
1	4.253668000	4.331290000	0.107067000
1	7.358141000	-1.412196000	-3.183142000
1	9.590194000	2.158460000	-0.703131000
1	6.402162000	1.028991000	2.960990000
1	9.180778000	-2.552975000	3.258846000
1	2.880078000	-1.061673000	-8.708424000
1	6.177025000	-2.427030000	-5.609692000
1	-7.491314000	-3.506355000	6.917632000
1	-6.826889000	0.090349000	3.759763000
1	-5.290479000	-6.380701000	4.416873000
1	-9.153905000	-2.945406000	0.154464000
1	8.202995000	-4.423239000	-0.459261000
1	-6.628586000	3.640853000	-5.588090000
1	0.026047000	-0.483466000	-5.295676000
1	3.901515000	-6.123400000	-3.447088000
1	-0.345179000	-4.243676000	-1.049409000
1	4.493556000	-1.989298000	3.585934000
1	-1.245806000	-2.801984000	6.090411000
1	-4.352200000	0.930628000	3.744727000

$^{9}Re_{BP}$:

6	8.629595000	-1.412637000	3.551910000
6	8.049739000	-0.327303000	2.655255000
8	8.052912000	-0.488274000	1.398179000
6	7.673152000	-2.633639000	3.755077000
6	6.871734000	-2.999643000	2.545785000
7	7.395623000	-3.417349000	1.315049000
6	5.522742000	-2.883236000	2.338994000
6	6.410472000	-3.515344000	0.391306000
7	5.264887000	-3.197359000	1.010009000
1	9.553406000	-1.748736000	3.068705000
1	8.269830000	-3.482208000	4.111834000
1	6.962069000	-2.395805000	4.552664000
1	4.741965000	-2.590816000	3.016114000
1	6.513720000	-3.758843000	-0.667416000
1	4.315514000	-3.025454000	0.569383000
7	7.566155000	0.792135000	3.249424000
6	6.862612000	1.845142000	2.508389000
6	7.605006000	2.427415000	1.330233000
8	6.959751000	2.981478000	0.390826000
1	7.380048000	0.809524000	4.255779000
1	5.902615000	1.488125000	2.118474000
7	8.949906000	2.342352000	1.265214000
6	9.675108000	2.779623000	0.070069000
6	9.200142000	2.129063000	-1.239137000
8	9.407160000	2.717201000	-2.321604000
1	9.450797000	1.917381000	2.031380000
1	9.570543000	3.857345000	-0.076397000
7	8.579101000	0.911023000	-1.156927000
6	8.046423000	0.277239000	-2.354905000
6	6.587963000	0.611863000	-2.690791000
8	6.056133000	0.111689000	-3.720908000
1	8.450286000	0.446401000	-0.257641000
1	8.651402000	0.580911000	-3.214271000
7	5.919366000	1.421289000	-1.836490000
6	4.502869000	1.745557000	-2.037918000
6	4.191198000	3.244482000	-1.982891000
8	3.266148000	3.709541000	-2.710987000
6	3.566303000	1.036289000	-1.021603000
6	3.699040000	-0.493045000	-1.014414000
6	2.689958000	-1.157032000	-0.091028000
8	1.666540000	-0.481901000	0.273241000
8	2.890546000	-2.392021000	0.286493000
1	6.416844000	1.801811000	-1.033557000
1	4.260434000	1.412396000	-3.048735000
1	2.535592000	1.302248000	-1.276970000
1	3.739321000	1.438965000	-0.016610000
1	4.708496000	-0.803749000	-0.720142000
1	3.528342000	-0.913883000	-2.016282000
7	4.853758000	4.004610000	-1.077285000
6	4.352442000	5.307025000	-0.643435000
6	4.181619000	6.354326000	-1.726239000
8	3.282996000	7.230593000	-1.608602000
1	5.549397000	3.583674000	-0.458112000
1	3.370929000	5.208666000	-0.170445000
7	5.004049000	6.323259000	-2.803325000
6	4.805386000	7.274672000	-3.898233000
6	3.392129000	7.216350000	-4.490340000
8	2.871190000	8.248755000	-4.984943000
1	5.716902000	5.609586000	-2.863835000
1	4.954987000	8.301509000	-3.556227000
7	2.759922000	6.012520000	-4.462511000
6	1.414286000	5.868080000	-5.018559000
6	0.343898000	6.602917000	-4.203060000
8	-0.794374000	6.794596000	-4.717062000
6	1.051460000	4.386595000	-5.194263000
1	3.141105000	5.224256000	-3.938255000
1	1.395789000	6.367537000	-5.993736000
1	0.025613000	4.307135000	-5.561611000
1	1.724205000	3.922125000	-5.922821000
1	1.139379000	3.849044000	-4.244979000
7	0.671559000	7.073927000	-2.977574000
6	-0.264071000	7.912554000	-2.232205000
6	-0.708692000	9.142786000	-3.042948000
8	-1.849005000	9.636218000	-2.836681000
6	0.344014000	8.353717000	-0.896831000
8	0.262958000	7.221650000	0.020049000
1	1.589353000	6.909537000	-2.570298000
1	-1.184270000	7.358255000	-2.023159000
1	1.383685000	8.666719000	-1.033624000
1	-0.244493000	9.197393000	-0.513610000
1	0.795505000	7.363606000	0.836370000
7	0.177253000	9.661280000	-3.931415000

6	-0.160586000	10.832636000	-4.737403000
6	-1.240069000	10.620674000	-5.805343000
8	-1.732001000	11.618305000	-6.377656000
1	1.064017000	9.195231000	-4.112516000
1	-0.527842000	11.637820000	-4.095100000
7	-1.608276000	9.334675000	-6.074298000
6	-2.686726000	9.030628000	-7.002164000
6	-3.861508000	8.301856000	-6.357334000
8	-4.648312000	7.624105000	-7.069571000
1	-1.166149000	8.549590000	-5.601947000
1	-3.036353000	9.980924000	-7.421243000
7	-4.003817000	8.430585000	-5.009065000
6	-5.117416000	7.819455000	-4.285655000
6	-4.685499000	6.750841000	-3.266069000
6	-4.052611000	5.479289000	-3.784676000
6	-4.007977000	5.128107000	-5.162500000
6	-3.504801000	4.571701000	-2.827973000
6	-3.411674000	3.950423000	-5.565865000
6	-2.916912000	3.387064000	-3.205692000
6	-2.833461000	3.047338000	-4.604500000
8	-2.244500000	1.956133000	-5.004406000
1	-3.321191000	8.966620000	-4.480685000
1	-5.797364000	7.406779000	-5.035275000
1	-5.576015000	6.464927000	-2.681795000
1	-3.991737000	7.213056000	-2.547306000
1	-4.426058000	5.795699000	-5.909531000
1	-3.565860000	4.826458000	-1.773877000
1	-3.357471000	3.675744000	-6.613175000
1	-2.500056000	2.692043000	-2.483158000
1	-1.697914000	0.791872000	-4.215195000
6	-4.596425000	6.385574000	3.022757000
6	-3.130924000	6.732545000	3.000506000
8	-2.231929000	5.843158000	3.062487000
6	-5.031874000	5.775811000	1.644232000
6	-5.798050000	4.438067000	1.774886000
6	-4.909697000	3.333273000	2.286708000
8	-4.200514000	2.625550000	1.527787000
8	-4.888864000	3.226749000	3.622188000
1	-4.750300000	5.638574000	3.807567000
1	-5.669554000	6.492590000	1.116006000
1	-4.149451000	5.609558000	1.013953000
1	-6.172594000	4.138080000	0.794604000
1	-6.653510000	4.538797000	2.445515000
7	-2.770586000	8.044786000	2.863145000
6	-1.406191000	8.396500000	2.443916000
6	-0.309611000	7.887397000	3.374654000
8	0.766631000	7.433496000	2.894965000
1	-3.495225000	8.741393000	2.759572000
1	-1.185288000	7.959460000	1.463974000
7	-0.515303000	7.980376000	4.714130000
6	0.486825000	7.516318000	5.675994000
6	0.576960000	5.987392000	5.802534000
8	1.641753000	5.459056000	6.209862000
1	-1.384295000	8.375355000	5.046352000
1	1.476560000	7.870974000	5.381968000
7	-0.541963000	5.299047000	5.483188000
6	-0.644459000	3.826646000	5.513247000
6	0.206099000	3.236326000	4.368638000
8	1.114924000	2.401654000	4.617226000
6	-2.148900000	3.492110000	5.459619000
6	-2.527197000	2.025995000	5.669949000
8	-2.006589000	1.375122000	6.618772000
8	-3.448009000	1.569007000	4.854797000
1	-1.284089000	5.784542000	4.991993000
1	-0.203576000	3.448760000	6.437143000
1	-2.571667000	3.846766000	4.518604000
1	-2.629524000	4.057199000	6.270564000
7	-0.005336000	3.721701000	3.115400000
6	0.865480000	3.296080000	2.007961000
6	2.316105000	3.760520000	2.240332000
8	3.288829000	3.065665000	1.840897000
6	0.352767000	3.788959000	0.634090000
6	-0.996969000	3.200157000	0.143315000
6	-0.999062000	1.695247000	-0.047834000
8	-0.731163000	0.979197000	0.981205000
8	-1.285561000	1.189001000	-1.210950000
1	-0.766673000	4.371802000	2.937474000
1	0.904899000	2.206467000	1.999999000
1	1.128904000	3.547331000	-0.102595000
1	0.255738000	4.881968000	0.632782000
1	-1.799496000	3.429932000	0.855690000
1	-1.245817000	3.682681000	-0.804365000
7	2.494454000	4.954110000	2.871675000
6	3.823443000	5.458816000	3.206622000
6	4.627446000	4.545045000	4.136312000
8	5.839162000	4.313007000	3.885485000
1	1.701974000	5.556621000	3.063022000
1	4.442753000	5.577377000	2.315099000
7	3.998565000	4.036608000	5.225091000
6	4.710320000	3.121176000	6.115781000
6	4.857999000	1.704956000	5.544060000
8	5.973281000	1.102258000	5.587262000
1	3.062444000	4.348911000	5.479600000
1	5.722070000	3.485774000	6.303300000
7	3.750331000	1.135902000	5.020456000
6	3.820169000	-0.192191000	4.400671000
6	2.677095000	-0.436474000	3.409818000
6	1.356869000	-0.746812000	4.043424000
7	0.202545000	-0.942940000	3.273052000
6	1.004045000	-0.870646000	5.370161000
6	-0.810402000	-1.160708000	4.129291000
7	-0.356726000	-1.131835000	5.398097000
1	2.874013000	1.656919000	4.946465000
1	3.835152000	-0.969723000	5.179051000
1	2.957088000	-1.269656000	2.751764000
1	2.583287000	0.440052000	2.757448000
1	1.577061000	-0.738978000	6.270788000
1	-1.843756000	-1.301803000	3.864970000
6	1.963396000	0.372692000	-5.125623000
6	2.764044000	-0.731634000	-5.785409000
8	2.540055000	-1.956243000	-5.541733000
6	0.685985000	0.771480000	-5.915952000
6	-0.370896000	-0.342319000	-6.110332000
6	-0.845676000	-0.987980000	-4.825275000
8	-1.369297000	-0.129947000	-3.863005000
8	-0.765493000	-2.189100000	-4.580940000
1	1.684161000	0.028282000	-4.124273000
1	0.224195000	1.615476000	-5.395818000
1	0.971265000	1.137190000	-6.910524000
1	0.011608000	-1.150148000	-6.738369000
1	-1.235746000	0.107007000	-6.614052000
7	3.714885000	-0.372214000	-6.685835000
6	4.417976000	-1.353245000	-7.521744000
6	5.318301000	-2.349202000	-6.782896000
8	5.575856000	-3.447504000	-7.322494000
1	3.895728000	0.609632000	-6.837498000
1	3.696755000	-1.961612000	-8.073971000
7	5.801463000	-1.970378000	-5.567719000
6	6.607072000	-2.863160000	-4.749952000
6	5.847831000	-3.435988000	-3.553363000
8	6.473454000	-3.769915000	-2.503875000
1	5.648252000	-1.044265000	-5.178139000
1	6.939755000	-3.694668000	-5.381825000
7	4.519550000	-3.608512000	-3.708192000
6	3.677301000	-4.272333000	-2.706847000
6	2.208233000	-4.286451000	-3.139452000
8	1.708141000	-2.904525000	-3.169699000
6	1.338713000	-5.098102000	-2.184861000
1	4.075102000	-3.238336000	-4.543212000
1	3.742637000	-3.746885000	-1.748538000
1	2.124901000	-4.699127000	-4.154221000
1	1.799714000	-2.523504000	-4.083380000
1	0.294702000	-5.058180000	-2.507542000
1	1.656429000	-6.146274000	-2.168482000
1	1.403292000	-4.699465000	-1.167329000
6	-5.317503000	-4.126876000	2.921559000
6	-6.509518000	-3.491028000	3.627313000
8	-7.570840000	-3.227829000	2.993224000
6	-5.772151000	-5.172520000	1.886649000
6	-4.648157000	-5.648935000	0.924823000

6	-4.070828000	-4.503827000	0.121884000
8	-3.109760000	-3.812705000	0.609449000
7	-4.626986000	-4.196905000	-1.056761000
1	-4.767918000	-3.318370000	2.421851000
1	-6.605170000	-4.751378000	1.313901000
1	-6.167497000	-6.055068000	2.404199000
1	-5.050641000	-6.419281000	0.257138000
1	-3.828813000	-6.099605000	1.494409000
1	-4.281775000	-3.398464000	-1.605751000
1	-5.373926000	-4.757932000	-1.437896000
7	-6.351831000	-3.196816000	4.943031000
6	-7.442229000	-2.625571000	5.731855000
6	-7.905821000	-1.224239000	5.323351000
8	-9.043815000	-0.830333000	5.663538000
1	-5.421319000	-3.262476000	5.361995000
1	-8.329443000	-3.260739000	5.662674000
7	-7.028919000	-0.469478000	4.600355000
6	-7.414754000	0.827298000	4.066610000
6	-7.631093000	0.841221000	2.552582000
8	-7.592033000	1.937501000	1.929836000
1	-6.073389000	-0.784006000	4.422535000
1	-8.356861000	1.114390000	4.547569000
7	-7.899058000	-0.353034000	1.964561000
6	-8.225512000	-0.479805000	0.546793000
6	-7.194612000	-1.286562000	-0.237332000
8	-5.911945000	-0.588447000	-0.264093000
1	-7.917205000	-1.200394000	2.529628000
1	-8.307498000	0.525646000	0.130740000
1	-7.054924000	-2.270694000	0.225716000
1	-7.550448000	-1.423366000	-1.265483000
1	-5.430423000	-0.622762000	-1.128798000
6	-9.709285000	5.660738000	-3.743180000
6	-8.826460000	4.670531000	-3.014129000
6	-9.062227000	4.349393000	-1.665511000
6	-7.768945000	4.022091000	-3.678051000
6	-8.273479000	3.404361000	-0.997800000
6	-6.976531000	3.076150000	-3.016164000
6	-7.224090000	2.764426000	-1.673167000
1	-10.574941000	5.160208000	-4.197998000
1	-10.099965000	6.426924000	-3.064648000
1	-9.875671000	4.840336000	-1.137202000
1	-7.573010000	4.254293000	-4.721799000
1	-8.462029000	3.156758000	0.042188000
1	-6.168005000	2.584524000	-3.549067000
1	-6.615356000	2.031996000	-1.151394000
26	-0.140831000	-0.964051000	1.237307000
26	-1.776454000	-0.736915000	-1.830317000
8	0.338340000	-2.994547000	0.850320000
1	1.332737000	-3.032846000	0.779025000
1	-0.137864000	-3.638778000	1.515244000
8	-4.160861000	-0.762613000	3.934215000
1	-3.879614000	0.081785000	4.416179000
1	-4.135560000	-0.596284000	2.944163000
8	-4.025988000	-0.030078000	1.370534000
1	-4.861745000	-0.251631000	0.825428000
1	-3.945632000	0.962959000	1.466274000
8	-2.091860000	-1.346580000	0.354150000
1	-2.426718000	-2.293291000	0.346568000
1	-2.832554000	-0.735952000	0.768175000
8	-1.047973000	-2.657591000	8.866519000
1	-0.651764000	-3.303591000	8.196170000
1	-1.191969000	-3.045112000	9.742637000
8	-3.920048000	-0.636688000	-2.079190000
8	-3.444615000	-1.953634000	-2.313073000
8	-0.266415000	-2.100484000	-1.626462000
1	0.405953000	-2.396336000	-2.316182000
8	-1.138681000	-4.339674000	2.352827000
1	-1.977770000	-4.291212000	1.831226000
1	-1.299376000	-4.356504000	3.335333000
8	-2.322028000	-1.213334000	7.253566000
1	-1.913527000	-1.645768000	8.081340000
1	-2.369886000	-0.217115000	7.210227000
8	-1.906425000	-4.480397000	4.958765000
1	-2.266299000	-5.377029000	5.092151000
1	-2.636554000	-3.732870000	5.178530000

8	-0.152476000	-4.114812000	6.932264000
1	-0.767332000	-4.260091000	6.150573000
1	0.774471000	-4.065185000	6.650006000
8	-3.545043000	-2.685997000	5.533171000
1	-3.798363000	-2.014497000	4.810305000
1	-3.185088000	-2.150788000	6.329206000
1	4.165209000	3.077026000	7.063619000
1	3.692383000	6.440530000	3.671673000
1	0.252100000	7.938776000	6.657422000
1	-1.343934000	9.487186000	2.371425000
1	-5.208797000	7.260623000	3.265591000
1	-5.662938000	8.600762000	-3.742533000
1	-2.341132000	8.405050000	-7.830544000
1	0.749989000	11.181006000	-5.231896000
1	5.539042000	7.060912000	-4.680956000
1	5.051810000	5.685895000	0.108468000
1	8.111319000	-0.811614000	-2.256660000
1	10.736637000	2.558482000	0.214639000
1	6.629455000	2.664896000	3.201678000
1	8.897949000	-1.017198000	4.536283000
1	5.022796000	-0.803180000	-8.248722000
1	7.484764000	-2.340620000	-4.366780000
1	-7.126074000	-2.601704000	6.779194000
1	-6.668030000	1.590054000	4.299119000
1	-4.626004000	-4.568444000	3.647933000
1	-9.198603000	-0.978090000	0.443649000
1	8.376076000	-3.518240000	1.103016000
1	-9.168376000	6.166012000	-4.550659000
1	2.581335000	1.269310000	-4.994428000
1	4.035301000	-5.299130000	-2.558342000
1	-0.145772000	-2.609320000	-0.780325000
1	4.778985000	-0.258251000	3.876597000
1	-0.987570000	-1.220235000	6.220789000
1	-4.251833000	2.503490000	4.055354000

^{9}TS1$_{BP}$:

6	8.628994000	-1.391917000	3.498028000
6	8.041239000	-0.301773000	2.612257000
8	8.036430000	-0.451078000	1.353935000
6	7.664479000	-2.601688000	3.723183000
6	6.866682000	-2.985328000	2.517351000
7	7.394164000	-3.445246000	1.302880000
6	5.521856000	-2.851592000	2.295037000
6	6.414375000	-3.554393000	0.374194000
7	5.270119000	-3.198632000	0.973376000
1	9.539260000	-1.738063000	2.996760000
1	8.254759000	-3.446830000	4.098327000
1	6.952557000	-2.343668000	4.513796000
1	4.740068000	-2.528417000	2.956688000
1	6.514037000	-3.837727000	-0.673474000
1	4.326718000	-3.027552000	0.521591000
7	7.557731000	0.811297000	3.219741000
6	6.849102000	1.868843000	2.490848000
6	7.592555000	2.467023000	1.321773000
8	6.949302000	3.028608000	0.385040000
1	7.375482000	0.819334000	4.226922000
1	5.891833000	1.511533000	2.094677000
7	8.937877000	2.387635000	1.261879000
6	9.667754000	2.844705000	0.077310000
6	9.201766000	2.211496000	-1.243011000
8	9.412293000	2.815506000	-2.315999000
1	9.436427000	1.957688000	2.026901000
1	9.560439000	3.924120000	-0.054234000
7	8.584013000	0.990121000	-1.181350000
6	8.060734000	0.371627000	-2.391028000
6	6.587720000	0.663152000	-2.702489000
8	6.050702000	0.140058000	-3.718869000
1	8.451595000	0.511830000	-0.289648000
1	8.646119000	0.724764000	-3.245445000
7	5.917657000	1.471489000	-1.848596000
6	4.495893000	1.781016000	-2.039265000
6	4.172031000	3.277654000	-1.975521000
8	3.250768000	3.742032000	-2.709323000
6	3.570367000	1.054766000	-1.024733000
6	3.707869000	-0.474346000	-1.041327000

6	2.686636000	-1.162857000	-0.146192000
8	1.641177000	-0.506398000	0.194160000
8	2.901871000	-2.393693000	0.226903000
1	6.419631000	1.865911000	-1.054814000
1	4.251411000	1.451741000	-3.050925000
1	2.536638000	1.320785000	-1.267852000
1	3.749610000	1.444496000	-0.015671000
1	4.713401000	-0.786324000	-0.735898000
1	3.559029000	-0.874816000	-2.055094000
7	4.823063000	4.034096000	-1.059284000
6	4.316985000	5.333083000	-0.621245000
6	4.163127000	6.390701000	-1.696048000
8	3.267007000	7.269887000	-1.580172000
1	5.516861000	3.612458000	-0.437992000
1	3.329356000	5.231970000	-0.162270000
7	4.995939000	6.365522000	-2.765250000
6	4.810970000	7.327589000	-3.853288000
6	3.401484000	7.280266000	-4.454999000
8	2.886594000	8.318219000	-4.943698000
1	5.708981000	5.651865000	-2.823637000
1	4.963200000	8.350730000	-3.501543000
7	2.765159000	6.078018000	-4.441295000
6	1.423535000	5.941952000	-5.008122000
6	0.346394000	6.664945000	-4.190381000
8	-0.790492000	6.855851000	-4.708172000
6	1.063006000	4.463066000	-5.207409000
1	3.141396000	5.284033000	-3.922492000
1	1.411982000	6.455266000	-5.976202000
1	0.038477000	4.387674000	-5.578971000
1	1.738898000	4.009973000	-5.940228000
1	1.148892000	3.912482000	-4.265384000
7	0.666923000	7.127298000	-2.959798000
6	-0.274270000	7.957609000	-2.211482000
6	-0.717422000	9.193169000	-3.015012000
8	-1.861355000	9.680871000	-2.815294000
6	0.328523000	8.392002000	-0.871205000
8	0.247824000	7.255136000	0.039424000
1	1.583763000	6.962976000	-2.549993000
1	-1.193828000	7.399529000	-2.010175000
1	1.367730000	8.708738000	-1.003104000
1	-0.263174000	9.232163000	-0.485119000
1	0.774403000	7.396798000	0.859828000
7	0.176486000	9.723470000	-3.888623000
6	-0.152976000	10.904501000	-4.683824000
6	-1.214300000	10.703187000	-5.771413000
8	-1.693765000	11.705726000	-6.345500000
1	1.065554000	9.260444000	-4.065471000
1	-0.533271000	11.699188000	-4.035992000
7	-1.582717000	9.420029000	-6.053691000
6	-2.646735000	9.125307000	-7.000715000
6	-3.836869000	8.404237000	-6.376320000
8	-4.608642000	7.720423000	-7.099064000
1	-1.151216000	8.630738000	-5.578916000
1	-2.982558000	10.078888000	-7.423746000
7	-4.008862000	8.543869000	-5.032291000
6	-5.145539000	7.947856000	-4.331988000
6	-4.745184000	6.904625000	-3.274716000
6	-4.096622000	5.622790000	-3.744648000
6	-3.960965000	5.265278000	-5.115438000
6	-3.620236000	4.715756000	-2.749471000
6	-3.334281000	4.089619000	-5.473283000
6	-3.007909000	3.531220000	-3.083259000
6	-2.817977000	3.193912000	-4.471431000
8	-2.187734000	2.110137000	-4.830311000
1	-3.333578000	9.077321000	-4.491862000
1	-5.798836000	7.515889000	-5.094484000
1	-5.650437000	6.632774000	-2.707116000
1	-4.070245000	7.384406000	-2.548805000
1	-4.337810000	5.924014000	-5.891284000
1	-3.758404000	4.969586000	-1.702616000
1	-3.207859000	3.811946000	-6.513476000
1	-2.651380000	2.833931000	-2.332486000
1	-1.655965000	0.983596000	-4.058512000
6	-4.611207000	6.406466000	3.003040000
6	-3.146603000	6.759316000	3.000639000
8	-2.244086000	5.874144000	3.063897000
6	-5.027649000	5.806691000	1.613660000
6	-5.758234000	4.447013000	1.719999000
6	-4.846849000	3.363061000	2.237490000
8	-4.093738000	2.693014000	1.486920000
8	-4.859613000	3.232828000	3.570502000
1	-4.772574000	5.652997000	3.780135000
1	-5.682322000	6.515156000	1.095021000
1	-4.139262000	5.673414000	0.983682000
1	-6.107908000	4.143733000	0.731273000
1	-6.626963000	4.519693000	2.377267000
7	-2.791384000	8.074678000	2.876629000
6	-1.426758000	8.435433000	2.466118000
6	-0.330747000	7.923843000	3.396216000
8	0.745245000	7.470520000	2.915494000
1	-3.518523000	8.768818000	2.774222000
1	-1.201075000	8.006315000	1.483958000
7	-0.535845000	8.015616000	4.735854000
6	0.468996000	7.553754000	5.696419000
6	0.566805000	6.024752000	5.816284000
8	1.638887000	5.498297000	6.206193000
1	-1.403896000	8.411597000	5.069350000
1	1.456962000	7.914469000	5.403856000
7	-0.554457000	5.334150000	5.509652000
6	-0.652134000	3.861209000	5.531901000
6	0.203817000	3.279889000	4.387515000
8	1.115799000	2.448377000	4.632840000
6	-2.155135000	3.521109000	5.469337000
6	-2.520116000	2.047045000	5.649094000
8	-2.008464000	1.392878000	6.600422000
8	-3.414850000	1.588105000	4.807495000
1	-1.305144000	5.820949000	5.033797000
1	-0.214478000	3.478800000	6.455480000
1	-2.577800000	3.891580000	4.534268000
1	-2.639821000	4.066186000	6.291285000
7	-0.009498000	3.769715000	3.135874000
6	0.858325000	3.344572000	2.027165000
6	2.309437000	3.810928000	2.249987000
8	3.278967000	3.119831000	1.835626000
6	0.337786000	3.830833000	0.654577000
6	-1.018729000	3.246912000	0.179723000
6	-1.032016000	1.743383000	-0.001730000
8	-0.681669000	1.015853000	0.980083000
8	-1.427015000	1.247806000	-1.157931000
1	-0.772230000	4.419036000	2.960937000
1	0.900236000	2.255057000	2.022471000
1	1.107607000	3.581449000	-0.086126000
1	0.244294000	4.924001000	0.646586000
1	-1.815869000	3.481328000	0.896766000
1	-1.277781000	3.726067000	-0.766563000
7	2.490770000	4.998737000	2.890072000
6	3.820275000	5.498752000	3.230290000
6	4.623261000	4.570446000	4.146588000
8	5.832063000	4.332181000	3.887626000
1	1.697989000	5.597028000	3.092827000
1	4.438857000	5.630228000	2.340134000
7	3.995278000	4.055422000	5.232686000
6	4.704505000	3.126507000	6.111521000
6	4.855336000	1.719296000	5.518641000
8	5.970471000	1.116056000	5.559493000
1	3.060566000	4.368818000	5.490725000
1	5.715484000	3.488414000	6.308441000
7	3.751210000	1.158773000	4.979050000
6	3.825041000	-0.160463000	4.340764000
6	2.680336000	-0.397164000	3.350068000
6	1.363966000	-0.719941000	3.985165000
7	0.215875000	-0.946403000	3.214779000
6	1.010639000	-0.829059000	5.313358000
6	-0.795310000	-1.167578000	4.072342000
7	-0.345360000	-1.111215000	5.342304000
1	2.874138000	1.679466000	4.912271000
1	3.843769000	-0.948038000	5.108820000
1	2.960631000	-1.222726000	2.682684000
1	2.582734000	0.486317000	2.707119000
1	1.579857000	-0.675487000	6.212935000

1	-1.825564000	-1.331279000	3.808097000	6	-8.497301000	4.758784000	-3.149521000
6	1.912083000	0.424069000	-5.072874000	6	-8.588693000	4.353685000	-1.805814000
6	2.688312000	-0.689053000	-5.746533000	6	-7.666918000	4.020262000	-4.012553000
8	2.419817000	-1.912106000	-5.539774000	6	-7.881680000	3.239706000	-1.334286000
6	0.638854000	0.854619000	-5.853234000	6	-6.957626000	2.906247000	-3.547583000
6	-0.455627000	-0.224833000	-6.024821000	6	-7.060418000	2.512490000	-2.207655000
6	-0.937869000	-0.848217000	-4.733956000	1	-10.270608000	5.600771000	-4.069259000
8	-1.334760000	0.034480000	-3.728161000	1	-9.517285000	6.654599000	-2.871022000
8	-0.970342000	-2.054841000	-4.507278000	1	-9.230901000	4.909247000	-1.126725000
1	1.631150000	0.077314000	-4.072599000	1	-7.586427000	4.314588000	-5.055926000
1	0.209701000	1.717915000	-5.337458000	1	-7.966449000	2.927332000	-0.297226000
1	0.924686000	1.200987000	-6.854430000	1	-6.327433000	2.346972000	-4.233531000
1	-0.112532000	-1.049404000	-6.653962000	1	-6.513914000	1.650350000	-1.836591000
1	-1.313437000	0.249367000	-6.518425000	26	-0.150057000	-1.019143000	1.180075000
7	3.667228000	-0.337586000	-6.618071000	26	-1.647162000	-0.664501000	-1.718630000
6	4.363412000	-1.319528000	-7.458841000	8	0.332947000	-3.051727000	0.760831000
6	5.221205000	-2.352073000	-6.719290000	1	1.324911000	-3.091662000	0.707739000
8	5.449612000	-3.452808000	-7.266345000	1	-0.156825000	-3.705852000	1.435394000
1	3.885640000	0.641222000	-6.736374000	8	-4.133152000	-0.758035000	3.872075000
1	3.639173000	-1.897940000	-8.038329000	1	-3.845794000	0.083102000	4.349659000
7	5.701929000	-2.001137000	-5.494098000	1	-4.113534000	-0.594378000	2.879952000
6	6.477368000	-2.927630000	-4.684025000	8	-3.995704000	0.010008000	1.324952000
6	5.685492000	-3.539106000	-3.528022000	1	-4.776568000	-0.203123000	0.714701000
8	6.286195000	-3.950291000	-2.492825000	1	-3.905109000	0.998462000	1.420339000
1	5.587951000	-1.068728000	-5.104462000	8	-1.992767000	-1.333958000	0.281015000
1	6.824980000	-3.740580000	-5.331729000	1	-2.729876000	-2.257991000	-0.302813000
7	4.354091000	-3.658947000	-3.702906000	1	-2.697359000	-0.749568000	0.738895000
6	3.482529000	-4.343757000	-2.740502000	8	-1.009112000	-2.631764000	8.828113000
6	2.028970000	-4.350672000	-3.220656000	1	-0.601729000	-3.271385000	8.156690000
8	1.531693000	-2.965643000	-3.242011000	1	-1.169911000	-3.031329000	9.695979000
6	1.126186000	-5.177848000	-2.311391000	8	-3.490661000	-1.384847000	-1.943610000
1	3.932514000	-3.243152000	-4.527605000	8	-3.491734000	-2.582586000	-1.126675000
1	3.519308000	-3.837655000	-1.769864000	8	-0.300201000	-2.129602000	-1.640901000
1	1.977551000	-4.740467000	-4.246342000	1	0.339092000	-2.460861000	-2.361909000
1	1.662039000	-2.549287000	-4.135431000	8	-0.996647000	-4.475863000	2.295278000
1	0.091501000	-5.123368000	-2.660679000	1	-1.825501000	-4.720768000	1.805624000
1	1.436822000	-6.228105000	-2.313772000	1	-1.166660000	-4.365801000	3.266636000
1	1.162422000	-4.809279000	-1.281014000	8	-2.296536000	-1.201305000	7.209463000
6	-5.420404000	-4.327936000	3.087596000	1	-1.883839000	-1.632947000	8.035617000
6	-6.545447000	-3.530378000	3.730674000	1	-2.351870000	-0.205425000	7.171423000
8	-7.587490000	-3.242076000	3.075147000	8	-1.824234000	-4.435964000	4.918675000
6	-5.983116000	-5.536669000	2.311995000	1	-2.175590000	-5.340419000	5.017902000
6	-4.923053000	-6.281548000	1.457566000	1	-2.573988000	-3.711800000	5.123366000
6	-4.237583000	-5.334720000	0.493023000	8	-0.093331000	-4.077618000	6.899723000
8	-3.136641000	-4.781999000	0.779497000	1	-0.707715000	-4.221293000	6.115190000
7	-4.887154000	-5.020389000	-0.648976000	1	0.833398000	-4.020909000	6.618290000
1	-4.889647000	-3.657471000	2.399095000	8	-3.523065000	-2.681048000	5.488438000
1	-6.800792000	-5.181867000	1.675181000	1	-3.783450000	-2.019922000	4.760401000
1	-6.424055000	-6.253288000	3.015963000	1	-3.161126000	-2.139969000	6.277502000
1	-5.407784000	-7.102845000	0.915883000	1	4.156114000	3.067465000	7.056596000
1	-4.150676000	-6.716394000	2.099148000	1	3.689752000	6.473379000	3.709989000
1	-4.501860000	-4.272074000	-1.222379000	1	0.231294000	7.971545000	6.679120000
1	-5.759716000	-5.454438000	-0.904874000	1	-1.368951000	9.526899000	2.402196000
7	-6.363453000	-3.147440000	5.020915000	1	-5.228950000	7.277855000	3.245266000
6	-7.425332000	-2.488380000	5.779582000	1	-5.710570000	8.740194000	-3.825935000
6	-7.875785000	-1.119666000	5.264705000	1	-2.291499000	8.498487000	-7.823921000
8	-9.013711000	-0.693724000	5.565446000	1	0.763713000	11.263355000	-5.159045000
1	-5.431496000	-3.227484000	5.433315000	1	5.549072000	7.116512000	-4.632545000
1	-8.325002000	-3.109955000	5.785778000	1	5.006125000	5.703686000	0.143949000
7	-6.984181000	-0.422852000	4.503955000	1	8.169769000	-0.715844000	-2.328780000
6	-7.350938000	0.841809000	3.888834000	1	10.729270000	2.624939000	0.224011000
6	-7.504905000	0.779507000	2.369408000	1	6.611784000	2.679789000	3.193069000
8	-7.454267000	1.847244000	1.699387000	1	8.920698000	-0.997213000	4.476143000
1	-6.030106000	-0.757337000	4.353678000	1	4.997678000	-0.770474000	-8.161059000
1	-8.313842000	1.146378000	4.315291000	1	7.346133000	-2.421688000	-4.259636000
7	-7.735080000	-0.445000000	1.828088000	1	-7.083540000	-2.381288000	6.813834000
6	-8.039306000	-0.641358000	0.412384000	1	-6.619862000	1.621516000	4.114656000
6	-6.971055000	-1.417077000	-0.351887000	1	-4.690149000	-4.649413000	3.837794000
8	-5.768679000	-0.592255000	-0.495400000	1	-8.983492000	-1.194537000	0.327603000
1	-7.780505000	-1.262428000	2.436157000	1	8.373428000	-3.583473000	1.106872000
1	-8.173797000	0.342006000	-0.042414000	1	-8.782878000	6.457493000	-4.473888000
1	-6.720248000	-2.348144000	0.171523000	1	2.546677000	1.308139000	-4.937329000
1	-7.355353000	-1.661717000	-1.349671000	1	3.838453000	-5.372312000	-2.603152000
1	-5.185061000	-0.897959000	-1.228199000	1	-0.168470000	-2.639376000	-0.781396000
6	-9.303916000	5.931573000	-3.665887000	1	4.783206000	-0.215558000	3.814202000

1	-0.975558000	-1.200147000	6.166184000
1	-4.213656000	2.515798000	4.009948000

^{9}IM1$_{BP}$:

6	8.655762000	-1.389934000	3.537913000
6	8.068565000	-0.294438000	2.658920000
8	8.075086000	-0.431261000	1.399198000
6	7.696844000	-2.606613000	3.746029000
6	6.911669000	-2.994813000	2.533041000
7	7.451483000	-3.449012000	1.321595000
6	5.564840000	-2.889425000	2.306749000
6	6.475446000	-3.584495000	0.391773000
7	5.322966000	-3.250220000	0.986878000
1	9.571241000	-1.726397000	3.039513000
1	8.289511000	-3.449404000	4.123052000
1	6.975808000	-2.358545000	4.531636000
1	4.774949000	-2.578435000	2.965001000
1	6.577738000	-3.871764000	-0.653851000
1	4.375904000	-3.102314000	0.530761000
7	7.572204000	0.809470000	3.272683000
6	6.865609000	1.868940000	2.544718000
6	7.621558000	2.477716000	1.389232000
8	6.989622000	3.022581000	0.435931000
1	7.385418000	0.813422000	4.279021000
1	5.914960000	1.509911000	2.135081000
7	8.969576000	2.426271000	1.362997000
6	9.719524000	2.907329000	0.200755000
6	9.298870000	2.277281000	-1.135676000
8	9.523611000	2.895729000	-2.197467000
1	9.457962000	2.011451000	2.142914000
1	9.594742000	3.985666000	0.075351000
7	8.706721000	1.042177000	-1.100480000
6	8.226592000	0.425416000	-2.328426000
6	6.748430000	0.662749000	-2.659501000
8	6.256459000	0.140979000	-3.698233000
1	8.557727000	0.554568000	-0.216759000
1	8.809905000	0.819389000	-3.166178000
7	6.029399000	1.424604000	-1.802495000
6	4.603510000	1.696324000	-2.020291000
6	4.246491000	3.188455000	-1.983367000
8	3.326215000	3.624167000	-2.736222000
6	3.678194000	0.966809000	-1.008431000
6	3.811673000	-0.563979000	-1.018786000
6	2.777828000	-1.239821000	-0.124517000
8	1.761280000	-0.550304000	0.234038000
8	2.949991000	-2.481836000	0.231766000
1	6.501605000	1.823923000	-0.993292000
1	4.383555000	1.346725000	-3.030901000
1	2.644738000	1.231756000	-1.252762000
1	3.853989000	1.359470000	0.000141000
1	4.814878000	-0.879141000	-0.708197000
1	3.663112000	-0.968250000	-2.031102000
7	4.873963000	3.967516000	-1.069538000
6	4.341078000	5.258076000	-0.638549000
6	4.184202000	6.315900000	-1.711277000
8	3.285793000	7.192902000	-1.592997000
1	5.566607000	3.563005000	-0.436255000
1	3.351332000	5.139020000	-0.190152000
7	5.014137000	6.291318000	-2.782467000
6	4.821990000	7.247592000	-3.873642000
6	3.408751000	7.195284000	-4.465097000
8	2.892183000	8.229277000	-4.960958000
1	5.729028000	5.579486000	-2.840292000
1	4.975170000	8.272922000	-3.528537000
7	2.769072000	5.995388000	-4.432883000
6	1.422420000	5.859445000	-4.987755000
6	0.356362000	6.610994000	-4.181555000
8	-0.779545000	6.801995000	-4.701468000
6	1.045754000	4.380774000	-5.150244000
1	3.152910000	5.201487000	-3.919119000
1	1.408757000	6.350238000	-5.967538000
1	0.019451000	4.308166000	-5.517452000
1	1.714278000	3.902176000	-5.873318000
1	1.127927000	3.851555000	-4.195709000
7	0.682300000	7.095685000	-2.961162000
6	-0.250838000	7.955965000	-2.236005000
6	-0.677897000	9.176086000	-3.072069000
8	-1.816844000	9.680758000	-2.887587000
6	0.350838000	8.413728000	-0.902968000
8	0.237406000	7.303352000	0.036710000
1	1.598095000	6.931989000	-2.547702000
1	-1.177837000	7.414207000	-2.024690000
1	1.397945000	8.704455000	-1.034194000
1	-0.225034000	9.276231000	-0.543620000
1	0.767860000	7.447887000	0.853933000
7	0.222636000	9.674086000	-3.958062000
6	-0.096668000	10.835470000	-4.785653000
6	-1.148829000	10.610337000	-5.877862000
8	-1.615343000	11.599425000	-6.485167000
1	1.107572000	9.198383000	-4.123513000
1	-0.480399000	11.646853000	-4.161010000
7	-1.523420000	9.322201000	-6.127455000
6	-2.578010000	9.006299000	-7.078241000
6	-3.780683000	8.311205000	-6.448112000
8	-4.552607000	7.618173000	-7.161741000
1	-1.103442000	8.543793000	-5.625145000
1	-2.903054000	9.948943000	-7.532989000
7	-3.964498000	8.487152000	-5.110103000
6	-5.115981000	7.924931000	-4.406466000
6	-4.741787000	6.897016000	-3.324732000
6	-4.116614000	5.591882000	-3.762914000
6	-4.005388000	5.188897000	-5.122822000
6	-3.644873000	4.707904000	-2.745667000
6	-3.411664000	3.986747000	-5.450507000
6	-3.065626000	3.498479000	-3.048864000
6	-2.903625000	3.109667000	-4.427289000
8	-2.305009000	2.000276000	-4.756362000
1	-3.288199000	9.026487000	-4.577273000
1	-5.771437000	7.489082000	-5.164770000
1	-5.656277000	6.655280000	-2.757926000
1	-4.063007000	7.379097000	-2.604296000
1	-4.371311000	5.834431000	-5.915180000
1	-3.760639000	5.000887000	-1.706277000
1	-3.305345000	3.673790000	-6.482963000
1	-2.714620000	2.816617000	-2.282378000
1	-1.754878000	0.847437000	-3.958323000
6	-4.599739000	6.367847000	3.021132000
6	-3.142211000	6.747869000	3.008654000
8	-2.224992000	5.877591000	3.070658000
6	-5.008168000	5.734729000	1.644693000
6	-5.745759000	4.381726000	1.781155000
6	-4.841268000	3.304492000	2.324496000
8	-4.089392000	2.613227000	1.591755000
8	-4.858898000	3.203352000	3.660582000
1	-4.744232000	5.624748000	3.811710000
1	-5.655897000	6.432401000	1.103141000
1	-4.115477000	5.581614000	1.025493000
1	-6.096557000	4.058813000	0.799137000
1	-6.615447000	4.471980000	2.434575000
7	-2.809276000	8.068120000	2.881186000
6	-1.450735000	8.453732000	2.471645000
6	-0.346284000	7.950659000	3.396840000
8	0.734450000	7.513207000	2.912291000
1	-3.548263000	8.749812000	2.780033000
1	-1.219177000	8.039335000	1.484292000
7	-0.549352000	8.033874000	4.737576000
6	0.463671000	7.581049000	5.693877000
6	0.580056000	6.053078000	5.811006000
8	1.659135000	5.539669000	6.199654000
1	-1.421546000	8.417173000	5.075001000
1	1.446448000	7.953616000	5.398677000
7	-0.532623000	5.348874000	5.505309000
6	-0.610031000	3.874526000	5.523348000
6	0.234061000	3.309789000	4.360919000
8	1.148052000	2.474681000	4.588072000
6	-2.108870000	3.514641000	5.490369000
6	-2.466934000	2.043835000	5.713607000
8	-1.920467000	1.397716000	6.651363000
8	-3.403701000	1.580779000	4.921299000
1	-1.289896000	5.826437000	5.030250000

1	-0.149179000	3.495826000	6.437168000
1	-2.548814000	3.857673000	4.552689000
1	-2.588508000	4.076540000	6.304259000
7	0.011844000	3.817176000	3.117722000
6	0.881343000	3.415487000	2.000797000
6	2.329307000	3.889455000	2.225486000
8	3.300084000	3.206605000	1.800389000
6	0.354257000	3.898573000	0.627562000
6	-0.975540000	3.275121000	0.122465000
6	-0.938217000	1.768731000	-0.040334000
8	-0.492118000	1.087274000	0.931463000
8	-1.380702000	1.227999000	-1.163077000
1	-0.753560000	4.467183000	2.956451000
1	0.937642000	2.327773000	1.987702000
1	1.140180000	3.677825000	-0.105615000
1	0.228066000	4.988288000	0.625967000
1	-1.796951000	3.490262000	0.819705000
1	-1.227303000	3.740062000	-0.833088000
7	2.510345000	5.068140000	2.882343000
6	3.841826000	5.559630000	3.228211000
6	4.640178000	4.618304000	4.135997000
8	5.846510000	4.371269000	3.873819000
1	1.718930000	5.667875000	3.086750000
1	4.461175000	5.699586000	2.339839000
7	4.010529000	4.105685000	5.222724000
6	4.719256000	3.185113000	6.110223000
6	4.872596000	1.770283000	5.537178000
8	5.985934000	1.165591000	5.602113000
1	3.077369000	4.423988000	5.480659000
1	5.730122000	3.549209000	6.304314000
7	3.774392000	1.204038000	4.991754000
6	3.851624000	-0.128750000	4.382490000
6	2.726588000	-0.377661000	3.372530000
6	1.397711000	-0.695420000	3.984306000
7	0.265496000	-0.924779000	3.193079000
6	1.018604000	-0.797524000	5.306724000
6	-0.762971000	-1.140325000	4.030790000
7	-0.338653000	-1.076791000	5.309230000
1	2.898749000	1.724991000	4.906705000
1	3.849067000	-0.900604000	5.166509000
1	3.021751000	-1.209773000	2.719731000
1	2.638372000	0.497876000	2.717589000
1	1.567673000	-0.636032000	6.217541000
1	-1.787044000	-1.309447000	3.747956000
6	1.867172000	0.413100000	-4.946655000
6	2.663398000	-0.655223000	-5.666943000
8	2.390769000	-1.888561000	-5.536821000
6	0.578522000	0.843021000	-5.703083000
6	-0.490538000	-0.251635000	-5.926972000
6	-0.989225000	-0.934452000	-4.670519000
8	-1.425050000	-0.090655000	-3.641820000
8	-1.007344000	-2.148391000	-4.499426000
1	1.598663000	0.019741000	-3.959887000
1	0.130402000	1.670217000	-5.146383000
1	0.852678000	1.244170000	-6.687058000
1	-0.120764000	-1.046410000	-6.578820000
1	-1.348697000	0.224383000	-6.418049000
7	3.666218000	-0.259938000	-6.490789000
6	4.385279000	-1.203438000	-7.357449000
6	5.192741000	-2.291611000	-6.639683000
8	5.378184000	-3.388059000	-7.211151000
1	3.886956000	0.723295000	-6.556148000
1	3.679745000	-1.736281000	-7.999930000
7	5.683629000	-1.989739000	-5.405369000
6	6.432882000	-2.959409000	-4.620093000
6	5.619650000	-3.593918000	-3.490655000
8	6.197198000	-4.029699000	-2.452321000
1	5.623526000	-1.056728000	-5.005108000
1	6.772089000	-3.757243000	-5.290739000
7	4.291533000	-3.704162000	-3.693399000
6	3.393418000	-4.391030000	-2.757183000
6	1.941744000	-4.312221000	-3.236221000
8	1.521899000	-2.901732000	-3.234006000
6	0.994401000	-5.110616000	-2.347153000
1	3.890237000	-3.277085000	-4.522086000

1	3.457393000	-3.926585000	-1.767705000
1	1.871047000	-4.678450000	-4.269553000
1	1.653033000	-2.497974000	-4.134624000
1	-0.033195000	-4.998489000	-2.703752000
1	1.254833000	-6.174443000	-2.366257000
1	1.041679000	-4.761324000	-1.310759000
6	-5.356074000	-4.164428000	2.943373000
6	-6.519700000	-3.505476000	3.671353000
8	-7.596147000	-3.252964000	3.057634000
6	-5.848152000	-5.343947000	2.081426000
6	-4.788610000	-5.892487000	1.084902000
6	-4.399484000	-4.863634000	0.050798000
8	-3.548203000	-3.953953000	0.333333000
7	-5.006012000	-4.885698000	-1.151868000
1	-4.898899000	-3.401209000	2.300600000
1	-6.745246000	-5.025007000	1.539612000
1	-6.153805000	-6.172359000	2.732332000
1	-5.182364000	-6.792791000	0.600008000
1	-3.877048000	-6.175824000	1.622645000
1	-4.761700000	-4.180881000	-1.844413000
1	-5.681044000	-5.594279000	-1.393504000
7	-6.331513000	-3.191615000	4.977785000
6	-7.404987000	-2.605437000	5.779921000
6	-7.893681000	-1.223042000	5.336041000
8	-9.041733000	-0.843224000	5.658682000
1	-5.393339000	-3.255842000	5.379719000
1	-8.287656000	-3.250168000	5.758388000
7	-7.022474000	-0.468252000	4.607347000
6	-7.422584000	0.811110000	4.043267000
6	-7.598813000	0.797483000	2.523743000
8	-7.545116000	1.883597000	1.884138000
1	-6.056370000	-0.766556000	4.451711000
1	-8.382221000	1.085323000	4.496457000
7	-7.850684000	-0.407304000	1.950388000
6	-8.135894000	-0.561984000	0.526175000
6	-7.068651000	-1.355667000	-0.223196000
8	-5.806946000	-0.626232000	-0.215321000
1	-7.885004000	-1.244022000	2.530781000
1	-8.227849000	0.435487000	0.093374000
1	-6.929439000	-2.336884000	0.249309000
1	-7.395697000	-1.503881000	-1.259771000
1	-5.219362000	-0.775695000	-1.005093000
6	-9.507369000	5.629750000	-3.803862000
6	-8.664206000	4.583898000	-3.105420000
6	-8.857560000	4.294375000	-1.742483000
6	-7.692447000	3.851280000	-3.810669000
6	-8.110412000	3.299111000	-1.100702000
6	-6.941739000	2.855020000	-3.174458000
6	-7.145267000	2.576221000	-1.817477000
1	-10.467190000	5.209763000	-4.134006000
1	-9.736713000	6.471450000	-3.140642000
1	-9.607710000	4.848312000	-1.183336000
1	-7.531463000	4.057980000	-4.865599000
1	-8.270251000	3.072029000	-0.050994000
1	-6.200387000	2.297073000	-3.739444000
1	-6.567491000	1.805384000	-1.316003000
26	-0.067461000	-0.953767000	1.147952000
26	-1.818392000	-0.695617000	-1.597724000
8	0.300931000	-3.029248000	0.716089000
1	1.283490000	-3.154665000	0.703966000
1	-0.304135000	-3.635377000	1.319797000
8	-4.165555000	-0.752819000	4.035159000
1	-3.869031000	0.087679000	4.510350000
1	-4.116495000	-0.601661000	3.036910000
8	-3.989603000	-0.082571000	1.510024000
1	-4.803247000	-0.303295000	0.937705000
1	-3.888242000	0.907907000	1.566040000
8	-1.897487000	-1.154692000	0.283751000
1	-3.396488000	-2.749095000	-0.890170000
1	-2.697437000	-0.791990000	0.795372000
8	-0.739019000	-2.732607000	8.597938000
1	-0.413003000	-3.336344000	7.854206000
1	-0.739276000	-3.156472000	9.469127000
8	-3.732693000	-0.980138000	-1.856430000
8	-3.436430000	-2.474315000	-1.866687000

8	-0.300917000	-2.006711000	-1.640847000
1	0.325891000	-2.334938000	-2.369873000
8	-1.411917000	-4.240324000	2.053063000
1	-2.243887000	-4.113189000	1.534629000
1	-1.554020000	-4.282131000	3.038970000
8	-2.228659000	-1.224662000	7.241676000
1	-1.723069000	-1.685535000	7.994819000
1	-2.280214000	-0.229064000	7.241048000
8	-1.948293000	-4.446232000	4.718348000
1	-2.271021000	-5.352947000	4.877012000
1	-2.651264000	-3.712638000	5.068279000
8	0.012401000	-4.057300000	6.498522000
1	-0.679360000	-4.225684000	5.789638000
1	0.843901000	-3.751658000	6.099755000
8	-3.513190000	-2.696828000	5.542504000
1	-3.787507000	-1.988659000	4.851292000
1	-3.127304000	-2.189848000	6.338564000
1	4.170759000	3.136411000	7.055956000
1	3.714974000	6.528862000	3.719818000
1	0.224669000	7.994282000	6.678203000
1	-1.410342000	9.546624000	2.418868000
1	-5.234574000	7.230451000	3.250207000
1	-5.670534000	8.737352000	-3.921086000
1	-2.216520000	8.353648000	-7.878369000
1	0.824983000	11.180075000	-5.261824000
1	5.555352000	7.033776000	-4.656619000
1	5.013249000	5.637240000	0.137325000
1	8.380609000	-0.657488000	-2.287347000
1	10.781167000	2.706492000	0.371934000
1	6.619333000	2.675160000	3.248972000
1	8.939113000	-1.003355000	4.521793000
1	5.058178000	-0.624060000	-7.996712000
1	7.304856000	-2.480777000	-4.171627000
1	-7.056545000	-2.543552000	6.815504000
1	-6.697823000	1.593489000	4.280950000
1	-4.582767000	-4.490521000	3.646849000
1	-9.095342000	-1.082003000	0.406366000
1	8.434031000	-3.567722000	1.129262000
1	-9.004837000	6.024773000	-4.693238000
1	2.479699000	1.306901000	-4.776573000
1	3.701885000	-5.439894000	-2.662709000
1	-0.180334000	-2.551451000	-0.802966000
1	4.819983000	-0.202372000	3.877473000
1	-0.976667000	-1.169584000	6.125802000
1	-4.215257000	2.495853000	4.113818000

^{9}TS2$_{BP}$:

6	8.637129000	-1.361434000	3.546300000
6	8.022415000	-0.248145000	2.708922000
8	7.988658000	-0.360292000	1.447049000
6	7.745685000	-2.639647000	3.667629000
6	6.991624000	-3.010248000	2.429568000
7	7.553857000	-3.446700000	1.221214000
6	5.647283000	-2.918452000	2.185416000
6	6.591294000	-3.586404000	0.276968000
7	5.428572000	-3.270297000	0.859932000
1	9.581351000	-1.623607000	3.055814000
1	8.380936000	-3.467605000	4.005963000
1	7.004266000	-2.479141000	4.457154000
1	4.843778000	-2.621787000	2.833931000
1	6.696309000	-3.869222000	-0.766930000
1	4.486207000	-3.134121000	0.405977000
7	7.564738000	0.854236000	3.354160000
6	6.889768000	1.952739000	2.652672000
6	7.674072000	2.563963000	1.516199000
8	7.071705000	3.132901000	0.558465000
1	7.395982000	0.847086000	4.363165000
1	5.929357000	1.636479000	2.231798000
7	9.022168000	2.486715000	1.516983000
6	9.806406000	2.975938000	0.381011000
6	9.417964000	2.361401000	-0.971812000
8	9.688406000	2.981126000	-2.021887000
1	9.486729000	2.064201000	2.307463000
1	9.690503000	4.056261000	0.263912000
7	8.800154000	1.138546000	-0.959805000
6	8.330091000	0.538273000	-2.198458000
6	6.835427000	0.704673000	-2.487625000
8	6.333586000	0.108071000	-3.479976000
1	8.610591000	0.651468000	-0.084060000
1	8.874843000	1.001415000	-3.027758000
7	6.118885000	1.502656000	-1.661002000
6	4.690782000	1.758305000	-1.884849000
6	4.323316000	3.246804000	-1.875489000
8	3.380953000	3.653575000	-2.616311000
6	3.758296000	1.035847000	-0.874547000
6	3.940565000	-0.487172000	-0.842677000
6	2.861198000	-1.199289000	-0.035346000
8	1.829438000	-0.529444000	0.325230000
8	3.019886000	-2.461264000	0.250696000
1	6.595520000	1.942662000	-0.875832000
1	4.477381000	1.392955000	-2.891812000
1	2.726534000	1.264013000	-1.160134000
1	3.892984000	1.462463000	0.125892000
1	4.921036000	-0.755439000	-0.430014000
1	3.918533000	-0.907872000	-1.858514000
7	4.970001000	4.056817000	-1.002850000
6	4.452310000	5.372878000	-0.637245000
6	4.309162000	6.371271000	-1.768731000
8	3.427531000	7.268437000	-1.697690000
1	5.658012000	3.671845000	-0.352749000
1	3.459240000	5.291938000	-0.186293000
7	5.137344000	6.270749000	-2.837067000
6	4.973909000	7.178131000	-3.974129000
6	3.563984000	7.136283000	-4.574212000
8	3.076431000	8.166179000	-5.105980000
1	5.842729000	5.547500000	-2.852211000
1	5.150862000	8.214595000	-3.676866000
7	2.895718000	5.953482000	-4.513886000
6	1.549263000	5.831860000	-5.081862000
6	0.511408000	6.683335000	-4.338948000
8	-0.577140000	6.967802000	-4.908137000
6	1.118670000	4.359152000	-5.139541000
1	3.256103000	5.170453000	-3.970260000
1	1.566163000	6.249978000	-6.094003000
1	0.097707000	4.284971000	-5.520318000
1	1.783210000	3.803981000	-5.810937000
1	1.161544000	3.899721000	-4.147054000
7	0.820916000	7.134331000	-3.098428000
6	-0.080650000	8.037797000	-2.390049000
6	-0.394835000	9.307963000	-3.200964000
8	-1.489388000	9.901974000	-3.008053000
6	0.498928000	8.427910000	-1.027492000
8	0.353650000	7.281207000	-0.133772000
1	1.713909000	6.919993000	-2.663121000
1	-1.048500000	7.552090000	-2.230276000
1	1.552674000	8.705874000	-1.125850000
1	-0.072605000	9.283579000	-0.645971000
1	0.841678000	7.411177000	0.711871000
7	0.550490000	9.749650000	-4.067393000
6	0.338508000	10.953334000	-4.868122000
6	-0.750702000	10.859247000	-5.942283000
8	-1.140913000	11.908763000	-6.500741000
1	1.390698000	9.202601000	-4.243943000
1	0.051967000	11.789145000	-4.223827000
7	-1.239641000	9.619122000	-6.232538000
6	-2.345624000	9.442572000	-7.161552000
6	-3.624487000	8.915134000	-6.516107000
8	-4.520867000	8.404267000	-7.236504000
1	-0.870832000	8.787429000	-5.777304000
1	-2.551776000	10.417997000	-7.615214000
7	-3.730114000	9.032175000	-5.163179000
6	-4.911737000	8.567537000	-4.439640000
6	-4.616032000	7.423653000	-3.450608000
6	-4.064435000	6.131687000	-4.020015000
6	-4.155870000	5.788898000	-5.393467000
6	-3.451171000	5.208706000	-3.125680000
6	-3.621029000	4.602042000	-5.861057000
6	-2.922586000	4.015858000	-3.569145000
6	-2.961413000	3.677392000	-4.973161000
8	-2.408318000	2.593179000	-5.416181000

1	-2.970626000	9.446346000	-4.629905000
1	-5.646572000	8.268778000	-5.191008000
1	-5.553258000	7.196029000	-2.915166000
1	-3.914375000	7.794664000	-2.689370000
1	-4.614231000	6.476075000	-6.097049000
1	-3.401497000	5.462805000	-2.069683000
1	-3.664139000	4.343114000	-6.913264000
1	-2.460624000	3.303099000	-2.893490000
1	-3.092670000	-1.246487000	-2.990285000
6	-4.535898000	6.165172000	2.618479000
6	-3.097686000	6.608858000	2.675249000
8	-2.149956000	5.776162000	2.789365000
6	-4.840363000	5.476572000	1.243668000
6	-5.618704000	4.144874000	1.374197000
6	-4.781984000	3.054391000	1.992086000
8	-4.104185000	2.243679000	1.310672000
8	-4.768373000	3.074428000	3.333002000
1	-4.689812000	5.435746000	3.419764000
1	-5.425741000	6.158229000	0.617452000
1	-3.902837000	5.280951000	0.708549000
1	-5.927177000	3.810405000	0.382899000
1	-6.519669000	4.274790000	1.977406000
7	-2.812428000	7.940219000	2.561088000
6	-1.451191000	8.381949000	2.219882000
6	-0.373706000	7.911521000	3.193197000
8	0.730741000	7.480727000	2.758693000
1	-3.572272000	8.590649000	2.416806000
1	-1.158933000	7.985269000	1.241454000
7	-0.625803000	8.016050000	4.524709000
6	0.363867000	7.602253000	5.522607000
6	0.509264000	6.079311000	5.664816000
8	1.585770000	5.590991000	6.090807000
1	-1.511512000	8.398861000	4.825566000
1	1.347760000	7.993477000	5.256437000
7	-0.580385000	5.349911000	5.335960000
6	-0.633105000	3.875927000	5.373716000
6	0.264094000	3.306380000	4.253876000
8	1.170824000	2.476183000	4.526291000
6	-2.123760000	3.490858000	5.285861000
6	-2.471295000	2.025659000	5.552469000
8	-1.960318000	1.424926000	6.538844000
8	-3.365204000	1.517210000	4.737717000
1	-1.330886000	5.808559000	4.833205000
1	-0.204274000	3.518494000	6.311506000
1	-2.525223000	3.790848000	4.316713000
1	-2.648039000	4.075399000	6.055133000
7	0.090264000	3.801387000	2.998370000
6	1.004095000	3.393894000	1.921162000
6	2.436259000	3.897195000	2.185081000
8	3.430414000	3.218323000	1.809998000
6	0.512083000	3.830706000	0.519772000
6	-0.792430000	3.162086000	0.000931000
6	-0.741154000	1.651888000	-0.049365000
8	-0.440938000	1.024518000	1.009906000
8	-1.023919000	1.038659000	-1.191312000
1	-0.669440000	4.449314000	2.801323000
1	1.083559000	2.307234000	1.940802000
1	1.320817000	3.609213000	-0.187259000
1	0.359858000	4.916710000	0.482890000
1	-1.638577000	3.421049000	0.651034000
1	-1.008200000	3.550820000	-0.996693000
7	2.576765000	5.088440000	2.826465000
6	3.888607000	5.603098000	3.213370000
6	4.663009000	4.687842000	4.167286000
8	5.883025000	4.458895000	3.959147000
1	1.770113000	5.679696000	2.994435000
1	4.539214000	5.733634000	2.346168000
7	3.996748000	4.175036000	5.232354000
6	4.685517000	3.276530000	6.158158000
6	4.883170000	1.858045000	5.607246000
8	6.005478000	1.276523000	5.705501000
1	3.050208000	4.483377000	5.450699000
1	5.682152000	3.659570000	6.386111000
7	3.809627000	1.262170000	5.042203000
6	3.927835000	-0.079535000	4.459922000
6	2.803422000	-0.382021000	3.464904000
6	1.482141000	-0.697632000	4.093404000
7	0.342919000	-0.944298000	3.315583000
6	1.113167000	-0.784173000	5.418391000
6	-0.678512000	-1.154726000	4.165355000
7	-0.242230000	-1.071565000	5.436192000
1	2.927709000	1.765583000	4.924263000
1	3.955426000	-0.834602000	5.259302000
1	3.110478000	-1.231628000	2.839901000
1	2.697779000	0.469262000	2.781485000
1	1.671363000	-0.613910000	6.321811000
1	-1.704868000	-1.330420000	3.894044000
6	1.408075000	0.151409000	-4.231950000
6	2.224821000	-0.659561000	-5.213611000
8	2.027613000	-1.905483000	-5.385377000
6	0.116261000	0.777371000	-4.832523000
6	-0.853126000	-0.166472000	-5.559933000
6	-1.628777000	-1.179380000	-4.750114000
8	-2.038464000	-0.771989000	-3.483002000
8	-1.988552000	-2.270800000	-5.190270000
1	1.150832000	-0.513695000	-3.401887000
1	-0.417240000	1.285988000	-4.025350000
1	0.402826000	1.556781000	-5.547363000
1	-0.365447000	-0.719314000	-6.368413000
1	-1.613764000	0.484707000	-6.014467000
7	3.190420000	-0.036486000	-5.931735000
6	3.964372000	-0.746074000	-6.962052000
6	4.769350000	-1.946171000	-6.442349000
8	4.902317000	-2.963414000	-7.154725000
1	3.335225000	0.955031000	-5.804607000
1	3.298296000	-1.145720000	-7.730380000
7	5.311885000	-1.819896000	-5.196266000
6	6.048680000	-2.901574000	-4.557733000
6	5.247955000	-3.612660000	-3.464589000
8	5.819009000	-4.100382000	-2.445140000
1	5.345927000	-0.927039000	-4.711611000
1	6.318800000	-3.634765000	-5.326768000
7	3.919142000	-3.729787000	-3.675655000
6	3.038812000	-4.486087000	-2.776314000
6	1.640297000	-4.664711000	-3.379890000
8	0.980782000	-3.360107000	-3.503723000
6	0.752140000	-5.556955000	-2.518152000
1	3.510122000	-3.261923000	-4.478752000
1	2.950346000	-3.970030000	-1.812193000
1	1.721497000	-5.083562000	-4.391060000
1	1.241411000	-2.839504000	-4.321958000
1	-0.250886000	-5.604604000	-2.949721000
1	1.160205000	-6.571752000	-2.465220000
1	0.672128000	-5.164168000	-1.497857000
6	-5.234280000	-4.265688000	3.028408000
6	-6.418177000	-3.606237000	3.718826000
8	-7.493139000	-3.401498000	3.083628000
6	-5.671107000	-5.551915000	2.298818000
6	-4.577546000	-6.150981000	1.371230000
6	-4.184821000	-5.190148000	0.274332000
8	-3.293587000	-4.297820000	0.481832000
7	-4.832421000	-5.253683000	-0.903291000
1	-4.844553000	-3.543239000	2.299932000
1	-6.576119000	-5.333768000	1.721439000
1	-5.944363000	-6.319324000	3.033496000
1	-4.942475000	-7.091567000	0.943353000
1	-3.675423000	-6.376345000	1.949704000
1	-4.601621000	-4.579509000	-1.630885000
1	-5.536048000	-5.952164000	-1.085879000
7	-6.254010000	-3.236140000	5.013290000
6	-7.346789000	-2.624730000	5.770200000
6	-7.842662000	-1.273508000	5.244165000
8	-9.003603000	-0.893949000	5.514429000
1	-5.324026000	-3.282942000	5.434888000
1	-8.221546000	-3.280340000	5.769708000
7	-6.959861000	-0.543843000	4.502479000
6	-7.357040000	0.701116000	3.863930000
6	-7.469108000	0.611230000	2.341445000
8	-7.363116000	1.658486000	1.645002000
1	-5.987594000	-0.840473000	4.389701000

1	-8.337866000	0.979606000	4.266785000
7	-7.713203000	-0.616388000	1.817397000
6	-7.913898000	-0.838492000	0.386554000
6	-7.010962000	-1.942414000	-0.157836000
8	-5.598523000	-1.562980000	-0.110304000
1	-7.792302000	-1.422469000	2.436095000
1	-7.725594000	0.106713000	-0.127274000
1	-7.122858000	-2.846152000	0.448193000
1	-7.289085000	-2.168887000	-1.193607000
1	-5.153297000	-1.449356000	-0.984945000
6	-10.775252000	5.362773000	-3.134928000
6	-9.498027000	4.614727000	-2.818888000
6	-9.350962000	3.914876000	-1.608944000
6	-8.436485000	4.581739000	-3.741455000
6	-8.184117000	3.193446000	-1.325211000
6	-7.266877000	3.864478000	-3.466842000
6	-7.138075000	3.168298000	-2.257758000
1	-11.479988000	4.729123000	-3.690631000
1	-11.284562000	5.690704000	-2.222468000
1	-10.159152000	3.933415000	-0.882269000
1	-8.530656000	5.118602000	-4.681811000
1	-8.086883000	2.663601000	-0.381434000
1	-6.460529000	3.850148000	-4.193585000
1	-6.229828000	2.611185000	-2.044544000
26	0.028167000	-1.004340000	1.294494000
26	-1.536085000	-0.834999000	-1.504620000
8	0.451670000	-3.037063000	0.782059000
1	1.442621000	-3.128481000	0.758626000
1	-0.133962000	-3.658921000	1.405689000
8	-4.081448000	-0.867043000	4.023713000
1	-3.801715000	0.013718000	4.439050000
1	-4.027254000	-0.793358000	3.019718000
8	-3.936032000	-0.413475000	1.446015000
1	-4.710753000	-0.827048000	0.928697000
1	-3.952706000	0.588011000	1.409385000
8	-1.780866000	-1.241158000	0.316439000
1	-3.209070000	-3.112805000	-0.786945000
1	-2.638117000	-0.957619000	0.820111000
8	-0.833572000	-2.622125000	8.805571000
1	-0.478878000	-3.260106000	8.106409000
1	-0.918456000	-3.020634000	9.684666000
8	-3.616709000	-1.484290000	-1.929612000
8	-3.287809000	-2.961940000	-1.784773000
8	-0.111503000	-2.171851000	-1.615606000
1	0.305507000	-2.671551000	-2.435668000
8	-1.169679000	-4.280108000	2.181811000
1	-2.011402000	-4.300194000	1.660193000
1	-1.310867000	-4.292086000	3.168708000
8	-2.201016000	-1.170915000	7.281008000
1	-1.754455000	-1.589008000	8.094531000
1	-2.286537000	-0.179966000	7.227946000
8	-1.780270000	-4.441864000	4.841224000
1	-2.092748000	-5.354750000	4.987000000
1	-2.516402000	-3.725569000	5.162315000
8	-0.002135000	-4.062653000	6.813393000
1	-0.613722000	-4.207679000	6.030582000
1	0.921841000	-3.982293000	6.527396000
8	-3.420121000	-2.735033000	5.610353000
1	-3.701781000	-2.050990000	4.896660000
1	-3.058829000	-2.196660000	6.396349000
1	4.101504000	3.229513000	7.082438000
1	3.731778000	6.579431000	3.681538000
1	0.080616000	8.023418000	6.491689000
1	-1.451387000	9.475963000	2.177099000
1	-5.220945000	7.002939000	2.787028000
1	-5.342149000	9.403175000	-3.873928000
1	-2.084394000	8.745931000	-7.963515000
1	1.283425000	11.207971000	-5.355336000
1	5.706243000	6.908736000	-4.740802000
1	5.129994000	5.786866000	0.115806000
1	8.546454000	-0.534205000	-2.211994000
1	10.862055000	2.767879000	0.578234000
1	6.667514000	2.747718000	3.376644000
1	8.878402000	-1.014949000	4.555970000
1	4.640781000	-0.023989000	-7.429200000
1	6.958789000	-2.508969000	-4.102445000
1	-7.013976000	-2.503953000	6.805648000
1	-6.654440000	1.506759000	4.090957000
1	-4.426139000	-4.477045000	3.736321000
1	-8.958242000	-1.124240000	0.196667000
1	8.539807000	-3.565627000	1.046522000
1	-10.581281000	6.247018000	-3.751878000
1	2.013654000	0.968664000	-3.819496000
1	3.490490000	-5.466912000	-2.587569000
1	0.057402000	-2.651017000	-0.736375000
1	4.893297000	-0.136085000	3.946843000
1	-0.884147000	-1.151094000	6.252802000
1	-4.153379000	2.382880000	3.839805000

[9]Pr$_{BP}$:

6	8.776541000	-2.791010000	2.268426000
6	7.983315000	-1.630898000	1.684726000
8	7.823355000	-1.555117000	0.430380000
6	7.929860000	-4.103780000	2.382654000
6	6.943611000	-4.295812000	1.273385000
7	7.269505000	-4.445218000	-0.081864000
6	5.575013000	-4.243468000	1.310039000
6	6.151218000	-4.451979000	-0.841671000
7	5.113762000	-4.338874000	0.001377000
1	9.624322000	-2.957205000	1.596380000
1	8.618125000	-4.954692000	2.454532000
1	7.362755000	-4.084107000	3.318707000
1	4.907259000	-4.133279000	2.145423000
1	6.085204000	-4.483224000	-1.933039000
1	4.109403000	-4.208137000	-0.255089000
7	7.459626000	-0.722262000	2.544177000
6	6.511278000	0.319308000	2.133104000
6	6.882349000	1.105684000	0.902604000
8	5.971527000	1.520727000	0.130497000
1	7.477099000	-0.881401000	3.548284000
1	5.518037000	-0.095843000	1.938684000
7	8.172645000	1.396382000	0.621740000
6	8.503415000	2.162190000	-0.584124000
6	7.857991000	1.613774000	-1.864967000
8	7.495895000	2.406157000	-2.770518000
1	8.905131000	1.091732000	1.245444000
1	8.167087000	3.198407000	-0.495034000
7	7.731174000	0.262267000	-1.980727000
6	7.163666000	-0.330969000	-3.189973000
6	5.654016000	-0.145088000	-3.379328000
8	5.123120000	-0.464976000	-4.474512000
1	7.932430000	-0.342870000	-1.185656000
1	7.638912000	0.100612000	-4.075198000
7	4.956133000	0.331851000	-2.320285000
6	3.526787000	0.656791000	-2.408097000
6	3.315593000	2.174492000	-2.249555000
8	2.650176000	2.833618000	-3.097481000
6	2.691815000	-0.147772000	-1.385747000
6	2.868108000	-1.666132000	-1.555891000
6	2.157916000	-2.481515000	-0.499362000
8	1.136742000	-1.928616000	0.087906000
8	2.546009000	-3.673012000	-0.210061000
1	5.456244000	0.585868000	-1.470683000
1	3.218720000	0.411304000	-3.426303000
1	1.636310000	0.112896000	-1.518236000
1	2.965029000	0.147320000	-0.365359000
1	3.928968000	-1.938428000	-1.554358000
1	2.468371000	-1.999528000	-2.526393000
7	3.894494000	2.745519000	-1.167098000
6	3.785265000	4.166642000	-0.869051000
6	4.436381000	5.106043000	-1.875222000
8	4.072443000	6.319248000	-1.891814000
1	4.421490000	2.186333000	-0.498759000
1	2.736454000	4.474053000	-0.791016000
7	5.348644000	4.622306000	-2.745029000
6	5.876088000	5.471591000	-3.814644000
6	4.798092000	6.080066000	-4.720465000
8	5.052688000	7.123254000	-5.361108000
1	5.741762000	3.690884000	-2.642688000
1	6.432743000	6.321155000	-3.409404000

7	3.586837000	5.444634000	-4.779776000
6	2.462683000	6.029151000	-5.517162000
6	1.268289000	6.280626000	-4.579729000
8	0.080530000	6.204094000	-4.995017000
6	2.056508000	5.192947000	-6.737438000
1	3.434206000	4.564404000	-4.293919000
1	2.828779000	7.006478000	-5.862067000
1	1.199842000	5.646018000	-7.241399000
1	2.897047000	5.137813000	-7.434392000
1	1.774735000	4.177665000	-6.442586000
7	1.549373000	6.636814000	-3.289691000
6	0.497439000	6.618894000	-2.263307000
6	-0.655443000	7.585021000	-2.536583000
8	-1.790710000	7.350122000	-2.039385000
6	1.079480000	6.863866000	-0.850633000
8	0.850714000	5.642324000	-0.078588000
1	2.518520000	6.623581000	-2.964806000
1	0.030357000	5.628241000	-2.240679000
1	2.150910000	7.076087000	-0.908509000
1	0.570630000	7.708716000	-0.370031000
1	1.041032000	5.768953000	0.883810000
7	-0.389821000	8.687349000	-3.277469000
6	-1.429518000	9.655403000	-3.618407000
6	-2.560257000	9.127501000	-4.512747000
8	-3.620809000	9.786187000	-4.587802000
1	0.537377000	8.786375000	-3.667782000
1	-1.916243000	10.022599000	-2.711021000
7	-2.338119000	7.960748000	-5.183663000
6	-3.391904000	7.332232000	-5.970932000
6	-3.899477000	6.010418000	-5.394239000
8	-4.586509000	5.239447000	-6.114198000
1	-1.445840000	7.472680000	-5.114480000
1	-4.226824000	8.037952000	-6.030467000
7	-3.569316000	5.740299000	-4.102946000
6	-3.937608000	4.490240000	-3.446870000
6	-2.729872000	3.575764000	-3.160976000
6	-1.925271000	3.081766000	-4.344630000
6	-2.497688000	2.860522000	-5.628127000
6	-0.554696000	2.753109000	-4.146050000
6	-1.762854000	2.266562000	-6.636750000
6	0.193631000	2.153160000	-5.135658000
6	-0.403400000	1.837357000	-6.411938000
8	0.238162000	1.153408000	-7.310359000
1	-3.035301000	6.418029000	-3.566835000
1	-4.673257000	3.985854000	-4.074931000
1	-3.115689000	2.690628000	-2.626108000
1	-2.053862000	4.089138000	-2.463443000
1	-3.511527000	3.187572000	-5.828410000
1	-0.082288000	2.978428000	-3.195291000
1	-2.191808000	2.082997000	-7.615565000
1	1.239127000	1.921265000	-4.967095000
1	-3.617209000	-4.709216000	-2.517109000
6	-4.631658000	4.703622000	2.410775000
6	-3.171556000	5.069404000	2.489293000
8	-2.273368000	4.196153000	2.668922000
6	-4.953249000	3.982131000	1.056132000
6	-5.755013000	2.667432000	1.226310000
6	-4.932110000	1.589351000	1.882132000
8	-4.228726000	0.776728000	1.232284000
8	-4.951890000	1.624326000	3.223996000
1	-4.843981000	4.017036000	3.236368000
1	-5.528348000	4.657597000	0.414059000
1	-4.023330000	3.754977000	0.521115000
1	-6.058045000	2.299075000	0.244616000
1	-6.655416000	2.836455000	1.821687000
7	-2.816897000	6.380972000	2.331180000
6	-1.425382000	6.749872000	2.039174000
6	-0.417627000	6.283881000	3.086479000
8	0.706488000	5.829908000	2.726354000
1	-3.536969000	7.054170000	2.107594000
1	-1.104356000	6.318458000	1.085235000
7	-0.729595000	6.421972000	4.399401000
6	0.218473000	6.020691000	5.447117000
6	0.354112000	4.496353000	5.607232000
8	1.461052000	3.983562000	5.901560000
1	-1.619563000	6.829821000	4.651275000
1	1.213441000	6.403796000	5.214574000
7	-0.787278000	3.786939000	5.431819000
6	-0.866447000	2.313006000	5.482332000
6	0.031025000	1.710072000	4.381133000
8	0.885619000	0.825922000	4.652216000
6	-2.364321000	1.960720000	5.351456000
6	-2.734212000	0.485827000	5.525724000
8	-2.258682000	-0.162872000	6.499248000
8	-3.593176000	0.025907000	4.648265000
1	-1.589935000	4.265745000	5.045155000
1	-0.470347000	1.947533000	6.431709000
1	-2.740006000	2.317089000	4.389684000
1	-2.894290000	2.510063000	6.142534000
7	-0.117035000	2.226421000	3.132893000
6	0.732518000	1.770164000	2.028581000
6	2.199534000	2.183837000	2.224218000
8	3.129779000	1.430620000	1.827091000
6	0.200683000	2.270625000	0.667372000
6	-1.176879000	1.696381000	0.243852000
6	-1.180706000	0.203061000	0.055634000
8	-0.924876000	-0.533744000	1.093877000
8	-1.414783000	-0.305971000	-1.106940000
1	-0.847792000	2.909594000	2.943872000
1	0.743240000	0.679943000	2.032310000
1	0.949353000	2.019287000	-0.091187000
1	0.123331000	3.363947000	0.667706000
1	-1.934391000	1.928531000	0.998817000
1	-1.483052000	2.160343000	-0.695944000
7	2.430719000	3.382332000	2.818742000
6	3.776522000	3.852454000	3.128903000
6	4.564742000	2.904803000	4.032856000
8	5.783872000	2.695068000	3.801947000
1	1.657725000	4.000655000	3.035500000
1	4.384155000	3.962248000	2.227222000
7	3.903588000	2.328176000	5.067569000
6	4.577044000	1.327983000	5.897265000
6	4.687999000	-0.048360000	5.221458000
8	5.806795000	-0.623543000	5.088592000
1	2.955403000	2.618691000	5.293651000
1	5.596445000	1.651176000	6.114019000
7	3.529049000	-0.615086000	4.801036000
6	3.548586000	-1.920816000	4.132033000
6	2.375953000	-2.106818000	3.161418000
6	1.061195000	-2.378311000	3.828441000
7	-0.134182000	-2.525243000	3.100940000
6	0.741087000	-2.506473000	5.160353000
6	-1.131792000	-2.719402000	3.992987000
7	-0.626772000	-2.721166000	5.234681000
1	2.651776000	-0.093069000	4.827716000
1	3.562661000	-2.728701000	4.877976000
1	2.617867000	-2.942110000	2.488608000
1	2.304218000	-1.212467000	2.529536000
1	1.351558000	-2.424188000	6.041675000
1	-2.181414000	-2.818406000	3.766777000
6	-0.160423000	-1.552169000	-5.409679000
6	0.940167000	-2.172558000	-6.238441000
8	1.244249000	-3.411865000	-6.111521000
6	-1.576076000	-1.757127000	-6.030832000
6	-2.205766000	-3.133781000	-5.765029000
6	-2.647033000	-3.367364000	-4.328528000
8	-2.498657000	-2.363304000	-3.481732000
8	-3.130526000	-4.484349000	-3.986591000
1	-0.135120000	-1.992037000	-4.409685000
1	-2.230141000	-0.982672000	-5.619742000
1	-1.518764000	-1.584598000	-7.112144000
1	-1.520243000	-3.947707000	-6.034238000
1	-3.091754000	-3.271275000	-6.398899000
7	1.569285000	-1.385730000	-7.130710000
6	2.491629000	-1.916910000	-8.143989000
6	3.661531000	-2.754957000	-7.621917000
8	4.046356000	-3.749732000	-8.274273000
1	1.254575000	-0.410958000	-7.219515000
1	1.959883000	-2.568750000	-8.842920000
7	4.261645000	-2.355750000	-6.459736000

6	5.364405000	-3.108978000	-5.879168000
6	4.964043000	-3.892647000	-4.631966000
8	5.799450000	-4.093104000	-3.696848000
1	4.031335000	-1.482190000	-6.000821000
1	5.717630000	-3.817357000	-6.637638000
7	3.709737000	-4.390406000	-4.611329000
6	3.224421000	-5.265317000	-3.540286000
6	1.791738000	-5.745477000	-3.802627000
8	0.887006000	-4.594839000	-3.836345000
6	1.306666000	-6.705166000	-2.720271000
1	3.067214000	-4.124085000	-5.355889000
1	3.236135000	-4.732598000	-2.581302000
1	1.741618000	-6.235139000	-4.784244000
1	0.883716000	-4.150742000	-4.744096000
1	0.264829000	-6.979740000	-2.905224000
1	1.910080000	-7.619073000	-2.711178000
1	1.373324000	-6.238998000	-1.730042000
6	-5.899934000	-6.062233000	3.568326000
6	-6.989104000	-5.108873000	4.035876000
8	-7.999815000	-4.861730000	3.319468000
6	-6.481018000	-7.282871000	2.830412000
6	-5.393365000	-8.316360000	2.427048000
6	-4.233329000	-7.648850000	1.720372000
8	-3.160324000	-7.384220000	2.360165000
7	-4.401903000	-7.274479000	0.443346000
1	-5.236434000	-5.504144000	2.892861000
1	-7.029711000	-6.937032000	1.947938000
1	-7.214577000	-7.789437000	3.468232000
1	-5.840782000	-9.087528000	1.788358000
1	-4.993119000	-8.810290000	3.316459000
1	-3.722014000	-6.657241000	-0.017749000
1	-5.249544000	-7.496733000	-0.056420000
7	-6.796476000	-4.543582000	5.257407000
6	-7.828694000	-3.737162000	5.906206000
6	-8.206231000	-2.430140000	5.205980000
8	-9.327180000	-1.915781000	5.417127000
1	-5.871961000	-4.626228000	5.684239000
1	-8.758339000	-4.307801000	5.984261000
7	-7.266727000	-1.883744000	4.382592000
6	-7.554367000	-0.702386000	3.586032000
6	-7.590880000	-0.970214000	2.081904000
8	-7.351747000	-0.030580000	1.272960000
1	-6.329303000	-2.279083000	4.304790000
1	-8.537979000	-0.332139000	3.898524000
7	-7.922943000	-2.229966000	1.699964000
6	-8.119090000	-2.612883000	0.302432000
6	-7.198362000	-3.741461000	-0.147109000
8	-5.822880000	-3.261074000	-0.235069000
1	-8.092185000	-2.941184000	2.410462000
1	-7.954870000	-1.727523000	-0.315177000
1	-7.255857000	-4.577242000	0.562089000
1	-7.516511000	-4.089019000	-1.137275000
1	-5.229274000	-3.846997000	-0.778067000
6	-7.394908000	2.904770000	-5.329315000
6	-6.937665000	1.953885000	-4.242416000
6	-7.602466000	1.883804000	-3.005459000
6	-5.831481000	1.105841000	-4.448324000
6	-7.181747000	0.997356000	-2.004512000
6	-5.403380000	0.219175000	-3.452876000
6	-6.078210000	0.163325000	-2.225295000
1	-7.645054000	2.362141000	-6.249904000
1	-8.287218000	3.458602000	-5.020352000
1	-8.463630000	2.523656000	-2.831353000
1	-5.310973000	1.137211000	-5.402264000
1	-7.705920000	0.941135000	-1.055278000
1	-4.552885000	-0.432661000	-3.635364000
1	-5.762724000	-0.526478000	-1.449734000
26	-0.444951000	-2.486855000	1.127664000
26	-1.798646000	-2.183235000	-1.708373000
8	0.145377000	-4.417759000	0.783413000
1	1.117352000	-4.480038000	0.603575000
1	-0.340565000	-5.140596000	1.479767000
8	-4.401239000	-2.319406000	3.822406000
1	-4.094964000	-1.455947000	4.249192000
1	-4.382435000	-2.240173000	2.822623000
8	-4.256098000	-1.881141000	1.189837000
1	-5.017615000	-2.337429000	0.672640000
1	-4.280927000	-0.881937000	1.161325000
8	-2.125625000	-2.863444000	0.249811000
1	-2.576333000	-4.386212000	-0.228354000
1	-2.988740000	-2.437857000	0.679563000
8	-1.560508000	-4.469799000	8.734174000
1	-1.310504000	-5.220228000	8.093803000
1	-1.884515000	-4.796462000	9.587290000
8	-4.002015000	-4.746909000	-1.558216000
8	-2.847726000	-5.237387000	-0.712923000
8	-0.393041000	-3.664270000	-1.843112000
1	0.104970000	-4.046299000	-2.652609000
8	-1.036405000	-5.870640000	2.308496000
1	-1.753238000	-6.498614000	2.012489000
1	-1.224853000	-5.684671000	3.270343000
8	-2.442004000	-2.775946000	7.125945000
1	-2.135261000	-3.296136000	7.955724000
1	-2.536090000	-1.785562000	7.146241000
8	-2.260085000	-6.007970000	4.676190000
1	-2.700148000	-6.741884000	4.188220000
1	-2.954273000	-5.293761000	4.978245000
8	-1.001566000	-6.255252000	6.971931000
1	-1.461850000	-6.251077000	6.073688000
1	-0.230823000	-6.842932000	6.987192000
8	-3.891762000	-4.223747000	5.498357000
1	-4.147061000	-3.556462000	4.779139000
1	-3.446486000	-3.706897000	6.247525000
1	4.021514000	1.232155000	6.834730000
1	3.681403000	4.831587000	3.607204000
1	-0.109256000	6.457586000	6.394981000
1	-1.378618000	7.840715000	1.955206000
1	-5.268714000	5.585895000	2.533584000
1	-4.427886000	4.714777000	-2.491967000
1	-3.051804000	7.125288000	-6.989773000
1	-0.958549000	10.508036000	-4.115572000
1	6.562841000	4.865437000	-4.411572000
1	4.248531000	4.333742000	0.107662000
1	7.365367000	-1.406798000	-3.184491000
1	9.591447000	2.163187000	-0.700891000
1	6.402615000	1.030350000	2.961536000
1	9.181602000	-2.551327000	3.256564000
1	2.881941000	-1.062429000	-8.705777000
1	6.174484000	-2.435529000	-5.605248000
1	-7.489648000	-3.508841000	6.921605000
1	-6.821984000	0.088362000	3.763418000
1	-5.289305000	-6.381377000	4.418614000
1	-9.156728000	-2.942930000	0.160097000
1	8.204192000	-4.429039000	-0.460249000
1	-6.619105000	3.636838000	-5.589392000
1	0.023910000	-0.483348000	-5.296958000
1	3.893162000	-6.130998000	-3.450715000
1	-0.344069000	-4.245761000	-1.049133000
1	4.494421000	-1.987638000	3.585106000
1	-1.243587000	-2.804132000	6.090605000
1	-4.351052000	0.931280000	3.741899000

$^{7}Re_{BP}$:

6	8.609158000	-1.431731000	3.508541000
6	8.033664000	-0.338884000	2.618296000
8	8.025625000	-0.497194000	1.360957000
6	7.644048000	-2.645763000	3.712510000
6	6.835456000	-3.003487000	2.505506000
7	7.351719000	-3.422162000	1.271860000
6	5.486406000	-2.877949000	2.304326000
6	6.362318000	-3.511833000	0.351838000
7	5.221344000	-3.187499000	0.975702000
1	9.527940000	-1.773712000	3.019871000
1	8.235576000	-3.499529000	4.065429000
1	6.937735000	-2.404068000	4.513165000
1	4.710142000	-2.582853000	2.985463000
1	6.459345000	-3.754096000	-0.707713000
1	4.271493000	-3.010617000	0.538854000
7	7.566551000	0.784502000	3.218247000
6	6.867872000	1.845657000	2.484335000

6	7.605796000	2.419265000	1.299114000
8	6.957598000	2.978980000	0.365042000
1	7.387045000	0.800954000	4.225787000
1	5.900684000	1.499141000	2.102621000
7	8.948960000	2.320019000	1.221324000
6	9.667131000	2.748585000	0.018679000
6	9.172531000	2.102875000	-1.285719000
8	9.376151000	2.688410000	-2.370223000
1	9.452783000	1.890185000	1.982852000
1	9.573237000	3.827352000	-0.127306000
7	8.539118000	0.891651000	-1.197527000
6	7.991033000	0.262142000	-2.390868000
6	6.533298000	0.610294000	-2.715868000
8	5.988362000	0.114377000	-3.741206000
1	8.412947000	0.428993000	-0.296863000
1	8.592308000	0.559309000	-3.255104000
7	5.878813000	1.426274000	-1.856672000
6	4.464026000	1.763488000	-2.048418000
6	4.163615000	3.264018000	-1.977846000
8	3.237560000	3.742034000	-2.696382000
6	3.526176000	1.049586000	-1.036433000
6	3.643775000	-0.481341000	-1.052310000
6	2.634062000	-1.148986000	-0.132127000
8	1.599805000	-0.480917000	0.216592000
8	2.844815000	-2.376996000	0.259434000
1	6.386383000	1.802615000	-1.058124000
1	4.214645000	1.442461000	-3.061516000
1	2.496588000	1.329161000	-1.281289000
1	3.710346000	1.436283000	-0.027035000
1	4.651703000	-0.806313000	-0.769595000
1	3.462770000	-0.884352000	-2.059684000
7	4.836802000	4.012234000	-1.070551000
6	4.348618000	5.316319000	-0.627499000
6	4.188471000	6.372194000	-1.703453000
8	3.298465000	7.256543000	-1.580649000
1	5.534309000	3.583485000	-0.458786000
1	3.366218000	5.224466000	-0.155275000
7	5.010857000	6.339459000	-2.780570000
6	4.821802000	7.299517000	-3.869525000
6	3.407997000	7.257802000	-4.461413000
8	2.895893000	8.297986000	-4.948947000
1	5.717273000	5.619714000	-2.844887000
1	4.981127000	8.322738000	-3.521143000
7	2.765110000	6.059597000	-4.440979000
6	1.417854000	5.930751000	-4.996977000
6	0.355961000	6.674171000	-4.178258000
8	-0.778422000	6.885477000	-4.693145000
6	1.040000000	4.453564000	-5.177562000
1	3.139070000	5.265107000	-3.921192000
1	1.403901000	6.433607000	-5.970452000
1	0.013428000	4.385656000	-5.545173000
1	1.708018000	3.984765000	-5.907712000
1	1.122843000	3.912072000	-4.230115000
7	0.687168000	7.131973000	-2.948595000
6	-0.238726000	7.979527000	-2.201286000
6	-0.662484000	9.220621000	-3.007119000
8	-1.797967000	9.727094000	-2.804824000
6	0.369799000	8.404113000	-0.860739000
8	0.269251000	7.267781000	0.049360000
1	1.602040000	6.953451000	-2.540825000
1	-1.167456000	7.437182000	-1.999089000
1	1.414240000	8.703597000	-0.991217000
1	-0.208778000	9.253374000	-0.474829000
1	0.800463000	7.398053000	0.868443000
7	0.235985000	9.733807000	-3.885962000
6	-0.079280000	10.916792000	-4.684381000
6	-1.151584000	10.729220000	-5.764018000
8	-1.624040000	11.738350000	-6.332523000
1	1.116587000	9.255996000	-4.067177000
1	-0.441856000	11.720680000	-4.037805000
7	-1.536263000	9.450933000	-6.045809000
6	-2.614909000	9.168872000	-6.980518000
6	-3.801144000	8.452064000	-6.342862000
8	-4.590320000	7.780613000	-7.058146000
1	-1.110011000	8.656321000	-5.575064000
1	-2.949267000	10.126932000	-7.394321000
7	-3.948381000	8.583877000	-4.995471000
6	-5.066249000	7.979630000	-4.273395000
6	-4.635184000	6.916547000	-3.247600000
6	-4.025087000	5.630845000	-3.760349000
6	-3.976226000	5.277953000	-5.137465000
6	-3.501680000	4.714859000	-2.798809000
6	-3.398975000	4.089102000	-5.534939000
6	-2.936112000	3.517642000	-3.170638000
6	-2.847499000	3.175231000	-4.568321000
8	-2.277217000	2.072608000	-4.963757000
1	-3.261767000	9.111811000	-4.464038000
1	-5.745355000	7.564570000	-5.022462000
1	-5.519216000	6.650903000	-2.644245000
1	-3.924692000	7.379348000	-2.545433000
1	-4.377552000	5.951436000	-5.888249000
1	-3.565220000	4.972643000	-1.745543000
1	-3.340944000	3.812524000	-6.581542000
1	-2.546556000	2.811798000	-2.444017000
1	-1.751971000	0.900086000	-4.177550000
6	-4.580394000	6.379861000	3.051312000
6	-3.117412000	6.737778000	3.027642000
8	-2.212239000	5.854043000	3.078753000
6	-5.015914000	5.777719000	1.669985000
6	-5.802372000	4.450438000	1.792607000
6	-4.931424000	3.327437000	2.295432000
8	-4.272133000	2.584086000	1.525976000
8	-4.863801000	3.248082000	3.631175000
1	-4.725644000	5.624342000	3.829459000
1	-5.639234000	6.504809000	1.138424000
1	-4.131664000	5.600017000	1.045290000
1	-6.180595000	4.161831000	0.810325000
1	-6.655461000	4.558866000	2.465306000
7	-2.765695000	8.053549000	2.902473000
6	-1.403625000	8.417956000	2.486025000
6	-0.303738000	7.903209000	3.410107000
8	0.771030000	7.451906000	2.924937000
1	-3.494660000	8.746902000	2.807999000
1	-1.181286000	7.994585000	1.500401000
7	-0.505160000	7.989546000	4.750924000
6	0.501527000	7.523679000	5.707371000
6	0.593965000	5.994382000	5.827913000
8	1.663576000	5.464608000	6.220096000
1	-1.371869000	8.385328000	5.088113000
1	1.489680000	7.880466000	5.410636000
7	-0.528983000	5.307123000	5.519935000
6	-0.630409000	3.834352000	5.540428000
6	0.218197000	3.253033000	4.389996000
8	1.126465000	2.415040000	4.629081000
6	-2.134935000	3.499487000	5.485617000
6	-2.512169000	2.030909000	5.682282000
8	-1.991732000	1.372357000	6.625874000
8	-3.431135000	1.580195000	4.861657000
1	-1.276690000	5.796144000	5.041496000
1	-0.188345000	3.449965000	6.461072000
1	-2.558759000	3.862360000	4.548049000
1	-2.614951000	4.056445000	6.302511000
7	0.003280000	3.749124000	3.141730000
6	0.866071000	3.328117000	2.027263000
6	2.319374000	3.787693000	2.250488000
8	3.286301000	3.090110000	1.841172000
6	0.341542000	3.824894000	0.659550000
6	-1.013115000	3.236560000	0.181739000
6	-1.019562000	1.731174000	0.001241000
8	-0.731135000	1.017876000	1.023176000
8	-1.330474000	1.220879000	-1.157144000
1	-0.756844000	4.403039000	2.972059000
1	0.904045000	2.238601000	2.015180000
1	1.110094000	3.584562000	-0.085434000
1	0.244562000	4.917878000	0.661222000
1	-1.809831000	3.472045000	0.898754000
1	-1.268767000	3.713609000	-0.766677000
7	2.506168000	4.977213000	2.886423000
6	3.838803000	5.474756000	3.218081000
6	4.643972000	4.551636000	4.137624000

8	5.852204000	4.312793000	3.876935000
1	1.716767000	5.581478000	3.085441000
1	4.453977000	5.597446000	2.324330000
7	4.019902000	4.043116000	5.229256000
6	4.733168000	3.120243000	6.111155000
6	4.876542000	1.708360000	5.527806000
8	5.989872000	1.101913000	5.564284000
1	3.086386000	4.358084000	5.489990000
1	5.746226000	3.481618000	6.297978000
7	3.766758000	1.147500000	5.000083000
6	3.832428000	-0.172756000	4.363566000
6	2.673658000	-0.407702000	3.388916000
6	1.365913000	-0.729819000	4.041956000
7	0.202695000	-0.930388000	3.286976000
6	1.032591000	-0.863629000	5.372706000
6	-0.796711000	-1.161362000	4.155530000
7	-0.325672000	-1.135879000	5.417864000
1	2.891122000	1.670728000	4.934560000
1	3.861451000	-0.959418000	5.132276000
1	2.943719000	-1.231416000	2.715006000
1	2.567429000	0.478034000	2.750613000
1	1.617527000	-0.734165000	6.265909000
1	-1.832272000	-1.309029000	3.903857000
6	1.899294000	0.410183000	-5.120243000
6	2.679358000	-0.705180000	-5.786590000
8	2.449055000	-1.926979000	-5.536319000
6	0.626727000	0.835411000	-5.904436000
6	-0.452973000	-0.257745000	-6.087709000
6	-0.923905000	-0.888328000	-4.794390000
8	-1.419125000	-0.022378000	-3.828791000
8	-0.860870000	-2.090784000	-4.545192000
1	1.617367000	0.066847000	-4.119328000
1	0.184979000	1.689197000	-5.382793000
1	0.913480000	1.193431000	-6.901351000
1	-0.091920000	-1.074587000	-6.716829000
1	-1.314482000	0.206044000	-6.583918000
7	3.621694000	-0.355721000	-6.700360000
6	4.307608000	-1.344138000	-7.541314000
6	5.215970000	-2.338316000	-6.810121000
8	5.468954000	-3.437236000	-7.350601000
1	3.807064000	0.624337000	-6.858044000
1	3.575497000	-1.953435000	-8.078018000
7	5.709529000	-1.958135000	-5.599503000
6	6.518872000	-2.851549000	-4.786405000
6	5.765447000	-3.424986000	-3.586617000
8	6.397434000	-3.765349000	-2.542919000
1	5.558914000	-1.032045000	-5.209260000
1	6.847774000	-3.683073000	-5.420257000
7	4.435392000	-3.592570000	-3.731950000
6	3.601585000	-4.263357000	-2.728009000
6	2.132416000	-4.303244000	-3.159702000
8	1.602662000	-2.933174000	-3.181955000
6	1.281859000	-5.133147000	-2.203337000
1	3.984400000	-3.216547000	-4.560863000
1	3.657595000	-3.732654000	-1.771889000
1	2.055321000	-4.715807000	-4.175084000
1	1.697762000	-2.533298000	-4.086526000
1	0.236027000	-5.113846000	-2.521916000
1	1.620138000	-6.174810000	-2.188082000
1	1.341904000	-4.732917000	-1.185948000
6	-5.361134000	-4.085223000	2.909313000
6	-6.535605000	-3.473309000	3.665372000
8	-7.621635000	-3.210798000	3.074074000
6	-5.836748000	-5.078817000	1.833479000
6	-4.727236000	-5.533693000	0.843748000
6	-4.102434000	-4.364240000	0.115097000
8	-3.107534000	-3.753565000	0.644816000
7	-4.639651000	-3.949429000	-1.036460000
1	-4.815035000	-3.257097000	2.438580000
1	-6.665807000	-4.621522000	1.283940000
1	-6.244325000	-5.976238000	2.314746000
1	-5.156960000	-6.244421000	0.128507000
1	-3.927745000	-6.050518000	1.384400000
1	-4.286543000	-3.100202000	-1.504254000
1	-5.417398000	-4.440336000	-1.451287000

7	-6.335038000	-3.190807000	4.978147000
6	-7.398748000	-2.621527000	5.804276000
6	-7.862082000	-1.211024000	5.425232000
8	-8.987628000	-0.812078000	5.799314000
1	-5.393124000	-3.260633000	5.370643000
1	-8.292729000	-3.248621000	5.751712000
7	-6.998712000	-0.455236000	4.686786000
6	-7.388245000	0.847314000	4.168140000
6	-7.634988000	0.864403000	2.657947000
8	-7.564279000	1.953672000	2.024034000
1	-6.049858000	-0.774790000	4.483707000
1	-8.317834000	1.137412000	4.671186000
7	-7.960993000	-0.321852000	2.086677000
6	-8.303471000	-0.457631000	0.673631000
6	-7.361652000	-1.402959000	-0.067978000
8	-6.000363000	-0.876372000	-0.133889000
1	-7.988595000	-1.164722000	2.657461000
1	-8.281182000	0.536116000	0.224208000
1	-7.321578000	-2.365716000	0.451071000
1	-7.733072000	-1.561676000	-1.087339000
1	-5.655318000	-0.712047000	-1.051775000
6	-9.281031000	4.756484000	-4.376365000
6	-8.449097000	3.946352000	-3.405092000
6	-8.773460000	3.905069000	-2.037224000
6	-7.350759000	3.189959000	-3.853091000
6	-8.027585000	3.129423000	-1.141299000
6	-6.600664000	2.412301000	-2.962967000
6	-6.934321000	2.382598000	-1.603150000
1	-10.130096000	4.171944000	-4.756240000
1	-9.694613000	5.653032000	-3.901593000
1	-9.621717000	4.480405000	-1.674739000
1	-7.085731000	3.210589000	-4.906984000
1	-8.288021000	3.089176000	-0.088054000
1	-5.758953000	1.829828000	-3.325846000
1	-6.356750000	1.785877000	-0.905128000
26	-0.164990000	-0.946761000	1.260744000
26	-1.764515000	-0.674633000	-1.785089000
8	0.300383000	-2.980975000	0.854197000
1	1.293116000	-3.020306000	0.767049000
1	-0.165217000	-3.631777000	1.522929000
8	-4.147143000	-0.758904000	3.963456000
1	-3.861022000	0.090271000	4.433981000
1	-4.146422000	-0.602058000	2.971083000
8	-4.104495000	-0.073072000	1.389048000
1	-4.936379000	-0.380796000	0.881810000
1	-4.075717000	0.925367000	1.454307000
8	-2.105733000	-1.309533000	0.404716000
1	-2.435811000	-2.261848000	0.391326000
1	-2.868320000	-0.719508000	0.805694000
8	-1.050605000	-2.676302000	8.890855000
1	-0.662366000	-3.327773000	8.220810000
1	-1.213545000	-3.066204000	9.762632000
8	-4.378197000	-0.413193000	-2.207738000
8	-3.578720000	-1.553243000	-2.134345000
8	-0.384735000	-2.165061000	-1.631050000
1	0.277174000	-2.458597000	-2.329624000
8	-1.145696000	-4.348481000	2.361216000
1	-1.989040000	-4.293366000	1.846245000
1	-1.299367000	-4.368070000	3.344680000
8	-2.293311000	-1.220331000	7.265759000
1	-1.896536000	-1.653723000	8.098771000
1	-2.345249000	-0.224778000	7.221468000
8	-1.905011000	-4.493996000	4.968705000
1	-2.275608000	-5.387798000	5.091440000
1	-2.629385000	-3.740301000	5.186767000
8	-0.172018000	-4.147768000	6.960873000
1	-0.779625000	-4.287007000	6.171997000
1	0.761007000	-4.143337000	6.695223000
8	-3.527259000	-2.687548000	5.546255000
1	-3.783362000	-2.010203000	4.828582000
1	-3.161017000	-2.156866000	6.342129000
1	4.191158000	3.070145000	7.060445000
1	3.713701000	6.453607000	3.690728000
1	0.269837000	7.942581000	6.691055000
1	-1.347576000	9.509818000	2.427168000

1	-5.198977000	7.248051000	3.302971000
1	-5.611168000	8.764991000	-3.735478000
1	-2.275139000	8.543809000	-7.811607000
1	0.840374000	11.257667000	-5.167120000
1	5.553339000	7.083574000	-4.653626000
1	5.051774000	5.682815000	0.126921000
1	8.046221000	-0.827225000	-2.292214000
1	10.727462000	2.515619000	0.153039000
1	6.648519000	2.666950000	3.180299000
1	8.885411000	-1.041701000	4.492873000
1	4.902631000	-0.801272000	-8.281649000
1	7.398835000	-2.329727000	-4.407677000
1	-7.053244000	-2.613147000	6.842541000
1	-6.631308000	1.603821000	4.387012000
1	-4.660211000	-4.563241000	3.603243000
1	-9.324811000	-0.852436000	0.582443000
1	8.330583000	-3.529879000	1.055893000
1	-8.692667000	5.073081000	-5.244260000
1	2.534322000	1.294727000	-4.988156000
1	3.975984000	-5.283455000	-2.574825000
1	-0.225025000	-2.637306000	-0.769865000
1	4.782810000	-0.230213000	3.823350000
1	-0.948543000	-1.230203000	6.246073000
1	-4.233608000	2.516472000	4.060063000

^{7}TS1$_{BP}$:

6	8.588489000	-1.446639000	3.480731000
6	8.008300000	-0.342313000	2.607726000
8	7.998990000	-0.482060000	1.348118000
6	7.633628000	-2.672362000	3.660255000
6	6.832285000	-3.017692000	2.444949000
7	7.355377000	-3.418139000	1.207997000
6	5.483246000	-2.897181000	2.240636000
6	6.369044000	-3.502160000	0.283685000
7	5.224626000	-3.192286000	0.907794000
1	9.511744000	-1.772357000	2.989163000
1	8.231554000	-3.526529000	4.001252000
1	6.922163000	-2.450842000	4.462170000
1	4.702395000	-2.614562000	2.922063000
1	6.467483000	-3.731126000	-0.777733000
1	4.273111000	-3.014755000	0.472391000
7	7.541912000	0.772020000	3.224737000
6	6.846590000	1.847047000	2.507585000
6	7.585353000	2.432595000	1.328860000
8	6.938887000	3.001463000	0.398967000
1	7.359818000	0.772725000	4.231999000
1	5.875854000	1.512432000	2.124716000
7	8.928853000	2.336053000	1.252574000
6	9.648708000	2.780058000	0.056595000
6	9.155123000	2.152068000	-1.256733000
8	9.354818000	2.754626000	-2.332707000
1	9.431938000	1.899824000	2.010975000
1	9.555902000	3.860739000	-0.075298000
7	8.527813000	0.936518000	-1.186066000
6	7.982056000	0.322209000	-2.388293000
6	6.521733000	0.665700000	-2.705106000
8	5.979187000	0.183484000	-3.738130000
1	8.401003000	0.461578000	-0.291786000
1	8.579706000	0.637430000	-3.248611000
7	5.863299000	1.463469000	-1.831970000
6	4.447121000	1.797251000	-2.017923000
6	4.144986000	3.296814000	-1.939494000
8	3.215605000	3.776839000	-2.653693000
6	3.513598000	1.070540000	-1.010885000
6	3.635982000	-0.459337000	-1.048501000
6	2.632547000	-1.150007000	-0.136043000
8	1.603243000	-0.489419000	0.240341000
8	2.847849000	-2.387240000	0.216676000
1	6.369717000	1.831577000	-1.028792000
1	4.197556000	1.482423000	-3.033060000
1	2.482549000	1.350887000	-1.249075000
1	3.701003000	1.445497000	0.002257000
1	4.646339000	-0.783559000	-0.773592000
1	3.456574000	-0.846273000	-2.062586000
7	4.823846000	4.042975000	-1.035751000
6	4.346944000	5.353075000	-0.598278000
6	4.195842000	6.403119000	-1.681186000
8	3.310852000	7.293760000	-1.567393000
1	5.517964000	3.610829000	-0.422046000
1	3.364321000	5.272569000	-0.124989000
7	5.020347000	6.358341000	-2.756404000
6	4.840482000	7.314065000	-3.850816000
6	3.427770000	7.278696000	-4.445688000
8	2.922321000	8.319366000	-4.938789000
1	5.724434000	5.635635000	-2.813447000
1	5.005935000	8.338023000	-3.507476000
7	2.777810000	6.083993000	-4.421713000
6	1.430985000	5.961123000	-4.979233000
6	0.369842000	6.705384000	-4.160288000
8	-0.767117000	6.910930000	-4.672411000
6	1.046901000	4.486058000	-5.162676000
1	3.144320000	5.290886000	-3.894623000
1	1.420223000	6.466182000	-5.951621000
1	0.018871000	4.424300000	-5.527553000
1	1.710768000	4.015563000	-5.895548000
1	1.130158000	3.943023000	-4.216098000
7	0.703799000	7.169153000	-2.933973000
6	-0.222148000	8.016444000	-2.186428000
6	-0.649328000	9.254650000	-2.994322000
8	-1.786299000	9.758923000	-2.793060000
6	0.388846000	8.442717000	-0.847541000
8	0.290660000	7.305780000	0.061298000
1	1.619861000	6.993355000	-2.527277000
1	-1.149440000	7.472556000	-1.981725000
1	1.432726000	8.743127000	-0.980168000
1	-0.189571000	9.291681000	-0.460494000
1	0.813047000	7.439917000	0.885803000
7	0.247045000	9.766677000	-3.875698000
6	-0.070809000	10.945189000	-4.679906000
6	-1.147659000	10.750306000	-5.753742000
8	-1.625632000	11.755319000	-6.324680000
1	1.129206000	9.290659000	-4.053456000
1	-0.430582000	11.753291000	-4.037023000
7	-1.530656000	9.469582000	-6.027050000
6	-2.616711000	9.179598000	-6.950367000
6	-3.803922000	8.483073000	-6.292399000
8	-4.599266000	7.798659000	-6.988613000
1	-1.101197000	8.678181000	-5.553900000
1	-2.947469000	10.132750000	-7.378240000
7	-3.946457000	8.646831000	-4.947960000
6	-5.072757000	8.076528000	-4.211459000
6	-4.656036000	7.041889000	-3.151589000
6	-4.085705000	5.722312000	-3.621921000
6	-4.003062000	5.341982000	-4.990319000
6	-3.631494000	4.804496000	-2.627013000
6	-3.457161000	4.126119000	-5.346939000
6	-3.099683000	3.581206000	-2.959449000
6	-2.972510000	3.211053000	-4.346629000
8	-2.427443000	2.083270000	-4.705951000
1	-3.252820000	9.178157000	-4.428723000
1	-5.753640000	7.644953000	-4.949697000
1	-5.537264000	6.821185000	-2.527001000
1	-3.923985000	7.513753000	-2.477457000
1	-4.359820000	6.012271000	-5.765922000
1	-3.722240000	5.080869000	-1.580708000
1	-3.375010000	3.828135000	-6.385954000
1	-2.760485000	2.877975000	-2.207499000
1	-1.886486000	0.926368000	-3.952753000
6	-4.573693000	6.412760000	2.962078000
6	-3.110831000	6.775862000	2.972507000
8	-2.204194000	5.894647000	3.025296000
6	-4.976362000	5.815524000	1.568141000
6	-5.726769000	4.464904000	1.663110000
6	-4.835736000	3.369121000	2.192634000
8	-4.094713000	2.678841000	1.448279000
8	-4.853062000	3.253465000	3.526275000
1	-4.734801000	5.654321000	3.734520000
1	-5.611926000	6.532398000	1.037142000
1	-4.078956000	5.668583000	0.954295000
1	-6.065125000	4.166402000	0.668719000

1	-6.603153000	4.549183000	2.309158000
7	-2.762062000	8.095136000	2.874534000
6	-1.396148000	8.469713000	2.479432000
6	-0.303288000	7.951408000	3.410147000
8	0.774382000	7.500171000	2.931633000
1	-3.491876000	8.787629000	2.780558000
1	-1.161323000	8.055071000	1.493241000
7	-0.513170000	8.035494000	4.749872000
6	0.489063000	7.567218000	5.710379000
6	0.583283000	6.037213000	5.819922000
8	1.653857000	5.503740000	6.203685000
1	-1.380474000	8.432989000	5.083436000
1	1.478144000	7.928314000	5.422149000
7	-0.541557000	5.353719000	5.509654000
6	-0.646603000	3.881432000	5.518963000
6	0.228055000	3.304180000	4.386707000
8	1.126830000	2.461589000	4.641157000
6	-2.149093000	3.549162000	5.420804000
6	-2.529230000	2.083111000	5.628443000
8	-2.021132000	1.440481000	6.589044000
8	-3.435448000	1.620093000	4.801086000
1	-1.289728000	5.848881000	5.039464000
1	-0.231508000	3.488594000	6.448634000
1	-2.543790000	3.899483000	4.466035000
1	-2.654462000	4.116197000	6.215409000
7	0.042556000	3.809673000	3.136796000
6	0.919507000	3.381219000	2.037575000
6	2.369744000	3.842610000	2.269732000
8	3.338275000	3.149762000	1.856097000
6	0.411055000	3.861327000	0.658668000
6	-0.940810000	3.270378000	0.178541000
6	-0.957982000	1.761057000	0.057579000
8	-0.669568000	1.069947000	1.081089000
8	-1.291217000	1.225618000	-1.103240000
1	-0.713722000	4.464489000	2.954714000
1	0.959160000	2.291451000	2.039293000
1	1.185748000	3.608535000	-0.075455000
1	0.314932000	4.954308000	0.643438000
1	-1.747678000	3.535217000	0.873651000
1	-1.178043000	3.714944000	-0.789959000
7	2.553400000	5.025031000	2.918791000
6	3.884758000	5.508761000	3.275079000
6	4.668447000	4.559321000	4.186881000
8	5.879019000	4.316192000	3.942153000
1	1.762089000	5.624138000	3.124290000
1	4.511881000	5.644770000	2.391403000
7	4.019022000	4.031399000	5.254292000
6	4.705598000	3.082459000	6.129698000
6	4.844240000	1.678201000	5.525946000
8	5.954311000	1.065483000	5.565363000
1	3.082116000	4.347208000	5.500060000
1	5.719309000	3.428730000	6.339916000
7	3.736057000	1.129398000	4.982579000
6	3.797077000	-0.191812000	4.345688000
6	2.649534000	-0.421092000	3.356354000
6	1.329253000	-0.728827000	3.991497000
7	0.179421000	-0.953443000	3.223037000
6	0.976277000	-0.822523000	5.321532000
6	-0.831688000	-1.159132000	4.082955000
7	-0.381607000	-1.094132000	5.353850000
1	2.864188000	1.658740000	4.914740000
1	3.809355000	-0.977710000	5.115517000
1	2.923152000	-1.251462000	2.692270000
1	2.559951000	0.460108000	2.709411000
1	1.546790000	-0.661852000	6.219152000
1	-1.863093000	-1.319173000	3.821443000
6	1.770929000	0.434692000	-4.997674000
6	2.540848000	-0.625225000	-5.760051000
8	2.299704000	-1.862448000	-5.617260000
6	0.476213000	0.911981000	-5.713283000
6	-0.620970000	-0.157566000	-5.927922000
6	-1.076675000	-0.850038000	-4.661008000
8	-1.539210000	-0.012402000	-3.639998000
8	-1.032509000	-2.060280000	-4.472556000
1	1.517877000	0.017682000	-4.016907000
1	0.060278000	1.737024000	-5.128096000
1	0.733921000	1.326635000	-6.696014000
1	-0.282900000	-0.946736000	-6.603294000
1	-1.485731000	0.341295000	-6.382058000
7	3.493468000	-0.209714000	-6.634174000
6	4.192953000	-1.140356000	-7.528521000
6	5.102080000	-2.169950000	-6.846624000
8	5.349276000	-3.245244000	-7.434568000
1	3.690335000	0.777748000	-6.710996000
1	3.468979000	-1.721773000	-8.105013000
7	5.603318000	-1.842004000	-5.623439000
6	6.425173000	-2.764399000	-4.854775000
6	5.691262000	-3.368026000	-3.657211000
8	6.333230000	-3.715461000	-2.622808000
1	5.468938000	-0.927315000	-5.201542000
1	6.736962000	-3.577056000	-5.520894000
7	4.362281000	-3.550603000	-3.798011000
6	3.536307000	-4.238558000	-2.799129000
6	2.072559000	-4.305963000	-3.245421000
8	1.527792000	-2.942912000	-3.290470000
6	1.220908000	-5.136205000	-2.290364000
1	3.906477000	-3.172718000	-4.622500000
1	3.574543000	-3.706116000	-1.842854000
1	2.012960000	-4.729646000	-4.257517000
1	1.607600000	-2.547377000	-4.199016000
1	0.177846000	-5.128422000	-2.618359000
1	1.568730000	-6.174593000	-2.264935000
1	1.266109000	-4.729718000	-1.274786000
6	-5.430876000	-4.319540000	3.209169000
6	-6.574367000	-3.517154000	3.812337000
8	-7.607140000	-3.250428000	3.132788000
6	-5.963881000	-5.518388000	2.400389000
6	-4.878282000	-6.248642000	1.562537000
6	-4.243025000	-5.319317000	0.550721000
8	-3.301940000	-4.535607000	0.881170000
7	-4.759872000	-5.281885000	-0.695584000
1	-4.862262000	-3.650641000	2.551001000
1	-6.769071000	-5.164694000	1.747091000
1	-6.414544000	-6.250426000	3.082061000
1	-5.329266000	-7.114982000	1.064965000
1	-4.083273000	-6.619616000	2.218161000
1	-4.379842000	-4.598656000	-1.345233000
1	-5.503166000	-5.895860000	-0.988134000
7	-6.414175000	-3.098968000	5.094429000
6	-7.488068000	-2.419373000	5.817362000
6	-7.924613000	-1.062895000	5.259547000
8	-9.066886000	-0.625801000	5.525915000
1	-5.488505000	-3.167988000	5.523025000
1	-8.389216000	-3.038754000	5.822460000
7	-7.015630000	-0.389675000	4.498223000
6	-7.363534000	0.859344000	3.841851000
6	-7.489381000	0.752524000	2.322384000
8	-7.393451000	1.794350000	1.617427000
1	-6.059116000	-0.729842000	4.376531000
1	-8.332727000	1.180764000	4.241165000
7	-7.747806000	-0.482907000	1.818313000
6	-8.042079000	-0.718889000	0.406975000
6	-6.998421000	-1.569656000	-0.309380000
8	-5.754268000	-0.804422000	-0.445961000
1	-7.814075000	-1.279499000	2.451561000
1	-8.127464000	0.251245000	-0.086342000
1	-6.803060000	-2.492329000	0.250600000
1	-7.370245000	-1.826962000	-1.308268000
1	-5.168023000	-1.145462000	-1.155139000
6	-8.989084000	5.556681000	-4.041374000
6	-8.229872000	4.384549000	-3.456258000
6	-8.302290000	4.085445000	-2.084194000
6	-7.460330000	3.544454000	-4.281733000
6	-7.632729000	2.977980000	-1.546537000
6	-6.789856000	2.436120000	-3.752130000
6	-6.871016000	2.150556000	-2.383062000
1	-9.929462000	5.229303000	-4.504931000
1	-9.246782000	6.292540000	-3.272215000
1	-8.900970000	4.719029000	-1.434236000
1	-7.394017000	3.758650000	-5.345268000

1	-7.706328000	2.746726000	-0.487405000
1	-6.207864000	1.795467000	-4.409137000
1	-6.354851000	1.292916000	-1.961965000
26	-0.206848000	-1.002762000	1.177187000
26	-1.715365000	-0.626761000	-1.632402000
8	0.290416000	-3.045483000	0.797453000
1	1.281246000	-3.085806000	0.740711000
1	-0.194259000	-3.668802000	1.485455000
8	-4.168417000	-0.740092000	3.916061000
1	-3.874826000	0.107177000	4.378954000
1	-4.150373000	-0.589400000	2.921537000
8	-4.060030000	-0.005677000	1.362417000
1	-4.830886000	-0.256927000	0.760545000
1	-3.970395000	0.985922000	1.425488000
8	-2.016334000	-1.251124000	0.259471000
1	-2.939544000	-2.541907000	-0.369859000
1	-2.762581000	-0.741032000	0.729643000
8	-0.992434000	-2.527668000	8.873544000
1	-0.588215000	-3.175711000	8.208264000
1	-1.127072000	-2.908171000	9.754271000
8	-3.401591000	-1.595350000	-2.015527000
8	-3.404496000	-2.821548000	-1.244040000
8	-0.310638000	-2.092854000	-1.649103000
1	0.303457000	-2.428613000	-2.375593000
8	-1.103546000	-4.380480000	2.381385000
1	-1.961089000	-4.529121000	1.906586000
1	-1.239612000	-4.304702000	3.361349000
8	-2.320441000	-1.138688000	7.249742000
1	-1.889870000	-1.552825000	8.075429000
1	-2.366251000	-0.143523000	7.190035000
8	-1.866392000	-4.394151000	5.023427000
1	-2.206953000	-5.300467000	5.139854000
1	-2.620557000	-3.672645000	5.223582000
8	-0.088457000	-3.991856000	6.953031000
1	-0.723883000	-4.153100000	6.188771000
1	0.821489000	-3.878309000	6.635458000
8	-3.570976000	-2.638059000	5.570318000
1	-3.823905000	-1.989255000	4.829279000
1	-3.200021000	-2.084329000	6.347340000
1	4.147023000	3.021436000	7.068685000
1	3.760676000	6.478327000	3.766532000
1	0.249262000	7.979358000	6.694944000
1	-1.344755000	9.562287000	2.431131000
1	-5.200133000	7.278036000	3.203867000
1	-5.610993000	8.884055000	-3.700214000
1	-2.286182000	8.537793000	-7.772196000
1	0.847109000	11.282764000	-5.168199000
1	5.572330000	7.088925000	-4.632040000
1	5.053465000	5.717206000	0.154109000
1	8.044772000	-0.768223000	-2.308025000
1	10.708698000	2.544356000	0.189131000
1	6.636099000	2.660076000	3.216006000
1	8.858560000	-1.071803000	4.472657000
1	4.789592000	-0.549564000	-8.229870000
1	7.314382000	-2.257399000	-4.477628000
1	-7.164870000	-2.287300000	6.854667000
1	-6.631833000	1.640797000	4.058906000
1	-4.737474000	-4.653331000	3.988424000
1	-9.008009000	-1.234059000	0.326690000
1	8.335575000	-3.519331000	0.995148000
1	-8.409955000	6.066236000	-4.820347000
1	2.404426000	1.311972000	-4.818205000
1	3.929551000	-5.250963000	-2.643371000
1	-0.193217000	-2.613945000	-0.804075000
1	4.754199000	-0.257359000	3.818373000
1	-1.009030000	-1.166962000	6.179882000
1	-4.215623000	2.535390000	3.982224000

^{7}IM1$_{BP}$:

6	8.671361000	-1.389223000	3.509127000
6	8.078764000	-0.293173000	2.634002000
8	8.078070000	-0.428905000	1.374110000
6	7.714281000	-2.606421000	3.722205000
6	6.923472000	-2.991464000	2.512088000
7	7.457571000	-3.442487000	1.297017000
6	5.576756000	-2.877620000	2.290055000
6	6.478788000	-3.567132000	0.368798000
7	5.329633000	-3.229365000	0.968759000
1	9.584023000	-1.724971000	3.005085000
1	8.308989000	-3.449498000	4.095236000
1	6.997284000	-2.358433000	4.511504000
1	4.790351000	-2.565356000	2.951712000
1	6.579785000	-3.849220000	-0.678657000
1	4.382466000	-3.071800000	0.518364000
7	7.587990000	0.810647000	3.252046000
6	6.881366000	1.875535000	2.531485000
6	7.632047000	2.484972000	1.372809000
8	6.996329000	3.040661000	0.427925000
1	7.403621000	0.808846000	4.258812000
1	5.925000000	1.524109000	2.128714000
7	8.979384000	2.422812000	1.334316000
6	9.722842000	2.899883000	0.166337000
6	9.288013000	2.271856000	-1.166630000
8	9.509111000	2.887712000	-2.230698000
1	9.471111000	1.999626000	2.107576000
1	9.602827000	3.978858000	0.041889000
7	8.686083000	1.041712000	-1.125439000
6	8.189946000	0.427762000	-2.348352000
6	6.711639000	0.680388000	-2.666364000
8	6.201471000	0.158009000	-3.696305000
1	8.541895000	0.555150000	-0.240186000
1	8.770360000	0.814318000	-3.191644000
7	6.010572000	1.456877000	-1.807574000
6	4.585524000	1.739865000	-2.013464000
6	4.237069000	3.232881000	-1.970361000
8	3.304264000	3.670092000	-2.706727000
6	3.661543000	1.012037000	-0.998935000
6	3.803808000	-0.517324000	-1.005141000
6	2.768833000	-1.201185000	-0.119478000
8	1.741621000	-0.523435000	0.233440000
8	2.953757000	-2.441654000	0.235755000
1	6.493915000	1.853753000	-1.003640000
1	4.355994000	1.394773000	-3.023514000
1	2.627522000	1.271170000	-1.247371000
1	3.834786000	1.408827000	0.008427000
1	4.806019000	-0.824803000	-0.683727000
1	3.669934000	-0.923997000	-2.018503000
7	4.882765000	4.012527000	-1.069751000
6	4.362263000	5.309291000	-0.641125000
6	4.181812000	6.354807000	-1.723418000
8	3.282796000	7.229914000	-1.597774000
1	5.580473000	3.606022000	-0.443004000
1	3.381542000	5.198938000	-0.170177000
7	4.991878000	6.323083000	-2.809484000
6	4.777343000	7.270726000	-3.904723000
6	3.355645000	7.208368000	-4.475931000
8	2.827006000	8.236261000	-4.970575000
1	5.708602000	5.613764000	-2.874558000
1	4.930513000	8.299006000	-3.568646000
7	2.723429000	6.004321000	-4.428703000
6	1.369954000	5.856651000	-4.963290000
6	0.307491000	6.585073000	-4.131314000
8	-0.845753000	6.750471000	-4.620805000
6	1.008463000	4.374687000	-5.133608000
1	3.117016000	5.216994000	-3.912362000
1	1.334897000	6.356669000	-5.937837000
1	-0.024195000	4.293046000	-5.480736000
1	1.668324000	3.911440000	-5.874485000
1	1.115663000	3.837819000	-4.185773000
7	0.655621000	7.076942000	-2.920082000
6	-0.277439000	7.905552000	-2.159351000
6	-0.768840000	9.117772000	-2.969498000
8	-1.913077000	9.590113000	-2.739608000
6	0.359195000	8.372644000	-0.846089000
8	0.308136000	7.253684000	0.088080000
1	1.585414000	6.931437000	-2.532195000
1	-1.180497000	7.335825000	-1.919188000
1	1.392791000	8.691679000	-1.012412000
1	-0.226221000	9.217621000	-0.460951000
1	0.844203000	7.415957000	0.898503000

7	0.085528000	9.646145000	-3.883282000
6	-0.295494000	10.801605000	-4.692289000
6	-1.379729000	10.550893000	-5.746897000
8	-1.891185000	11.528731000	-6.335894000
1	0.978094000	9.197410000	-4.076650000
1	-0.678773000	11.599138000	-4.049739000
7	-1.730963000	9.254036000	-5.985467000
6	-2.800463000	8.914394000	-6.910833000
6	-3.971816000	8.187826000	-6.258535000
8	-4.743869000	7.484230000	-6.961699000
1	-1.272524000	8.484889000	-5.502377000
1	-3.159781000	9.850033000	-7.354258000
7	-4.131908000	8.346921000	-4.914922000
6	-5.266745000	7.765296000	-4.198389000
6	-4.871034000	6.738125000	-3.123960000
6	-4.229847000	5.445006000	-3.571296000
6	-4.085393000	5.068238000	-4.936280000
6	-3.770027000	4.546968000	-2.560266000
6	-3.465685000	3.882829000	-5.273045000
6	-3.163534000	3.354267000	-2.873643000
6	-2.964558000	2.996581000	-4.255066000
8	-2.341202000	1.903917000	-4.597508000
1	-3.457373000	8.898247000	-4.392380000
1	-5.924165000	7.321273000	-4.950166000
1	-5.779100000	6.480132000	-2.554233000
1	-4.194371000	7.224958000	-2.404087000
1	-4.453320000	5.718134000	-5.723712000
1	-3.916441000	4.810669000	-1.517306000
1	-3.334161000	3.589380000	-6.308247000
1	-2.815168000	2.668980000	-2.110240000
1	-1.782416000	0.808072000	-3.836340000
6	-4.538604000	6.442366000	2.958626000
6	-3.074302000	6.795857000	2.986340000
8	-2.172822000	5.909607000	3.047756000
6	-4.929912000	5.866961000	1.551597000
6	-5.654896000	4.501598000	1.619049000
6	-4.750594000	3.411157000	2.135766000
8	-3.984116000	2.751113000	1.388916000
8	-4.787375000	3.262978000	3.466082000
1	-4.712779000	5.675319000	3.719396000
1	-5.580594000	6.581992000	1.036895000
1	-4.030459000	5.752513000	0.933608000
1	-5.986666000	4.213985000	0.619288000
1	-6.536450000	4.558411000	2.260542000
7	-2.717431000	8.113191000	2.889751000
6	-1.348939000	8.478377000	2.495544000
6	-0.259818000	7.955390000	3.427148000
8	0.818878000	7.506004000	2.948700000
1	-3.443181000	8.809008000	2.788590000
1	-1.116996000	8.057114000	1.511785000
7	-0.474197000	8.031054000	4.766268000
6	0.522098000	7.551995000	5.727011000
6	0.614492000	6.021119000	5.822580000
8	1.679058000	5.485104000	6.220196000
1	-1.344033000	8.423788000	5.098924000
1	1.513128000	7.915074000	5.447967000
7	-0.503736000	5.339322000	5.487180000
6	-0.608258000	3.867189000	5.489302000
6	0.260187000	3.294744000	4.349871000
8	1.161371000	2.452658000	4.600094000
6	-2.111806000	3.534970000	5.404473000
6	-2.488367000	2.063730000	5.579998000
8	-1.997201000	1.407602000	6.540882000
8	-3.372833000	1.609768000	4.724844000
1	-1.245470000	5.835172000	5.006527000
1	-0.184348000	3.471552000	6.413786000
1	-2.519107000	3.908851000	4.464174000
1	-2.604950000	4.082222000	6.219989000
7	0.072362000	3.800985000	3.100326000
6	0.958213000	3.377550000	2.004819000
6	2.403864000	3.848784000	2.245968000
8	3.379128000	3.163270000	1.835444000
6	0.459903000	3.846193000	0.617488000
6	-0.876094000	3.238865000	0.114973000
6	-0.876450000	1.728824000	-0.010371000

8	-0.536635000	1.046405000	1.006509000
8	-1.248550000	1.189432000	-1.154738000
1	-0.683721000	4.456275000	2.918998000
1	1.005328000	2.288649000	2.009844000
1	1.249485000	3.598515000	-0.102702000
1	0.351996000	4.937949000	0.595778000
1	-1.698419000	3.492959000	0.796723000
1	-1.106400000	3.682251000	-0.856054000
7	2.577113000	5.031019000	2.898387000
6	3.904347000	5.525143000	3.255653000
6	4.691743000	4.586794000	4.175590000
8	5.904458000	4.349740000	3.934584000
1	1.782111000	5.626403000	3.100791000
1	4.533188000	5.659077000	2.372743000
7	4.044525000	4.061083000	5.245468000
6	4.737741000	3.125916000	6.130257000
6	4.883340000	1.716082000	5.542100000
8	5.995660000	1.107990000	5.590573000
1	3.106257000	4.373539000	5.491471000
1	5.749728000	3.480134000	6.335510000
7	3.778974000	1.156950000	5.000989000
6	3.849642000	-0.170265000	4.378475000
6	2.718043000	-0.410480000	3.373469000
6	1.391153000	-0.728314000	3.989666000
7	0.254880000	-0.960508000	3.203222000
6	1.017616000	-0.831080000	5.312820000
6	-0.768723000	-1.179324000	4.045855000
7	-0.338462000	-1.115523000	5.322437000
1	2.905050000	1.681615000	4.923129000
1	3.851326000	-0.949180000	5.155412000
1	3.007554000	-1.240936000	2.716071000
1	2.630487000	0.468153000	2.722798000
1	1.573073000	-0.672188000	6.220084000
1	-1.794390000	-1.346299000	3.766827000
6	1.852945000	0.376476000	-4.895517000
6	2.640229000	-0.671704000	-5.654075000
8	2.370435000	-1.908727000	-5.553501000
6	0.543733000	0.811273000	-5.612619000
6	-0.523728000	-0.285864000	-5.827710000
6	-1.002137000	-0.983596000	-4.572047000
8	-1.401935000	-0.133606000	-3.524003000
8	-1.042637000	-2.196405000	-4.417138000
1	1.611452000	-0.037353000	-3.910090000
1	0.105770000	1.627643000	-5.032335000
1	0.791683000	1.228327000	-6.596894000
1	-0.161136000	-1.074362000	-6.491261000
1	-1.392453000	0.188509000	-6.301497000
7	3.633114000	-0.254363000	-6.478780000
6	4.350709000	-1.178244000	-7.367120000
6	5.188041000	-2.258885000	-6.672064000
8	5.390504000	-3.343830000	-7.259008000
1	3.853347000	0.730223000	-6.521589000
1	3.641385000	-1.716574000	-8.000592000
7	5.683778000	-1.961538000	-5.438274000
6	6.457552000	-2.924800000	-4.669035000
6	5.671110000	-3.562594000	-3.523041000
8	6.270340000	-3.985114000	-2.491759000
1	5.607690000	-1.034823000	-5.026221000
1	6.788246000	-3.719722000	-5.347331000
7	4.340908000	-3.688847000	-3.703099000
6	3.466654000	-4.379612000	-2.747705000
6	2.011712000	-4.355151000	-3.222619000
8	1.551469000	-2.958103000	-3.249789000
6	1.091274000	-5.158269000	-2.309760000
1	3.921298000	-3.269276000	-4.526290000
1	3.517329000	-3.889753000	-1.769467000
1	1.948535000	-4.747579000	-4.246790000
1	1.666721000	-2.560616000	-4.155388000
1	0.058197000	-5.079372000	-2.659216000
1	1.377493000	-6.215590000	-2.308993000
1	1.137642000	-4.786886000	-1.280873000
6	-5.402873000	-4.257499000	3.031126000
6	-6.555180000	-3.476970000	3.646629000
8	-7.587629000	-3.206390000	2.970027000
6	-5.919376000	-5.454860000	2.209717000

6	-4.833390000	-6.120438000	1.318462000
6	-4.290278000	-5.137621000	0.304940000
8	-3.347211000	-4.338792000	0.610837000
7	-4.892076000	-5.062040000	-0.897439000
1	-4.850119000	-3.570809000	2.377535000
1	-6.754804000	-5.111988000	1.589436000
1	-6.322212000	-6.219987000	2.884806000
1	-5.262823000	-6.996106000	0.818403000
1	-3.996232000	-6.465734000	1.934070000
1	-4.598262000	-4.323684000	-1.537152000
1	-5.644151000	-5.680569000	-1.157349000
7	-6.400104000	-3.078062000	4.936003000
6	-7.478313000	-2.408179000	5.660404000
6	-7.898193000	-1.036285000	5.128203000
8	-9.033568000	-0.589739000	5.407603000
1	-5.476755000	-3.151262000	5.367969000
1	-8.383445000	-3.021300000	5.638873000
7	-6.983921000	-0.359927000	4.375396000
6	-7.319196000	0.908141000	3.749294000
6	-7.434714000	0.847697000	2.226348000
8	-7.356051000	1.916382000	1.560547000
1	-6.032881000	-0.709116000	4.244289000
1	-8.288524000	1.225054000	4.151444000
7	-7.662370000	-0.373387000	1.676559000
6	-7.926655000	-0.564909000	0.250788000
6	-6.813628000	-1.292989000	-0.497692000
8	-5.633811000	-0.436711000	-0.580866000
1	-7.732521000	-1.189495000	2.283091000
1	-8.084050000	0.418735000	-0.196038000
1	-6.557738000	-2.232861000	0.007998000
1	-7.159064000	-1.523975000	-1.512715000
1	-4.991985000	-0.714606000	-1.288770000
6	-9.572319000	6.013023000	-3.703083000
6	-8.719709000	4.875534000	-3.182013000
6	-8.748072000	4.514129000	-1.822993000
6	-7.908738000	4.125425000	-4.053512000
6	-7.998877000	3.430852000	-1.344852000
6	-7.156431000	3.042974000	-3.581437000
6	-7.196208000	2.692368000	-2.225988000
1	-10.554285000	5.650838000	-4.036632000
1	-9.753335000	6.767795000	-2.929945000
1	-9.375251000	5.078416000	-1.137037000
1	-7.876694000	4.386263000	-5.108331000
1	-8.038446000	3.145335000	-0.297941000
1	-6.541019000	2.475125000	-4.273450000
1	-6.613987000	1.856113000	-1.849804000
26	-0.074240000	-0.993984000	1.161393000
26	-1.695622000	-0.712078000	-1.627470000
8	0.340391000	-3.042029000	0.769144000
1	1.327842000	-3.128829000	0.731221000
1	-0.211307000	-3.665288000	1.417519000
8	-4.112360000	-0.725214000	3.789315000
1	-3.821748000	0.113622000	4.269694000
1	-4.065301000	-0.564050000	2.798513000
8	-3.897515000	0.058673000	1.245200000
1	-4.693554000	-0.119488000	0.627919000
1	-3.786831000	1.043325000	1.358029000
8	-1.890114000	-1.191514000	0.153987000
1	-3.190973000	-2.643335000	-0.564266000
1	-2.657412000	-0.673631000	0.607987000
8	-1.074497000	-2.649152000	8.768088000
1	-0.682654000	-3.293641000	8.092841000
1	-1.233431000	-3.046515000	9.637250000
8	-3.475739000	-1.242669000	-1.997885000
8	-3.683081000	-2.627040000	-1.433437000
8	-0.248543000	-2.063764000	-1.632031000
1	0.377293000	-2.399413000	-2.356444000
8	-1.206346000	-4.326419000	2.217490000
1	-2.052183000	-4.399875000	1.703007000
1	-1.355473000	-4.318167000	3.200215000
8	-2.322235000	-1.177706000	7.157484000
1	-1.924538000	-1.619811000	7.984951000
1	-2.361676000	-0.180956000	7.117613000
8	-1.932730000	-4.438199000	4.852755000
1	-2.298466000	-5.334658000	4.970261000

1	-2.660939000	-3.690591000	5.077388000
8	-0.181236000	-4.099593000	6.827283000
1	-0.797171000	-4.237543000	6.043965000
1	0.742513000	-4.020963000	6.540748000
8	-3.560945000	-2.635204000	5.434762000
1	-3.800758000	-1.975961000	4.696773000
1	-3.191227000	-2.096257000	6.223052000
1	4.180676000	3.072163000	7.070636000
1	3.772598000	6.497450000	3.739730000
1	0.277811000	7.954277000	6.714535000
1	-1.290152000	9.570323000	2.443366000
1	-5.161089000	7.309155000	3.205007000
1	-5.827626000	8.568511000	-3.704938000
1	-2.442022000	8.273978000	-7.722230000
1	0.597803000	11.174686000	-5.199920000
1	5.500095000	7.055040000	-4.696990000
1	5.052627000	5.697134000	0.114440000
1	8.332497000	-0.656699000	-2.306370000
1	10.784702000	2.693466000	0.329152000
1	6.645599000	2.680179000	3.241336000
1	8.960308000	-1.002791000	4.491400000
1	5.002515000	-0.583254000	-8.013794000
1	7.335605000	-2.440934000	-4.238230000
1	-7.169043000	-2.301530000	6.704836000
1	-6.584351000	1.679148000	3.992655000
1	-4.699921000	-4.589105000	3.802545000
1	-8.849472000	-1.148284000	0.141027000
1	8.438830000	-3.566322000	1.101252000
1	-9.104968000	6.509418000	-4.560736000
1	2.462389000	1.272128000	-4.724640000
1	3.807866000	-5.415599000	-2.629457000
1	-0.145819000	-2.595866000	-0.788181000
1	4.814833000	-0.239212000	3.866615000
1	-0.982153000	-1.196463000	6.136075000
1	-4.150440000	2.542106000	3.911825000

$^{7}TS2_{BP}$:

6	8.992804000	-1.404653000	2.900770000
6	8.257025000	-0.204500000	2.323399000
8	8.213905000	-0.040214000	1.067846000
6	8.069260000	-2.648232000	3.098770000
6	7.119651000	-2.927716000	1.973554000
7	7.486845000	-3.121719000	0.633311000
6	5.750564000	-3.016752000	1.985169000
6	6.384545000	-3.303191000	-0.134468000
7	5.325185000	-3.246869000	0.681854000
1	9.798394000	-1.643613000	2.200019000
1	8.711089000	-3.518339000	3.289707000
1	7.464952000	-2.509603000	4.001414000
1	5.058278000	-2.930088000	2.803204000
1	6.320172000	-3.417728000	-1.213496000
1	4.301913000	-3.201839000	0.384887000
7	7.641688000	0.631963000	3.193627000
6	6.696848000	1.683547000	2.795578000
6	7.054649000	2.475016000	1.564897000
8	6.201466000	2.636993000	0.648891000
1	7.622675000	0.436750000	4.190569000
1	5.702055000	1.272206000	2.606586000
7	8.274693000	3.051879000	1.452874000
6	8.570200000	3.912863000	0.302863000
6	8.251316000	3.258187000	-1.044982000
8	7.835209000	3.952785000	-2.006394000
1	8.928562000	2.998500000	2.220408000
1	7.986376000	4.836601000	0.344172000
7	8.475296000	1.920506000	-1.153178000
6	8.208106000	1.230033000	-2.408290000
6	6.729901000	1.117631000	-2.791913000
8	6.438633000	0.871810000	-3.994243000
1	8.654662000	1.358188000	-0.323315000
1	8.692973000	1.750091000	-3.237968000
7	5.814280000	1.264217000	-1.806939000
6	4.374601000	1.402741000	-2.075012000
6	3.978152000	2.886961000	-2.173532000
8	3.346497000	3.325538000	-3.184951000
6	3.504578000	0.710075000	-1.004122000

6	3.524852000	-0.821485000	-1.089874000
6	2.593718000	-1.468132000	-0.075139000
8	1.598967000	-0.769819000	0.342558000
8	2.811075000	-2.690264000	0.310081000
1	6.134555000	1.506200000	-0.870200000
1	4.182875000	0.967715000	-3.055122000
1	2.471444000	1.049099000	-1.125094000
1	3.806071000	1.046938000	-0.005641000
1	4.537752000	-1.215228000	-0.971902000
1	3.183949000	-1.162481000	-2.079007000
7	4.298051000	3.645839000	-1.104358000
6	3.827907000	5.004706000	-0.893381000
6	4.412567000	6.083880000	-1.786200000
8	3.827823000	7.205448000	-1.812029000
1	4.812752000	3.229163000	-0.328597000
1	2.744041000	5.072963000	-1.048529000
7	5.448889000	5.806646000	-2.606618000
6	5.751851000	6.750849000	-3.687975000
6	4.510208000	7.053307000	-4.544140000
8	4.358144000	8.179173000	-5.074316000
1	6.052357000	4.999703000	-2.459349000
1	6.101652000	7.711963000	-3.304095000
7	3.601642000	6.042528000	-4.691947000
6	2.383315000	6.237715000	-5.479070000
6	1.290580000	7.030765000	-4.743328000
8	0.252626000	7.374401000	-5.373915000
6	1.821617000	4.894306000	-5.971033000
1	3.721163000	5.151495000	-4.214489000
1	2.644481000	6.861730000	-6.341293000
1	0.903334000	5.070950000	-6.535535000
1	2.548648000	4.397484000	-6.621149000
1	1.592530000	4.236342000	-5.125716000
7	1.493305000	7.375856000	-3.447100000
6	0.496572000	8.155350000	-2.716275000
6	0.253643000	9.534435000	-3.356239000
8	-0.836251000	10.132527000	-3.146526000
6	0.889829000	8.339259000	-1.245639000
8	0.646907000	7.087197000	-0.528159000
1	2.359247000	7.148587000	-2.961547000
1	-0.471844000	7.645623000	-2.758964000
1	1.944216000	8.621581000	-1.167493000
1	0.263339000	9.139392000	-0.831734000
1	0.996178000	7.144263000	0.391463000
7	1.248384000	10.049398000	-4.119840000
6	1.092448000	11.315992000	-4.831215000
6	0.060196000	11.315601000	-5.967862000
8	-0.285312000	12.410966000	-6.463768000
1	2.098117000	9.513804000	-4.259475000
1	0.773937000	12.100424000	-4.139015000
7	-0.438376000	10.109555000	-6.365232000
6	-1.529209000	10.019554000	-7.326359000
6	-2.878332000	9.652984000	-6.704759000
8	-3.810396000	9.225831000	-7.433320000
1	-0.101276000	9.235464000	-5.965695000
1	-1.626391000	10.993606000	-7.817044000
7	-2.998996000	9.830795000	-5.360791000
6	-4.244887000	9.573375000	-4.644071000
6	-4.102986000	8.474634000	-3.576993000
6	-3.835383000	7.061675000	-4.057470000
6	-4.001388000	6.650040000	-5.404672000
6	-3.434039000	6.093961000	-3.092821000
6	-3.779998000	5.333824000	-5.771198000
6	-3.216041000	4.777937000	-3.437180000
6	-3.378404000	4.342924000	-4.804354000
8	-3.166700000	3.110980000	-5.146296000
1	-2.199422000	10.140126000	-4.815867000
1	-5.001932000	9.321956000	-5.390776000
1	-5.029738000	8.466190000	-2.978676000
1	-3.298939000	8.769239000	-2.886726000
1	-4.278829000	7.372370000	-6.165868000
1	-3.305674000	6.411717000	-2.060790000
1	-3.896386000	5.010921000	-6.799818000
1	-2.929399000	4.033344000	-2.702728000
1	-3.453276000	-1.390534000	-2.676281000
6	-4.583846000	6.105239000	2.543024000

6	-3.124577000	6.475581000	2.485573000
8	-2.211600000	5.616962000	2.660310000
6	-5.002141000	5.305031000	1.261575000
6	-5.790228000	4.008926000	1.569975000
6	-4.924404000	2.964850000	2.228060000
8	-4.256257000	2.122758000	1.573249000
8	-4.864610000	3.064313000	3.562095000
1	-4.728412000	5.468510000	3.421534000
1	-5.621915000	5.942515000	0.622191000
1	-4.113828000	5.039318000	0.675626000
1	-6.167318000	3.589528000	0.636348000
1	-6.644719000	4.213555000	2.218372000
7	-2.788176000	7.770738000	2.203461000
6	-1.415771000	8.121147000	1.812970000
6	-0.355554000	7.720919000	2.836019000
8	0.741180000	7.229779000	2.449852000
1	-3.524188000	8.434608000	2.006999000
1	-1.134285000	7.626522000	0.876781000
7	-0.612395000	7.959306000	4.148853000
6	0.366737000	7.637444000	5.192169000
6	0.496169000	6.134632000	5.489640000
8	1.599504000	5.661162000	5.856828000
1	-1.490213000	8.391713000	4.401968000
1	1.357274000	7.985389000	4.893445000
7	-0.633507000	5.401084000	5.351313000
6	-0.685708000	3.933447000	5.515515000
6	0.190579000	3.269485000	4.431398000
8	1.078114000	2.434411000	4.745585000
6	-2.178447000	3.545288000	5.474420000
6	-2.513871000	2.080286000	5.766625000
8	-1.977726000	1.496130000	6.748580000
8	-3.419530000	1.556135000	4.974502000
1	-1.430187000	5.830716000	4.899046000
1	-0.245495000	3.650942000	6.473396000
1	-2.602494000	3.825747000	4.507893000
1	-2.686264000	4.140857000	6.246252000
7	0.000711000	3.690544000	3.152536000
6	0.856970000	3.189556000	2.071355000
6	2.316798000	3.644662000	2.247183000
8	3.263984000	2.893623000	1.893586000
6	0.315517000	3.588497000	0.677544000
6	-1.061672000	2.995180000	0.276357000
6	-1.093330000	1.484496000	0.243847000
8	-0.639950000	0.837109000	1.230363000
8	-1.602108000	0.902824000	-0.834211000
1	-0.747257000	4.344071000	2.932676000
1	0.891148000	2.102579000	2.136552000
1	1.066454000	3.272306000	-0.056278000
1	0.240954000	4.680055000	0.598076000
1	-1.838298000	3.312519000	0.983801000
1	-1.342229000	3.378682000	-0.707699000
7	2.529547000	4.876161000	2.785422000
6	3.868018000	5.363651000	3.103135000
6	4.638031000	4.489453000	4.096377000
8	5.865356000	4.274912000	3.909268000
1	1.753288000	5.513149000	2.925377000
1	4.504321000	5.416070000	2.215607000
7	3.959668000	3.997193000	5.161324000
6	4.622859000	3.110010000	6.118071000
6	4.818549000	1.675531000	5.602328000
8	5.941996000	1.100004000	5.698953000
1	3.007519000	4.306420000	5.344841000
1	5.618166000	3.489360000	6.357168000
7	3.739099000	1.065727000	5.058117000
6	3.855163000	-0.293056000	4.514420000
6	2.731099000	-0.621780000	3.527720000
6	1.403797000	-0.862455000	4.177522000
7	0.247378000	-1.102210000	3.425159000
6	1.047898000	-0.866224000	5.510256000
6	-0.770640000	-1.226535000	4.294670000
7	-0.316995000	-1.095387000	5.556404000
1	2.855948000	1.566464000	4.944519000
1	3.878249000	-1.024581000	5.335304000
1	3.017924000	-1.513015000	2.951587000
1	2.648564000	0.194754000	2.798714000

1	1.621644000	-0.669750000	6.398507000
1	-1.806619000	-1.374243000	4.044974000
6	0.721407000	0.933287000	-3.638832000
6	1.562984000	0.260448000	-4.696167000
8	1.310974000	-0.927587000	-5.066866000
6	-0.673030000	1.383476000	-4.165821000
6	-1.468508000	0.370686000	-5.012865000
6	-1.937845000	-0.899732000	-4.351911000
8	-2.496364000	-0.731962000	-3.071770000
8	-1.963364000	-2.007298000	-4.880814000
1	0.585014000	0.213436000	-2.823405000
1	-1.269493000	1.676536000	-3.297580000
1	-0.551465000	2.280509000	-4.782438000
1	-0.919220000	0.069318000	-5.907318000
1	-2.371281000	0.906580000	-5.340497000
7	2.576936000	0.939613000	-5.285510000
6	3.288101000	0.355442000	-6.438986000
6	3.914507000	-1.019763000	-6.155680000
8	3.565837000	-2.044824000	-6.796919000
1	2.837390000	1.856774000	-4.931359000
1	2.591374000	0.202030000	-7.266057000
7	4.862112000	-1.063587000	-5.184778000
6	5.547791000	-2.317512000	-4.857886000
6	4.746589000	-3.207252000	-3.900071000
8	5.178927000	-3.478914000	-2.739733000
1	5.258616000	-0.213053000	-4.774781000
1	5.749663000	-2.869744000	-5.783552000
7	3.573839000	-3.666861000	-4.387516000
6	2.652037000	-4.510445000	-3.620626000
6	1.179183000	-4.143015000	-3.852349000
8	0.923002000	-2.796043000	-3.334494000
6	0.234244000	-5.118083000	-3.153328000
1	3.333059000	-3.393771000	-5.343574000
1	2.896406000	-4.396742000	-2.561114000
1	0.959988000	-4.128452000	-4.928284000
1	1.007608000	-2.088445000	-4.046953000
1	-0.802298000	-4.805745000	-3.306517000
1	0.355708000	-6.129789000	-3.554894000
1	0.435171000	-5.150547000	-2.075776000
6	-5.362979000	-4.330449000	3.490026000
6	-6.512899000	-3.650678000	4.218250000
8	-7.622529000	-3.479188000	3.634712000
6	-5.825231000	-5.658256000	2.855845000
6	-4.787305000	-6.283680000	1.884041000
6	-4.483315000	-5.367756000	0.721578000
8	-3.528368000	-4.522570000	0.790636000
7	-5.276998000	-5.420830000	-0.364625000
1	-5.031246000	-3.640804000	2.702621000
1	-6.771981000	-5.481833000	2.333653000
1	-6.033422000	-6.391978000	3.644320000
1	-5.166976000	-7.245490000	1.520127000
1	-3.844445000	-6.473991000	2.406508000
1	-5.114247000	-4.766645000	-1.127574000
1	-6.032149000	-6.084459000	-0.439101000
7	-6.282809000	-3.227047000	5.485770000
6	-7.338180000	-2.587753000	6.272559000
6	-7.860729000	-1.257782000	5.717918000
8	-9.012326000	-0.871333000	6.016852000
1	-5.332043000	-3.253776000	5.862260000
1	-8.210858000	-3.242463000	6.340643000
7	-7.008883000	-0.554863000	4.916800000
6	-7.432862000	0.660150000	4.238551000
6	-7.588173000	0.499885000	2.724692000
8	-7.475454000	1.507982000	1.973254000
1	-6.040126000	-0.852129000	4.779441000
1	-8.402888000	0.950791000	4.658614000
7	-7.879979000	-0.746671000	2.274762000
6	-8.137079000	-1.046473000	0.867756000
6	-7.244633000	-2.166455000	0.339892000
8	-5.834459000	-1.777286000	0.315316000
1	-7.952024000	-1.516681000	2.937966000
1	-7.982490000	-0.132740000	0.292699000
1	-7.325244000	-3.043210000	0.988736000
1	-7.560051000	-2.438946000	-0.673795000
1	-5.430095000	-1.682203000	-0.581712000
6	-8.659774000	2.078886000	-5.026014000
6	-7.690707000	1.963997000	-3.868951000
6	-8.140557000	2.011373000	-2.538526000
6	-6.312322000	1.802342000	-4.101508000
6	-7.244848000	1.896040000	-1.466880000
6	-5.412129000	1.682572000	-3.037203000
6	-5.875318000	1.726550000	-1.714807000
1	-8.786374000	1.116279000	-5.539421000
1	-9.648936000	2.404380000	-4.688346000
1	-9.200990000	2.146402000	-2.341153000
1	-5.936272000	1.787348000	-5.120834000
1	-7.604726000	1.938098000	-0.442242000
1	-4.353319000	1.572250000	-3.246572000
1	-5.179367000	1.656161000	-0.883729000
26	-0.126371000	-1.214779000	1.406104000
26	-2.016627000	-0.948128000	-1.175597000
8	0.217575000	-3.260927000	0.916377000
1	1.198447000	-3.386237000	0.832850000
1	-0.327405000	-3.864464000	1.577037000
8	-4.153114000	-0.858550000	4.336201000
1	-3.859298000	0.034454000	4.711325000
1	-4.138448000	-0.828824000	3.330440000
8	-4.112654000	-0.537841000	1.722682000
1	-4.912347000	-0.981117000	1.267778000
1	-4.143288000	0.460622000	1.662332000
8	-1.991678000	-1.435204000	0.580985000
1	-3.495553000	-3.442285000	-0.561920000
1	-2.837802000	-1.111803000	1.083131000
8	-0.657537000	-2.513276000	8.953379000
1	-0.341899000	-3.165856000	8.248005000
1	-0.667605000	-2.886869000	9.847324000
8	-3.927458000	-1.758040000	-1.583597000
8	-3.703803000	-3.258639000	-1.535800000
8	-0.603349000	-2.269794000	-1.420185000
1	-0.024830000	-2.504772000	-2.251779000
8	-1.348044000	-4.466537000	2.434909000
1	-2.203257000	-4.497906000	1.937643000
1	-1.461946000	-4.404066000	3.421332000
8	-2.163628000	-1.097099000	7.524245000
1	-1.641429000	-1.499264000	8.300130000
1	-2.250493000	-0.107041000	7.461471000
8	-1.861356000	-4.453309000	5.141993000
1	-2.170136000	-5.356871000	5.342773000
1	-2.566724000	-3.722402000	5.478210000
8	0.067711000	-3.994295000	6.949545000
1	-0.605645000	-4.180473000	6.228707000
1	0.944048000	-3.821796000	6.568437000
8	-3.449063000	-2.700355000	5.945915000
1	-3.746373000	-2.029677000	5.228385000
1	-3.058066000	-2.146135000	6.705939000
1	4.020957000	3.089417000	7.031627000
1	3.760905000	6.373253000	3.510896000
1	0.080953000	8.162237000	6.108621000
1	-1.376336000	9.204362000	1.656695000
1	-5.215094000	6.992326000	2.662848000
1	-4.570351000	10.494709000	-4.144619000
1	-1.311953000	9.270295000	-8.091801000
1	2.064157000	11.601942000	-5.241902000
1	6.538214000	6.312565000	-4.308901000
1	4.017987000	5.263380000	0.150056000
1	8.632449000	0.223680000	-2.344951000
1	9.632348000	4.174247000	0.333408000
1	6.606422000	2.390171000	3.624565000
1	9.456860000	-1.165796000	3.863760000
1	4.059580000	1.067920000	-6.744385000
1	6.491487000	-2.069857000	-4.372958000
1	-6.953810000	-2.426930000	7.284474000
1	-6.728140000	1.477471000	4.409974000
1	-4.509094000	-4.495015000	4.155103000
1	-9.184409000	-1.355672000	0.741281000
1	8.425050000	-3.045111000	0.271829000
1	-8.306331000	2.797253000	-5.775087000
1	1.236638000	1.808920000	-3.231914000
1	2.800561000	-5.564875000	-3.892828000
1	-0.376366000	-2.815040000	-0.607191000

1	4.823697000	-0.369312000	4.009534000
1	-0.940505000	-1.119930000	6.390810000
1	-4.232587000	2.394758000	4.084074000

$^{7}Pr_{BP}$:

6	8.576498000	-2.920856000	2.288392000
6	7.854271000	-1.721700000	1.692489000
8	7.730216000	-1.634059000	0.434202000
6	7.664492000	-4.190938000	2.373798000
6	6.694060000	-4.335105000	1.242518000
7	7.041010000	-4.435066000	-0.111398000
6	5.324231000	-4.292777000	1.259589000
6	5.935015000	-4.420253000	-0.888241000
7	4.882473000	-4.343743000	-0.058765000
1	9.433349000	-3.124835000	1.638461000
1	8.309948000	-5.074118000	2.456393000
1	7.077840000	-4.149011000	3.297039000
1	4.642482000	-4.219596000	2.087693000
1	5.900598000	-4.410906000	-1.982071000
1	3.879252000	-4.204360000	-0.327133000
7	7.348673000	-0.797876000	2.545118000
6	6.443340000	0.278535000	2.126491000
6	6.836932000	1.039198000	0.887326000
8	5.937494000	1.461144000	0.105449000
1	7.338815000	-0.960547000	3.549911000
1	5.433103000	-0.097590000	1.940022000
7	8.133047000	1.305372000	0.610754000
6	8.482170000	2.056904000	-0.599055000
6	7.826390000	1.516867000	-1.878057000
8	7.478404000	2.313376000	-2.785609000
1	8.857710000	0.990818000	1.238695000
1	8.168947000	3.100839000	-0.515581000
7	7.677218000	0.167521000	-1.991940000
6	7.107029000	-0.416225000	-3.204790000
6	5.607940000	-0.180455000	-3.415527000
8	5.086997000	-0.454295000	-4.527817000
1	7.865826000	-0.439018000	-1.194979000
1	7.607684000	-0.007365000	-4.086700000
7	4.901627000	0.282790000	-2.355215000
6	3.483272000	0.646584000	-2.466592000
6	3.303695000	2.165490000	-2.284106000
8	2.655897000	2.850801000	-3.125466000
6	2.602332000	-0.156802000	-1.483495000
6	2.727974000	-1.677299000	-1.689506000
6	1.944986000	-2.485485000	-0.674296000
8	0.886807000	-1.918713000	-0.174139000
8	2.320788000	-3.671265000	-0.343061000
1	5.394272000	0.509011000	-1.493709000
1	3.193076000	0.428980000	-3.496344000
1	1.558840000	0.142702000	-1.626679000
1	2.866931000	0.101622000	-0.450506000
1	3.777202000	-1.988211000	-1.653768000
1	2.357969000	-1.967314000	-2.685107000
7	3.889259000	2.707810000	-1.191518000
6	3.811194000	4.126432000	-0.872160000
6	4.468241000	5.066590000	-1.874004000
8	4.118111000	6.283985000	-1.879867000
1	4.408357000	2.130426000	-0.532686000
1	2.769120000	4.450872000	-0.774992000
7	5.372383000	4.579896000	-2.750740000
6	5.903748000	5.431502000	-3.816642000
6	4.828874000	6.058897000	-4.713506000
8	5.093789000	7.103203000	-5.348257000
1	5.754859000	3.642998000	-2.657456000
1	6.471405000	6.272016000	-3.407745000
7	3.609467000	5.439437000	-4.771284000
6	2.489712000	6.044893000	-5.498750000
6	1.298403000	6.289710000	-4.555746000
8	0.108955000	6.218493000	-4.966924000
6	2.075990000	5.232272000	-6.732343000
1	3.446920000	4.558048000	-4.290048000
1	2.863656000	7.024594000	-5.828264000
1	1.225044000	5.702300000	-7.230614000
1	2.916989000	5.179205000	-7.428912000
1	1.782879000	4.215646000	-6.453754000
7	1.584697000	6.632090000	-3.262802000
6	0.539125000	6.590862000	-2.230376000
6	-0.616500000	7.561275000	-2.472658000
8	-1.743966000	7.322439000	-1.958606000
6	1.130591000	6.805514000	-0.816681000
8	0.880614000	5.578917000	-0.059985000
1	2.555464000	6.613518000	-2.943124000
1	0.074607000	5.598670000	-2.227476000
1	2.205893000	6.996941000	-0.876451000
1	0.642134000	7.654806000	-0.322835000
1	1.049940000	5.700639000	0.907417000
7	-0.362104000	8.671005000	-3.206071000
6	-1.407100000	9.640980000	-3.523643000
6	-2.545962000	9.119846000	-4.411615000
8	-3.610707000	9.773819000	-4.465464000
1	0.559098000	8.772890000	-3.609662000
1	-1.884386000	9.996614000	-2.606758000
7	-2.325771000	7.962982000	-5.100296000
6	-3.384374000	7.341866000	-5.886922000
6	-3.884676000	6.011734000	-5.323381000
8	-4.578023000	5.249870000	-6.046770000
1	-1.430229000	7.478732000	-5.047778000
1	-4.221452000	8.046180000	-5.930934000
7	-3.540311000	5.723741000	-4.039493000
6	-3.901533000	4.464275000	-3.397606000
6	-2.691080000	3.546020000	-3.136463000
6	-1.895146000	3.074283000	-4.334851000
6	-2.477815000	2.871402000	-5.616895000
6	-0.521477000	2.748555000	-4.153916000
6	-1.750798000	2.293174000	-6.640301000
6	0.219644000	2.166060000	-5.159366000
6	-0.388519000	1.863573000	-6.433534000
8	0.245969000	1.190880000	-7.345240000
1	-3.001622000	6.394827000	-3.500014000
1	-4.642983000	3.968968000	-4.026104000
1	-3.073266000	2.650894000	-2.615723000
1	-2.009959000	4.046188000	-2.434348000
1	-3.493952000	3.199158000	-5.803858000
1	-0.040607000	2.962625000	-3.204883000
1	-2.188196000	2.122606000	-7.617748000
1	1.268237000	1.938918000	-5.004781000
1	-3.529683000	-4.686183000	-2.521258000
6	-4.643490000	4.702163000	2.358422000
6	-3.182447000	5.063975000	2.446001000
8	-2.290004000	4.190472000	2.651084000
6	-4.955143000	3.957420000	1.014351000
6	-5.775079000	2.655374000	1.199342000
6	-4.969033000	1.583311000	1.884841000
8	-4.264962000	0.753056000	1.259312000
8	-5.002735000	1.646916000	3.225790000
1	-4.866755000	4.031397000	3.194077000
1	-5.512560000	4.626403000	0.350258000
1	-4.020348000	3.707826000	0.498227000
1	-6.070858000	2.271997000	0.221300000
1	-6.679781000	2.846529000	1.781634000
7	-2.821166000	6.371077000	2.268484000
6	-1.424616000	6.728933000	1.986673000
6	-0.432932000	6.282780000	3.057815000
8	0.692081000	5.810142000	2.725102000
1	-3.535330000	7.042021000	2.020467000
1	-1.093673000	6.276713000	1.045995000
7	-0.756998000	6.458350000	4.363293000
6	0.180960000	6.079052000	5.428907000
6	0.317088000	4.557529000	5.615705000
8	1.426844000	4.045891000	5.899758000
1	-1.645095000	6.881121000	4.596271000
1	1.177959000	6.458692000	5.199502000
7	-0.829731000	3.849100000	5.468245000
6	-0.916801000	2.376160000	5.539836000
6	0.002439000	1.752964000	4.468018000
8	0.861523000	0.883622000	4.769393000
6	-2.413929000	2.030673000	5.374106000
6	-2.794777000	0.562133000	5.568614000
8	-2.335517000	-0.065858000	6.563277000
8	-3.641643000	0.085402000	4.688779000

1	-1.637785000	4.327761000	5.093625000
1	-0.547593000	2.020870000	6.503840000
1	-2.762314000	2.369642000	4.395836000
1	-2.962019000	2.598056000	6.139892000
7	-0.139567000	2.237079000	3.205705000
6	0.699268000	1.740845000	2.111842000
6	2.169611000	2.153757000	2.273619000
8	3.089181000	1.395888000	1.860061000
6	0.148904000	2.199785000	0.744918000
6	-1.243133000	1.618943000	0.387950000
6	-1.228959000	0.130624000	0.159345000
8	-1.015176000	-0.622676000	1.208790000
8	-1.414287000	-0.334516000	-1.020800000
1	-0.866554000	2.918931000	2.997487000
1	0.704290000	0.650371000	2.152258000
1	0.877571000	1.916889000	-0.021710000
1	0.078967000	3.293247000	0.712008000
1	-1.960118000	1.828308000	1.185354000
1	-1.605359000	2.089850000	-0.528447000
7	2.411585000	3.354537000	2.857452000
6	3.760819000	3.825827000	3.148756000
6	4.555040000	2.882728000	4.051893000
8	5.772002000	2.669648000	3.814349000
1	1.640987000	3.969947000	3.089417000
1	4.358629000	3.928395000	2.239810000
7	3.899247000	2.308456000	5.092009000
6	4.574847000	1.302233000	5.912753000
6	4.689275000	-0.065137000	5.219319000
8	5.806288000	-0.646383000	5.097169000
1	2.948930000	2.591489000	5.316316000
1	5.593172000	1.625399000	6.134405000
7	3.534998000	-0.616423000	4.769594000
6	3.553296000	-1.909475000	4.075624000
6	2.360861000	-2.079681000	3.128568000
6	1.052902000	-2.282976000	3.829296000
7	-0.160189000	-2.329465000	3.133262000
6	0.756528000	-2.423793000	5.168125000
6	-1.148664000	-2.482665000	4.031963000
7	-0.617753000	-2.554117000	5.267949000
1	2.658148000	-0.094928000	4.808198000
1	3.582851000	-2.729819000	4.807513000
1	2.554056000	-2.940623000	2.472209000
1	2.308931000	-1.196838000	2.477008000
1	1.390475000	-2.396667000	6.036266000
1	-2.204715000	-2.526174000	3.821681000
6	-0.108805000	-1.524558000	-5.461534000
6	0.964210000	-2.145825000	-6.325640000
8	1.260985000	-3.389020000	-6.224276000
6	-1.543106000	-1.667416000	-6.058077000
6	-2.227865000	-3.013919000	-5.776329000
6	-2.613234000	-3.236545000	-4.322291000
8	-2.429780000	-2.233504000	-3.487029000
8	-3.076315000	-4.360539000	-3.959773000
1	-0.076076000	-1.998150000	-4.477226000
1	-2.155384000	-0.865399000	-5.636076000
1	-1.499422000	-1.498355000	-7.140408000
1	-1.597832000	-3.859191000	-6.081274000
1	-3.147063000	-3.099106000	-6.371477000
7	1.584291000	-1.350733000	-7.217121000
6	2.496423000	-1.876247000	-8.241973000
6	3.683007000	-2.697959000	-7.730013000
8	4.080212000	-3.683692000	-8.388688000
1	1.273299000	-0.373646000	-7.290018000
1	1.962298000	-2.538827000	-8.928732000
7	4.279694000	-2.295480000	-6.567360000
6	5.389506000	-3.038277000	-5.985918000
6	4.991357000	-3.808304000	-4.729700000
8	5.814168000	-3.976590000	-3.776357000
1	4.033612000	-1.430666000	-6.100205000
1	5.739845000	-3.753032000	-6.739721000
7	3.747961000	-4.333347000	-4.718986000
6	3.270997000	-5.206110000	-3.643567000
6	1.864328000	-5.741648000	-3.930896000
8	0.917056000	-4.627169000	-3.977905000
6	1.398626000	-6.717151000	-2.853823000
1	3.107805000	-4.088217000	-5.472314000
1	3.237560000	-4.657980000	-2.693882000
1	1.851416000	-6.233539000	-4.912729000
1	0.912437000	-4.169499000	-4.879530000
1	0.384328000	-7.061556000	-3.074461000
1	2.055484000	-7.592318000	-2.809816000
1	1.398613000	-6.230919000	-1.871524000
6	-5.754803000	-6.049443000	3.571767000
6	-6.888114000	-5.132639000	4.009251000
8	-7.889863000	-4.925422000	3.268426000
6	-6.273695000	-7.271900000	2.791762000
6	-5.152078000	-8.285928000	2.435438000
6	-3.974246000	-7.604758000	1.772779000
8	-2.946252000	-7.297285000	2.465022000
7	-4.083797000	-7.271690000	0.478254000
1	-5.076382000	-5.463483000	2.936007000
1	-6.782095000	-6.926826000	1.885260000
1	-7.030785000	-7.797339000	3.385171000
1	-5.561148000	-9.066673000	1.783024000
1	-4.780627000	-8.770035000	3.342695000
1	-3.398112000	-6.659848000	0.022245000
1	-4.903691000	-7.520169000	-0.054549000
7	-6.745689000	-4.556507000	5.232595000
6	-7.823210000	-3.786172000	5.851901000
6	-8.228236000	-2.493898000	5.139557000
8	-9.370342000	-2.017364000	5.324137000
1	-5.832058000	-4.605259000	5.686611000
1	-8.733444000	-4.389933000	5.905756000
7	-7.289043000	-1.918935000	4.335863000
6	-7.595249000	-0.747102000	3.532322000
6	-7.597803000	-1.016493000	2.027918000
8	-7.379510000	-0.069143000	1.222734000
1	-6.336927000	-2.282585000	4.281938000
1	-8.593963000	-0.403022000	3.826200000
7	-7.878659000	-2.287893000	1.641831000
6	-8.040398000	-2.677646000	0.241781000
6	-7.122016000	-3.821776000	-0.171857000
8	-5.737300000	-3.359126000	-0.214331000
1	-8.036621000	-3.002934000	2.350766000
1	-7.847826000	-1.798249000	-0.376285000
1	-7.214224000	-4.652244000	0.539637000
1	-7.412520000	-4.169421000	-1.170397000
1	-5.133967000	-3.948627000	-0.737386000
6	-7.367369000	2.845848000	-5.357768000
6	-6.899354000	1.914447000	-4.258725000
6	-7.585734000	1.824703000	-3.035055000
6	-5.757995000	1.108004000	-4.438327000
6	-7.150483000	0.961117000	-2.020347000
6	-5.313652000	0.245823000	-3.428556000
6	-6.009472000	0.171872000	-2.213598000
1	-7.571501000	2.294582000	-6.284501000
1	-8.288534000	3.363485000	-5.071849000
1	-8.473996000	2.431890000	-2.881507000
1	-5.221284000	1.153755000	-5.382630000
1	-7.690327000	0.888581000	-1.081140000
1	-4.434054000	-0.372047000	-3.588754000
1	-5.677911000	-0.495325000	-1.425008000
26	-0.472079000	-2.466094000	1.181300000
26	-1.646786000	-2.200142000	-1.713888000
8	0.187611000	-4.195495000	1.246880000
1	0.958970000	-4.327671000	0.637901000
1	-0.293722000	-5.017513000	1.986242000
8	-4.412283000	-2.277275000	3.857067000
1	-4.135996000	-1.401393000	4.278887000
1	-4.362547000	-2.221550000	2.855739000
8	-4.221407000	-1.908767000	1.212236000
1	-4.965963000	-2.385468000	0.696897000
1	-4.277333000	-0.911641000	1.182881000
8	-2.095130000	-2.897149000	0.211387000
1	-2.461558000	-4.329485000	-0.221211000
1	-2.938529000	-2.457468000	0.645787000
8	-1.555437000	-4.256496000	8.894345000
1	-1.298987000	-5.046277000	8.308568000
1	-1.892309000	-4.526259000	9.762289000
8	-3.872568000	-4.857157000	-1.553464000

8	-2.617047000	-5.200214000	-0.771284000
8	-0.583413000	-3.929957000	-2.050545000
1	0.040865000	-4.193747000	-2.817696000
8	-0.886838000	-5.773004000	2.675527000
1	-1.572334000	-6.396140000	2.291907000
1	-1.160757000	-5.617274000	3.634756000
8	-2.443039000	-2.659039000	7.181096000
1	-2.132074000	-3.142115000	8.030228000
1	-2.561017000	-1.669017000	7.179710000
8	-2.198501000	-5.935155000	4.902752000
1	-2.626978000	-6.691380000	4.442921000
1	-2.908106000	-5.196708000	5.150252000
8	-0.996234000	-6.148849000	7.247042000
1	-1.437265000	-6.152816000	6.342550000
1	-0.220290000	-6.728986000	7.280231000
8	-3.843392000	-4.139095000	5.572917000
1	-4.104462000	-3.495273000	4.834862000
1	-3.412940000	-3.594451000	6.317840000
1	4.018451000	1.194353000	6.848312000
1	3.671747000	4.808268000	3.621256000
1	-0.158514000	6.532904000	6.364550000
1	-1.373247000	7.817426000	1.879087000
1	-5.278239000	5.588929000	2.459019000
1	-4.382946000	4.674803000	-2.434975000
1	-3.051676000	7.148887000	-6.910926000
1	-0.943564000	10.500284000	-4.016310000
1	6.581113000	4.822619000	-4.421483000
1	4.291126000	4.269479000	0.100424000
1	7.270287000	-1.498682000	-3.189931000
1	9.570102000	2.032884000	-0.714309000
1	6.366701000	0.999902000	2.949633000
1	8.966434000	-2.705967000	3.288189000
1	2.868945000	-1.021031000	-8.814428000
1	6.199429000	-2.360768000	-5.722613000
1	-7.520097000	-3.545243000	6.875670000
1	-6.887916000	0.063673000	3.721808000
1	-5.174751000	-6.367320000	4.443993000
1	-9.077601000	-2.995537000	0.069403000
1	7.980136000	-4.382951000	-0.475277000
1	-6.614371000	3.608291000	-5.596792000
1	0.108182000	-0.465359000	-5.317567000
1	3.969321000	-6.044453000	-3.525024000
1	-1.118137000	-4.692180000	-1.721610000
1	4.487987000	-1.964021000	3.508864000
1	-1.216713000	-2.645164000	6.129340000
1	-4.402948000	0.969220000	3.762863000

[1]Re$_{BP}$:

6	8.629733000	-1.413212000	3.551542000
6	8.050167000	-0.327278000	2.655406000
8	8.053753000	-0.487379000	1.398219000
6	7.673223000	-2.634385000	3.753623000
6	6.871623000	-2.999015000	2.544037000
7	7.395326000	-3.414725000	1.312552000
6	5.522555000	-2.882630000	2.337702000
6	6.410026000	-3.511441000	0.388843000
7	5.264477000	-3.194781000	1.008310000
1	9.553661000	-1.749034000	3.068375000
1	8.269920000	-3.483353000	4.109383000
1	6.962284000	-2.397296000	4.551552000
1	4.741844000	-2.591477000	3.015434000
1	6.513442000	-3.753009000	-0.670324000
1	4.314844000	-3.022250000	0.568422000
7	7.566555000	0.791815000	3.250170000
6	6.863227000	1.845346000	2.509730000
6	7.605449000	2.427739000	1.331526000
8	6.959991000	2.982652000	0.392742000
1	7.380405000	0.808448000	4.256517000
1	5.903010000	1.488801000	2.119926000
7	8.950213000	2.341547000	1.265474000
6	9.674776000	2.778006000	0.069620000
6	9.198401000	2.127211000	-1.238979000
8	9.404972000	2.714741000	-2.321845000
1	9.451344000	1.915854000	2.031076000
1	9.570801000	3.855743000	-0.077157000
7	8.576619000	0.909616000	-1.155766000
6	8.043221000	0.275248000	-2.353123000
6	6.585182000	0.611364000	-2.689488000
8	6.052907000	0.111525000	-3.719555000
1	8.448303000	0.445450000	-0.256170000
1	8.648635000	0.577083000	-3.212824000
7	5.917152000	1.421395000	-1.835314000
6	4.500828000	1.746348000	-2.036625000
6	4.189621000	3.245329000	-1.981070000
8	3.264503000	3.710851000	-2.708780000
6	3.563908000	1.036923000	-1.020673000
6	3.695696000	-0.492512000	-1.014720000
6	2.686942000	-1.156492000	-0.091016000
8	1.663043000	-0.481655000	0.272597000
8	2.888603000	-2.390905000	0.287941000
1	6.414978000	1.801828000	-1.032559000
1	4.258210000	1.413752000	-3.047570000
1	2.533319000	1.303658000	-1.275704000
1	3.737415000	1.438717000	-0.015403000
1	4.705171000	-0.804069000	-0.721524000
1	3.523875000	-0.912439000	-2.016806000
7	4.852595000	4.005195000	-1.075548000
6	4.351516000	5.307712000	-0.641683000
6	4.180320000	6.354712000	-1.724742000
8	3.280959000	7.230297000	-1.607729000
1	5.548487000	3.584193000	-0.456689000
1	3.370170000	5.209423000	-0.168371000
7	5.003380000	6.324048000	-2.801374000
6	4.804723000	7.275114000	-3.896556000
6	3.391871000	7.215666000	-4.489468000
8	2.870767000	8.247438000	-4.985261000
1	5.716577000	5.610675000	-2.861450000
1	4.953431000	8.302112000	-3.554642000
7	2.760029000	6.011706000	-4.460515000
6	1.414584000	5.866128000	-5.016756000
6	0.343782000	6.601597000	-4.202359000
8	-0.794232000	6.792864000	-4.717036000
6	1.052333000	4.384318000	-5.190929000
1	3.141203000	5.224217000	-3.935113000
1	1.396176000	6.364455000	-5.992516000
1	0.026600000	4.304089000	-5.558418000
1	1.725395000	3.919289000	-5.918841000
1	1.140238000	3.847811000	-4.241059000
7	0.670841000	7.073424000	-2.977006000
6	-0.264919000	7.912935000	-2.232868000
6	-0.708924000	9.142473000	-3.045060000
8	-1.849404000	9.636045000	-2.840092000
6	0.342811000	8.355435000	-0.897804000
8	0.261507000	7.224278000	0.020287000
1	1.588272000	6.908926000	-2.568987000
1	-1.185319000	7.359067000	-2.023566000
1	1.382519000	8.668267000	-1.034625000
1	-0.245855000	9.199397000	-0.515522000
1	0.796908000	7.365473000	0.834764000
7	0.177749000	9.660263000	-3.933201000
6	-0.159348000	10.831136000	-4.740229000
6	-1.238057000	10.618678000	-5.808890000
8	-1.729293000	11.615997000	-6.382329000
1	1.064740000	9.194169000	-4.113247000
1	-0.526951000	11.636838000	-4.098782000
7	-1.606076000	9.332563000	-6.077511000
6	-2.683570000	9.027960000	-7.006312000
6	-3.859166000	8.299802000	-6.362229000
8	-4.645129000	7.621421000	-7.074794000
1	-1.164546000	8.547737000	-5.604193000
1	-3.032619000	9.977936000	-7.426580000
7	-4.003025000	8.429740000	-5.014259000
6	-5.117218000	7.818995000	-4.291460000
6	-4.685926000	6.751763000	-3.270187000
6	-4.052192000	5.479756000	-3.786715000
6	-4.005964000	5.127111000	-5.164112000
6	-3.505176000	4.573358000	-2.828463000
6	-3.408874000	3.949172000	-5.565561000
6	-2.916608000	3.388424000	-3.204263000
6	-2.831463000	3.047249000	-4.602608000

8	-2.241616000	1.955876000	-5.000751000
1	-3.321075000	8.966355000	-4.485600000
1	-5.796113000	7.405146000	-5.041387000
1	-5.576844000	6.466349000	-2.686290000
1	-3.992936000	7.215047000	-2.551369000
1	-4.423499000	5.793724000	-5.912318000
1	-3.567332000	4.829279000	-1.774711000
1	-3.353506000	3.673388000	-6.612522000
1	-2.500444000	2.694235000	-2.480536000
1	-1.695771000	0.791304000	-4.209691000
6	-4.595277000	6.384833000	3.023819000
6	-3.129798000	6.732050000	3.002247000
8	-2.230691000	5.842785000	3.064209000
6	-5.030043000	5.775220000	1.645010000
6	-5.796576000	4.437592000	1.775054000
6	-4.908881000	3.332499000	2.287364000
8	-4.199864000	2.624215000	1.528827000
8	-4.888498000	3.226274000	3.622881000
1	-4.749439000	5.637710000	3.808458000
1	-5.667316000	6.492142000	1.116491000
1	-4.147270000	5.608946000	1.015236000
1	-6.170551000	4.137803000	0.794496000
1	-6.652457000	4.538468000	2.445137000
7	-2.769710000	8.044414000	2.865379000
6	-1.405411000	8.396719000	2.446255000
6	-0.308560000	7.886752000	3.376225000
8	0.767422000	7.432902000	2.896007000
1	-3.494511000	8.740870000	2.761952000
1	-1.184727000	7.960785000	1.465774000
7	-0.513922000	7.979193000	4.715846000
6	0.488282000	7.514623000	5.677336000
6	0.578072000	5.985662000	5.803226000
8	1.643006000	5.456816000	6.209536000
1	-1.382607000	8.374552000	5.048423000
1	1.478052000	7.869182000	5.383310000
7	-0.541378000	5.297799000	5.484641000
6	-0.644062000	3.825418000	5.514099000
6	0.206386000	3.235558000	4.369170000
8	1.114924000	2.400446000	4.617366000
6	-2.148593000	3.491024000	5.460761000
6	-2.527085000	2.024905000	5.670762000
8	-2.007007000	1.373810000	6.619717000
8	-3.447587000	1.568036000	4.855146000
1	-1.283737000	5.783740000	4.994245000
1	-0.203008000	3.447158000	6.437773000
1	-2.571571000	3.846050000	4.519979000
1	-2.628809000	4.055907000	6.272076000
7	-0.004636000	3.721877000	3.116269000
6	0.866100000	3.296758000	2.008655000
6	2.316863000	3.760657000	2.241248000
8	3.289360000	3.064969000	1.842605000
6	0.353487000	3.790582000	0.635097000
6	-0.996432000	3.202384000	0.144253000
6	-0.999729000	1.697314000	-0.045840000
8	-0.733427000	0.981803000	0.984061000
8	-1.286071000	1.190789000	-1.208784000
1	-0.765864000	4.372217000	2.938687000
1	0.905259000	2.207139000	2.000021000
1	1.129440000	3.549133000	-0.101819000
1	0.256663000	4.883613000	0.634491000
1	-1.798809000	3.433116000	0.856436000
1	-1.244733000	3.684596000	-0.803731000
7	2.495624000	4.954488000	2.871952000
6	3.824807000	5.458731000	3.206776000
6	4.628303000	4.545130000	4.137067000
8	5.840128000	4.313025000	3.886833000
1	1.703401000	5.557611000	3.062545000
1	4.444291000	5.576341000	2.315233000
7	3.998927000	4.036820000	5.225623000
6	4.710242000	3.121128000	6.116410000
6	4.857561000	1.704940000	5.544462000
8	5.972758000	1.102038000	5.587018000
1	3.062777000	4.349280000	5.479821000
1	5.722064000	3.485468000	6.303974000
7	3.749569000	1.136087000	5.021294000
6	3.819083000	-0.191726000	4.400891000
6	2.675695000	-0.435497000	3.410237000
6	1.355816000	-0.746467000	4.044301000
7	0.200981000	-0.942154000	3.274512000
6	1.003787000	-0.871191000	5.371161000
6	-0.811383000	-1.160587000	4.131284000
7	-0.356936000	-1.132521000	5.399801000
1	2.873334000	1.657267000	4.947460000
1	3.834083000	-0.969646000	5.178898000
1	2.955538000	-1.268178000	2.751514000
1	2.581527000	0.441574000	2.758649000
1	1.577359000	-0.740075000	6.271508000
1	-1.844883000	-1.301589000	3.867515000
6	1.962620000	0.373436000	-5.126845000
6	2.763058000	-0.730459000	-5.787590000
8	2.540402000	-1.955239000	-5.543672000
6	0.684190000	0.772071000	-5.915600000
6	-0.372918000	-0.341761000	-6.108169000
6	-0.845119000	-0.987949000	-4.822340000
8	-1.366311000	-0.130340000	-3.858333000
8	-0.764314000	-2.189185000	-4.578833000
1	1.684510000	0.028818000	-4.125260000
1	0.223125000	1.616178000	-5.395003000
1	0.968138000	1.137617000	-6.910615000
1	0.008432000	-1.149450000	-6.737087000
1	-1.238840000	0.107518000	-6.610094000
7	3.712661000	-0.370466000	-6.689106000
6	4.415811000	-1.351237000	-7.525211000
6	5.318051000	-2.345722000	-6.786675000
8	5.577461000	-3.443484000	-7.326474000
1	3.892773000	0.611472000	-6.841023000
1	3.694535000	-1.960773000	-8.076073000
7	5.800646000	-1.966309000	-5.571455000
6	6.607320000	-2.857983000	-4.753580000
6	5.849106000	-3.430536000	-3.556224000
8	6.475612000	-3.762842000	-2.506704000
1	5.645586000	-1.040662000	-5.181489000
1	6.940317000	-3.689634000	-5.385117000
7	4.520947000	-3.604742000	-3.710286000
6	3.680186000	-4.269198000	-2.708106000
6	2.211034000	-4.287420000	-3.140494000
8	1.707206000	-2.907020000	-3.171248000
6	1.344059000	-5.100600000	-2.184865000
1	4.075528000	-3.235424000	-4.545185000
1	3.744120000	-3.742366000	-1.750417000
1	2.128526000	-4.701220000	-4.154881000
1	1.798484000	-2.525346000	-4.084665000
1	0.299742000	-5.063323000	-2.506870000
1	1.664267000	-6.148000000	-2.168007000
1	1.408356000	-4.701080000	-1.167642000
6	-5.316828000	-4.126754000	2.920517000
6	-6.509310000	-3.491764000	3.626272000
8	-7.570681000	-3.229172000	2.992054000
6	-5.770601000	-5.172823000	1.885639000
6	-4.646180000	-5.647946000	0.923555000
6	-4.069917000	-4.501829000	0.121461000
8	-3.109317000	-3.810344000	0.609575000
7	-4.626278000	-4.194427000	-1.056957000
1	-4.768134000	-3.317750000	2.420651000
1	-6.604128000	-4.752553000	1.312966000
1	-6.164893000	-6.055844000	2.403168000
1	-5.048026000	-6.418239000	0.255410000
1	-3.826579000	-6.098362000	1.492968000
1	-4.281771000	-3.395066000	-1.605252000
1	-5.372860000	-4.755726000	-1.438379000
7	-6.351894000	-3.197503000	4.942025000
6	-7.442465000	-2.626393000	5.730709000
6	-7.905893000	-1.224851000	5.322572000
8	-9.043792000	-0.830872000	5.662925000
1	-5.421442000	-3.262828000	5.361170000
1	-8.329683000	-3.261525000	5.661180000
7	-7.028913000	-0.469940000	4.599784000
6	-7.414480000	0.827102000	4.066472000
6	-7.631241000	0.841634000	2.552429000
8	-7.592421000	1.938177000	1.930145000

1	-6.073382000	-0.784388000	4.421922000
1	-8.356301000	1.114487000	4.547792000
7	-7.899174000	-0.352335000	1.963904000
6	-8.225051000	-0.478580000	0.545975000
6	-7.193775000	-1.285404000	-0.237597000
8	-5.910631000	-0.588043000	-0.263254000
1	-7.917102000	-1.199955000	2.528571000
1	-8.306713000	0.526951000	0.130052000
1	-7.054784000	-2.269624000	0.225442000
1	-7.548890000	-1.421915000	-1.266010000
1	-5.430148000	-0.619192000	-1.128589000
6	-9.713917000	5.656191000	-3.744431000
6	-8.829208000	4.668235000	-3.014604000
6	-9.065005000	4.346885000	-1.666039000
6	-7.769731000	4.022200000	-3.677719000
6	-8.274339000	3.403976000	-0.997604000
6	-6.975425000	3.078344000	-3.015132000
6	-7.223033000	2.766380000	-1.672204000
1	-10.577780000	5.153437000	-4.200208000
1	-10.107320000	6.421251000	-3.066201000
1	-9.879901000	4.836058000	-1.138328000
1	-7.573675000	4.254703000	-4.721376000
1	-8.462776000	3.156284000	0.042383000
1	-6.165385000	2.588546000	-3.547417000
1	-6.612835000	2.035578000	-1.149869000
26	-0.142413000	-0.962692000	1.239454000
26	-1.775635000	-0.737909000	-1.825488000
8	0.337821000	-2.994737000	0.851414000
1	1.332397000	-3.032263000	0.780731000
1	-0.138129000	-3.639194000	1.516120000
8	-4.161103000	-0.763166000	3.934460000
1	-3.879799000	0.081246000	4.416502000
1	-4.134999000	-0.597089000	2.944487000
8	-4.023780000	-0.030700000	1.370048000
1	-4.859904000	-0.252229000	0.825354000
1	-3.943773000	0.962365000	1.466579000
8	-2.090521000	-1.345693000	0.353596000
1	-2.425129000	-2.292674000	0.347003000
1	-2.831678000	-0.734820000	0.768226000
8	-1.049795000	-2.659623000	8.867837000
1	-0.653374000	-3.305654000	8.197650000
1	-1.194086000	-3.047053000	9.743942000
8	-3.919308000	-0.631760000	-2.077959000
8	-3.445775000	-1.950053000	-2.310570000
8	-0.268116000	-2.104954000	-1.626710000
1	0.402509000	-2.400818000	-2.317696000
8	-1.139609000	-4.339913000	2.353829000
1	-1.978584000	-4.290467000	1.832077000
1	-1.300166000	-4.357833000	3.336341000
8	-2.322548000	-1.214622000	7.254624000
1	-1.914546000	-1.647323000	8.082515000
1	-2.370282000	-0.218398000	7.211287000
8	-1.908131000	-4.481415000	4.959540000
1	-2.268614000	-5.377847000	5.092603000
1	-2.637715000	-3.733503000	5.179123000
8	-0.154583000	-4.117099000	6.933659000
1	-0.769329000	-4.262013000	6.151836000
1	0.772567000	-4.068989000	6.651833000
8	-3.546290000	-2.686553000	5.534230000
1	-3.799617000	-2.015285000	4.811350000
1	-3.185912000	-2.151390000	6.330147000
1	4.164995000	3.077095000	7.064173000
1	3.694270000	6.440855000	3.671103000
1	0.253800000	7.936728000	6.658982000
1	-1.343330000	9.487501000	2.374948000
1	-5.207857000	7.259784000	3.266500000
1	-5.663716000	8.600697000	-3.749883000
1	-2.337112000	8.401648000	-7.833779000
1	0.751691000	11.178971000	-5.234250000
1	5.538997000	7.061748000	-4.678808000
1	5.051169000	5.686683000	0.109896000
1	8.106637000	-0.813587000	-2.253552000
1	10.736254000	2.556199000	0.213490000
1	6.630594000	2.664855000	3.203473000
1	8.897826000	-1.018423000	4.536242000
1	5.019108000	-0.801102000	-8.253398000
1	7.484882000	-2.334685000	-4.371147000
1	-7.126546000	-2.602875000	6.778125000
1	-6.667359000	1.589503000	4.298894000
1	-4.624805000	-4.567615000	3.646803000
1	-9.198128000	-0.976777000	0.442181000
1	8.375772000	-3.514731000	1.100049000
1	-9.173500000	6.163010000	-4.551271000
1	2.580520000	1.270158000	-4.996227000
1	4.040760000	-5.294870000	-2.558220000
1	-0.144944000	-2.612453000	-0.780261000
1	4.777806000	-0.257605000	3.876641000
1	-0.987559000	-1.221417000	6.222640000
1	-4.251891000	2.502774000	4.056388000

^{1}TS1$_{BP}$:

6	8.607790000	-1.416709000	3.519174000
6	8.025452000	-0.325267000	2.631511000
8	8.026010000	-0.476733000	1.373193000
6	7.657576000	-2.644788000	3.705959000
6	6.871107000	-3.008379000	2.486289000
7	7.411228000	-3.417551000	1.259373000
6	5.523927000	-2.897906000	2.265279000
6	6.437602000	-3.516775000	0.324002000
7	5.284084000	-3.208020000	0.932151000
1	9.535546000	-1.742708000	3.036391000
1	8.256153000	-3.491788000	4.063203000
1	6.937256000	-2.416431000	4.498013000
1	4.733091000	-2.613478000	2.934436000
1	6.548164000	-3.753039000	-0.734826000
1	4.341623000	-3.048421000	0.484913000
7	7.544375000	0.789842000	3.235584000
6	6.842258000	1.851258000	2.505510000
6	7.580735000	2.437450000	1.327106000
8	6.931673000	2.998771000	0.394287000
1	7.354662000	0.795704000	4.241268000
1	5.878605000	1.501562000	2.118009000
7	8.924629000	2.348863000	1.252524000
6	9.642357000	2.790528000	0.054247000
6	9.151813000	2.152561000	-1.255428000
8	9.348576000	2.749400000	-2.334973000
1	9.429998000	1.918817000	2.012889000
1	9.543644000	3.869943000	-0.083451000
7	8.529310000	0.934772000	-1.177444000
6	7.986685000	0.310789000	-2.376082000
6	6.524696000	0.645077000	-2.695871000
8	5.982209000	0.148510000	-3.722132000
1	8.407924000	0.463429000	-0.280607000
1	8.583357000	0.623662000	-3.238032000
7	5.863324000	1.450526000	-1.831578000
6	4.445833000	1.776551000	-2.022725000
6	4.131335000	3.273836000	-1.937311000
8	3.197274000	3.747746000	-2.648798000
6	3.512993000	1.035142000	-1.025477000
6	3.644226000	-0.494682000	-1.080324000
6	2.656922000	-1.201552000	-0.167270000
8	1.601134000	-0.557891000	0.185352000
8	2.893925000	-2.417383000	0.219119000
1	6.368842000	1.830301000	-1.033155000
1	4.202429000	1.466226000	-3.040753000
1	2.481493000	1.313785000	-1.263101000
1	3.697949000	1.400624000	-0.008239000
1	4.658584000	-0.817186000	-0.820025000
1	3.453819000	-0.872675000	-2.095718000
7	4.805548000	4.023400000	-1.032958000
6	4.319741000	5.329892000	-0.594209000
6	4.163610000	6.382066000	-1.674495000
8	3.277159000	7.270389000	-1.555496000
1	5.504229000	3.596637000	-0.420914000
1	3.337066000	5.242130000	-0.122634000
7	4.985315000	6.341441000	-2.752068000
6	4.800952000	7.299045000	-3.844065000
6	3.387635000	7.261322000	-4.437379000
8	2.881061000	8.301100000	-4.930961000
1	5.691084000	5.620707000	-2.812331000

1	4.964041000	8.322702000	-3.498672000
7	2.738799000	6.066097000	-4.411563000
6	1.391602000	5.940962000	-4.968317000
6	0.330779000	6.688132000	-4.151384000
8	-0.805174000	6.894720000	-4.664648000
6	1.009520000	4.464843000	-5.147508000
1	3.108461000	5.272763000	-3.887376000
1	1.380264000	6.442910000	-5.942309000
1	-0.017858000	4.399456000	-5.513336000
1	1.675108000	3.993738000	-5.878391000
1	1.092434000	3.923976000	-4.199626000
7	0.664918000	7.152808000	-2.925234000
6	-0.259596000	8.001199000	-2.177065000
6	-0.692142000	9.237403000	-2.985105000
8	-1.827729000	9.741289000	-2.778136000
6	0.357476000	8.434058000	-0.843157000
8	0.267499000	7.300962000	0.071629000
1	1.580547000	6.975478000	-2.518542000
1	-1.185500000	7.457160000	-1.966311000
1	1.399930000	8.736253000	-0.982828000
1	-0.220791000	9.283105000	-0.456280000
1	0.800957000	7.436852000	0.888301000
7	0.199865000	9.749965000	-3.870937000
6	-0.124434000	10.928321000	-4.672473000
6	-1.205818000	10.732618000	-5.741449000
8	-1.686002000	11.737674000	-6.310577000
1	1.081702000	9.275118000	-4.052814000
1	-0.482646000	11.735084000	-4.027047000
7	-1.588535000	9.451812000	-6.014874000
6	-2.669524000	9.163173000	-6.944577000
6	-3.853522000	8.447270000	-6.302477000
8	-4.644540000	7.775728000	-7.015819000
1	-1.156037000	8.660286000	-5.544571000
1	-3.007199000	10.118684000	-7.361658000
7	-3.999121000	8.579111000	-4.954663000
6	-5.123801000	7.983046000	-4.235601000
6	-4.708846000	6.919475000	-3.204020000
6	-4.089503000	5.635358000	-3.707823000
6	-4.016475000	5.284112000	-5.084412000
6	-3.581421000	4.718547000	-2.738468000
6	-3.430993000	4.096965000	-5.473594000
6	-3.007262000	3.523629000	-3.102882000
6	-2.894904000	3.182560000	-4.499130000
8	-2.318749000	2.080046000	-4.887347000
1	-3.312266000	9.107713000	-4.424181000
1	-5.800387000	7.569109000	-4.987730000
1	-5.603782000	6.650490000	-2.618671000
1	-4.011198000	7.380709000	-2.487956000
1	-4.409293000	5.956309000	-5.840674000
1	-3.663695000	4.972913000	-1.685751000
1	-3.356160000	3.821202000	-6.519323000
1	-2.623557000	2.822085000	-2.369458000
1	-1.789096000	0.909542000	-4.119921000
6	-4.584443000	6.432716000	2.978630000
6	-3.120622000	6.790338000	2.994871000
8	-2.217100000	5.906500000	3.059941000
6	-4.983279000	5.833263000	1.584031000
6	-5.725173000	4.478340000	1.680194000
6	-4.827956000	3.388491000	2.209517000
8	-4.073539000	2.709247000	1.469026000
8	-4.853752000	3.263618000	3.543345000
1	-4.752379000	5.677476000	3.752701000
1	-5.624507000	6.545538000	1.053900000
1	-4.085931000	5.692676000	0.968641000
1	-6.062678000	4.176828000	0.686595000
1	-6.601994000	4.557344000	2.326102000
7	-2.767274000	8.107635000	2.888237000
6	-1.399467000	8.476280000	2.494183000
6	-0.309710000	7.954843000	3.426431000
8	0.765813000	7.497350000	2.948944000
1	-3.495043000	8.800900000	2.784255000
1	-1.165261000	8.059797000	1.508626000
7	-0.518650000	8.043424000	4.766032000
6	0.482473000	7.573555000	5.726864000
6	0.574950000	6.043468000	5.835145000
8	1.647258000	5.507722000	6.211366000
1	-1.384059000	8.445533000	5.098993000
1	1.472272000	7.933511000	5.439635000
7	-0.552709000	5.361257000	5.532469000
6	-0.656434000	3.889238000	5.541514000
6	0.207883000	3.311607000	4.401733000
8	1.099720000	2.458315000	4.647395000
6	-2.159578000	3.554394000	5.457299000
6	-2.527794000	2.082007000	5.639993000
8	-2.019986000	1.433319000	6.597684000
8	-3.417523000	1.618632000	4.796511000
1	-1.303508000	5.856525000	5.066309000
1	-0.232372000	3.496309000	6.467076000
1	-2.567348000	3.921531000	4.514384000
1	-2.655475000	4.103628000	6.269859000
7	0.019915000	3.824285000	3.155625000
6	0.891290000	3.398843000	2.050389000
6	2.343852000	3.853460000	2.276557000
8	3.306861000	3.153507000	1.860496000
6	0.372038000	3.881351000	0.676487000
6	-0.965509000	3.261305000	0.194527000
6	-0.927158000	1.760487000	-0.002532000
8	-0.537542000	1.043933000	0.986324000
8	-1.299434000	1.252803000	-1.144677000
1	-0.730462000	4.488360000	2.981228000
1	0.927641000	2.309404000	2.046249000
1	1.151075000	3.654100000	-0.061637000
1	0.252119000	4.971934000	0.672007000
1	-1.770393000	3.464148000	0.912747000
1	-1.240023000	3.736584000	-0.749592000
7	2.536842000	5.034765000	2.924545000
6	3.871942000	5.509604000	3.278572000
6	4.650697000	4.553620000	4.187475000
8	5.859724000	4.303757000	3.941572000
1	1.750547000	5.641211000	3.129289000
1	4.498389000	5.643289000	2.394037000
7	3.999049000	4.026485000	5.253840000
6	4.680623000	3.070353000	6.125139000
6	4.816593000	1.669595000	5.512982000
8	5.924502000	1.052907000	5.550587000
1	3.065367000	4.349384000	5.503193000
1	5.694901000	3.411942000	6.340468000
7	3.707950000	1.126952000	4.963465000
6	3.769950000	-0.188569000	4.315749000
6	2.633311000	-0.406597000	3.310471000
6	1.305875000	-0.718760000	3.929319000
7	0.159933000	-0.938045000	3.151549000
6	0.940235000	-0.820547000	5.254362000
6	-0.860416000	-1.147894000	4.003072000
7	-0.417930000	-1.091549000	5.275320000
1	2.836735000	1.658204000	4.899175000
1	3.770610000	-0.982002000	5.077956000
1	2.915801000	-1.232538000	2.644354000
1	2.553522000	0.481711000	2.671533000
1	1.503851000	-0.669235000	6.157768000
1	-1.890334000	-1.302061000	3.731147000
6	1.871224000	0.420896000	-5.093404000
6	2.652068000	-0.677489000	-5.787505000
8	2.426735000	-1.905325000	-5.565113000
6	0.591108000	0.862018000	-5.856557000
6	-0.495898000	-0.223359000	-6.044932000
6	-0.952297000	-0.871357000	-4.755112000
8	-1.456975000	-0.018323000	-3.779144000
8	-0.869040000	-2.073351000	-4.514717000
1	1.598342000	0.054699000	-4.098012000
1	0.158514000	1.708971000	-5.316277000
1	0.867710000	1.235060000	-6.850779000
1	-0.144857000	-1.032150000	-6.689806000
1	-1.360357000	0.252104000	-6.524187000
7	3.595080000	-0.304475000	-6.691446000
6	4.290846000	-1.273896000	-7.546339000
6	5.207687000	-2.270405000	-6.828424000
8	5.470762000	-3.359297000	-7.383940000
1	3.776505000	0.679298000	-6.829348000
1	3.564746000	-1.881911000	-8.092419000

7	5.697161000	-1.903500000	-5.611711000
6	6.517534000	-2.799841000	-4.812273000
6	5.771655000	-3.400455000	-3.620750000
8	6.404884000	-3.739344000	-2.577600000
1	5.540174000	-0.983579000	-5.210050000
1	6.855599000	-3.617717000	-5.458947000
7	4.445745000	-3.592635000	-3.774312000
6	3.618979000	-4.288553000	-2.781791000
6	2.149144000	-4.329214000	-3.210949000
8	1.618151000	-2.959429000	-3.224252000
6	1.301114000	-5.165919000	-2.258186000
1	3.992734000	-3.218390000	-4.602510000
1	3.676444000	-3.775503000	-1.816334000
1	2.071047000	-4.735388000	-4.228851000
1	1.700625000	-2.552744000	-4.127215000
1	0.254642000	-5.148380000	-2.574535000
1	1.641726000	-6.206846000	-2.248115000
1	1.360728000	-4.771555000	-1.238355000
6	-5.429824000	-4.329392000	3.145665000
6	-6.562927000	-3.514774000	3.751695000
8	-7.592721000	-3.238940000	3.072423000
6	-5.981478000	-5.582289000	2.434053000
6	-4.907538000	-6.369508000	1.636991000
6	-4.212575000	-5.474073000	0.631041000
8	-3.106909000	-4.917264000	0.889324000
7	-4.860405000	-5.208853000	-0.523991000
1	-4.909520000	-3.689157000	2.421422000
1	-6.792365000	-5.266686000	1.768471000
1	-6.428273000	-6.259153000	3.172752000
1	-5.381868000	-7.220700000	1.133490000
1	-4.143242000	-6.766443000	2.311874000
1	-4.476917000	-4.481045000	-1.121588000
1	-5.738970000	-5.642827000	-0.758364000
7	-6.404387000	-3.108814000	5.038407000
6	-7.481238000	-2.439675000	5.766734000
6	-7.919666000	-1.077022000	5.225710000
8	-9.061664000	-0.644589000	5.500458000
1	-5.480070000	-3.181490000	5.467988000
1	-8.381136000	-3.060900000	5.761625000
7	-7.012593000	-0.393216000	4.471878000
6	-7.361700000	0.865098000	3.834093000
6	-7.483345000	0.784513000	2.312550000
8	-7.403895000	1.842233000	1.629725000
1	-6.057316000	-0.732761000	4.342696000
1	-8.332631000	1.177743000	4.235941000
7	-7.720541000	-0.443992000	1.783134000
6	-8.011635000	-0.655484000	0.366456000
6	-6.948872000	-1.458370000	-0.377318000
8	-5.738852000	-0.647944000	-0.537586000
1	-7.782093000	-1.252549000	2.401230000
1	-8.126534000	0.323333000	-0.103290000
1	-6.708478000	-2.380011000	0.167492000
1	-7.333194000	-1.721507000	-1.370279000
1	-5.140593000	-1.004486000	-1.237191000
6	-9.183723000	5.868539000	-3.804946000
6	-8.400797000	4.688233000	-3.269453000
6	-8.482902000	4.318227000	-1.915348000
6	-7.600751000	3.908862000	-4.125483000
6	-7.795478000	3.199276000	-1.425929000
6	-6.911927000	2.790012000	-3.643105000
6	-7.004491000	2.431724000	-2.292250000
1	-10.122669000	5.542560000	-4.272459000
1	-9.446855000	6.571428000	-3.007237000
1	-9.103235000	4.904837000	-1.242009000
1	-7.526747000	4.176865000	-5.176321000
1	-7.874429000	2.913161000	-0.380923000
1	-6.305476000	2.198542000	-4.323472000
1	-6.472999000	1.565686000	-1.908566000
26	-0.173882000	-0.992185000	1.115090000
26	-1.679153000	-0.663008000	-1.709609000
8	0.326711000	-3.022507000	0.858366000
1	1.312660000	-3.097358000	0.776457000
1	-0.168649000	-3.669570000	1.547204000
8	-4.160202000	-0.729639000	3.871394000
1	-3.867602000	0.112289000	4.343413000
1	-4.156160000	-0.567233000	2.877209000
8	-4.037594000	0.021342000	1.325988000
1	-4.792632000	-0.208936000	0.690003000
1	-3.944663000	1.010900000	1.401707000
8	-1.962622000	-1.294024000	0.353451000
1	-2.812028000	-2.402984000	-0.281292000
1	-2.705479000	-0.744995000	0.781330000
8	-0.975841000	-2.532050000	8.832537000
1	-0.571720000	-3.188052000	8.175222000
1	-1.131396000	-2.909685000	9.711148000
8	-3.513709000	-1.640556000	-1.956049000
8	-3.544238000	-2.707575000	-0.979004000
8	-0.323822000	-2.182473000	-1.645299000
1	0.316625000	-2.494832000	-2.357111000
8	-0.993984000	-4.438883000	2.390869000
1	-1.800823000	-4.742817000	1.897144000
1	-1.181407000	-4.330209000	3.360134000
8	-2.303866000	-1.147333000	7.203845000
1	-1.869420000	-1.567422000	8.025811000
1	-2.355658000	-0.150072000	7.158879000
8	-1.855751000	-4.397561000	4.986647000
1	-2.209897000	-5.299467000	5.097821000
1	-2.606494000	-3.667807000	5.176644000
8	-0.092912000	-4.030811000	6.934727000
1	-0.721728000	-4.176130000	6.162140000
1	0.833672000	-4.038745000	6.648150000
8	-3.555898000	-2.635183000	5.516751000
1	-3.816006000	-1.985969000	4.778593000
1	-3.191722000	-2.083702000	6.298193000
1	4.119278000	3.005131000	7.062173000
1	3.755013000	6.479120000	3.771909000
1	0.242298000	7.985395000	6.711474000
1	-1.343749000	9.568613000	2.444874000
1	-5.208210000	7.301424000	3.214968000
1	-5.667802000	8.773197000	-3.703759000
1	-2.330730000	8.535425000	-7.774081000
1	0.790182000	11.268333000	-5.165302000
1	5.532350000	7.077872000	-4.626844000
1	5.022560000	5.696765000	0.160263000
1	8.054641000	-0.778826000	-2.289080000
1	10.703579000	2.561297000	0.188088000
1	6.616609000	2.666746000	3.206599000
1	8.870109000	-1.030077000	4.508606000
1	4.881497000	-0.714784000	-8.278026000
1	7.391201000	-2.272936000	-4.426231000
1	-7.161212000	-2.319928000	6.806534000
1	-6.633054000	1.645252000	4.066176000
1	-4.693780000	-4.606621000	3.907439000
1	-8.963467000	-1.195078000	0.279653000
1	8.394612000	-3.514895000	1.059536000
1	-8.620126000	6.416162000	-4.569238000
1	2.504642000	1.304310000	-4.946655000
1	3.999119000	-5.309328000	-2.648507000
1	-0.200279000	-2.688754000	-0.797660000
1	4.732840000	-0.252814000	3.798940000
1	-1.046448000	-1.171269000	6.104248000
1	-4.214244000	2.546736000	3.988651000

^{1}IM1$_{BP}$:

6	8.550726000	-1.398591000	3.615190000
6	8.073132000	-0.299209000	2.677241000
8	8.217222000	-0.433012000	1.424700000
6	7.488606000	-2.525389000	3.812399000
6	6.795680000	-2.942699000	2.552205000
7	7.443024000	-3.340982000	1.373670000
6	5.460111000	-2.933547000	2.241270000
6	6.538191000	-3.533854000	0.385536000
7	5.330416000	-3.296503000	0.906140000
1	9.463657000	-1.815529000	3.177540000
1	7.979725000	-3.379251000	4.296542000
1	6.720591000	-2.169319000	4.506950000
1	4.608816000	-2.689463000	2.849591000
1	6.708591000	-3.771918000	-0.654595000
1	4.418734000	-3.210278000	0.378059000
7	7.492748000	0.787003000	3.244741000

6	6.788776000	1.817224000	2.476227000	1	-0.804879000	8.923973000	-5.609605000
6	7.566217000	2.455965000	1.355091000	1	-2.526320000	10.512809000	-7.443083000
8	6.945911000	2.972631000	0.377203000	7	-3.664881000	9.102505000	-5.025796000
1	7.220386000	0.770413000	4.232446000	6	-4.849576000	8.636431000	-4.307816000
1	5.871635000	1.421139000	2.025534000	6	-4.543009000	7.571283000	-3.239819000
7	8.913674000	2.470436000	1.383701000	6	-4.101960000	6.198631000	-3.702179000
6	9.684157000	2.991723000	0.252714000	6	-4.033891000	5.815473000	-5.069987000
6	9.333389000	2.357927000	-1.101310000	6	-3.753579000	5.239489000	-2.703747000
8	9.557463000	2.998452000	-2.149874000	6	-3.596199000	4.554144000	-5.421064000
1	9.390365000	2.069463000	2.177912000	6	-3.326395000	3.972566000	-3.029009000
1	9.521401000	4.065437000	0.131621000	6	-3.209108000	3.596236000	-4.417080000
7	8.803493000	1.093654000	-1.096666000	8	-2.754538000	2.430677000	-4.774645000
6	8.407113000	0.466871000	-2.349381000	1	-2.936122000	9.569293000	-4.492266000
6	6.936041000	0.633498000	-2.750637000	1	-5.554999000	8.267345000	-5.056620000
8	6.539250000	0.117252000	-3.831312000	1	-5.443416000	7.447153000	-2.615874000
1	8.659058000	0.589255000	-0.221817000	1	-3.770587000	7.974238000	-2.566069000
1	9.005972000	0.897763000	-3.157249000	1	-4.310846000	6.518130000	-5.849773000
7	6.131072000	1.323608000	-1.908778000	1	-3.836127000	5.524514000	-1.658523000
6	4.702248000	1.538581000	-2.177155000	1	-3.524013000	4.251592000	-6.459567000
6	4.289319000	3.019303000	-2.139734000	1	-3.069638000	3.237602000	-2.272751000
8	3.424965000	3.453505000	-2.957020000	1	-2.324066000	1.184748000	-4.003189000
6	3.776708000	0.778073000	-1.188077000	6	-4.684987000	6.374137000	2.982181000
6	3.840668000	-0.756448000	-1.293664000	6	-3.225555000	6.750812000	2.940186000
6	2.777818000	-1.425001000	-0.429833000	8	-2.310824000	5.879934000	3.016393000
8	1.696471000	-0.754974000	-0.229521000	6	-5.114080000	5.675629000	1.645031000
8	2.965709000	-2.601613000	0.070958000	6	-5.889841000	4.352905000	1.859415000
1	6.548424000	1.728369000	-1.072490000	6	-5.002911000	3.289083000	2.455716000
1	4.525526000	1.188089000	-3.195884000	8	-4.240998000	2.572889000	1.760346000
1	2.744820000	1.092876000	-1.372414000	8	-5.035605000	3.240432000	3.795088000
1	4.006024000	1.101840000	-0.164857000	1	-4.821086000	5.670264000	3.809732000
1	4.827119000	-1.149080000	-1.032663000	1	-5.740277000	6.360811000	1.063308000
1	3.640746000	-1.082752000	-2.324445000	1	-4.227310000	5.456650000	1.037794000
7	4.803814000	3.780140000	-1.142634000	1	-6.247836000	3.982541000	0.896824000
6	4.190712000	5.028109000	-0.700273000	1	-6.752555000	4.504968000	2.511565000
6	4.208739000	6.181757000	-1.680962000	7	-2.890239000	8.067058000	2.780054000
8	3.378504000	7.121670000	-1.543616000	6	-1.528536000	8.443304000	2.371755000
1	5.493740000	3.392213000	-0.495679000	6	-0.431399000	7.965696000	3.320140000
1	3.138513000	4.873087000	-0.443988000	8	0.647603000	7.498842000	2.859818000
7	5.091906000	6.159636000	-2.707371000	1	-3.627224000	8.748960000	2.667061000
6	5.014431000	7.182256000	-3.751190000	1	-1.285789000	8.008690000	1.395798000
6	3.624615000	7.248582000	-4.395795000	7	-0.635667000	8.102648000	4.656338000
8	3.185678000	8.335701000	-4.849023000	6	0.377402000	7.677675000	5.626958000
1	5.759433000	5.403737000	-2.771138000	6	0.494437000	6.152328000	5.773030000
1	5.216352000	8.176561000	-3.346112000	8	1.585466000	5.636565000	6.121090000
7	2.912952000	6.089114000	-4.449239000	1	-1.499844000	8.514956000	4.979703000
6	1.577767000	6.067002000	-5.047135000	1	1.360115000	8.045941000	5.326637000
6	0.535822000	6.853336000	-4.242110000	7	-0.636651000	5.450491000	5.530300000
8	-0.567224000	7.135684000	-4.789846000	6	-0.724161000	3.979154000	5.584893000
6	1.105032000	4.625166000	-5.272309000	6	0.127593000	3.371358000	4.451561000
1	3.250967000	5.237483000	-4.001523000	8	1.028762000	2.532545000	4.712150000
1	1.624828000	6.591579000	-6.008238000	6	-2.221489000	3.620264000	5.528359000
1	0.091385000	4.638754000	-5.679301000	6	-2.552973000	2.151426000	5.786771000
1	1.765822000	4.118006000	-5.982813000	8	-1.920000000	1.525880000	6.683848000
1	1.107891000	4.066022000	-4.331294000	8	-3.534656000	1.662703000	5.072075000
7	0.844399000	7.261811000	-2.989656000	1	-1.409518000	5.927040000	5.082621000
6	-0.072080000	8.131760000	-2.255037000	1	-0.285402000	3.615753000	6.515525000
6	-0.396180000	9.417425000	-3.036301000	1	-2.645182000	3.929321000	4.571168000
8	-1.509255000	9.981125000	-2.856538000	1	-2.726227000	4.200169000	6.314244000
6	0.487105000	8.479638000	-0.871717000	7	-0.088057000	3.839785000	3.193179000
8	0.273399000	7.326093000	-0.006464000	6	0.768521000	3.401714000	2.079724000
1	1.735957000	7.033654000	-2.553386000	6	2.228800000	3.840532000	2.293329000
1	-1.034657000	7.629751000	-2.116596000	8	3.181788000	3.132601000	1.869236000
1	1.552660000	8.722230000	-0.938650000	6	0.243009000	3.917434000	0.721354000
1	-0.060668000	9.351206000	-0.490247000	6	-1.099005000	3.322230000	0.253133000
1	0.739084000	7.420763000	0.857290000	6	-1.042469000	1.884833000	-0.223141000
7	0.556074000	9.901124000	-3.872975000	8	-0.164040000	1.090083000	0.284157000
6	0.327724000	11.111346000	-4.660930000	8	-1.897196000	1.509504000	-1.119882000
6	-0.735397000	10.999823000	-5.760820000	1	-0.839223000	4.502891000	3.015798000
8	-1.146033000	12.042646000	-6.316319000	1	0.791274000	2.311265000	2.053368000
1	1.417452000	9.382396000	-4.031959000	1	1.010297000	3.685767000	-0.026209000
1	0.002305000	11.928376000	-4.011074000	1	0.147141000	5.009397000	0.743829000
7	-1.181382000	9.748591000	-6.071376000	1	-1.859981000	3.357956000	1.046856000
6	-2.272819000	9.538797000	-7.009784000	1	-1.491046000	3.928450000	-0.568905000
6	-3.520426000	8.935817000	-6.369597000	7	2.435277000	5.022677000	2.935447000
8	-4.361403000	8.325654000	-7.080791000	6	3.773874000	5.483071000	3.295907000

6	4.540736000	4.499503000	4.185479000
8	5.745090000	4.232313000	3.934330000
1	1.654414000	5.634592000	3.141674000
1	4.401817000	5.633081000	2.414548000
7	3.878528000	3.959299000	5.238180000
6	4.528128000	2.949402000	6.071720000
6	4.666121000	1.583559000	5.383937000
8	5.755096000	0.933686000	5.452457000
1	2.949340000	4.292922000	5.486562000
1	5.539690000	3.265417000	6.334650000
7	3.583082000	1.110446000	4.733166000
6	3.649341000	-0.155451000	3.994572000
6	2.555835000	-0.285929000	2.927500000
6	1.188991000	-0.544016000	3.481714000
7	0.050635000	-0.667759000	2.667509000
6	0.785699000	-0.683623000	4.792219000
6	-0.999954000	-0.864526000	3.487328000
7	-0.584977000	-0.883580000	4.770858000
1	2.721755000	1.659849000	4.685881000
1	3.602243000	-0.999508000	4.700000000
1	2.833423000	-1.113930000	2.262895000
1	2.552709000	0.618188000	2.305749000
1	1.320814000	-0.601825000	5.722011000
1	-2.022034000	-0.994879000	3.186098000
6	1.432981000	0.807051000	-4.736664000
6	2.217212000	-0.296791000	-5.409399000
8	1.905137000	-1.514085000	-5.267234000
6	0.174214000	1.225273000	-5.549077000
6	-0.864549000	0.112283000	-5.835862000
6	-1.414482000	-0.574989000	-4.603317000
8	-2.043387000	0.246049000	-3.671855000
8	-1.316164000	-1.777093000	-4.370600000
1	1.127455000	0.438904000	-3.750676000
1	-0.315590000	2.040629000	-5.009505000
1	0.488391000	1.638558000	-6.515915000
1	-0.441130000	-0.673725000	-6.464446000
1	-1.699469000	0.576296000	-6.375504000
7	3.255017000	0.034643000	-6.223080000
6	3.916706000	-0.984386000	-7.053916000
6	4.463391000	-2.183251000	-6.270955000
8	4.172806000	-3.355521000	-6.628699000
1	3.510977000	1.004240000	-6.339906000
1	3.209888000	-1.403355000	-7.774436000
7	5.284249000	-1.931976000	-5.223974000
6	5.924579000	-3.028550000	-4.493045000
6	5.045816000	-3.642357000	-3.396580000
8	5.468570000	-3.722177000	-2.205107000
1	5.531114000	-0.992863000	-4.914142000
1	6.205314000	-3.811086000	-5.207683000
7	3.835472000	-4.086266000	-3.798662000
6	2.827360000	-4.643554000	-2.891831000
6	1.406332000	-4.154974000	-3.213412000
8	1.315057000	-2.702527000	-2.997293000
6	0.364472000	-4.849298000	-2.338785000
1	3.639291000	-4.051937000	-4.800329000
1	3.090547000	-4.350314000	-1.873798000
1	1.180858000	-4.343249000	-4.271898000
1	1.444035000	-2.221075000	-3.863071000
1	-0.643132000	-4.541322000	-2.633282000
1	0.432463000	-5.937289000	-2.448283000
1	0.516337000	-4.595858000	-1.283950000
6	-5.337333000	-4.309850000	3.256999000
6	-6.503436000	-3.634111000	3.963964000
8	-7.596706000	-3.441716000	3.358464000
6	-5.822615000	-5.529347000	2.448319000
6	-4.733413000	-6.184077000	1.553663000
6	-4.190242000	-5.234339000	0.513009000
8	-3.339877000	-4.338149000	0.838920000
7	-4.646668000	-5.308458000	-0.750514000
1	-4.889911000	-3.571906000	2.579151000
1	-6.678637000	-5.223056000	1.837577000
1	-6.192651000	-6.301048000	3.134817000
1	-5.151193000	-7.077672000	1.076356000
1	-3.888700000	-6.504823000	2.173952000
1	-4.273392000	-4.651889000	-1.438239000
1	-5.328704000	-5.994671000	-1.032952000
7	-6.303180000	-3.235198000	5.246384000
6	-7.383202000	-2.627510000	6.025887000
6	-7.929330000	-1.302331000	5.482490000
8	-9.102347000	-0.958038000	5.749940000
1	-5.361588000	-3.266106000	5.641122000
1	-8.241891000	-3.302365000	6.073426000
7	-7.070281000	-0.556076000	4.732170000
6	-7.498253000	0.666354000	4.070527000
6	-7.550578000	0.554326000	2.545174000
8	-7.399132000	1.588542000	1.836333000
1	-6.087553000	-0.822392000	4.625563000
1	-8.503482000	0.904223000	4.438119000
7	-7.804752000	-0.679957000	2.041043000
6	-7.995551000	-0.939001000	0.615038000
6	-6.985719000	-1.930262000	0.041214000
8	-5.639292000	-1.377048000	0.080382000
1	-7.896654000	-1.470928000	2.676967000
1	-7.929043000	0.014414000	0.089155000
1	-7.011604000	-2.861906000	0.619523000
1	-7.254722000	-2.149589000	-0.999426000
1	-5.042047000	-1.621100000	-0.695808000
6	-8.178026000	4.449658000	-4.604213000
6	-7.539186000	3.374177000	-3.751648000
6	-7.750065000	3.325050000	-2.363339000
6	-6.722356000	2.389126000	-4.337391000
6	-7.163784000	2.325482000	-1.575259000
6	-6.133128000	1.387771000	-3.557879000
6	-6.349678000	1.354894000	-2.173615000
1	-8.849287000	4.018219000	-5.357420000
1	-8.766920000	5.143829000	-3.996153000
1	-8.390872000	4.070029000	-1.898216000
1	-6.555495000	2.404999000	-5.411467000
1	-7.348190000	2.285960000	-0.504749000
1	-5.509259000	0.632939000	-4.028469000
1	-5.906686000	0.574147000	-1.563492000
26	-0.157751000	-0.985058000	0.583885000
26	-2.332296000	-0.419212000	-1.628073000
8	0.386717000	-2.903655000	0.974223000
1	1.324074000	-3.110682000	0.740658000
1	-0.165016000	-3.521818000	1.623478000
8	-4.231035000	-0.734699000	4.240035000
1	-3.961553000	0.117798000	4.703922000
1	-4.228535000	-0.579351000	3.240838000
8	-4.140860000	-0.115980000	1.699652000
1	-4.861975000	-0.558430000	1.123737000
1	-4.183870000	0.879489000	1.678840000
8	-2.021926000	-0.961427000	0.442038000
1	-3.117303000	-3.147293000	-0.445426000
1	-2.789724000	-0.630037000	1.019592000
8	0.158405000	-2.122526000	8.063243000
1	0.377399000	-2.834675000	7.373968000
1	0.464670000	-2.340579000	8.956857000
8	-3.805884000	-1.752158000	-1.763789000
8	-3.004691000	-3.021947000	-1.436141000
8	-0.592947000	-1.761082000	-1.507332000
1	0.175275000	-2.051433000	-2.119498000
8	-1.143412000	-4.237522000	2.385899000
1	-1.986197000	-4.327156000	1.875598000
1	-1.306115000	-4.271330000	3.369469000
8	-1.967102000	-1.089203000	7.111106000
1	-1.187195000	-1.426945000	7.670301000
1	-2.091016000	-0.095361000	7.134755000
8	-1.785902000	-4.396772000	4.985107000
1	-2.050016000	-5.301369000	5.232224000
1	-2.523905000	-3.673881000	5.279765000
8	0.518205000	-3.754934000	6.112182000
1	-0.350767000	-4.038189000	5.687797000
1	1.140934000	-3.443940000	5.434057000
8	-3.432693000	-2.678112000	5.714412000
1	-3.758015000	-1.996170000	5.024739000
1	-2.988226000	-2.131746000	6.451139000
1	3.941235000	2.836693000	6.988174000
1	3.663100000	6.441302000	3.811615000
1	0.131173000	8.111959000	6.600325000

1	-1.489700000	9.534731000	2.292459000
1	-5.316638000	7.247077000	3.178764000
1	-5.320420000	9.490391000	-3.805261000
1	-1.978158000	8.872183000	-7.825271000
1	1.275289000	11.401491000	-5.122305000
1	5.765861000	6.959689000	-4.514244000
1	4.705444000	5.331695000	0.215073000
1	8.613583000	-0.607253000	-2.312679000
1	10.746224000	2.830313000	0.459235000
1	6.481447000	2.614767000	3.167287000
1	8.811070000	-0.996988000	4.599367000
1	4.729418000	-0.492838000	-7.596733000
1	6.819307000	-2.634437000	-4.013953000
1	-7.015840000	-2.472094000	7.045069000
1	-6.839310000	1.503223000	4.313854000
1	-4.557203000	-4.598653000	3.969797000
1	-9.002203000	-1.348404000	0.453910000
1	8.441809000	-3.347895000	1.237455000
1	-7.423197000	5.033746000	-5.146353000
1	2.058509000	1.690905000	-4.565040000
1	2.843019000	-5.741132000	-2.948538000
1	-1.259842000	-2.504022000	-1.457789000
1	4.631329000	-0.206130000	3.514184000
1	-1.176659000	-0.995504000	5.622864000
1	-4.389580000	2.550438000	4.269100000

^{1}TS2$_{BP}$:

6	8.638189000	-1.364165000	3.541811000
6	8.022023000	-0.250695000	2.705775000
8	7.987639000	-0.361862000	1.443859000
6	7.748041000	-2.643334000	3.662667000
6	6.993476000	-3.014127000	2.424918000
7	7.555316000	-3.449766000	1.216037000
6	5.648874000	-2.923650000	2.181681000
6	6.592244000	-3.590474000	0.272451000
7	5.429639000	-3.275638000	0.856307000
1	9.582321000	-1.625155000	3.050536000
1	8.384354000	-3.470900000	3.999966000
1	7.007010000	-2.484206000	4.452843000
1	4.845472000	-2.628004000	2.830807000
1	6.696579000	-3.873371000	-0.771542000
1	4.487073000	-3.140399000	0.403065000
7	7.563758000	0.850849000	3.352101000
6	6.887447000	1.949269000	2.651747000
6	7.670639000	2.562242000	1.515444000
8	7.067226000	3.130965000	0.558309000
1	7.395719000	0.843130000	4.361158000
1	5.927226000	1.632315000	2.230972000
7	9.018845000	2.486716000	1.515871000
6	9.802075000	2.977641000	0.379927000
6	9.413895000	2.363075000	-0.972949000
8	9.682979000	2.983443000	-2.022982000
1	9.484195000	2.064391000	2.305988000
1	9.684616000	4.057854000	0.263391000
7	8.797753000	1.139399000	-0.960861000
6	8.327797000	0.538601000	-2.199233000
6	6.832800000	0.702979000	-2.487664000
8	6.331656000	0.105836000	-3.479971000
1	8.609222000	0.651917000	-0.085148000
1	8.871439000	1.002498000	-3.028841000
7	6.115202000	1.499959000	-1.660965000
6	4.686775000	1.754067000	-1.885162000
6	4.318836000	3.242618000	-1.877051000
8	3.377254000	3.649155000	-2.618910000
6	3.754580000	1.031421000	-0.874628000
6	3.937820000	-0.491564000	-0.842062000
6	2.859682000	-1.204710000	-0.034093000
8	1.827512000	-0.535906000	0.328051000
8	3.019331000	-2.466432000	0.251706000
1	6.591292000	1.940607000	-0.875839000
1	4.474018000	1.387968000	-2.891977000
1	2.722697000	1.258962000	-1.160317000
1	3.888906000	1.458557000	0.125626000
1	4.918652000	-0.758679000	-0.429466000
1	3.915831000	-0.912916000	-1.857614000
7	4.965173000	4.052812000	-1.004262000
6	4.448217000	5.369208000	-0.639113000
6	4.307137000	6.368189000	-1.770251000
8	3.426544000	7.266388000	-1.699314000
1	5.652360000	3.667712000	-0.353449000
1	3.454722000	5.289180000	-0.188949000
7	5.136132000	6.267493000	-2.837921000
6	4.975004000	7.176456000	-3.974071000
6	3.565461000	7.137600000	-4.575206000
8	3.079892000	8.168940000	-5.105938000
1	5.840561000	5.543310000	-2.853145000
1	5.153319000	8.212273000	-3.675362000
7	2.895323000	5.955750000	-4.516974000
6	1.549086000	5.836990000	-5.086146000
6	0.511914000	6.688849000	-4.342682000
8	-0.575823000	6.975818000	-4.912114000
6	1.116603000	4.364979000	-5.146286000
1	3.254233000	5.171472000	-3.974276000
1	1.567383000	6.256613000	-6.097632000
1	0.095826000	4.292451000	-5.527820000
1	1.780865000	3.809902000	-5.818015000
1	1.158185000	3.904118000	-4.154404000
7	0.821230000	7.137369000	-3.101182000
6	-0.079605000	8.040593000	-2.391625000
6	-0.391774000	9.312785000	-3.200082000
8	-1.485615000	9.907873000	-3.006358000
6	0.499634000	8.427453000	-1.028003000
8	0.352565000	7.279383000	-0.136304000
1	1.713693000	6.921153000	-2.665750000
1	-1.048142000	7.555758000	-2.233330000
1	1.553760000	8.704339000	-1.125246000
1	-0.071102000	9.283127000	-0.645281000
1	0.840260000	7.407757000	0.709773000
7	0.554444000	9.755033000	-4.065242000
6	0.344270000	10.960769000	-4.863358000
6	-0.744920000	10.870546000	-5.937875000
8	-1.133639000	11.921844000	-6.494028000
1	1.393912000	9.207155000	-4.242696000
1	0.058693000	11.795499000	-4.217233000
7	-1.235533000	9.631751000	-6.230865000
6	-2.341831000	9.458713000	-7.160174000
6	-3.621318000	8.931572000	-6.515705000
8	-4.518273000	8.423137000	-7.237089000
1	-0.867841000	8.798562000	-5.777463000
1	-2.546669000	10.435355000	-7.611796000
7	-3.726798000	9.046028000	-5.162532000
6	-4.908752000	8.580759000	-4.439901000
6	-4.613324000	7.434881000	-3.453046000
6	-4.061749000	6.144080000	-4.025159000
6	-4.153130000	5.804360000	-5.399330000
6	-3.448243000	5.219276000	-3.132887000
6	-3.617714000	4.618850000	-5.869633000
6	-2.919087000	4.027670000	-3.579057000
6	-2.957595000	3.692529000	-4.983880000
8	-2.403746000	2.609850000	-5.429604000
1	-2.966958000	9.458466000	-4.628413000
1	-5.643684000	8.283787000	-5.191879000
1	-5.550491000	7.206448000	-2.917861000
1	-3.911465000	7.804355000	-2.691239000
1	-4.611713000	6.492946000	-6.101366000
1	-3.398638000	5.471039000	-2.076314000
1	-3.660586000	4.362333000	-6.922437000
1	-2.456828000	3.313494000	-2.905081000
1	-3.092607000	-1.247935000	-2.987568000
6	-4.537812000	6.163587000	2.616296000
6	-3.099568000	6.607213000	2.673063000
8	-2.151918000	5.774484000	2.788110000
6	-4.841799000	5.473936000	1.241882000
6	-5.620084000	4.142231000	1.372965000
6	-4.783391000	3.052497000	1.992036000
8	-4.104663000	2.241651000	1.311651000
8	-4.770563000	3.073497000	3.332985000
1	-4.691972000	5.434808000	3.418117000
1	-5.426951000	6.155073000	0.614907000
1	-3.904075000	5.277934000	0.707260000

1	-5.927937000	3.807084000	0.381712000
1	-6.521385000	4.272352000	1.975634000
7	-2.814144000	7.938405000	2.557820000
6	-1.452823000	8.379780000	2.216431000
6	-0.375361000	7.910265000	3.190176000
8	0.729138000	7.479254000	2.755994000
1	-3.573890000	8.588803000	2.412869000
1	-1.160445000	7.982235000	1.238374000
7	-0.627241000	8.015688000	4.521650000
6	0.362841000	7.602703000	5.519496000
6	0.508534000	6.079880000	5.662594000
8	1.585075000	5.591869000	6.088834000
1	-1.512936000	8.398548000	4.822489000
1	1.346577000	7.993934000	5.252764000
7	-0.581080000	5.350172000	5.334302000
6	-0.634064000	3.876270000	5.373447000
6	0.262253000	3.305117000	4.253612000
8	1.169363000	2.475363000	4.526343000
6	-2.124902000	3.491799000	5.286534000
6	-2.472676000	2.027029000	5.554270000
8	-1.961453000	1.426809000	6.540878000
8	-3.366646000	1.518134000	4.739952000
1	-1.331646000	5.808480000	4.831311000
1	-0.204862000	3.519622000	6.311364000
1	-2.526615000	3.791094000	4.317271000
1	-2.648738000	4.077210000	6.055433000
7	0.086844000	3.798263000	2.997757000
6	0.999401000	3.390939000	1.919220000
6	2.431439000	3.895741000	2.180850000
8	3.425499000	3.217325000	1.804657000
6	0.502240000	3.827033000	0.519397000
6	-0.803242000	3.155659000	0.006251000
6	-0.748334000	1.645144000	-0.046612000
8	-0.436332000	1.017387000	1.010039000
8	-1.035859000	1.035732000	-1.187881000
1	-0.673495000	4.445566000	2.800987000
1	1.080435000	2.304457000	1.938866000
1	1.308386000	3.607798000	-0.191272000
1	0.347897000	4.912817000	0.484108000
1	-1.646496000	3.412393000	0.660949000
1	-1.025505000	3.543885000	-0.990073000
7	2.572229000	5.087142000	2.821881000
6	3.884413000	5.602395000	3.206964000
6	4.660016000	4.687955000	4.160696000
8	5.879768000	4.458761000	3.951421000
1	1.765638000	5.678238000	2.990703000
1	4.533990000	5.732476000	2.338979000
7	3.994913000	4.176212000	5.227027000
6	4.684630000	3.279083000	6.153398000
6	4.881974000	1.859991000	5.604053000
8	6.004229000	1.278476000	5.702354000
1	3.048586000	4.484685000	5.446075000
1	5.681410000	3.662464000	6.380163000
7	3.808077000	1.263570000	5.040115000
6	3.926128000	-0.078832000	4.459517000
6	2.801621000	-0.382980000	3.465083000
6	1.480708000	-0.697815000	4.094698000
7	0.340506000	-0.946078000	3.318419000
6	1.112741000	-0.782055000	5.419925000
6	-0.680357000	-1.155415000	4.169743000
7	-0.242623000	-1.069851000	5.439646000
1	2.926226000	1.767044000	4.921836000
1	3.953848000	-0.832789000	5.259941000
1	3.108844000	-1.233507000	2.841401000
1	2.695340000	0.467088000	2.780256000
1	1.671758000	-0.610129000	6.322504000
1	-1.706962000	-1.332055000	3.899887000
6	1.409267000	0.155050000	-4.222401000
6	2.223731000	-0.655103000	-5.206598000
8	2.023576000	-1.900035000	-5.381799000
6	0.118979000	0.786093000	-4.820897000
6	-0.851319000	-0.152847000	-5.552855000
6	-1.627975000	-1.168375000	-4.747566000
8	-2.038514000	-0.768335000	-3.479787000
8	-1.988325000	-2.257451000	-5.194359000

1	1.150228000	-0.512108000	-3.394535000
1	-0.413614000	1.292072000	-4.011397000
1	0.407350000	1.567821000	-5.532467000
1	-0.363854000	-0.702540000	-6.363617000
1	-1.610928000	0.501275000	-6.004785000
7	3.190485000	-0.032251000	-5.923601000
6	3.962127000	-0.740840000	-6.956313000
6	4.764762000	-1.943946000	-6.440017000
8	4.895548000	-2.959924000	-7.154575000
1	3.337676000	0.958560000	-5.793796000
1	3.294717000	-1.136899000	-7.725344000
7	5.307941000	-1.821128000	-5.193888000
6	6.043356000	-2.904820000	-4.557409000
6	5.241885000	-3.616777000	-3.465373000
8	5.812441000	-4.106820000	-2.446633000
1	5.343434000	-0.929065000	-4.707887000
1	6.312423000	-3.637133000	-5.327668000
7	3.913036000	-3.732233000	-3.676790000
6	3.031866000	-4.489954000	-2.779476000
6	1.633428000	-4.666354000	-3.384017000
8	0.974334000	-3.361486000	-3.504281000
6	0.744767000	-5.560539000	-2.524802000
1	3.504498000	-3.262346000	-4.478984000
1	2.943325000	-3.976109000	-1.814196000
1	1.714844000	-5.082783000	-4.396199000
1	1.235431000	-2.838306000	-4.320482000
1	-0.258159000	-5.606610000	-2.956754000
1	1.152394000	-6.575653000	-2.474359000
1	0.664697000	-5.170275000	-1.503540000
6	-5.230435000	-4.268341000	3.034631000
6	-6.415224000	-3.609263000	3.723832000
8	-7.490001000	-3.406018000	3.087896000
6	-5.665738000	-5.555693000	2.306104000
6	-4.571350000	-6.154351000	1.379245000
6	-4.179853000	-5.193878000	0.281531000
8	-3.289936000	-4.300072000	0.488315000
7	-4.827404000	-5.259318000	-0.896008000
1	-4.841127000	-3.546273000	2.305550000
1	-6.570810000	-5.338992000	1.728282000
1	-5.938422000	-6.322706000	3.041411000
1	-4.935140000	-7.095744000	0.952183000
1	-3.668977000	-6.378134000	1.957943000
1	-4.597829000	-4.585158000	-1.624118000
1	-5.530005000	-5.958988000	-1.077999000
7	-6.252181000	-3.237693000	5.018053000
6	-7.346092000	-2.626372000	5.773441000
6	-7.842672000	-1.276137000	5.245494000
8	-9.004146000	-0.897244000	5.514287000
1	-5.322472000	-3.283279000	5.440279000
1	-8.220283000	-3.282729000	5.772922000
7	-6.959776000	-0.546525000	4.503838000
6	-7.357227000	0.697459000	3.863531000
6	-7.467652000	0.605877000	2.340999000
8	-7.361553000	1.652485000	1.643590000
1	-5.987212000	-0.842508000	4.392419000
1	-8.338624000	0.975732000	4.265134000
7	-7.710283000	-0.622465000	1.818013000
6	-7.909564000	-0.846379000	0.387218000
6	-7.004873000	-1.949882000	-0.155159000
8	-5.592982000	-1.568622000	-0.106837000
1	-7.789313000	-1.427936000	2.437506000
1	-7.721865000	0.098424000	-0.127568000
1	-7.116196000	-2.853076000	0.451776000
1	-7.281727000	-2.177840000	-1.190932000
1	-5.147165000	-1.454962000	-0.981349000
6	-10.763942000	5.349913000	-3.149772000
6	-9.487045000	4.603411000	-2.828789000
6	-9.343404000	3.904653000	-1.617804000
6	-8.422303000	4.570802000	-3.747677000
6	-8.176803000	3.184631000	-1.329538000
6	-7.252910000	3.854954000	-3.468523000
6	-7.127547000	3.159802000	-2.258490000
1	-11.466126000	4.715115000	-3.707390000
1	-11.276705000	5.678031000	-2.239313000
1	-10.154076000	3.922947000	-0.893889000

1	-8.513764000	5.106863000	-4.688755000
1	-8.082207000	2.655705000	-0.384883000
1	-6.444054000	3.840946000	-4.192472000
1	-6.219456000	2.603804000	-2.041712000
26	0.026460000	-1.007229000	1.299146000
26	-1.543539000	-0.840277000	-1.499093000
8	0.454006000	-3.037093000	0.785361000
1	1.445329000	-3.127561000	0.760426000
1	-0.130075000	-3.658398000	1.410760000
8	-4.080253000	-0.866738000	4.027832000
1	-3.802036000	0.014635000	4.442926000
1	-4.025548000	-0.793015000	3.023949000
8	-3.933716000	-0.414847000	1.449641000
1	-4.707559000	-0.830647000	0.932492000
1	-3.952038000	0.586625000	1.411372000
8	-1.777207000	-1.246089000	0.325138000
1	-3.205554000	-3.118383000	-0.785750000
1	-2.635024000	-0.956474000	0.825659000
8	-0.833773000	-2.615985000	8.812352000
1	-0.478601000	-3.254761000	8.114234000
1	-0.919036000	-3.013335000	9.691935000
8	-3.613268000	-1.490131000	-1.926646000
8	-3.288225000	-2.968961000	-1.783358000
8	-0.120011000	-2.177007000	-1.611703000
1	0.294618000	-2.675143000	-2.432195000
8	-1.165686000	-4.279209000	2.188970000
1	-2.007565000	-4.299713000	1.667517000
1	-1.306727000	-4.290840000	3.175835000
8	-2.200852000	-1.167045000	7.285157000
1	-1.754614000	-1.583954000	8.099489000
1	-2.286953000	-0.176153000	7.230793000
8	-1.776462000	-4.439713000	4.848661000
1	-2.088622000	-5.352650000	4.994816000
1	-2.513155000	-3.723648000	5.168824000
8	-0.000270000	-4.058139000	6.822219000
1	-0.611066000	-4.204362000	6.039034000
1	0.923898000	-3.977223000	6.536989000
8	-3.417834000	-2.733314000	5.615581000
1	-3.699718000	-2.049931000	4.901426000
1	-3.057570000	-2.194167000	6.401480000
1	4.101305000	3.233221000	7.078175000
1	3.727901000	6.579032000	3.674603000
1	0.079837000	8.024416000	6.488412000
1	-1.452988000	9.473752000	2.172621000
1	-5.222885000	7.001496000	2.783997000
1	-5.338798000	9.415501000	-3.872583000
1	-2.081611000	8.763360000	-7.963577000
1	1.289617000	11.215265000	-5.349804000
1	5.707519000	6.906899000	-4.740511000
1	5.125759000	5.782463000	0.114463000
1	8.545585000	-0.533582000	-2.212977000
1	10.858077000	2.770956000	0.576695000
1	6.664759000	2.743528000	3.376303000
1	8.879824000	-1.018254000	4.551597000
1	4.639957000	-0.019057000	-7.421883000
1	6.954114000	-2.514196000	-4.101650000
1	-7.014253000	-2.504185000	6.809037000
1	-6.655398000	1.503825000	4.090380000
1	-4.422363000	-4.478236000	3.743069000
1	-8.953450000	-1.133452000	0.196854000
1	8.541262000	-3.567786000	1.040666000
1	-10.568769000	6.233881000	-3.766739000
1	2.016645000	0.969760000	-3.807556000
1	3.482981000	-5.471455000	-2.592881000
1	0.051837000	-2.654587000	-0.732933000
1	4.891485000	-0.136183000	3.946356000
1	-0.883575000	-1.148130000	6.257325000
1	-4.155634000	2.382863000	3.840587000

$^{1}P_{rBP}$:

6	8.580961000	-2.919372000	2.291186000
6	7.860549000	-1.720015000	1.693491000
8	7.739306000	-1.632362000	0.434927000
6	7.667403000	-4.188038000	2.378489000
6	6.697232000	-4.333463000	1.247131000
7	7.044104000	-4.430653000	-0.107001000
6	5.327213000	-4.296815000	1.264689000
6	5.937671000	-4.419810000	-0.883415000
7	4.885118000	-4.348162000	-0.053545000
1	9.437608000	-3.125405000	1.641646000
1	8.311878000	-5.071798000	2.462983000
1	7.080371000	-4.143786000	3.301374000
1	4.645549000	-4.227021000	2.093145000
1	5.902985000	-4.409410000	-1.977349000
1	3.880953000	-4.210098000	-0.321669000
7	7.353931000	-0.795827000	2.545158000
6	6.449916000	0.281233000	2.125529000
6	6.846034000	1.044016000	0.888586000
8	5.947913000	1.470756000	0.107680000
1	7.341260000	-0.959197000	3.549867000
1	5.439879000	-0.094322000	1.936610000
7	8.142896000	1.307043000	0.612429000
6	8.493655000	2.060019000	-0.596034000
6	7.837738000	1.522506000	-1.876116000
8	7.489684000	2.320549000	-2.782225000
1	8.866799000	0.988320000	1.239125000
1	8.181434000	3.104107000	-0.511059000
7	7.689086000	0.173224000	-1.992350000
6	7.118952000	-0.408807000	-3.205999000
6	5.619100000	-0.175903000	-3.414333000
8	5.097043000	-0.450132000	-4.526032000
1	7.878138000	-0.434660000	-1.196512000
1	7.617818000	0.003351000	-4.087405000
7	4.914153000	0.287130000	-2.353135000
6	3.494633000	0.647259000	-2.461292000
6	3.312023000	2.166117000	-2.281883000
8	2.661714000	2.848603000	-3.123652000
6	2.618806000	-0.154918000	-1.472874000
6	2.749306000	-1.675737000	-1.670342000
6	1.962595000	-2.480570000	-0.655008000
8	0.918244000	-1.904339000	-0.141969000
8	2.324335000	-3.676225000	-0.338226000
1	5.407968000	0.514230000	-1.492595000
1	3.201942000	0.426308000	-3.489599000
1	1.573968000	0.139857000	-1.616073000
1	2.883242000	0.109715000	-0.441503000
1	3.799181000	-1.984097000	-1.628580000
1	2.384392000	-1.972625000	-2.665876000
7	3.897102000	2.711630000	-1.190542000
6	3.815633000	4.130512000	-0.873592000
6	4.470340000	5.070150000	-1.877380000
8	4.117840000	6.286874000	-1.885284000
1	4.417905000	2.136419000	-0.531121000
1	2.772756000	4.452603000	-0.777431000
7	5.374031000	4.583199000	-2.754390000
6	5.902416000	5.433407000	-3.822906000
6	4.825114000	6.057462000	-4.719188000
8	5.087576000	7.100735000	-5.356654000
1	5.757880000	3.647047000	-2.659560000
1	6.469755000	6.275451000	-3.416741000
7	3.606792000	5.435616000	-4.774552000
6	2.485318000	6.037250000	-5.502553000
6	1.294603000	6.283593000	-4.559197000
8	0.104874000	6.211274000	-4.969441000
6	2.071508000	5.220301000	-6.733277000
1	3.446251000	4.555042000	-4.291191000
1	2.857490000	7.016482000	-5.835469000
1	1.218610000	5.687003000	-7.231321000
1	2.911467000	5.167350000	-7.431108000
1	1.781362000	4.203689000	-6.451541000
7	1.581714000	6.627818000	-3.266961000
6	0.536512000	6.589038000	-2.234169000
6	-0.618553000	7.559642000	-2.478407000
8	-1.746252000	7.322286000	-1.964167000
6	1.128173000	6.806211000	-0.820997000
8	0.880248000	5.579962000	-0.062728000
1	2.552700000	6.610501000	-2.947823000
1	0.071379000	5.597163000	-2.229210000
1	2.203135000	6.999378000	-0.881269000
1	0.638265000	7.655101000	-0.327915000

1	1.053788000	5.701906000	0.903779000
7	-0.363395000	8.667805000	-3.213861000
6	-1.407531000	9.638118000	-3.533270000
6	-2.547373000	9.116149000	-4.419454000
8	-3.611671000	9.770830000	-4.473700000
1	0.558070000	8.768527000	-3.617160000
1	-1.884034000	9.996553000	-2.617054000
7	-2.327960000	7.958829000	-5.107655000
6	-3.387635000	7.337308000	-5.892529000
6	-3.889621000	6.009149000	-5.325803000
8	-4.584208000	5.246631000	-6.047382000
1	-1.432431000	7.474509000	-5.055638000
1	-4.223805000	8.042605000	-5.937863000
7	-3.545672000	5.723783000	-4.041259000
6	-3.907829000	4.466058000	-3.396508000
6	-2.697456000	3.548644000	-3.131510000
6	-1.898732000	3.076625000	-4.327943000
6	-2.478646000	2.874246000	-5.611319000
6	-0.525517000	2.750750000	-4.144073000
6	-1.749069000	2.297648000	-6.633745000
6	0.218064000	2.169365000	-5.148360000
6	-0.387006000	1.868738000	-6.424412000
8	0.249905000	1.197910000	-7.335813000
1	-3.006086000	6.395333000	-3.503135000
1	-4.648414000	3.969023000	-4.024672000
1	-3.080270000	2.653838000	-2.610691000
1	-2.018259000	4.050044000	-2.428415000
1	-3.494650000	3.201298000	-5.800150000
1	-0.047032000	2.963631000	-3.193531000
1	-2.184110000	2.127886000	-7.612383000
1	1.266215000	1.941824000	-4.991331000
1	-3.526996000	-4.683286000	-2.525971000
6	-4.641332000	4.698622000	2.353630000
6	-3.180471000	5.061001000	2.442432000
8	-2.287843000	4.187709000	2.647539000
6	-4.951299000	3.953580000	1.009439000
6	-5.771798000	2.651703000	1.193000000
6	-4.967534000	1.578609000	1.878948000
8	-4.266686000	0.745456000	1.253300000
8	-4.998924000	1.644137000	3.219555000
1	-4.865135000	4.027903000	3.189178000
1	-5.507733000	4.622487000	0.344424000
1	-4.015822000	3.703894000	0.494591000
1	-6.066685000	2.269100000	0.214395000
1	-6.677169000	2.842714000	1.774309000
7	-2.819619000	6.368435000	2.266493000
6	-1.422958000	6.727116000	1.985974000
6	-0.431802000	6.280702000	3.057502000
8	0.693177000	5.807980000	2.725193000
1	-3.533907000	7.039424000	2.018954000
1	-1.090965000	6.275412000	1.045413000
7	-0.756884000	6.455580000	4.362936000
6	0.180486000	6.075907000	5.428918000
6	0.317150000	4.554332000	5.614721000
8	1.426360000	4.043309000	5.901905000
1	-1.644998000	6.878508000	4.595533000
1	1.177483000	6.456138000	5.200448000
7	-0.828889000	3.845309000	5.464099000
6	-0.915408000	2.372376000	5.534790000
6	0.004170000	1.750109000	4.462713000
8	0.862561000	0.880075000	4.763815000
6	-2.412322000	2.026304000	5.369078000
6	-2.792285000	0.557465000	5.562806000
8	-2.328775000	-0.072666000	6.554000000
8	-3.640632000	0.081521000	4.683769000
1	-1.636196000	4.323808000	5.087648000
1	-0.546081000	2.016670000	6.498598000
1	-2.761189000	2.365730000	4.391142000
1	-2.960585000	2.592848000	6.135355000
7	-0.137866000	2.234396000	3.200446000
6	0.701891000	1.738997000	2.106681000
6	2.172033000	2.152647000	2.270284000
8	3.092691000	1.395355000	1.858328000
6	0.152620000	2.198637000	0.739467000
6	-1.239474000	1.619213000	0.380402000
6	-1.228893000	0.130786000	0.152449000
8	-1.018947000	-0.622331000	1.203329000
8	-1.417651000	-0.336077000	-1.026217000
1	-0.864477000	2.916678000	2.992423000
1	0.707326000	0.648532000	2.146258000
1	0.881723000	1.915457000	-0.026569000
1	0.083318000	3.292184000	0.706835000
1	-1.957539000	1.830352000	1.176544000
1	-1.599405000	2.090483000	-0.536721000
7	2.412840000	3.353461000	2.854647000
6	3.761636000	3.824674000	3.148268000
6	4.554645000	2.881146000	4.052074000
8	5.772604000	2.670425000	3.817408000
1	1.641903000	3.968709000	3.085798000
1	4.360905000	3.927565000	2.240311000
7	3.897343000	2.304969000	5.090068000
6	4.571993000	1.297881000	5.910642000
6	4.687631000	-0.068710000	5.215791000
8	5.805324000	-0.648381000	5.092081000
1	2.946698000	2.587615000	5.313545000
1	5.589859000	1.621044000	6.134385000
7	3.533981000	-0.619922000	4.764581000
6	3.553170000	-1.911454000	4.067809000
6	2.360955000	-2.080604000	3.120205000
6	1.052923000	-2.287299000	3.819952000
7	-0.160657000	-2.334146000	3.124528000
6	0.757382000	-2.430826000	5.158745000
6	-1.148227000	-2.490643000	4.023657000
7	-0.616513000	-2.563660000	5.259325000
1	2.656891000	-0.098858000	4.803406000
1	3.583902000	-2.733310000	4.797979000
1	2.555676000	-2.939453000	2.461612000
1	2.308282000	-1.196537000	2.470633000
1	1.391966000	-2.404152000	6.026430000
1	-2.204356000	-2.535420000	3.814548000
6	-0.113702000	-1.520213000	-5.458834000
6	0.963801000	-2.139419000	-6.318983000
8	1.262036000	-3.382172000	-6.216359000
6	-1.545422000	-1.669706000	-6.059970000
6	-2.226046000	-3.018683000	-5.779276000
6	-2.616563000	-3.240813000	-4.326810000
8	-2.443717000	-2.234537000	-3.493182000
8	-3.073840000	-4.367063000	-3.963025000
1	-0.082221000	-1.991442000	-4.473328000
1	-2.162421000	-0.869641000	-5.641136000
1	-1.498769000	-1.501964000	-7.142385000
1	-1.591448000	-3.861842000	-6.080447000
1	-3.142453000	-3.108193000	-6.378143000
7	1.585532000	-1.343465000	-7.208485000
6	2.499376000	-1.867547000	-8.232716000
6	3.683455000	-2.692852000	-7.720855000
8	4.077307000	-3.680065000	-8.379327000
1	1.274355000	-0.366392000	-7.281375000
1	1.965674000	-2.527006000	-8.922807000
7	4.282164000	-2.291546000	-6.558874000
6	5.390933000	-3.036805000	-5.978539000
6	4.992396000	-3.806490000	-4.722274000
8	5.816422000	-3.977808000	-3.770592000
1	4.039329000	-1.425584000	-6.092144000
1	5.739434000	-3.751926000	-6.732831000
7	3.747411000	-4.327817000	-4.709965000
6	3.269124000	-5.199012000	-3.633776000
6	1.860163000	-5.729628000	-3.918892000
8	0.916953000	-4.611469000	-3.965405000
6	1.392496000	-6.703003000	-2.840760000
1	3.107209000	-4.081130000	-5.462715000
1	3.238813000	-4.650822000	-2.684110000
1	1.843999000	-6.222043000	-4.900446000
1	0.911418000	-4.157049000	-4.868597000
1	0.376901000	-7.044482000	-3.060164000
1	2.046650000	-7.580210000	-2.797093000
1	1.395384000	-6.216193000	-1.858787000
6	-5.761373000	-6.049410000	3.575857000
6	-6.891940000	-5.129774000	4.014373000
8	-7.893239000	-4.919132000	3.273859000

6	-6.284515000	-7.273152000	2.800829000
6	-5.164902000	-8.288646000	2.441826000
6	-3.989463000	-7.608911000	1.773529000
8	-2.958860000	-7.299919000	2.460870000
7	-4.104383000	-7.278169000	0.478618000
1	-5.084260000	-5.466059000	2.936324000
1	-6.796660000	-6.929815000	1.895765000
1	-7.039226000	-7.796919000	3.398753000
1	-5.576975000	-9.070308000	1.792401000
1	-4.790234000	-8.771396000	3.348461000
1	-3.420187000	-6.667236000	0.019551000
1	-4.925614000	-7.529663000	-0.050689000
7	-6.747094000	-4.554226000	5.237671000
6	-7.822324000	-3.781983000	5.858564000
6	-8.226553000	-2.489205000	5.146729000
8	-9.367482000	-2.010704000	5.333508000
1	-5.833083000	-4.605419000	5.690620000
1	-8.733453000	-4.384247000	5.914156000
7	-7.288162000	-1.916150000	4.340701000
6	-7.594436000	-0.744208000	3.537348000
6	-7.600892000	-1.014141000	2.033073000
8	-7.385230000	-0.067180000	1.226763000
1	-6.336863000	-2.281719000	4.284301000
1	-8.591990000	-0.398512000	3.833308000
7	-7.881576000	-2.285922000	1.648001000
6	-8.046444000	-2.675547000	0.248382000
6	-7.129147000	-3.819667000	-0.167416000
8	-5.744073000	-3.357637000	-0.211865000
1	-8.038081000	-3.000834000	2.357433000
1	-7.855011000	-1.796045000	-0.369918000
1	-7.220196000	-4.650754000	0.543447000
1	-7.421175000	-4.166215000	-1.165860000
1	-5.142460000	-3.944822000	-0.739339000
6	-7.374900000	2.859023000	-5.349358000
6	-6.906269000	1.921437000	-4.255860000
6	-7.590142000	1.826976000	-3.031121000
6	-5.767097000	1.113393000	-4.442137000
6	-7.154635000	0.957153000	-2.021891000
6	-5.322731000	0.244672000	-3.438011000
6	-6.016083000	0.166028000	-2.221965000
1	-7.590547000	2.311257000	-6.275603000
1	-8.289803000	3.383280000	-5.055492000
1	-8.476509000	2.435593000	-2.872386000
1	-5.232047000	1.163267000	-5.387159000
1	-7.691755000	0.881537000	-1.081381000
1	-4.444782000	-0.374193000	-3.603345000
1	-5.684544000	-0.506142000	-1.437510000
26	-0.479751000	-2.465542000	1.171515000
26	-1.689301000	-2.203770000	-1.709952000
8	0.177055000	-4.197268000	1.230882000
1	0.955100000	-4.326777000	0.628971000
1	-0.296953000	-5.015868000	1.965921000
8	-4.412904000	-2.279781000	3.854420000
1	-4.133614000	-1.403966000	4.274953000
1	-4.361447000	-2.226252000	2.853811000
8	-4.218457000	-1.912264000	1.205509000
1	-4.967650000	-2.387434000	0.694112000
1	-4.273464000	-0.914343000	1.179288000
8	-2.097014000	-2.892976000	0.208238000
1	-2.465689000	-4.342186000	-0.218315000
1	-2.944884000	-2.455901000	0.646347000
8	-1.549731000	-4.267250000	8.885416000
1	-1.296444000	-5.057663000	8.298852000
1	-1.883284000	-4.536278000	9.754864000
8	-3.874282000	-4.841128000	-1.556202000
8	-2.629191000	-5.207404000	-0.768151000
8	-0.594985000	-3.919016000	-2.042742000
1	0.036744000	-4.174568000	-2.806260000
8	-0.892224000	-5.776544000	2.661006000
1	-1.578280000	-6.399564000	2.280037000
1	-1.162980000	-5.621689000	3.620670000
8	-2.437806000	-2.666970000	7.176189000
1	-2.126269000	-3.151570000	8.024486000
1	-2.553825000	-1.676928000	7.175327000
8	-2.199317000	-5.940100000	4.894844000

1	-2.628966000	-6.693927000	4.432029000
1	-2.907433000	-5.201604000	5.144705000
8	-0.995219000	-6.158845000	7.236408000
1	-1.436175000	-6.161377000	6.331688000
1	-0.217859000	-6.737155000	7.268234000
8	-3.842398000	-4.143178000	5.569692000
1	-4.104393000	-3.498894000	4.832844000
1	-3.410437000	-3.599171000	6.314436000
1	4.014083000	1.188672000	6.845148000
1	3.671723000	4.806901000	3.621042000
1	-0.159700000	6.528950000	6.364693000
1	-1.372067000	7.815692000	1.879089000
1	-5.276510000	5.585170000	2.453463000
1	-4.390460000	4.679073000	-2.435024000
1	-3.055446000	7.141544000	-6.916159000
1	-0.943273000	10.495713000	-4.028232000
1	6.579486000	4.824168000	-4.427731000
1	4.295262000	4.276647000	0.098696000
1	7.284668000	-1.490903000	-3.193920000
1	9.581621000	2.035015000	-0.710708000
1	6.371250000	1.001746000	2.949355000
1	8.971071000	-2.703380000	3.290680000
1	2.874620000	-1.011094000	-8.801556000
1	6.202231000	-2.360802000	-5.715559000
1	-7.517076000	-3.541326000	6.881763000
1	-6.885544000	0.065701000	3.724810000
1	-5.178844000	-6.365787000	4.446943000
1	-9.084071000	-2.993139000	0.077908000
1	7.982794000	-4.373285000	-0.471214000
1	-6.617652000	3.615885000	-5.592561000
1	0.098829000	-0.459994000	-5.316335000
1	3.964761000	-6.039761000	-3.516485000
1	-1.111772000	-4.691857000	-1.712779000
1	4.487700000	-1.963901000	3.500543000
1	-1.214721000	-2.656045000	6.120929000
1	-4.399893000	0.965878000	3.757502000
